# A revision of *Xylopia* L. (Annonaceae): the species of Tropical Africa

**DOI:** 10.3897/phytokeys.97.20975

**Published:** 2018-04-24

**Authors:** David M. Johnson, Nancy A. Murray

**Affiliations:** 1 Department of Botany-Microbiology, Ohio Wesleyan University, Delaware, OH, 43015,USA Ohio Wesleyan University Delaware United States of America

**Keywords:** *Xylopia*, pantropical Annonaceae, Tropical Africa, long distance dispersal, bird/monkey syndrome, *X.aethiopica*, conservation, new species

## Abstract

A revision of the 45 species of the pantropical genus *Xylopia* in Tropical Africa includes descriptions of six new species and a new section of the genus. The fruits and seeds of *Xylopia* show specializations that promote vertebrate dispersal, primarily by hornbills and monkeys. Over half of the African species have an Area of Occupancy (AOO) less than 80 km^2^, suggesting that they are in need of protection. African species are classified into five sections. Section Neoxylopia , with four species, is centered in the Guineo-Congolian Region and includes *X.globosa***sp. nov.** Section Ancistropetala, with three species, occurs in the same region. Both of these sections are endemic to Africa. Section Xylopia, which extends to Madagascar and the American tropics, has only a single species in Africa, *X.aethiopica*. The three species of section Verdcourtia**sect. nov.** are restricted to the East African coast and Madagascar. The largest number of African species, (34) belong to section Stenoxylopia, in which the seeds lack the arils found in the other sections and instead have a fleshy sarcotesta. Section Stenoxylopia is divided into two informal groups, one centered in eastern and southern Africa (*X.odoratissima* group) and the other centered in the wetter forests of western and central Africa (*X.acutiflora* group). Five new species are described in section Stenoxylopia: *Xylopianilotica***sp. nov.** from Sudan, South Sudan, and Uganda, *Xylopiacalva***sp. nov.** from Nigeria and Cameroon, which is allied to *X.phloiodora*, and *Xylopiamonticola***sp. nov.** from Nigeria and Cameroon, *X.piratae***sp. nov.** from Ivory Coast and Ghana, and *X.unguiculata***sp. nov.** from Gabon. The latter three species are segregates of the former *Xylopiaacutiflora* s. l. One new combination is made at the species level, *X.shirensis***comb. nov.** Keys, descriptions, illustrations, distribution maps, and an index to numbered collections document diversity and assist with species identification. The name *Unonaoliveriana* Baill. was found to pre-date the name *Unonalepidota* Oliv., requiring the combination *Meiocarpidiumoliverianum***comb. nov.**

## Introduction

*Xylopia* L. is unique within the early-divergent angiosperm family Annonaceae in its pantropical distribution and, with 160–180 species worldwide, it is the second largest genus in the family. The plants bear distinctive fruits composed of clusters of fingerlike monocarps, which dehisce when ripe, displaying their seeds against an endocarp of contrasting color. In Africa, birds and monkeys play an important role in the dispersal of the seeds (Whitney et al. 1998, Poulsen et al. 2001, Koné et al. 2008), underscoring the interdependence of the plants and their vertebrate dispersers in tropical forest systems. The fruits of one widespread African species, *Xylopiaaethiopica* (Dun.) A. Rich., are the source of a spice and medicine, which has been used since antiquity (Avicenna 1544, Matthioli 1565, Dunal 1817, Burkill 1985).

*Xylopia* is classified, together with its sister group *Artabotrys* R. Br., in the tribe Xylopieae of subfamily Annonoideae (Chatrou et al. 2012). Evidence from four molecular markers and seed morphology data points to the origin and early diversification of *Xylopia* in tropical Africa (Thomas et al. 2015, Stull et al. 2017). Thomas et al. (2015) also provided a divergence time estimate for the separation of *Xylopia* and *Artabotrys* at 54.6–63.2 million years ago (Mya), as well as evidence of rapid radiation of *Xylopia* into four clades at about 30 Mya, 27 Mya, and 23 Mya, respectively. These divergence times post-date the breakup of the Gondwanan supercontinent, suggesting that the pantropical distribution of *Xylopia* was established by long-distance dispersal rather than by tectonic movement of major Southern Hemisphere landmasses (Stull et al. 2017).

**Figure 1.**
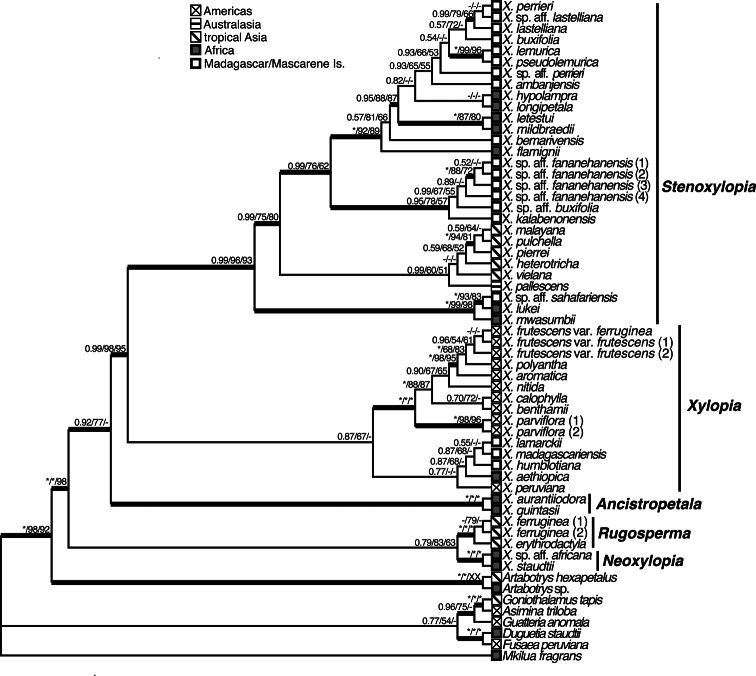
Maximum clade credibility tree for *Xylopia*. Tree obtained from Bayesian analysis of the four-marker data set for 44 *Xylopia* species, from Stull et al. (2017), showing geographic distribution, major clades, and relationship of named sections. Species of the first diverging subclade of the Stenoxylopia clade, *X.mwasumbii*, *X.lukei*, and X.sp. aff.sahafariensis, are placed in this revision into a new section, sect. Verdcourtia. Figure reproduced with permission of the American Society of Plant Taxonomists.

Species from all four major clades are present in Africa, concomitant with the widest range of morphological and ecological diversity found in the genus. Included in the first diverging clade (Fig. 1) of *Xylopia* are species with brushlike arils, which comprise section Neoxylopia Engl. & Diels. Diverging next are all species with fimbriate arils, which comprise section Ancistropetala (Engl. & Diels) D. M. Johnson & N. A. Murray. Both of these sections are exclusively African. The remaining species are divided into a clade with bilobed arils and a clade with various aril types or lacking arils entirely. Bilobed arils define section Xylopia, a section present in tropical America, Madagascar, the Mascarene Islands, and with a single species, *X.aethiopica*, present in Africa. Species of the fourth clade, which were placed in section Stenoxylopia Engl. & Diels, are present in Africa, Madagascar, and Australasia, and form the largest African group (Stull et al. 2017).

In 1901, Engler and Diels recognized 18 African species of *Xylopia*, which they classified among four sections primarily on the basis of fruit and seed characters. Development of *Xylopia* taxonomy in Africa then proceeded through floras, e.g. Davy (1926), Andrews (1950), Boutique (1951b), Tisserant and Sillans (1953), Keay (1954–1958), Robson (1960), Paiva (1966), Le Thomas (1969), and Verdcourt (1971b). These works provided descriptions and identification keys for plants in most of the individual African countries where *Xylopia* occurs, but did not agree in their taxonomic concepts. We have made incremental contributions to the taxonomy through descriptions of new species (e.g. Johnson et al. 1999, 2017) and through a new infrageneric classification (Stull et al. 2017). This revision of tropical African species has two goals: to integrate and update taxonomic understanding of *Xylopia* in Africa and to provide a framework for the study of diversity and biology of *Xylopia* across its global distribution.

## Taxonomic history

*Xylopia* was initially conceived as an exclusively American group. The name *Xylopicrum*, an appellation denoting bitter wood, was proposed by Patrick Browne in 1756 for two species of trees from Jamaica. In 1759, Linnaeus shortened the generic name to *Xylopia* and proposed the names *Xylopiamuricata* L. and *Xylopiaglabra* L. for these Jamaican species. Adanson (1763) returned to Browne’s form of the generic name, with the orthographic change to *Xylopicron*, and Crantz (1766) subsequently made combinations using the same epithets but Adanson’s form of the name.

**Figure 2.**
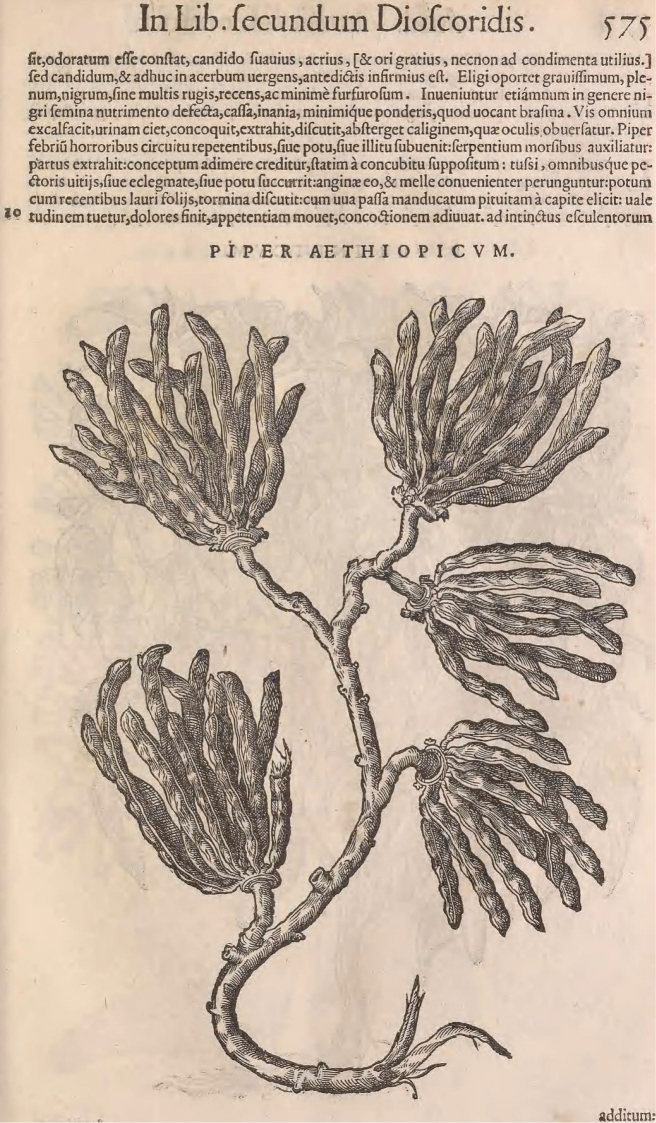
Plate of *Piper AEthiopicum* from Matthioli (1565), the earliest known illustration of *Xylopiaaethiopica*.

The written record of *Xylopia* from Africa, however, far preceded the formalization of binomial nomenclature by Linnaeus. The first record of a species now placed in *Xylopia* likely comes from seeds and fruits described in the work of Avicenna (980–1037). In Europe, Matthioli (1565) provided the earliest known illustration of a *Xylopia*, which he called *Piper AEthiopicum* (p. 575: Fig. 2). [Note: Dunal (1817) cited the 1544 edition of Matthioli’s work as having an illustration on p. 434, but Stephen Greenberg of the National Library of Medicine reports that the only entry on p. 434 of the work is for sect. 99 concerning “Pietra Asia,” and he added that there is no mention of *Piperaethiopicum* in the work; there was neither description nor illustration of the plant in the 1554, 1558, or 1560 editions of the work that we examined in digital copy form.] The woodcut illustration in Matthioli (1565) shows a branch bearing five fruits, and the plant is readily recognizable as the plant now known as *Xylopiaaethiopica* (Fig. 2). Matthioli was also the first to link his *Piperaethiopicum* with the plant referred to as “piper nigrorum” and “hab zelim, id est, grano zelim” by Serapion (Latin translation 1531), which was in turn linked to the plant called “granum azelem” in a Latin translation of Avicenna (1544). Matthioli added that the fruits came through Alexandria in Egypt along with other spices: “Affertur ex Alexandria Aegypticum cum alijs aromatis.” The Matthioli woodcut was copied and modified in various ways over the next 200 years. During this time the name of the plant became standardized as *Piperaethiopicum*.

Oddly, Linnaeus completely ignored the African plant in his works. Gatherings of plants that included *X.aethiopica* first received a post-Linnaean Latin binomial from Lamarck (1785), who proposed the name *Uvariaaromatica* Lam. in recognition of the spicy fruits. Lamarck’s species was founded upon a mixture of materials from varied geographic sources, including not only references to pre-Linnean herbals but also to a specimen from Peru collected by Joseph de Jussieu and others from French Guiana and possibly Mauritius collected by Fusée Aublet.

Two other early generic names proposed by Necker (1790) are connected with *Xylopia*. In his description of *Krockeria* Necker made reference to the plant identified by Aublet (1775) as “*Wariazeylanica*,” a mis-print for *Uvariazeylanica* L. Aublet’s plant had already been incorporated at the species level into *Uvariaaromatica* by Lamarck, but was later separated out by Willdenow (1799), who used Guianan material described by Aublet as the type of his *Unonaconcolor* Willd. The second Necker name, *Bulliarda* Necker, appears to be a re-naming of the genus *Unona* L. f. (Linnaeus 1782), as evidenced by details of the description (umbellate fruits, fruits 2-seeded) that appear in the original description of *Unona*. Jussieu (1810) adopted that interpretation.

In 1817, Dunal’s *Monographie de la famille des Anonacées* provided the first monograph of the family. Dunal adopted *Xylopia*, the shortened form of the generic name used by Linnaeus, recognizing eight tropical American species in the genus, most of them *Xylopia* species in the modern sense but also including two species now placed in *Anaxagorea* A.St.-Hil. Dunal distinguished *Xylopia* from the other Annonaceae genera by the combination of campanulate calyx, outer petals broader than the inner, and short-stipitate compressed monocarps. For unexplained reasons, Dunal transferred Lamarck’s *Uvariaaromatica* to the genus *Unona*, which had been proposed by Linnaeus (1782) for a plant he named *Unonadiscreta* L. f., collected by Dahlberg in Suriname. Dunal also distinguished the African material of Lamarck’s *Uvariaaromatica* as *Unonaaethiopica* Dun. and Jussieu’s plant from Peru as *Unonalucida* Dun. The only elements that then remained with Lamarck’s *Uvariaaromatica* were plants identified in Aublet (1775) as *Wariazeylanica*. Dunal expanded the genus *Unona* to encompass a heterogeneous grouping of 35 species from Africa, Asia, and the Americas. The plates in Dunal’s work clearly illustrate, however, similarities between the species he placed in *Xylopia* and some of those in *Unona*. He himself recognized that the distinctions between *Xylopia* and *Unona* were unclear, noting, at the end of the description of *Xylopianitida* Dun., “Confert *Unonaxylopioides*.” Brown (1818) followed Dunal’s generic description of *Unona*, reporting plants collected by Christian Smith from the Congo region to be “very nearly related to *Piper AEthiopicum* of the shops, the Unonaaethiopica and, perhaps also Unonaaromatica of Dunal.”

The work of Richard, the author for the “Anonaceae” in Guillemin et al. (1831), becomes important at this juncture. Richard argued that the genera *Asimina* Adans., *Porcelia* Ruiz & Pavon, and *Unona* accepted by Dunal should all be reduced to taxonomic synonyms of *Uvaria*. Accordingly, he moved *Unonaaethiopica* to the genus *Uvaria* and described an additional African species of *Uvaria*, *U.parviflora*. Alphonse de Candolle (1832) did not follow Richard’s generic concepts but instead moved in the opposite direction. He distinguished 17 genera in the family and expanded *Xylopia* to ten tropical American species. For Dunal’s *Unonaaethiopica*, Candolle proposed a new genus, *Habzelia* A. DC., and transferred to this new genus *Unonaaethiopica* from Africa, *Unonadiscreta* from tropical America, and *Xylopiaundulata* Palisot de Beauvois, also from Tropical Africa, and described a new species, *Habzeliaobtusifolia* A. DC., from Cuba. Candolle distinguished *Habzelia* from *Unona* by its arillate seeds and striate, glabrous, and irregularly moniliform fruits, and from *Xylopia* by its more elongate and scarcely swollen carpels (i.e. the monocarps), and the convex rather than concave torus [“….toroque verosimiliter non concavo.”]. In the same work, Candolle also proposed the genus *Coelocline* A. DC., noting its similarities to *Habzelia*, *Unona*, and *Xylopia* but distinguishing it on the basis of the concave torus, to which the generic name alludes. Only one species, *Unonaacutiflora* Dunal, was placed in *Coelocline* with certainty, but Candolle proposed new combinations for *C.parviflora* (A. Rich.) A. DC., *C.oxypetala* (Dun.) A. DC., *C.polycarpa* (Dun.) A. DC., and *C.lucida* (Dun.) A. DC. as *Species dubiae*. These were African species except for *C.lucida* from South America. In summary, Candolle adopted a much narrower concept of *Unona* than that of Dunal, restricting it to five species of Asian Annonaceae now classified as species of *Desmos* Lour. and *Dasymaschalon* Dalla-Torre & Harms, and two species from Madagascar now placed in *Uvaria*. This narrower concept circumscribed *Unona* in a way that excluded the type of the genus, *Unonadiscreta*, which Candolle placed under *Habzelia*.

Richard (1841) was the first author to extend the concept of the genus *Xylopia* to plants from Africa, and, in the process, he circumscribed the genus in what would come to be its modern sense. He rejected the genera *Habzelia* and *Coelocline*, transferred species from these genera to *Xylopia*, namely *X.acutiflora* (Dun.) A. Rich., *X.aethiopica* (Dun.) A. Rich., and *X.obtusifolia* (A. DC.) A. Rich., and described the new species *X.cubensis* A. Rich. His concept was then adopted and expanded for Africa by Bentham (1862), Baillon (1864), and Oliver (1868), and, for Asia, by Hooker and Thomson (1855, 1872). *Xylopia*, the Linnaean form of the generic name, was used by these and subsequent authors for most of the 19^th^ century. Kuntze (1891, 1903), however, attempted to re-assert the use of the earlier name *Xylopicrum*, making many new combinations. In the 20^th^ century the name *Xylopia* was conserved over *Xylopicrum* (see Lanjouw et al. 1956). A pantropical generic concept of the genus was now in place, but taxonomic work on the genus generally proceeded separately in the Americas, Asia, and Africa.

In Africa, the dimensions of *Xylopia* diversity were rapidly defined between 1899 and 1936 through multiple works of Engler, Diels, De Wildeman and Durand, Mildbraed, and Exell. The major work of this period was the monograph of African Annonaceae by Engler and Diels (1901), which provided a subgeneric classification of *Xylopia*, dividing it into four sections. Also important was Safford’s (1912) discovery that the type species of the genus *Unona* was in fact a *Xylopia*; the combination *Xylopiadiscreta* was later made by Sprague and Hutchinson (1916). *Unona* was now regarded as a taxonomic synonym of *Xylopia* and no longer a name for *Desmos* and its allies. Local floristic works adding many species to the genus followed, many of them recently incorporated into the sectional classification of Stull et al. (2017).

## Methods

We studied over 2000 collections of African *Xylopia* from the following herbaria (acronyms from Thiers 2017): A, B, BISH, BM, BNRH, BR, COI, DSM, EA, F, FHO, FI-T, FI-W, G, GH, GOET, HBG, K, L, LISC, LISU, LMA, LYD, M, MA, MO, NHT, NY, P, PH, PR, PRC, PRE, RSA, TFD, U, US, W, WAG, WU, and YF. Fieldwork was carried out in eastern Tanzania from January to July 1996. All measurements of plant parts, unless otherwise indicated, are based on dried herbarium specimens. Measurements of fine details of indument, flower parts, and seeds were made using an ocular micrometer mounted on a stereomicroscope and measuring to the nearest 0.1 mm. Publication dates, when unclear, were obtained from the Taxonomic Literature II (TL-II) series (Stafleu and Cowan 1976 and succeeding volumes). Specimen citations are organized geographically from west to east, and then south across the African continent. Within countries, specimens are cited alphabetically by collector or, in the case of some larger countries, alphabetically by political subdivision and then alphabetically by collector.

Maps were generated from herbarium specimen data. In cases where latitude and longitude coordinates were not provided on the specimen label, we determined the coordinates from published maps. Coordinates were assembled in an Excel spreadsheet for each species, and imported into ArcGIS to produce the final maps. Summary maps of species diversity per 1° × 1° grid cell, and of the distribution of individual sections and two informal groups of species, were produced using the individual species maps in ArcGIS. We also summarized species diversity and endemism by biogeographic region and subregion, and superimposed the numbers on a map published by Linder et al. (2012). The individual species maps, generated with ArcGIS, were also used to calculate Extent of Occurrence (EOO) and Area of Occupancy (AOO), following IUCN Red List guidelines (IUCN Species Survival Commission 2012). Cell size for AOO was 2 × 2 km.

### Morphology and anatomy

**Habit.** African xylopias span the size range for the genus and likely for the entire family. Forest species, such as *Xylopiastaudtii* Engl. & Diels, are canopy trees reaching 50 m in height. Many species of tree habit form buttresses at the base of the trunk. In section Neoxylopia, *X.africana* (Benth.) Oliv., *X.rubescens* Oliv., and *X.staudtii*, form conspicuous stilt roots; in *X.staudtii*, the stilt roots bear peglike pneumatophores (Jeník 1970). At the other size extreme in the genus are dryland shrubs such as *X.collina* Diels and *X.tomentosa* Exell, which may be multi-stemmed and flower when only 0.5 m tall. A unique growth form is found in *Xylopiapiratae* D. M. Johnson & N. A. Murray, from West Africa, which is described by most collectors as a liana. Herbarium material of this species exhibits a marked tendency for ultimate branches to be short and divergent from the larger branch axis at nearly right angles. No specialized holdfast structures, such as the hook-shaped inflorescence branches of *Artabotrys* or the twining short-shoots of *Fissistigma* and *Monanthotaxis*, are present, so the shoots may simply sprawl upon surrounding vegetation.

All *Xylopia* species examined have spiral phyllotaxis of the main axis giving rise to a spiral arrangement of the primary branches (Johnson 2003, Hallé 2004). Leaf arrangement on the lateral branches is always distichous. In sect. Verdcourtia D. M. Johnson & N. A. Murray (see pp. 88-89), sect. Stenoxylopia, and some species of sect. Neoxylopia, collateral buds are present, usually two per node but occasionally three. The buds grow out simultaneously, producing two or three branches from the same node (Fries 1919, 1959, Johnson 2003). This pattern produces a “messy” tree architecture that differs from the regularly spaced branches typical of other *Xylopia* species and many other Annonaceae as well (Spruce 1861, Hallé et al. 1978, Johnson 2003, Hallé 2004). Indurated bud scales were seen in *X.arenaria* Engl., *X.collina*, and *X.elliotii* Engl. & Diels, all species of seasonally dry habitats, which in the case of *X.collina* may also be subjected to fire. Twigs in most species are initially pubescent, rarely glabrous, but at length, the hairs are lost and the longitudinal wrinkling characteristic of Annonaceae twigs develops.

**Bark and wood anatomy.** Where reported, the bark is most often described as smooth and light gray to brown. In contrast, in *X.congolensis* De Wild., *X.pynaertii* De Wild., *X.quintasii* Engl. & Diels, *X.staudtii*, and *X.villosa* Chipp, the bark is described as rough and scaly. The upper bark of *X.pynaertii* is red and flakes and peels away from the trunk, a unique feature among African species. Irregular exfoliating patches may develop on otherwise smooth bark in *X.mwasumbii* D. M. Johnson, *X.odoratissima* Welw. ex Oliv., *X.gracilipes* D. M. Johnson & N. A. Murray, and *X.nilotica* D. M. Johnson & N. A. Murray. Fine longitudinal cracks or fissures in the bark have been reported for *X.aethiopica*, *X.gilbertii* Boutique, *X.toussaintii* Boutique, *X.letestui* Pellegr., *X.paniculata* Exell, *X.phloiodora* Mildbr., and *X.tanganyikensis* D. M. Johnson. Collectors frequently remark on the aromatic character of the cut bark, and the species *X.phloiodora* is named for that property.

Koek-Noorman and Westra (2012) surveyed Annonaceae wood anatomy, including 24 species of *Xylopia*, five of them African. The wood of *Xylopia* species surveyed had the anatomical structure characteristic of the Annonaceae, namely wide and high parenchyma rays and closely spaced tangential parenchyma bands. *Xylopia* and *Artabotrys* R. Br. have largely similar wood, showing relatively large solitary vessels, often with a single vessel in contact with the parenchyma ray on either side of it, or, alternatively, vessels in small radial multiples with widely spaced tangential parenchyma bands. A major departure was found in two tropical American species of *Xylopia*, *X.peruviana* R. E. Fr. and *X.cuspidata* Diels: their vessels have relatively small diameters and the tangential parenchyma bands are more numerous and closer together. The African species surveyed represented three of the four major *Xylopia* clades, suggesting that wood structure has largely been conserved evolutionarily across the genus.

**Leaves.** Leaves of *Xylopia* are always distichous, exstipulate, simple, and entire, as is characteristic of the family. The leaf blades are commonly lanceolate, elliptic, or oblong, and chartaceous to subcoriaceous. In African species, the blades most often bear inconspicuous hairs, but in *X.hypolampra* Mildbr., *X.letestui*, *X.talbotii* Exell, and *X.villosa*, the blades are densely pubescent on the abaxial surface. The indument of the leaf is most pronounced on the abaxial surface in *X.hypolampra*, so named for the shining sericeous covering formed by the hairs. The secondary vein pattern is brochidodromous; in some species, such as *X.quintasii*, the loops are distinctly closed and conspicuous, while in others, such as *X.odoratissima*, the secondary veins may branch near the leaf margin and form only weak connections with the succeeding secondary vein. The secondary veins can be strongly arcuate in some species, such as *X.paniculata* Exell. The petiole is usually short, less than 1/5 of the length of the blade, and flattened to canaliculate on the adaxial surface.

Leaf anatomy was studied by Kramer (1969), who summarized the anatomical features as the following: small to medium-sized druses present in nearly every cell of the upper and lower epidermis, venation reticulate, midvein simple with a single arc of vascular tissue usually divided into more or less distinct bundles, palisade mesophyll comprised of 2–3 cell layers, and bicellular trichomes that are more numerous on the abaxial surface. He noted several anatomical features that were variable in the genus: presence of a multiple epidermis or hypodermis, sclereids in the mesophyll, papillate lower epidermis, and sculpturing of the internal walls of the basal cells of the trichomes, the latter only occurring in some Neotropical species. Both a uniseriate epidermis and a multiseriate epidermis were found in African and in non-African species. Mesophyll sclereids, in contrast, were found exclusively in Paleotropical species, including *X.acutiflora*, *X.letestui*, *X.quintasii*, *X.piratae*, and *X.thomsonii* Oliv. from Africa. *Xylopialetestui* was unique among the *Xylopia* species sampled in having multicellular papillae on the lower leaf surfaces and septate apical cells of the bicellular trichomes.

**Inflorescences.** Inflorescences in *Xylopia* are always axillary, sometimes arising from the axils of fallen leaves. Cauliflory was not observed in any African species. The rhipidiate inflorescence described for the Neotropical species *X.brasiliensis* by Fries (1919) is only evident in *X.aethiopica*; both are members of section Xylopia. In many species the inflorescences consist of pedicels that arise independently side-by-side from the leaf axil; in others, there may be one common peduncle from which some or all pedicels of the axil arise. In species such as *X.calva* and *X.phloiodora* the peduncle may be broader than long and somewhat embedded in the stem. The peduncle always lacks bracts; the pedicels bear 2–6 small (0.5–7.4 mm long) bracts that may be caducous or persistent, somewhat clasping, and usually pubescent on the abaxial surface. The largest bracts occur in the closely related species *X.villosa* (up to 7.4 mm) and *X.letestui* (up to 6 mm). Bracts in species such as *X.acutiflora* and *X.thomsonii* number 3–6 per pedicel and are often imbricate. In these species, the pedicel superficially resembles a grass spikelet, and the bracts often persist into the fruiting stage. The number of flowers per inflorescence is typically 1–2, occasionally up to five. That number is exceeded in the following species: *Xylopiaholtzii* Engl. and *X.shirensis* (Engl. & Diels) D. M. Johnson & N. A. Murray, with up to 6 flowers per inflorescence, *X.aethiopica* and *X.quintasii* with up to 7, *X.tomentosa* and *X.villosa* with up to 8, *X.calva* D. M. Johnson & N. A. Murray and *X.phloiodora* with up to 10, and *X.katangensis* De Wild. with up to 12. Exceptionally, in *X.paniculata* there are up to 32 flowers per inflorescence.

Flower buds in *Xylopia* are linear to ovoid. Because the petals lengthen early in development, the buds provide a reliable indicator of the ultimate shape of the petals. The petals of both whorls remain connivent until anthesis, when they spread apart in various ways. The apex of the bud may be acute to obtuse, and, in narrower buds, is often falciform at the apex.

**Flowers.***Xylopia* flowers have the perianth format typical of Annonaceae, a single whorl of three sepals, a whorl of three outer petals, and a whorl of three inner petals borne on a flat or slightly concave receptacle. Perianth parts are valvate in aestivation within each whorl; the sepal bases are imbricate in a few species. The sepals are much shorter than the petals, often connate at the base to form a cuplike or bowl-shaped calyx, and coriaceous in texture. In most species, they remain erect or may spread slightly, but in *X.longipetala* De. Wild. & T. Durand they are completely reflexed. The outer petals are membranous to fleshy, and vary in shape from broadly ovate to linear, and from acute to rounded at the apex. The adaxial pubescence consists of finer and more erect hairs than are present on the abaxial surface, where the hairs tend to be longer, more appressed, and often yellow-brown to orange-brown in the dried condition. The base of the petal is concave and usually glabrous on both surfaces. The adaxial surface of the outer petal is shallowly concave but becomes ridged toward the apex where the petals are pressed together in bud above the inner petals; the abaxial surface often has a weak longitudinal ridge for the length of the petal.

In most species, the inner petals are slightly shorter and narrower than the outer petals. In *X.thomsonii*, it is common for the inner petals to be only ca. 2/3 of the length of the outer petals. The most pronounced difference in the lengths of outer and inner petals occurs in *Xylopiarubescens*, where the linear outer petals are ca. eight times the length of the ovate inner petals. Inner petal texture varies from coriaceous to fleshy for most species, but is subcoriaceous in *X.piratae*, chartaceous in *X.staudtii*, *X.africana*, *X.globosa* D. M. Johnson & N. A. Murray, *X.katangensis*, and *X.wilwerthii* De Wild. & T. Durand, chartaceous to membranous in *X.longipetala*, and membranous in *X.tenuipetala* D. M. Johnson & Goyder. The pubescence of the abaxial surface in inner petals resembles that of the adaxial surface, and both surfaces bear a longitudinal ridge. The base of the inner petal is concave and glabrous, and is more differentiated than the base of the outer petal, often with a transverse “heel” where the petal may be bent outward above a thinner basal claw of the petal, for example in *X.tomentosa*. In the case of *X.aurantiiodora* De Wild. & T. Durand, there is a distinct tooth that forms on the adaxial surface at this widest point. In several African species, the margins of the inner petals are differentiated toward the base. In sect. Ancistropetala, the petal base has thin inrolled glabrous margins, and, in the species *X.gilbertii*, *X.flamignii* Boutique, and *X.toussaintii* of sect. Stenoxylopia, the margins are thickened and knobby. These differentiated petal margins may represent food tissue for flower visitors as in, for example, *Cymbopetalum* Benth. (Murray 1993), or perhaps they are secretory, but no supporting field observations have been published.

**Figure 3.**
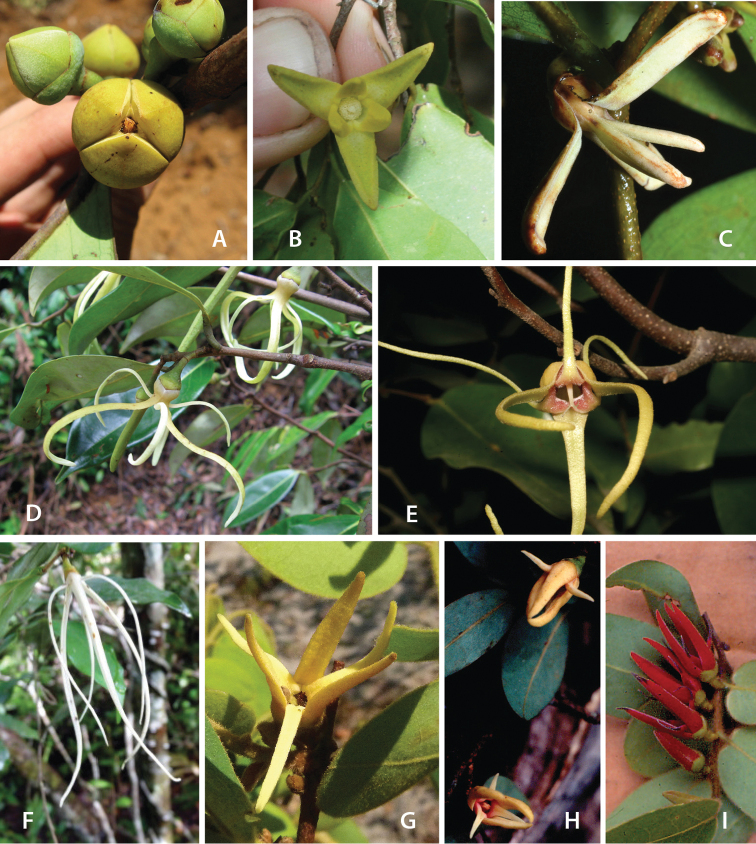
Flowers of representative *Xylopia* species. **A** Flower from type collection of *Xylopiaglobosa* from Gabon **B***Xylopiatenuipetala* from Mozambique **C***Xylopiaquintasii* from Gabon **D***Xylopiaaethiopica* from Gabon **E***Xylopialongipetala* from Mali, representing a record for the country not otherwise documented **F***Xylopiapiratae* from Ivory Coast **G***Xylopiaodoratissima* from Zambia **H***Xylopiaarenaria* from Tanzania **I***Xylopiacollina* from Tanzania. **A, D** by Thomas L. P. Couvreur **B** by Frances Chase **C** by Ehoarn Bidault **E** by Philip Birnbaum **F** by Céline Pirat **G** by Warren McClelland **H** and **I** by D. M. Johnson.

Petal color typically ranges from pure white to pale yellow or cream-colored to pale orange, often with purple or red blotches around the pollination chamber aperture; the apex of the anther connective is often red as well. A limited number of variations occur. The flowers of *X.mwasumbii* and *X.tenuipetala* are tinted green at anthesis, and the flowers of *X.collina* and *X.flamignii* becoming dull red at maturity (Fig. 3). In *X.rubescens*, the long outer petals are yellow to orange, but the short inner petals are dark red. Flower color has not been reported for many species.

Petal orientation at anthesis is variable in African xylopias and determines the shape of the pollination chamber formed by the petals. In most species, the outer and inner petals separate at the beginning of anthesis, curving gradually outward at the base and then curving inward toward the apices, the petal bases alone forming the chamber (Fig. 3D, E, G). In species of sect. Neoxylopia, however, the petals hardly separate from one another and the entire corolla forms the chamber. In sect. Stenoxylopia, species including *X.arenaria*, *X.collina*, and *X.tomentosa*, have outer petals that remain erect, while the apices of the inner petals bend outward and extend horizontally through the gaps between the outer petals; the chamber is formed by the outer petals and the bases of the inner petals (Fig. 3H, I). In sect. Verdcourtia, it is the outer petals that bend outward perpendicular to the erect inner petals, and the inner petals in their entirety form the chamber (Fig. 3B).

The androecium of *Xylopia* is one of the most specialized in the Annonaceae, but also has a number of typical family characteristics. Most species have an androecium with a large number (60–200) of fertile stamens, the apex of the anther connective is short (0.1–0.4 mm), truncate or shieldlike in shape and overhanging the thecae, the filaments are short (0.2–0.6 mm), and the anthers have extrorse dehiscence. A trend toward fewer stamens is evident in section Verdcourtia, with stamen numbers ranging from ca. 65 in *X.lukei* D. M. Johnson & Goyder to as few as 40 in *X.aurantiiodora* and *X.mwasumbii*. The apex of the anther connective in species of sect. Ancistropetala is globose or conical and does not overhang the thecae, lending a pebbly appearance to the surface of the androecium, in contrast to the typical flat surface; in species of sect. Verdcourtia, the apices are rudimentary (≤0.1 mm long). Filaments exceeding 0.6 mm in length occur in several species: *X.rubescens*, *X.africana*, *X.lukei*, *X.mwasumbii*, *X.tenuipetala*, *X.unguiculata* D. M. Johnson & N. A. Murray, and *X.calva*. The anthers of *Xylopia* are transversely septate, the septa forming 4–14 locelli, each of which contains one pollen tetrad or a polyad of 5–6 coherent tetrads (Walker 1971); *X.arenaria* and *X.collina* were found to occasionally have up to 17–18 locelli and *X.phloiodora* characteristically has the anthers divided into 20–24 locelli. The walls of the septa are parenchymatous (Tsou and Johnson 2003) and are typically sturdy enough to withstand the tearing of the outer wall during anther dehiscence and thus are still visible in dehisced anthers.

The androecium of all species of *Xylopia* includes staminodes, which rarely contain rudimentary anther sacs. Both outer and inner staminodes are typically present in the same flower, a unique condition in the Annonaceae (van Heusden 1992). The outer staminodes are about the same length as fertile stamens but are usually wider and flattened all the way to the apex. The inner staminodes are more similar to fertile stamens in shape, sometimes with a flattened anther connective apex retained; they often adhere to the bases of the stigmas in dried specimens. In Africa, outer staminodes are absent in only two species, *X.mwasumbii* and *X.tenuipetala*, both members of section Verdcourtia, in which fertile stamen numbers are also reduced. Inner staminodes are absent in *X.aurantiiodora* and *X.quintasii*, and, probably, in *X.congolensis* and *X.mildbraedii* Diels as well. Occasional flowers of *X.mwasumbii* may have reduced numbers of locelli on the innermost stamens or lack inner staminodes. Staminodes are rare in Annonaceae, occurring in only seven genera (van Heusden 1992, Saunders 2010). The unique presence of both outer and inner staminodes in *Xylopia* probably represents an independent evolution of sterile stamens within the Annonaceae, as *Xylopia* is not closely allied to any of the other staminodial genera.

The most distinctive aspect of the androecium of *Xylopia*, however, is the cone on which the stamens are borne. In some species, the cone forms a chamber completely enclosing the ovaries, with a narrow aperture from which only the stigmas protrude; this is the “hollow torus” on which Candolle (1832) based his concept of the genus *Coelocline*. Kramer (1969) indicated that the cone was probably receptacular rather than staminal in origin, and Deroin (1989), in a study of floral cortical vascular systems in Annonaceae, followed this interpretation. Verdcourt (1971b) described the cone as “filaments articulated, sometimes partly united at the base and enclosing the gynoecium.” In an anatomical study of mature *X.aromatica* flowers, Dias et al. (1998) found that the cone surrounding the carpels was of mixed origin, consisting of fused sepals, petals, and stamen filaments but with the apical portion “exclusively formed by the fusion of the filaments, thus constituting a staminal tube.” In all species examined in this study, individual filaments are distinguishable on the cone, below the stamen abscission scars. In sect. Xylopia and in some species of sect. Stenoxylopia, the staminal cone is well developed and completely encloses the ovaries. In species of sect. Neoxylopia, sect. Verdcourtia, and most species of sect. Stenoxylopia, the cone is rudimentary and consists only of a low laciniate rim around the base of the ovaries, and, in the species of sect. Ancistropetala, the cone is completely absent. Remnants of the cone often persist into the fruiting stage, but are torn apart by the expansion of the torus as the fruit matures.

*Xylopia* pollen is shed in tetragonal tetrads or, in *X.africana*, the Asian species *X.ferruginea* (Hook. f. & Thoms.) Baill., and the Neotropical species *X.brasiliensis* and *X.micans* in polyads of about 5–6 individually discernible tetrads, with each tetrad or polyad in a separate compartment of the septate anther (Walker 1971). Walker (1971) described the individual grains as heteropolar, bilateral, catasulcate to cataulcerate, boat-shaped to triangular to disklike, with the long axis of 52–170 µm. The exine is smooth and microperforate in many species (Walker 1971, Le Thomas 1980, 1981), but verrucose in *X.quintasii* and so thin as to be nearly absent in species such as *X.arenaria* and *X.holtzii* (Tsou and Johnson 2003). Le Thomas (1980, 1981) described the structure of the pollen wall in *X.aethiopica* as consisting of a thick smooth continuous tectum over a granular infratectal layer (*Bos 2198*) and that the exine of *X.staudtii* (*Letouzey 11854*) was similar except for the greater fusion of the granules in the infratectal layer to form a “massive discontinuous basal layer in which their contours are no longer recognizable.” Doyle and Le Thomas (2012) later concluded that their previous interpretation of the infratectal granules as becoming fused needed to be re-examined as a possible case of reduced columellae.

The carpels in *Xylopia* are always separate and, in African species, vary in number from two in some flowers of *X.tenuipetala* to ca. 40 in *X.aethiopica*; no unisexual flowers were found, such as those described for the Asian species *X.championii* Hook. f. & Thoms. by Ratnayake et al. (2007). The carpels consist of a short, oblong, pubescent ovary surmounted by a relatively long filiform structure bearing the stigmatic surface but not differentiated into style and stigma. We refer to the entire structure as the stigma. In *Xylopia*, the stigmas are usually glabrous except for a tuft of hairs at the apex. They are connivent or free; when connivent, the stigmas drop as a group as they abscise from the ovaries at the end of anthesis. There is a large amount of exudate produced on the stigmas in species observed in the field and connivent stigmas are difficult to separate from each other in the dried condition. It is not clear whether there is also a cellular connection between the connivent stigmas. The exudate may be a pollinator reward, an aid to pollen germination or form an extragynoecial compitum, which has been shown to guide intercarpellary growth of pollen tubes in other Annonaceae (Lau et al. 2017). In the species of sect. Neoxylopia, the stigmas bear papillae or tubercles along their sides; in the case of *X.rubescens*, the tubercles are stalked (Le Thomas 1969, Plate 28, fig. 8). In sect. Ancistropetala, the stigmas are clavate or narrowly oblong, and stand apart from one another without touching. In the *X.mwasumbii* group, they are short and thick, and, again, not at all connivent (Johnson et al. 1999). The most conspicuous stigmas of the genus occur in *X.longipetala*, where the connivent stigmas reach a length of 7 mm and extend well beyond the aperture formed by the overlapping petal bases (Fig. 3E).

The ovaries contain 2–12 ovules, attached laterally in a single or an irregular double row. Kramer (1969) showed that the ovules of the single row alternate in arising from placentas on either side of the suture of the conduplicate carpel, and that they are anatropous in orientation; he further observed that the vascular supply to each carpel consists of an unbranched dorsal bundle that extends to the stigma and two ventral bundles that produce short branches approaching the ovules with the main bundles then extending to the stigma as well. Sillans (1953) described and illustrated the carpels of *X.ardua* Sillans [= *X.gilbertii* in this work] as borne on a gynophore; we found the structure described as a “gynophore” in this species to be, in fact, the staminal cone outside of and surrounding the bases of the ovaries. The torus itself is flat or slightly concave underneath the gynoecium, but is neither conspicuously elevated nor hollow.

**Figure 4.**
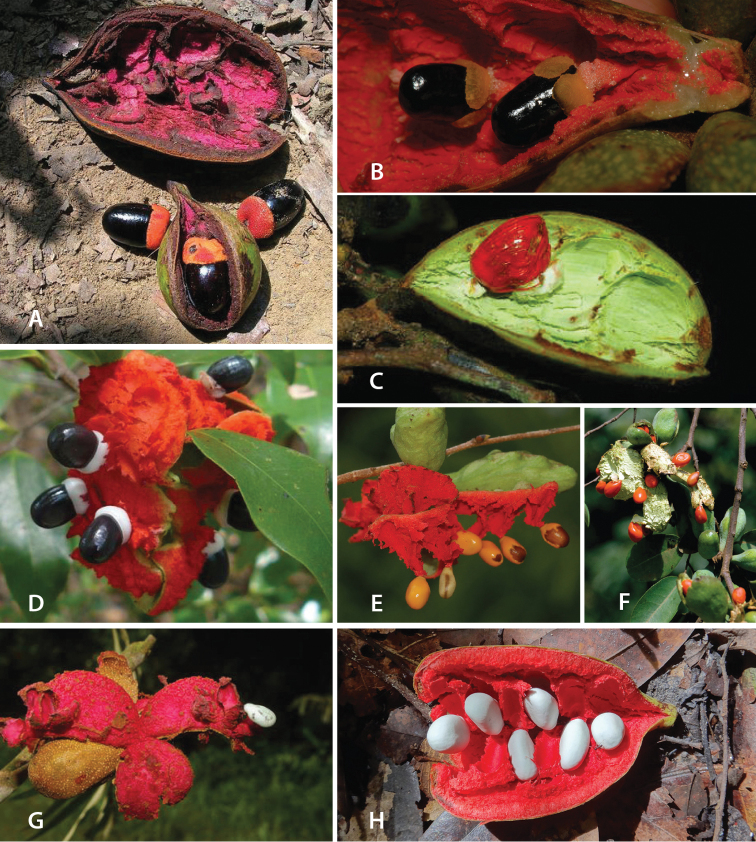
Fruits and seeds of representative *Xylopia* species. **A***Xylopiastaudtii* from Democratic Republic of the Congo **B***Xylopiaaethiopica* from Republic of the Congo **C***Xylopiaquintasii* from Cameroon **D***Xylopiatenuipetala* from Mozambique **E***Xylopiacollina* from Mozambique **F***Xylopiagracilipes* from Mozambique **G***Xylopiahypolampra* from Gabon **H***Xylopiatanganyikensis* from Tanzania. **A** by Quentin Luke **B** by David Harris **C, G** by Thomas L. P. Couvreur **D** by Jonathan Timberlake **E, F** by Mervyn Lötter **H** by Noriko Itoh. **C** reproduced with permission of Thomas L. P. Couvreur and of the American Society of Plant Taxonomists **D** reproduced with the permission of Jonathan Timberlake and of the Board of Trustees, Royal Botanic Gardens Kew.

**Fruit.** Fruits follow the typical Annonaceae aggregate pattern, with each carpel developing into a discrete fruitlet called a monocarp. Monocarps vary in length from 1.9 cm in *X.katangensis* to 16.3 cm in *X.rubescens*, in width, i.e. adaxial to abaxial distance, from 0.3 cm in *X.aethiopica* to 4.0 cm in *X.paniculata*, and in thickness, i.e. perpendicular to width, from 0.3 cm in *X.aethiopica* to 3.5 cm in *X.letestui*. The ripe monocarps of all species, despite being somewhat fleshy and berrylike, dehisce longitudinally along the abaxial surface to expose the seeds. *Xylopia* shares this morphology with members of tribe Bocageeae (Johnson and Murray 1995). Monocarp number depends on effectiveness of pollination as well as carpel number; the highest numbers recorded for African species are up to 18 in *X.cupularis* Mildbr., up to 22 in *X.flamignii*, and up to 36 in *X.aethiopica*. The monocarps are broadly oblong or ovoid to linear and often have slight constrictions between the seeds; in *X.mwasumbii* and *X.tenuipetala* they are laterally compressed. The outside of the fruit is usually green, red, or purple, may be glabrous or pubescent, and may have oblique wrinkles and small warts when dry; in a few species, such as *X.hypolampra*, *X.letestui*, and *X.phloiodora*, the exocarp is strongly marked with lenticels and is brown (Fig. 4G), and in *X.hypolampra* and *X.longipetala* the fruit frequently has longitudinal ridges or ribs. The mesocarp is fibrous and green to brown, and the endocarp is usually pink to scarlet (Fig. 4A, B, D, E, G, H). In *Xylopiaquintasii*, and in *X.gracilipes*, *X.shirensis*, *X.torrei* Exell, and, perhaps, other species of the *Xylopiaodoratissima* group, the endocarp is green (Fig. 4C, F). The apex of each monocarp is obtuse to rounded or sometimes is formed into a short (1–6 mm) beak, and the monocarp base is contracted into a stipe of varying length or rarely (e.g. *X.keniensis*, *X.paniculata*, *X.villosa*) the monocarps are sessile on the torus.

**Seeds.** Monocarps contain seeds in one or two rows. The seeds are attached laterally and are parallel, at an oblique angle, or perpendicular to the long axis of the monocarp. Seed number per monocarp in African species varies from one to 20. The seeds are ellipsoid and vary in length from 5 mm in *X.aethiopica* to nearly 22 mm in *X.paniculata*. Seeds are rounded or pointed at the chalazal end and usually truncate at the micropylar end with a distinct micropylar scar. In a few species, for example, *X.arenaria*, *X.collina*, *X.longipetala*, and *X.tomentosa*, the seed suddenly narrows toward the micropylar end to form a “neck” so that the seed is pyriform. The seed coat is smooth or rarely slightly wrinkled or pitted. In all Annonaceae, the raphe/antiraphe forms a hoop that encircles the seed (Corner 1949, the “perichalazal ring” of Pirie and Doyle 2012). In *Xylopia* ,it may be raised into a keel, as in *X.staudtii*, or be flush with the surface of the seed and visible only as a faint line. The seed coat, which has only been studied in three Neotropical species, consists primarily of the outer integument, a morphology found in a number of other genera of Annonaceae (Christmann 1986). The endosperm has the ruminate pattern that is a diagnostic feature of the family, the ruminations formed by ingrowths of the inner integument perpendicular to the long axis of the seed. Lamelliform ruminations in quadrants, typical of many Annonaceae (van Setten and Koek-Noorman 1992), were present in dissected seeds of *X.staudtii* (sect. Neoxylopia), and *X.holtzii* and *X.phloiodora* (sect. Stenoxylopia), but in *Xylopiaquintasii* (sect. Ancistropetala), the ruminations were peglike, irregular, and somewhat branched. Rumination pattern has not been examined for most African species, but might, if better known, prove useful for interpreting the family’s fossil record (see Pirie and Doyle 2012).

Arils of four types, arising near the micropyle, are found in African *Xylopia* species (Fig. 4A–D, reviewed in Stull et al. 2017). In sect. Neoxylopia, arils are yellow to red, their brushlike appearance being due to the many tightly packed fleshy rod-shaped appendages attached to a disklike base (Fig. 4A). In sect. Ancistropetala, arils consist of a ring of orange to red strips forming a reflexed fringe that completely encloses the seed (Fig. 4C). In sect. Xylopia, the aril is orange or white, fleshy, and bilobed (Fig. 4B). Finally, in *X.mwasumbii* and *X.tenuipetala* of sect. Verdcourtia, the aril forms a fleshy white cup around the end of the seed (Fig. 4D). In the African species of sect. Stenoxylopia, an aril is absent.

In species lacking an aril, a fleshy pigmented outer seed coat layer, or sarcotesta, is present. The sarcotesta is only a few cell layers thick and is not conspicuous in dried specimens. However, seeds with a sarcotesta usually dry dull brown, while those lacking a sarcotesta are shiny dark brown to black. *In vivo*, the sarcotesta in the *Xylopiaodoratissima* group is orange or red (Fig. 4E, F). In the *X.acutiflora* group, however, the colors range from white to pale gray, light green, or verdigris (Fig. 4G, H), with the exception of reports of an orange sarcotesta in *X.phloiodora*.

### Floral biology and seed dispersal

**Floral biology.** At anthesis, *Xylopia* flowers produce a pollination chamber surrounding the androecium and gynoecium. It is formed by the petals and reinforced by the calyx. Flowers are protogynous, with varying degrees of temporal separation between stigmatic receptivity and anther dehiscence. What we know of floral biology in the genus is in accordance with a small beetle pollination system, considered ancestral for the Annonaceae (Saunders 2012).

Petal orientation at anthesis varies among species within the genus, with corresponding differences in the size and shape of the floral chamber. Flower colors, which range from white to red, and fragrances are the likely attractants for pollinators. The fragrance of *Xylopia* flowers is often noted by collectors, but seldom with qualitative descriptors. Of descriptors provided, some are positive, for example the fragrance of *X.odoratissima* was compared by one collector to that of a sweet pea (*Jenkins 1*) and *X.aurantiiodora* was named for the resemblance of its floral fragrance to that of *Citrus* flowers. On the other hand, the scent of *Xylopiaarenaria* flowers is described variously as the smell of over-ripe bananas to the “smell of Annonaceae with overlying smell of cats” (*Mabberley and Harris 1516*). There is, thus, enough variation in attractants to expect variation in flower visitors.

Little is known of pollinator rewards provided by *Xylopia* flowers. Even small flowers probably provide enough pollen to serve as a food reward. The differentiated margins of the inner petal base, seen in the species of sect. Ancistropetala and in *X.flamignii*, *X.gilbertii*, and *X.toussaintii* of sect. Stenoxylopia, may serve as food tissue, or are perhaps secretory in nature, but no evidence of feeding damage to these tissues was observed. Likewise, most species have both inner and outer staminodes that could have nutritional value. While the inner staminodes usually adhere tightly to the bases of the stigmas, and may have a protective function, the outer staminodes are readily accessible to visitors inside the pollination chamber. The floral chamber may provide a mating place for visitors, and, in some species, the chamber may also provide thermogenesis (Saunders 2012.)

We briefly observed the floral biology of *Xylopiaarenaria*, sect. Stenoxylopia, in eastern Tanzania in April, 1996. A total of 27 flowers in or approaching anthesis were marked on two individual trees and followed over 3–4 visits during a 21-hour period. Flowers were inspected in the late afternoon of 26 April and then again in early morning and early and late afternoon on 27 April. We recorded the petal color, petal position, stigmatic receptivity via exudate production (Ratnayake et al. 2007), anther dehiscence, and the presence of visitors for each flower.

By comparing the stages of anthesis present at each observation time, we reconstructed the sequence of floral events. In *X.arenaria*, stigmatic receptivity was signaled by outward movement of the narrow apices of the inner petals, beginning in the late afternoon or evening of Day 1 (Fig. 3H).

By the morning of Day 2, the inner petal apices were bent nearly perpendicular to the outer petals, which remained erect. Color darkened from cream to yellow or from yellow to pale orange. The stigmas were white, pressed tightly together, and shining with stigmatic exudate. An unpleasant rotting fruit scent was very strong. This stage continued until the following morning.

On the morning of Day 3, the flowers were still in the pistillate stage, but the scent was weaker. By early afternoon, the stigmas had begun to separate and turn dark, which we interpreted as signaling the end of the pistillate stage. By early afternoon, the stigmas were beginning to abscise and the stamens to loosen. Scent was less strong during the staminate stage, and petal position did not change. During the night of Day 3, the petals and stamens dropped so that by morning only pedicel, sepals, and carpels remained. Most pedicels dropped soon after.

Four floral visitors were observed. A weevil was present in one flower from early morning until early afternoon during the pistillate stage. Staphylinid beetles were observed in three separate flowers, two in pistillate-stage flowers in early morning and one in a staminate-stage flower in late afternoon.

In a detailed study of a second species of section Stenoxylopia, *Xylopiachampionii* from Sri Lanka, Ratnayake et al. (2007) marked ten flowers on five individual trees to follow the stages of anthesis, and found that the flower buds grew over a period of one month, with the petals of both whorls gradually bending apart at the end of that time. Stigmatic receptivity lasted for 15–17 hours, and was signaled externally by the partial closing of the outer petals and the tight closing of the inner petals to form a pollination chamber. The petals forming the chamber then reopened and a 6-hour non-receptive interim stage commenced. This was followed by anther dehiscence lasting for ca. 17 hours. Overall petal color changed from greenish yellow to light yellow before the onset of stigmatic receptivity, and the stamens and adaxial base of the inner petals gradually darkened from pink to dark red or purple by the onset of anther dehiscence. Small weevils carried pollen and visited the flowers during the pistillate and staminate stages, but were not present before anthesis or during the interim stage. While limited self-compatibility was demonstrated, the breeding system is clearly directed toward xenogamy (see also Pang and Saunders 2014).

In contrast with *X.championii*, the petals of *X.arenaria* did not open and close during anthesis but rather maintained the same orientation throughout all stages. The pollination chamber was composed of the outer petals and only the bases of the inner petals, rather than of the entire inner petals. The period of stigmatic receptivity was longer, about 36 hours, but the period of anther dehiscence was much shorter (6–8 hours). There was no distinct interim stage between stigmatic receptivity and anther dehiscence in *X.arenaria*. *Xylopiaarenaria* observations thus point to a system in which visitors are likely to remain in the flowers throughout anthesis. As with *X.championii*, floral synchrony within individuals (Murray and Johnson 1987) was not observed. *Xylopiaarenaria* produces hundreds of flowers that form few fruits, a possible indicator of a breeding system that promotes outcrossing, as Ratnayake et al. (2007) found in *X.championii*. In contrast, collections of *X.aethiopica*, *X.longipetala*, and *X.thomsonii* frequently have many fruits with many monocarps per fruit, suggesting inbreeding mechanisms may be present in these species (see Pang and Saunders 2014 for an overview of floral biology in the family).

These comparisons suggest the limitations of generalizing from a few examples of floral biology. Five of the six sections of *Xylopia* are represented in Africa and these include the basal clades of the genus. Other studies of *Xylopia* species, reviewed by Saunders (2012), all belong to section Xylopia, represented by only a single species in Africa. Species in this section all have a well-developed staminal cone and flat-topped anther connective apices typical of Annonaceae, characteristics that have been suggested to protect against feeding by floral visitors (Ratnayake et al. 2007). However, neither of these characteristics is present, for example, in the Ancistropetala section of the genus endemic to Africa. Given the range of variation in floral morphology and the events of anthesis, selection on floral biology traits appears to have been important in evolution within the genus but is still poorly known.

**Seed dispersal.** Ripe *Xylopia* fruits hang downward on a short woody pedicel with the abaxial surface of the separate monocarps facing outward. Depending on the species, there may be up to 36 monocarps per fruit. The monocarps dehisce asynchronously, opening longitudinally to display the seeds (Fig. 4). A single monocarp may hold up to 20 seeds varying in length from 5 to 22 mm. An aril or sarcotesta provides a food reward for dispersers. The striking color contrasts between the fruit and seeds are achieved by a variety of morphologies, but all combinations are in accord with a recognized syndrome of dispersal by diurnal vertebrates with trichromatic color vision. This syndrome encompasses the two main groups of *Xylopia* seed dispersers documented for Africa, birds and primates (Gautier-Hion et al. 1985, Lahm 1986, Whitney et al. 1998, Holbrook and Smith 2000, Clark and Poulsen 2001, Poulsen et al. 2001, Eckardt and Zuberbühler 2004, Lamperti 2004, Koné et al. 2008, Lamperti et al. 2014).

A study of hornbills in the Dja Reserve in southeastern Cameroon documented bird dispersal for 56 plant species, including four species of *Xylopia* (Whitney et al. 1998). The canopy species *X.hypolampra*, *X.letestui*, *X.rubescens*, and *X.staudtii* were fed upon by up to three species of large hornbills, *Ceratogymnaatrata*, *C.cylindricus*, and *C.fistulator*. The birds effectively dispersed seeds away from the parent trees. Defecated seeds of three of the four species germinated, and, in the case of *X.rubescens*, seed germination was greater after gut passage compared to controls. Holbrook and Smith (2000) calculated the seed shadow produced by two hornbill species for several canopy trees, including *X.hypolampra*, and found that in *X.hypolampra* the seed shadow extended to more than 6500 m from the parent tree, with a peak at 1000 m.

Poulsen et al. (2001), in a study of seed dispersal by a primate community, also in the Dja Reserve, reported that seeds of five *Xylopia* species, *X.aethiopica*, *X.quintasii*, *X.parviflora*, *X.rubescens*, and *X.staudtii*, were consumed by monkeys including gray-cheeked mangabeys (*Lophocebusalbigena*), white-nosed guenons (*Cercopithecusnictitansnictitans*), and crowned guenons (*C.monapogonias*). Seeds of *X.rubescens* and *X.staudtii* were recovered from fecal samples of gray-cheeked mangabeys, and showed about 40% and 10% germination, respectively, demonstrating that seeds can pass intact and viable through the monkey’s gut. The average passage time of 22 hours reported in this study allows for dispersal of the seeds to significant distances from the parent trees. Koné et al. (2008) reported a similar system of primate dispersers and *Xylopia* species in Taï National Park, Ivory Coast. Six different primates took the seeds from one or more of five *Xylopia* species, *X.acutiflora*, *X.aethiopica*, *X.parviflora*, *X.quintasii*, and *X.villosa*. The monkeys either swallowed the seeds or spat them out undamaged.

The species of *Xylopia* seeds taken by hornbills and by monkeys thus overlap but the overlap is not complete. For example, *Xylopiaquintasii* is reported as a monkey food item in five studies (Mitani 1999, Poulsen et al. 2001, Eckardt and Zuberbühler 2004, Koné et al. 2008, Wang 2008) but not as a hornbill food item. Conversely, *X.hypolampra* was taken only by hornbills. The observed preferences may relate to tree architecture, phenology, or the nature of the food reward.

Clark and Poulsen (2001) analyzed the contributions of hornbills and monkeys to seed dispersal of canopy tree species and concluded that monkeys feed lower in the trees and feed on more species, while hornbills feed higher in the canopy and disperse greater numbers of seeds. This may explain some feeding preferences: *X.hypolampra* ,for example, is a slender high canopy species with a small exposed crown and therefore perhaps undesirable to monkeys. Furthermore, no smaller-stature *Xylopia* species were reported in the hornbill studies, while monkeys have been reported to feed on two understory species, *X.acutiflora* and *X.parviflora* (=*X.longipetala*).

Phenology determines the seasonal availability of fruits and seeds to dispersers as well as competition for disperser services. In *Xylopia*, fruiting seasons are long, and asynchronous dehiscence of the individual monocarps promotes multiple visits by dispersers to each fruit. Fourrier (2013) described fruiting patterns for three *Xylopia* species in the Lopé Reserve of Gabon over a ten-year period. The mean fruiting dates for the *Xylopia* species all fell sometime during August, but each species exhibited a different pattern. *X.quintasii* (probably actually *X.congolensis*, K. Abernethy, personal communication) had an availability duration of 4.1 months, *X.aethiopica* 5.3 months, and *X.hypolampra* 11.5 months. Thus *Xylopia* alone may provide a nearly year-round food source for local forest dispersers and appears to be solely dependent on these dispersers, rather than on a wider mix that includes migratory species.

The nature of the food reward presents additional variables that influence dispersers. As noted above, attractants provided by *Xylopia* fruits include the color contrasts of endocarp and seeds that provide a search image for dispersers. The diverse morphologies by which *Xylopia* species achieve these contrasts suggest intense selection. For example, arils are present in the two earlier-diverging clades of the genus (Stull et al. 2017) and could be energetically more costly for the plant than the sarcotestas that predominate in more recently diverging clades.

Alternatively, there may be differences in effectiveness of dispersal. Lambert (1999) showed that method of seed handling by primates influences dispersal. Primates may either spit or swallow *Xylopia* seeds. Spitting led to more limited dispersal than swallowing. Koné et al. (2008) found that arillate *Xylopia* seeds were both swallowed and probably spat by multiple monkey species, while seeds with sarcotestas were spat for two species but those of a third were always swallowed. These behaviors may be influenced by ease of detachment of the food reward. Arils are attached only at the base of the seed, a morphology that may promote easier removal in comparison to sarcotestas, which are formed by the outermost cell layers of the seed coat and thus adhere tightly to the entire seed.

Hornbills swallow seeds with both arils and sarcotestas whole. Surprisingly, seeds of all *Xylopia* species, even the large seeds of *X.letestui* and *X.staudtii*, were reported by Whitney et al. (1998) to be defecated by the hornbills rather than regurgitated from the gizzard. Seed diameter of African *Xylopia* species ranges from 3.2 mm to 17.3 mm, but seeds of the species fed upon by the hornbills in the Whitney et al. (1998) study trended strongly toward the larger end of this range: three of the four species had seed diameters that can exceed 10 mm. From these data, it appears that hornbills preferentially select seeds with larger rewards.

Questions remain about the nutritional qualities of the food rewards. Nutrient and mineral analysis of the aril of *Xylopiastaudtii* by Sourd and Gautier-Hion (1986) showed that it had the highest fatty acid content and the third highest protein content as a percentage of dry weight of the 23 species of fruits and seeds analyzed. An analysis of four species of *Xylopia* by Lamperti et al. (2014) also indicated high fat and protein content, in addition to soluble carbohydrates and minerals. Aaron Lamperti (personal communication, July 2017) reports that the fruits and seeds were separated in this study and only the food tissues attached to the seeds were analyzed. If arils and sarcotestas provide protein and fat, these rewards could be significant sources of essential nutrients in the diet of obligate frugivores, even if not a large source of calories. Feeding preferences may also be directed by taste; for example, the arils of *X.quintasii* taste bitter (K. Abernethy, personal communication) but secondary chemical constituents have not been analyzed.

*Xylopia* seeds are eaten by several animals in addition to hornbills and monkeys. Gautier-Hion et al. (1985) suggested that seed predators such as squirrels and other small rodents may sometimes act as dispersers for *Xylopia*. Seeds of *X.aethiopica* were recovered from dung of the nocturnal genet cat (*Genetta* sp.) collected in the Shimba Hills of Kenya (T. Engel, personal communication); surprisingly, the aril was still intact. Smaller-seeded *Xylopia* species are likely to have a distinct suite of dispersers from those described here, as are the diverse orange-sarcotesta species from eastern and southern Africa.

At a community level, it is clear that multiple species of *Xylopia* interact with multiple dispersers. Chapman and Onderdonk (1998) argued for a co-dependency between primates and the plants on which they feed; in Congolian forests this certainly seems to be the case for *Xylopia*, not only with monkeys but with hornbills as well. Over 30 species of *Xylopia* occur in Congolian forests, all presenting a similar dispersal syndrome and perhaps reinforcing and sustaining these mutualisms. Conservation efforts must account for these co-dependencies.

### Ethnobiology

There are many reports of local use of *Xylopia* wood as a material for building and for tools (e.g. Tessmann 1913, Focho et al. 2010). Two species, *X.hypolampra* and *X.quintasii*, are used commercially for timber: the International Tropical Timber Organisation (ITTO, http://www.itto.int/) uses the trade names “ekui” for the former and “elo” for the latter.

Bark, roots, leaves, fruits, and seeds of *Xylopia* have all been used in medicines throughout western and central Africa. There have also been a few reports of use in perfumes and insecticides. The plants have been prepared in myriad ways for both external and internal use (Reviewed by Burkill 1985, Prelude Medicinal Plants Database: http://www.africamuseum.be/collections/external/prelude/view_plant?pi=13170. [accessed 01.08.2017]). Various species, most frequently *X.aethiopica*, have been used to treat a wide variety of maladies. We found no consistent pattern of efficacy either by malady or by locality, (e.g. Agbovie et al. 2002, Focho et al. 2010, Malan et al. 2015.) When taken together, however, the record of local uses provides strong evidence that extracts of each of the plant parts have significant bioactivity; this has been borne out by phytochemical analysis. Information on individual species is recorded in the taxonomic treatment.

Knowledge of *Xylopiaaethiopica* as a medicinal plant travelled out of Africa at an early time. The first record is possibly found in the Persian polymath Avicenna’s (Ibn Sina, 980–1037) work, the *Canon of Medicine*. We saw a Latin translation of this work, published in 1544, where, in Cap. 304, Avicenna describes the properties of seeds called “grano azelem” and claims that they “increase the sperm.” He compares the seeds to black pepper, points out their aromatic qualities and strong taste, and records that the plant comes from “Sceherazura.” The next reference to the plants comes several hundred years after Avicenna’s lifetime, from the *Book of Simple Medicaments* by the physician Serapion the Younger, later translated from the Arabic and published in Europe in various editions. Serapion (1531) refers to the hab zelim, or granum zelim, giving a brief description of the seed and its properties. In Italy, Matthioli (1565) described and illustrated *Piper AEthiopicum*, cited Serapion’s work, and noted that the fruits came through Alexandria in Egypt. Through the mid-eighteenth century, there are regular European references to the plants in herbals, but adding little new information. In the early 19^th^ Century, the plant was increasingly less known in Europe (Dunal 1817) but probably still available (Brown 1818).

The boundaries between food plants and medicinal plants is often only a matter of degree (Johns 1990) and, in contrast to the wide variation in medicinal uses, the use of the fruits and seeds of *X.aethiopica* as a drink and as a flavoring for savory foods is widespread and consistent across much of West and Central Africa. The fruits are widely available in local markets (e.g. Van Andel 2014, Maame Dontoh, personal communication). In modern times, from a base in Senegal, a coffee drink flavored with *X.aethiopica* called Café Touba has become widely popular. We have seen two commercially packaged brands in the U.S.; once again *X.aethiopica* is available far outside the wet forests where the plants grow.

Café Touba began as a ceremonial and special occasion drink of the Mourides, a Muslim brotherhood of Senegal whose founder, Sheikh Ahmadou Bemba, is said to have brought it from Gabon. There are of course many ways to prepare the drink, but the recipe given by Mbaye Niasse, in an interview by the BBC in Dakar, called for 5 liters of water, a kilo of coffee, and half a kilo of ground Guinea pepper (*Xylopiaaethiopica*). This mixture is then cooked for many hours (http://www.bbc.com/afrique/mobile/region/2012/08/120814_touba_senegal.shtml BBC 14 August 2012). Sugar to taste and sometimes cloves are added.

The fruits of *Xylopiaaethiopica* are processed by drying and may be smoked. Although there is no evidence of domesticated varieties, herbarium labels indicate that the plants are occasionally cultivated. Fruits can no doubt also be easily gathered from the wild. The plants grow quickly, are relatively common in African wet forests, and are often a second growth species. Occasionally, the plants have even been described as weeds (Savill and Fox 1967). The species has a limited distribution in Senegal and, for commercial production, fruits are said to be imported from Ivory Coast or from Gabon (http://cafetouba.coffee). While the fruits of all species of *Xylopia* we have encountered are strongly aromatic, species other than *X.aethiopica* seem to be of only incidental use (see Burkill 1985). There are, however, two reports on herbarium labels of cultivated X. *elliotiii* from the area of Bayangam, Cameroon.

Species of *Xylopia* contain a wide array of bioactive and fragrance compounds. Alkaloids, tannins, and saponosides were obtained by Yemoa et al. (2008) from the fruits of *X.aethiopica*. Types of constituents present vary from one organ to another on the same plant: Aguoru et al. (2016) qualitatively screened the petioles, seeds, leaves, bark, and roots of *X.aethiopica* for nine different types of molecules and found different chemical profiles in each plant part. Alkaloids, saponins, and flavonoids were present in all parts except the roots. Other types of biologically active molecules, which included anthraquinones, glycosides, and reducing sugars, were more limited in distribution. All species that have been tested contain pungently aromatic essential oils comprising a variety of terpenes. Terpenes have been extracted from bark (Yapi et al. 2012), leaves (Konan et al. 2009, Aguoru et al. 2016), fruits (Noudjou et al. 2007, Yemoa et al. 2008, Konan et al. 2009, Bakarnga-Via et al. 2014), and seeds (Aguoru et al. 2016). Konan et al. (2009) found the overall yield of essential oils extracted from *X.aethiopica* leaves to be 0.25% and from fruits 1.9%. *Xylopiaaethiopica* forms over 20% of the diet of red colobus monkeys in Korup National Park, Cameroon (Usongo and Amubode 2001) perhaps suggesting that terpenes provide stronger protection against herbivory in the fruits than in the leaves.

Among these secondary compounds, terpenes and alkaloids have been studied in the most detail. Terpene-containing oils from the fruits of *X.aethiopica* have shown anti-microbial activity against both Gram-positive and Gram-negative bacteria as well as fungi (e.g., Tatsadjieu et al. 2003, Fleischer et al. 2008, Hassan et al. 2014). Terpenes isolated from the fruits of *X.aethiopica* and *X.parviflora* [= *X.longipetala*] showed cytotoxicity against cancer cells (e.g., Konan et al. 2009, Bakarnga-Via et al. 2014). A wide variety of alkaloids have been isolated from *Xylopia* species, including the African species *X.aethiopica* and *X.longipetala* (listed as “*X.parviflora*”), and are reviewed by Lúcio et al. (2015). Anti-fungal properties may be attributable to oxoaporphine alkaloids, which were isolated from the leaves, twigs, and bark of *Xylopiaaethiopica* (Harrigan et al. 1994).

### Distribution, habitats, and conservation

**Figure 5.**
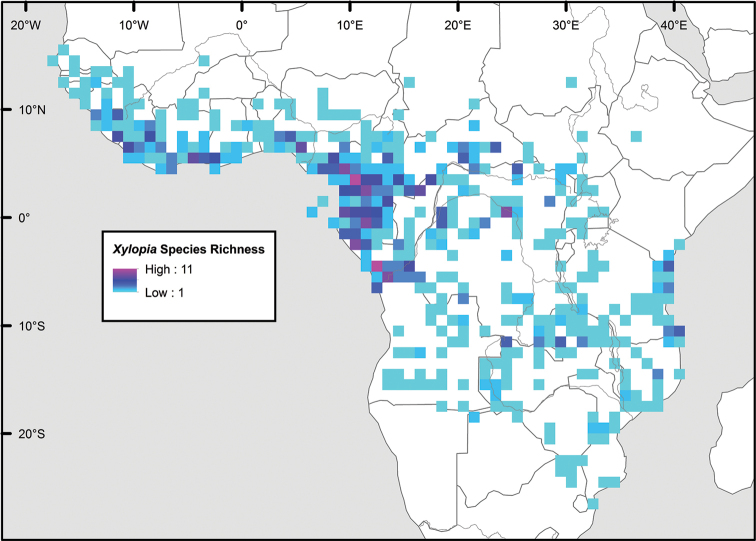
*Xylopia* species richness in Africa, plotted by 1° × 1° Lat × Long grid cell. Bolder lines represent country borders, fainter lines lakes and major rivers.

**Figure 6.**
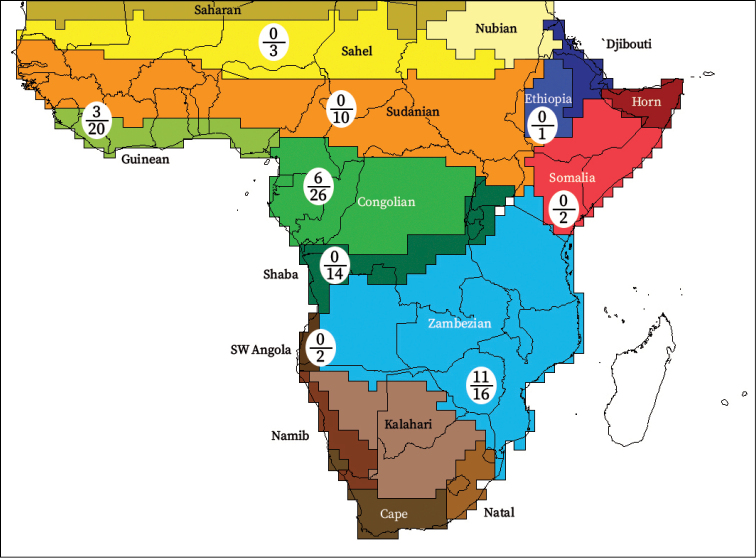
*Xylopia* species endemism by biogeographic region and subregion. For each region or subregion in which *Xylopia* species are present, the number above the line represents the number of endemic species and the number below the line the total number of species. Adapted from Figure 2 of Linder et al. (2012); figure used with the permission of the authors and of John Wiley & Sons, Inc.

The distribution of *Xylopia* in Africa extends from Senegal across the southern edge of the Sahel east to southern Sudan, and then south to southern Angola, northeastern South Africa, and southern Mozambique (Fig. 5). The majority of African *Xylopia* species occupy lowland tropical wet forest below 1000 m, a typical habitat for Annonaceae worldwide. The greatest concentration of *Xylopia* species occurs in the high rainfall countries of Cameroon and Gabon: together, the two countries contain 27 of the 45 African species. Habitat specializations include riparian species such as *X.longipetala*, *X.rubescens*, and *X.aurantiiodora*, and the montane species *X.africana* and *X.monticola*, which are only found between 650 and 2000 m. A number of species grow in seasonally dry woodlands, especially in southern and eastern Africa, an area of high endemism (Fig. 6) for the genus. Two species, *X.rubescens and X.aethiopica*, have large ranges that are nearly co-extensive with the African distribution of the entire genus. *Xylopiarubescens* appears to be an opportunistic wetland species. The distribution of *Xylopiaaethiopica*, a secondary forest species used as a spice and medicinal plant, has likely been augmented by human activity.

*Xylopia* occurs on islands in the Gulf of Guinea, which have a generally depauperate Annonaceae flora (Figueiredo et al. 2011, Velayos et al. 2013). Closest to the mainland, the island of Bioko has three or possibly four species (Velayos et al. 2013), Principe, the smallest island, has one species, and São Tomé has three species (Figueiredo et al. 2011). No species is endemic; only the widespread *Xylopiaaethiopica* occurs on all three islands. Other species include *Xylopiaafricana*, *X.quintasii*, and *X.rubescens*. Monkey and hornbill species known to disperse *Xylopia* seeds are present only on Bioko.

We mapped species richness of the genus to a 1 degree (i.e. ~ 100 km²) grid and found up to 11 species per cell (Fig. 5). The area with the greatest concentration of high-diversity cells runs in a band from southwestern Cameroon to western Democratic Republic of the Congo. The two cells, with the highest *Xylopia* species richness on the continent, fall within this wet forest area, one in southern Cameroon and the other including the Cabinda Province of Angola and parts of adjacent Republic of the Congo and Democratic Republic of the Congo. Both cells contain six of the same widely distributed species, but otherwise vary. Some single grid cell “hotspots” for high diversity, such as the Yangambi region of north-central Democratic Republic of Congo, are known to represent areas of intensive collecting, a bias observed previously by Sosef et al. (2017). Overall, the striking pattern of high species richness in *Xylopia* corresponds well to the western portion of the “Wet Central Africa” floristic cluster generated by Fayolle et al. (2014), which falls within the Congolian Biogeographic Subregion, as defined by Linder et al. (2012) and shown in Fig. 6.

*Xylopia* occurs in all seven of the sub-Saharan African biogeographic Regions defined by Linder et al. (2012) (Fig. 6). The areas with highest numbers of species are the wet forest Congolian Region, which includes the Guinean, Congo, and Shaba Subregions on the map, with 32 species, the southern dry forest Zambezian Region with 16 species, and the northern dry forest Sudanian Region with ten. Nearly 47% of African *Xylopia* species are restricted to the Congolian Region and 22% to the Zambezian Region, illustrating the high *Xylopia* species turnover between these two Regions. No other Region has endemic species of *Xylopia*, but the distributions of *X.elliotii* and *X.nilotica* are centered in the Sudanian Region.

In the Guinean Subregion (Fig. 6), the lowland wet forest is discontinuous for a short distance between southern Togo and southwestern Nigeria. This dry forest area, sometimes termed the Dahomey Gap, was proposed by White (1979, 1983) as a biogeographic boundary between western and eastern lowland wet forest species, but this boundary has not been maintained in recent biogeographic analyses (Fayolle et al. 2014, Linder 2014). Some *Xylopia* distributions in West Africa, however, do correspond to this boundary: three narrowly distributed species, *X.acutiflora*, *X.dinklagei*, and *X.piratae*, all segregates of the former *X.acutiflora* s. l., have distributions ending to the west of the dry forest area, while three species, *X.calva*, *X.cupularis*, and *X.thomsonii*, have distributions ending in south-central Nigeria east of the dry forest area. In contrast, *Xylopiavillosa* occurs on both sides of the dry forest area, along with six more widespread lowland wet forest *Xylopia* species.

In the Zambezian Region (Fig. 6), eight species are narrow endemics found in dry coastal forests from southern Kenya to southern Mozambique. This area has been the subject of several biogeographic analyses of its flora and fauna (Lovett and Wasser 1993, Timberlake et al. 2011). While Linder’s (2014) analysis did not recognize this region as distinct, Fayolle et al. (2014) argued for the biogeographic uniqueness of this coastal biota. In short, the center of diversity, both at the species level and at the level of major clades in the genus, lies in the Congolian Region, but in *Xylopia* the center of endemism lies in the coastal area of the Zambezian Region.

**Figure 7.**
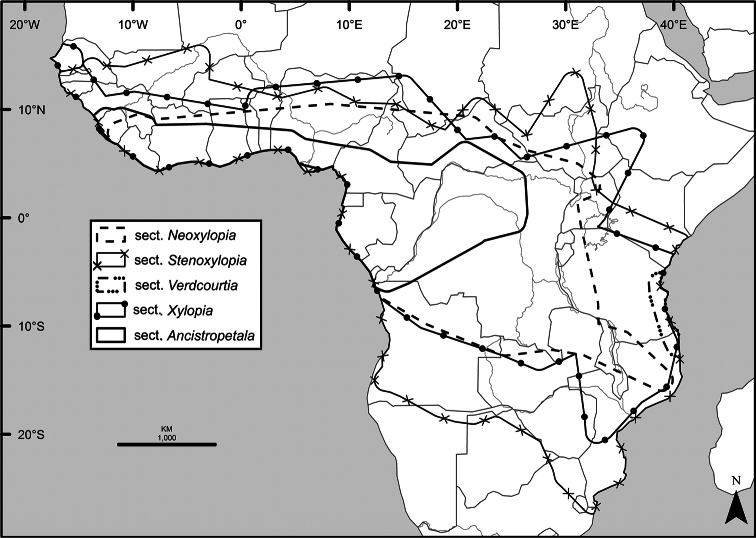
Aggregate distributions of the sections of *Xylopia* present in Africa. Distributions are as described in this work, based on the phylogenetic analysis of Stull et al. (2017), but with the addition of sect. Verdcourtia. Bolder lines represent country borders, fainter lines lakes and major rivers.

**Figure 8.**
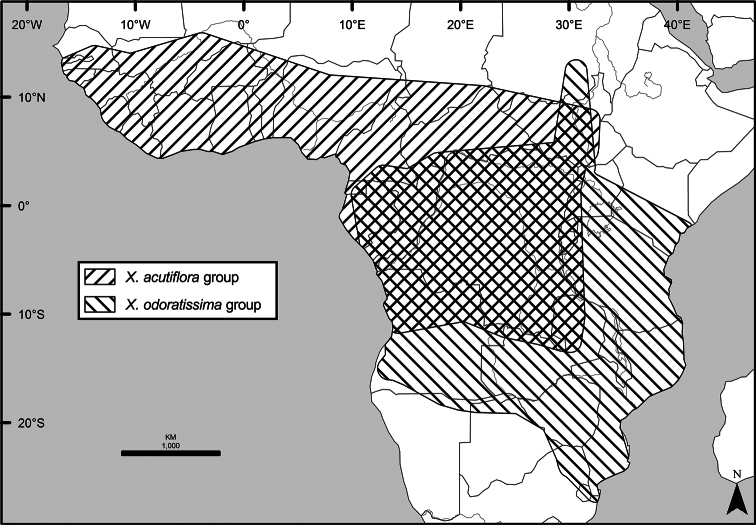
Aggregate distributions of the *Xylopiaacutiflora* and *X.odoratissima* groups of section Stenoxylopia in Africa. Bolder lines represent country borders, fainter lines lakes and major rivers.

Africa is the only continent where all major clades of *Xylopia* are represented (Fig. 7). Species of all sections in Africa, except the East African section Verdcourtia, occupy Linder’s Congolian Region. This lowland wet forest area forms the center of diversity for the major clades of *Xylopia* worldwide (Stull et al. 2017). The drylands diversification of *Xylopia* in southern and eastern Africa has arisen within section Verdcourtia and the *X.odoratissima* group of sect. Stenoxylopia. Section Stenoxylopia occupies the widest range of any section, but the two groups delimited within it show geographic structure (Fig. 8): the *X.acutiflora* group extends from Senegal east to South Sudan and south to central Angola and eastern Zambia, while the *X.odoratissima* group extends from western Gabon east to southern Sudan and the Kenyan coast and south to northern Namibia and southern Mozambique. These distinct distribution areas suggest separate radiations, but our molecular phylogenetic analysis (Stull et al. 2017) lacks adequate resolution to support that hypothesis.

While a number of African species of *Xylopia* are widespread, over half of the species on the continent have limited distributions and are potentially threatened (Table 1). Full IUCN conservation assessments have been completed for only six African *Xylopia* species. We therefore quantified distributions for the 24 least widely distributed species (including some of those assessed previously), following IUCN guidelines (IUCN Species Survival Commission 2012) for determining the EOO and the AOO (Table 1). Local field work is needed to evaluate the remaining conservation criteria. All 24 species were found to have AOOs below 100 km^2^. As might be expected, species including *X.keniensis* and *X.tenuipetala* show both a small EOO and a small AOO. However, species including *X.paniculata* and *X.pynaertii*, which occupy relatively large EOOs, also have AOOs under 100 km^2^. Noteworthy is the fact that eight of the species are confined to the Zambezian Region (sensu Linder et al. 2012), underscoring the fact that the East African dry forests are a center for rare *Xylopia* species of limited distribution.

**Table 1.** Conservation assessment data for 28 African species of *Xylopia*. Threat status was taken from the IUCN Red List on 3 January 2018. Extent of Occurrence (EOO), Area of Occupancy (AOO), and number of localities based on the present study. IUCN status abbreviations are DD = Data Deficient, EN = Endangered, NT = Near Threatened, VU = Vulnerable. Footnoted references for published non-IUCN conservation assessments are † = Onana and Cheek 2011, ‡ = Johnson et al. 2017.

Species	Status	EOO (km^2^)	AOO (km^2^)	Localities
**IUCN Red List**				
*X.africana*	VU	–	–	14
*X.arenaria*	VU	6454	58	8
*X.collina*	EN	12,374	76	10
*X.elliotii*	VU	–	–	40
*X.mwasumbii*	EN	3702	60	6
*X.talbotii*	VU	27,199	20	5
**Preliminary Assessments**				
*X.acutiflora*	DD	103,286	68	12
*X.aurantiiodora*	VU†	–	–	23
*X.calva*	DD	22,406	12	3
*X.congolensis*	DD	169,980	28	4
*X.dinklagei*	NT	4063	28	3
*X.flamignii*	VU†	108,538	32	8
*X.gilbertii*	VU†	249,251	52	6
*X.globosa*	DD	83,797	20	5
*X.keniensis*	EN‡	4	10	2
*X.letestui*	EN†	–	–	19
*X.lukei*	EN‡	2656	22	4
*X.mildbraedii*	EN†	129,258	36	9
*X.monticola*	DD	14,393	36	5
*X.paniculata*	DD	105,473	16	4
*X.piratae*	DD	31,476	64	11
*X.pynaertii*	DD	582,432	40	10
*X.tanganyikensis*	EN‡	527	10	3
*X.tenuipetala*	EN‡	250	13	3
*X.torrei*	DD	7053	12	3
*X.toussaintii*	DD	6766	16	4
*X.unguiculata*	DD	15,417	20	4
*X.wilwerthii*	DD	179,602	52	13

## Taxonomic treatment

### Xylopia

Taxon classificationPlantaeMagnolialesAnnonaceae

Linnaeus, Syst. nat., ed. 10, 2: 1250 [+1378]. 1759, nom. conserv.

EE7DF0D1-CB2D-5400-9834-3149AE923A3B

Xylopicrum P. Browne, Hist. Jamaic. 250–251 + t. 5, fig. 2. 1756. Xylopicron, orth. mut., Adanson, Fam. 2: 365. 1763. Type: Xylopiamuricata Linnaeus, typ. conserv. (lectotype designated by Setten and Maas, Regnum Vegetabile 127: 99, 1993: *Browne* s. n., Herb. Linn. No. 1077.1 (LINN)).
Unona Linnaeus f., Suppl. pl. 270. Apr 1782. Bulliarda Necker, Elem. bot. 2: 321. 1790, nom. superfl., non Candolle, 1801. Type: Unonadiscreta Linnaeus f.
Krockeria Necker, Elem. bot. 2: 317–318. 1790. Type: Unonaconcolor Willdenow (lectotype, here designated).
Coelocline A. de Candolle, Mém. Soc. Phys. Genève 5: 208–209. 1832. Type: Coeloclineacutiflora (Dunal) A. de Candolle.
Parartabotrys Miquel, Fl. Ned. Ind., Eerste bijv. 3: 374. 1860. Type: Parartabotryssumatranus Miquel. Note: Name only appears as a nomen nudum in Fl. Ned. Ind., Eerste bijv. 1: 154, 1860.
Pseudanona (Baillon) Safford, J. Wash. Acad. Sci. 3: 17. 1913, as “Pseudannona.” XylopiaSectionPseudanona Baillon, Adansonia 4: 141–142. Jan. 1864. Type: Pseudanonaamplexicaulis (Lamarck) Safford (lectotype, designated by Safford, J. Wash. Acad. Sci. 3: 18. 1913).

#### Description.

***Trees*** up to 50 m tall with a straight bole rising to a small conical to rounded crown, with narrow buttresses or, in a few species, stilt roots at the base of the trunk, or ***shrubs***, the branches, in a few species, lianescent; bark smooth to scaly, variable in color. Indument of simple hairs. ***Twigs*** persistently pubescent to glabrate, usually sparsely lenticellate, longitudinally wrinkled; nodes in many African species with two axillary branches. ***Leaf*** with blades chartaceous to coriaceous, lanceolate, oblong, elliptic, ovate, oblanceolate, or obovate, apex rounded to acuminate, base cuneate to rounded, sometimes decurrent on petiole, midrib impressed to slightly raised adaxially, raised abaxially, secondary veins brochidodromous to weakly brochidodromous, petiole short, usually shallowly canaliculate. ***Inflorescences*** axillary, occasionally arising from the leafless portions of branches, 1–32-flowered; peduncles 1 to several per axil or absent, pedicels 1–several per peduncle, with 1–6 small caducous to persistent bracts; buds ovoid to linear, apex obtuse to acute. ***Flowers*** bisexual. ***Sepals*** 3, usually connate at the base and forming a cuplike calyx, in a few species free and imbricate at base. ***Petals*** 6, in 2 series of three, free, white, cream-colored, pale yellow, pale orange, or, in a few species, red *in vivo*; outer petals erect, slightly spreading, or curved outward at anthesis, ovate to linear, flattened to a concave base adaxially, flat, often with a faint ridge, abaxially; inner petals slightly shorter and narrower (much shorter in *X.rubescens*) than outer petals, ovate to linear, keeled on both surfaces but becoming concave at the base adaxially and flat at the base abaxially, often narrowed into a short claw; in a few species, the margins of the basal concavity are differentiated from the tissue of the rest of the petal. ***Stamens*** 40–200; fertile stamens clavate to oblong, apex of connective shieldlike, conical, globose, or rudimentary, often overhanging anther thecae, pubescent or papillate, rarely glabrous, anthers transversely septate, 4–24-locellate, filament much shorter than anther, articulated with the staminal cone; outer staminodes oblong to clavate, apex obtuse to obliquely truncate, rarely absent; inner staminodes often adhering to the bases of the stigmas, usually shorter than outer staminodes, clavate to oblong, apex rounded, rarely absent; filament bases connate into a staminal cone that partially or completely encloses the ovaries, the rim even or sometimes laciniate, or staminal cone absent. ***Carpels*** 2–50; ovaries oblong or ovoid, usually hairy, stigmas free or loosely connivent with tips spreading, linear, falciform, narrowly oblong, or clavate, smooth or studded with round tubercles in some species, often hairy at the apex. ***Torus*** flat or slightly concave. ***Fruit*** of up to 36 glabrate to pubescent dehiscent monocarps borne on a short woody pedicel and slightly expanded torus. ***Monocarps*** with green, red, or purple exterior and a green or pink to scarlet endocarp *in vivo*, linear, oblong, ovoid, or globose, often somewhat falcate, occasionally weakly torulose, apex rounded or with a curved beak or mucro, base contracted into a stipe or sessile, smooth, longitudinally ridged, finely wrinkled, or verrucose; pericarp leathery to woody when dried. ***Seeds*** up to 20 per monocarp, attached laterally in one or two rows, lying parallel, oblique, or perpendicular to long axis, oblong to ellipsoid, oblong, elliptic, or ovate in cross section, flattened at micropylar end, rounded at chalazal end, brown to black, smooth or rarely slightly pitted, dull or shiny, raphe/antiraphe forming a raised ridge encircling the seed or flat and not evident, micropylar scar elliptic to circular; seeds arillate, the aril attached around the micropyle, white, yellow, orange, red, pink, or violet *in vivo*, fleshy or papery, or aril absent; seed coat with a fleshy outer layer (sarcotesta) and hard inner layer, or sarcotesta absent.

A pantropical genus of ca. 180 species, in tropical Africa represented by 45 species. The only other African Annonaceae genus with elongate flower buds such as those commonly seen in *Xylopia* is *Greenwayodendron* Verdc., and the latter genus is sometimes mistaken for *Xylopia* on this basis; *Greenwayodendron* species, however, always have terminal inflorescences, which appear supra-axillary or leaf-opposed (Verdcourt 1969). The dehiscent monocarps set *Xylopia* apart from all other African Annonaceae genera except the East African *Mkilua* Verdc. (Verdcourt 1970) and *Ophrypetalum* Diels, neither of which has a brightly colored endocarp or seeds.

*Patonia* Wight (Wight 1838) is usually treated as a generic synonym of *Xylopia* (van Setten and Maas 1990, Turner 2011). In his generic description, however, Wight explicitly described and mentioned an apical ovule and seed, and a calyx that grows up to cover the maturing fruit. These are characters of *Diospyros*, not of Annonaceae. We suggest that Hiern was correct in adopting the name *Patonia* as a synonym of *Diospyros* and that *Patoniawalkeri* Wight should be its type, as the lectotype designated is in conflict with the generic protologue. *Patoniaparvifolia*, the other species placed in the genus when it was proposed, is a *Xylopia*, now re-named *Xylopiapatoniae* I. M. Turner for nomenclatural reasons (Turner 2011).

#### Key to the African species of *Xylopia*

**Table:** 

1	Seeds arillate, sarcotesta absent; nodes with either one or two axillary branches; staminal cone rudimentary or absent, if well developed then rim of cone even and carpels and monocarps more than 22	**2**
–	Seeds not arillate, but a fleshy sarcotesta present on the seed (waxy layer that scratches off); some nodes with two axillary branches; staminal cone well developed but usually with an irregular laciniate rim, carpels and monocarps never more than 22, often many fewer (sect. Stenoxylopia)	**12**
2	Aril white (seed unknown for *X.lukei*); apex of anther connective rudimentary, not overhanging anther thecae; inner petals completely glabrous (sect. Verdcourtia)	**3**
–	Aril yellow, orange, red, or purple; apex of anther connective evident, overhanging anther thecae; inner petals with some hairs on both surfaces, especially toward the apex	**5**
3	Larger leaf blades 8.2–11.9 cm long, 4.1–5.8 cm wide	***X.lukei***
–	Larger leaf blades 4.6–8.7 cm long, 2.3–4.3 cm wide	**4**
4	Pedicels 2.4–3.5 mm long; leaf abruptly blunt-acuminate, the acumen 4–8 mm long, occasionally emarginate or obtuse; petals coriaceous to slightly fleshy	***X.mwasumbii***
–	Pedicels 6.7–7.2 mm long; leaf gradually acuminate, the acumen 4–11 mm long; petals membranous	***X.tenuipetala***
5	Aril bilobed, monocarps up to 36 per fruit; staminal cone well developed, completely enclosing the ovaries; leaf gradually long-acuminate, the acumen 6–20 mm long (sect. Xylopia)	***X.aethiopica***
–	Aril not bilobed, monocarps up to 15 per fruit; staminal cone absent or rudimentary in the form of a ring only covering the bases of the ovaries; leaves short-acuminate (up to 6 mm long) or, if longer, then sharply cuspidate, not gradually acuminate	**6**
6	Arils membranous, fimbriate, covering at least half and often the entire seed; stigmas usually discrete; inner petals with differentiated fleshy margins around basal concavity; apex of anther connective conical to capitate and papillate to long-papillate, giving a bumpy rather than flat surface to the androecium (sect. Ancistropetala)	**7**
–	Arils brushlike, covering only base of seed; stigmas more or less connivent; inner petals with margins undifferentiated; apex of anther connective flat or with a slight bump in the center and often pubescent, but forming a flat surface to the androecium (sect. Neoxylopia)	**9**
7	Leaves narrowly elliptic, acute to obtuse at the apex; pubescence of outer petals buff-brown; inner petal with a distinct truncate tooth overhanging basal concavity; stigma subequal in length to ovary; seeds 13.5–21 mm long, pointed at the chalazal end	***X.aurantiiodora***
–	Leaves oblanceolate to obovate, short-acuminate at the apex; pubescence of outer petals silvery to brownish gray; inner petal lacking tooth overhanging basal concavity; stigma distinctly shorter than ovary; seeds 10–12.5 mm long, rounded at chalazal end	**8**
8	Inflorescences usually 2-flowered, rarely up to 4, pedicels all arising independently from the axil, often recurved; outer petals chartaceous, up to 21 mm long; leaf flush white; leaf blades glabrous abaxially	***X.congolensis***
–	Inflorescences up to 7-flowered, some pedicels attached to a common peduncle arising from the axil; pedicels straight or sinuous; outer petals more or less fleshy, up to 13 mm long; leaf flush red to purple; leaf blades usually appressed-pubescent abaxially, sometimes glabrate	***X.quintasii***
9	Outer petals much longer than inner petals; monocarps strongly torulose, often moniliform	***X.rubescens***
–	Outer and inner petals subequal in length; monocarps torulose or not, but not moniliform (fruit of *X.globosa* unknown)	**10**
10	Leaves oblong or elliptic, widest near midpoint, 15.7–23.5 cm long, 8.3–11.7 cm wide	***X.globosa***
–	Leaves oblanceolate to obovate, widest distal to midpoint, 5.1–15.4 cm long, 2.0–7.9 cm wide	**11**
11	Sepals 2.6–3.5 mm wide; outer petals 5.8–9.6 mm long; aril usually yellow to orange *in vivo*; monocarps at most weakly torulose	***X.staudtii***
–	Sepals 4.6–5.5 mm wide; outer petals 8.2–13.5 mm long; aril blood-red *in vivo*; monocarps distinctly torulose	***X.africana***
12	Inner petals thickened and knobby on margins toward base; sarcotesta orange to red *in vivo*	**13**
–	Inner petals undifferentiated on the margins; sarcotesta orange to red or white to pale green *in vivo*	**15**
13	Leaves glabrous adaxially, veins forming a conspicuous reticulum on both surfaces; monocarps linear, glabrate, up to 22 per fruit	***X.flamignii***
–	Leaves pubescent adaxially, veins at most only slightly raised on either surface; monocarps oblong, sparsely pubescent, up to 10 per fruit	**14**
14	Sepals 2.8–4.2 mm wide; outer petals lanceolate to ovate, 6.6–11 mm long, 2.4–4.4 mm wide at midpoint; leaves usually pubescent across entire surface, rarely only along midrib adaxially	***X.gilbertii***
–	Sepals 1.9–2.3 mm wide; outer petals linear-lanceolate, 8.7–11.8 mm long but only 0.6–1.0 mm wide at midpoint; leaves always pubescent only along midrib adaxially	***X.toussaintii***
15	Leaves persistently appressed-pubescent abaxially, the hairs overlapping and forming a visible indument; seeds in two rows, oblique to perpendicular to long axis of monocarp	**16**
–	Leaves pubescent to glabrate abaxially, if persistently pubescent then hairs not appressed or hairs small and not overlapping; seeds in one or two rows, usually oblique to long axis of monocarp	**19**
16	Leaves silvery-sericeous abaxially; monocarps sessile, brown, strongly lenticellate, glabrate	***X.hypolampra***
–	Leaves with golden, gray, or brown pubescence abaxially; monocarps sessile or stipitate, green to brown, not lenticellate, pubescent or glabrate	**17**
17	Monocarps clearly stipitate, the stipes 7–24 mm long; leaf base cuneate to broadly cuneate, often oblique; flower pedicels 4.5–12 mm long	***X.cupularis***
–	Monocarps sessile, narrowing gradually toward base; leaf base truncate, rounded, or at most broadly cuneate, symmetrical; flower pedicels 1.4–6.9 mm long	**18**
18	Leaf blade truncate at base, obtuse to acute at the apex, hairs appressed but not shiny abaxially; inflorescences with up to 4 flowers	***X.letestui***
–	Leaf blade broadly cuneate to rounded at base, acute to acuminate at the apex, golden-sericeous abaxially; inflorescences with up to 8 flowers	***X.villosa***
19	Inflorescences with up to 32 flowers; seeds 15.7–17.3 mm wide	***X.paniculata***
–	Inflorescences with up to 10 flowers; seeds ≤ 10 mm wide	**20**
20	Secondary veins arcuate, diverging from the midrib at an angle of ca. 45° but continuously curving toward the apex, these and higher-order veins raised on both surfaces; rim of staminal cone even; monocarps ovoid, oblong, or broadly ellipsoid, 1.7–3.1 cm wide and thick	***X.phloiodora***
–	Secondary veins diverging straight from the midrib at an angle of 50–70°, these and higher-order veins plane, rarely raised and reticulate abaxially; rim of staminal cone irregularly laciniate; monocarps narrowly oblong to linear, 0.5–1.5 cm wide and thick	**21**
21	Most pedicels with 3–6 bracts, these often imbricate and more or less persistent, even in fruit; pedicels 1 or rarely 2 per axil	**22**
–	Most pedicels with 2 bracts, these often separated and with the upper persistent in flower and the lower caducous, not usually present in fruit; pedicels 1–12 per axil (some inflorescences with >1 pedicel)	**30**
22	Twigs covered with erect hairs uniformly 1.5–2.0 mm long	***X.talbotii***
–	Twigs covered with appressed hairs 0.1–0.5 mm long or with a mix of short appressed hairs and longer erect hairs up to 1.3 mm long	**23**
23	Hairs on twigs appressed and of uniform length, 0.1–0.5 mm long	**24**
–	Hairs on twigs a mix of short appressed hairs 0.1–0.4 mm long and longer erect hairs up to 1.3 mm long	**26**
24	Outer petals 2.2–4.1 mm wide at midpoint; seeds 12.5–14 mm long; leaf blades 9.5–17.5 cm long, 3.2–5.6 mm wide	***X.mildbraedii***
–	Outer petals 1.0-2.0 mm wide at midpoint; seeds 9.2–12.3 mm long; leaf blades 6–13.7 cm long, 1.8–4.7 mm wide	**25**
25	Outer petals 10.5–13 mm long; coastal Liberia	***X.dinklagei***
–	Outer petals (14.6–) 22–49 mm long; central Africa	***X.thomsonii***
26	Shrubs scandent on low vegetation, ultimate twigs usually thickened at the base; outer petals 24.3–73 mm long, 0.9–1.5 mm wide at midpoint; coastal Ivory Coast and Ghana	***X.piratae***
–	Shrubs or trees, usually upright but sometimes with lianescent branches, ultimate twigs not thickened at the base; outer petals 19.6–52 mm long, 1.0–2.5 mm wide at midpoint	**27**
27	Outer petals 2.2–2.5 mm wide at midpoint; beak on monocarps up to 5 mm long, narrow and curved; always treelike in habit	***X.unguiculata***
–	Outer petals 1–2 mm wide at midpoint; beak on monocarps broad, blunt, and up to 4 mm long or often absent; treelike or lianescent shrub habit	**28**
28	Monocarps with seeds in two rows, 1.1–1.5 cm wide, not torulose, stipes 3–7 mm long; Sierra Leone, Liberia, Guinea, and Ivory Coast	***X.acutiflora***
–	Monocarps with seeds in a single row, 0.6–1.2 cm wide, torulose, stipes 3–13 mm long; Nigeria eastward	**29**
29	Montane tree; monocarp stipes 8–13 mm long	***X.monticola***
–	Lowland lianescent shrub or small tree; monocarp stipes 3–11 mm long	***X.thomsonii***
30	Larger leaf blades 10–17.2 cm long, 3.6–6.5 cm wide; outer petals 2.8–5.5 mm wide at the midpoint, the adaxial surfaces glabrous except at the apex	***X.calva***
–	Larger leaf blades 3.6–11.4 cm long, 1.2–5.6 cm wide; outer petals 0.5–2.5 mm wide at the midpoint, the adaxial surfaces mostly pubescent	**31**
31	Sepals reflexed at anthesis; petals membranous to chartaceous, the apices lax and crinkled when dry; stigmas 3.8–7 mm long; flower pedicels 6.2–12 mm long; monocarps often longitudinally ridged	***X.longipetala***
–	Sepals erect to slightly spreading at anthesis; petals coriaceous to fleshy, the apices rigid or bent outward when dry; stigmas 1.3–4.4 mm long; flower pedicels 2.8–8.2 mm long; monocarps sometimes obliquely wrinkled but not longitudinally ridged	**32**
32	Adaxial surface of leaf blades uniformly pubescent to sparsely pubescent, the hairs sometimes denser along the midrib	**33**
–	Adaxial surface of leaf blades completely glabrous or with hairs confined to the midrib	**39**
33	Shrubs; outer petals erect, inner petals sharply bent outward at the base at anthesis; endocarp of mature monocarp pink to red	**34**
–	Small to large trees; outer and inner petals both curved outward from the base, the apices incurved at anthesis; endocarp of mature monocarp green (white, possibly immature, in *X.holtzii*)	**35**
34	Hairs on twigs erect, 1–2 mm long, flowers red, monocarps glabrous and somewhat pruinose	***X.collina***
–	Hairs on twigs matted and twisted, 0.2–0.6 mm long, flowers white to cream-colored, monocarps pubescent, not pruinose	***X.tomentosa***
35	Most leaves relatively narrow, the larger blades 6.1–11.4 cm long, 1.5–4.4 mm wide; some inflorescences usually branched, (2–) 3 or more flowers in an inflorescence; monocarps either uniformly pubescent or strongly verrucose	**36**
–	Most leaves relatively broad, the larger blades 3.3–10.2 cm long, 1.7–5.1 cm wide; inflorescences rarely branched, 1–2 (–6)-flowered, the flowers usually arising from the same axil on separate pedicels; monocarps sparsely pubescent to glabrate, smooth to minutely verrucose	**37**
36	Higher-order veins less prominently raised than the secondary veins, forming only a faint reticulum on the adaxial surface; larger leaf blades 7.4–11.4 cm long; monocarps obliquely wrinkled and minutely verrucose (visible with hand lens), stipe 3–3.5 mm thick at the midpoint; coastal Kenya and Tanzania	***X.holtzii***
–	Higher-order veins equal in prominence to the secondary veins, forming a conspicuous raised reticulum on the adaxial surface; larger leaf blades 6.5–9.2 cm long; monocarps conspicuously verrucose but not much wrinkled, stipe 3.5–5 mm thick at the midpoint; Sudan to Uganda	***X.nilotica***
37	Flower buds abruptly narrowed 3–4 mm above the base and then tapering to apex; outer petals usually 21–30 mm long, rarely shorter, and 3.4–3.9 mm wide at the base; monocarps strongly rugose, short-stipitate, 2.5–7 mm long, or sessile	***X.odoratissima***
–	Flower buds tapering gradually to apex; outer petals at most 21 mm long, usually shorter, and 2.3–3.3 mm wide at base; monocarps obliquely finely wrinkled, distinctly stipitate, the stipe 3.5–11 mm long	**38**
38	Base of leaf blade broadly cuneate, occasionally cuneate or rounded; flower pedicels slender, 0.3–1 mm thick	***X.gracilipes***
–	Base of leaf blades rounded, occasionally broadly cuneate, truncate, or subcordate; flower pedicels 1.2–1.5 mm thick	***X.shirensis***
39	Pedicel of flower 8.5–21.5 mm long; leaves acuminate to caudate, the acumen 5–16 mm long, rounded at the tip; secondary veins 15–22 per side	***X.wilwerthii***
–	Pedicel of flower 0.4–9.1 mm long; leaf apex rounded, emarginate, obtuse, or acute, if acuminate the acumen either sharp-pointed or less than 11 mm long; secondary veins 7–14 (–17) per side	**40**
40	Leaf blades glabrous or only with a few scattered hairs abaxially; inflorescences (1–) 2–12-flowered, with all pedicels usually branching from a single peduncle	***X.katangensis***
–	Leaf blades with a sparse to dense but uniform covering of hairs abaxially; inflorescences 1–8-flowered, pedicels arising separately from the axils or sometimes with multiple peduncles from the same axil	**41**
41	Monocarps sessile, with pink to red endocarp; seeds in two rows, with orange sarcotesta; bracts on flower pedicel 2, both attached distal to midpoint of pedicel	***X.keniensis***
–	Monocarps stipitate, the stipe 1.5–11 mm long, endocarp pink to red or green; seeds in one or two rows, with pale gray to green or orange sarcotesta; bracts on flower pedicel 2–4, attached at various points but at least one attached proximal to midpoint of pedicel (may be caducous but scar will be present)	**42**
42	Monocarps oblong to ovoid, not torulose, 2.6–5.4 cm long, 1.3–2.0 cm wide and thick	**43**
–	Monocarps narrowly oblong, sometimes slightly torulose, 1.6–5 cm long, 0.5–1.4 cm wide and thick	**44**
43	Upper bark red, rough, and scaly; outer petals 15.2–20.5 cm long; petiole 1–2.5 mm long	***X.pynaertii***
–	Upper bark light gray, finely fissured or scaly; outer petals 28–38 mm long; petiole 2–4 mm long	***X.tanganyikensis***
44	Some inflorescences with flowers branching from a common peduncle, (1–) 2–8-flowered; monocarps either uniformly pubescent or strongly verrucose	**45**
–	Inflorescences with the flowers arising from the same axil on separate pedicels, seldom branching from a common peduncle, 1–2-, rarely 4-flowered; monocarps with irregular patches of sparse pubescence or glabrate, smooth to minutely verrucose	**47**
45	Shrub or sub-shrub 0.4–4 m tall; larger leaf blades 2.5–6 cm long	***X.tomentosa***
–	Tree up to 25 m tall; larger leaf blades 6.5–11.4 cm long	**46**
46	Higher-order veins less prominently raised than the secondary veins, forming only a faint reticulum on the adaxial surface; larger leaf blades 7.4–11.4 cm long; monocarps obliquely wrinkled and minutely verrucose (visible with hand lens), stipe 3–3.5 mm thick at the midpoint; coastal Kenya and Tanzania	***X.holtzii***
–	Higher-order veins equal in prominence to the secondary veins, forming a conspicuous raised reticulum on the adaxial surface; larger leaf blades 6.5–9.2 cm long; monocarps conspicuously verrucose but not much wrinkled, stipe 3.5–5 mm thick at the midpoint; Sudan to Uganda	***X.nilotica***
47	Twigs densely erect-hairy; seeds in two rows and perpendicular to long axis of monocarp; riparian forests from Sierra Leone to the Central African Republic	***X.elliotii***
–	Twigs sparsely lax- or appressed-pubescent; seeds in a single row and oblique to long axis of monocarp; forests and open country of eastern and southern Africa	**48**
48	Flower buds ovoid-conic; outer petals 6.6–10.2 mm long, inner petals 4.7–7.2 mm long; endocarp pink to scarlet	***X.arenaria***
–	Flower buds linear-lanceolate to linear; outer petals 9.5–30.4 mm long, inner petals 6.1–15.3 mm long; endocarp green	**49**
49	Pubescence of younger shoots consisting of straight, erect to appressed hairs; petals chartaceous, linear-filiform; monocarps 2.8–3.1 cm long; southern Mozambique in sandy habitats near the coast	***X.torrei***
–	Pubescence of younger shoots consisting of kinked, bent, and twisted hairs; petals coriaceous, linear-subulate; monocarps 2.8–5.0 cm long; widespread species of southern Tanzania, Mozambique, eastern Zimbabwe, and northeastern South Africa	***X.gracilipes***

### Synoptic character list

Below are characters that are relatively noticeable and occur in only a small number of species. Species for which a character is unknown are not included, even when a prediction could probably be made. Not all species are included. Species are listed in alphabetical order.

Plants of scandent habit: *X.dinklagei*, *X.piratae*, *X.thomsonii*

Stilt roots: *X.africana*, *X.paniculata*, *X.phloiodora*, *X.rubescens*, *X.staudtii*

Bark scaly or peeling, orange to reddish brown: *X.congolensis*, *X.pynaertii*, *X.quintasii*, *X.staudtii*, *X.villosa*

Leaves abaxially shiny from appressed hairs: *X.cupularis*, *X.hypolampra*, *X.villosa*

Leaves lanceolate, truncate at base: *X.letestui*

Abaxial surface of leaves reddish brown: *X.rubescens*

Twigs with some or all hairs erect and 1–2 mm long: *X.acutiflora*, *X.monticola*, *X.piratae*, *X.pynaertii*, *X.talbotii*, *X.thomsonii*, *X.unguiculata*, *X.villosa*

Sepals reflexed at anthesis: *X.longipetala*

Outer petals ovate (no more than twice as long as wide): *X.africana*, *X.flamignii*, *X.gilbertii*, *X.globosa*, *X.staudtii*

Petals red to brown: *X.collina*, *X.flamignii*, *X.gilbertii*

Petals often greater than 5 cm long, linear: *X.aethiopica*, *X.longipetala*, *X.mildbraedii*, *X.monticola*, *X.piratae*, *X.thomsonii*

Inner petals with differentiated margins at base: *X.aurantiiodora*, *X.congolensis*, *X.flamignii*, *X.gilbertii*, *X.quintasii*, *X.toussaintii*

Outer petals spreading and inner petals erect at anthesis: *X.lukei*, *X.mwasumbii*, *X.tenuipetala*

Outer petals erect with inner petal apices emerging at right angles between them: *X.arenaria*, *X.collina*, *X.keniensis*, *X.tomentosa*

Anthers over 20-locellate: *X.phloiodora*

Staminal cone completely conceals the ovaries with only stigmas emergent: *X.aethiopica*, *X.elliotii*, *X.hypolampra*, *X.monticola*, *X.odoratissima*, *X.phloiodora*, *X.wilwerthii*

Stigmas discrete, not connivent: *X.aurantiiodora*, *X.congolensis*, *X.lukei*, *X.mwasumbii*, *X.quintasii*, *X.tenuipetala*

Stigmas bearing stalked glands: *X.rubescens*

Monocarps sessile, ovoid, and splitting into three segments: *X.hypolampra*, *X.letestui*, *X.phloiodora*, *X.tanganyikensis*, *X.villosa*

Endocarp of dehisced monocarp green: *X.gracilipes*, *X.quintasii*, *X.shirensis*, *X.torrei*

Aril covers basal half of seed or less: *X.aethiopica*, *X.africana*, *X.mwasumbii*, *X.rubescens*, *X.staudtii*, *X.tenuipetala*

Aril completely covers the seed: *X.aurantiiodora*, *X.congolensis*, *X.quintasii*

### Geographic distribution of African species

Species lists for geographical areas in which *Xylopia* species occur. Species are listed in alphabetical order.

Senegal to Benin: *X.acutiflora*, *X.aethiopica*, *X.dinklagei*, *X.elliotii*, *X.letestui*, *X.longipetala*, *X.piratae*, *X.quintasii*, *X.rubescens*, *X.staudtii*, *X.villosa* (11)

Nigeria: *X.aethiopica*, *X.africana*, *X.calva*, *X.cupularis*, *X.katangensis*, *X.letestui*, *X.longipetala*, *X.monticola*, *X.phloiodora*, *X.pynaertii*, *X.quintasii*, *X.rubescens*, *X.staudtii*, *X.talbotii*, *X.thomsonii*, *X.villosa* (16)

Cameroon, Equatorial Guinea, São Tomé & Principe, Central African Republic: *X.aethiopica*, *X.africana*, *X.aurantiiodora*, *X.calva*, *X.cupularis*, *X.elliotii*, *X.gilbertii*, *X.hypolampra*, *X.katangensis*, *X.letestui*, *X.longipetala*, *X.mildbraedii*, *X.monticola*, *X.paniculata*, *X.phloiodora*, *X.pynaertii*, *X.quintasii*, *X.rubescens*, *X.staudtii*, *X.talbotii*, *X.thomsonii*, *X.villosa* (22)

Gabon and Republic of the Congo: *X.aethiopica*, *X.congolensis*, *X.cupularis*, *X.flamignii*, *X.gilbertii*, *X.globosa*, *X.hypolampra*, *X.katangensis*, *X.letestui*, *X.longipetala*, *X.mildbraedii*, *X.paniculata*, *X.phloiodora*, *X.pynaertii*, *X.quintasii*, *X.rubescens*, *X.staudtii*, *X.thomsonii*, *X.toussaintii*, *X.unguiculata*, *X.wilwerthii* (21)

Chad, South Sudan, Sudan, Uganda, Ethiopia: *X.aethiopica*, *X.nilotica*, *X.rubescens*, *X.thomsonii* (4)

Democratic Republic of the Congo: *X.aethiopica*, *X.aurantiiodora*, *X.congolensis*, *X.cupularis*, *X.flamignii*, *X.gilbertii*, *X.hypolampra*, *X.katangensis*, *X.letestui*, *X.longipetala*, *X.phloiodora*, *X.pynaertii*, *X.quintasii*, *X.rubescens*, *X.shirensis*, *X.staudtii*, *X.thomsonii*, *X.tomentosa*, *X.toussaintii*, *X.wilwerthii* (20)

Angola: *X.aethiopica*, *X.aurantiiodora*, *X.cupularis*, *X.flamignii*, *X.hypolampra*, *X.longipetala*, *X.odoratissima*, *X.paniculata*, *X.quintasii*, *X.rubescens*, *X.staudtii*, *X.thomsonii*, *X.tomentosa*, *X.toussaintii*, *X.wilwerthii* (15)

Eastern and Southern Africa: *X.aethiopica*, *X.arenaria*, *X.collina*, *X.gracilipes*, *X.holtzii*, *X.katangensis*, *X.keniensis*, *X.lukei*, *X.mwasumbii*, *X.odoratissima*, *X.rubescens*, *X.shirensis*, *X.tanganyikensis*, *X.tenuipetala*, *X.tomentosa*, *X.torrei* (16)

### I. Xylopia Section Neoxylopia

Taxon classificationPlantaeMagnolialesAnnonaceae

Engler & Diels, Monogr. afrik. Pflanzen-Fam. 6: 58. 1901.

758D871B-B0DC-5194-8B8F-D6E98D39B6A1

#### Type.

*Xylopiaafricana* (Bentham) Oliver (lectotype designated in Stull et al. 2017, p. 221).

#### Description.

Nodes with branches from 2–3 axillary buds; outer petals ovate or linear, if outer petals linear then over four times as long as the inner petals; inner petals ovate, lacking differentiated fleshy basal margins; anther connectives shieldlike at apex, overhanging the anther thecae, rarely with the center formed into a conical point; staminal cone rudimentary, surrounding only the bases of the ovaries, rim laciniate; carpels 3–15, the stigmas free to connivent, marked with warts, tubercles, or glandular appendages; aril brushlike; seed coat smooth, sarcotesta absent. Four species in Tropical Africa.

#### Notes.

The section was defined by Engler and Diels primarily on the basis of the distinctive aril. Three of the four species classified in this section are known to have such an aril. Fruits and seeds of the fourth species, *X.globosa*, are unknown, but the floral morphology of the species is strikingly similar to that of other species in the section, and molecular analysis (Stull et al. 2017) placed it as sister to *X.staudtii*. Stilt roots are another feature of species in this section: *Xylopiaafricana*, *X.rubescens*, and *X.staudtii* are regularly described as having such roots, but they have not been confirmed for *X.globosa*.

### 1. Xylopia africana

Taxon classificationPlantaeMagnolialesAnnonaceae

(Bentham) Oliver, Fl. trop. Afr. 1: 30. 1868.

3EA19EC5-15ED-5A33-8CD8-D11EF8B55EBB

Fig. 9B–F, J, K

Melodorum africanum Bentham, Trans. Linn. Soc. 23: 477. 1862.
Xylopicrum africanum (Bentham) Kuntze, Revis. gen. pl. 1: 8. 1891.
Fissistigma africanum (Bentham) Merrill, Philipp. J. Sci. 15: 130. 1919. Type. CAMEROON. Southwest Region, Camer[oon] Mount., Feb 1862, *G. Mann 1193* (lectotype, here designated: K! [000105591]; isolectotypes: GH—2 sheets! K! [000105592], P! [00169119], U! [0095511]).

#### Description.

***Tree*** up to 20 m tall, d.b.h. ca. 30 cm, stilt roots emerging from the trunk up to 2 m above base. ***Twigs*** brown, fine appressed-pubescent, the hairs 0.2–0.3 mm long; nodes occasionally with two axillary branches. ***Leaf*** with larger blades 6.3–15.4 cm long, 2.9–7.9 cm wide, subcoriaceous to coriaceous, discolorous, much paler abaxially *in vivo*, obovate, occasionally oblong or elliptic, apex short-acuminate to cuspidate, the acumen 2–10 mm long, base cuneate and decurrent on petiole, glabrous adaxially, fine appressed-pubescent abaxially; midrib plane to slightly impressed adaxially, raised abaxially, secondary veins weakly brochidodromous, 10–13 per side, diverging at 60–65° from the midrib, raised on both surfaces, higher-order veins forming a conspicuous reticulum that is slightly raised adaxially and strongly raised abaxially; petiole 6.4–9 mm long, semi-terete or canaliculate, appressed-pubescent to glabrate. ***Inflorescences*** axillary, 1–2-flowered, when 2-flowered the pedicels arising side by side from the axil, appressed-pubescent; pedicels 6.4–10.5 mm long, 0.7–1.6 mm thick; bracts 2, one to either side of midpoint, persistent, 1.2–3 mm long, broadly ovate to semicircular, apex obtuse to rounded, lower bract usually bifid from tearing down the center as the inflorescence enlarges; buds broadly ovoid, apex obtuse. ***Sepals*** erect or slightly spreading at anthesis, 1/10–1/5-connate, 4.5–7 mm long, 4.6–5.5 mm wide, coriaceous, ovate to triangular, apex acute, appressed-pubescent abaxially. ***Petals*** yellow to yellow-orange *in vivo*; outer petals more or less erect at anthesis, 8.2–9.5 mm long, 3.0–4.5 mm wide at base, 6.2–7.1 mm wide at midpoint, fleshy, ovate, apex acute to nearly rounded, concave in basal half adaxially, appressed-pubescent except for glabrous adaxial concavity; inner petals more or less erect at anthesis, 5.9–8.3 mm long, 1–2.6 mm wide at base, 3–3.9 mm wide at midpoint, chartaceous, narrowly rhombic to elliptic, apex acuminate or acute, base with undifferentiated margin, pubescent at apex, with finer pubescence extending into upper portion of concavity and base of concavity glabrous adaxially, pubescent at apex but otherwise glabrous abaxially. ***Stamens*** 100–120; fertile stamens 1.6–2.9 mm long, oblong to clavate, apex of connective 0.4–0.6 mm long, shieldlike, overhanging anther thecae, erect-pubescent, anthers 5–9-locellate, filament 0.5–1 mm long; outer staminodes 1.3–1.8 mm long, wedge-shaped to quadrate, apex obtuse to truncate; inner staminodes 2.8–3.4 mm long, clavate, apex rounded; staminal cone 1.8–2.7 mm in diameter, 0.6–0.8 mm high, concealing only the bases of the ovaries, rim laciniate. ***Carpels*** 9–15; ovaries 1.6–2.1 mm long, linear-oblong, pubescent, stigmas connivent, 3.2–4.6 mm long, linear, verrucose toward base, glabrous. ***Torus*** flat, 3.4–4 mm in diameter. ***Fruit*** of up to 10 glabrate monocarps borne on a pedicel 10–18 mm long, 4–5 mm thick, glabrate; torus 8–16 mm in diameter, 5–8 mm high, depressed-globose. ***Monocarps*** reddish to purplish green with red endocarp *in vivo*, 6.6–10.5 cm long, 1.0–1.4 cm wide, ca. 1.1 cm thick, linear-oblong to cylindrical, occasionally slightly falciform, torulose, apex mucronate, the beak 1–1.5 mm long, base contracted into a stipe 10–18 mm long, 2.5–5 mm thick, verrucose and longitudinally wrinkled; pericarp ca. 0.8 mm thick. ***Seeds*** in a single row, lying parallel to long axis of monocarp, up to 5 per monocarp, 13–15 mm long, 9–10.5 mm wide, 8.5–9 mm thick, ellipsoid, broadly elliptic in cross-section, truncate at micropylar end, rounded at chalazal end, black, smooth, shiny, raphe/antiraphe plane, micropylar scar ca. 1.5 mm in diameter, circular; sarcotesta absent; aril blood-red *in vivo*, dull orange-brown when dried, brushlike, 8–10 mm in diameter, ca. 4 mm high, fleshy, granular.

**Figure 9.**
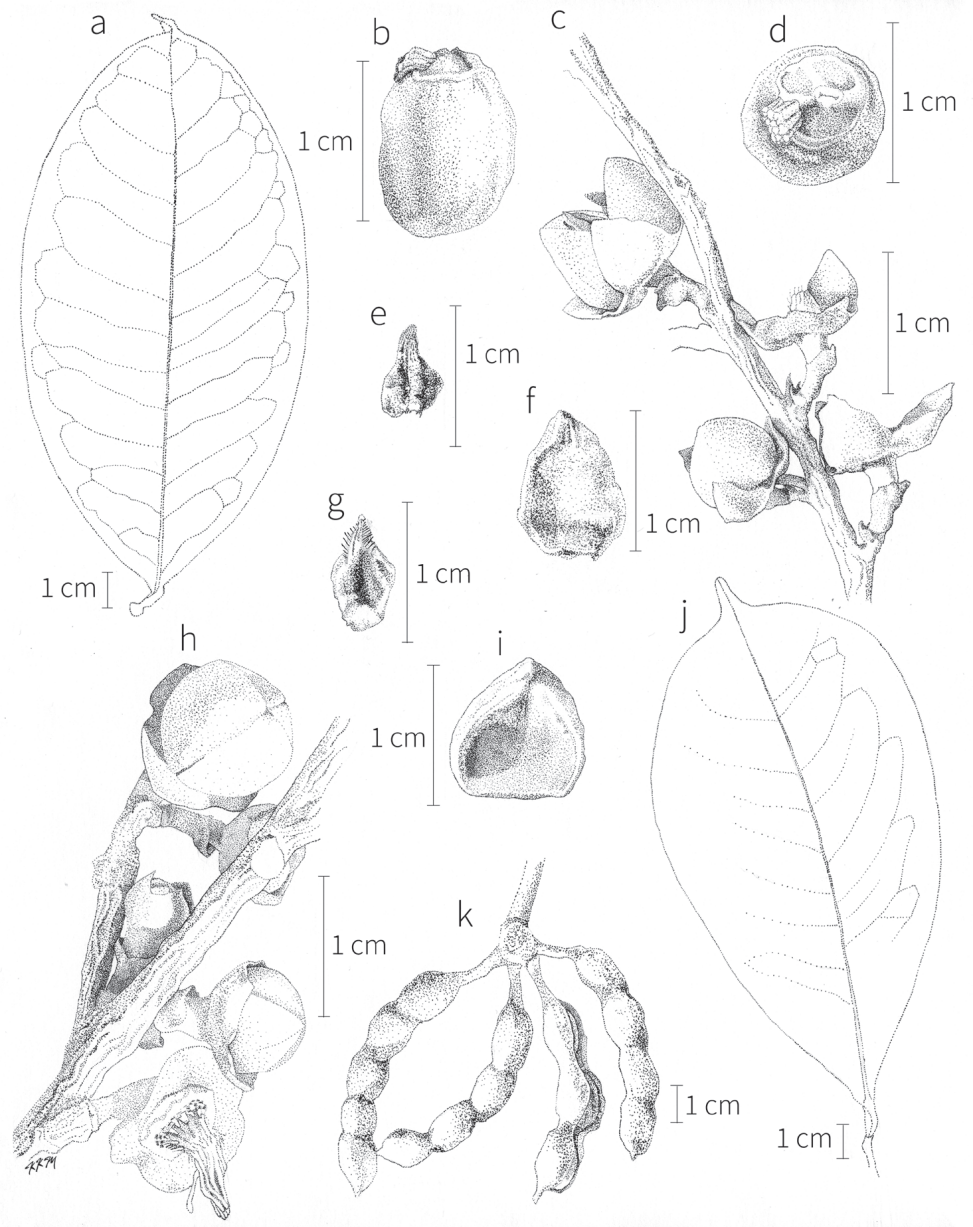
*Xylopiaglobosa* and *X.africana*. **A, G–I***X.globosa***A** Leaf **G** Inner petal, abaxial view, showing keel **H** Inflorescence, showing four buds and one flower with petals fallen **I** Outer petal, adaxial view **B–F, J, K***X.africana***B** Seed, lateral view with most of aril appendages fallen **C** Four inflorescences **D** Seed, micropylar end view **E** Inner petal, abaxial view **F** Outer petal, adaxial view **J** Leaf **K** Fruit. **A, G–I** from *Normand s. n.* (P), **B, D, E, F, J, K** from *Letouzey 14551* (WAG) **C** from Thomas *4554* (MO).

#### Phenology.

Specimens with flowers have been collected October–March and in May, with buds in August and November, and with fruits in September–January, March, and May.

#### Distribution

**Figure 10.**
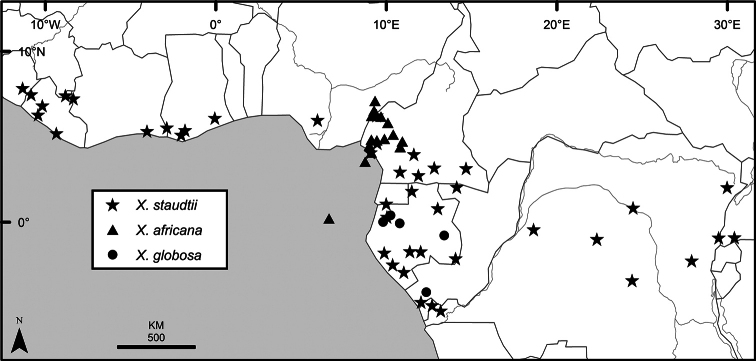
Distributions of *Xylopiaafricana*, *X.globosa*, and *X.staudtii*. Bolder lines represent country borders, fainter lines lakes and major rivers.

(Fig. 10). Occurs in montane and submontane mossy forests at elevations of 900–2000 m in southeastern Nigeria, southwestern Cameroon, and on the islands of Bioko (Equatorial Guinea) and São Tomé (São Tomé & Principe).

#### Local names.

No local names were reported for this species on specimen labels, but Focho et al. (2010) listed the name “Hweneta (Ghana)” as applied to this species in the Mt. Cameroon area of Cameroon; the name “hwentea” is used widely in Ghana for *Xylopiaaethiopica*.

#### Additional specimens examined.

**NIGERIA.** Cross River: Obudu District, Obudu Plateau, 18 Mar 1964 (fl), *Hopkins FHI 54307* (WAG); Ogoja Province, Sonkwala area of Obudu Division, grass plateau above Ikwette, 5200 ft, 28 Dec 1948 (fl, fr), *Savory & Keay FHI 25179* (K); Northern Ranges, Obudu Ranch, SE State, ca. 5200’, 4 Jan 1973 (fl), *Lock GC43569* (K); Boshi Extension Forest Reserve, 6°20'N, 9°20'E, alt. ca. 1600 m, 23 May 1971 (fl, fr), *van Meer 1768* (WAG—2 sheets). **CAMEROON.** North: Chaîne de Nkohom à 42 km SSW de Ndiki, 14 Nov 1983 (buds, fr), *Nkongmeneck 580* (P).—Northwest: Gazette Bali Ngemba F. R., 5°48.02'N, 10°05.78'E, 1700 m, 14 Nov 2000 (buds, fr), *Cheek 10527* (K, MO); West Division, Gazette Bali Ngemba F. R., 5°49'N, 10°05'E, 1600 m, Mantum, 5 Oct 2001 (fr), *Onana 1825* (K), *Onana 1835* (K); Bali Ngemba F. R., 5°49.59'N, 10°05.57'E, 1700 m, 9 Nov 2000 (bud), *Tadjouteu 410* (K).—Southwest: Buea, 1906 (fl, fr), *Deistel 154* (A, BM, P); without definite locality, s. d. (fr), *Deistel 454* (GH); Buea, 1000 m, *Lehmbach 41* (B); Buea, *Lehmbach 137a* (B, M); Monts Rumpi-Rata Mount, 1788 m, 2 km au SW de Dikome Balua, 35 km NNW Kumba, 24 Mar 1976 (fl, fr), *Letouzey 14551* (K—2 sheets, MO, P, WAG); Buea, 3000 ft., *Maitland 233* (K, PRC); Cameroon Mountain, Buea area, 3–4000’, 1930 (fl, fr), *Maitland s. n.* (K); Mt. Cameroun, NW de Buèa, 13 Mar 1981 (st), *Meijer 15378* (DSM, K, MO); southern slope of Mount Cameroon above Batoke, 4°08'N, 9°05'E, 900-2000 m, 9–20 Jan 1984 (fl), *Thomas 2981* (K, MO); forest in the Rumpi Hills, near Dikome Balue, 4°53'N, 9°53'E, Mar 1984 (fl), *Thomas 3305* (K, MO, P, WAG); savanna with forest galleries near Aguosho, 10 km SSW of Akwaya, 6°18'N, 9°28'E, 1200 m, 19-20 Mar 1985 (fl), *Thomas 4554* (MO, P); Limbe District, Fako Division, Mt. Etinde, N face of N ridge, ca. 1220 m, 24 Oct 1992 (fl, fr), *Wheatley 605* (P).—West: Route Batcha-Batschingou (22 km ESE Bafang), 23 Nov 1974 (fl, fr), *Letouzey 13300* (K, MO–2 sheets, P, WAG); Region M’Bamileke [“M’Bamileleke”], 19 Dec 1957 (fr), *de Wit 7947* (WAG—3 sheets). **EQUATORIAL GUINEA.** Bioko: entre Moca y el cruce Luba—Riaba, 1400 m, (st), *Fernández Casas 11693* (K); entre Moca y Riaba por el camino viejo, 1280 m, 20 Feb 1989 (fl, fr), *Fernández Casas 11823* (K); entre Moca y el lago Loreto, 1510 m, 21 Feb 1989 (fl, fr), *Fernández Casas 11884* (K); Finca Puente, carretera de Usola a Moná, km 17, 20 Jan 1947 (fr), *Guinea 1658* (MO); Biao Peak Trail, Pt 128-Pt 130, 3.36272°S, 8.65264°E, 1500 m, 13 Mar 2007 (fl, fr), *Luke et al. 11858* (K); Bioko Sul, Balacha North Trail, 3.3797°N, 8.6669°E, 1300 m, 28 Sep 2009 (fr), *Luke 13606* (MO). **SAO TOME & PRINCIPE.** Vila José, NW of Ribeira Peixe, track between Vila José and Cão Grande, 150–250 m, 21 Jan 1980 (fl, fr), *de Wilde et al. 220* (P, WAG—2 sheets); Island of St. Thomas, rec. Nov 1861 (fr), *Mann s. n.* (K-000199061, K-000199062, K-000199063, P-00169118 as *Mann 1193*); Ins. S. Thome in sinu Biafra, ad Fazenda do Monte Coffé, 1860 (fl, yg fr in drawing), *Welwitsch 764* (BM).

*Xylopiaafricana* is most similar to *Xylopiastaudtii*. It is a smaller tree that can be distinguished by its larger sepals, obtuse outer petal apices, and red arils on the seeds. Keay (1954–1958, 1989) indicated that the leaf veins of *X.africana* are dark crimson on the underside of the leaves, but this has not been reported by other collectors and we did not observe this characteristic in dried specimens. The label of *Cheek 10527* described the fresh leaves as “nearly white below” and the leaves of dried specimens usually have a uniform tan color abaxially. *Xylopiaafricana* also resembles *X.globosa*, but has smaller obovate leaves, obtuse ovate outer petals, and lacks a keel on the abaxial surface of the inner petals.

*Xylopiaafricana* occupies a unique habitat among African *Xylopia* species, occurring in mossy submontane to lower montane forest, reaching elevations of 2000 m. Bryophytes attached to the branches of several collections of *X.africana* (particularly conspicuous on the specimen *Nkongmeneck 580*) suggest the high humidity of the forests in which it grows. Associated species at one site in Cameroon included *Carapagrandiflora*, *Garcinia* spp., *Psychotria* spp., and *Syzygiumstaudtii* (*Letouzey 14551*). Soils in the forests in which the plants grow are acidic and sandy, with variable mineral nutrient composition (Fonge et al. 2013). *Xylopiaafricana* is a canopy tree confined to these forests and is one of many endemic plant species threatened by continued clearing of the forests for agriculture (Onana and Cheek 2011, Fonge et al. 2013). It is one of the few *Xylopia* species with a distribution extending to islands in the Gulf of Guinea, where it occurs on Bioko and São Tomé. On the former, it is found at elevations above 1200 m, while the collection *de Wilde et al. 220* from São Tomé gives an elevation of only 150–250 m and a habitat of “old secondary forest with remnants of primary forest.”

The collection *de Wit 7947* appears to be a mixed collection. Leaves in packets and some of the monocarps are *X.africana*, but some detached monocarps in packets have much smaller seeds oriented obliquely to the long axis of the monocarp and may be those of *X.thomsonii*, which was collected by de Wit as *de Wit Herb. 7952* (WAG).

Bentham gave the type information as from “Island of S. Thomas off the West Coast, and Cameroon Mountain, at 4000 feet (*G. Mann*).” The specimen *Mann 1193* at K (ex Herb. Hook., K-000105591) from the “Cameroon Mountain 4000 ft” locality, collected in Feb. 1862, has a flower and relatively small leaves, as well as sketches, presumably by Bentham, of stamens, carpels, and petals mounted on the sheet. This specimen is chosen as the lectotype. A second sheet at K (K-000105592), stamped Herb. Hook., is also numbered *Mann 1193* from the same location and date, has flowers and relatively small leaves and is considered an isolectotype. The other sheets at Kew collected by Mann (K-000199061, K-000199062, and K-000199063) all labeled as having come from “St. Thomas,” have no collection number, have leaves that are larger than those of the lectotype specimens, and have fruits.

There are two sheets at P. A sheet numbered 1193 and labeled Cameroon Mt, *Mann*, 1862, has fruits and larger leaves. A second sheet has a specimen with flowers and smaller leaves and a printed ticket giving the S. Thomas locality but no collection number. The two labels seem to have become reversed in the distribution of the duplicates. We consider the lectotype material to be the specimens with smaller leaves and flowers whatever collection number or locality may be indicated on the sheet, as they all seem to represent the same gathering.

### 2. Xylopia globosa

Taxon classificationPlantaeMagnolialesAnnonaceae

D. M. Johnson & N. A. Murray sp. nov.

8B493076-EE81-5B1C-8F62-D4D8EA72C2E7

urn:lsid:ipni.org:names:60476241-2

Figs 3A , 9A, G–I

#### Diagnosis.

Species resembling *Xylopiaafricana* in its short broad flowers with outer petals thicker and broader than the inner petals, but differing in the glabrous twigs and leaves, the leaf blades consistently larger (15.7–23.5 cm long, 8.3–11.7 cm wide), the smaller and wider sepals, the more rounded apices of the outer petals, and the conspicuously keeled inner petals.

Type: GABON. Ogooué-Lolo, Chantier Bambidie (CEB), ca. 70 km E of Lastoursville, 57 km on CEB road to Okondja, 0°46.6'S, 13°23.6'E, 325 m, 1 Nov 2005, *M. S. M. Sosef et al. 2180* (holotype: WAG! [1540447]; isotypes: OWU—2 sheets! WAG! [1540446, 1540448, 1540449]).

#### Description.

***Small tree or shrub*** up to 8 m tall, d.b.h. up to 25 cm, bole cylindrical, knee roots [ex *Sosef et al. 2180*] on lower 0.5 m of trunk; bark light gray, smooth. ***Twigs*** light brown to gray-brown, eventually reddish brown, glabrous or with a few weak hairs ca. 0.4 mm long and then soon glabrate; nodes with axillary branches not seen. ***Leaf*** with larger blades 15.7–23.5 cm long, 8.3–11.7 cm wide, subcoriaceous, paler or discolorous abaxially, oblong to oblong-elliptic or oblong-oblanceolate, apex cuspidate, the cusp 3.5–9 mm long, or rarely emarginate, base rounded to broadly cuneate and decurrent on petiole, glabrous on both surfaces; midrib slightly impressed adaxially, raised abaxially, secondary veins weakly brochidodromous, 10–14 per side, diverging at 65–75° from the midrib, slightly raised on both surfaces, higher-order veins indistinct or slightly raised adaxially, plane to slightly raised abaxially, usually forming a conspicuous reticulum on both surfaces; petiole 9–26 mm long, canaliculate adaxially, glabrous. ***Inflorescences*** axillary, 1–2-flowered, finely appressed-pubescent; peduncle 1 per axil, 3.5–5.2 mm long; pedicels 1–2 per peduncle, 6.7–10.5 mm long, 2.6-2.9 mm thick; bracts 3, evenly spaced on pedicel, persistent, 2.4–4 mm long, broadly ovate to semicircular, apex rounded; buds depressed-globose, apex rounded. ***Sepals*** spreading at anthesis, 1/3–1/2-connate, 4.8–6 mm long, 7–10 mm wide, coriaceous, crescent-shaped to nearly semicircular, apex rounded or obtuse, sparsely pubescent to glabrate abaxially. ***Petals*** yellow *in vivo*; outer petals slightly spreading at anthesis, 9–13.4 mm long, 10–10.5 mm wide at base, 8.7–9.3 mm wide at midpoint, fleshy, broadly ovate, apex obtuse to rounded, finely pubescent on both surfaces except for the glabrous adaxial concavity; inner petals slightly spreading at anthesis, 7.8–9.3 mm long, 1.5–1.6 mm wide at base, 3.5–4.7 mm wide at midpoint, chartaceous to coriaceous, narrowly rhombic, deeply concave, strongly keeled abaxially, apex acuminate, base with undifferentiated margin, pubescent at apex on both surfaces but otherwise glabrous and somewhat verrucose. ***Stamens*** ca. 100; fertile stamens 1.7–2.3 mm long, narrowly oblong, apex of connective 0.5–0.8 mm long, shieldlike, overhanging anther thecae, puberulent to glabrate, anthers 8–11-locellate, filament 0.3–0.4 mm long; outer staminodes 1.6–1.8 mm long, wedge-shaped to quadrate, apex truncate; inner staminodes 1.7–2.6 mm long, clavate or narrowly oblong, apex rounded; staminal cone 6–7 mm in diameter, 1.3–1.9 mm high, concealing only the bases of the ovaries, rim laciniate. ***Carpels*** ca. 15; ovaries 1.8–2.5 mm long, narrowly oblong, densely pubescent, stigmas loosely connivent, 5–5.7 mm long, linear, studded on sides with round tubercles. ***Torus*** flat, 6–7.5 mm in diameter. ***Fruit*** and ***seeds*** unknown.

#### Phenology.

Specimens with flowers were gathered in September and November.

#### Distribution

(Fig. 10). Known from four scattered localities in Gabon and from one locality in the southern Republic of the Congo; secondary forest and forest edge; 80–325 m.

#### Local name.

Ntsua (*Normand s. n.*).

#### Additional specimens examined.

**GABON.** Estuaire: Cristal Mountains, 9 km S of Kinguélé, ca. 0°24'N, 10°15'E, 80 m, 21 Sep 1994 (st), *Breteler et al. 12978* (WAG); Estuaire du Gabon, sud de Chinchoua [0.0149°N, 9.8094°E], 16 Sep 1959 (fl), *Normand s. n.* (P).—Moyen-Ogooué: about 10 km NNW of Ndjolé, 0°04'S, 10°47'E, 150 m, 25 Sep 1994 (st), *Breteler et al. 13000* (MO, WAG). **REPUBLIC OF THE CONGO.** Environs de Dimonika, forêt vieille secondaire entre tour meteo et piste Kuilila–Makaba, 14 Dec 1982 (st), *Cusset 1200* (P-01963323).

The flowers of *Xylopiaglobosa* are large, fleshy, and spherical, and scarcely resemble those of a *Xylopia*. In addition, the leaves of this new species are the largest of any African *Xylopia*, oblong to elliptic in shape, and usually sharply cuspidate at the apex, and allow confident identification of sterile specimens. The species is most similar to *X.africana* from which it differs in several additional floral features: the sepals are wider than long and crescent-shaped to semicircular, the outer petals are rounded at the apex, and the inner petals have a crestlike keel on the abaxial surface.

Fruits and seeds of *Xylopiaglobosa* are not yet known. A specimen from Equatorial Guinea (Litoral: Monte Alen National Park, Monte Mitra, 01°21'46"N, 9°57'40"E, 1000 m, 30 Sep 2005 (fr), *Leal et al. 817* (MO)) is possibly a collection of this species with fruits. This specimen was taken from a tree 10 m tall, the fruit described as having black seeds with red arils. The plant was growing on a swampy mountain summit at 1000 m in elevation. The specimen was initially identified as *X.staudtii*, but its leaf blades are thinner, larger, and more rounded at the bases than is typical for that species, with a sharply short-acuminate to cuspidate apex. *Xylopiastaudtii* typically has yellow to orange arils, and fruits on a tree 10 m in height would also be unusual. The leaves of this specimen are more similar in shape to those of *X.globosa* than to those of *X.staudtii*, but are smaller (largest 12.4 cm long, 5.8 mm wide, with a petiole 5 mm long and acumen 9 mm long) and chartaceous. There are no seeds present on the specimen, only two detached and dehisced monocarps.

### 3. Xylopia rubescens

Taxon classificationPlantaeMagnolialesAnnonaceae

Oliver, Fl. trop. Afr. 1: 30. 1868.

F2D4EF76-811A-5251-8DB5-2E9C7F7A4E64

Fig. 11

Xylopicrum rubescens (Oliver) Kuntze, Revis. gen. pl. 1: 8. 1891. Type. NIGERIA [“Upper Guinea”]. Rivers, Old Calabar, s. d., *W. C. Thomson 53* (holotype: K! [000199073]).
Xylopia klaineana Pierre ex Engler & Diels, Monogr. afrik. Pflanzen-Fam. 6: 59–60. 1901.
Xylopia rubescens Oliver var. klaineana (Engler & Diels) Pellegrin, Bull. Soc. Bot. France, Mém. 31: 70. 1949. Type. GABON. Without definite locality, Oct 1898, *T.-J. Klaine 1327* (holotype: P! [00169139]; isotypes: B! [100154150], P! [00169138]).
Xylopia humilis Engler & Diels, Monogr. afrik. Pflanzen-Fam. 6: 60 + t. 21B, a–f. 1901. Type. LIBERIA [“Oberguinea”]. Grand Bassa County, Fishtown bei Granbassa, 27 Aug 1898, *M. Dinklage 2006* (lectotype, here designated: B! [10 0154147]; isotypes: A! [00061927, 00062417], B! [100154145, 100154146, 1001541480], K! [000199074, 000199075, 000199076]).
Xylopia batesii Engler & Diels, Monogr. afrik. Pflanzen-Fam. 6: 62. 1901. Type. CAMEROON or GABON. Angom, 70 engl. Meilen östlich von Gabun, Oct 1896 [29 Oct 1897 on P sheet], *G. L. Bates 561* (holotype: K! [000199058]; isotypes: BM! [000510769], G! [00190711], P! [00169131, 00169132]).
Xylopia butayei De Wildeman, Ann. Mus. Congo, Sér. 4, Bot. 1: 33. 1902. Type. DEMOCRATIC REPUBLIC OF THE CONGO. Kongo Central, Malela (Bas-Congo), *R. P. Butaye, coll. J. Gillet 2239* (holotype: BR!; isotype: BR! [0000008825391]).
Xylopia zenkeri Engler & Diels, Bot. Jahrb. Syst. 39: 480. 1907. Type. CAMEROON. South Province, bei Bipindi unweit Mimfia im Urwald, Mar 1904, *G. A. Zenker 2827* (holotype B! [100154149]; isotypes: BM! [000511041], G! [00190712, 00190713], GOET! [005735], HBG! [502474], K! [000199060], L! [0196246], M! [0107919], P! S! [07-13458], WAG! [0065882], WU! [0025792]).
Xylopia gossweileri Exell, J. Bot. 64: Suppl. 6. 1926. Type. ANGOLA [“Portuguese Congo”]. Cabinda Province, Cabinda, Pango Munga, Mayumbe, s. d., *J. Gossweiler 6222* (holotype: BM! [000511046], photos at GH, MO, NY).

#### Description.

***Tree*** up to 25 m tall, d.b.h. up to 70 cm, rarely described as a shrub or liana, bole straight, cylindrical, usually with stilt roots arising from the trunk up to a height of 2 m, short secondary branches forming a narrow crown; bark white, pale yellowish brown, or light gray, smooth. ***Twigs*** brown to gray, eventually whitish gray, initially appressed golden-pubescent, the hairs 0.2–0.5 mm long, but soon glabrate; nodes occasionally with two axillary branches. ***Leaf*** with larger blades 7.3–21.3 cm long, 3.6–8.4 cm wide, subcoriaceous, occasionally chartaceous, strongly discolorous, olive-green adaxially, rusty or orange-brown abaxially, rarely concolorous, oblong, elliptic, or oblanceolate, apex short-acuminate or cuspidate, the acumen or cusp 2–15 mm long, occasionally acute or rounded, base cuneate to occasionally rounded and decurrent on petiole, glabrous adaxially, finely appressed-pubescent abaxially, rarely glabrous; midrib impressed to plane adaxially, raised and usually formed into a sharp keel distal to the midpoint abaxially, secondary veins weakly brochidodromous, 8–16 per side, diverging at 45–70° from the midrib, slightly raised adaxially and abaxially, higher-order veins forming a conspicuous reticulum that is distinctly raised on both surfaces, rarely indistinct; petiole 5.4–12 mm long, canaliculate, sparsely appressed-pubescent to glabrous. ***Inflorescences*** axillary, usually from the axils of fallen leaves, 1–3 flowered, pubescent; peduncles 1 per axil, 1.5–2.5 mm long; pedicels 1–3 per peduncle, 3.5–8.5 mm long, 1–1.3 mm thick; bracts (2) 3–5, evenly spaced up to 1/2–2/3 distance from base, persistent, uppermost 1.1–2.9 mm long, ovate, apex obtuse; buds linear-subulate to linear-lanceolate, occasionally falciform, apex acute, occasionally uncinate. ***Sepals*** spreading at anthesis, nearly free to 1/3-connate, 2.2–4.2 mm long, 3.0–3.5 mm wide, coriaceous, ovate to broadly triangular, apex acute, sericeous abaxially. ***Petals*** yellow, yellow-orange, or caramel-colored, the inner petals red except for the cream to yellow base and apex *in vivo*; outer petals erect or slightly spreading at anthesis, 17.6–35 mm long, 2.5–5.1 mm wide at base, 1.3–3.4 mm wide at midpoint, fleshy, linear, apex acute, appressed-pubescent adaxially but with a glabrous patch at base, appressed-pubescent abaxially; inner petals erect at anthesis, 3.5–6.7 mm long, 2.2–4.6 mm wide, fleshy, rhombic to ovate, apex acuminate, the acumen 1.4–3.1 mm long, base with undifferentiated margin, pubescent on acumen and glabrous toward base adaxially, keeled and pubescent in distal half to either side of the keel abaxially. ***Stamens*** 70–77; fertile stamens 1.6–2.2 mm long, quadrate, oblong, or clavate, apex of connective 0.4–0.7 mm long, shieldlike, overhanging anther thecae, puberulent or papillate, anthers 6–8-locellate, filament 0.3–0.9 mm long; outer staminodes 1.2–2.5 mm long, oblong-pentagonal, oblong, or ovate, apex obtuse; inner staminodes 1.1–1.3 mm long, narrowly oblong, apex obtuse to truncate; staminal cone 2.1–2.4 mm in diameter, 0.3–0.8 mm high, concealing only the bases of the ovaries, rim laciniate. ***Carpels*** (4–) 7–12; ovaries 1.0–2.1 mm long, narrowly oblong, golden-tomentose, stigmas loosely connivent, 1.4–2.9 mm long, linear, bearing glandular appendages. ***Torus*** flat, 2.3–3.0 mm in diameter. ***Fruit*** of up to 15 glabrate monocarps borne on a pedicel 9–15 mm long, 2.2–7 mm thick, glabrate; torus 6–14 mm in diameter, 5–10 mm high, subglobose to globose. ***Monocarps*** with black or dark purple exterior and pink to scarlet endocarp *in vivo*, 4.1–16.3 cm long, 0.6–1.2 cm wide, 0.6–1.1 cm thick, narrowly oblong, torulose to moniliform, somewhat falcate, apex rounded to a distinct beak up to 5 mm long, base contracted into a stipe 4–20 mm long, 2–6 mm thick, obliquely wrinkled, finely verrucose; pericarp 0.6–1.0 (2.0) mm thick. ***Seeds*** 1–7 per monocarp, in a single row, lying parallel to long axis, 9.5–19.8 mm long, 5.5–10.7 mm wide, 5.5–9.8 mm thick, oblong-ellipsoid, circular in cross-section, truncate at micropylar end, rounded at chalazal end, reddish brown to brownish black, smooth or slightly wrinkled, shiny, raphe/antriraphe raised, less distinct around chalazal end, micropylar scar 1.5–3.5 mm long, 1.5–2.5 mm wide, ovate; sarcotesta absent; aril orange to red *in vivo*, light brown when dried, brushlike, 5.5–9 mm in diameter, 3.9–4 mm high, fleshy, granular.

#### Phenology.

Specimens with flowers have been collected from all months of the year except December, although flowering appears more limited for specimens from the northeastern (February, May, and July) and southeastern (June, August–November) areas of the distribution, perhaps in relation to the more restricted seasonal rainfall patterns of these areas compared to the coast. Similarly, collections have been gathered from the coastal localities with fruits in February, May–June, August, and October–November, but only from November in the northeast, and May and August–October in the southeast.

**Figure 11.**
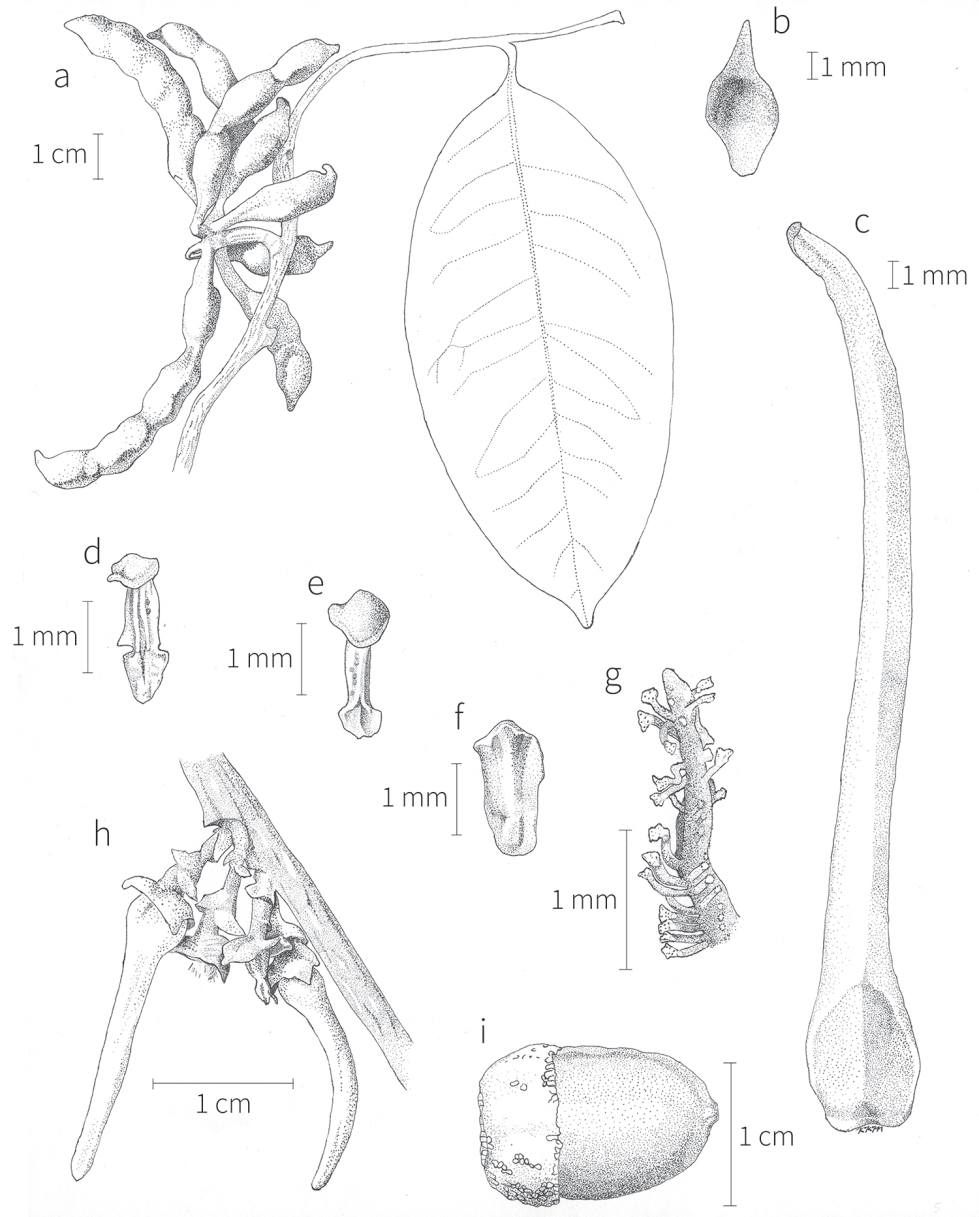
*Xylopiarubescens*. **A** Habit with fruit **B** Inner petal, adaxial surface **C** Outer petal, adaxial surface **D, E** Stamens, abaxial view **F** Staminode, abaxial view **G** Stigma apex **H** Inflorescence **I** Seed, longitudinal view. **A** from *Brenan et al. 8022* (K), **B–H** from *Angus 870* (MO) **I** from *Breteler et al. 10446* (WAG, spirit collection).

#### Distribution

**Figure 12.**
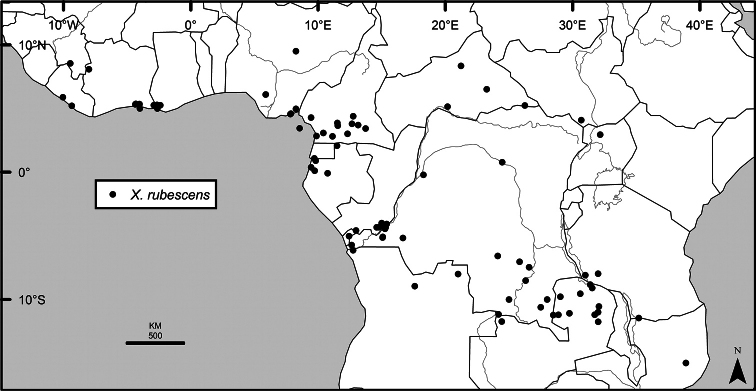
Distribution of *Xylopiarubescens*. Bolder lines represent country borders, fainter lines lakes and major rivers.

(Fig. 12). Occurs from Guinea and Liberia in West Africa east to South Sudan and south to Angola, Zambia, and Mozambique at elevations from sea level up to 1690 m. This species occurs in a range of wetland habitats, including gallery and other riparian forests, swamp forest, *Raphia* swamps, and pond edges.

#### Local names.

Fondé de marais (*Aubréville 1511*), fondé des rivières, fula-bifum (*Bates 561*), iyere (Kakwa, *Myers 13598*), mabama (*Gossweiler 8957, Gossweiler s. n.*), majindi (Banda, *Tisserant 1885*), mbowobowo (Kimatengo, *Ruffo & Kisena 3235*), mtua (Pahouin du Gabon, *Fleury 33135*), mut (*Gossweiler 8747*), muyombo (Kiluba de Kabongo, *Schmitz 5771*), mwengele (Wemba, *Brenan & Greenway 8022*, Kiswahili or Kirungu, *Hopper 1*), nitumbo (*Gossweiler 8747*), ntom (Ntomou, Focho et al. 2010), ntua (Pahouin de Gabon, *Fleury 33135*), odjobi (Ntoumou, Focho et al. 2010; Yaoundé-boulou, *Letouzey 1611*), odjobi nzam (Cameroon, Focho et al. 2010), odzobé (Yaoundé, *Fleury 33135*), odžǖ’ē (Tessmann 1913), ôjobi (*Bates 1317*).

#### Additional specimens examined.

**GUINEA.** Macenta, Soulakoto, 10 Aug 1949 (st), *Adam 5910* (MO, P); Macenta, Tènèmadou 10 Aug 1949 (st), *Adam 5911* (MO, P); Macenta+Beyla Prefectures, Simandou Range, 8°33'41"N, 8°53'06"W, 868 m, 23 Mar 2008 (fl buds), *Tchinegue 3113* (K). **LIBERIA.** Troh, Sangwin River drainage, 6 May 1948 (fr), *Mayer 150* (US). **IVORY COAST.** Route de Dabou, Marais de l’Agnéby, 27 Nov 1968 (fl), *Aké Assi 10378* (MO); Banco, *Aubréville 1511* (BR, P); Assinie dans le Sanvi, *Chevalier 16321 bis* (P); Abidjan, Banco Forest Reserve, 9 Oct 1974 (fr), *de Koning 4070* (WAG—2 sheets), 2 Feb 1975 (fl, fr), *de Koning 5296* (WAG), 22 Feb 1976 (fl), *6615* (MO, WAG—2 sheets); Abidjan, 3 km N of Armébé, about 10 km NE of Dabou, c. 5°22'N, 4°19'W, 15 May 1979 (fr on label), *de Kruif E 20* (WAG—2 sheets); Abidjan, along Agnéby R., not far from Armébé, about 10 km W of Dabou, 5°21'N, 4°20'W, 21 May 1979 (yg fr), *de Kruif 57* (WAG). **GHANA.**Ankasa Forest Reserve, Mpatabo-Elubo Road, *Enti 890* (MO); Atuabo Road near Chrisan, 24 Oct 1973 (st), *Enti & Awriah R1136* (K, MO). **NIGERIA.** Prov. Calabar, Eket District, Stubbs Creek Forest Reserve, 3 May 1951 (fr), *Amachi FHI 24311* (K); “Southern Nigeria,” without specific locality s. d. (fl), *Kennedy 1960* (A, B, BM, K); Jamieson River, Sapoba, 1935 (fl), *Kennedy 2752* (BM); without definite locality, *van Meer 985* (WAG); S. Nigeria, *Thomewill s. n.* (K); Benue Plateau, Jos District, 30 km S of Jos, 9°45'N, 8°50'E, 3 Feb 1972 (fr), *Wit & Wit FHI 64927* (K, WAG—2 sheets). **CAMEROON.** Bitye, Yaunde, 1919 (fl), *Bates 1317* (BM, MO); environs de Duala, Jun 1917 (fl, fr), *Fleury 33135* (P); Vuneli, 1 Feb 1928 (fl), *Hedin 1668* (OWU, P); marecage du Niagoul entre Koumbou et Miambo, 14 Apr 1959 (fl, fr), *Letouzey 1611* (K—3 sheets, P); près Akok Bikele, 3 Mar 1962 (fl), *Letouzey 4464* (K, P); station du Cacaouer de N’Koemvone, 14 km on the road from Ebolowa to Ambam, 2°49'N, 11°08'E, 11 Apr 1975 (fl), *de Wilde 8166* (B, BR, K, MO, P, WAG); Bipindi, Mimfia Bergen, Jan 1903 (fl), *Zenker s. n.* (F, NY). **CENTRAL AFRICAN REPUBLIC.** Oubangui, reg. de Yango, Mar 1920 (fl), *Allouette s. n.* (L); Manovo-Gounda-St. Floris National Park, 9.3 km S of Koumbala Pende confluence on Pende Creek, 8°21'N, 21°14'E, 610 m 15 May 1984 (fl), *Fay 6639* (MO, P); Yalinga, 15 Jul 1922 (fl), *Le Testu 3998* (BM, P); Waka, bassi[n?] riv. Dangava, 10 May 1925 (fl), *Tisserant 1885* (BM, BR, P). **SOUTH SUDAN.** Equatoria, Yei, Libogo, Khor Ini, 19 Nov 1940 (st), *Myers 13586* (K); Libogo, Yei, 27 Nov 1940 (fr), *Myers 13598* (K). **EQUATORIAL GUINEA.** Bioko: Malabo—Luba, Estrada km 46, 3 Nov 1986 (fl, fr), *Carvalho 2646* (B, BM, F, FI-T, K—2 sheets, MO, NY, WAG).—Río Muni: Nkolentangan [bei Alén, Tessmann 1913], Span. Guinea-Nintod, Engong, 450 m, 24 Feb 1908 (fl), *Tessmann 160* (K). **GABON.** 5–15 km NW of Ndjolé, Missanga, 0°05'S, 10°45'E, 13 Nov 1991 (fr), *Breteler & Jongkind 10446* (WAG—2 sheets); Uboro sur le Ramboué, *Chevalier 27105* (P); environs de Libreville, Nov 1898 (fl), *Klaine 186* (OWU, P); Bitam, region entre Ogooué et Cameroun, 9 Mar 1933 (fl), *Le Testu 9019* (BM, BR, OWU, P); Obour, region entre Ogooué et Cameroun, 1 May 1933 (fl), *Le Testu 9095* (BM, P). **REPUBLIC OF THE CONGO.** NE de Brazzaville, route des falaises de Douvres, village de Gamakala, mare à Sphaignes, 22 Jun 1960 (fl), *Descoings 5904* (P); Marais du Djili, près de Brazzaville (M. Congo), *Koechlin 5299* (P); champ de ter de Lifuola, mare de Gawa Kala, 12 Aug 1966 (fl), *Lissouba 22* (P); Tourbière de Ngamakala, 24 Feb 1973 (fl), *Makany 882* (K); Plateau des Cataractes, région de Boko, 4 Aug 1963 (fr), *de Nere 404* (P); Route de Brazzaville, forêts du Mayumbe, Jan 1891 (fl), *Thollon 4026* (P). **DEMOCRATIC REPUBLIC OF THE CONGO.** Équateur: Bamania [next to Mbandaka], 1930 or 1934 (fl), *Lebrun 1246* (A, B, K).—Haut-Katanga: Left-hand side of Luapula R. to Fort Rosebery, 5 Oct 1947 (fl, fr), *Brenan & Greenway 8022* (BM, EA, K—2 sheets, NY, P); a 6.2 km au NNW du Post de Katshupa, Plateau de Kundelungu, forêt galerie de la riviére Luanza, à 500 m de la cource, en lisiére, 1690 m, 28 Oct 1968 (fl), *Malaisse 6127* (K, P); Kundelungu, Oct 1950 (fl, fr), *Schmitz 3176* (BR).—Haut-Lomami: Kamunza, 25°50'E, 7°02'S, fin Sep 1957 (fl), *Schmitz 5771* (BR); Riviére Kilwesi, Prov. Katanga, Terr. Mitwaba, Parc national de l’Upemba, 19 Aug 1948 (fl), *de Witte 4121* (WAG—2 sheets).—Kasaï-Oriental: Gandajika, Prov. Kasai, 26 Jun 1956 (fl), *Risopoulos 531* (K, WAG).—Kinshasa: Bas-Congo, Kinshasa, Maluku, Lac Vert, 13 Apr 1970 (fl), *Breyne 864* (MO).—Kongo Central: Kisantu, 1 Nov 1949 (fr), *Callens 2143* (K); Bas-Congo, route Kisantu-Madimba, 20 Sep 1952 (fl), *Troupin 2104* (BR); Leopoldville, Territ. Boma, Estuaire du fleuve Congo, 6 Sep 1958 (fl), *Wagemans 2000* (K).—Kwango: entre Kwango-Wamba tête de sources de la Mpfulula [ca. 5°10'S, 16°40'E], 700 m, 14 Aug 1944 (fl), *Germain 2793* (K).—Lualaba: environs de Kolwezi, Tuissesu Kanamwamfwe, 25°25'114E, 10°37'645S, 22 Aug (fl), *Malaisse & Kisimba Kibuye 117* (K).—Tshopo: lac Yandja (Yangambi), 19 May 1944 (st), *Louis 16985* (K). **UGANDA.** Amua River, West Madi, May 1948 (fl), *Eggeling E.5775* (K—2 sheets). **TANZANIA.** Kassanga area, Ufipa District, 4000–4500 ft, 1960 (fl), *Hopper 1* (EA, K, TFD); T8, Mbinga District, Kitanda ponds at Ndondo Village–Liparamba at 1180 m, 2 May 1991 (fr), *Ruffo & Kisena 3235* (K, TFD). **ANGOLA.** Sumba, Peco, proximum flumen Zaire (Congo), 0 m, 15 May 1923 (fl, fr), *Gossweiler 8747* (BM, K—3 sheets, MO, US); Sumba, Peco, proximum flumen Zaire (Congo), Feb 1925 (fl, fr), *Gossweiler 8957* (BM, K, US); Sumba, Peco, proximum flumen Zaire (Congo), Aug 1926 (fl), *Gossweiler 9126* or *9126A* (B, BM, US); Sumba, Peco, proximum flumen Zaire (Congo), Jul 1926 (fl), *Gossweiler s. n.* (BM, US); Hochland von Quela, 1200 m, Oct 1938 (fl), *Nolde 845* (BM) **. ZAMBIA.** Northern Province, Chinsali District, Shiva Ngandu, 29 Nov 1952 (fl), *Angus 870* (BM—2 sheets, EA, K, MO, NY [as *White 870*]); Luwinga, 15 May 1958 (st), *Angus 1942* (K); Kawambwa, 23 Aug 1957 (fl), *Fanshawe 3557* (K); Chinsali, 25 Sep 1967 (fl, fr), *Fanshawe F10170* (K); Shiwa Ngandu, 3 Aug 1938 (fr), *Greenway & Trapnell 5573* (EA, K); Northern Province, Chinsali District, Chipomo Falls National Monument, along Chimanabuwi River, 11°44'58"S, 32°00'17"E, 1310 m, 17 May 1994 (fr), *Harder et al. 3145* (MO), (fr), *Harder et al. 3164* (MO); Mwinilunga, 10 Sep 1955 (fl), *Holmes 1192* (K); Inono Valley Falls above Mukoma, 3000’, 21 Dec 1954 (fl), *Richards 3696* (K); Abercorn District, Kasulo, 5000’, 11 Jan 1955 (fl), *Richards 4036* (K); Abercorn District, Lucheche R., Abercorn, 1200 m, 10 Oct 1964 (fl, fr), *Richards 19193* (BR, K); Abercorn District, Lunzuwa Falls, 26 Oct 1952 (fl), *Robertson 184* (BM, EA, K, P); Kamuswazi River, Tunduma Road, 5000’, 20 Jul 1970 (fr), *Sanane 1293* (K); Western Province, Fort Rosebery District, near Samfya Mission, Lake Bangweulu, 30 Aug 1952 (st), *White 3165A* (K—2 sheets), near Samfya Mission, Lake Bangweulu, 30 Aug 1952 (fr), *White 3182* (K); Western Province, Mwinilunga District, tributary of Zambezi River, 4 mi N of Kalene Hill Mission, 20 Sep 1952 (fl), *White 3309* (K, MO). **MOZAMBIQUE.** Dist. Moçambique, adm. Mecuburi-Serra Chinga, no vale entre as duas Chinga 10 e Chinga 2, *Aguiar Macedo 3274* (DSM).

*Xylopiarubescens* is readily recognized by the combination of relatively large leaves, which are often orange-red on the abaxial surface of the leaf in dried specimens, twigs with light gray bark, narrow flowers that are often clustered on leafless portions of twigs, stalked glands on the stigmas, and distinctly moniliform monocarps. Throughout its wide range, it is a wetland species. The orange-red cast to the dried leaves, from which the species epithet is presumably derived, is especially pronounced in young leaves. The stalked glands on the stigmas (Fig. 11g) are unique in the genus. William Hawthorne (personal communication) reported that a gland is present on the adaxial surface of the petiole apex in this species, but this characteristic is not discernible in dried specimens and is perhaps only useful for identifying living plants. Specimens are occasionally misidentified as *X.aethiopica*, which shares the relatively large and subcoriaceous leaves, narrow flower buds, and torulose monocarps, and which is also occasionally found in wet habitats. *Xylopiarubescens* differs from *X.aethiopica*, however, in having short inner petals, larger but fewer (<15 per fruit) monocarps, and seeds with the brushlike aril typical of sect. Neoxylopia.

*Xylopiarubescens* has a number of taxonomic synonyms, which represent minor variations connected by intermediates among the collections examined in this study. Engler and Diels (1901) separated *X.humilis* from *X.rubescens* on the basis of the less prominent vein reticulum of the lower leaf surface, but we found this character to vary widely over the range and to be a function of leaf thickness. Engler and Diels, acknowledging that they had not seen the type material of *X.rubescens*, distinguished *X.klaineana* from it on the basis of its thinner leaves that lacked the reddish cast. Le Thomas (1969) reduced *X.klaineana* to a variety of *X.rubescens*, but drew a sharper distinction between the two taxa, calling attention, again, to the thinner leaves with more prominent venation on the abaxial leaf surface, and also to the much larger seeds of the type of *X.klaineana* compared to the type material of nominate *X.rubescens*. Study of material from the entire distribution, however, revealed that mature fruits of this species, such as those present on the type specimen of *X.klaineana*, are seldom collected, and that larger seeds are found from scattered localities across the range. While the seeds of the type specimen of *X.klaineana* are the largest seen for this species (17–20 mm long), they are followed closely, in descending order, by seeds of specimens from Gabon (17–18 mm long, *Breteler & Jongkind 10446*), Cameroon (15–16 mm long, *Fleury 33135*), Bioko, Equatorial Guinea (14.6–14.9 mm long, *Carvalho 2646*), and Ivory Coast (14.5 mm long, *de Koning 4070*). Smaller seeds from throughout the range are sunken and paler brown, with incompletely formed ruminate endosperm, all characteristics of immature seeds. Thus seed size alone is insufficient for maintaining *X.klaineana* as a distinct taxon.

The label of the type specimen of *Xylopiabatesii* describes the plant as having a shrub or vine habit. The leaves of the type are rounded to nearly truncate at the base, and the outer petals are gradually tapered. While this combination of characters is not usual for *X.rubescens*, we found each character to vary independently. For example, the specimen *Harder 3145* from Zambia was described as a lianescent shrub but it has cuneate leaf bases, the specimen *Gossweiler 6222* from Angola (type of *X.gossweileri*) has strongly rounded leaf bases but has abruptly narrowed petals and was collected from a tall tree, and the specimen *Carvalho 2646* from Bioko has very broad petals (over 3 mm wide at the midpoint), strongly cuneate leaf bases, and was collected from a tree 12–15 m tall.

*Xylopiarubescens* has the second-widest distribution of any African *Xylopia* species after *X.aethiopica* and appears to be a wetland opportunist. It has not been collected from most of the Congo River basin within the Democratic Republic of the Congo, where a suitable habitat would seem to be present. Plant associates reported in Cameroon included *Ancistrophyllum* sp., *Cyrtosperma* sp., *Gaertnera* sp., *Gardeniaimperialis*, *Mitragyna* sp., *Raphia* sp., and *Rhynchosporacorymbosa*; in Zambia *Garciniambulwe*, *Mitragynastipulosa*, *Syzygiumelegans*, and *S.cordatum* were noted to be growing with *X.rubescens*.

Seeds of *Xylopiarubescens* are eaten and dispersed by three species of hornbills and three species of monkeys in the Dja Reserve of southern Cameroon. Seeds of *X.rubescens* defecated by hornbills were shown to germinate and at a higher rate than uneaten seeds and seeds recovered from gray-cheeked mangabey fecal samples showed 40% germination when planted (Whitney et al. 1998, Poulsen et al. 2001)

Tessmann (1913) reported that the leaves of this plant (called there *Xylopiazenkeri*) were used in a former time by Fang people in Equatorial Guinea to make a preparation for the treatment of elephantiasis. The leaves were ground together with those of *Dioscoreapreussii* between pieces of bark and then cooked, the resulting water then administered in an enema.

### 4. Xylopia staudtii

Taxon classificationPlantaeMagnolialesAnnonaceae

Engler & Diels, Notizbl. Königl. Bot. Gart. Berlin 2: 298. 1899.

8A471C56-CA96-5971-9ACD-5A68F66390D0

Figs 4A , 13

Xylopicrum staudtii (Engler) Kuntze, Deutsch. Bot. Monatsschr. 21:173–174. 1903. Type. CAMEROON. Southwest Province, Johann-Albrechtshöhe, 1896, *A. Staudt 530* (holotype: B!; isotypes: K! [000105614], P! [00169112, 00169113]).
Xylopia mayombensis De Wildeman, Bull. Jard. Bot. État 4: 386. 1914. Type. DEMOCRATIC REPUBLIC OF THE CONGO [“Belgian Congo”]. Kongo Central Province, Ganda-Sundi, 1913, *Comte J. de Briey 219* (holotype: BR!; isotypes: BR! [8825421, 8825438, 8825445, 8825506]).

#### Description.

***Tree*** up to 35 (–50) m tall, d.b.h. up to 80 cm, bole straight, slender, with branching stilt roots and small buttresses extending from the base, secondary branches horizontal, forming a conical to rounded crown; bark whitish to orangish gray or gray brown, rough, somewhat scaly. ***Twigs*** brown to gray, eventually light gray to light brown, appressed-pubescent, the hairs 0.3–0.4 mm long, soon glabrate; nodes frequently with two axillary branches. ***Leaf*** with larger blades 5.1–11.8 cm long, 2.0–5.6 cm wide, subcoriaceous to coriaceous, discolorous, often paler abaxially, oblanceolate to obovate, occasionally elliptic, apex blunt-acuminate, acumen 2–3 mm long, or acute, base cuneate and decurrent on petiole, glabrous adaxially, sparsely sericeous abaxially; midrib impressed to plane adaxially, raised abaxially, secondary veins brochidodromous, 7–11 per side, diverging at 45–70° from the midrib, plane or raised adaxially, strongly raised abaxially, higher-order veins forming a conspicuous reticulum that is slightly raised adaxially and strongly raised abaxially; petiole 2.5–9 mm long, canaliculate, sparsely appressed-pubescent or glabrate. ***Inflorescences*** axillary, 1–2 (–3)-flowered, sparsely pubescent to glabrate; peduncle 1 per axil, ca. 0.5–2.4 mm long; pedicels 2 per peduncle, 2.5–7 (–8) mm long, 0.8–1.3 mm thick; bracts 2–4, evenly spaced on pedicel, 1.1–2 mm long, semicircular, occasionally tearing down the center as the inflorescence enlarges, apex rounded, occasionally with a tiny apiculum; buds ovoid, apex obtuse. ***Sepals*** spreading at anthesis, 1/4–1/2-connate, 1.6–2.7 mm long, 2.6–3.5 mm wide, coriaceous, broadly ovate to semicircular, apex obtuse to acute, appressed-pubescent. ***Petals*** yellow to yellow-orange *in vivo*, outer petals slightly spreading at anthesis, 5.8–9.6 mm long, 4–5.2 mm wide toward base, 3.5–4.3 mm wide at midpoint, fleshy, ovate, apex acute, appressed-pubescent but with a glabrous patch at base adaxially, velutinous or appressed-pubescent abaxially; inner petals erect to slightly spreading at anthesis, 4.1–8.4 mm long, 2.0–3.2 mm wide toward base, 1.7–2.5 mm wide at midpoint, chartaceous, rhombic to broadly lanceolate, apex acute, base with undifferentiated margin, slightly keeled abaxially, pubescent adaxially, pubescent on apical half and glabrous and verrucose on basal half abaxially. ***Stamens*** ca. 100–120; fertile stamens 1.6–2.1 mm long, clavate, apex of connective 0.3–0.5 mm long, shieldlike but center formed into a conical point, overhanging anther thecae, pubescent, anthers 6–7-locellate, filament 0.3–0.5 mm long; outer staminodes 1.4–1.7 mm long, oblong to broadly clavate, apex obtuse to obliquely truncate; inner staminodes 1.7–2.6 mm long, clavate, apex rounded; staminal cone 1.3–2.1 mm in diameter, 0.4–0.9 mm high, concealing only the bases of the ovaries, rim laciniate. ***Carpels*** 3–11; ovaries 1.3–2.4 mm long, narrowly oblong, tomentose, stigmas free or loosely connivent with tips spreading, 2.6–4.6 mm long, linear, studded with round tubercles on the side and pubescent toward base. ***Torus*** flat, 2.2–3 mm in diameter. ***Fruit*** of up to 5 glabrate or sparsely appressed-pubescent monocarps borne on a pedicel 7–13 mm long, 3–8 mm thick, sparsely pubescent to glabrate; torus 6–16 mm in diameter, 4.3–5 mm high, depressed-globose. ***Monocarps*** with green exterior and scarlet endocarp *in vivo*, 3.7–9.8 cm long, 1.2–2.1 cm wide, 1.0–1.7 cm thick, oblong and somewhat falcate, occasionally weakly torulose, apex rounded or with a curved beak or mucro 1.3–3 mm long, base contracted into a stipe 7–15 mm long, 2.5–6 mm thick, finely wrinkled, strongly verrucose; pericarp 0.7–2.5 mm thick. ***Seeds*** 1–5 per monocarp, in a single row, lying parallel to long axis, 14–19 mm long, 9–12.9 mm wide, 9–11 mm thick, oblong-ellipsoid, oblong-elliptic in cross section, truncate at micropylar end, rounded at chalazal end, brown to black, smooth, shiny, raphe/antiraphe forming a raised ridge encircling the seed, micropylar scar 1.5–3.5 mm long, 1.5–2.0 mm wide, elliptic to circular; sarcotesta absent; aril bright yellow or orange, rarely pink or violet *in vivo*, amber-colored when dried, brushlike, 10–14 mm in diameter, 4.8–8 mm high, fleshy, granular.

#### Phenology.

Specimens with flowers and with fruits have been gathered in all months of the year and with fruits from all months except March. In Sierra Leone, “flowers appear during the rains from July to August and fruits are ripe by October” (Savill and Fox 1967); in Ghana, the flowering season is given as June to October, and the fruiting season as January to March (Hall and Swaine 1981).

#### Distribution

(Fig. 10). Occurs from Sierra Leone to Ghana, and then again from eastern Nigeria east to southwestern Uganda and south to the Cabinda Province of Angola and east-central Democratic Republic of the Congo; high forest and occasionally freshwater swamp forest, at elevations from sea level up to 1350 m. In Sierra Leone, the trees have a preference for moist valleys, and can be weedy in timber regeneration plots (Savill and Fox 1967).

#### Local names.

Diroma (*Gossweiler 7992*), drehn (*Cooper 60*, *234*), duanan (Ghana, *Vigne 982*, Hall and Swaine 1981), fofois (Gola, *Voorhoeve 19*), fondé (*Aubréville 38*, *66*, *1941*), niumbi (Kitetela, *Germain 7631*), nkala (*Hauzer 29*; Bulu, *de Wilde 7941*), ntom (Ntoumou, Focho et al. 2010), odjobi (Ntomou, Focho et al. 2010; *Letouzey 8178*), takon-blu-chu (*Cooper 139*), yengetomei (Sierra Leone, Savill and Fox 1967).

**Figure 13.**
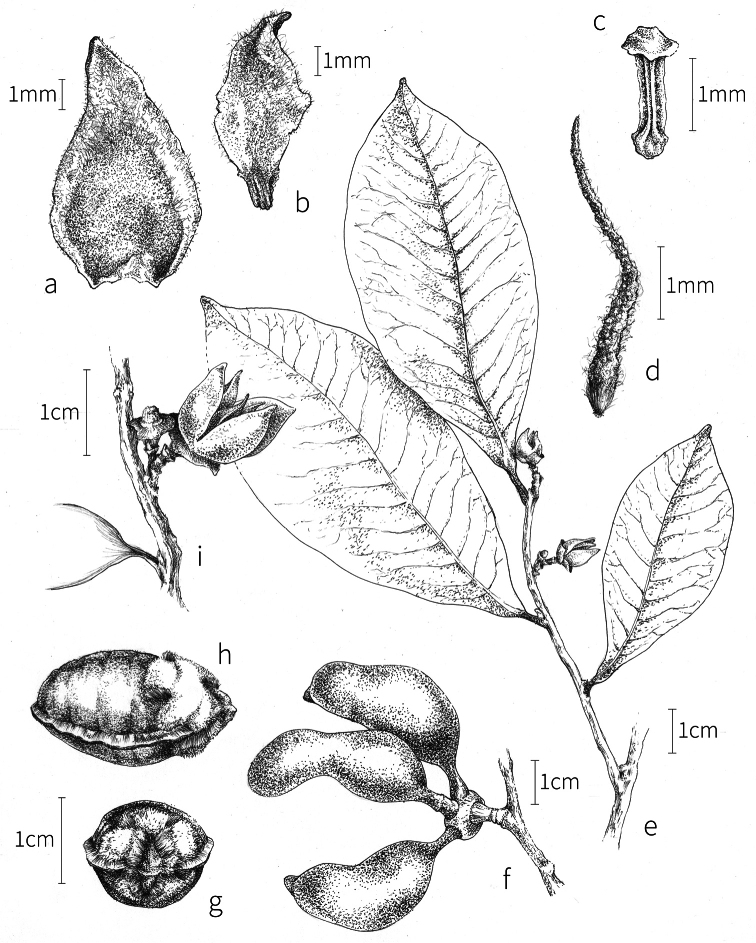
*Xylopiastaudtii*. **A** Outer petal, adaxial view **B** Inner petal, adaxial view **C** Stamen, abaxial view **D** Carpel **E** Habit **F** Fruit **G** Seed, view of micropylar end **H** Seed, lateral view **I** Close-up of inflorescence. **A–D** from *Le Testu 8630* (BM) **E, I** from *Evrard 5212* (BR) **F** from *Reitsma 1071* (WAG) **G, H** from *Reitsma & Reitsma 1168* (BISH).

#### Additional specimens examined.

**SIERRA LEONE.** Yengetumeh M., Kambin Hangha, 21 Aug 1918 (buds), *Aylmer 239* (K); Neaboi Valley, Kambui Forest Reserve, 1 Apr 1955 (fr), *Jordan 2024* (K); Gola Forest, Bagbe Line, 21 May 1952 (buds), *Small 724* (B, K, MO, P). **LIBERIA.** Saniquellie, Kitoma, 21 Mar 1959 (st), *Adam 16813* (MO), 17 Dec 1969 (fr), *Adam 25287* (MO); 20 Oct 1971 (st), *Adam 26378* (MO); Kakata, Blazie, 7 Mar 1959 (st), *Adam 16733* (MO, WAG); Ganta, 30 km S Ganta, 15 Oct 1975 (fr), *Adam 29894* (MO); from vicinity of Firestone Plantations along Dukwia ["Dukwai"] R., Monrovia, 3 Oct 1928 (fl, fr), *Cooper 60* (BM, F, GH, K, NY, US, YF), 17 Nov 1928 (fl, fr), *Cooper 139* (BM, F, GH, K, NY, PH, US, YF), 23 Feb 1929 (st), *Cooper 234* (BM, F, K—2 sheets, NY, PH, US, YF); Firestone Plantation #3, 2 Aug 1926 (fl, fr), *Linder 66* (A, K—2 sheets); near Blohni, Blohni River drainage, 28 Apr 1948 (fr), *Mayer 97* (US); Bong Range, 200 m, 19 Jul 1960 (fr), *Voorhoeve 19* (WAG). **IVORY COAST.** Abidjan, *Aubréville 38* (B, K, P), *66* (K, P, US); Banco, undated (fr), *Aubréville 1941* (A, P); forêt du Téké, Adzopé-Abidjan km 73, 5°33'N, 4°03'W, 11 Feb 1969 (fr), *Bamps 2044* (BR); Abidjan, Banco Forest Reserve, part N of Route du Rail, 29 Apr 1976 (fr), *de Koning 6833* (WAG—3 sheets). **GHANA.** Tarkwa, Subiri F. R. (Benso), Sept 1951 (fl), *Andoh A30/51 (FH 5561)* (B, K, NY, P); Neung Forest Reserve (W), Dompim, 16 May 1974 (fr), *Enti FE-1315* (K, MO, WAG); Neung Forest Reserve, nr. Bonsa River, Tarkwa Dist., 3 Sept 1981 (fr), *Enti FE-2095* (MO); Atewa F. R., 1800’, 25 Feb 1971 (fl, fr), *Hall GC 43251* (MO); Western Province, Elubo, approx. 22 km ESE towards Takoradi, along Elubo-Taboradi road, then N 8 km on road to Ankasa Forest Reserve, N of Ankasa River, 05°17'N, 02°45'W, 80 m, 14 Jul 1995 (fl, fr), *Harder et al. 3406* (MO); Abosso, Aug 1926 (fl), *Vigne 982* (K, P). **NIGERIA.** Southern Nigeria, Central Province, 1931 (fl), *Rosevear C.21* (K). **CAMEROON.** East Province, Department Haut-Nyong, Dja Reserve, Bouamir Research Area, 90 km SE of Akonolinga, 03°12'N, 12°49'E, 640–700 m, 24 Aug 1994 (fl), *Fogiel 864* (MO—2 sheets); 22 km à l’Ouest de Masea (village situé à 50 km au SSW de Yokadouma, 4 Jul 1963 (fl), *Letouzey 5404* (P); Mebemonko, 20 km NO d’Oveng, 24 Oct 1966 (fl, fr), *Letouzey 8178* (K, P); près Nteigne, Pk 108, Route Mintom–Mbalam, near Djoum, Jan 1973 (fl), *Letouzey 11854* (K); colline a l’ENE de Mbalam 140 km ESE de Djoum près Souanke-Congo, 20 Jan 1973 (fl, fr), *Letouzey 11865* (BR, K—2 sheets, P); Übergangs- und Kampfgebiet gegen die Savanne an der Nord-grenze der Hylaea südlich des Sanaga zwischen Jaunde und Dengdeng unweit der Vereinigung von Lom (Sanaga) und Djerem, etwa 88 km NO Jaunde, Feb 1914 (fl), *Mildbraed 8171* (K); Southwest Province, forested lower slopes of Mt. Cameroon above Batoke, 4°05'N, 9°05'E, 300–600 m, 24 Apr 1984 (fl), *Thomas 3463* (B, K, MO); Prov. Southwest, Takamanda Forest Reserve, footpath from Mbilishi to Kaluma, 6°15'N, 9°26'E, 650 m, 1 May 1987 (fr), *Thomas et al. 7401* (MO); Station du Cacaoyer de N’koemvone, 11 km on the road from Ebolowa to Ambam, 2°49'N, 11°08'E, 31 Jan 1975 (fl), *de Wilde 7941* (B, BR, K, MO, P); Station de Cacaoyer de N’Koemvone, 14 km on the road from Ebolowa to Ambam—track crossing the Mvila river, 2°49'N, 11°06'E, 4 Mar 1975 (fl), *de Wilde 8029* (B, K, MO); South Province, hill above Nlonacko near village Ebianemeyong, c. 2°26'N, 10°21'E, 500 m, 12 Dec 1998 (fl), *de Wilde et al. 12161* (MO); Mimfia, Mar 1913 (fl), *Zenker 246* (B, G, GH, M, MO, P, US, WAG); Bipinde, 1908 (fl), *Zenker 3653* (BM, F, G, K, L, M, MO, US, WU), Bipinde, Mimfia, Aug 1909 (fl), *Zenker 3953* (BM, F, G, K, L, M, MO, P, WU); Bipinde, 1903 (fl), *Zenker 4862* (B, BM, G, K, L, M, MO, P, PR, US). **EQUATORIAL GUINEA.** Río Muni: Près de la frontière gabonaise, chantier forestier à l’est de Cogo, 1°05'N, 10°00'E, 3 May 1989 (bud), *McPherson 14003* (MO); Bebai, Campo-gebiet, Weg u Tkum [locality is in NE corner of modern Equatorial Guinea (Tessmann 1913)], 18 Nov 1908 (fl), *Tessmann 644* (K). **GABON.** Estuaire: S of Estuaire du Gabon along Remboué River, British Gas site, 00°12'S, 10°01'E, 10 m, 11 Jan 1991 (fl, fr), *McPherson 15113* (MO); côté plantation Sogacel 3 km N de Ndouaniang, 12 Aug 1984 (fl, fr), *Wilks WIL 991* (WAG—2 sheets).—Haut-Ogooué: Plateau Batéké National Parc, 02°06'59"S, 14°04'03"E, 421 m, 26 Feb 2003 (fr), *Niangadouma & Walters 144* (MO, WAG).—Ngounié: new rd from Mouila to Yeno, 5 km either side of Kembela village, 1°42'S, 11°23'E, 20 Jul 1986 (fl, fr), *Thomas & Wilks 6515* (K, MO, P).—Nyanga: région du Nyanga, Inganga, Mayombe Bayaka, 20 May 1914 (fl), *Le Testu 1748* (BM, MO, P, US); forêt du Mayombe Bayaka, près de Tchibanga, 10 Dec 1914 (fl), *Le Testu 1913* (BM, K—2 sheets, MO, P); chantier CEB, 35 km SW of Doussala, 2°30'S, 10°30'E, 19 May 1985 (fl), *Reitsma et al. 1071* (MO, NY, RSA, WAG); chantier CEB, ca. 50 km SW of Doussala, 2°36'S, 10°35'E, 14 Jun 1985 (fr), *Reitsma & Reitsma 1168* (BISH, MO, NY, RSA).—Ogooué-Maritime: Toucan, ca. 01°47'S, 09°53'E, 9 Jun 2002 (fl, fr), *Bourobou Bourobou et al. 707* (MO—3 sheets).—Ogooué-Ivindo: M’Passa Field Station, near Makokou on Riviere l’Ivindo, 8 Jul 1981 (fr) *Gentry 33219* (MO); Ile Ipassa, Makokou, 17 Jun 1972 (fr), *Hladik 2334* (P); Monts Iboundji, 27 Dec 1930 (fl), *Le Testu 8630* (BM, BR, P).—Woleu-Ntem: Oyem, région entre Ogooué et Cameroun, 9 Sep 1933 (fl), *Le Testu 9287* (BM, BR, K—2 sheets, P).—Province unknown: Billagone, 100’, 22 May 1938 (fl), *Thomson 2* (K). **REPUBLIC OF THE CONGO**. Route du chantier de Boungolo (Pointe-Noire), 31 Jan 1966 (fl), *Farron 4892* (K, P). **DEMOCRATIC REPUBLIC OF THE CONGO.** Équateur: Entre Bokatola and Bikoro, Sep 1930 (fl), *Lebrun 1425* (BM, K, MO, NY, RSA, US).— Ituri: Ituri District, Lodjo, SW of Lodjo village, 2°03.09'N, 29°59.51'E, 1080 m, 26 Jan 2011 (fr), *Bytebier et al. B 3367* (K, MO).—Kongo Central: Kisafu [Maduda], 10 Oct 1951 (fr), *Hauzer 29* (BR).—Sankuru: Katako-Kombe, Rubber Plantation, Jun 1952 (fr), *Germain 7631* (BR).—Sud-Kivu: Mukono, Terr. Mwenga, 8 Jul 1959 (fl, fr), *Leonard 4877* (BR).—Tshopo: Tsangi Terr., Yangambi, 120 m, 5 Mar 1952 (fl), *Toussaint 918* (K).—Tshuapa: Piste Yalikungu-riv. Tshuapa, Terr. Ikela, 25 Nov 1958 (fl), *Evrard 5212* (BR, K). **ANGOLA.** Mayumbe, M’bulu hills, source of N’Zanga River, 11 Apr 1919 (fl, fr), *Gossweiler 7992* (BM, BR, K—3 sheets). **UGANDA.** Impenetrable Forest, Kigezi, Apr 1946 (fl), *Butt 45* (ENT, not seen, photo at K); [U2], Rukungiri District, Kayonza, Bwindi forest, Ishasha Gorge, 0°53’–1°08'S, 30°25’–30°35'E, 1350 m, Apr 1998 (fl), *Hafashimana 0504* (K).

*Xylopiastaudtii* is the tallest of any *Xylopia* species, becoming a canopy tree of up to 50 m. The elaborate stilt roots of this species, described in detail by Jeník (1970), consist not only of adventitious stilt roots emerging from the trunk up to one meter above the ground, but also stilted pneumatophores (“peg roots”) that arise from lateral roots up to 10 m distant from the tree trunk. These striking roots suggest adaptation to swamp forests, although the species is not restricted to such habitats.

With its broad flower buds, *Xylopiastaudtii* is most similar to *Xylopiaafricana*, also sharing with that species the oblong thick-walled monocarps and large seeds with a brushlike aril. In fact, many collections of *X.staudtii* made by Zenker in Cameroon were distributed as *X.africana*. The lone taxonomic synonym, *Xylopiamayombensis* De Wild., was based on a specimen with petals at the larger end of the range of petal size for *X.staudtii* but is not otherwise exceptional for the species.

Sunderland et al. (2003) reported *X.staudtii* to be a dominant tree in lowland (300–500 m), mid-elevation (500–800 m), and montane (800–1500 m) forests of the Takamanda Reserve, having the second-highest relative density and the fourth-highest basal area of all tree species sampled in montane forest plots. The seeds of *X.staudtii* have been reported as a food item for two hornbill species (Whitney et al. 1998), four species of mangabeys and guenons (Sourd and Gautier-Hion 1986, Poulsen et al. 2001), and mandrills (Lahm 1986). The nutrient analysis of the arils of *X.staudtii* by Sourd and Gautier-Hion (1986) showed high levels of lipids and proteins, suggesting that the species provides a high-value food resource to these dispersers and perhaps others. Some seeds defecated by gray-cheeked mangabeys and by hornbills germinated (Whitney et al. 1998, Poulsen et al. 2001).

### II. Xylopia Section Ancistropetala

Taxon classificationPlantaeMagnolialesAnnonaceae

(Engler), D. M. Johnson & N. A. Murray, Syst. Bot. 42(2): 221. 2017.

A9E2D917-1D0D-578B-9845-94A47A2CA2B6

Artabotrys section Ancistropetalum Engler, Monogr. afrik. Pflanzen-Fam. 6: 71. 1901.

#### Type.

*Artabotrys auranti[i]odorus* (De Wildeman & T. Durand) Engler [=*Xylopiaaurantiiodora* De Wildeman & T. Durand].

#### Description.

**Figure 14.**
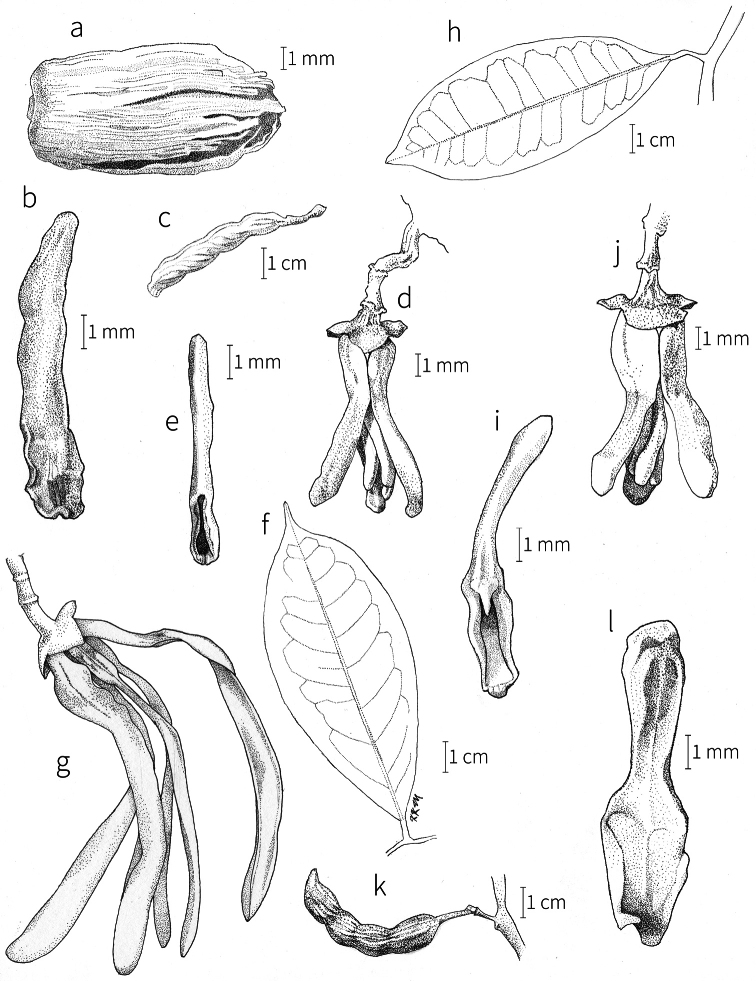
*Xylopiaquintasii*, *X.congolensis*, and *X.aurantiiodora*. **A–F***X.quintasii***A** Seed, lateral view **B** Outer petal, adaxial view **C** Monocarp, lateral view **D** Flower, lateral view **E** Inner petal, adaxial view **F** Leaf **G***X.congolensis*, flower, lateral view. **H–K***X.aurantiiodora***H** Leaf **I** Inner petal, adaxial view showing tooth **J** Flower, lateral view **K** Monocarp, attached to pedicel **L** Outer petal, adaxial view. **A, C** from *Reitsma 1423* (WAG) **B, D–F** from *de Wilde et al. 9156* (WAG) **G** from *Tutin 80* (MO) **H, K** from *Harris 752* (MO) **I, J, L** from *Harris & Fay 1846* (MO).

Nodes with a branch from a single axillary bud; outer and inner petals linear, similar in length; inner petals with differentiated fleshy basal margins (Fig. 14E, I); anther connectives conical to globose at apex, not overhanging anther thecae: staminal cone absent; carpels 3–5, stigmas free, not connivent; aril fimbriate, enveloping seed; seed coat smooth, sarcotesta absent. Three species in Tropical Africa.

#### Notes.

Section Ancistropetala comprises species restricted to wet forests of western and central Africa. Species of the section are set apart from all other members of the genus by the fimbriate membranous orange arils and conical to globose anther connectives. Differentiated inner petal margins are present outside of sect. Ancistropetala only in the species *Xylopiatoussaintii*, *X.gilbertii*, and *X.flamignii* of section Stenoxylopia (Fig. 23D, G), but these species have a distinct staminal cone, anther connectives that are truncate at the apex, and seeds with an orange sarcotesta, and the seeds lack arils.

### 5. Xylopia aurantiiodora

Taxon classificationPlantaeMagnolialesAnnonaceae

De Wildeman & T. Durand, Ann. Mus. Congo, Sér. 2, Bot. 1(1): 4. 1899.

DA3B5BB3-8F68-5253-82C9-8C1481F6FA0E

Fig. 14H–K

Artabotrys aurantiiodorus [“aurantiodorus”] (De Wildeman & T. Durand) Engler, Monogr. afrik. Pflanzen-Fam. 6: 76. 1901. Type. DEMOCRATIC REPUBLIC OF THE CONGO [“Belgian Congo”]. Équateur Province [“Région III”], Mbandaka [“Coquilhatville”], 24 Jan 1896, *A. Dewèvre 660* (holotype: BR!; isotypes: BR! [8824271, 8824677, 8824684, 8824691]).
Xylopia bequaertii [“bequaerti”] De Wildeman, Pl. bequaert. 1: 469–470. 1922. Type. DEMOCRATIC REPUBLIC OF THE CONGO [“Belgian Congo”]. Tshopo Province, Kisangani [“Stanleyville”], bords boisés de la Tshopo, 27 Feb 1915, *J. C. C. Bequaert 6994* (holotype: BR!).

#### Description.

***Tree or shrub*** 2–10 m tall, d.b.h. up to 30 cm; bark light colored. ***Twigs*** brown to gray, glabrous or with sparse hairs 0.2–0.3 mm long, usually marked by two ridges decurrent from each petiole base, epidermis eventually exfoliating. ***Leaf*** with larger blades 6.3–11.1 cm long, 2.5–4.7 cm wide, chartaceous to subcoriaceous, discolorous, the lower surface tan-colored, elliptic to oblong-elliptic, occasionally oblanceolate or obovate, apex rounded, obtuse, or acute, occasionally emarginate, base cuneate and decurrent, glabrous adaxially, glabrous or with a few scattered hairs abaxially; midrib plane to slightly raised adaxially, raised and somewhat keeled abaxially, secondary veins brochidodromous, 10–14 per side, diverging at 60–70° from the midrib, these and higher-order veins forming a raised reticulum on both surfaces; petiole 2.5–7 mm long, shallowly canaliculate, glabrate or sparsely pubescent. ***Inflorescences*** axillary, 1–5-flowered, occasionally with two pedicels borne on a common peduncle, glabrous to sparsely pubescent; peduncle 1.5–2.0 mm long; pedicels 3.5–7.1 mm long, 0.5–1.0 mm thick; bracts 2–3, the uppermost often just beneath the sepals, caducous, 1.1–1.5 mm long, semicircular, apex rounded; buds panduriform, apex obtuse. ***Sepals*** free and somewhat imbricate at the base, 1.5–2.1 mm long, 1.9–2.4 mm wide, coriaceous, semicircular to triangular, apex apiculate, pubescent abaxially with hairs extending beyond margins to form cilia. ***Petals*** white or pale yellow to greenish yellow *in vivo*; outer petals erect with the apices slightly spreading at anthesis, 8.9–12 mm long, 2.4–3.5 mm wide, 1.8 mm wide at midpoint, fleshy, ligulate-lanceolate, apex obtuse, pubescent except for glabrous center of basal concavity adaxially, buff-brown pubescent abaxially; inner petals with apices divergent, 9.3–11.2 mm long, 1.2–2.3 mm wide at base, ca. 0.8 mm wide at midpoint, narrowly oblong, apex obtuse, base with differentiated margin, with a truncate tooth 0.8–1.1 mm long overhanging basal concavity, densely puberulent on both surfaces except for the glabrous differentiated margins and basal concavity. ***Stamens*** 40–60; fertile stamens ca. 1.8 mm long, oblong, apex of connective 0.2–0.3 mm long, globose, not overhanging the anther thecae, papillate, anthers 6–8-locellate, filament ca. 0.5 mm long; outer staminodes ca. 1 mm long, broadly clavate, apex rounded, emarginate, or bilobed; inner staminodes absent; staminal cone absent. ***Carpels*** 3–5; ovaries 0.7–1 mm long, globose, pubescent; stigmas discrete, not connivent, 0.6–0.8 mm long, narrowly oblong, glabrous except for fine setae at the apex. ***Torus*** flat, 1.5–1.8 mm in diameter. ***Fruit*** of up to 3 glabrate monocarps borne on a pedicel 6.5–10 mm long, 1.2–1.8 mm thick, slightly curved, sparsely pubescent to glabrate; torus 2.8–4 mm in diameter, 2–2.5 mm high, depressed-globose. ***Monocarps*** green *in vivo*, 3.3–6.5 cm long, 0.7–0.9 cm wide, 0.7–0.8 cm thick, finger-shaped to narrowly oblong, strongly torulose, apex acute to rostrate, beak up to 5 mm long, base abruptly contracted into a stipe 5.5–9.5 mm long, 2–3 mm thick, wrinkled and somewhat flattened, striate and somewhat shining; pericarp 0.1–0.4 mm thick. ***Seeds*** 1–4 per monocarp, in a single row, parallel or slightly oblique to long axis, 13.3–21 mm long, 5.5–8.3 mm wide, 5.5–7.5 mm thick, narrowly ovoid to oblong, circular to elliptic in cross-section, obliquely truncate at micropylar end, distinctly pointed at the chalazal end, light reddish brown, smooth, raphe/antiraphe not evident, micropylar scar 2.7–4.5 mm long, 2–2.5 mm wide, elliptic; sarcotesta absent; aril red *in vivo*, orange-brown when dried, fimbriate, extending the length of the seed, membranous, smooth.

#### Phenology.

Specimens with flowers have been collected from January to March and from May to November; fruits have been collected in May, June, August, and September.

#### Distribution

**Figure 15.**
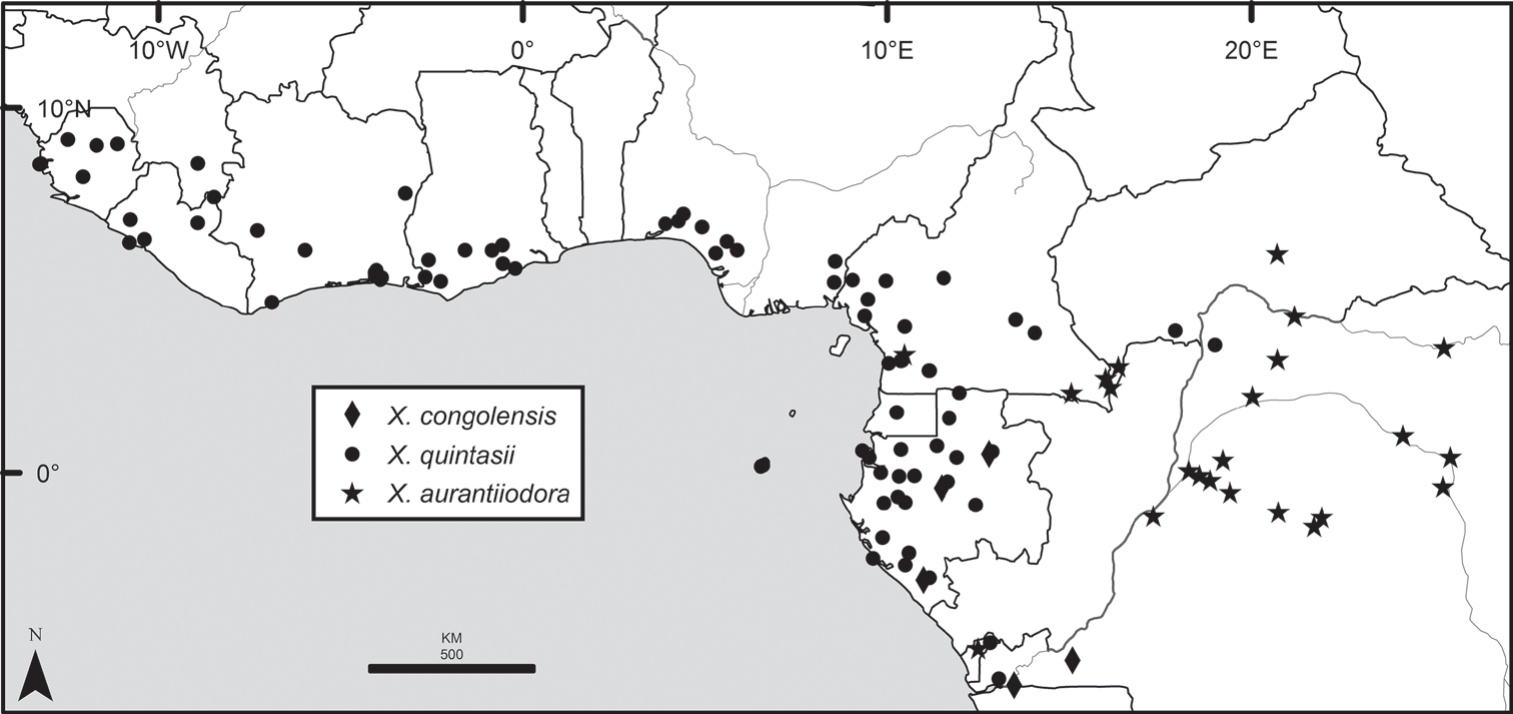
Distributions of *Xylopiaaurantiiodora*, *X.congolensis*, and *X.quintasii*. Bolder lines represent country borders, fainter lines lakes and major rivers.

(Fig. 15). *Xylopiaaurantiiodora* is found in the Congo River basin in the Democratic Republic of the Congo, the southern Central African Republic, and eastern Cameroon, with disjunct occurrences in the Cabinda Province of Angola and near Lolodorf in southern Cameroon. It grows in riparian forest, on riverbanks, and in inundated forest, from near sea level up to 470 m.

#### Local name.

Gongo-sange (Bangala, *Robyns 465*).

#### Additional specimens examined.

**CAMEROON.** Eastern Province, west bank of the Sangha River, 02°23'N, 16°10'S, 22 May 1988 (fr), *Harris & Fay 752* (K, OWU); Est, west bank of Sangha River opposite Ndakan camp and 2 km S, 02°21'N, 16°08'E, 12 Feb 1989 (fl), *Harris & Fay 1846* (GH, MO); Mbekou près Moloundou, 17 Oct 1949 (fl), *Letouzey SRFK 1374* (P); env. de Lolodorf, 1919 (fl), *Rouyer s. n.* (L). **CENTRAL AFRICAN REPUBLIC.** Sangha-Mbaere, west bank of Sangha River, opposite Bayanga, ca. 200 m upstream, 02°55'N, 16°21'E, 20 Feb 1991 (fl), *Harris 2750* (OWU); bord. Riv. Ouaka [“Waka”] 30 km N Bambari, 29 Jan 1923 (fl), *Tisserant 936* (BM, P, US). **DEMOCRATIC REPUBLIC OF THE CONGO.** Bas-Uele: Terr. Bambesa, bord de l’Uéle, 20 Feb 1956 (st), *Gerard 2162* (WAG).—Équateur: Eala, 7 Jul 1933 (fl), *Corbisier-Baland 1687* (BM, K, MO, P, US); Scierie de Loukoléla, 26 Aug 1912 (st), *Chevalier 28289* (P); Eala, *Couteaux 425* (K, P); Botsima, parc au layon central, 1°09'S, 21°57'E, 28 Mar 1991 (fl), *Dhetchuvi 1067* (BR); District de la Tshuapa [Basankusu, fide Boutique 1951b], Jul 1934 (fl), *Dubois 485* (K, MO, P); district de la Tshuapa, Aug 1938 (fr), *Dubois 972* (K); Popolo, forêt inondée de la Mongala, 10 Aug 1955 (fr), *Evrard 1594* (BR); Popolo, forêt marécageuse de la Mongala, *Evrard 1658* (P); Territ. Bolomba, R. Busisa or Busira, en amont de Isalo, Oubangui, 2 Mar 1958 (bud), *Evrard 3601* (K); Territ. Monkoto, river Yenge, 25 km from mouth, 2 Aug 1958 (bud), *Evrard 4431* (K); Territ. Bokota, Watsi Kengo, inundated forest of the Salonga (hautes eaux), 14 Nov 1957 (fl), *Evrard 2964* (K); Wangata, Aug 1930 (fl), *Lebrun 922* (A, K, MO, P, PR); entre Eala et Boyeka, rive droite de la Ruki, 8 Sep 1925 (fl, fr), *Robyns 465* (B, BR—2 sheets, K); environs de Mbandaka [“Coquilhatville”], 15 Sep 1925 (fr), *Robyns 580* (A, B, K—2 sheets, P).—Maniema: Secteur Bangengele, Parc National Proposé de la Lomami, ca. 3.9 km au NNO de Katopa (en ligne directe) en descendant la rivière Lomami, 02°43'00"S, 025°05'03"E, 430 m, 3 May 2015 (fl), *Gereau et al. 7632* (MO).—Mongala: Monza (village) along the Loika (Itimberi), 02.0491°N, 022.743°E, 335 m, 30 Jun 2009 (fr), *Stoffelen 453* (MO).—Nord-Ubangi: entre Banzyville et Yakoma (Ubangi), Feb 1931 (fl), *Lebrun 2167* (K, MO, P).—Tshopo: Terr. Isangi, Yangambi, île Tutuku, Jul 1963 (fl) *Bolema 1192* (K, P, WAG—2 sheets); 0°26'S, 25°28'E, Ubundu, rives du fleuve Zaire et îles en amont d’Ubundu, 12 Mar 1978 (fl), *Lejoly 2915* (BR); près d’Ubundu, bord d’une île sur le Zaire, 11 Mar 1978 (fl), *Lisowski 47959* (K); env. de Yangambi, bord du Zaire au pied de la Falaise, 28 Apr 1979 (bud), *Lisowski 52352* (K); Yangambi, 17 Aug 1937 (fl), *Louis 5804* (B, BM, F—2 sheets, K, MO, NY, P, US); Yangambi, 9 Feb 1938 (fl), *Louis 7839* (B, K, MO, P, US), Yangambi, 21 Mar 1938 (fl), *Louis 8542* (MO); Yangambi, 28 Jul 1938 (fl), *Louis 10543* (BM, EA, FI-T, K, P), Yangambi, 3 Aug 1938 (fr), *Louis 10671* (BM, EA, FI-T, K, P); Yangambi, 13 Aug 1938 (fr), *Louis 10790* (BM, EA, FI-T, K, P), Yangambi, 29 Jan 1939 (fl), *Louis 13464* (BM, K); Yangambi, île Tutuku, 31 Jul 1939 (fl), *Louis 15674* (K, MO, P, US). **ANGOLA.** Maiombe, banks of river Luali, *Gossweiler 7168* (BM, COI-00067909); banks of the river Luali at Sera, Maiombe, 7 May 1917 (fl), *Gossweiler 7169* (BM, COI-0067910); Maiombe Portuguesa, Sera, proximum flumen Luali-Chiloango, Jun 1924 (fl, fr), *Gossweiler 9006* (B, BM, K).

*Xylopiaaurantiiodora* differs from *X.congolensis* and *X.quintasii* in having a prominent tooth on the inner surface of the inner petals (Fig. 14I), strongly striate monocarps that are acute at the apex, and larger pointed seeds. The tooth of the inner petal is especially useful for identification, because it appears early in petal development and is visible even in young buds. The connivance of the bases of the inner petals is more pronounced in this species than in other species of the genus and is reminiscent of *Artabotrys*; presumably on this basis, *X.aurantiiodora* was moved to *Artabotrys* by Engler and Diels (1901), who erected a special section for it. *Xylopiaaurantiiodora* also differs from *X.congolensis* and *X.quintasii* in both habit and habitat, being a riverine tree reaching only 10 m in height, while the other two species are trees of upland forest commonly reaching a height of 20 m or more. Associated species of *X.aurantiiodora* mentioned on herbarium labels include *Irvingiasmithii*, *Trichiliaretusa*, and *Uapacaheudelotii*. The species epithet marks the field observation of Dewèvre that the flowers had the scent of citrus flowers.

We treat *Xylopiabequaertii* as a taxonomic synonym of *X.aurantiiodora*, as previous authors have (e.g. Boutique 1951b), observing only that, in his original description of *X.bequaertii*, De Wildeman (1922) made no mention of *X.aurantiiodora* but instead contrasted *X.bequaertii* with *X.striata* Engler, drawing attention to floral differences between the two species as well as the larger number of secondary veins in the leaves of *X.bequaertii*.

### 6. Xylopia congolensis

Taxon classificationPlantaeMagnolialesAnnonaceae

De Wildeman, Ann. Mus. Congo, Sér. 5, Bot. 1: 41–42. 1903.

AE8438B5-5E78-58B5-8B1E-046F0E5A7FF7

Fig. 14G

#### Type.

DEMOCRATIC REPUBLIC OF THE CONGO [“Belgian Congo”]. Kongo Central Province, Inkisi-Kisantu [“Kisantu”], 1900, *J. Gillet 812* (lectotype, here designated: BR!; isotypes: BR! [0000008824707, 0000008824714, 0000008824721, 0000008824769, 0000008824776]).

#### Description.

***Tree*** up to 20 m tall, d.b.h. up to 50 cm; bark medium brown to red-brown, flaking. ***Twigs*** brown to shiny reddish brown or orange-brown, eventually light gray to gray-brown, glabrous, the bark sometimes exfoliating in strips, often marked with sharp ridges decurrent from both sides of petiole base. ***Leaf*** with larger blades 6.9–11.5 cm long, 2.6–4.4 cm wide, subcoriaceous to chartaceous, discolorous, oblanceolate to narrowly elliptic, apex acuminate, the acumen 4–11 mm long, base cuneate and decurrent, glabrous on both surfaces; midrib slightly impressed to plane adaxially, raised and sharply keeled abaxially, secondary veins brochidodromous, 9–12 per side, diverging at 45–75° from the midrib, often festooned with extra loops on the outside, secondary and higher-order veins indistinct to slightly raised adaxially, raised abaxially; petiole 3–8 mm long, shallowly canaliculate, glabrous or with a few hairs. ***Inflorescences*** axillary or from the axils of fallen leaves, 1–4-flowered, appressed-pubescent to glabrate; pedicels arising from axils independently, rarely from a common peduncle ca. 2 mm long, pedicels 3.4–6.7 mm long, 0.7–1.0 mm thick, often curved; bracts 2–4, caducous, rarely persistent, 0.8–1.5 mm long, semicircular to lunate, apex rounded; buds narrowly oblong, apex obtuse. ***Sepals*** slightly spreading at anthesis, free or 1/8–1/6-connate, 1.5–2.5 mm long, 2.0–2.5 mm wide, coriaceous, triangular to triangular-ovate, apex acute to obtuse, appressed-pubescent abaxially. ***Petals*** white, pale green, or yellowish white *in vivo*; outer petals slightly spreading at anthesis, 12.7–22 mm long, 2.1–3.3 mm wide at base, 1.0–2.3 mm wide at midpoint, coriaceous or slightly fleshy, linear-ligulate, apex obtuse, densely puberulent except for small glabrous patch at base adaxially, appressed-pubescent abaxially; inner petals more or less erect at anthesis, 10.3–18.6 mm long, 0.9–1.0 mm wide at base, 0.6–0.9 mm wide at midpoint, coriaceous or slightly fleshy, linear or sometimes slightly spatulate, apex obtuse, base with differentiated fleshy or membranous margins, densely puberulent on both surfaces except in basal concavity and on fleshy margins. ***Stamens*** 48–61; fertile stamens 1.5–1.8 mm long, oblong, apex of connective 0.4–0.5 mm long, conical, not overhanging anther thecae, long-papillate, anthers 8–10-locellate, filament 0.3–0.5 mm long; outer staminodes 1.2–1.5 mm long, oblanceolate, apex obtuse; inner staminodes apparently absent; staminal cone absent. ***Carpels*** 3–5; ovaries 1.0–1.6 mm long, oblong, densely pubescent, stigmas discrete, not connivent, 0.4–0.7 mm long, narrowly oblong to clavate, glabrous. ***Torus*** flat, 1.7–2.1 mm in diameter. ***Fruit*** of up to 3 glabrate monocarps borne on a pedicel 12–14.8 mm long, 1.6–2 mm thick, glabrate or with scattered hairs; torus 3–4.7 mm in diameter, 2.5-3 mm high, globose. ***Monocarps*** 3.7–4.6 cm long, 0.6–0.9 cm wide, 0.8–1.0 cm thick, linear-oblong, weakly torulose, apex rostrate, the beak ca. 2.5 mm long, base contracted into a more or less distinct stipe ca. 6 mm long, 2.4–2.5 mm thick, longitudinally wrinkled and obliquely striate, faintly verrucose; pericarp ca. 0.5 mm thick. ***Seeds*** up to 5 per monocarp, in a single row, lying parallel to long axis, 10.4–11.2 mm long, 6.8–7.0 mm wide, ca. 6.7 mm thick, ellipsoid, elliptic in cross-section, truncate at micropylar end, rounded or obtuse at chalazal end, brown, smooth, dull to shiny, raphe/antiraphe not evident, micropylar scar 2.7–4 mm long, 3–3.7 mm wide, roughly circular; sarcotesta absent; aril color *in vivo* unknown, straw-colored when dried, fimbriate, extending the length of the seed, membranous, smooth.

#### Phenology.

Specimens with flowers have been collected in February and once in August, with large buds in January, and with fruits in October.

#### Distribution

(Fig. 15). Gabon and southwestern Democratic Republic of the Congo, occurring in evergreen rainforest at 200–500 m.

#### Local names.

Lukanga (*Hauzer 18*), mvouma (Fang, *Hladik 1689 part A, part C*), ngambo (Bakota, *Hladik 1689 part C*).

#### Additional specimens examined.

**GABON.** Nyanga: Mayombe bayaka, Tchibanga, région du Nyanga, 2 Feb 1915 (fl), *Le Testu 2006* (A, BM, MO—2 sheets, P).—Ogooué-Ivindo: SEGC, 11 Jan 2017 (buds), *Abernethy s. n.* (OWU); Ipassa, vieille route, Makokou, 500 m, 29 Feb 1972 (fl), *Hladik 1689 part A* (P); Ipassa, 10 km S of Makokou, 29 Feb 1972 (fl), *Hladik 1689 part C* (US); Station d’Etudes des Gorilles et Chimpanzés, forêt de l’Aeroport, 2 Feb 1993 (fl), *Tutin 80* (MO); Reserve de Lopé-Okanda, 0°25'S, 11°30'E, 20 Dec 1990 (tiny buds), *White [series 2] 285* (MO), Reserve de Lopé-Okanda, 0°25'S, 11°30'E, 10 Feb 1991 (fl), *White [series 2] 345* (MO); Lope Reserve, Pothos death site, Aug 1993 (fl), *White 960* (MO). **DEMOCRATIC REPUBLIC OF THE CONGO.** Kongo Central: Kisafu (Maduda), Oct 1951 (fr), *Hauzer 18* (BR).

In the herbarium this species is separable with difficulty from *X.quintasii* s. s. but is more readily distinguished in the field. The flush of leaves on new growth is white (L. White and K. Abernethy, personal communication), and in central Gabon the flowers appear mostly in February. The most striking quantitative difference is in the size of mature flowers, with the outer petals of *X.congolensis* sometimes exceeding 20 mm in length. There are, however, small differences that can be discerned in herbarium material. The leaves of *X.congolensis* tend to be proportionally narrower than those of *X.quintasii* and are usually glabrous; the decurrent ridges on the twigs are raised and persistent, the pedicels are relatively short, and the bracts are caducous well before anthesis. In the Lopé Reserve field site, both species are present and have maintained discrete phenologies over a period of many years (K. Abernethy and E. Bush, personal communication). Associates of *X.congolensis* mentioned by collectors are species of *Parinari*, *Treculia*, and *Pentadesma* (*Hauzer 18*), and understory Marantaceae (*White [series 2] 285*).

Boutique (1951b) expressed the opinion that *Xylopiacongolensis* was not necessarily conspecific with *X.quintasii*, calling attention to the smaller seeds and shorter aril of the type specimen. While the fruits and seeds on the type specimen of *X.congolensis* are immature, thus accounting for these differences, the specimen shows vegetative features that place it with a number of specimens from Gabon.

### 7. Xylopia quintasii

Taxon classificationPlantaeMagnolialesAnnonaceae

Engler & Diels, Monogr. afrik. Pflanzen-Fam. 6: 62. 1901.

BD05527E-251D-5B8E-AD11-890A3DE6DE3F

Figs 3C , 4C , 14A–F

Xylopia striata Engler, Bot. Jahrb. Syst. 34: 160. 1904. Type. CAMEROON. South Province, Bipindihof, Jan 1903 (fr), *G. A. Zenker 2663* (lectotype, here designated: B!; isolectotypes: BM! [000511005, right hand portion of sheet], K! [001096587], P!).
Xylopia lanepoolei Sprague & Hutchinson, Kew Bull. Misc. Inform. 1916: 160–161. 1916. Type. SIERRA LEONE. Western Area, Headquarters District, Heddles Farm, Apr 1914, *C. E. Lane-Poole 210* (lectotype, here designated: K! [000380211]; isolectotype: K, n. v. [spirit collection 15057.000]).
Polyalthia mayumbensis Exell, J. Bot. 64: Suppl. 4. 1926. Type. ANGOLA [“Portuguese Congo”]. Cabinda Province, Buco Zau, Mayumbe, 28 Nov 1916 (fl), *J. Gossweiler 6845* (holotype: BM! [000511084]; isotype: COI! [00004887]).

#### Type.

SAO TOME & PRINCIPE. “Insel St. Thomé, bei Angolares um 100 m. ü. d. M., Jan 1886, *F. Quintas 3* (lectotype, here designated: K! [000199059]; isolectotype: COI).”

#### Description.

***Tree*** up to 42 m tall, commonly 10–30 m, d.b.h. up to 75 cm, bole cylindrical, up to 37 m high, with narrow thin buttresses at the base, secondary branches horizontal, forming a small (3–5 m high) flat to conical crown; bark brown to reddish brown, scaly and peeling. ***Twigs*** light to dark brown, sometimes orange- or red-tinged, eventually light gray to brown, glabrous or sparsely pubescent, the hairs 0.1–0.2 mm long, soon glabrate, usually with bark exfoliating, sometimes marked with ridges decurrent from both sides of petiole base. ***Leaf*** with larger blades 6.0–11.9 cm long, 2.6–5.5 cm wide, subcoriaceous to chartaceous, discolorous with the lower surface often tan-colored, obovate to oblanceolate, occasionally elliptic, apex short-acuminate, the acumen 1.5–6.5 mm long, occasionally obtuse, rounded or emarginate, base cuneate and decurrent, glabrous adaxially, sparsely appressed-pubescent to glabrate abaxially; midrib plane to impressed adaxially, raised and somewhat keeled abaxially, secondary veins brochidodromous, 7–12 per side, diverging at 60–70° from the midrib, secondary veins slightly raised adaxially, raised abaxially, higher-order veins indistinct or occasionally slightly raised or impressed adaxially, usually forming a raised reticulum abaxially; petiole 2.5–6.5 mm long, shallowly canaliculate, sparsely pubescent to glabrate. ***Inflorescences*** axillary or from the axils of fallen leaves, 1–7-flowered, appressed-pubescent; peduncle 1 per axil, 2.6–2.8 mm long or pedicels arising directly from axil or both; pedicels 3–4 per peduncle, 6.5–8.5 mm long, 0.5–0.7 mm thick; bracts usually 3, sometimes 2 or 4, evenly spaced along length of pedicel, uppermost often persistent, but lower usually caducous, 1.2–1.5 mm long, lunate to semicircular, apex rounded; buds panduriform or narrowly oblong, apex obtuse. ***Sepals*** spreading at anthesis, free or only connate at very base, the bases sometimes slightly imbricate, 1.5–1.9 mm long, 1.9–2.4 mm wide, coriaceous, semicircular, triangular or ovate, apex acute to obtuse, pubescent abaxially. ***Petals*** pale green, white, or cream-colored *in vivo*; outer petals bent outward above the base at anthesis, 8–15 mm long, 1.8–2.8 mm wide at base, 1.5–1.8 mm wide at midpoint, coriaceous to slightly fleshy, ligulate, apex obtuse, puberulent except for glabrous patch at base adaxially, appressed-pubescent abaxially; inner petals erect at anthesis, 7.1–13.2 mm long, 0.7–1.5 mm wide at base, ca. 0.6 wide at midpoint, fleshy, linear, apex obtuse, base with differentiated fleshy or membranous margins, densely puberulent on both surfaces except for glabrous concavity and basal margins adaxially. ***Stamens*** 50–80; fertile stamens 1.3–1.8 mm long, oblong, apex of connective ca. 0.3 mm long, conical, slightly overhanging the anther thecae, long-papillate, anthers 8–10-locellate, filament 0.4–0.6 mm long; outer staminodes 1–1.3 mm long, broadly clavate to oblanceolate, apex conical; inner staminodes absent; staminal cone absent. ***Carpels*** 3–5; ovaries (0.8–) 1.2–1.6 mm long, ellipsoid, pubescent, stigmas discrete, not connivent, 0.3–0.7 mm long, clavate, glabrous except for fine setae at the apex. ***Torus*** flat, 1.4–1.9 mm in diameter. ***Fruit*** of up to 4 glabrate monocarps borne on a pedicel 8.3–19 mm long, 1.5–2.2 mm thick, glabrate; torus 2.4–4.7 mm in diameter, 1–3.5 mm high, depressed-globose. ***Monocarps*** with green exterior, tinged with brown, maroon, or purple, and green endocarp *in vivo*, 3.5–6.4 cm long, 0.7–1.0 cm wide, 0.6–0.9 cm thick, narrowly oblong, occasionally slightly falciform, torulose, apex obtuse to rostrate, the beak 1.5–4 mm long, base contracted into a more or less distinct stipe 6.5–11 mm long and 1.9–3.5 mm thick, obliquely striate, occasionally verrucose; pericarp 0.2–0.9 mm thick. ***Seeds*** up to 5, commonly 3, per monocarp, in a single row, parallel or slightly oblique to long axis, 10–12.5 mm long, 5.5–7 mm wide, 5–6 mm thick, ellipsoid, elliptic in cross-section, truncate at micropylar end, rounded and somewhat wedge-shaped at chalazal end, brown to reddish brown, smooth, shiny, raphe/antiraphe not or only faintly evident, micropylar scar 1.8–3.5 mm long, 2.5–3 mm wide, roughly circular or transversely elliptic; sarcotesta absent; aril red to deep orange *in vivo*, straw-colored when dried, fimbriate, extending the length of the seed, membranous, smooth.

#### Phenology.

Specimens with flowers have been collected from September to June and with fruits from March to October and in December. In West Africa (Sierra Leone to Nigeria), flowering begins in December and continues until May, while, in central Africa (Cameroon and eastward and southward), flowering begins in September and lasts until February. The fruiting phenology is also offset slightly, with fruits collected in western Africa in March, May, June, July, and October, and fruits in central Africa collected from April to August, and in October and December. The West African pattern is confirmed by Hall and Swaine (1981), who give the flowering period in southern Ghana as December to March and the fruiting period as July to October. In Sierra Leone, flowering is reported to occur from February to April, with ripe fruits following from June to September (Savill and Fox 1967).

#### Distribution

(Fig. 15). *Xylopiaquintasii* is distributed from Sierra Leone along the coast eastward to Ghana, resuming in southern Nigeria and extending eastward to the southern Central African Republic and southward across Gabon and the westernmost Democratic Republic of the Congo into the Cabinda Province of Angola. It occurs in primary lowland rainforest on a variety of soil types, occasionally in secondary forest or rarely in inundated forest, at elevations of 0–1000 m, although it is most common below 200 m.

#### Local names.

Brala (ébrié, Aubréville 1959), gbay (*Cooper 222*), gbay-dee (*Cooper 372*), elo (*Service forestier 458*), lucanga (*Gossweiler 6845*), melasomba (Lissongo, *Tisserant 527*), aghako (Benin, *Kennedy 1319*), mbonba (Yaounde, *Letouzey 5510*), muomba (*Wilde & Wilde-Duyfjes 1320*), mvoma (Pahouin, *Fleury 26594*), mvǔ’ma (Fang, *Tessmann 760*, Tessmann 1913), nkala (Lissongo, *Tisserant 1786*), nzange (Ngwaka, *Evrard 813*; Lissongo, *Tisserant 1786*), obaa (Akan, Hall and Swaine 1981), opalifon (Yoruba), kpaini, bajineh, or bajmeh (Mende, *Aylmer 202*), kpaini (*Lane-Poole 210*). A number of these common names are also given to other species of *Xylopia*. The label of *Deighton 4153*, from a collection made in Sierra Leone in 1946, gives these additional details concerning common names applied to *Xylopiaquintasii* there: “Kpa-hinei (Mende), the male Kpa tree. The Kpa (definite Kpei) is *Tylostemonmannii*. Forestry officers have usually given Kpaini as the Mende name for *Xylopiaquintasii*, but I believe my spelling is more correct.” Savill and Fox (1967) gave both the spellings “kpa-hinei” and “kpainii” and added that the same common name is also applied to *Xylopiaacutiflora*.

#### Representative specimens.

**GUINEA.** Macenta+Beyla Prefectures, Simandou Range, S of Pic de Fon, 8°28'51"N, 8°54'29"W, 765 m, 15 Sep 2008 (yg fr), *van der Burgt 1305* (K). **SIERRA LEONE**. Kabala, Mt. Loma, Kondembaya, 4 Feb 1966 (st), *Adam 23553* (MO); Kabala, Mt. Loma, Mousouia, 4 Feb 1966 (fl), *Adam 23647* (MO); Railway Hill, 12 Mar 1918 (fl), *Aylmer 202* (K); Freetown, 20 March 1914 (fl), *Dalziel 956* (K); Njala, 12 Feb 1946 (fl), *Deighton 4153* (K); forêt sommet P?eraukouko (Loma), 800 m, 9 Feb 1966 (fl), *Jaeger 9283* (K); Trigpoint III Kesewe, 8 Apr 1913 (fl), *Lane-Poole 123* (communicated by M. Bañi?) (K); Northern Region, Tankolili District, Sula Mountains South, E of village Farangbaya near Bumbuna, forest patch on higher slopes of Simbili, 8°58'30"N, 11°41'21"W, 820 m, 26 Feb 2010 (fl), *Sesay 30* (K); without definite locality, *Smeathman s. n.* (BM). **LIBERIA.** Near Firestone Plantations along Dukwia ["Dukwai"] R., 23 Feb 1929 (fl), *Cooper 222* (A, BM, F, GH, K, NY, US, YF), Dukwia ["Dukwai"] River, Monrovia, 1929 (st), *Cooper 337* (A, BM, F, GH, K, NY, PH, US, YF), 1929 (st), *Cooper 372* (A, BM, F, GH, K, MICH, NY, PH, US, YF), 24 May 1929 (yg fr), *Cooper 464* (A, BM, F, GH, K, NY, PH, US, YF); National Forest 18 mi N of Tapeta, *Voorhoeve 144a* (WAG); Nimba Mts., *Voorhoeve 910* (WAG). **IVORY COAST.** Région d’Anyama, forêt du Téké, *Aké Assi s. n.* (MO); le Banco, Jun 1932 (fr), *Aubréville 1345* (MO); Without definite locality [boqueteaux des savanes de Bingerville (Aubréville 1959)], s. d. (fr), *Aubréville 1943* (A, P), s. d. (fr), *Aubréville1945* (A); Duékoué—Buyo, 4 km E de Pinhou, 26 Mar 1969 (fl), *Bamps 2260* (K); Bouroukrou, 20 Dec 1906–20 Jan 1907 (fl), *Chevalier 16118 (bois)* (K, MO–-3 sheets, P); Dakpadou-Sago, 5°06'N, 5°58'W, 29 Mar 1968 (fl), *Geerling & Bokdam 2314* (K, MO, WAG); San Pedro, Nero River near Grand Berybery, 4°40'N, 6°53'W, 5 m, 14 Dec 1997 (fl), *Jongkind et al. 4218* (OWU); Abidjan, Banco Forest Reserve, c. 5°25'N, 4°03'W, 22 Dec 1972 (fl), *de Koning 985* (OWU); Abidjan, Banco Forest Reserve, Route Martineau, 5°23'N, 4°03'W, 28 Dec 1974 (fr), *de Koning 4009* (OWU); forêt de l’Abouabou, between Abidjan and Grand Bassam, 8 Jan 1959 (fl), *Leeuwenberg 2406* (K, MO, P, WAG); Banco, s. d. (yg fr), *Service forestier 458* (NY, P). **GHANA.** Tanosu, W. frontier, 18 Sep 1912 (fr), *Brent 389* (K—2 sheets); Prov. Western, Enchi Dist., Tano Anwia F. R., Jan 1952 (fl), *Andoh 5610* (BM); Fure Forest Reserve near Prestea, 15 Dec 1971 (fl), *Deaw Sp 463* (MO, NY, RSA); Eastern Region, District Oda, Kade (University College Farm), 20 Jan 1958 (fl), *Enti FH 6881* (K); Sikamang nr. Obuasi, 6 Mar 1975 (fl), *Hall & Abbiw GC45145* (MO); Eastern, Atewa Range Forest Reserve, along the Old Geological Survey road, 06°14'06"N, 0°33'0"W, 19 Oct 1994 (fr), *Jongkind et al. 1783* (MO); Eastern, Kade Agricultural Research Station, 11 km N of Kade, 6°08'28"N, 00°53'56"W, 200 m, 2 Dec 1996 (fl), *Schmidt et al. 2267* (MO); Adeambra, 4 May 1923 (fl, yg fr), *Vigne 856* (K). **NIGERIA.** Benin Province, Iyekuselu District, 8 Dec 1961 (fl), *Daramola FHI 45673* (K); Ogun, Ijebu East, Omo forest reserve, 30 Aug 1994 (st), *Daramola 434* (F, MO); Cross River State, 25 km N of Oban on road to Ekang, near Cameroun border, 28 Jun 1981 (fr), *Gentry & Pilz 32875* (K, MO, WAG); Southwestern Nigeria, 2 mi S of Etemi fishing village, H. F. by the Omo River, 20 Mar 1946 (fl, fr), *Jones & Onochie FHI 17012* (K); Ijebu-Ode Province, Akilla, plantations of *Nauclea*, etc., 12 Nov 1960 (fl), *Keay FHI 37826* (K); S. Nigeria, Sapoba, 1930 (fl), *Kennedy 702* (K—4 sheets); Mar 1930 (fr), *Kennedy 1319* (K); S. Nigeria, without definite locality, s. d. (fr), *Kennedy 1543* (A, BM, MO, US), s. d. (fl), *Kennedy 1662* (A, BM, K, PR, US, YF), s. d. (fl), *1987* (A, K); Bendel State, forest about 15 km SW of Ekenwan, 6°01'N, 5°18'E, 28 Mar 1977 (fl, fr), *Leeuwenberg 11276* (WAG—2 sheets); Prov. Ondo, Dist. Ondo, 1.5 miles from Ore on Agbabu road, 22 Sep 1965 (buds), *Okafor & Latilo 057285* (MO); Prov. Ijebu, Shasha Forest Reserve, 26 Feb 1935 (st), *Richards [Ross?] 3159* (BM), 12 Jun 1935 (fl), *Richards 3334* (BM, F, MO, NY—2 sheets), 2 Sep 1935 (fr), *3434* (BM, MO—3 sheets, NY); Prov. Ijebu, Shasha Forest Reserve, Akilla, 8 Mar 1935 (fl, yg fr), *Ross 68* (BM, MO); S. Nigeria, Oban District, 30 Jan 1912 (fl), *Talbot & Talbot 1302* (BM, K, NY). **SAO TOME & PRINCIPE.** S. Tomé, Angolares, Agoa Gombela, 100 m, Jan 1886 (st), *Quintas 1083* (BM). **CAMEROON.** Bipinde, 20–23 Jun 1918 (fl), *Annet 319* (OWU, P); 20 km from Kribi, Lolodorf road, 3°00'N, 10°03'E, 9 Jun 1969 (fr), *Bos 4773* (K, M, MO, P, WAG—2 sheets); 27 km SW of Bertoua, near Toungrélo, 5 Jan 1962 (fl), *Breteler et al. 2398* (A, FI-T, K, M, P, WAG—2 sheets); Southwest Province, near Ngusi village, N of Nyassosso, 4°53'N, 9°42'E, 26 Apr 1986 (fr), *Etuge & Thomas 56* (B, K, MO, NY, WAG); bassin du Mungo, village de Mayouka près de la gare de Mujuka, au km 59 du chemin de fer du Nord dans la forêt de la Dzigo, Jul 1917 (fr), *Fleury 33517 [Bois no. 23*] (OWU, P–2 sheets); without definite locality, Service forestier du Cameroun (Yaoundé), 1935 (fl), *Foury 36* (OWU); sud Cameroun, *Hallé 4240* (WAG), *4247* (WAG); South West Province, Ndian Division, Korup National Park, Korup Forest Dynamics Plot, 29 May 1999 (fr), *Kenfack 1187* (MO); 26 km au SSW de Koso (village situé à 60 km au SSW de Batouri, 27 Jul 1963 (fr), *Letouzey 5510* (K—2 sheets); Piste Sanchou-Bale, 18 km SSW Dschang, 26 Nov 1974 (fl), *Letouzey 13327* (K, MO, P. WAG); Bipinde-Ebolowa, Dec 1913 (fl), *Mildbraed 7613* (K); südlich des Sanaga zwischen Jaunde und Dengdeng unweit der Vereinigung von Lom (Sanaga) und Djerem, etwa 125 km NO Jaunde, Feb 1914 (st), *Mildbraed 8294* (K); Prov. Southwest, mile 12 Mamfe road between Kumba and Baduma, 4°45'N, 9°29'E, 4 Oct 1986 (fl), *Nemba & Thomas 293* (GH, MO, P, WAG); without definite locality, *SRFK 1913* (P); hill facing the village of N’Kolandom, 2°48'N, 11°10'E, 3 Jan 1975 (fl), *de Wilde 7871* (K, M, MO, NY); hill roughly between N’Kolandom and N’Koemvone, 2°48'N, 11°09'E, 9 Jan 1975 (fl), *de Wilde 7889* (K, MO, P); ca. 50 km NW of Eséka, W of Yaoundé, on opposite of [sic] the Kelè-river, 23 Nov 1963 (fl), *de Wilde & de Wilde-Duyfjes 1320* (K, P, WAG—2 sheets); Bipinde, 1900 (fr), *Zenker 2080* (B, BM, K, L, M, MO, P, WU), Oct 1913 (fl), *Zenker 2094* [*408*?] (B, GH, M, MO, P, US, WAG); Bipindihof, Dec 1902 (fl), *Zenker 2655* (B, BM [mounted on same sheet with 2663], K, L, M, P—2 sheets, WAG, WU); Bipinde, 1913 (fl), *Zenker 4738* (B, BM, K, M, MO, P, PR, US); Mimfia, May 1914 (yg fr), *Zenker 2095* [*580*?] (GH, M, MO, P, U, US, WAG), Jun 1913 (fl), *Zenker s. n.* [*359*?] (B, GH, M, MO, P, US); Hermanshof, Beguiberge, Sep 1910 (buds), *Zenker 4096* (BM, F, K, L, M, MO, P, PR, WU). **CENTRAL AFRICAN REPUBLIC.** Région de Mbaïki, Station Centrale de Boukoko, *Tisserant 527* (BM, K, P), 19 Jun 1950 (fr), *Tisserant 1786* (BM, P). **EQUATORIAL GUINEA.** [Locality not legible], *Tessmann 760* (K). **GABON.** Estuaire: S of Ekouk, 0°06'S, 10°20'E, 3 Nov 1983 (buds, fr), *Louis et al. 350* (K, U, WAG); rivière Ayemé, 0°05'S, 9°55'E, 24 Jan 1991 (fr), *Louis 3304* (MO); S of Estuaire du Gabon along Remboué River, 0°00'N, 9°50'E, 23 Oct 1991 (fr), *McPherson 15432* (MO, WAG); région de Sibang, *Heitz 3* (P); Mission Nyonyie, Plaine Zabor, 4 Jul 1990 (fr), *Wilks et al. 2107* (MO).—Moyen-Ogooué: Mabounié, 00°50'00"S, 010°27'00"E, 30 Oct 2012 (buds), *Boupoya 807* (MO); environs du lac Zilé, près d’Atsié, sur l’Ogooué circonscription de Lambaréné, 12 Aug 1912 (st), *Fleury 26594* (MO, P).—Ngounié: Mabounié, along bank of Ngounié River, 00°48'43"S, 010°30'03"E, 12 Oct 2012 (fl, fr), *Stévart et al. 4642* (MO); about 22 km along a track in a northern direction from Doussala, 2°12'S, 10°36'E, 4 Dec 1986 (fl), *Wilde et al. 9156* (K, P, WAG).—Nyanga: Doudou Mountains, ca. 35 km SW of Doussala, chantier CEB, 2°32'S, 10°30'E, 27 Aug 1985 (fr), *Reitsma & Reitsma 1423* (MO, NY—2 sheets, RSA, WAG).—Ogooué-Ivindo: western border of Lopé-Okanda Reserve, along roads S of SEEF chantier, 0°25'S, 11°30'E, 28 December 1991 (fl), *McPherson 15698* (MO, WAG); Lopé-reserve, chantier SOFORGA, 0°30'S, 11°33'E, 24 Jun 1986 (fr), *Reitsma & Reitsma 2342* (MO, NY, RSA, U, WAG); Lopé Reserve, 0°15'S, 11°40'E, Aug 1991 (fr), *SEGC 444* (MO); Reserve de Lopé-Okanda, 0°25'S, 11°30'E, 8 Dec 1990 (fl), *White [series 2] 239* (MO); 25 km NNE de Koumameyong, 0°25'N, 11°55'E, 18 May 1987 (yg fr), *Wilks 1536* (GH, MO).—Ogooué-Maritime: Rabi-Kounga, halfway down road to well Rab-71, 1°54'S, 9°50'E, 30 m, 11 Dec 1995 (fl), *van Bergen 153* (MO, WAG); Toucan, ca. 01°47'S, 09°53'E, 1 Jun 2002 (buds), *Bourobou Bourobou et al. 655* (MO, WAG); Petit Loango, 2°20.65'S, 09°36.82'E, 1 Oct 2002 (fr), *Bourobou Bourobou et al. 930* (K, MO, WAG).—Woleu-ntem: Crystal Mountains, Tchimbélé, ca. 0°38'N, 10°23'E, 600 m, 18 Nov 2001 (st), *Breteler 15795* (MO); la circonscription du Woleu-Ntem, Acourenzoc, région entre Ogooué et Cameroun, 29 December 1933 (fl), *Le Testu 9438* (BM, K, P).—Province unknown: Kouilou inférieur, [reçu le 9 Mar 1925] (fl), *Sargos 242* (A, P). **DEMOCRATIC REPUBLIC OF THE CONGO.** Kongo Central: INEAC Luki Mayumbe (Bas-Congo), s. d. (fr), *Hombert 475* (BR); INEAC, Luki, Mayambe, 1957 (fr), *Mahieu 206* (WAG).—Sud-Ubangi: Boywaza, galerie de la Lua-Vindu [Lua Vindu is ca. 3°30'N, 19°E], 28 Apr 1955 (fr), *Evrard 813* (BR).

*Xylopiaquintasii* bears paddle-shaped obovate to oblanceolate leaves, small blunt flower buds, and a small number of striate torulose 1–5-seeded monocarps. The species is remarkably uniform over its broad distribution, varying little in leaf, flower or fruit morphology. The most notable variations are the larger leaves seen in some sterile specimens, and the occasional longer petals, e.g. in *Leeuwenberg 11276* from Nigeria the outer petals are ca. 13 mm and the inner petals ca. 11 mm in length. Significantly, the taxonomic synonyms listed above were never compared to *X.quintasii* when they were published, but instead to other species: *X.striata* was contrasted with “*X.acutifolia*” [*X.acutiflora*], *X.lanepoolei* with *X.parviflora* [*X.longipetala*], and *Polyalthiamayumbensis* indirectly compared to other African species then placed in *Polyalthia*, both of which had unisexual flowers, by the observation “Apparently a species with no unisexual flowers.” Pellegrin (1949) seems to have been the first author to recognize the similarity of these species, placing *X.striata*, *X.lanepoolei*, *X.congolensis*, and *X.aurantiiodora* as taxonomic synonyms of *X.quintasii*, while Paiva (1966) first placed *Polyalthiamayumbensis* as a synonym of the species.

*Xylopiaquintasii* resembles *X.aurantiiodora*, sharing with it the characteristic features of sect. Ancistropetala, i.e. absence of the staminal cone, fleshy basal margins of the inner petals, and the fimbriate aril surrounding the seed. *Xylopiaquintasii* is typically a tall tree, however, with a straight bole and compact crown with crowded horizontal branches, while *X.aurantiiodora* is a shrub or small tree, reaching eventually a height of up to 10 m. The two differ in other respects as well: the leaf of *X.quintasii* is usually oblanceolate to obovate and short-acuminate, while that of *X.aurantiiodora* is characteristically elliptic and obtuse to rounded at the apex; the inner petals of *X.quintasii* lack the overhanging tooth present in *X.aurantiiodora*; the styles in *X.quintasii* are distinctly shorter than the ovary, while they are subequal in length in *X.aurantiiodora*; the monocarps of *X.quintasii* are usually tipped by a blunt curved beak, while those of *X.aurantiiodora* are distinctly acute; and the seeds of *X.quintasii* are rounded at the apex while those of *X.aurantiiodora* are distinctly pointed. The two species also differ in habitat: *Xylopiaquintasii* occurs most frequently in upland forest, while *X.aurantiiodora* is typically a species of inundated forest. *Xylopiaquintasii* is the more western of the two species, but the two overlap in range in Cameroon, the Central African Republic, the western part of the Democratic Republic of the Congo, and the Cabinda Province of Angola.

Recent fieldwork in Gabon by K. Abernethy and collaborators has revealed that the red or purple color of the leaf flush distinguishes *X.quintasii* from the similar *X.congolensis*, the latter with a white leaf flush, growing in the same field site. This color difference is not reliably visible in dried specimens, and is seldom reported on collection labels but has been reported for two collections of *X.quintasii* from Nigeria (*Okafor & Latilo FHI 57285*, *Ross 68*), and two from Gabon (*Breteler & Jongkind 10470*, *Breteler 15795*). Other differences between the two species are described under *X.congolensis*.

Collectors often call attention to the strong fragrance of the flowers–“pungently scented” (*Gossweiler 6845*), “sweetly fragrant” (*Schmidt et al. 2267*), “heavy sweet scent” (*de Wilde 7889*)–but nothing is known of pollinators or pollination in this species. Monkeys of several species have been reported as dispersers of the seeds in both Ivory Coast (Eckardt and Zuberühler 2004, Koné et al. 2008) and Cameroon (Mitani 1999, Poulsen et al. 2001, Wang 2008). In Ivory Coast, the seeds were seen to be spat out by the monkeys (Koné et al. 2008) while in Cameroon they were recovered from gray-cheeked mangabey feces (Poulsen et al. 2001).

*Xylopiaquintasii* is reported to be an important forest species in southern Ghana (Hall and Swaine 1981). Species associated with it in upland forest include *Khaya* sp., *Lophiraalata*, *Swartzia* sp., and *Terminalia superba*, and, in inundated forest, *Pandanuscandelabrum* and *Marantochloapurpurea*. Savill and Fox (1967) describe it as a lower canopy tree in *Heritierautilis*-*Cynometraleonensis* forest of the Gola Forest, Sierra Leone, and that it can be found in secondary forests of various stages. Tessmann (1913) reported that the wood of the species was used to make handles for weapons and tools by the Fang people in Equatorial Guinea.

Engler and Diels, in the protologue of *Xylopiaquintasii*, cited sheets of the type from both K and COI. We examined the K sheet, which corresponds well to the protologue and is annotated by Engler and have chosen it as the lectotype. A specimen at BM with slightly different information (Angolares, Agoa Gombela, 100 m, Jan 1886, *Quintas 1083*) may represent an additional isolectotype, as it has the same broader than normal leaves found on the K lectotype. Sprague and Hutchinson (1916), in their description of *Xylopialanepoolei*, cited two specimens, *Lane-Poole 210* and *Dalziel 956*, but did not designate one as the type. The sheet of *Lane-Poole 210* has slightly better flower material, including a packet containing dissected flower parts, and has been designated as the lectotype.

### III. Xylopia Section Xylopia

Taxon classificationPlantaeMagnolialesAnnonaceae

34CF72EB-0B35-5E78-AA4D-AB2D3740BCD8

Xylopia Section Habzelia Engler & Diels, Monogr. afrik. Pflanzen-Fam. 6: 58. 1901. Type: Xylopiaaethiopica (Dunal) A. Richard (lectotype designated in Stull et al. 2017, p. 221).

#### Type.

*Xylopiamuricata* Linnaeus.

#### Description.

Nodes with a branch from a single axillary bud; outer and inner petals linear, similar in length, in African species; inner petal margins curved inward at base but of uniform texture, lacking differentiated fleshy basal margins; anther connectives shieldlike at apex, overhanging the anther thecae; staminal cone present, completely concealing the ovaries, rim even; carpels (11–14) 45–50 in African species, the stigmas connivent, smooth; aril bilobed, fleshy; seed coat smooth, sarcotesta present or absent (absent in African species).

#### Notes.

All species of *Xylopia* occurring in the Mascarene Islands and in tropical America belong to this section. The section is represented in Africa by a single species, *X.aethiopica*, in Madagascar by four species, on the Mascarene Islands by three species, and in tropical America by about 60 species. The staminal cone is well-developed in all species of section Xylopia, as is the bilobed fleshy aril.

### 8. Xylopia aethiopica

Taxon classificationPlantaeMagnolialesAnnonaceae

(Dunal) A. Richard, Hist. phys. Cuba, Pl. vasc. 53. 1841 [“1845”].

0A53FBD6-62DC-50C0-A1A9-5DD171C09C7E

**Figure 16.**
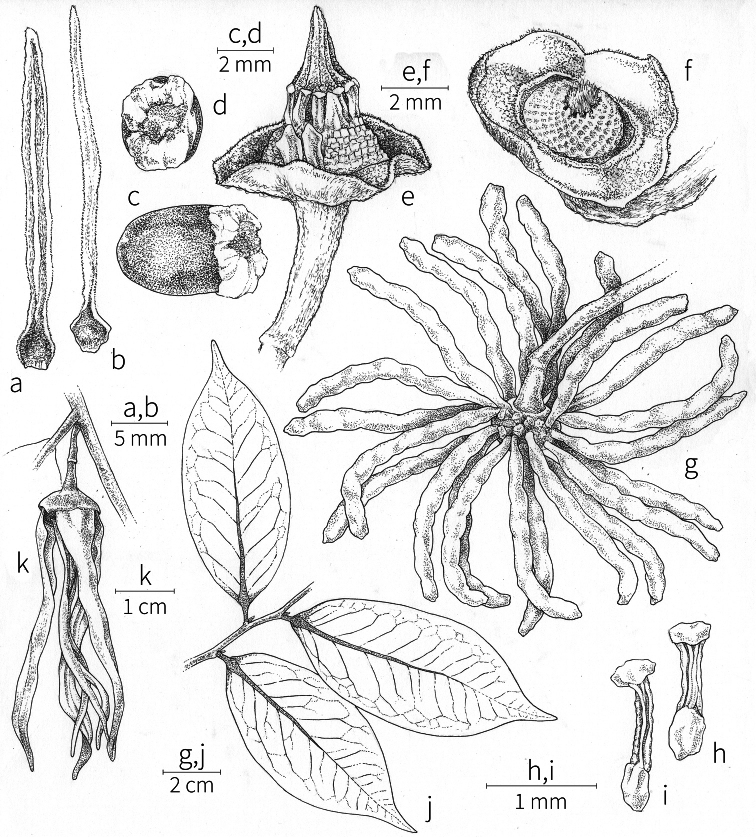
*Xylopiaaethiopica*. **A** Outer petal, adaxial view **B** Inner petal, adaxial view **C** Seed, side view **D** Seed, view of micropylar end **E** Flower with petals and most stamens removed to show gynoecium, inner staminodes, staminal cone, two outer staminodes, and sepals **F** Staminal cone **G** Fruit. **H, I** Stamens, abaxial view **J** Leaves **K** Flower, side view. **A, B, E, H, I** from *McPherson 15168* (MO) **C, D** from *Wilks WIL 922* (WAG) **F, G, K** from *Wieringa et al. 3031* (WAG), **J** from *Nkongmeneck 399* (P).

Figs 2 , 3D , 4B , 16

Unona aethiopica Dunal, Monogr. Anonac. 113–114. Aug–Nov 1817.
Uvaria aethiopica (Dunal) A. Richard, Fl. Senegamb. Tent. 1: 9. 1831.
Anona aethiopica (Dunal) Steudel, Nomencl. Bot., ed. 2, 2: 737. 1841.
Xylopicrum aethiopicum (Dunal) Kuntze, Revis. gen. pl. 1: 8. 1891. Type. SIERRA LEONE. Without definite locality, s. d., *H. Smeathman s. n.* (lectotype, designated by Verdcourt (1971b, p. 77): G!, secondary lectotype, designated here: G-DC! [00201441 on 2 sheets]; isolectotypes: BM! [000510763, branch on right-hand side of sheet]], FI-W! [005603]).
Xylopia eminii Engler, Pflanzenw. Ost-Afrikas C: 179. 1895. Type. UGANDA. Western Province, Bugoma, Ins. Ssese [“Sesse”], 15 Dec 1890, *F. L. Stuhlmann 1233* (holotype: B! [100153132]).
Xylopia dekeyzeriana De Wildeman, Ann. Mus. Congo, Sér. 5, Bot. 1: 43 + t. 19. 1903. Type. DEMOCRATIC REPUBLIC OF THE CONGO [“Belgian Congo”]. Kongo Central Province, Sanda, 1902, *M. L. van Houtte in J. Gillet 2258* (lectotype, here designated: BR!; isolectotypes: BR! [8824257, 8824264]).
Xylopia gilletii De Wildeman, Ann. Mus. Congo, Sér. 5, Bot. 1: 42. 1903. Type. DEMOCRATIC REPUBLIC OF THE CONGO [“Belgian Congo”]. Kongo Central Province, Inkisi-Kisantu [“Kisantu”], 1899, *J. Gillet 207* (holotype: BR!).

#### Description.

***Tree*** up to 46 m tall, commonly 15–30 m, d.b.h. 30–58 cm, bole slender, base of trunk with plank buttresses 0.5–3 m high extending 0.9 m from trunk, forming a relatively small dense crown; bark gray with shallow longitudinal fissures. ***Twigs*** brown to dark gray, glabrous or sparsely and finely pubescent, the hairs 0.1–0.4 mm long, but soon blackish gray, glabrate. ***Leaf*** with larger blades 7.3–16.3 cm long, 2.1–6.6 cm wide, subcoriaceous, slightly discolorous, shiny adaxially and dull and often paler abaxially, lanceolate-ovate to elliptic, occasionally oblong, narrowly elliptic, oblanceolate, or rarely ovate, apex acuminate, the acumen 6–20 mm long and sometimes falcate, rarely obtuse, base cuneate to broadly cuneate and short-decurrent on the petiole, glabrous adaxially, finely appressed-pubescent to glabrate abaxially; midrib raised or plane at the base but becoming plane to impressed toward the leaf apex adaxially, often drying dark red toward the base, raised abaxially, secondary veins indistinctly and irregularly brochidodromous, 9–13 per side, diverging at (35–) 45–60° from the midrib, somewhat arcuate, these and higher-order veins slightly raised on both surfaces, sometimes forming a visible reticulum abaxially; petiole 4–9 mm long, somewhat flattened or shallowly canaliculate, transversely wrinkled, sparsely pubescent to glabrate. ***Inflorescences*** axillary or from the axils of fallen leaves, 1–5 (–7)-flowered, pedunculate, appressed-pubescent; peduncle 1.5–2.8 (-4) mm long; pedicels 2–4 per peduncle, 4.5–15 mm long, 0.8–1.4 mm thick; bracts 1–2, typically one to either side of pedicel midpoint, caducous, 1.7–2.4 mm long, 2.5–2.9 mm wide, quadrate, semicircular, or ovate, often splitting down the middle, apex obtuse, rounded, or truncate; buds linear-oblong, rarely oblong, apex obtuse to acute, often curved. ***Sepals*** erect to slightly spreading at anthesis, 1/4–2/3-connate, 2.2–4.1 mm long, (3.2–) 4.4–6 mm wide, coriaceous, semicircular to broadly triangular, apex acute to rounded, sparsely pubescent to glabrate abaxially. ***Petals*** greenish white to yellow, the inner petals sometimes with a tinge of purple at the base *in vivo*; outer petals spreading but with the apices incurved at anthesis, (11.6–20.9) 28–64 mm long, 3.8–6 mm wide at base, 2.7–3.7 mm wide at midpoint, coriaceous, linear, apex obtuse, longitudinally concave adaxially up to ca. 5 mm from apex, glabrous at base but otherwise densely puberulent adaxially, appressed-pubescent abaxially; inner petals spreading but with the apices incurved at anthesis, (18.7–19.5) 33–51 mm long, 3.6–5.0 mm wide at base, 0.8–2.0 mm wide at midpoint, coriaceous, linear, apex acute, base with undifferentiated margin, densely puberulent adaxially, the hairs becoming sparser toward base, but then densely pubescent within the basal concavity and glabrous again at very base, puberulent except at base abaxially. ***Stamens*** 140–300; fertile stamens 1.2–1.7 mm long, oblong, apex of connective 0.2–0.5 mm high, dome-shaped, bluntly conical, or somewhat flattened, overhanging anther thecae, pubescent to glabrous, anthers 6–10-locellate, filament 0.3–0.5 mm long; outer staminodes 1.4–1.7 mm long, oblong, apex acute to truncate; inner staminodes 1.1–1.5 mm long, narrowly oblong, apex rounded; staminal cone (2.2–2.4) 2.9–4.0 mm in diameter, 1.1–1.5 mm high, completely concealing the ovaries, rim even. ***Carpels*** (11–14) 45–50; ovaries 1.4–2 mm long, narrowly oblong, densely pubescent; stigmas connivent, 3.5–4.7 mm long, linear, warty, glabrous to pubescent. ***Torus*** concave beneath the ovaries but otherwise flat, 2.6–4.6 mm in diameter. ***Fruit*** of up to 36 glabrate monocarps borne on a pedicel 10–18 mm long, 2–6 mm thick, glabrate; torus 7–20 mm in diameter, 4–10 mm high, depressed-globose. ***Monocarps*** with a green exterior and a pink to pale red endocarp *in vivo*, 3.2–8.2 cm long, 0.3–0.8 cm wide and thick, linear-cylindrical to narrowly oblong, weakly torulose, apex formed into a blunt beak 1.5–3.5 mm long, base slightly narrowed or contracted into a discernible stipe (1.5–) 3–8 mm long and 1.7–3.5 mm thick, strongly and obliquely wrinkled, verrucose; pericarp 0.5–0.8 mm thick. ***Seeds*** 4–12 per monocarp, in a single row, lying oblique to nearly parallel to long axis, 5.0–6.0 mm long, 3.4–3.7 mm wide, 2.5–3.1 mm thick, ellipsoid, elliptic to nearly circular in cross-section, obliquely truncate at micropylar end, rounded at chalazal end, dark brown to black, smooth, shiny, raphe/antiraphe slightly raised, micropylar scar minute; sarcotesta absent; aril pale yellow to orange *in vivo*, white to dull yellow when dried, bilobed, the lobes 1.5–1.9 mm long, 1.9–2.8 mm wide, fleshy, smooth.

#### Phenology.

Phenology varies with geography. From Senegal to Nigeria, specimens with flowers have been collected from March to December and with fruits from all months of the year except September and November. From central Africa, specimens with flowers have been collected in all months of the year except May, and with fruits from every month of the year. From the eastern and southern parts of the range, specimens with flowers have been collected in March, May, and from September to December, and with fruits in March and from June to January.

#### Distribution

**Figure 17.**
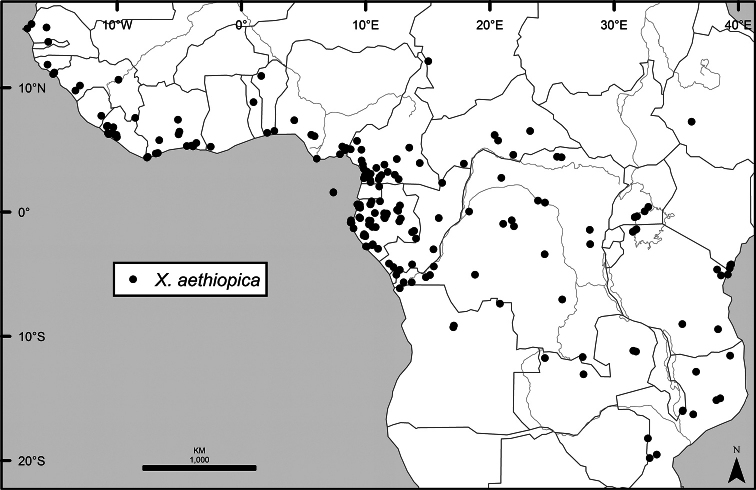
Distribution of *Xylopiaaethiopica*. Bolder lines represent country borders, fainter lines lakes and major rivers.

(Fig. 17). *Xylopiaaethiopica* has the widest distribution of any African species of *Xylopia*, occurring in moist forests from Senegal eastward to southwestern Ethiopia and then south to northern Angola, eastern Zimbabwe, and Mozambique. It also occurs on the Gulf of Guinea islands of Bioko and Principe. *Xylopiaaethiopica* grows in a wide range of habitats, including primary forest, secondary forest, upland forest, inundated forest, and lower montane forest, from near sea level up to 1600 meters.

#### Local names.

Békala (Kelai, *Le Testu 2178*), luvinda (*Gossweiler 9060*), dodo (Ivory Coast, *Thoiré 85*), eru (Yoruba, *Forestry Department, Benin City 194*), macua (*Gossweiler 8972*), mgana (Bayaka, *Le Testu 1877*), msaou (Chilunda, *Milne-Redhead 2883*), muengeve (*Harder et al. 2126*), mughâna (Echira, Pounou, Vili ou la legounyé, *Le Testu 2178*), mwawia (Tanzania, *Johnson & Murray 1944*), mweya (Kiluba de Kabongo, *Schmitz 5764*), n’cana (Angola, *Gossweiler 7845*), n’gani (Dioula, *Ambe 186*), nkana (Havili, *Le Testu 1877*, Vile eu Loango, *Le Testu 2178*), nsagalane (Luganda, *Eggeling 1524*), ojă,ṅ [name would be approximately rendered ‘oyang”] (Tessmann 1913), oyang (*Carvalho 4751*), oukalla (Pahouin, *Fleury 26582*), ogana (Gallois, *Fleury 26582*), okala (*Wilks 922*, Pahouin, *Le Testu 2178*), pedjericou (Fon, *Laffitte 92*), sange (Angola, *Welwitsch 765*), sengi (Aka, *Carroll 1077*), ughâ (oghâ?) (Itsogho, Viya, *Le Testu 2178*), unien (Benin, *Forestry Department, Benin City 194*, Johnson and Johnson 1974). Additional names reported in the research literature for this species include hwentea (Ghana, Aburi and Kondonuru Regions, Agbovie et al. 2002) or its variant hwentia (Ghana, Konning et al. 2004, Abbiw 1990; a Twi name meaning “slender nose,” referring to the shape of the fruits, according to Burkill 1985), kimba (Nigeria, Barminas et al. 1999, Abubakar et al. 2007), mchofu (Tanzania, World Agroforestry Centre 2017) and spais tik (Sierra Leone, Cline-Cole 1987). Additional common names for the species in West Africa are catalogued in Burkill (1985).

#### Representative specimens.

**SENEGAL.** Rufisque, Sangalkam, 29 Jan 1948 (st), *Adam 417* (MO, P); Presqu’ile du Cap Vert, Sangalkam, *Berhaut 1035* (P); Niayes, Dec 1899 (fl, fr), *Chevalier 2545* (K, P); D. Pierre du Yolofs, 1837 (fl), *Heudelot 566* (P); Senegambie, 1824–1829, *Perrottet 9* (P); without definite locality, *Perrot[t]et s. n.* (BM); Mboro niaye degradée au bord de la route neuve des Niayes, 9 Mar 1965 (yg fr), *Raynal 13562* (K, P). **GAMBIA.** Without definite locality, *Ingram s. n.*, comm. Whiteley (K-000354675). **GUINEA BISSAU.** Bissau, Brene, 31 Jan 1945 (yg fr), *Explorações Botânicas 1717* (B, K, WAG) [=*1709* at B, FI-T, P]; Bissau, Prabis, 22 Feb 1945 (yg fr), *Explorações Botânicas 1809* (BR, K, P, WAG); Maolina de Cantankez, 11°13'312N, 15°02'503W, 12 m, 14 Nov 1995 (fl), *Malaisse & Claes 14911* (BR); Cacine, 30 Jan 1954, *d’Orey 264* (K). **GUINEA.** Kouria, 22 Sep 1905 (fl), *Caille 14771* (P); Kouria, Bourkonkourí, 16 Nov 1905 (fl), *Caille 14812* (P); [“Senegambia,”] Rio Nunez, 1828, *Heudelot s. n.* (K—2 sheets, one with date and flowers ex herb. Hook.); environs de Kindia, 1929–1932 (fl), *Jacques-Félix s. n.* (P); Nzérékoré, Nimba Mountains, just N of Camp 1 (Mifergui), 7°42.40’ N, 8°23.90'W, 650 m, 1 Dec 2006 (fl, fr), *Jongkind et al. 7407* (MO); Nzérékoré, Nimba Mountains, between Mifergui camp and old camp 2, 7°41.93'N, 8°23.76'W, 750 m, 23 Jun 2007 (fl), *Jongkind et al. 7818* (MO); Conakry (see Keay 1954–1958) [K sheet says Conokry on one label and Coleah on the other], 21 Oct 1896 (fl), *Maclaud 189* (K-000354678, P); Cungha, 11°05'50"N, 15°08'45"W, environs Jembezem, 27 Jan 1975 (fr), *Malaisse 14705* (BR); Biudélya, Apr 1903 (fr), *Paroisse 7* (P); Franceville, Ile Tristão [near Guinea-Bissau border on coast, 10°55, 14°55], *Paroisse 54* (P); Kouroussa, Jan 1903 (st), *Pobéguin 849* (P). **SIERRA LEONE.** Without definite locality, *Afzelius s. n.* (BM—same sheet as Smeathman specimen); without definite locality, 1854, *Daniell s. n.* (BM, MO); Mt Loma, 28 Sept 1945, *Jaeger 2018* (K); Waterloo, 29 June 1914, *Lane-Poole 319* (K); Sierra Leon Boundary Commission, 1891–92, *Scott-Elliot s. n.* (K). **LIBERIA.** Yékepa, Grassfield, 22 Feb 1965 (st), *Adam 20994* (MO), 23 May 1970 (fl), *Adam 25627* (MO); Sanniquellie, 1 Jun 1973 (fl), *Adam 27753* (MO); Grand Bassa Co., Fortsville, 3 Mar 1948 (fr), *Baldwin 11141* (MO); Bomi Hills, *Bos 2344* (K); along Dukwia [“Dukwai”] River (near Firestone Plantations), 1929 (fr), *Cooper 174* (A, K, BM, F, GH, NY, US); Grand Bassa, 27 Aug 1898 (fl), *Dinklage 2005* (A—2 sheets, B, K, MO); 22 mi N of Buchanan, 19 Feb 1970 (fr), *Jansen 1874* (MO); Division 33, Firestone plantations 10 mi S of Kakata, Aug 1970 (fl), *Jansen 2184* (MO); Bomi Hills, 8 Oct 1970 (fr), *Jansen 2219* (MO); 5 mi N of Bomi Hills, 18 Nov 1970 (fl), *Jansen 2281* (MO); near Cavalla River, E of Harper, 20 Jul 1971 (yg fr), *Jansen 2433* (MO); Bong Range, 32 km N of Kakata, 200 m, 11 Aug 1962 (fl), *Leeuwenberg & Voorhoeve 4924* (B, MO, P); near Fetoma, 2 Oct 1926 (fl), *Linder 881* (A); on road Bomi Hills–Yoma, 1 mile after bifurcation to “Smal Bopolu,” 24 Jul 1965 (fl), *van Meer 74* (MO); Gola National Forest, Bomi Hills, 15 Oct 1965 (fl), *van Meer 184* (MO); ca. 15 km E of Monrovia, between Paynesville and Duport, 22 Mar 1962 (fl, fr), *de Wilde & Voorhoeve 3633* (A, K, B, P). **IVORY COAST**. Soubré, Parc National de Taï, 18 Dec 1975 (fr), *Aké Assi 13159* (MO); région de Bassam, Yakassé, 18 May 1982 (buds, old fr), *Aké Assi 16073* (B, MO); Tchesso, 12 Oct 1997 (st), *Ambe 186* (MO); route Bouaké – Tiébissou, 2 Feb 1932 (yg fr), *Aubréville 828* (K, P); Abidjan, Jun 1932 (fl, yg fr), *Aubréville 1329* (P); Port-Bouet, *Aubréville 1944* (P); Lamto Station, ca. 6°15'N, 5°03'W, 11 Jul 1968 (st), *Breteler 5251* (K, MO); embouchure du Cavally à Bliéron, 11–13 Aug 1907 (fl, fr), *Chevalier 19912* (MO–-2 sheets, P); Bingerville, Jul 1901 (fl), *Jolly 287* (K, P); Baoulé, Oct 1895 (fl), *Pobéguin 255* (MO—2 sheets, P); bassin du Haut Sassandra et Bassin du Haut Cavally, Dec 1930 (fr), *Portères s. n.* (MO); 10 km SE of Dabou, direction lagune, 21 May 1969 (st), *Versteegh & den Outer 97* (MO); c. 6 km E. and 5 km inland of Bébéby forest bordering Savane de Néro-Mer, 6 Nov 1963, *Oldeman 514* (K); San Pedro, 13 Nov 1900 (fr), *Thoiré 85* (P). **GHANA**. Gold Coast, Princes W. Province, 15 January 1939, *Akpabla 797* (K); “Gold Coast,” 20 Dec 1923, *G. H. Eady s. n.* (K); Ankasa Forest Reserve, Mpataba-Elubo Rd., W Region, Jan 1973 (fr), *Enti R.1002* (MO). **TOGO**. Lzory [?], 1905, *Kersting A.181* (A, K). **BENIN**. Adjara, pres du Marché (cercle de Porto Novo), 17 Jan 1910, *Chevalier 22747* (K, MO—2 sheets, P); Mts Atakora [“Atacora”] Pays des So(rn)mbas de 400 m a 600 m alt de Natitingou à Bocorona, 21 Jun 1910 (fl), *Chevalier 24177* (K, P); Savi environs de Ouidah, Apr 1937 (fl), *Laffitte 92* (P). **NIGERIA**. Akwa Ibom: without definite locality [Eket, according to Keay 1954–1958], *Talbot 3281* (BM).—Cross River: Calabar Province, Uwet Dist., Buden Dunlop Estate, 23 Jun 1959 (yg fr), *Binuyo FHI 41375* (P); Southern Nigeria “Consulate Hill. O. Calabar, Mar 1900 (fl), *Holland 296* (K); Cross River National Park, Oban Hills, ca. 5 km NNW of Ntebachot, 5°32'2"N, 8°43'8"E, 19 Feb 1995 (st), *Schmitt et al. 429* (MO); Oban, S. Nigeria, 1911 (fl, fr), *Talbot 226* (BM).—Edo: Ohoghobi, 17 Aug 1908 (fr), *Forestry Department, Benin City 194* (K-000354684).—Lagos or Osun: Lagos, *Dawodu s. n.* (K); Southern, near Modakeke, Lagos (sic) Ado, *Foster 116* (K).—Ogun: Ijebu East, near Itele Junction along Shagamu Benin Road, 31 Aug 1994 (st), *Daramola 441* (F, MO).—Rivers: Nun River, 1860, *Mann s. n.* (no other data except name (K-0006233420 in herb. Hook.); Keay (1954–1958) cites *Mann 485* from this locality, so possibly the same as: “West tropical Africa,” 1859-63, *Mann 485* (GH)).–State unknown: S. Nigeria, Without definite locality [probably Sapoba], *Kennedy 2172* (A, BM, F, K, MO, US). **CAMEROON**. Bitya near the River Ja, *Bates 1813* (P); a 27 km route Mbalmayo, subdivision Yaoundé, 3 Oct 1953 (fl), *Benoit 61 (SRFK 1719)* (P); pres du Campement, forêt de Bidou—subdiv. Kribi, 27 Sep 1955 (fl), *Benoit 111 (SRFK 1916)*; km 48 route Kribi—Ndjabilobi, village Elone, 13 Dec 1958 (bud), *Benoit 282* (P); Ebolowa road, 5 km from Kribi, 2°55'N, 9°56'E, 20 Nov 1968 (fr), *Bos 3341* (P, WAG—2 sheets); NE of Mt. Elephant, about 20 km SE of Kribi, 2°48'N, 10°02'E, 10 Feb 1970 (fl), *Bos 6296* (B, MO, P); about 15 km from Kribi, 1 km S of Ebolowa road, 2°51'N, 10°01'E, 20 Feb 1970 (fl), *Bos 6382* (B, M, MO, P, WAG—3 sheets); South Province, Department Ocean, Mvie, about 11 km ENE of village Mvie, 2.55N, 10.39E, 400 m, 20 Jan 1998 (fl, yg fr), *van der Burgt 328* (MO); 3°37'33"N, 11°34'49"E, 700 m, Oct (fr), *Cheek 11057* (K); Douala route Razel, 1955-6, *Elias s. n.* (P); Bakolle Bakossi on Kumba-Mamfe road, 5°01'N, 9°40'E, 350 m, 24 May 1986 (fr), *Etuge & Thomas 148* (MO); 3°36'06"N, 11°35'49"E,700 m, 16 Oct 2002 (fl), *Gosline 423* (K); Dengdeng, Jul 1939 (fl, yg fr), *Jacques-Félix 4673* (P), *4679* (P); 60 km S of Edea, S of Mboké, 11 km E of km 58 of road Edea-Kribi, ca. 100 m, 22 Apr 1965 (fl, fr), *Leeuwenberg 5504* (K, MO, P, WAG); Piste du massif de Fessimi, 9 Feb 1961 (fl), *Letouzey 3433* (K, P—2 sheets); a 15 km au SE de Molobo (village situe à 50 km au S de Batouri), 21 Jul 1963 (yg fr), *Letouzey 5456* (P, WAG); Mebemonko (20 km NW d’Oveng), 26 Oct 1966 (fl, fr), *Letouzey 8186* (B, P—2 sheets); route Mintom I (70 km N de Djoum) a Alati (100 km SE de Djoum), PK 14, 1 Jan 1973 (fl), *Letouzey 11773* (K, P, WAG); Dengdeng ca. 700 m April 1914, *Mildbraed 8826* (K); Prov. Littoral, 5 km S of Dibombari, near Bonambwasse Village, 04°09'N, 09°41'E, 0-20 m, 18-20 Feb 1988 (fr), *Nemba et al. 812* (MO); Canon du Ntem, 16 km SW de Nyabessan, 30 Nov 1982 (fr), *Nkongmeneck 399* (P); Nkalé, 1924-5, *Pobéguin s. n.* (P); Southwest Province, forest in the Korup National Park, elev. 50 m, 5°03'N, 8°48'E, Feb-Apr 1984 (fr), *Thomas 3177* (B, MO, P); 16 km on the newly reconstructed road from Ebolowa to Minkok, and then about 2 km to the left along a forest exploitation track, ca. 650 m, 2°58'N, 11°17'E, 29 Jan 1975 (fl, fr), *de Wilde 7931* (B, K, MO, P); station du Cacaoyer de N’Koemvone, 14 km on the road from Ebolowa to Ambam, 2°49'N, 11°08'E, 17 Feb 1975 (fl), *de Wilde 7970* (K, MO, P); 17 km S of the Lobé River, along road to Campo, 2°43'N, 9°52'E, 18 Mar 1975 (fl), *de Wilde 8085* (BR, MO, P); station du Cacaoyer de N’Koemvone, 14 km along the road from Ebolowa to Ambam, 2°49'N, 11°08'E, 10 Dec 1975 (fr), *de Wilde 8707* (BR, MO, P); ca. 50 km NW of Eséka, W of Yaoundé, on opposite of the Kelè-river, 23 Nov 1963 (fl), *de Wilde 1322* (B, EA, K, MO, P); Mimfia, Jan 1914 (fl), *Zenker 499* (B, GH, M, MO, P, US); Bipinde, 1900 (fl, fr), *Zenker 2112* (A, B, BM, G, L, MO, WAG); Bipindi, Oct 1903 (fr), *Zenker s. n.* (F). **CHAD**. N’Djamena [“Fort Lamy”], Nov 1919 (fl), *Allouette s. n.* (L). **CENTRAL AFRICAN REPUBLIC**. Bai Hoker gorilla study area 25 km E of Bayanga, 02°52'N, 16°29'E, 435 m, 26 Feb 1988 (fl), *Carroll 1077* (MO); Chari oriental (Pays de Snoussi) [Dar Kouti, fide Tisserant and Sillans 1953], Bakala, 22 Jan 1903 (st), *Chevalier 7331* (P); Seriki près Kembe, (fl), *Eaux Forêts et Chasses 1946* (P); Botonnti, Tokoli, 4 Dec 1959 (yg fr), *Eaux Forêts et Chasses 1989* (P); Yagounda creek 5 km from Koumbala confluence 600 m, 8°25'N × 21°19'E, *Fay 4121* (K); Sangha Economique Prefecture, Ndakan, gorilla study area, 02°21'N, 16°10'E, 350 m, 8 Apr 1988 (fl), *Harris & Fay 447* (MO); Sangha Economique Prefecture, Ndakan, gorilla study area, 02°21'N, 16°09'E, 350 m, 12 Apr 1988 (fr), *Harris & Fay 479* (MO); Sangha Economique Prefecture, Ndakan, gorilla study area, 02°20'N, 16°10'E, 350 m, 28 Apr 1988 (fr), *Harris & Fay 536* (MO); Sangha Economique, Dzanga-Sangha Reserve, Ndakan Gorilla study area, 02°21'N, 16°10'E, 350 m, 16 Sep 1988 (fl), *Harris & Fay 1132* (K, MO); Yalinga, 1 Apr 1921 (fr), *Le Testu 2613* (BM, MO, P); Yalinga, 27 Mar 1923 (fl), *Le Testu 4635* (BM, P); Boukoko, 11 Oct 1947 (fl), *Tisserant 337* (BM, OWU, P); Boukoko, 20 Mar 1948 (fr), *Tisserant 791* (BM); région de Bambari, Maroubas, 3 Mar 1924 (fl, fr), *Tisserant 1436* (BM, P—2 sheets). **ETHIOPIA**. Rég. de Bonga, Jul 1909 (yg fr), *Rousseau s. n.* (L). **SAO TOME & PRINCIPE**. Principe: Ôque Pipi, ca. 1000 ft., 6 Dec 1932 (fl), *Exell 490* (BM—2 sheets); forest above Infante D. Henrique, ca. 1000 ft., 19 Dec 1932 (fl, fr), *Exell 620* (BM); Principe [“Prince’s Island”], 1861, *Mann s. n.* (K-000623422 ex herb. Hook); Infante Dom. Henrique [“Ricque”], 1956, *Rose 386* (P). **EQUATORIAL GUINEA.** Bioko: Bata-Bolondo, estrada km 15 e nos arredores da povoacao Macomo, 25 Jun 1991 (fr), *Carvalho 4751* (B, MO, NY); Moca to Areha Beach pt168 to pt 169, 3.2355 N, 8.6778 E, *Luke 12099* (K).—Río Muni: Spanish Guinea, 1908 und 1909 (fl), *Tessmann 913* (K). **GABON**. Estuaire: Envs. Libreville, Mar 1900 (fl), *Chalot 45* (P); env. Libreville, 1–5 May 1917 (fr), *Fleury 33530*, *33531*, *33532* (P); Libreville, Apr 1891 (fl, fr), *Klaine 176* (P); Libreville, 26 Jan 1896 (fl), *Klaine 310* (P); au platform de forage, Remboué 1, 0°13'S, 10°02'E, 21 Jan 1991 (fl), *Louis & Moungazi 3293* (MO); Réserve de Wongua Wongué, 23 Jan 1986 (fl), *Louis & Reitsma 2006* (WAG—2 sheets); Libreville, Route d’Idokogo, Reserve forestiere de la Mondah, 28 Oct 1948 (fr), *Morel 63 SRF* (P); Chantier Rougier-Ocean, Oveng, 0°52'N, 11°10'E, 7 May 1985 (fr), *Reitsma et al. 855* (MO, NY, RSA); Okala, N of Libreville, forêt de Mondah, Odeg, 0°37'N, 9°20'E, 4 Aug 1992 (fl, fr), *Wilks 2610* (MO).—Haut-Ogooué: Without definite locality, 10 Mar 1930 (fl, fr), *Le Testu 7960* (BM—2 sheets, MO); Batéké Plateau, Mpassa River watershed, Projet de Protection des Gorilles, 4 km S on Mpassa River, 02°08'15"S, 014°03'59"E, 6 Dec 2001 (buds), *Walters et al. 1079* (OWU); ca. 20 km on the road from Franceville to Lékoni, 450 m, 1°37'S, 13°45'E, 28 Aug 1992 (bud, fr), *Wieringa & van de Poll 1506* (MO, WAG—2 sheets).—Moyen-Ogooué: 5–15 km NNW of Ndjolé, 0°05'S, 10°45'E, Missanga, 13 Nov 1991 (fl, fr), *Breteler & Jongkind 10458* (W, WAG—2 sheets); sur l’Ogooué, près du lac Zile aux env. de Lambaréné, 10 Aug 1912 (st), *Fleury 26582* (MO, P); Petit Bam Bam, 21 Aug 1966 (fr), *Hallé & Le Thomas 581* (P); SW of Lambaréné, near Lake Ezanga, 01°00'S, 10°17'E, 15 m, 29 Jan 1991 (fl), *McPherson 15168* (MO, P); eastern part of the Presidential Reserve Wonga-Wongué, about 100 km S of Libreville, ca. 130 m, 28 Feb 1983 (fl), *de Wilde et al. 804* (MO).—Ngounié: Outembo 4 Nov 1916 (fl), *Le Testu 2178* (BM, MO); Waka National Park, along bank of Oténinga, 01°09'08"S, 011°06'36"E, 350 m, 2 May 2005 (fr), *Leal et al. 634* (MO); SE of Sindora, right bank of Ngoumié R., 17 km W of Camp Waka, 1°13'S, 10°49'E, 400 m, 22 Sep 1985 (fr), *Leeuwenberg & Persoon 13614* (MO, P, WAG), 27 Sep 1985 (fl), *Leeuwenberg & Persoon 13701* (K, MO, P); Fougamou, 5 km on forestry road following Bendolo River, 90 m, 1°11.7'S, 10°33.1'E, 2 Nov 1994 (fl, fr), *Wieringa et al. 3031* (W, WAG—2 sheets).—Nyanga: Tchibanga, 21 Nov 1914 (fl), *Le Testu 1877* (BM, EA, K, P); chantier CEB, ca. 50 km SW of Doussala, 2°36'S, 10°35'E, 28 Aug 1985 (fl, fr), *Reitsma & Reitsma 1442* (MO, NY—2 sheets, RSA); about 46 km along the track from Doussala to Igotchi, in SW direction from Doussala, 2°40'S, 10°32'E, ca. 160 m, 29 Nov 1986 (fr), *de Wilde et al. 9040* (B, BR, K, MO, WAG—3 sheets).—Ogooué-Ivindo: M’Passa Field Station, near Makokou on Riviere Ivindo, 480 m, 14 Jul 1981 (bud, fr), *Gentry 33323* (MO); Ipassa, 10 km S of Makokou, 500 m, 29 Oct 1971 (fl), *Hladik 1470 part A & B* (P), *part C* (US); Ijessa route du barage Makokou, 8 Feb 1972 (yg fr), *Hladik 1943a* (P); Res. Lopé-Okanda, 1 km S of Campement (= 26 km E of Ayem), 0°06'S, 11°30'E, ca. 200 m, 1 Nov 1982 (fl), *Leeuwenberg & Louis 12424* (K, MO, P, WAG); Réserve de la Lopé, au sud d’Ayem, chantier SOFORGA, ca. 200 m, 0°25'S, 11°30'E, 5 Mar 1989 (fr), *McPherson 13712* (MO); Lopé-reserve chantier SOFORGA, 0°30'S, 11°33'E, 25 Jun 1986 (st), *Reitsma & Reitsma 2355* (MO, NY); Ivindo National Park, ca. 2 km W of Langoúe Bai, 0°10'15"S, 12°32'33"E, 5 Dec 2002 (fr), *Stone & Niangadouma 3664* (MO); Lopé-Okanda Reserve, 2.6 km E along road from Kazambika to Offoué River, 00°07'12"S, 11°42'57"E, 290 m, 31 Oct 2000 (fl), *Walters et al. 465* (MO); Reserve de Lopé-Okanda, 200 m, 00°25'S, 11°30'E, 15 Jul 1990 (fr), *White [series 2] 29* (MO); Reserve de Lopé-Okanda, 00°25'S, 11°30'E, 200 m, 2 Oct 1990 (fl, fr), *White [series 2] 154* (MO); Lopé Reserve, 0°15'S, 11°40'E, 8/5/91 (yg fr), *White LJTW 0471* (MO); forêt des Abeilles, 21 km S du confluent Ogooué-Ivindo, 4 May 1983 (fr), *Wilks WIL 922* (WAG—2 sheets).—Ogooué-Lolo: “Deforestage Eurotrag,” about 20 km N of Lastoursville, 0°45'S, 12°43'E, ca. 250 m, 16 Nov 1983 (yg fr), *Louis et al. 798* (MO).—Ogooué-Maritime: Toucan, 01°47'665S, 9°53'387E, 1 June 2002 (yg fr), *Bourobou Bourobou et al. 654* (K, MO—2 sheets); SE of Port Gentil, about 0°40'S, 8°50'E, 16 Sep 1968 (bud, fr), *Breteler & van Raalte 5563* (MO, WAG); Gamba, about 2°46'S, 10°02'E, 21 Sep 1968 (fl, fr), *Breteler & van Raalte 5612* (MO, WAG—2 sheets); Cap Lopez, 2 Jul, (fl, fr), *Chevalier 4305* (P); Rabi, North of Divangui Road, S 1 52.6, E 9 56.1, 6 Mar 2007 (fr), *Choo 764* (MO); near Lake Divangui, 20 m, 1°57'S, 9°59'E, 2 Jan 1994 (fr), *Haegens & van der Burgt 233* (WAG); near RamboRabi, NW of Rabi site, 1°53.8'S, 9°50.5'E, 10 Nov 1990 (fr), *van Nek 300* (WAG); forest near Checkpoint Est, 1°52'S, 9°58'E, 17 Nov 1990 (fr), *van Nek 345* (WAG); Rabi-Kounga, Divangui, ca. 1°57'S, 9°59'E, 25 Dec 1991 (fr), *Schoenmaker 306* (WAG); Ogooué, route entre Batanga et la riviére Awagné 0 18 10 S 9 18 40 E, *Sosef 1759* (K); Gamba, 7.3 along road from airport to Vera, ca. 2°47'S, 10°06'E, 23 Nov 1994 (fl, fr), *de Wilde & de Wilde-Bakhuizen 11172* (WAG—2 sheets); Olendé, 01°19'S, 09°02'E, 29 Dec 1988 (fl), *Wilks 1890* (MO, OWU).—Woleu-Ntem: Woleu-Ntem, Mbe National Park, Monts de Cristal, Tchimbele Dam area, 00°37'08"N, 010°24'35"E, 300 m, Apr 2004 (st), *SIMAB 020102* (MO); about 22 km NE of Asok, newly constructed road, close to the Mbé River, ca. 600 m, 23 Aug 1978 (fr), *Breteler & de Wilde 246* (K, MO); 0.5 km NW of Tchimbélé, 550 m, ca. 0°37'N, 10°23'E, 28 Jan 1990 (fl), *Wieringa 485* (WAG).—Province unknown: Kouilou, N’kana, 4 Mar 1920 (fr), *Sargos 60* (P), 4 Mar 1920 (bud), *Sargos 126* (P), 12 Feb 1924 (fr), *Sargos 206* (P), 15 Oct 1929 (fr), *Sargos 223* (K, P); in ditione Munda Sibange = Farm, 22 Sep 1880 (fr), *Soyaux 131* (K). **REPUBLIC OF THE CONGO**. Village de Loubofo, forêt sur les bords de la Bouénza depuis les chutes jusqu’au bord de la route à 5 kms du village, 8 Nov 1964 (bud, fr), *Bouquet 685* (P); bassin de l’Alima-Likouala, environs de Fort Rousset à 16 km sur la route de Gamboma, 21 Jul 1961 (fl), *Descoings 8272* (P); Guéne [Guena = Bilala, 4°26’, 12°15’, NE of Pointe Noire], 1960 (fr), *Centre Technique Forestier Tropical CTFT 170* (P); Ouesso, 12 May 1971 (st), *Grison FG 87* (P); Kouilou, Bena, 8 Oct 1990 (fr), *Lisowski B-7179* (BR); Pool, Réserve de Chasse de Léfini, banks of the Léfini River near its confluence with the Louna River, ca. 20 km upstream from Mbouanbé, 320 m, 3°00'S, 15°28'E, 24 Oct 1991 (fl), *Thomas et al. 8746* (MO). **DEMOCRATIC REPUBLIC OF THE CONGO**. Bas-Uele: Tukpwo, galeire de la Diagbe, 25 Jun 1954 (fr), *Gerard 1532* (BR). Digba, Prov. Orientale, Terr. Ango, forêt des Akare entre riviére Bili et Asa, 7 Nov 1963 (fr), *Gerard 5574* (WAG—2 sheets), *Gerard 5616* (MO, WAG); piste Gwane-Balikwe, Jan 1946 (fr), *Germain 737* (FI-T, K, P).—Équateur: Eala, 30 Apr 1928 (fl), *Corbisier[-Baland] 806* (A, EA, K, MO, US); Botsima, 1°09'S, 21°57'E, 26 Feb 1991 (buds), *Dhetchuvi 756* (BR); Eala, 1936 (fl), *Leemans 488* (K, MO, P, US); Boyeka (Eala), 16 Aug 1946 (fl, fr), *Léonard 311* (BM, K, MO, P, US); de long d’un bras de la riviere Ruki le “Yali,” près d’Eala, Équateur, 2 Jun 1936 (fl), *Louis 2147* (RSA).—Haut-Katanga: environs de Elisabethville, galerie de la Kisanga Dec 1939 (fl), *Quarré 6201* (BR).—Katanga: Kamunza, 25°50'E, 07°02'S, fin Sep 1957 (buds), *Schmitz 5764* (BR).—Kinshasa: Maluku, Tpiene (Menkao ambouchure Bombo), 21 Oct 1970 (fl), *Breyne 934* (MO); Kimuenza, 23 Jun 1959 (st), *Pauwels 3411* (BR); au dela de la Nsele terr. Maluku, 26 Jan 1965 (fl), *Pauwels 4877* (MO, WAG—2 sheets).—Kongo Central: Leopoldville, Prov. Thysville, Territ. Songa, *Devred 885* (K); Kinkasi (Benga), Terr. Popakabaka, 1 Jul 1959 (st), *Pauwels 3617* (BR); vers Kingoma, Terr. Popakabaka, 4 Jul 1959 (fr), *Pauwels 3760* (BR), 22 Jul 1959 (st), *Pauwels 3936* (BR); Luki, 8 Aug 1949 (yg fr), *Toussaint 2430* (EA, K, P).—Kwilu: Kiyaka – Kwango, Prov. Katanga, Terr. Kikwit, 7 Sep 1955 (fr), *Devred 2585* (WAG—2 sheets).—Mongala: Équateur Prov territ Bumba loc. Vallee de la Loeka, 5 Feb 1958 (fr), *Evrard 3436* (K).—Nord-Kivu: Walikale Mt. Mika, 1°26'S, 28°03'E, 850 m, 27 Jun 1957 (fr), *Pierlot 1707* (BR).—Sankuru: Katakokombe, Sep 1932 (fr), *Lebrun 6117* (K, P).—Sud-Kivu: Bulumbu Terr. Shabunda, 780 m, 9 Apr 1959 (buds), *Léonard 3759* (BR); Nzowo, Prov. Kivu, Terr. S[c]habunda, 1180 m, 13 Apr 1959 (fl), *Léonard 3837* (MO).—Tshopo: Prov. Orientale, Terr. Tsangi, Yangambi, Jan 1961 (fl), *Bolema 374* (P); Prov. Orientale, Terr. Tsangi, Ligasa-Mangala, forêt periodiquement inondeé de la Lukombe, 21 Mar 1957 (fl), *Evrard 2262* (WAG); Yangambi, Prov. Orientale, Terr. Tsangi, 23 Jul 1958 (fl), *Léonard 980* (BR, EA, MO, WAG); Yangambi, 6 Feb 1936 (fr), *Louis 1203* (BM, K); Yangambi, km 8.400 de la route de Ngazi, 17 Nov 1936 (old fl), *Louis 2848* (NY); Yangambi, ca. 470 m, 27 Apr 1938 (fr), *Louis 9099* (B, BM, K, MO, P, US).—Tshuapa: Botsima, 1°09'S, 21°57'E, 7 Mar 1991 (fr), *Dhetchuvi 874* (MO); dist. de la Tshuapa, Sep 95 (fl), *Dubois 780* (K, P); cerque de Malela 16 Nov 1945 (fl), *Donis 1354* (K).—Province unknown: entre Bubinga et Tarawa, Jan 1931 (fl), *Lebrun 1964* (K, MO, P). **UGANDA**. Entebbe District, 3900 ft., 1904, *Dawe 118* (K); Bujeje District, 3600 ft, 1905 (fl, fr), *Dawe 229* (K); Masaka District, NW side of Lake Nabugabo, 1140 m, 9 Oct 1953 (fl, fr), *Drummond & Hemsley 4714* (B, EA, FI-T, K—2 sheets); Namanve, Mengo, 3700 ft., Jan 1935 (fr), *Eggeling 1524* (BR, K—indicates *Eggeling 1583* to be a wood collection of this number, but duplicates at EA and NY bear only the number 1583); without definite locality, 1922 (yr fr), *Maitland s. n.* (K). **KENYA.** Kwale: Ramisi-Mrima Hill road, ca. 7 km past the crossing of the Ramisi River, ca. 4°32'S, 39°19'E, ca. 40 m, 23 Mar 1974 (fl, fr), *Faden & Faden 74/306* (K, MO, WAG); Shimba Hills, Mwele Grid Ref. 3922E0416S, 300 m, 13 Nov 1992 (st), *Luke 3378* (EA); Shimba Hills, Buffalo Ridge, Grid Ref 3926E0413S, 280 m, 15 Mar 1991 (fl), *Luke & Robertson 2709* (K, MO, US). **TANZANIA.** About 20–30 km SW of Bukoba, 1300 m, 31 Oct 1992 (fl), *Breteler 11626* (WAG). Rubare, Bukoba, Bukoba District, Lake Province, 4000 ft., Jul 1951 (buds, fr), *Eggeling 6241* (K); Lindi, SE Tanganyika, Chilangal, 800 m, 16 Dec 1942 (fl), *Gillman 1261* (K); Bukoba District, Rubogo Swamp, Sep-Oct 1935 (fl), *Gillman 398* (BM, K); Amani, 16 Nov 1928 (fl, fr), *Greenway 1006* (K); Mlinga, 2800 ft. alt., 18 Feb 1937 (fl), *Greenway 4909* (EA, K); Tanga Region, Muheza District, Amani, Mbomole Hill Trail, 05°02'S, 39°10'E, 900 m, 4 Jun 1996 (st), *Johnson et al. 1943A* (OWU), *1943B* (OWU), *1944* (OWU); Stromgebiet des oberen Ruhudje [tributary of Kilombero by Njombe], Landschaft Massagati nördlich des Flusses, ca. 35°30'E, 700 m, 27 Nov 1931 (fl), *Schlieben 1480* (BM, K); Amani, *Zimmerman 2630* (A, BM, EA). **ANGOLA.** At Rio Munze, Buco Zau, 26 Aug 1916 (bud), *Gossweiler 6612* (BM); along the river Luali, Buco Zau, Mayumbe, 27 Dec 1916 (fl), *Gossweiler 6903* (BM, K—2 sheets); Belize, 10 Mar 1917 (fl), *Gossweiler 7032* (BM); banks of the rio Lufo, Maiombe, 22 Feb 1919 (fl), *Gossweiler 7845* (BM, K–3 sheets); Sumba, Peco, proximum flumen Zaire, 3 Dec 1924 (fl), *Gossweiler 8972* (BM, K, US); Maiombe, Nkanda Mbaku, proximum flumen Luali-Chiloango, 50 m, 22 Jun 1924 (fr), *Gossweiler 9060* (BM); Maiombe, Nkanda Mbaku, proximum flumen Luali-Chiloango, 50 m, 25 Apr 1923 (fl), *Gossweiler 9073* (BM); Sumba, Peco, July 1926 (fr), *Gossweiler 9134* (A, BM, MO, US); Sumba, Peco, proximum flumen Zaire, Jun 1926 (fr), *Gossweiler 9138* (BM, US); Dundo, proximum flumen Luachimo, Mar 1949 (st), *Gossweiler 14222* [leg. Barros Machado] (BM, P); Mayumbe, *Gossweiler s. n.* (BM); Hochland von Quela, *Nolde 216* (BM); in regno Hungo ad limites boreali-orientales regni Angolae, s. d. (fr), *Welwitsch 765* (BM). **ZAMBIA.** Copperbelt: Chingola, Luano Catchments, 31 Oct 1978 (fl), *Bingham 3250* (K); Mufulira, 11 Aug 1954 (fr), *Fanshawe 1447* (K).—Northern: Shiwa Ngandu, 5400 ft, 21 Jul 1938 (fl, fr), *Greenway 5452* (EA, K); Chinsali District, Shiwa Ng’andu, Mansha River, Chusa Falls, 11°09'19"S, 31°33'04"E, 1470 m, 25 Nov 1993 (fl, fr), *Harder et al. 2126* (MO).—Northwestern: Mwinilunga, 7 Sep 1955 (fr), *Holmes H.1179* (K); Mwinilunga District, R. Matonchi, 21 Oct 1937 (fl), *Milne-Redhead 2883* (A, B, BM, BR, EA, FI-T, K—2 sheets, P). **MALAWI.** Southern: near Likabula Forest Station, at about 3000 ft, 4 Dec 1957 (fl), *Chapman 494* (BM, K); lower slopes Mt. Mulanje, near Manager’s House at Esperanza Estate, 700 m, 18 Nov 1985 (fl, fr), *Chapman & Chapman 6829* (MO); half way up the outer slopes of Mt. Mulanje at a cliff foot in a hollow on the mountainside above the Chitakale stream (east branch), in full view of the Boma Path to Lichenya Plateau, 4 Dec 1985 (fl), *Chapman & Chapman 6921* (K, MO). **MOZAMBIQUE.** Cabo Delgado: Cabo Delgado, 11°33'05"S, 39°20'51"E, 796 m, 11 Sep 2009 (st), *Lötter & Turpin 1898* (K).—Mozambique: Serra Chinga, entre a Chinga-1 e a Chinga 2-A caminho do Chinga 2 [14°59'37.01"S, 38°33'20.98"E], 28 May (fl), *Aguiar Macedo & Macuácua 3291* (DSM).—Niassa: Chomba, entre o cruzamento & Chomba, 20 Sep 1948 (st), *Pedro & Pedrogão 5285* (EA).—Ribáuè: encosta da Serra Mepáluè (Ie.), 700 m, 12 Dec 1967 (yg fr), *Torre & Correia 16453* (BR, EA, K); serra de Chinga, 1100 m, 12 Dec 1967 (fl), *Torre & Correia 16460* (B, WAG).—Sofala: Cheringoma, Dando, serração de Inhansato, de Cardoso & Lopes, 1 Jun 1948 (yg fr), *Mendonça 4442* (EA, K, MO, WAG).—Zambezia: Mabu Mountain [16.2825°S, 36.3817°E], (fl, fr), *Harris 644*(K). **ZIMBABWE.** Manicaland: [Melsetter District], 1300 ft, 3 Sep 1964 (fl, fr), *Wild et al. 6618* (BR, K); Inyanga District, Eastern Highlands Plantation, Pungwe Valley, 3500 ft, 9 Nov 1960 (fl, fr), *Wild 5267* (K, MO).—Province unknown: Near Mozambique border, *Müller 564* (K).

*Xylopiaaethiopica* is readily distinguished from all other African *Xylopia* species by the distinctly connate sepals, the large number (up to 36) of narrow, weakly torulose monocarps, and the bilobed aril of the small seeds. The inflorescences are always pedunculate, with the 2–4 large flowers borne on long pedicels. In dried condition, the subcoriaceous leaves have a distinctive dark red midrib on the adaxial surface and a pale abaxial blade surface. *Xylopiaaethiopica* is sometimes confused with *Xylopiarubescens*, which also has relatively narrow monocarps. *Xylopiarubescens* has, however, inner petals much shorter than the outer petals, up to 15 monocarps that are strongly torulose and usually distinctly beaked at the apex, and a distinctive reddish cast on the abaxial leaf surface The aril on the seeds of *Xylopiarubescens* also has the brushlike appendages characteristic of section Neoxylopia.

Although *Xylopiaaethiopica* shows morphological variability, we could not detect any consistent ecological or geographic pattern to the variation. In the field in northeastern Tanzania, we observed that smaller individuals have relatively large “shade” leaves, and that leaves in the canopy of large trees tend to be smaller and narrower “sun leaves.” Herbarium specimens with such small leaves were occasionally seen, e.g. *Leemans 488*, *Léonard 311*, both from the western Democratic Republic of the Congo). There is variability in the number of monocarps per fruit and in the number of seeds per monocarp, but this seems to reflect pollination success, with small fruits being scattered throughout the distribution. A number of specimens from the Congo River basin exhibited relatively small flowers at maturity, with petals only ca. 20 mm long, more flowers per inflorescence, sometimes as many as seven, glabrous or sparsely pubescent foliage and inflorescences, smaller numbers of carpels (11–14), and a smaller staminal cone (2.2–2.4 mm in diameter) in the flower. It is possible that this variant is the plant described as *Xylopiagilletii* by De Wildeman, but the type specimen of that name is in fruit. A field study of this variant is needed.

*Xylopiaaethiopica* grows in a range of habitats across its distribution, and it is difficult to determine from herbarium label data whether its presence in secondary vegetation, and along riverbanks, is spontaneous or the result of deliberate planting and tending. Only a single collection (Kisantu, cultivé, 7 Sep 1912 (st), *Chevalier 28331* (P)) explicitly states that the specimen came from a cultivated plant. Savill and Fox (1967) described *X.aethiopica* in Sierra Leone as inhabiting a wide variety of forest zones, and explicitly characterized it as a tree of secondary forest, where it often formed pure stands in regenerating forest but was absent from older forests (Savill and Fox 1967). In eastern Africa, the species is largely restricted to mountainous areas where the rainfall is higher than in the surrounding lowlands. In the Usambara Mountains near Amani, we found it growing in sapling, subcanopy, and canopy stages in an area of disturbed forest, at one site occurring with smaller trees of *Parinariexcelsa* at the top of a steep slope along a road.

As discussed in the Ethnobiology section, the ethnobotanical literature attests to the continued wide use of the monocarps across West and Central Africa as a spice and medicine. It has been used since ancient times for these purposes. We have seen herbarium specimens from Nigeria (Lagos, *Dawodu s. n.* (K)), Cameroon (Messa Market, Yaoundé, 7 Mar 1979, *Westphal 10202* (P), and Gabon (without definite locality, 1854, *Aubry-Lecomte s. n.* (P)) with labels stating that the fruits were obtained from or sold in the market. The label of *Dawodu s. n.* adds “invariably included in the Agbo pot to give tone and strength to the whole compound.” Van Andel (2015) recently reported that the fruits of the Neotropical species *X.discreta* in Suriname are used medicinally, in ways similar to those for *X.aethiopica*, by local people of African descent. The name van Andel reported for the plants in Suriname was “pechereku,” and she observed its similarity to the Fon name “pedjericou” applied to *X.aethiopica* in Benin.

*Xylopiaaethiopica* has been reported as a food source for animals. Usongo and Amubode (2001) indicated that the young leaves of *Xylopiaaethiopica* made up 22.2% of the diet of red colobus monkeys (*Colobusbadiuspreussi*) at a field site in Korup National Park in western Cameroon. In Ivory Coast, the arillate seeds are consumed by Diana monkeys, which spit out the seeds (Koné et al. 2008) and, in Cameroon, three species of monkeys eat and defecate the seeds (Poulsen et al. 2001). The seeds are also reported to be taken by two species of hornbills in Cameroon (Wang 2008); Gautier-Hion (1985) and Clark and Poulsen (2001) reported the seeds of *X.aethiopica* to be bird-dispersed. Seeds of *X.aethiopica*, with the aril still intact, were recovered by T. Engel from the dung of a genet cat in the Shimba Hills of Kenya.

We follow the conclusion of Brizicky (1962) in accepting the year of publication of the combination *Xylopiaaethiopica* as 1841, rather than the printed date of 1845. A lectotype for *Unonaaethiopica* Dun. was chosen by Verdcourt (1971b), who indicated the lectotype to be at G. In the Prodromus herbarium at G there are three sheets, however, and it is not clear whether the lectotypification was intended to include all of them or just a single sheet, so a secondary lectotypification is explicitly designated here.

### IV. Xylopia Section Verdcourtia

Taxon classificationPlantaeMagnolialesAnnonaceae

D. M. Johnson & N. A. Murray sect. nov.

B9FF4DA4-CDED-5176-9B1B-1D8075E08A81

#### Type.

*Xylopiamwasumbii* D. M. Johnson.

#### Diagnosis.

Nodes with branches from 2–3 axillary buds; outer and inner petals lanceolate, similar in length; inner petals glabrous, flat at the base and lacking differentiated fleshy basal margins; anther connectives rudimentary at apex, not overhanging the anther thecae, staminal cone rudimentary, surrounding only the bases of the ovaries, rim laciniate; carpels 2–5, the stigmas discrete, thickened and falciform; aril cupular, surrounding only the base of the seed; seed coat smooth, sarcotesta absent. Three species in Tropical Africa and at least two additional species in Madagascar.

#### Notes.

This species group in *Xylopia* was distinguished on morphological grounds (Johnson et al. 2017) and was well supported as an early-diverging monophyletic subclade within the Stenoxylopia clade in the molecular phylogenetic analysis (Stull et al. 2017). With a more comprehensive study of *Xylopia* species, we find that this species group is marked by synapomorphies comparable to those used to distinguish other sections in the genus. These include glabrous inner petals, rudimentary anther connective apices, relatively short (0.7–2 mm long) and broad stigmas, and a smooth fleshy white cupular aril (Fig. 4D). Species of this section also share outer petals spreading and inner petals erect at anthesis, stamens reduced in number (65 or fewer), and monocarps, where known, that are laterally compressed.

The section is named in memory of Bernard Verdcourt, expert on African Annonaceae and the first author to draw attention to the species of this section, through his descriptions of *Xylopia* “species A” and *Xylopia* “species B” (Verdcourt 1971b).

### 9. Xylopia lukei

Taxon classificationPlantaeMagnolialesAnnonaceae

D. M. Johnson & Goyder, Kew Bull. 72:11: 3–7. 2017.

6F5D8AA8-8BCC-5D14-A93A-C2DABEF560DD

Fig. 18A–F, H

#### Type.

MOZAMBIQUE. Cabo Delgado Province, Nangade–Palma, 180 m, Lat 10°54'S, Long 39°54'E, 15 Dec 2003, *Q. Luke et al. 10166* (holotype: EA!; isotypes: K! MO! [5795498]).

#### Description.

***Tree or shrub*** up to 9 m tall. ***Twigs*** light gray to light brown, sparsely appressed pubescent, the hairs 0.2–0.3 mm long, eventually reddish brown, glabrate, with bark exfoliating; nodes occasionally with two axillary branches. ***Leaf*** with larger blades 8.2–11.9 cm long, 4.1–5.8 cm wide, subcoriaceous, slightly discolorous, broadly elliptic to ovate, occasionally oblong-elliptic, apex acuminate with an acumen 7–14 mm long, base broadly cuneate to rounded and slightly decurrent on petiole, glabrous adaxially, sparsely appressed-pubescent to glabrous abaxially; midrib plane adaxially, raised abaxially, secondary veins irregularly brochidodromous, 8–15 per side, diverging at 45–80° from the midrib, these and higher-order veins slightly raised on both surfaces; petiole 3.5–10 mm long, flattened to shallowly canaliculate, glabrous to sparsely pubescent. ***Inflorescences*** axillary or rarely slightly supra-axillary, 1–5-flowered, appressed-pubescent; peduncles 1–2 per axil, 1.3–2.6 mm long; pedicels 1–3 per peduncle, 4.5–6 mm long, 1.0–1.2 mm thick; bracts 1–3, usually 2, evenly spaced on pedicel, persistent or with lowest caducous, 1.6–2.9 mm long, ovate to semicircular, apex acute to rounded; buds oblong-lanceolate to oblong, apex obtuse. ***Sepals*** slightly spreading at anthesis, 1/4–1/2-connate, 2.6–4.7 mm long, 2.6–4.2 mm wide, coriaceous, oblong, the free portion somewhat pentagonal, apex short-acuminate to acute, appressed-pubescent abaxially. ***Petals*** apricot-colored to brownish yellow *in vivo*; outer petals wide-spreading at anthesis, 8.8–15.5 mm long, 2.8–4.2 mm wide at base, 2.5–3.3 mm wide at midpoint, coriaceous or slightly fleshy, lanceolate, apex acute to obtuse, densely pubescent along margins and at the apex but glabrous and verrucose toward the base adaxially, densely appressed-pubescent abaxially; inner petals erect at anthesis, 9.8–12.5 mm long, 2–2.7 mm wide at base, 1.3–2.1 mm wide at midpoint, coriaceous or slightly fleshy, lanceolate, apex acute, base with undifferentiated margin, wrinkled, verrucose, glabrous. ***Stamens*** ca. 65; fertile stamens 1.6–2.3 mm long, narrowly oblong, apex of connective ca. 0.1 mm long, truncate, not overhanging anther thecae, glabrous, anthers 6–12-locellate, filament 0.7–1.0 mm long; outer staminodes ca. 1.5 mm long, oblong, apex rounded to truncate; inner staminodes 1.0–1.2 mm long, oblong, apex truncate; staminal cone 1.0–1.2 mm in diameter, ca. 0.8 mm high, concealing only the bases of the ovaries, rim laciniate. ***Carpels*** 3–4; ovaries 1.8–2.0 mm long, narrowly oblong, pubescent, stigmas loosely appressed, 1.5–2.0 mm long, lanceolate, apex acute, sparsely pubescent to glabrous. ***Torus*** flat or a little bumpy, 1.3–1.7 mm in diameter. ***Fruit*** and ***seeds*** unknown.

**Figure 18.**
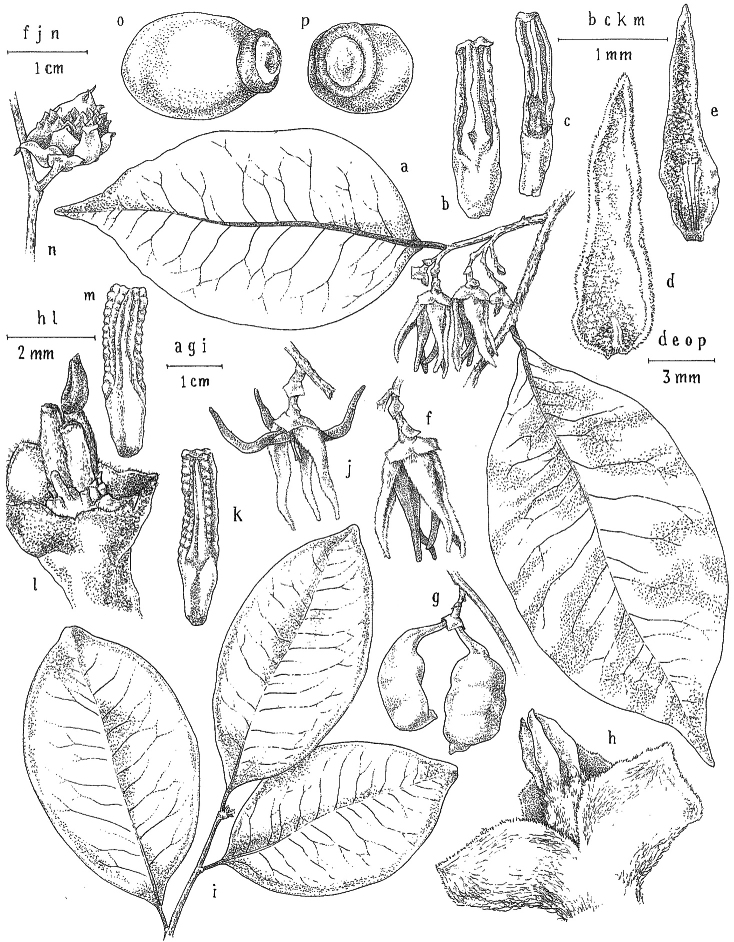
*Xylopialukei* and *X.mwasumbii*. **A–F, H***X.lukei***A** Habit **B, C** Stamens, abaxial view **D** Outer petal, adaxial view **E** Inner petal, adaxial view **F** Flower, side view **H** Flower with petals removed, showing carpels **G, I–P***X.mwasumbii***G** Fruit **I** Leaves **J** Flower, side view **K, M** Stamens, abaxial view **L** Gynoecium and surrounding staminal cone **N** Flower gall **O** Seed with aril removed, side view **P** Seed with aril removed, view of micropylar end. **A–F, H** from *Luke et al. 10166* (MO) **G** from *Johnson & Ndangalasi 1899* (OWU) **I, J** from *Johnson 1920* (OWU) **K–M** from *Johnson 1928A* (OWU) **N** from *Johnson & Ndangalasi 1948* (OWU) **O, P** from *Johnson & Ndangalasi 1884* (OWU) **K–M, O, P** were drawn from ethanol-preserved material and so have dimensions slightly greater than those in the description, which is based on dried specimens. Reproduced with the permission of the Board of Trustees, Royal Botanic Gardens Kew.

#### Phenology.

Specimens bearing mature flowers have been gathered in November and December; specimens with flower buds only have been collected in May and September.

#### Distribution

**Figure 19.**
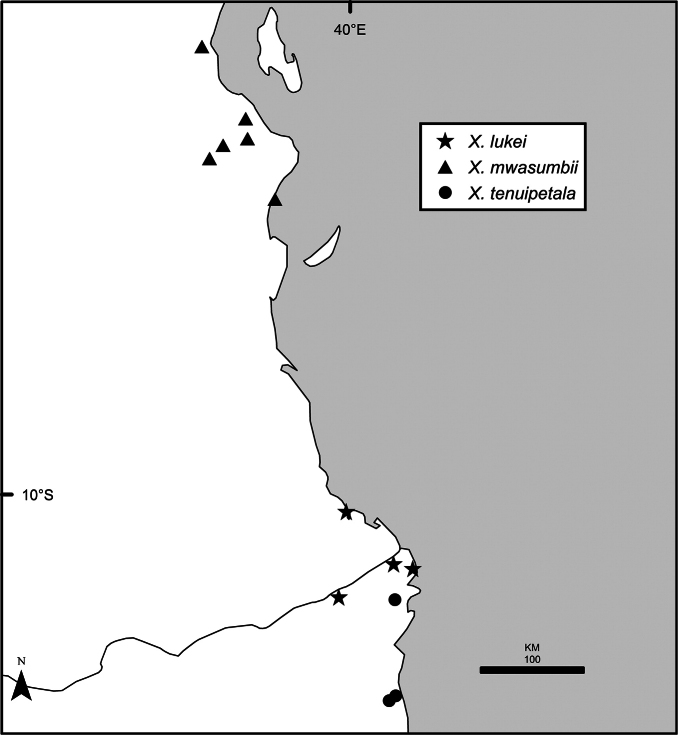
Distributions of *Xylopialukei*, *X.mwasumbii*, and *X.tenuipetala* in eastern Africa. Bold lines represent country borders.

(Fig. 19). Restricted to southeastern Tanzania and northeastern Mozambique, where it occurs in coastal dry forest on reddish orange sands below 200 m.

#### Additional specimens examined.

**TANZANIA.** Lindi: Sudi [ca. 10°09'S, 39°58'E], 25 May 1943 (fl buds), *Gillman 1465* (EA, K). **MOZAMBIQUE.** Cabo Delgado: between Pundanhar and turnoff to Hunters Mozambique hunting camp, towards Nangade, 10° 54’ 7"S, 39° 56’ 44"E, 169 m, 7 Sept 2009 (fl buds), *Burrows et al. 11390* (BNRH, K); Palma District, patch of forest on the coastal plain between the Cabo Delgado peninsula and Quionga, 10°39'03"S, 40°32'59"E, 16 m, 6 Nov 2009 (fl buds), *Clarke 97* (K, LMA); N of Palma, on cut line 11 off main road from Palma to Namoto, Way Point: 082 10˚37'27.3"S, 40˚25'05.7"E, ca. 90 m, 6 Dec 2008 (fl buds), *Crawford et al. FC323* (K, LMA, P); Nangade to Pundanhar pt427 10.9091°S, 39.9170°E, 168 m, 12 Nov 2009 (fl), *Luke & Luke 13760* (EA, K, LMA, MO, P); Quionga to Nachindundo pt437 10.6010°S, 40.6160°E, 60m, 14 Nov 2009 (fl), *Luke & Luke 13794* (EA, K, LMA, P); Palma District, Pundanhar to Nangade road. 10°54'29"S, 39°55'12"E., alt. 177 m, 6 Nov 2009 (fl buds), *Müller & Clarke 4159* (K, LMA).

*Xylopialukei* is distinctive within section Verdcourtia by virtue of its larger leaf blades, 8.2–11.9 cm long and 4.1–5.8 cm wide, usually distinctly brown-discolored on the abaxial surface, a complex inflorescence consisting of up to five flowers with some pedicels branching from a common peduncle, the pedicels with bracts 2.5–2.9 mm long, and pubescent sepals 3.7–4.7 mm with the free portion of the sepal somewhat pentagonal in shape. In other species of the section, the leaf blades do not exceed 8.7 cm in length and are concolorous, the inflorescences comprise one to two (rarely three) flowers, the bracts of the pedicel only reach 1.4 mm in length and are glabrous, and the sepals do not exceed 2.6 mm in length, are at most sparsely sericeous, and have the free portion broadly triangular in shape.

The coastal dry forests where *X.lukei* occurs were the subject of a recent floristic analysis by Timberlake et al. (2011), who identified at least 23 other plant species with a similar pattern of endemism. Species mentioned on herbarium labels as associates of *X.lukei* include *Chassalia* sp., *Erythrococcaatrovirens*, Pancoviaholtziisubsp.holtzii, Pyrostriasp.cf.bibracteata, Rinoreaangustifoliasubsp.ardisiiflora, *Uvariaacuminata*, and *Warneckeasousae*. The forests where the plants occur are currently under threat from road expansion and other land development, and a conservation assessment of Endangered was recently proposed for it (Johnson et al. 2017; Table 1).

*Xylopialukei* was informally designated as *Xylopia* “species A” by Verdcourt (1971b), who did not have adequate material to formally describe it. Additional material now supports Verdcourt’s taxonomy.

### 10. Xylopia mwasumbii

Taxon classificationPlantaeMagnolialesAnnonaceae

D. M. Johnson, Novon 9: 55–58. 1999.

F9BDC148-EE76-5B41-9109-DA35040799E9

Figs 18G, I–P

#### Type.

TANZANIA. Dar-es-Salaam Region, Kisarawe District, Pugu Forest Reserve, along N road 0.5 km E of brick factory, 6°52'S, 39°06'E, 200 m, 16 Feb 1996, *D. M. Johnson & H. J. Ndangalasi 1884* (holotype: OWU!; isotypes: DSM! K! MO! [217000]).

#### Description.

***Tree*** up to 9 m tall, d.b.h. up to 10 cm, often with multiple trunks, the principal trunk often arching rather than erect; bark white with gray and orange mottling, mostly smooth but exfoliating in patches. ***Twigs*** gray, sparsely appressed-pubescent, the hairs 0.2–0.4 mm long, eventually light gray to gray-brown, glabrate; nodes frequently with two or more axillary branches. ***Leaf*** with larger blades 4.6–8.7 cm long, 2.4–4.3 cm wide, subcoriaceous, concolorous but paler abaxially, broadly elliptic to elliptic, occasionally ovate, elliptic-ovate, or obovate-elliptic, apex blunt-acuminate with an acumen 4–8 mm long, occasionally obtuse and emarginate, base broadly cuneate and decurrent on petiole, glabrous adaxially, sparsely sericeous abaxially; midrib plane adaxially, raised and somewhat keeled abaxially, secondary veins weakly brochidodromous, 9–11 per side, diverging at 55–70° from the midrib, these and higher-order veins indistinct but raised adaxially, slightly raised abaxially; petiole 4.5–8 mm long, flattened to canaliculate adaxially, glabrous to sparsely appressed-pubescent. ***Inflorescences*** axillary or from the axils of fallen leaves, 1–3-flowered, sparsely pubescent to glabrate; peduncle 1 per axil or absent, 1.9–4 mm long; pedicels 1–2 per peduncle, 2.4–3.5 mm long, 0.8–1.6 mm thick; bracts 2–3, evenly spaced along pedicel, caducous to persistent, 0.8–1.6 mm long, ovate, quadrate, semicircular, or deltate, occasionally tearing down the center as the inflorescence enlarges, apex obtuse; buds ovate-lanceolate to lanceolate, apex obtuse. ***Sepals*** slightly spreading at anthesis, 1/4–1/2-connate, 1.4–2.2 mm long, 1.5–2.2 mm wide, coriaceous, elliptic, apex obtuse to acute, sparsely sericeous abaxially. ***Petals*** olive-yellow to white *in vivo*; outer petals spreading at anthesis, 8–10 mm long, 2–3 mm wide at base, 2.3–2.6 mm wide at midpoint, coriaceous to slightly fleshy, linear-lanceolate to narrowly triangular, apex acute, appressed-pubescent adaxially, sericeous abaxially; inner petals erect at anthesis, 5.7–7.5 mm long, 1.8–2.2 mm wide at base, ca. 2.0 mm wide at midpoint, coriaceous to slightly fleshy, lanceolate, apex acute, base with undifferentiated margin, glabrous or with a few hairs. ***Stamens*** 40–45; fertile stamens 1.5–1.8 mm long, narrowly oblong, apex of connective obtuse to truncate, not exceeding anther thecae, anthers 6–13-locellate, filament ca. 0.7 mm long; outer staminodes absent; inner staminodes absent, but sometimes innermost stamens only 3–4-locellate; staminal cone 0.8–0.9 mm in diameter, 0.4–0.5 mm high, concealing only the bases of the ovaries, rim laciniate. ***Carpels*** 2–4; ovaries 1.5–1.7 mm long, narrowly oblong, sericeous, stigmas slightly separated, 0.7–1.1 mm long, lanceolate-falcate, flattened and longitudinally grooved on inner surface, glabrous. ***Torus*** flat, 1.5–1.6 mm in diameter. ***Fruit*** of up to 3 sparsely pubescent to glabrate monocarps borne on a pedicel 4.5–6 mm long, 1.1–1.4 mm thick, sparsely pubescent to glabrate, with sepals and sometimes persistent; torus not expanded in fruit, obscured by sepals. ***Monocarps*** with yellow-green exterior marked with contrasting green raised venation *in vivo*, 2–2.7 cm long, ca. 1.1 cm wide, 0.4–0.5 cm thick, oblong, not or only weakly torulose, apex truncate, obliquely mucronate, the beak 1–2 mm long, base contracted into a stipe 5–8.5 mm long, 1.1–1.6 mm thick, with raised veins forming oblique ridges and otherwise slightly wrinkled and finely verrucose; pericarp 0.5–0.6 mm thick. ***Seeds*** 2–4 per monocarp, in a single row, lying oblique or perpendicular to long axis, 8.5–9 mm long, 5.5–6 mm wide, ca. 5.4 mm thick, ellipsoid, broadly elliptic in cross section, truncate at micropylar end, rounded at chalazal end, light brown, smooth, shiny, raphe/antiraphe plane, visible as a band encircling the seed, micropylar scar 2.2–2.5 mm long, 2.0–2.5 mm wide, elliptic to circular; sarcotesta absent; aril white *in vivo*, light brown when dried, cupular, 4–5 mm in diameter, 1.7–2.2 mm high, fleshy, smooth.

#### Phenology.

Specimens with flowers were collected from February to May, and with fruits from February to June. We observed no definable peak of flowering in the field but only a few flowers at anthesis were present at any time on a given tree during the flowering period. Similarly, very few fruits could be found on an individual tree at any one time. Vegetative growth seemed to have been stimulated by the short rains of November and December, with production of flower buds following shortly thereafter.

#### Distribution

(Fig. 19). Occurs in east-central Tanzania, in dry evergreen forest on small ridgetops and plateaus near the coast, with *Scorodophloeusfischeri* and *Manilkarasulcata* being frequent canopy associates; 0–300 m. Understory associates in the Pugu Forest Reserve include *Lasiodiscusholtzii*, *Uvariapandensis*, *Uvariapuguensis*, and species of *Hugonia*, *Hymenocardia*, *Landolphia*, *Ochna*, and *Suregada*.

#### Local names.

Mgwaza, mgwaza dume (Kizaramo), mlambambulu (Kiswahili), nnoga (Kiswahili).

#### Additional specimens examined.

**TANZANIA.** Pwani: Ruvu South Forest, Kisarawe District, 6°58'S, 38°54'E, 260 m, 30 mi SW of Dar es Salaam, 12 Feb–14 Mar 1991 (fr), *Frontier Tanzania 2128* (K); Pande, 20 Jul 1982 (st), *Hawthorne 1360* (K), *Hawthorne 1714* (K); Pande, W. edge, 19 Aug 1982 (buds), *Hawthorne 1469* (K); Kisiju, by the sea, *Hawthorne 1790* (DSM); Kisarawe District, Pugu Forest Reserve, ridges along north road between Pugu railway station and brick works, 6°52'30"S, 39°06'E, 29 Feb 1996 (fl, fr), *Johnson & Mwasumbi 1899* (DSM, OWU); Kisarawe District, Pugu Forest Reserve, along N road 0.5 km E of brick factory, 6°52'S, 39°06'E, 2 Apr 1996 (fl, fr), *Johnson 1920* (DSM, OWU), 24 Apr 1996 (fl), *Johnson 1928A* (OWU, spirit collection only); Kisarawe District, Pugu Forest Reserve, ridgetop over road tunnel, 6°52'30"S, 39°05'30"E, 6 May 1996 (fl), *Johnson & Mwasumbi 1936* (DSM, OWU); Kisarawe District, Pugu Forest Reserve, S of Dar–Kisarawe road, ridgetop near Mwakanga railway station, 6°55'S, 39°06'E, 22 Jun 1996 (fl, fr), *Johnson & Ndangalasi 1948* (DSM, OWU); Bagamoyo District, Zaraninge Forest Reserve, 54 km E of Hwy, 6°04–13'S, 38°35–42'E, 28 Jun 1996 (buds), *Johnson & Mbago 1963* (DSM, OWU); Kisarawe District, Pugu Forest Reserve, bus roundabout area ca. 4 km E of Kisarawe, 06°53'30"S, 39°06'E, 29 Jun 1996 (fr), *Johnson 1964B* (DSM, OWU); Kisarawe District, Kazimzumbwi Ruvu South [sic], *Magogo 618* (NHT, TFD); T6, Ruvu South Forest Reserve, Ufang’ombe area, 06°56'S, 38°49'E, 180 m, 30 Apr 2001 (st), *Mwangoka & Saidi 2099* (MO); Kisarawe District: Banda Forest Reserve near Mfyoza village, 12 Nov 1969 (buds), *Ruffo 301* (EA, K, NHT, TFD); Pande, *Rulangaranga et al. 53* (K, MO); Kisarawe District, Pugu Forest Reserve, 10 Mar 1964 (buds), *Semsei 3704* (EA, K, TFD); Kisarawe District, Banda Forest Reserve, 12 Nov 1969 (buds), *Shabani 471* (EA, K, TFD); Pande Forest Reserve, 25 km WNW of Dar [es Salaam], 8 Feb 1975 (fl), *Wingfield 3311* (DSM, EA, K).

An inconspicuous plant, *Xylopiamwasumbii*, was first recognized as *Xylopia* “species B” by Verdcourt (1971b). It is most similar to *X.tenuipetala* from northeastern Mozambique, but the leaf blades are usually elliptic to broadly elliptic, with cuneate bases and blunt-acuminate to obtuse apices, the pedicels and petals are shorter, and the ovaries are pubescent. These two species have some of the lowest stamen numbers, 50 or fewer, of any African species.

For *X.mwasumbii*, we calculated an EOO of 3,702 km^2^ and an AOO of 60 km^2^; there are only four other African species with a smaller global distribution. It was categorized as Endangered in the IUCN Red List, version 3.1 (IUCN Species Survival Commission 2012), with a conservation assessment of B1ab(iii), Decreasing. The dry evergreen coastal forests where *Xylopiamwasumbii* grows are still poorly known and very limited in extent. Within this rare forest formation, however, the species may be more common than is currently recognized: its green-tinted flowers and fruits are inconspicuous, and its principal flowering and fruiting occur largely during the long wet season. *Xylopiamwasumbii* also has a strong resemblance to species of *Diospyros* and, in herbarium material, to other genera of Annonaceae such as *Sphaerocoryne* and *Toussaintia* and may thus be misidentified in collections. The flower-galls, described by Verdcourt (1971b), seem to be a frequent feature of the species, and may be useful for field identification.

### 11. Xylopia tenuipetala

Taxon classificationPlantaeMagnolialesAnnonaceae

D.M. Johnson & Goyder, Kew Bull. 72:11: 3, 5, 7–8. 2017.

F7D74A64-1B7A-585C-B6F2-278A1F81B81A

Figs 3B , 4D , 20

#### Type.

MOZAMBIQUE. Cabo Delgado Province, ca. 1 km W of Quiterajo airstrip on track towards Namacubi Forest, Waypoint JT 627, 11°45'58.9"S, 40°23'59.8"E, 88 m, 21 Nov 2009, *D. J. Goyder et al. 6090* (holotype K!; isotypes LMA, LMU, P).

#### Description.

***Shrub or tree*** up to 8 m tall; bark gray, smooth. ***Twigs*** green to brownish gray, glabrous, with epidermis soon exfoliating; nodes occasionally with two or more axillary branches. ***Leaf*** with larger blades 4.6–5.7 cm long, 2.3–2.7 cm wide, chartaceous to subcoriaceous, concolorous, lanceolate to ovate, apex gradually acuminate with an acumen 4 –11 mm long, base broadly cuneate to rounded, glabrous on both surfaces; midrib plane adaxially, raised abaxially, secondary veins weakly brochidodromous; 9–12 per side, diverging at 60–70° from the midrib, these and higher-order veins slightly raised on both surfaces; petiole 3.5–5 mm long, nearly terete to shallowly canaliculate adaxially, glabrous. ***Inflorescences*** axillary, rarely pseudoterminal from abortion of the terminal bud, 1-flowered, glabrous; peduncle rudimentary; pedicels 6.7–7.2 mm long; bracts 2, persistent, the lower one proximal to the pedicel midpoint, 0.7 – 0.8 mm long, ovate, the upper one distal to the pedicel midpoint, 0.9–1.4 mm long, broadly ovate; buds lanceolate, apex acute. ***Sepals*** slightly spreading at anthesis, 3/5-connate, 1.7–2.6 mm long, 2.1–2.5 mm wide, chartaceous, broadly ovate, apex acute, glabrous abaxially. ***Petals*** yellow-green *in vivo*; outer petals spreading at anthesis, 13–16 mm long, 2.9–3.4 mm wide at base, 1.6–1.9 mm wide at midpoint, membranous with venation evident, lanceolate, flat, shallowly concave adaxially, apex acute, sparsely pubescent in the center, becoming more densely pubescent toward margins adaxially, appressed-pubescent abaxially; inner petals erect at anthesis, 9.0–10.5 mm long, 1.8–2.1 mm wide at base, 1.2–1.7 mm wide at midpoint, membranous with venation evident, linear-lanceolate, apex acute, base with undifferentiated margin, glabrous. ***Stamens*** ca. 50; fertile stamens 1.2–1.5 mm long, narrowly oblong, apex of connective minute, truncate, not exceeding anther thecae, glabrous, anthers 8–10-locellate, filament 0.5–0.7 mm long; outer staminodes absent; inner staminodes 1–1.1 mm long, oblong, apex truncate; staminal cone ca. 0.8 mm in diameter, ca. 0.6 mm high, concealing only the bases of the ovaries, rim laciniate. ***Carpels*** 2–5; ovaries 1.1–1.5 mm long, narrowly oblong, glabrous; stigmas loosely appressed, 1.1–1.2 mm long, lanceolate-falcate, apex acute, glabrous; ***Torus*** flat, ca. 1 mm in diameter. ***Fruit*** of up to 2 glabrous monocarps borne on a pedicel ca. 6.3 mm long, ca. 1.3 mm thick, glabrous; torus 2.8 mm in diameter, 1.5 mm high, globose. ***Monocarps*** green with a red endocarp *in vivo*, ca. 1.8 cm long, ca. 0.9 cm wide, ca. 0.5 cm thick, oblong, flattened-ellipsoid in cross section, apex truncate and obliquely mucronate (1.5 mm), base contracted into a stipe ca. 4 mm long, 1.4 mm thick, finely verrucose; pericarp ca. 0.3 mm thick. ***Seeds*** 4–5 per monocarp, in a single row, lying perpendicular to long axis, 7.1–7.9 mm long, 4.4–4.7 mm wide, 3.3–3.7 mm thick, oblong-ellipsoid, elliptic to oblong in cross section, obliquely truncate at micropylar end, rounded at chalazal end, blue-gray to black, smooth, shiny, raphe/antiraphe not evident, micropylar scar 1.2–1.5 mm in diameter, circular; sarcotesta absent; aril white *in vivo*, amber-colored when dried, cupular, 3.9–4.4 mm in diameter, 2.0–2.4 mm high, fleshy, smooth.

#### Phenology.

Specimens with flowers have been collected in November, the one specimen with fruit was collected in December.

#### Distribution

(Fig. 19). Endemic to northeastern Mozambique, where it occurs in coastal dry sand forest at elevations of 65–134 m.

#### Additional specimens examined.

**MOZAMBIQUE.** Cabo Delgado: Namacubi Forest (the Banana), W of Quiterajo, 11°45'55"S, 40°23'45"E, 25 Nov 2008 (st), *Burrows & Burrows 10746* (BNRH, K); Quiterajo, within Namacubi Forest, W of Quiterajo, 11°45'47.0"S, 40°21'14.8"E, 29 Nov 2008 (fl), *Crawford et al. FC265* (K—2 sheets); Palma area 1 km E of Muangaza, S of Palma, 10°55'24.5"S, 40°23'34.8"E, 65 m, 5 Dec 2008 (fr), *Goyder et al. 5089* (K); Quitarajo Pt 463, 11.7676S, 40.3743E, 24 Nov 2009 (fl), *Luke & Luke 13884* (EA, K, LMA, P); Macomia District, Quiterajo, Namparamnera forest, 11°49'03.8"S, 40°20'33.1"E, 29 Nov 2008 (fl), *Timberlake et al. 5570* (K-738101).

This poorly known species resembles *Xylopiamwasumbii*, which occurs to the north of its range in central Tanzania, but in *X.tenuipetala* the leaf blades are lanceolate to ovate, broadly cuneate to rounded at the base and acuminate at the apex, the pedicels and petals are longer, and the ovaries are glabrous. The petals of *X.tenuipetala* are unusual in the genus—thin, membranous, and translucent rather than coriaceous or fleshy. It occurs in the same area of high plant endemism as *X.lukei* (Timberlake et al. 2011). A conservation assessment of Endangered was assigned to it in Johnson et al. (2017; Table 1).

### V. Xylopia Section Stenoxylopia

Taxon classificationPlantaeMagnolialesAnnonaceae

Engler & Diels, Monogr. afrik. Pflanzen-Fam. 6: 59. 1901.

85CC9676-03F1-5694-84E7-8614FE4AD102

#### Type.

*Xylopiaodoratissima* Welwitsch ex Oliver (lectotype, designated in Stull et al. 2017, p. 221).

#### Description.

Nodes with branches from 2–3 axillary buds; outer petals lanceolate to linear, rarely ovate; inner petals lanceolate to linear, rarely ovate, fleshy basal margins absent or present; anther connectives shieldlike at apex, overhanging the anther thecae; staminal cone rudimentary to well-developed, partially to completely concealing the ovaries, rim even to irregularly laciniate; carpels 3–20, the stigmas connivent, smooth; arils absent; seed coat smooth, rarely slightly papillate, pitted, or wrinkled, sarcotesta present, either orange to red or pale green, blue, or gray.

#### Notes.

Members of this section are separated here into two informal groups, named for an exemplar species in each. The species of the *Xylopiaodoratissima* group (Species 12–25) all have an orange sarcotesta, and the group includes all of the African dryland species. The distribution of this group extends from Mozambique, the southern limit of the genus in Africa, north to Sudan and west to southeastern Cameroon (Fig. 8). The *Xylopiaacutiflora* group (Species 26–45) includes all African species known to have a sarcotesta that is light green, blue, or gray in color, as well as one species with an orange sarcotesta. Members of this group are found from Senegal in West Africa east to South Sudan and south to central Angola and western Tanzania.

The two groups are not given formal taxonomic status in this revision. While the *Xylopiaacutiflora* group was strongly supported as a monophyletic group in our phylogenetic analysis (Stull et al. 2017), the *Xylopiaodoratissima* group was not. The groups are separated here to call attention to biological and ecological differences between them and as an aid to species identification.

##### *Xylopiaodoratissima* group

Plants of the *Xylopiaodoratissima* group are mostly shrubs and understory trees, with only a few species reaching canopy size. The leaves are thin-textured and often hairy on one or both leaf surfaces. The inflorescences are 1–6-flowered, with each pedicel usually arising from the axil separately but occasionally arising from a common peduncle. The monocarps may be sessile or stipitate, and are usually thin-walled, with the seeds lying in a single row or rarely in two rows in a few species. The endocarp, visible in the dehiscent fruit, may be either pink or green. Seeds of all species have a thin sarcotesta that is orange to red and oily or greasy to the touch, but lack arils.

In Africa, the *X.odoratissima* group has a predominantly Zambezian distribution (Fig. 8). Three subgroups may be distinguished. *Xylopiagracilipes*, *X.holtzii*, *X.nilotica*, *X.odoratissima*, *X.shirensis*, and *X.torrei* share petals of both whorls that are laxly spreading or rarely recurved at anthesis and monocarps with a green endocarp. In the *X.arenaria* subgroup, consisting of *X.arenaria*, *X.collina*, *X.keniensis*, and *X.tomentosa*, the outer petals are erect at anthesis, with the inner petal apices emerging at right angles between the outer petals. The fruit endocarp is pink. In the *X.gilbertii* group, which includes *X.flamignii*, *X.gilbertii*, and *X.toussaintii*, the petals of both whorls are relatively broad and only weakly separating at anthesis and the inner petals bear circular glands at the base. The endocarp color in this group is unknown. Isolated within the group but included because of its overall floral morphology and sarcotesta color is *X.wilwerthii* from the lower Congo River Basin.

### 12. Xylopia arenaria

Taxon classificationPlantaeMagnolialesAnnonaceae

Engler, Bot. Jahrb. Syst. 34: 159. 1904.

2A8F25C1-3153-5515-9C5C-62B5578FAECF

Figs 3H , 21B, D, E, G

#### Type.

TANZANIA [“Sansibarküstengebiet”]. Dar-es-Salaam Region, Dar-es-Salam, in parkartigem Buschgehölz des Sachsenwaldes auf Sandboden, Nov 1902, *A. Engler 2173* (lectotype, here designated: B! [10 01153133]).

#### Description.

***Shrub or small tree*** up to 6 m tall, secondary branches drooping; bark gray, sometimes blotched with white, smooth. ***Twigs*** reddish brown to light gray, pubescent, the hairs 0.2–0.8 mm long, bark soon peeling and flaking in reddish brown strips, becoming glabrate; nodes occasionally with two axillary branches. ***Leaf*** with larger blades 3.4–7 cm long, 1.5–2.5 cm wide, chartaceous to subcoriaceous, concolorous to slightly discolorous, ovate to lanceolate-ovate, occasionally lanceolate, apex acute to obtuse, base broadly cuneate, rounded, or subcordate, short-decurrent on petiole, glabrous or with a few hairs on the midrib adaxially, appressed-pubescent abaxially; midrib plane or slightly raised adaxially, raised abaxially, secondary veins indistinctly brochidodromous, 7–12 per side, diverging at 50–70° from the midrib, these and higher-order veins plane or slightly raised on both surfaces; petiole 2.5–5 mm long, shallowly canaliculate, pubescent. ***Inflorescences*** axillary or from axils of fallen leaves, 1(–2)-flowered, pubescent; pedicels not pedunculate, 1.8–3.5 mm long, 0.7–1.3 mm thick; bracts 3, evenly spaced along pedicel, persistent, 0.5–1.6 mm long, ovate, semicircular, or crescent-shaped, the upper two often bilobed, apex obtuse to acute; buds ovoid-conic, apex obtuse or less frequently acute. ***Sepals*** erect to slightly spreading at anthesis, 1/3–1/2-connate, 2.2–2.5 mm long, 2.1–2.7 mm wide, coriaceous, ovate to broadly ovate, apex acute, pubescent abaxially. ***Petals*** pale orange-yellow, cream, or white, the inner petals purplish red or rose-colored inside at the base *in vivo*; outer petals erect at anthesis, 6.6–10.2 mm long, 2.7–3.2 mm wide at base, 1.8–2.0 mm wide at midpoint, coriaceous or a little fleshy, lanceolate, apex acute, densely puberulent except for glabrous basal patch adaxially, densely appressed-pubescent abaxially; inner petals geniculate at anthesis, with the apices bent sharply outward between the outer petals, 4.7–7.2 mm long, 1.8–2.7 mm wide at base, 0.8–1.2 mm wide at midpoint, slightly fleshy, lanceolate-acuminate, apex acute, base with undifferentiated margin but transversely thickened at the widest point adaxially, puberulent except for glabrous base adaxially, puberulent on apex and medial portion of base but otherwise glabrous abaxially. ***Stamens*** 70–200; fertile stamens 1.0–1.2 mm long, narrowly oblong, apex of connective purplish red *in vivo*, 0.1–0.3 mm long, truncate to capitate, overhanging anther thecae, glabrous or minutely papillate, anthers 12–18-locellate, filament 0.2–0.3 mm long; outer staminodes 1.0–1.2 mm long, clavate to oblong, apex acute, obtuse, or truncate; inner staminodes 0.8–0.9 mm long, oblong, apex obtuse, rounded, or truncate; staminal cone 1.4–1.6 mm in diameter, 0.9–1.2 mm high, concealing only the bases of the ovaries, rim laciniate. ***Carpels***6–11; ovaries ca. 1 mm long, narrowly oblong, sericeous, stigmas connivent, ca. 2.3 mm long, trowel-shaped, bearing a tuft of hairs at the apex. ***Torus*** flat, 1.5–1.8 mm in diameter. ***Fruit*** of up to 4 glabrate monocarps borne on a pedicel 4.2–5.3 mm long, 1.2–1.6 mm thick, sometimes with bracts persistent, pubescent; torus of fruit 1.9–2.3 mm in diameter, 0.9–1.8 mm high, depressed-globose. ***Monocarps*** with light green exterior and scarlet endocarp *in vivo*, 1.3–2.5 cm long, 0.5–1.2 cm wide, ca. 0.5–0.7 cm thick, oblong, weakly torulose, apex rounded with an oblique blunt beak 1–2 mm long, base contracted into a stipe 3–5 mm long, 1.5–2.1 mm thick, smooth or occasionally somewhat verrucose; pericarp ca. 0.2 mm thick. ***Seeds*** 1–3 per monocarp, in a single row, lying oblique to long axis, 9.2–9.8 mm long, 5.3–5.8 mm wide, 4.5–6.3 mm thick, pyriform, narrowed toward micropyle into a cylindrical neck 2.3–2.8 mm long and 2.8–3.6 mm wide, broadly elliptic in cross-section, obliquely truncate at micropylar end, rounded at chalazal end, brown, smooth, shiny, raphe/antiraphe not evident, micropylar scar 1.2–1.6 mm long, 0.8–1.0 mm wide, elliptic; sarcotesta bright orange, fleshy *in vivo*; aril absent.

**Figure 20.**
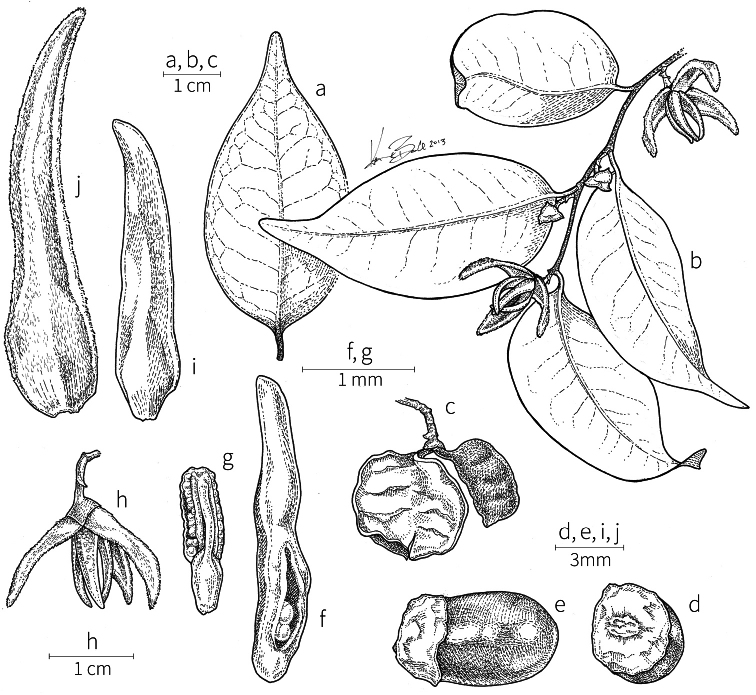
*Xylopiatenuipetala*. **A** Leaf **B** Habit **C** Fruit, showing one dehisced and one undehisced monocarp **D** Seed, view of micropylar end **E** Seed, side view **F** Carpel, with ovary dissected to show two ovules **G** Stamen, abaxial view **H** Flower **I** Inner petal, adaxial view **J** Outer petal, adaxial view. **A, F–J** from *Goyder et al. 6090* (K) **B** from photograph of same *in vivo***C–E** from *Goyder et al. 5089* (K). Reproduced with the permission of the Board of Trustees, Royal Botanic Gardens Kew.

**Figure 21.**
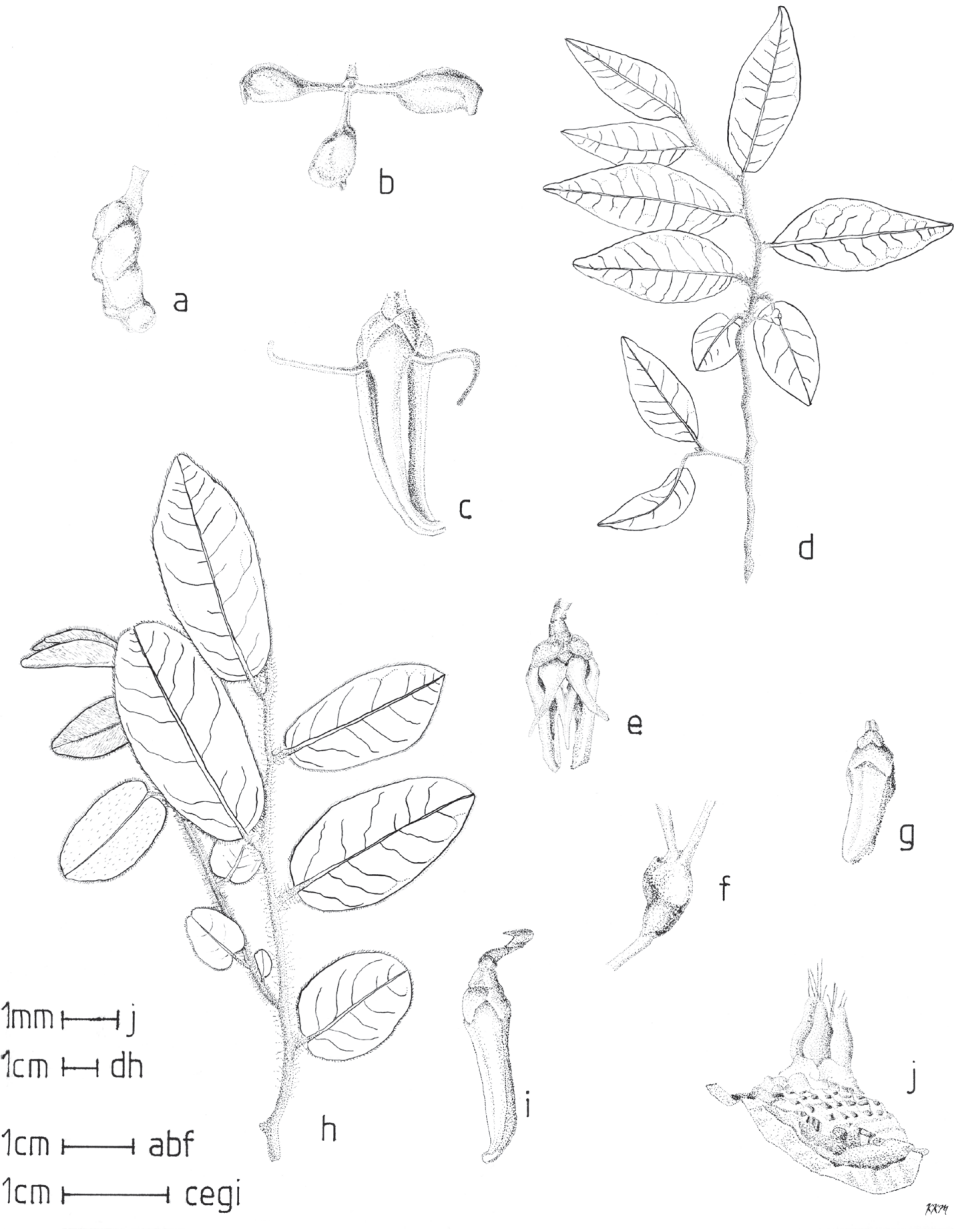
*Xylopiacollina* and *X.arenaria*. **A, C, F, H, I, J***X.collina*. **A** Monocarp, lateral view **C** Flower, lateral view **F** Stem gall **H** Habit **I** Bud **J** Torus with attached ovaries, lateral view **B, D, E, G***X.arenaria***B** Fruit **D** Habit **E** Flower bud with one outer petal removed, lateral view **G** Bud. **A, F, H** from *Johnson et al. 1914* (OWU) **B** from *Johnson & Murray 1890* (OWU) **C, I, J** from *Johnson et al. 1913* (OWU) **D, E, G** from *Johnson et al. 1937* (OWU).

#### Phenology.

Specimens with flowers have been collected in February, May to September, November, and December, and with fruits in February, March, June, July, and November.

#### Distribution

**Figure 22.**
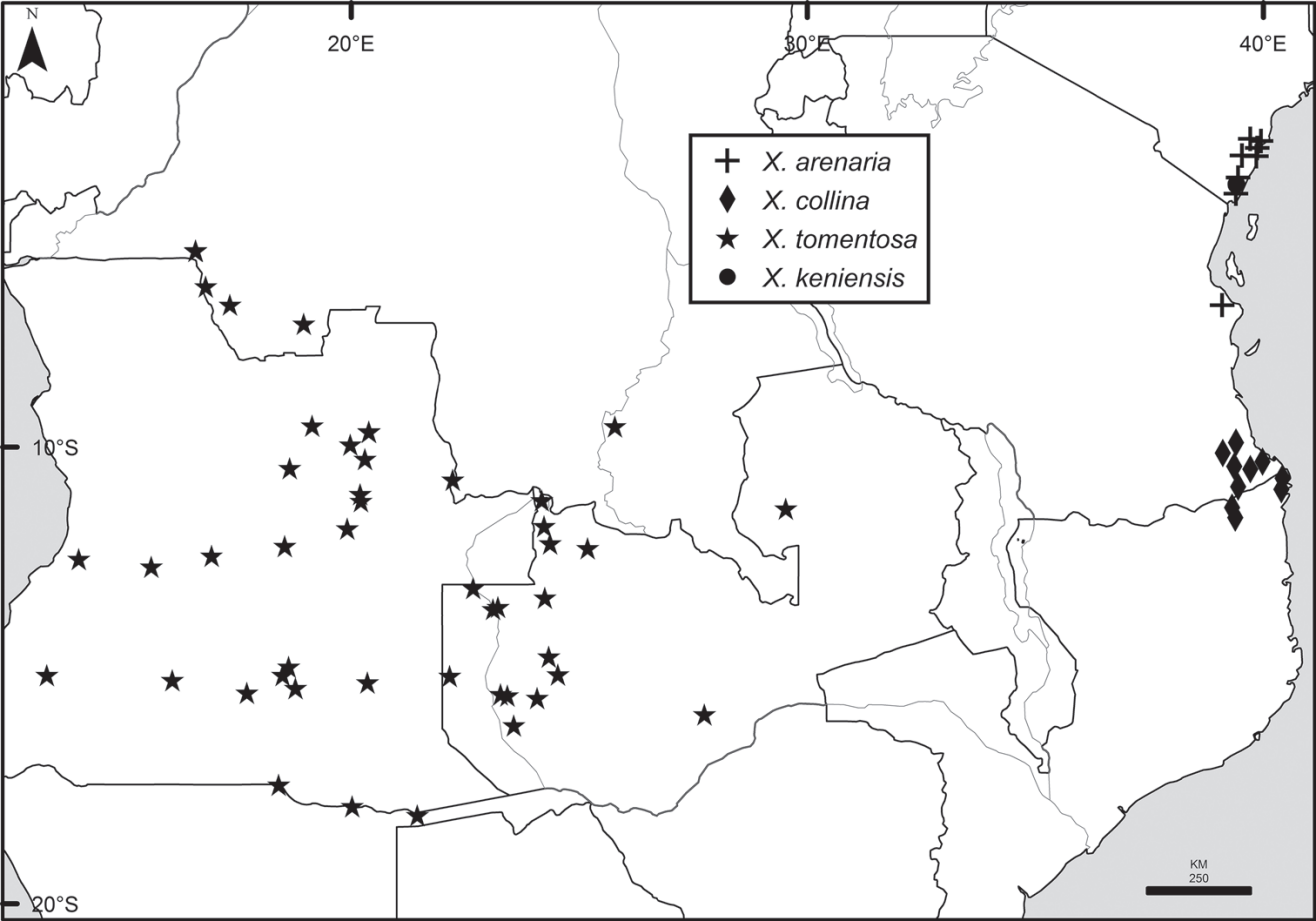
Distributions of *Xylopiaarenaria*, *X.collina*, *X.tomentosa*, and *X.keniensis* in southern and eastern Africa. Bolder lines represent country borders, fainter lines lakes and major rivers.

(Fig. 22). Occurs along the East African coast, from central Kenya south to central Tanzania, growing in forests and bushland, sometimes in secondary vegetation and usually on sandy soil, at elevations of 30–500 m.

#### Local names.

Mkabui (*Holtz 321*), mtuma-mrihi (Girama, *Trump 96*), mukunambawa (Girama, *Langridge 58*, *Moggridge 389*).

#### Additional specimens examined.

**KENYA.** Kilifi: Sokoke Forest, road to Jilore Forest Station, 3.2 km from turnoff on Kilifi-Malindi Road, *Faden 71/696* (EA); Sokoke Forest, ca. 3 km on track S of Gede to Jilore Forest Station, ca. 3°18'S, 39°57'E, *Faden & Faden 74/1225* (EA, K, MO, WAG); Sokoke Forest, *Gisau 10* (EA, K), *Langridge 58* (EA); K7, Mangea Hill summit, 03°15'S, 39°43'E, 500 m, 8 Apr 1987 (st), *Luke & Robertson 318* (EA); K7, Mangea Hill, 3942E0316S, 480 m, 18 Feb 1988 (buds), *Luke & Robertson 1008* (EA); 20.2 km Gotani to Bamba, 3°37'S, 39°32'E, 21 Nov 1989 (fl), *Luke & Robertson 2134* (EA, K); Kilifi, 17 May 1937 (fl), *Moggridge 389* (EA, K); Arabuko-Sokoke Forest, N of Sokoke Forest Station, 3°25’-3°30'S, 39°50-39°54'E, 8 Jun 1973 (fl), *Musyoki & Hansen 1007* (EA, K); forest 8 mi S of Jilore Forest Station, near road to Mida, 3°12'S, 39°55'E, *Perdue & Kibuwa 10046* (EA, FI-T, K); Arabuko Sokoke Forest, 3°17'S, 39°56'E, 26 Dec 1983 (fl), *Robertson 3727* (EA, K, MO); 4.5 km from Bamba toward Ganze, 3°33'S, 39°34'E, *Robertson & Luke 6063* (EA); Mangea Hill summit, 3°15'S, 39°43'E, *Robertson & Brummitt 6752* (K); Jilore-Mida, SW of Malindi, 3°19'S, 39°58'E, 18 Mar 1973 (st), *Sangai EA 15703* (EA); Sokoke Forest, Kilifi, Coast Province, 26 Mar 1954 (fr), *Trump 96* (EA, K).—Kwale: Mrima Hill, 7 Dec 1975 (fl), *Kokwaro 3951* (K); Buda Mafisini F. R., 04°27'S, 39°24'E, 70 m, 23 Feb 1989 (st), *Luke & Robertson 1686* (EA, K); Maluganji Forest Reserve (including Kaya Mtae), 04°06'S, 39°27'E, 200-300 m, 14 Nov 1989 (fl), *Robertson & Luke 6000* (EA). **TANZANIA.** Pwani: Pugu Hills Forest Reserve, Kisarawe, 23 Aug 1972 (fl), *Harris et al. DSM 2606* (MO); Dar es Salaam airport, 13 Sep 1970 (fl), *Harris & Harris 4989* (B, MO); Pugu Hills near Kisarawe, 6°52–53'S, 39°04–06'E, 2 Nov 1970 (st), *Harris & Schlieben BJH 5346* (DSM, EA, WAG); Pugu F. R., near Kisarawe, 31 Jul 1971 (fl), *Harris et al. 5859* (MO); Kisarawe District, Pugu Hills Forest Reserve, *Harris BJH 6771* (DSM), *Harris & Siddiqi BJH 6749* (DSM); Pugu Forest Reserve, *Hawthorne 1750* (K–2 sheets); Dar es Salaam Station, Sachsenwald, 30 Nov 1901 (fl bud, fr), *Holtz 321* (EA); Dar es Salaam, 6 Dec 1901 (fl), *Holtz 393* (EA, PRE); Kisarawe District, Pugu Forest Reserve, bus roundabout area ca. 4 km E of Kisarawe, 6°53'30"S, 39°06'E, 27 Feb 1996 (fl, fr), *Johnson 1890* (DSM, OWU), 26 May 1996 (fl), *Johnson & Murray 1937* (DSM, OWU); Pugu Forest Reserve, 6°54'18"S, 39°04'59"E, 270 m, 20 Aug 2003 (st), *Kibure et al. 1019* (MO); Kisarawe District, Pugu Forest Reserve, 23 Aug 1972 (fl), *Mabberley & Harris 1516* (K, WAG); Pugu Forest Reserve, E part of forest between Minaki Secondary School and Pugu Kajiungeno, 06°53'39"S, 39°06'00"E, 180 m, 2 Jun 2012 (fl), *Mwangoka & Mulungu 7959* (MO); Kisarawe District, Kisarawe Forest Reserve, *Paulo 118* (EA, K); Kisarawe District, Mogo Forest Reserve, Aug 1953 (fl), *Semsei 51285* (EA).

This narrowly distributed species has distinctive long hairs on the twigs and leaves, ovate to lanceolate leaves that are acute at the apex, and usually solitary flowers with blunt buds. It shares with *X.collina*, *X.keniensis*, and *X.tomentosa* the characteristic of the tips of the inner petals bending sharply outward at anthesis, such that they emerge through the gaps between the erect outer petals. Description of the floral scent is varied, from over-ripe bananas to “smell of ‘Annonaceae’ with overlying smell of cats” (*Mabberley & Harris 1516*).

A specimen from Tanzania, *Abeid et al. 892* (MO), Pwani, Rufiji District, Kichi Hill Forest Reserve, NW of Nawanje village, Miselu/Mkinga Peak, 8°12'55"S, 38°38'55"E, 594 m, 27 Apr 2001 (fl), has peeling twigs and leaves similar in shape to those of *X.arenaria*, but the flowers were said to be purple, the outer petals are 4 mm wide at the base, and the specimen was taken from a tree 9 m tall growing at an elevation of 600 m. Fieldwork is needed to determine whether this plant is simply an aberrant specimen of *X.arenaria* or represents a distinct species.

Despite being locally common in the Sokoke Forest area of Kenya and the Pugu Hills of Tanzania, *Xylopiaarenaria* has yet to be collected from any area in between these two localities. The species grows in *Brachystegiaspiciformis* woodland, and in lowland evergreen forests dominated by other ectomycorrhizal caesalpinioid legumes such as species of *Afzelia*, *Cynometra*, *Julbernardia*, *Paramacrolobium*, *Erythrophloeum*, and *Hymenaea*.

Other type material of the name *Xylopiaarenaria* was not located, and the B sheet of *Engler 2173*, with good correspondence to the protologue, is formally designated here as a lectotype.

### 13. Xylopia collina

Taxon classificationPlantaeMagnolialesAnnonaceae

Diels, Notizbl. Bot. Gart. Berlin-Dahlem 13: 271–272. 1936.

3AD1D651-7D69-56BD-9EFE-DA1DB676CF05

Figs 3I , 4E , 21A, C, F, H, I, J

Xylopia latipetala Verdcourt, Kew Bull. 25(1): 12. 1971. Type. TANZANIA. Lindi District: Rondo Plateau, Nahoro in Albizia, Chlorophora woodland, on old termite-hill, 11 Dec 1955 (fl), *Milne-Redhead & Taylor 7617* (holotype: K—2 sheets!).

#### Type.

TANZANIA. Lindi Region, 40 km westlich von Lindi, Lutambasee [“Lumbatasee”], 14 Oct 1934, *H.-J. E. Schlieben 5470* (lectotype, here designated: B! [10 0153136]; isotypes: B! [100153137, 100153138, 100153139], BM! [000511056], BR! [0000008824783], G! [00190716], HBG [502482, 502483, 502484], K! LISC! [000402], M! [0107918], MA! [385126], MO! [1703951], P! [00363256], PRE! [0397107-0], S! [07-13459], US! [2214646]).

#### Description.

***Shrub or small tree*** up to 4 m tall, d.b.h. up to 4 cm, twigs often somewhat flexuous on new growth. ***Twigs*** brown to reddish brown, densely erect-pubescent, the hairs 1–2 mm long, becoming gray-brown, glabrate or with the hairs somewhat persistent, bark developing minute exfoliations on the leafless growth; nodes occasionally with two axillary branches. ***Leaf*** with larger blades 4–8.4 cm long, 2.1–4.4 cm wide, chartaceous, concolorous or slightly discolorous, elliptic to ovate, occasionally lanceolate or obovate, apex acute to rounded, base subcordate or rounded, uniformly pubescent with hairs persistent on both surfaces and on the margin, slightly denser on the midrib; midrib plane to slightly raised adaxially, slightly raised abaxially, secondary veins indistinctly brochidodromous, 6–13 per side, diverging at 35–70° from the midrib, these and higher-order veins slightly raised on both surfaces; petiole 2–4 mm long, shallowly canaliculate, pubescent. ***Inflorescences*** axillary, 1-flowered, pubescent; pedicels not pedunculate, 1–3 mm long, 0.6–1.5 mm thick; bracts 2, evenly spaced along pedicel, caducous or rarely persistent, 1–2.7 mm long, semicircular or crescent-shaped, apex obtuse; buds lanceolate to ovoid, apex acute. ***Sepals*** erect to slightly spreading at anthesis, 1/5–1/2-connate, 2.5–4.1 mm long, 2.5–3 (–4.8) mm wide, coriaceous, ovate, apex acute, pubescent abaxially. ***Petals*** dull red to grayish pink *in vivo*; outer petals erect at anthesis, 9–20.4 mm long, 2.5–8 mm wide, 1.4–3.9 mm wide at midpoint, coriaceous to fleshy, linear-lanceolate to lanceolate, apex acute, densely puberulent on both surfaces except for a glabrous patch at base adaxially; inner petals geniculate at anthesis, with the apices bent sharply outward between the outer petals, 6–15.4 mm long, 2–5.2 mm wide, wide, 0.4–2.3 mm wide at midpoint, coriaceous to fleshy, lanceolate-acuminate, apex acute, base with undifferentiated margin, transversely thickened at the widest point adaxially, bearing a band of corrugated tissue just above the claw, puberulent except for glabrous base adaxially, puberulent on narrow apical portion and medial region of base but otherwise glabrous abaxially. ***Stamens*** 120–200; fertile stamens 0.8–1.4 mm long, narrowly oblong to clavate, apex of connective 0.1–0.3 mm long, shieldlike or slightly hemispheric, overhanging the anther thecae, papillate, anthers 10–17-locellate, filament 0.3–0.4 mm long; outer staminodes 1.0–1.3 mm long, clavate, apex truncate, sometimes oblique; inner staminodes 0.8–0.9 mm long, clavate to oblong, apex rounded to truncate; staminal cone 1.7–2.8 mm in diameter, 1.0–1.7 mm high, concealing only the bases of the ovaries, rim laciniate. ***Carpels*** 3–7; ovaries ca. 1 mm long, oblong, sericeous, stigmas connivent, ca. 1.3 mm long, lanceolate to trowel-shaped, bearing a tuft of hairs at the apex. ***Torus*** flat, 2.5–3.2 mm in diameter. ***Fruit*** of up to 6 glabrate monocarps borne on a pedicel 3–7 mm long, 1.2–2 mm thick, pubescent, with sepals persistent; torus 2.5–4 mm in diameter, 2.0–2.2 mm high, globose to depressed-globose. ***Monocarps*** with yellowish or glaucous green exterior and scarlet endocarp *in vivo*, 1.3–3.6 cm long, 0.6–1.3 cm wide, (0.5–) 0.7–1.2 cm thick, oblong, irregularly torulose, apex obtuse to acute, rarely mucronate, contracted into a stipe 3–10 mm long, 1.7–2.2 mm thick, surface pruinose, longitudinally wrinkled and finely verrucose; pericarp ca. 0.5 mm thick. ***Seeds*** up to 7 per monocarp, in two irregular rows, lying oblique to long axis, 9.8–12.2 mm long, 5.5–7.4 mm wide, 4.5–7.0 mm thick, pyriform, narrowed into a cylindrical neck 1.6–2.3 mm long and 2.7–3.5 mm wide toward micropyle, irregularly ellipsoid in cross section, obliquely truncate at micropylar end, rounded at chalazal end, brown, smooth, shiny, raphe/antiraphe not evident, micropylar scar 1.4–1.5 mm long, 1.0–1.1 mm wide, elliptic; sarcotesta bright orange, fleshy *in vivo*; aril absent.

#### Phenology.

Specimens with flowers have been collected in February, March, October, and December, and with fruits in June and from November to March.

#### Distribution

(Fig. 22). Restricted to southeastern Tanzania and northeastern Mozambique, where it grows in thickets and open woodland on slopes and plateau surfaces, usually on deep white sand and less commonly on loam, often locally common on roadsides and in areas mixed with planted cashew (*Anacardium*) trees, at elevations of 200–900 m.

#### Local names.

Mutuka (Kimakonde, *Busse 2858*), nampemedi (Kiswahili, *Johnson & Swedi 1914*), pempantavala (Kimuera, *Schlieben 5470*; the same common name was reported by Schlieben as being used for *Popowia* (now *Monanthotaxis*) *trichantha*, see Diels 1936).

#### Additional specimens examined.

**TANZANIA**. Lindi: Rondo Plateau, Rondo Forest Reserve, below Forest Station, 10°07'S, 39°14'E, 13 Feb 1991 (fl), *Bidgood et al. 1535* (DSM, EA, K, NHT), 13 Feb 1991 (fr), *Bidgood et al. 1544* (EA, K–2 sheets, MO, NHT); track from Nyengedi to Rondo Plateau, 10°12'S, 39°20'E, 16 Feb 1991 (fl), *Bidgood et al. 1600* (DSM, EA, K, NHT); [“Nordwestliches Muera-Plateau, etwa 600 m ü. M., in dichter Buschenklave auf sandigen Boden”, 14 Jun 1903 (fr), ex Diels 1936], *Busse 2858* (EA); Mchinjiri, Rondo Plateau, Nov 1951 (buds), *Eggeling 6386* (K, TFD); track from Nyengedi (between Masasi and Mnazimoja) to Rondo Plateau, ca. 3 km N of Nyengedi, 10°14'00"S, 39°24'00"E, 7 Mar 1996 (fl, fr), *Johnson & Swedi 1906*, 8 Mar 1996 (fl, fr), *Johnson & Swedi 1910* (DSM, OWU); Lindi District, Nyangamara Forest Reserve, 10°19'S, 39°59'E, 10 Mar 1996 (fl, fr), *Johnson & Swedi 1914* (DSM, OWU); Noto Plateau, N of Chitoa Plateau, 09°54'S, 39°24'E, 8 Jul 2008 (buds), *Mwangoka et al. 5801* (OWU); Lindi District, Rondo Forest Reserve, 21 Jan 1968 (fr), *Shabani 43* (EA, TFD); Lindi Rural District, Mtama Division, Namiupa Ward, Rondo Forest Reserve, NW of Mihima Village, 10°12'19"S, 39°09'21"E, 640 m, 3 Nov 2005 (fr), *Simon Laizer et al. 1352* (OWU).—Mtwara: Newala District, Mahuta, 16 Dec 1942 (buds), *Gillman 1055* (EA, K); Mnima, 23 Mar 1943 (fr), *Gillman 1293* (K); Newala District, road from Mtama to Mkwiti, ca. 2 km N of Mkwiti, 10°03'S, 40°11'E, 10 Mar 1996 (fl, fr), *Johnson & Swedi 1913* (DSM, OWU). **MOZAMBIQUE.** Cabo Delgado: Palma area, on old western road to Quionga, 10.8 km from the roundabout at the white church, 10°40'49"S, 40°26'11"E, 60 m, 12 Mar 2008 (fl buds), *Burrows & Burrows 10942* (K); Palma area, 1 km E of Muangaza S of Palma, 10°55'25.5"S, 40°23'34.8"E, 65 m, 5 Dec 2008 (fr), *Goyder et al. 5084* (K); Nanhamba, 7 km S of Mocimboa do Rovuma, 11°19'27"S, 39°18'55"E, 573 m, 10 Sep 2009 (fr), *Lötter & Turpin 1882* (K); Mueda Plateau, 11°24'S, 39°23'E, 860 m, 12 Dec 2003 (yg fr), *Luke et al. 10039* (K, MO); Niassa, Chomba, entre o cruzamento e Chomba, 20 Sep 1948 (st), *Pedro & Pedrogão 5279* (EA); Palma District, Miculumo area, 10 km NW of Palma, 10°40'39.9"S, 40°25'08.9"E, 56 m, 6 Dec 2008 (fl), *Timberlake et al. 5640* (K).

The red flowers of *Xylopiacollina* are unusual in the genus and, coupled with the shrub habit, the long erect hairs on the twigs and leaves, and the pruinose surface of the dried monocarps, make it a distinctive and easily recognized plant. With the examination of more extensive material, we do not maintain *Xylopialatipetala* Verdc. as a distinct species. The type specimen, *Milne-Redhead and Taylor 7617*, does indeed bear flowers with sepals and petals broader than in the type specimen of *X.collina*, but the gap in sizes presented in Verdcourt’s (1971a) description has now been filled by additional specimens: *Bidgood et al. 1535*, for example, has flowers with the outer petals 4.9–6.9 mm wide, i.e. intermediate in size between the two described species. We also observed considerable variation in petal size and shape within field populations of *X.collina*, suggesting that variation in petal size is a normal attribute of this species.

Although the flowers emit an ester/vinegar scent of rotting fruit during the middle of the day, suggesting small flies, nitidulid beetles, or thrips as possible flower visitors, no pollinators were found. Color cues may be important in determining the timing of pollinator behavior, as the flower buds are initially white, then become pink or pinkish orange, and finally turn pinkish red. The tips of the petals often become black or dark gray. A band of corrugated tissue crosses the adaxial surface of the inner petal at its widest extent and may provide a food reward for pollinators. As in *X.arenaria*, onset of anthesis is signalled by the bending of the apices of the inner petals so that they emerge through the gaps between the erect outer petals.

Associates observed at several sites included *Bosqueiopsisgilletii*, *Miliciaexcelsa*, *Grewiaconocarpa*, and species of *Albizia*, *Heinsia*, *Hymenocardia*, *Indigofera*, *Mimosa*, and *Pteleopsis*. In the area of a recent burn on the Rondo Plateau in March, 1996, *Xylopiacollina* was one of several Annonaceae, including also *Dielsiothamnusdivaricatus*, *Monanthotaxisbuchananii*, *Monodoraminor*, and *Uvariaacuminata*, observed to be regenerating from a rootstock after fire. The indurated black bud scales on the vegetative buds may play a role in allowing shoot regeneration after fire or drought; such bud scales are unusual in Annonaceae, where dormant shoot tips are typically naked. The growth of *X.collina* also exhibits distinctive stem galls (Fig. 20F) that are seen in other African species of *Xylopia*.

There are four sheets of the type collection, *Schlieben 5470*, at B. None is in exact agreement with the protologue concerning the type locality, but the sheet with detail drawings of the floral parts attached is chosen as the lectotype.

As shown in Table 1, the AOO for this species was relatively high (76 km^2^) for the size of its EOO (12,374 km^2^). We found *X.collina* to be locally frequent along roadsides, which may make it more frequently collected. In the IUCN Red List (version 3.1), it has been classified, however, as Endangered, largely due to the small size of its global distribution and local habitat alteration.

A decoction of the leaves of *Xylopiacollina* was reported by local informants in the Nyangamara region of southern Tanzania to be used as a treatment for hernia.

### 14. Xylopia flamignii

Taxon classificationPlantaeMagnolialesAnnonaceae

Boutique, Bull. Jard. Bot. État 21: 110. 1951.

40C1AA51-6448-5962-8FE0-5F959F653671

Fig. 23A–E

Xylopia lenombe Paiva, Mem. Soc. Brot. 19: 76 + pl. 12. 1966. Type. ANGOLA. Cabinda Province, Cabinda na Chiaca, 10 Jun 1960, *R. Monteiro & F. C. Murta 227* (holotype: LUAI; isotypes: BM! [0005109030], LUA, PRE! [0774857]).

#### Type.

DEMOCRATIC REPUBLIC OF THE CONGO [“Belgian Congo”]. Maï-Ndombe Province, Nioki, May 1941, *A. Flamigni 9026 ter* (holotype: BR! [0000008824745]; isotypes: BM! [000511044], BR! [0000008824745], K! [000199057], P! [00169149]).

#### Description.

***Tree*** up to 30 m tall. ***Twigs*** dark brown to gray brown, eventually light brown or gray, initially appressed-pubescent, the hairs 0.1–0.2 mm long, soon glabrate; nodes occasionally with two axillary branches. ***Leaf*** with larger blades 5.2–7.9 cm long, 1.3–2.7 cm wide, chartaceous, discolorous and shiny black adaxially, brown and dull adaxially, lanceolate to lanceolate-oblong, apex attenuate to short-acuminate with an acumen 7–8 mm long, base broadly and obliquely cuneate, glabrous or occasionally with a few hairs on the midrib adaxially, finely appressed-pubescent abaxially; midrib plane adaxially, raised abaxially, secondary veins indistinctly brochidodromous, 13–17 per side, diverging at 45–65° from the midrib, strongly raised adaxially, raised abaxially, higher-order veins strongly raised to form a conspicuous reticulum adaxially, slightly raised abaxially; petiole 3–5 mm long, somewhat flattened, semi-terete to shallowly canaliculate, sparsely pubescent. ***Inflorescences*** axillary, 1–2-flowered, sparsely pubescent; pedicels not pedunculate, 2.5–5 mm long, 0.7–1.1 mm thick; bracts 2–3, evenly spaced on pedicel, caducous or uppermost sometimes persistent, 1.4–2 mm long, ovate, sometimes bilobed, apex obtuse to rounded; buds broadly ovoid, apex obtuse to short-acuminate. ***Sepals*** spreading at anthesis, connate at base, 1.8–2.7 mm long, 2.7–3.3 mm wide, thickly coriaceous, almost fleshy, broadly ovate to semicircular, apex acute, obtuse, or rounded, sparsely pubescent abaxially. ***Petals*** yellowish white or red *in vivo*; outer petals slightly spreading at anthesis, 5.5–7.3 mm long, 3.5–4.3 mm wide at base, 2.5–2.8 mm wide at midpoint, fleshy, ovate, apex acuminate, densely pubescent on upper half, glabrous in lower half adaxially, sericeous abaxially; inner petals slightly spreading at anthesis, 4.7–6.0 mm long, 3.0–4.0 mm wide at base, 1.9–2.5 mm wide at midpoint, fleshy but thinner than outer petals, rhombic to sagittate, apex acute, base with margin differentiated into two circular corrugated glands 1–1.5 mm in diameter just above and to either side of the claw, the glands and claw glabrous, petal otherwise densely pubescent. ***Stamens*** ca. 100; fertile stamens 1.1–1.4 mm long, narrowly oblong-clavate, apex of connective 0.2–0.3 mm long, depressed-globose, overhanging the anther thecae, minutely papillate, anthers 9–11-locellate, filament 0.2–0.3 mm long; outer staminodes 1.2–1.4 mm long, obovate, narrowly oblong, or clavate, apex obtuse, rounded, or obliquely truncate; inner staminodes 1.1–1.3 mm long, oblong, apex obtuse; staminal cone 1.7–1.9 mm in diameter, 0.5–0.8 mm high, concealing only the bases of the ovaries, rim laciniate. ***Carpels*** 10–17; ovaries 1.2–1.5 mm long, linear-oblong, pubescent, stigmas loosely connivent, 0.9–1.5 mm long, trowel-shaped, glabrous or with a few orange hairs at apex. ***Torus*** flat, 1.7–2.1 mm in diameter. ***Fruit*** of up to 22 glabrate monocarps borne on a pedicel ca. 6 mm long, 2.0–2.8 mm thick, glabrate; torus 6–6.7 mm in diameter, ca. 4.5 mm high, depressed-globose. ***Monocarps*** with green exterior *in vivo*, endocarp color unknown, 3.7–4.8 cm long, 0.7–0.8 cm wide, 0.6–0.7 cm thick, narrowly oblong and slightly falciform, torulose, apex rounded or with a oblique blunt beak 1–2 mm long, base gradually contracted into a stipe 7–11 mm long, 1.8–3.2 mm thick, longitudinally wrinkled and striate; pericarp 0.2–0.4 mm thick. ***Seeds*** 3–5 per monocarp, in a single row, oblique to long axis, 7–7.6 mm long, 4.3–4.8 mm wide, 3.8–4.1 mm thick, ellipsoid, elliptic in cross-section, obliquely truncate at the micropylar end, rounded and sometimes flattened from the adjacent seed at the chalazal end, light brown, smooth, not shiny, raphe/antiraphe not evident, micropylar scar 1.7–1.8 mm long, 0.8–1.0 mm wide, oblong or elliptic; sarcotesta red *in vivo*; aril absent.

**Figure 23.**
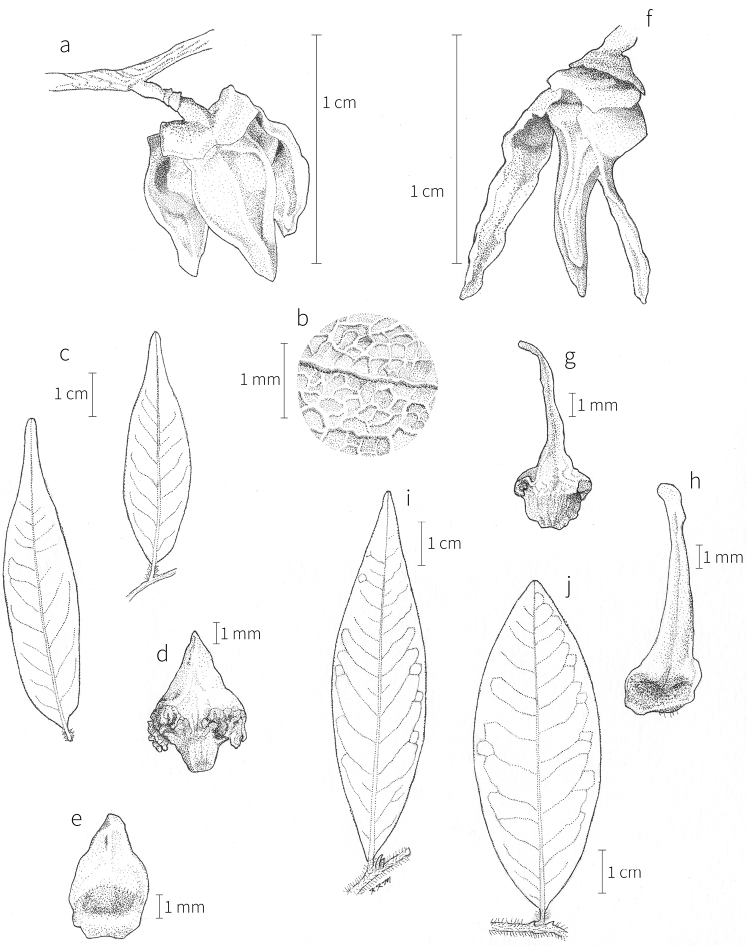
*Xylopiaflamignii* and *X.gilbertii*. **A–E***X.flamignii***A** Flower, side view **B** Close-up of adaxial leaf surface, showing raised venation **C** Leaves **D** Inner petal, adaxial view **E** Outer petal, adaxial view **F–J***X.gilbertii***F** Flower, side view **G** Inner petal, adaxial view **H** Outer petal, adaxial view **I, J** Leaves. **A–E** from *Bradley 1136* (OWU) **F–I** from *Louis 6777* (MO) **J** from *Letouzey 10707* (P).

#### Phenology.

Specimens with flowers have been collected in May, June, October, and December, and with fruits in June (immature) and July.

#### Distribution

**Figure 24.**
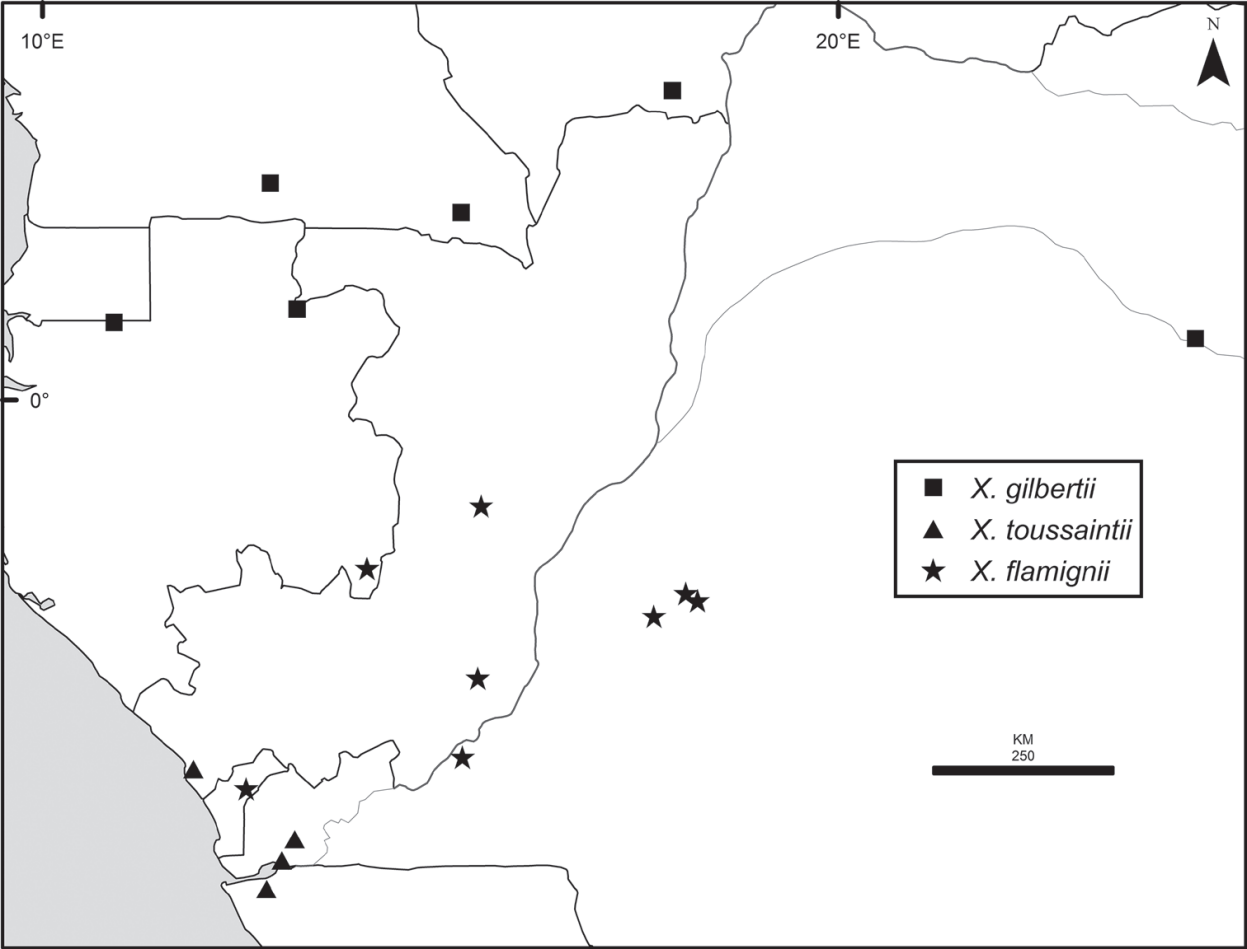
Distributions of *Xylopiagilbertii*, *X.toussaintii*, and *X.flamignii*. Bolder lines represent country borders, fainter lines lakes and major rivers.

(Fig. 24). Occurs from eastern Gabon to the western Democratic Republic of the Congo and south to the Cabinda Province of Angola. One collection is from upland forest, one from forest edge, one from mature riverine forest/savanna edge, and one from secondary forest; the only reported elevation is 430 m.

#### Local names.

Itomba (Loutumbe, *Gilbert 14335*), itumba (*Cauwe/Service Forestier S.F./66*), lucanga lenombe (*Monteiro & Murta 227*).

#### Additional specimens examined.

**GABON**. Haut-Ogooué: ca. 1 km S of Gorilla Research Station, across Mpassa River on savanna-primary riverine forest border, 02°07'15"S, 014°04'40"E, 430 m, 1 Dec 2001 (fl), *Bradley et al. 1136* (MO, OWU). **REPUBLIC OF THE CONGO**. Village Abala, terre Okouésé, Sous-Préfecture de Boundji, 20 Jun 1965 (st), *Bouquet 1447* (P); Plateau Batéké, piste km 46 Maluku—Tréchot, forêt de Mandiélé, 12 Oct 1968 (fl), *Sita 2737* (P). **DEMOCRATIC REPUBLIC OF THE CONGO**. Kinshasa: Kimuenza, 23 Jun 1959 (st), *Pauwels 3391* (BR).—Maï-Ndombe: Ipeke, Lac Mai-Ndombe [“Lac Léopold II”], Jun 1950 (st), *Cauwe/Service Forestier S.F./66* (BR); Lac Mai-Ndombe [“Lac Leo II”], Panza, 3 Jul 1953 (fr, old fl), *Gilbert 14335* (BR). **ANGOLA.** Cabinda, na Reserva da Chiaca, 6 Jun 1960 (st), *Monteiro & Murta 181* (PRE).

This species stands out from other African xylopias by the combination of narrow leaves with a pronounced vein reticulum on the adaxial surface, short broad flowers on short pedicels, and the basal margins of the inner petals marked by corrugated circular glands. In the latter character, it resembles *X.gilbertii* and *X.toussaintii*, but, unlike those species, the leaves are shiny and glabrous on the adaxial surface rather than dull and pubescent. The plant has a greater resemblance to *X.wilwerthii*, but differs from that species in the short pedicels, the short and broad petals, and leaves that are attenuate to short-acuminate; *X.wilwerthii*, in contrast, has pedicels 8.5–21.5 mm long, petals 14–31 mm long, and long-acuminate to caudate leaves.

Only two specimen labels gave notes regarding flower color: one described the petals as yellowish white, the other as red. The label of *Bradley et al. 1163* notes a mild cinnamon odor to the flowers.

With an EOO of 108,538 km^2^ and an AOO of only 32 km^2^, *Xylopiaflamignii* belongs to the group of species that occupy a relatively wide range but are either undercollected or rare (Table 1). It was given a conservation assessment of Vulnerable (D2) by Onana and Cheek (2011).

The isotype of *Xylopialenombe* at BM was received on loan already annotated as *Xylopiaflamignii*, and corresponds well with the type specimen of the latter in its flowers and leaves. This specimen, bearing both flowers and fruits, helped to confirm the identity of specimens of *X.flamignii* with fruits only.

### 15. Xylopia gilbertii

Taxon classificationPlantaeMagnolialesAnnonaceae

Boutique, Bull. Jard. Bot. État 21: 110–111. 1951.

8CADC97F-F388-5241-B841-48364D955E7A

Fig. 23F–J

Xylopia ardua Sillans, Rev. Bot. Appliq. 33: 555–556 + pl. 8. 1953. Type. CENTRAL AFRICAN REPUBLIC [“Oubangi-Chari”]. Boukoko, Dec 1951, *C. Tisserant 2329* (lectotype, here designated: P! [00169140]; isotypes: BM! [000510989], P! [00169141, 00169142, 00169143], US! [2679729]).

#### Type.

DEMOCRATIC REPUBLIC OF THE CONGO [“Belgian Congo”]. Tshopo Province, Yangambi, plateau de la Luweo, alt. 470 m, Nov 1937, *J. Louis 6777* (holotype: BR!; isotypes: B! [100249561], BM! [000510912, 000511052], BR! [8824738], K! [000542217], MO! [1639391], P! [00169152], US! [2091368]).

#### Description.

***Tree*** up to 40 m tall, d.b.h. up to 40 cm, bole slender, cylindrical, with small buttresses at the base; bark brown or mottled with white, yellow, green, or orange-brown, lightly cracked or longitudinally fissured. ***Twigs*** brown, eventually gray and longitudinally wrinkled, persistently erect-pubescent, the hairs orange to rusty and 0.3–0.8 mm long; nodes occasionally with two axillary branches. ***Leaf*** with larger blades 4.2–9.7 cm long, 2.0–3.3 cm wide, chartaceous to subcoriaceous, slightly discolorous, elliptic-oblong to lanceolate, occasionally oblong or ovate, apex acute to nearly rounded, base broadly cuneate to rounded, erect-pubescent (densely so on the midrib) to glabrate adaxially, persistently erect-pubescent abaxially; midrib plane adaxially, raised abaxially, secondary veins indistinctly brochidodromous, 7–14 per side, diverging at 45–60° from the midrib, plane to slightly raised adaxially, raised abaxially, higher-order veins indistinct adaxially, slightly raised abaxially; petiole 2.8–4 mm long, semi-terete to shallowly canaliculate, densely pubescent. ***Inflorescences*** axillary but often clustered at bases of newly elongating shoots, 1–6-flowered, densely pubescent; peduncle 1 per axil, 1.5-2.5 mm long; pedicels 2–6 per peduncle, 1–2 mm long, 1.3–1.7 mm thick; bracts 2, evenly spaced and somewhat overlapping, caducous or persistent, 2.3–3.5 mm long, ovate to broadly ovate, apex obtuse; buds ovoid, apex obtuse or acuminate to an expanded base. ***Sepals*** spreading at anthesis, free or connate just at base, sometimes slightly imbricate at base, 1.6–2.4 mm long, 2.8–4.2 mm wide, coriaceous, reniform to semicircular, apex acute to rounded, densely pubescent abaxially. ***Petals*** fawn-olive, brown-violet, or purple *in vivo*; outer petals slightly spreading at anthesis, 6.6–11 mm long, 2.8–6.4 mm wide at base, 2.4–4.4 mm wide at midpoint, fleshy, lanceolate to ovate, apex obtuse, pubescent on distal half and verrucose on proximal half adaxially, densely appressed-pubescent abaxially; inner petals slightly spreading at anthesis, 5.8–9.3 mm long, 3–4 mm wide, 1.4–3.2 mm wide at midpoint, fleshy, lanceolate, apex obtuse to acuminate, base with margin differentiated into two roughly circular glands 0.6–1.4 mm in diameter, pubescent on distal half and glabrous on concavity/claw adaxially, pubescent abaxially. ***Stamens*** 80–130; fertile stamens 1.1–1.3 mm long, oblong-clavate, apex of connective 0.3–0.4 mm long, shieldlike to capitate, overhanging the anther thecae, minutely puberulent to glabrous, anthers 10–13-locellate, filament 0.3–0.4 mm long; outer staminodes 1.1–1.3 mm long, broadly clavate, apex obtuse to obliquely truncate; inner staminodes 0.9–1.0 mm long, narrowly oblong to broadly clavate, apex obtuse to truncate; staminal cone 1.3–1.8 mm in diameter, 0.4–0.6 mm high, concealing only the bases of the ovaries, rim laciniate. ***Carpels*** 6–11; ovaries 1–1.5 mm long, oblong, pubescent, stigmas connivent, 1.5–2.2 mm long, linear, sparsely pubescent. ***Torus*** flat, 1.6–2.2 mm in diameter. ***Fruit*** of up to 10 sparsely pubescent monocarps borne on a pedicel 4.5–10 mm long, 1.5–2.5 mm thick, sparsely pubescent; torus 2.8–3.8 mm in diameter, 2.5–2.6 mm high, discoid. ***Monocarps*** reddish or purplish green *in vivo*, endocarp color unknown, 2.5–3.7 cm long, 1–1.3 cm wide, 0.5–0.8 cm thick, oblong to clavate, sometimes irregularly torulose, apex obtuse to obliquely truncate, sometimes with an offset blunt beak 1–1.5 mm long, base contracted into a stipe 5–13 mm long, 1.2–3.5 mm thick, sometimes with a longitudinal groove abaxially, obliquely wrinkled, sparsely verrucose; pericarp ca. 1 mm thick. ***Seeds*** up to 6 per monocarp, in a single row, oblique to long axis, 7.4–10 mm long, 4.2–6.7 mm wide, (3.1–) 3.5–5.4 mm thick, ellipsoid, elliptic in cross-section, truncate at micropylar end, rounded at chalazal end, light brown, smooth, dull or slightly shiny, raphe/antiraphe not evident, micropylar scar 1.3–2.2 mm long, 1.5–2.5 mm wide, elliptic, circular, or transversely elliptic; sarcotesta red *in vivo*; aril absent.

#### Phenology.

Specimens with flowers have been collected from October to February, and with fruits from February to April, in June, and from October to December.

#### Distribution

(Fig. 24). Scattered in a narrow band from southern Cameroon and northern Gabon east to the southwestern Central African Republic and the northern Democratic Republic of the Congo; upland forest; 470–900 m. Associates include *Coulaedulis* Baill. and *Millettiaduchesnei* De Wild. Harris (2002) reports the species from the Dzanga-Sangha region of SW Central African Republic, but we have not seen the vouchers.

#### Local names.

Bompaie bo fufow (Turumbu, *Louis 6777*), molo-mosoma (Lissongo, *Tisserant 625, 2329*).

#### Additional specimens examined.

**CAMEROON**. A 9 km à l’ouest de Yenga Port Gentil, village situé à 35 km au NNE de Moloundou, 21 Apr 1971 (fr), *Letouzey 10707* (P); près Akonetche, PK 95 sur route Minton I (70 km E de Djoum)-Mbalam (140 km ESE de Djoum), 22 Jan 1973 (fl), *Letouzey 11876* (BR, K). **CENTRAL AFRICAN REPUBLIC.** Boukoko, 17 Jan 1948 (fl), *Tisserant 625* (BM, P). **GABON**. Ogooué-Ivindo: Bélinga, Mines de Fer, 700–900 m, 4 Jun 1966 (fr), *Hallé 3735* (P).—Woleu-Ntem: ca. 9 km ESE Medouneu, Elot, bas inselberg Simanguen, 0°58.62'N, 10°54.01'E, 661 m, 21 Dec 2002 (fr), *Ngok Banak et al. 1057* (MO); Inselberg, ca. 28 km ESE of Medouneu, 0°55'N, 11°01'E, 500 m, 3 Feb 1986 (fl, fr), *Reitsma et al. 1821* (MO, NY, WAG). **DEMOCRATIC REPUBLIC OF THE CONGO**. Tshopo: Territ. Tsangi, Yangambi, 12 Feb 1952 (fl), *Donis 3634* (K); Yangambi, 1938 (st), *Gilbert 1099* (K, US); Yangambi, 1938 (buds) *Gilbert 1423* (K, MO, US); Yangambi, 29 Nov 1935 (fl), *Louis 733* (NY, RSA); Yangambi, 470 m, 17 Oct 1936 (yg fr), *Louis 2741* (K); Yangambi, réserve-flore Isalowe, 7 Oct 1937 (st), *Louis 6324* (FI-T, MO, P); Prov. Orientale, Territ. Tsangi, Yangambi, plateau de l’Isalowe, 12 Mar 1951 (fr), *Toussaint 886* (K); Prov. Orientale, Tsangi Territory, Yangambi, environs de la Réserve floristique, Nov 1960 (fr), *Yafunga 7* (WAG).

*Xylopiagilbertii* is either an uncommon or infrequently collected species. It can be recognized by a combination of the sparse but persistent erect pubescence of the twigs, leaves, and monocarps, the relatively broad but small flowers on short pedicels, and the rounded marginal glands on the inner petals. *Xylopiagilbertii* shares inner petal glands with *X.flamignii* and *X.toussaintii*, but the former has strongly reticulate leaves and more numerous, up to 22, glabrate monocarps, while the latter has proportionately narrower inner petals and less persistent indument of the twigs, leaves, and monocarps. An odd feature of *X.gilbertii*, not observed in the other two species or in fact in any African *Xylopia*, is the tendency for inflorescences to be produced at the bases of expanding shoots, such that it is difficult to determine the inflorescence position. *Xylopiastaudtii* has occasionally been confused with *X.gilbertii*, but lacks the hairy twigs and leaves and the inner petal glands, and has larger and distinctly arillate seeds.

Descriptions of flower color for *Xylopiagilbertii* are inconsistent. Boutique (1951a), in the protologue for the species, described “Flores lutei,” but on the label of the holotype specimen, the flowers are described as “olive-fauve,” i.e. fawn-olive. Other collectors give flower colors such as brown-violet and purple, suggesting a darker color for the flowers. The lighter colors likely represent immature flowers. Endocarp color is unknown, but Gautier-Hion et al. (1985) reported that the seeds are dispersed by birds, monkeys, and small rodents, noting that the small rodents can also be predators on the seeds.

Sillans (1953), while mentioning *X.gilbertii* in the protologue for *Xylopiaardua*, did not explain how the two species were to be distinguished. The type specimen of *X.ardua*, while showing shorter and broader leaves than those of the type of *X.gilbertii*, overlaps widely with other specimens in both qualitative and quantitative characters, and we have thus placed it in synonomy. Two specimens from northern Gabon, *Reitsma et al. 1821* and *Ngok Banak et al. 1057*, also with broader leaves, both differ somewhat from specimens elsewhere in having subcoriaceous leaves and longer stipes on the monocarps, but otherwise conform to this species.

We calculated an EOO of 249,251 km^2^ and an AOO of 52 km^2^ for *Xylopiagilberti*. It was given a conservation assessment of Vulnerable (D2) by Onana and Cheek (2011).

### 16. Xylopia gracilipes

Taxon classificationPlantaeMagnolialesAnnonaceae

D. M. Johnson & N. A. Murray in Burrows et al., Trees and Shrubs of Mozambique, Taxonomic Notes, 1035–1039, fig. 2, 2018.

E7CC9CF2-AD87-5AF5-A631-CFD34B4C266E

Figs 4F , 25A–L

#### Type.

MOZAMBIQUE. Zambézia Province, Altomolócuè, Gilé, ao km 10, monte Gilé (Ig), 300 m, 21 Dec 1967, *A. R. Torre & Correia 16681* (holotype: WAG).

#### Description.

***Tree*** up to 20 m tall or occasionally a shrub to 5 m, d.b.h. up to 20 cm, clear bole up to 4 m, sometimes ribbed, branches horizontal; bark light gray, sometimes mottled, smooth or flaking. ***Twigs*** reddish brown to brownish black, pubescent, the hairs 0.2–1.2 mm long, becoming gray to brown, glabrate, cross-cracked; nodes occasionally with two axillary branches. ***Leaf*** with larger blades 3.3–9.7 cm long, 1.7–4.1 cm wide, chartaceous to subcoriaceous, slightly discolorous, (narrowly) oblong to elliptic, occasionally lanceolate, elliptic-oblanceolate, or obovate, apex broadly acute, obtuse, or rounded, occasionally emarginate or acuminate with a broad acumen 1.5–7 mm long, base cuneate to obliquely rounded, rarely with some leaves on a specimen truncate, sparsely pubescent or with hairs restricted to the midrib adaxially, appressed-pubescent abaxially; midrib plane to slightly raised adaxially, raised abaxially, secondary veins weakly brochidodromous, 7–14 per side, diverging at 45–75° from the midrib, slightly raised on both surfaces, higher-order veins slightly raised on both surfaces but vein reticulum often more prominent adaxially; petiole 3.5–9.5 mm long, semi-terete or flattened to shallowly canaliculate, densely pubescent. ***Inflorescences*** axillary, 1–4-flowered; pedicels arising separately from axil, rarely 2 from a common peduncle, pubescent; peduncle, if present, 0.7–2.1 mm long; pedicels 2.7–8.5 mm long, 0.3–1.0 mm thick; bracts 2, usually one to either side of the midpoint, caducous to somewhat persistent, 1.3–1.6 mm long, ovate, apex obtuse; buds linear to lanceolate, straight to falciform, apex acute to obtuse. ***Sepals*** erect to slightly spreading at anthesis, 1/4–1/2-connate, 1.1–2.5 mm long, 1.8–3.0 mm wide, coriaceous, triangular to broadly ovate, apex acute, pubescent abaxially. ***Petals*** pale green or yellow *in vivo*; outer petals somewhat spreading at anthesis, 9.5–18.1 mm long, 2.5–3.0 mm wide at base, 0.8–1.8 mm wide at midpoint, coriaceous, linear to linear-lanceolate, attenuate, apex obtuse, channelled adaxially, keeled abaxially, gray-puberulent except for glabrous base adaxially, yellow-brown pubescent abaxially; inner petals curved outward to weakly geniculate at anthesis, 6.1–15.3 mm long, 1.5–2.5 mm wide at base, 0.4–1.1 mm wide at midpoint, coriaceous, linear-subulate, apex acute, base with undifferentiated margin, keeled on both surfaces, densely gray-puberulent on both surfaces except for glabrous base. ***Stamens*** ca. 120; fertile stamens 1.0–1.4 mm long, narrowly oblong, apex of connective 0.1–0.2 mm long, shieldlike, overhanging the anther thecae, glabrous, anthers ca. 9-locellate, filament 0.3–0.4 mm long; outer staminodes 0.9–1.3 mm long, oblong, apex irregularly truncate to rounded; inner staminodes 0.9–1.1 mm long, oblong to clavate, apex rounded; staminal cone 1.1–1.9 mm in diameter, 0.6–1.2 mm high, concealing only the bases of the ovaries, rim even or laciniate. ***Carpels*** 8–9; ovaries 0.8–1.1 mm long, narrowly oblong, ascending-pilose; stigmas more or less connivent, 1.6–3.3 mm long, linear, with a tuft of yellow hairs at the apex. ***Torus*** shallowly concave beneath ovaries, 1.5–2.7 mm in diameter. ***Fruit*** of up to 10 sparsely pubescent to glabrate monocarps borne on a pedicel 4.9–8.5 mm long, 2.2–4.8 mm thick, glabrate; torus 3.4–7.5 mm in diameter, 3.8–6.8 mm high, ovoid. ***Monocarps*** green with light green endocarp *in vivo*, 2.8–5.0 cm long, 0.8–1.1 cm wide, 0.8–0.9 cm thick, oblong, often irregularly torulose, apex obtuse with a broad blunt beak 2–3 mm long, base contracted into a stipe 4–11 mm long, 2–4.5 mm thick, obliquely wrinkled, verrucose, occasionally a little glaucous; pericarp ca. 0.4 mm thick. ***Seeds*** up to 5 per monocarp, in a single row, lying oblique to long axis, 9–12.2 mm long, 6.5–7.7 mm wide, 5.1–6.1 mm thick, ellipsoid, elliptic to semicircular in cross-section, narrowed and truncate at micropylar end, rounded at chalazal end, brown, smooth, dull, raphe/antiraphe not evident, micropylar scar 1.5–2.7 mm long, 1.0–2.3 mm wide, oblong; sarcotesta orange to red *in vivo*; aril absent.

**Figure 25.**
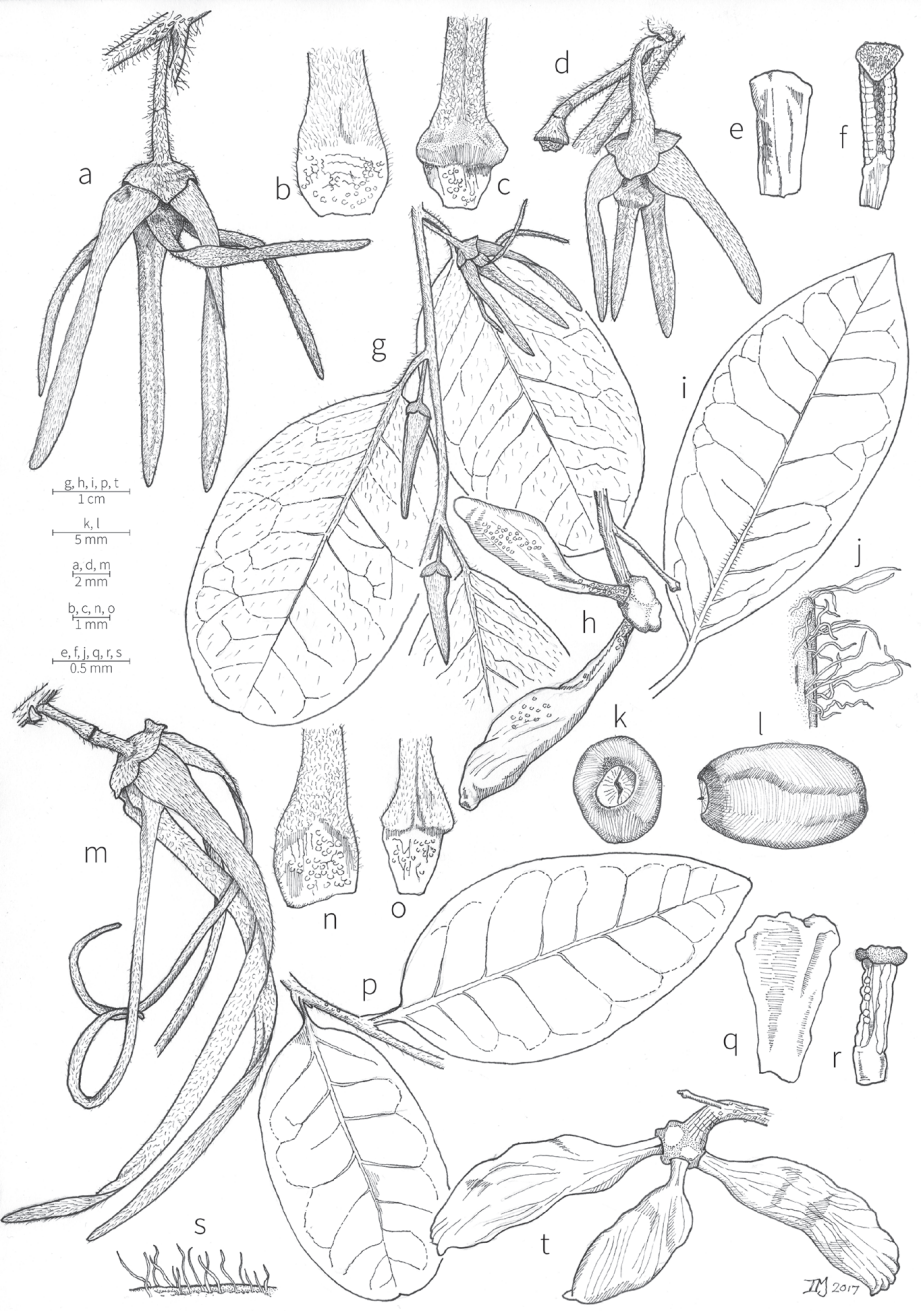
*Xylopiagracilipes* and *X.torrei*. **A–L***X.gracilipes***A** Flower, side view **B** Base of outer petal, adaxial view **C** Base of inner petal, adaxial view **D** Flower, side view **E** Outer staminode, abaxial view **F** Fertile stamen, abaxial view **G** Habit **H** Fruit, side view **I** Leaf from same specimen as G **J** Close-up of twig pubescence **K** Seed, view of micropylar end **L** Seed, side view **M–T***X.torrei***M** Flower, side view **N** Base of outer petal, adaxial view **O** Base of inner petal, adaxial view **P** Habit **Q** Outer staminode, abaxial view **R** Fertile stamen, abaxial view **S** Close-up of twig pubescence **T** Fruit, side view. **A–C, E–J** from *Torre & Correia 16681* (WAG) **D** from *Renny 253* (PRE) **K, L** from *Fidalgo de Carvalho 685* (MO) **M–T** from *Goyder 5037* (**K**). Figure originally published in Burrows et al. (2018), reproduced with permission.

#### Phenology.

Specimens with flowers have been collected in August and from October to February, and with fruits from January to March, and in May, October, November, and December. The specimens with flowers or fruits from outside of the October to February period are all from South Africa.

#### Distribution

**Figure 26.**
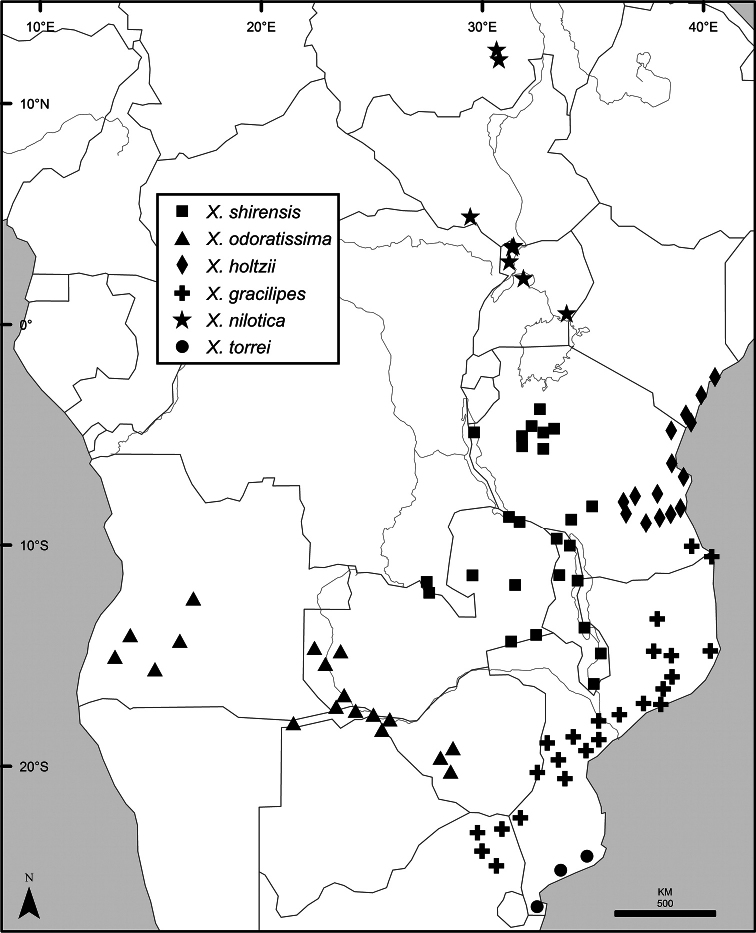
Distributions of *Xylopiashirensis*, *X.odoratissima*, *X.holtzii*, *X.gracilipes*, *X.nilotica*, and *X.torrei*. Bolder lines represent country borders, fainter lines lakes and major rivers.

(Fig. 26). Occurs from Tanzania south of the Rufiji River through Mozambique, where it is widespread, and then southwestward to eastern Zimbabwe and northeastern South Africa, in open miombo or other deciduous woodland, on rocky, sandy, or clay soils, at elevations of 40–1000 m. Associates include *Albiziaadianthifolia*, *Bosqueiaphoberos*, *Cathaedulis*, *Khayanyasica*, *Lecomtodoxahenriquezii*, and *Monanthotaxischasei*, as well as species of *Afzelia*, *Baphia*, *Bauhinia*, *Brachystegia*, *Dalbergia*, *Diospyros*, *Kigelia*, *Sclerocarya*, and *Sterculia*. The species also occurs in an evergreen forest area of particularly high rainfall in eastern Zimbabwe, where the forest is described by Farrell (1960) as having a 100 ft (ca. 30 m) canopy layer, a 50 ft (ca.15 m) understory layer, and a shrub layer.

#### Local names.

Garangerere (*Simão 946*), lòmué (Murroutho tôco-tôco, *Andrada 1898*), mugaranjerere (Chindao, *Gomes Pedro 4224*, *4394*), mukáramo (*D’Hondt 686*), mulalabungo (*Fidalgo de Carvalho 685*), murikiriki (*D’Hondt 686*), murrouthonambuadji (*Barbosa & Carvalho 2911*), nionjono (*D’Hondt 686*), sangué (*Torre & Correia 14168*), sânguè (Namae, *Andrada 1529*,) tongolo (Tongo, *Lamont 27984*).

#### Additional specimens examined.

**TANZANIA.** Lindi: Lindi-Bezirk, Lutambasee, 240 m, 1 Nov 1934 (fl), *Schlieben 5571* (BM, K, MO, P, PRE, US).—Mtwara: Mtwara District, 4.5 km S of Siwani Village on road from Mtwara to Maharunga and Rovuma R. mouth, 6 Nov 1978 (fl, fr), *Magogo & Innes RRI 471* (EA, K, NHT). **ZIMBABWE.** Umtali District, Burma farm, Burma Valley, 30 Dec 1959 (fr), *Chase 7238* (BM—2 sheets, MO); Chipinge [“Chipinga”] District, Upungura [Upungura Forest, in a Kloof at 2500–3000 ft. on the escarpment above Dumisayi], Feb 1960 (fr), *Farrell 202* (MO); Chipinga District, Craigmore Farm, on the Nyagadza River W of Chirinda, 2800 ft, Oct 1968 (fl, fr), *Goldsmith 159/68* (B, EA, MO, WAG—2 sheets); Melsetter District, forest on banks of lower Timbiri River, 14 Feb 1958 (st), *Hall 469* (BM); Chipinga District, 2032 D1, ca. 4 km NE Musirizwe/Bwazi River confluence, ca. 540 m, 28 Jan 1975 (st), *Pope et al. 1389* (MO, PRE); Melsetter District, Haroni River, 4 Dec 1965 (fl, fr), *Wild et al. 6651* (BM). **MOZAMBIQUE.** Cabo Delgado or Niassa: Distrito de Metoro, Namacuto, 30 Jan 1984 (fl), *Groenendijk et al. 856* (DSM, LMA—photo, MO, WAG).—Manica: Revue, 1 Oct 1946 (yg fr), *Simão 946* (LMA—photo); Spungabera, próx. da Missão Católica, 14 Nov 1943 (fl), *Torre 6192* (EA, LMA—photo, WAG); Dombe, entre Javera e Machire, a 6 km de Javera, 24 Oct 1953 (bud, fr), *Gomes Pedro 4394* (BR); Spungabera, próx. da Missão Católica, 14 Nov 1943 (fl), *Torre 6192* (EA, WAG); Moribane, entre Moribane e Sanguene, a 5 km de Moribane, 5 Oct 1953 (buds), *Gomes Pedro 4224* (PRE); Dombe, picada Serração Braunstaine-Machango (Machonga), a ca. 7 km de Serração, 26 Feb 1965 (fl), *Pereira & Marques 909* (LMA—photo).—Nampula: Prox. Serra Chinga, Estrada de Ribáuè, a ca. 28 km deste Poroação, 22 Oct 1968 (fl), *Aguiar Macedo 3716* (LMA—photo); Ribáuè-Namina, perto de Ribáuè, 21 Oct 1982 (buds), *D’Hondt 686* (LMA—photo); Monapo, andados 7 km de Itoculo para Nacala, (Ke), 4 Dec 1963 (fl), *Torre & Paiva 9413* (EA, K).—Sofala: Gorongoza, Estrada de Vila Paiva de Andrada, a mais ou menos 5.5 km a cima cruz, Pávua, 22 Nov 1965 (fr), *Aguiar Macedo & Balsinhas 1510* (LMA—photo); Búzi, Reserva Florestal do Mucheve (Talhões de ensaio), 28 Oct 1963 (fl, fr), *Fidalgo de Carvalho 685* (MO); 40 km N of Dondo towards Muanza, Beira region, 10 May 1998 (fr), *Lötter 261* (LYD—photo); 50 km N of Dondo, Beira region, 11 May 1998 (fr), *Lötter 269* (LYD—photo); between Muanza and Chinizua, 13 May 1998 (fr), *Lötter 284* (LYD—photo); Chissadze (Cheringoma), 29 Jun 1947, *Simão 1326* (LMA—photo); Beira District, Gorongosa National Park, western park limits, Missicadzi track, Feb 1972 (st), *Tinley 2382* (MO).—Zambézia: Namacurra, Estrada de Quelimane, perto de Naciaia, 28 May 1949, *Andrada 1529* (LMA—photo); A. Molócuè, junto da Estrada para o Niponde, 24 Jul 1949, *Andrada 1898* (LMA—photo); entre Mocuba e Quelimane a 95.2 km de Mocuba, 28 May 1949 (fr), *Barbosa & Carvalho 2911* (LMA—photo); Bajone, entre Namuera & Murroa, 2 Oct 1949 (fr), *Barbosa & Carvalho 4284* (LMA—photo); entrance road to Casa Branca, ca. 82 km from Nicoadala on Caia road, 29 Dec 2006 (fl), *Burrows & Burrows 9848* (BPNR—image); Nante, distrito Maganja da Costa, 18 Jul 1978, *Macuácua 578* (LMA—photo); Maganja da Costa, ao km 23, estrada para Namacurra (Hh), 40 m, 27 Jan 1966 (fl), *Torre & Correia 14168* (PRE). **SOUTH AFRICA.** Limpopo: [“Transvaal”], Zoutpansberg District, Eubabeni (klein Australië), 800 m, 11 Aug 1980 (fl), *von Breitenbach 16459* (PRE); Zoutpansberg District, Funduzi, 23 Jan 1931 (fl), *Bremekamp & Schweickerdt 348* (PRE); Transvaal, Zoutpansberg District, Makonde Mission Station, 15 mi NE of Sibasa, 2500 ft, 18 Feb 1952 (st), *Codd 6806* (K); Sikorora [near Leydsdorp] nr. Macoutsie Riv., Dec 1922 (fl), *van Dam 22934* (PRE); Pietersburg Tul., Letaba, New Agatha, *Forestry Department s. n.* (K—2 sheets); Venda, Messina, Mabila village, SE of Nwanedi Game Park, 15 Jan 1989 (fr), *Hardy 6914* (PRE); 2230 BD (Messina), Phiphidi, 15 Nov 1978 (fl), *Hemm 881* (MO); Woodbush, N. Transvaal, Dec 1928 (fl, fr), *Hutchinson 2240* (K); Shilowane, Oct–Nov (fl), *Junod 1427* (K); road between Mufulwi and Makuleni, 997 m, 22°42'06"S, 30°28'32"E, 17 Jan 2004 (fr), *Klein 767* (K); Regio Zoutpansberg, Punda Maria, K. N. P., Dec 1945 (buds), *Lamont 27984* (PRE); Zoutpansberg District, Elim, Dec 1930 (st), *Obermeyer 839* (PRE); Distr. Letaba, Cyprus farm, slopes of Kopje, 23 Nov 1968 (fl), *Renny 253* (PRE); E. Transvaal, Dist. Punda Maria, 15 Oct 1952 (buds, fr), *v. d. Schijff 969* (K); Kruger National Park, Shipudza, NW of Punda Maria, 1700 feet, 24 May 1954, *v. d. Schijff 3779* (EA, K, L, MO); Dist. Krugerpark, Wambia, 2 Jun 1961 (st), *v. d. Schijff 5686* (K); New Agatha - Trl., Letaba District, Feb 1933 (fl), *Schnetler 8177* (PRE); Lekgalameetse Nat. Res., Balloon, near entrance gate of reserve, 26 Nov 1985 (fl), *Stalmans 285* (K—2 sheets); Nor Transvaal, Zoutpansberg, Njelele - Tol, 10 Feb 1951 (fl, fr), *Stopp M79* (M); Farm Balloon 71 KT, along Makhuiswi road, 860 m, 2 Aug 1983 (fr), *Venter 9808* (LYD—photo); Dist. Sibasa, Ishakhuma, 23°03'S, 30°18'E, 2100 ft, 9 May 1951 (fr), *van Warmelo 5159/14* (K); Dist. Sibasa, Rambuda, Loen., 22°47'S, 30°21'E, 2400 ft, 19 Dec 1951 (fl), *van Warmelo 51219/7* (K); northern Transvaal, Sibasa District - Venda, 22°30° DA, next to tar road between Thengwe and Sagole Spa near top of mountain pass, 923 m, 31 Mar 1994 (fr), *van Wyk BSA 2023* (PRE).

*Xylopiagracilipes* can be separated from its congeners by elliptic to oblong leaf blades that are cuneate to broadly cuneate at the base, axillary inflorescences of 1–2 pedicels 0.3–1.0 mm thick arising separately from the axil, outer petals no more than c. 18 mm in length and obtuse at the apex. In addition, the petals are uniformly pale green to yellow and lack purple coloration on the inner base.

Robson (1960) cited two of the Mozambique collections (*Barbosa & Carvalho 4284*, *Torre 6192*) as *X.holtzii*. He used the specimen *Hutchinson 2240* from South Africa as the basis for the illustration of *Xylopiaholtzii* in the same work (tab. 14, figs A1–A7). Similarly, Verdcourt (1971b) identified the specimen *Schlieben 5571* from the Lindi Region of southern Tanzania as “*X.parviflora*,” i.e. *Xylopiaholtzii* s. s. (see Johnson et al. 2017 for explanation). A study of a wider range of material than was available to these authors, supplemented with field knowledge of the plants in Mozambique shared by Mervyn Lötter, has shown that this species differs from *Xylopiaholtzii* in a number of characters. The leaf blades, while somewhat variable in size and shape, are on the average smaller (3.3–9.7 cm long) and tend to be elliptic to oblong. The pedicels arise side by side in the inflorescences, and are more slender. The monocarps by maturity are glabrate. In contrast, in *X.holtzii* the leaf blades are slightly larger (4.8–11.4 cm long) and lanceolate or rarely narrowly oblong, most inflorescences have pedicels branching from a common peduncle, and the mature monocarps are persistently pubescent. Furthermore, the outer petals of *X.holtzii*, while green to yellow in color like those of *X.gracilipes*, are marked with a purple blotch on the inner base, are acute at the apex, and reach a length of 25 mm.

*Xylopiagracilipes* also resembles *Xylopiashirensis*, with which it is not known to overlap in range, but it has narrower leaf blades cuneate to broadly cuneate at the base and more slender (0.3–1.0 mm thick) pedicels; in contrast, the leaf blades in *X.shirensis* are proportionally broader and usually rounded to truncate at the base, and the pedicels, while of the same length as those of *X.gracilipes*, are instead 1.2–1.5 mm thick.

Some variants need further field study. The hairs on the specimen *Groenendijk et al. 856* are longer and more abundant than is otherwise typical for the species, and the leaves of specimens from the Venda area of South Africa (e.g., *van Wyk BSA 2023*) are much smaller and more rounded than those from elsewhere in the distribution.

### 17. Xylopia holtzii

Taxon classificationPlantaeMagnolialesAnnonaceae

Engler, Bot. Jahrb. Syst. 34: 159. 1904.

A0DCE580-A033-587D-8A49-3FDC832CB31C

Figs 27G–K , 28A

#### Type.

TANZANIA. Dar-es-Salaam Region, Dar-es-Salam, Puguberge, Dichter Busch, 29 Feb 1903, *W. Holtz s. n.* (lectotype, here designated: B! [100153143]).

***Tree*** up to 20 m tall, d.b.h. up to 30 cm, clear bole to 8 m on 12 m tree, trunk fluted or with small narrow buttresses at base; bark light gray to gray-brown, sometimes blotched with white, smooth or transversely ringed by fine lenticels. ***Twigs*** light brown, orange-brown, gray-brown, brownish gray, or blackish brown, initially pubescent, the hairs 0.1–0.8 mm long, becoming glabrate; nodes occasionally with two axillary branches. ***Leaf*** with larger blades 4.8–11.4 cm long, 1.7–4.4 cm wide, chartaceous to subcoriaceous, concolorous to slightly discolorous, lanceolate to narrowly oblong, elliptic, or oblanceolate, apex obtuse, often attenuate, rarely emarginate, base broadly cuneate to rounded, glabrous to sparsely pubescent adaxially, sparsely appressed-pubescent abaxially; midrib slightly raised to plane adaxially, raised abaxially, secondary veins weakly brochidodromous, 8–17 per side, diverging at 40–70° from the midrib, slightly raised on both surfaces, higher-order veins indistinct adaxially, indistinct to slightly raised abaxially; petiole 3–12 mm long, shallowly canaliculate to flattened, pubescent or rarely glabrous. ***Inflorescences*** axillary, 1–6-flowered, pubescent; at least some inflorescences with peduncles, 1-2 peduncles per axil, 2–4.5 mm long; pedicels 2–4 per peduncle, or arising directly from axil, 2.0–7.5 mm long, 0.7–0.8 mm thick; bracts 2–3, evenly spaced along length of pedicel, caducous or rarely persistent, 0.9–1.3 mm long, ovate to semicircular, apex acute to rounded; buds linear, slightly falciform, apex acute. ***Sepals*** erect to slightly spreading at anthesis, 2/5–1/2-connate, 1.6–2.4 mm long, 1.9–2.8 mm wide, coriaceous, orbicular, apex acute to short-acuminate, appressed-pubescent abaxially. ***Petals*** yellow-green, yellow, or cream-colored with pinkish purple at the base adaxially *in vivo*; outer petals curved outward but with the apices weakly incurved at anthesis, 10.5–25 mm long, 2.1–3.7 mm wide at base, (0.7–) 0.9–1.9 mm wide at midpoint, coriaceous, linear, apex acute to obtuse, weakly keeled on abaxial surface, gray-puberulent adaxially, golden appressed-pubescent abaxially; inner petals curved outward but with the apices strongly incurved at anthesis, 7.7–21.4 mm long, 1.6–2.7 mm wide at base, 0.5–1.2 mm wide at midpoint, coriaceous, linear-subulate, apex acute, base with undifferentiated margin, ridged on both surfaces, densely puberulent on both surfaces. ***Stamens*** 60–70; fertile stamens 1.0–1.5 mm long, narrowly oblong, apex of connective red to reddish purple *in vivo*, 0.1–0.3 mm long, shieldlike, overhanging anther thecae, glabrous, anthers 10–13-locellate, filament 0.3–0.5 mm long; outer staminodes 0.9–1.4 mm long, quadrate to broadly clavate, apex truncate; inner staminodes 1.0–1.2 mm long, clavate, apex truncate; staminal cone 0.7–1.9 mm in diameter, 0.7–1.1 mm high, concealing only the bases of the ovaries, rim laciniate. ***Carpels*** 5–12; ovaries 0.9–1.1 mm long, narrowly oblong, pubescent; stigmas loosely connivent, 0.9–1.8 mm long, linear, apices pubescent. ***Torus*** flat, 1.6–2.4 mm in diameter. ***Fruit*** of up to 8 pubescent monocarps borne on a pedicel 4.5–8 mm long, 1.6–3.0 mm thick, pubescent; torus 3.0–6.7 mm in diameter, 2.6–5.4 mm high, irregularly globose. ***Monocarps*** with green exterior and white (immature?) endocarp *in vivo*, up to 8 per fruit, 2.3–4.0 cm long, 0.8–1.3 cm wide, 0.7–0.8 cm thick, oblong, irregularly torulose, apex obtuse to rounded, often with a wide beak 1.5–2.2 mm long, base contracted into a stipe 6–10 mm long, 3–3.5 mm thick, finely verrucose, pubescence most persistent on stipe; pericarp 0.4–1.0 mm thick. ***Seeds*** 1–3 per monocarp, in a single row, lying oblique to long axis, 11.8–12.3 mm long, 5.9–7.0 mm wide, 4.9–6.0 mm thick, flattened-ellipsoid, broadly elliptic in cross-section, obliquely truncate at micropylar end, rounded at chalazal end, brown, smooth, shiny, raphe/antiraphe not evident, micropylar scar 1.4–1.9 mm long, 0.9–1.0 mm wide, oblong, elliptic, or ovate-elliptic; sarcotesta orange *in vivo*; aril absent.

**Figure 27.**
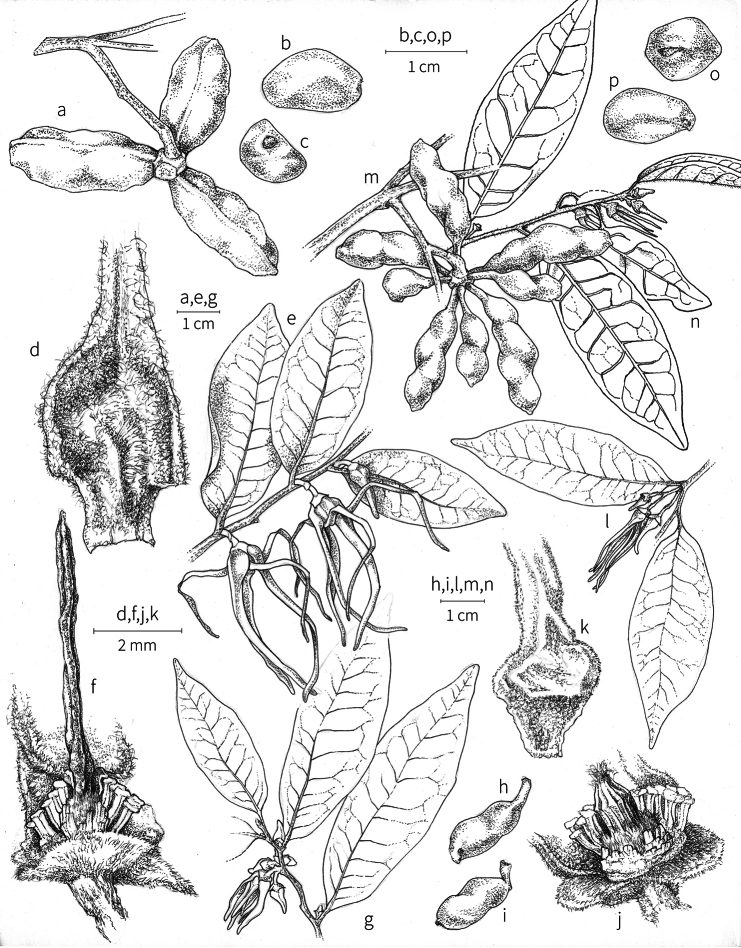
*Xylopialongipetala*, *X.holtzii*, *X.katangensis*, and *X.shirensis*. **A–F***X.longipetala***A** Fruit **B** Seed, side view **C** Seed, view of micropylar end **D** Base of inner petal, adaxial view **E** Habit **F** Gynoecium surrounded by staminal cone with a few attached stamens **G–K***X.holtzii***G** Habit **H, I** Detached monocarps **J** Gynoecium surrounded by staminal cone with a few attached stamens **K** Base of inner petal, inner (adaxial) surface **L***X.katangensis*, habit **M–P***X.shirensis***M** Fruit **N** Habit **O** Seed, view of micropylar end **P** Seed, side view. **A–C** from *Jongkind & Nieuwenhuis 3031* (WAG) **D, F** from *McPherson 17911* (MO) **E** from *Hallé & Le Thomas 190* (**P**), and following photograph of fresh flowers of same collection in Le Thomas (1969)**G, J, K** from *Johnson et al. 1938* (OWU) **H, I** from *Templer H167/53* (K), **L** from *Letouzey 4498* (MO) **M** from *Richards 13266* (K) **N** from *Richards 14438* (K) **O, P** from *Bredo 6276* (BR).

#### Phenology.

Specimens with flowers have been collected in February, March, from May to July, and from October to December, and with fruits from January to March, in May and August, and from October to December.

#### Distribution

(Fig. 26). Occurs near the Indian Ocean coast from southern Kenya to central Tanzania, extending inland in Tanzania to the scarp of the Udzungwa Mountains and the Selous region, growing in dry evergreen or semi-deciduous forest, less commonly in miombo (*Brachystegia*) woodland, at elevations of 0–800 (–1670) m.

#### Local names.

Lulema mbala (*Rodgers 388*); mlawilila (Kidoe, *Abeid 550*), muporota (Kihehe, *Haerdi 214/0*), mporoto (Kimbunga, *Haerdi 214/0*), mporota (Kisagara, *Haerdi 214/0*). An ethnobotanical study of the Digo people on the coast between Tanga and Mombasa reported mnyinyi [shining leaves], mchiza tasaka, and mwahula tsaka [forest breaker, i.e. emergent through the canopy] (Pakia 2005) as additional local names in that area.

#### Additional specimens examined.

**KENYA.** Kwale: Jardini, Jun 1962 (fl), *Birch 62/188* (K); Jilori, *Black M.37* (K); Kwale District, Mwasangombe Forest 15 km SW of Kwale, 230 m, 27 Aug 1953 (fr), *Drummond & Hemsley 4015* (B, EA, FI-T, K); Jardini Hotel, forest ca. 30 km S of Mombasa, 22-23 Jun 1970 (fl), *Faden 70/201* (EA, K, MO); Diani Forest, 1 km N to 1.5 km S of the turnoff for the new road to Jadini Hotel, 4°19'S, 11–13 Jul 1972 (fl), *Gillett & Kibuwa 19846* (B, BR, EA, K, MO, NHT, P, WAG); *Gillett & Kibuwa 19847* (K); Mkongani North FR, 4°17'S, 39°19'E, 280 m, 13 Jul 1987 (bud), *Luke & Robertson 508* (MO); Diani Forest, 4°20'S, 39°34'E, 5 m, 16 Jun 1994 (fl), *Robertson 6957* (K, MO); Mrima Hill, 4°29'S, 39°16'E, 170 m, 4 Feb 1989 (st), *Robertson et al. MDE 50* (K); Gogoni, 30 mi S of Mombasa, 7 Oct 1953 (fl, fr), *Templer H167/53* (K–2 sheets, MO).—Lamu: Witu District, Mambasasa, 7 Nov 1957 (old fl), *Greenway 9471* (FI-T, K, PRE). **TANZANIA.** Iringa: Kilolo District, Kisegese Village, at base of hill, 08°01'58"S, 036°22'46"E, 360 m, 13 Mar 2006 (fl, fr), *Festo et al. 2213* (MO).—Lindi: Selous Game Reserve, T8, ca. 15 km SSW of Kingupira, 8°35'S, 38°31'E, ca. 150 m, 15 Nov 1975 (fl), *Vollesen 2992* (K, WAG).—Morogoro: Hundupila/Kiberege, Ifakara, 6 Mar 1959 (fl), *Haerdi 214/0* (K, WAG); along trail to Mwanihana Peak, SW of Udzungwa Mountains National Park headquarters, 19/20 Feb 1996 (fl, fr), *Johnson & Murray 1889* (OWU spirit collection); Ulanga District, Iragua village, 100 m SE of main road from Iragua to Itete, 8°33'54"S, 36°29'52"E, 360 m, 21 Jan 1999 (fr), *Kayombo 1589* (MO); Sanje Logging Camp, 1670 m, 30 Dec 1980 (fr), *Rodgers 388* (DSM).—Pwani: Bagamoyo District, Zaraninge Forest Reserve, near WWF office, near forest guards’ camp, 6°17'S, 38°34'E, 150 m, 8 Jun 1999 (fl), *Abeid 550* (MO); Pugu-Berge, s. d., *Holtz 3202* (B, PH); Coast Region, Kisarawe District, Pugu Forest Reserve, bus roundabout area ca. 4 km E of Kisarawe, 06°53'30"S, 39°06'00"E, 200 m, 26 May 1996 (fl), *Johnson & Murray 1938* (OWU); Rufiji District, forest near WWF Office, 08°18'57"S, 38°57'39"E, 250 m, 6 Dec 1998 (fl), *Kibure 320* (MO); T6, Kisarawe, Pugu Forest Reserve, June 1954 (fl), *Semsei 1718* (EA, K—2 sheets); Selous Game Reserve, Beho Beho, 07°40'S, 37°55'E, 250 m, 6 Jun 1977 (fl), *Vollesen 4619* (DSM, WAG).—Tanga: Kihuhwi R., E Usambaras, 900 ft, *Greenway 4959* (K).—Region undetermined: T6, Nahomba, Selous Game Reserve, 7 Nov 1970 (fr), *Ludanga 1166* (K); Kijawe, Selous Game Preserve, 2 Feb 1971 (fl), *Ludanga 1193* (EA, K); T6, Selous Game Reserve, 24 May 1978 (fr), *Mhoro 2917* (MO); T8, Balani water hole, Selous Game Reserve, 4 Oct 1970 (fl), *Rodgers 1148* (K).

*Xylopiaholtzii* is another member of the *X.odoratissima* subgroup of eastern and southern Africa, set apart from other species of the subgroup by the combination of branched inflorescences, purple coloration at the bases of the petals, and pubescent stipitate monocarps that are only weakly verrucose. The number of branched inflorescences on herbarium specimens varies—some sheets of *Vollesen 2992* from the Selous region, for example, have no branched inflorescences, but correspond to the species in leaf and floral morphology. In Tanzania, *X.holtzii* is not known to overlap in distribution with either *X.shirensis* or *X.gracilipes*. The former is a species of the interior miombo woodlands to the west of the Eastern Arc Mountains, while the latter is only known from the miombo woodlands of the extreme southern Tanzania, where it reaches the northern limit of its distribution.

Given the variable nature of the habitats in which this species occurs, it is not surprising that tree associates vary correspondingly from location to location. The most frequently mentioned associates are *Miliciaexcelsa* and species of *Erythrophleum* and *Diospyros*. Marshall (2007) reported *X.holtzii* (as *X.parviflora*) to be one of the ten most common canopy tree species in disturbed semi-deciduous forest plots in the Udzungwa Mountains, comprising 7.5% of the 3346 canopy tree individuals sampled.

The type specimen of *Xylopiaholtzii* cited above, which was marked as the Typus at B, bears information slightly different from that which is included in Engler’s protologue: “Sansibarküstgebiet: Pugu-Berge in Buschgehölzen auf rotem Lehm (Holtz n. 897 – Fruchtend im Feb 1903).” The specimen is not discordant with the protologue, but in the case that there was a specimen with this number that has now been lost, the extant specimen is designated as a lectotype rather than assumed to be the holotype.

### 18. Xylopia keniensis

Taxon classificationPlantaeMagnolialesAnnonaceae

D. M. Johnson, Kew Bull. 72:11: 10–12. 2017.

555923DA-3C51-5123-AB73-09395E9D3E71

Fig. 28C–E

#### Type.

KENYA. Kwale District, Shimba Hills, 4.2407°S, 39.4218°E, 390 m, 30 Dec 2009, *Q. Luke & P. Luke 13949* (holotype EA!; isotypes BR, K! MO! [6467367, 6569824], NHT, PRE, US).

#### Description.

***Tree*** 15–25 m tall with long straight bole and rounded crown. ***Twigs*** greenish brown to blackish, glabrous or initially short-pubescent, the hairs 0.1–0.3 mm long, but soon light gray, glabrate; nodes occasionally with two axillary branches. ***Leaf*** with larger blades 6.7–10.5 cm long, 2.6–3.7 cm wide, chartaceous, shiny adaxially, dull and paler abaxially, lanceolate to elliptic, occasionally oblong or elliptic-oblanceolate, apex sharply acuminate, the acumen 6–18 mm long, occasionally obtuse, base cuneate to rounded, glabrous or with a few hairs at the base of the midrib adaxially, sparsely appressed-pubescent to glabrate abaxially; midrib slightly raised to plane adaxially, raised abaxially, secondary veins weakly brochidodromous, 12–15 per side, diverging at 60–75° from the midrib, these and higher order veins slightly raised on both surfaces; petiole 2–5.5 mm long, shallowly canaliculate, pubescent to glabrous. ***Inflorescences*** axillary, usually from the axils of fallen leaves, 1- or rarely 2-flowered, sparsely pubescent; pedicels 2.8–3.9 mm long, 0.6–0.7 mm thick; bracts 2, attached to the distal half of the pedicel, persistent or not, 1.2–1.6 mm long, broadly ovate to crescent-shaped, apex acute; buds lanceolate, apex acute. ***Sepals*** erect to slightly spreading at anthesis, 1/3-connate, 2.2–2.7 mm long, 2.3–2.4 mm wide, subcoriaceous, broadly ovate to triangular, apex obtuse to acute, sparsely pubescent abaxially. ***Petals*** cream-colored with reddish pink bases at anthesis *in vivo*; outer petals erect or slightly spreading at anthesis, 13.7–19.3 mm long, 2.2–2.6 mm wide at base, 0.9–1.0 mm wide at midpoint, coriaceous, linear, flat on both surfaces, apex obtuse, densely puberulent except for the glabrous warty base adaxially, golden-sericeous abaxially; inner petals geniculate at anthesis, with the apices bent sharply outward between the outer petals, 11.0–16.3 mm long, 1.8–2.2 mm wide at base, ca. 0.5 mm wide at midpoint, coriaceous, linear-subulate, longitudinally ridged on both surfaces, transversely thickened at widest point adaxially, apex acute, base with undifferentiated margin, densely puberulent on both surfaces except for the glabrous base. ***Stamens*** ca. 85; fertile stamens 1.0–1.3 mm long, narrowly oblong, apex of connective reddish pink at anthesis *in vivo*, 0.2–0.25 mm long, shieldlike, overhanging the anther thecae, smooth or finely papillate, anthers 7–10-locellate, filament 0.3–0.5 mm long; outer staminodes 1.2–1.5 mm long, oblong, apex rounded to truncate, sometimes emarginate; inner staminodes 1.0–1.1 mm long, oblong, apex truncate; staminal cone ca. 1.3 mm in diameter, 0.7 mm high, concealing only the bases of the ovaries, rim laciniate. ***Carpels*** ca. 6; ovaries ca. 0.3 mm long, oblong, pubescent; stigmas connivent, free at the apices, 1.8–2 mm long, filiform, apex acute, with a few hairs at the apices. ***Torus*** flat, ca. 1.8 mm in diameter ***Fruit*** of up to 4 glabrate monocarps borne on a pedicel ca. 5 mm long, ca. 2.6 mm thick, glabrate; torus ca. 4 mm in diameter, ca. 4.5 mm high, globose. ***Monocarps*** with a green exterior and red endocarp *in vivo*, 2.8–3.5 cm long, 1.0–1.6 cm wide, 1.1–1.4 cm thick, clavate-pyriform to ovoid, usually with a longitudinal ridge running down one side, apex obtuse, base tapered but not stipitate, verrucose; pericarp 0.4–1.0 mm thick. ***Seeds*** 4–6 per monocarp, in two rows, lying perpendicular to long axis, 11.0–11.2 mm long, 7.2–7.3 mm wide, ca. 5.1 mm thick, oblong to ellipsoid, obovate to elliptic in cross-section, obliquely truncate at micropylar end, rounded at chalazal end, dark brown, slightly wrinkled/roughened, dull, raphe/antiraphe not evident, micropylar scar ca. 3 mm long, 1.5 mm wide, elliptic; sarcotesta orange-red *in vivo*; aril absent.

**Figure 28.**
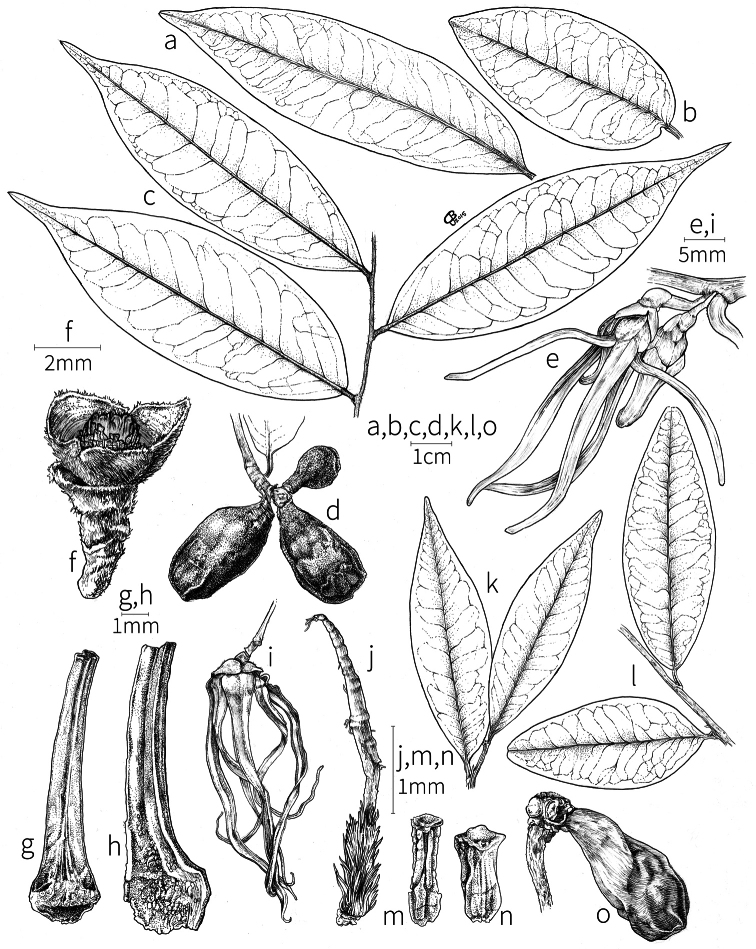
*Xylopiakeniensis* and *X.tanganyikensis*, with leaves of *X.holtzii* and *X.longipetala* for comparison. **A** Leaf of *X.holtzii* from Tanzania **B** Leaf of *X.longipetala* from Ghana **C–E***X.keniensis***C** Habit **D** Fruit **E** Inflorescence **F–O***X.tanganyikensis***F** Pedicel, sepals, and staminal cone **G** Base of inner petal, adaxial view **H** Base of outer petal, adaxial view **I** Flower, lateral view **J** Carpel **K, L** Habit **M** Stamen, abaxial view **N** Staminode, abaxial view **O** Fruit. **A** from *Semsei 1718* (K) **B** from *Jongkind & Nieuwenhuis 2130* (WAG) **C, D** from *Luke & Robertson 2723* (MO) **F–J, L–N** from *Nishida 57* (K) **K, O** from *Abeid et al. 1028* (L). Reproduced with the permission of the Board of Trustees, Royal Botanic Gardens Kew.

#### Phenology.

Specimens with flowers or flower buds have been collected in November and December, and with fruits in March and September.

#### Distribution

(Fig. 22). Endemic to the Shimba Hills region of southern Kenya, where it grows in lowland forest at elevations of 350–430 m (Luke 2005). Associates at one site (from *Luke & Robertson 2723*) included species of *Antiaris*, *Milicia*, *Lovoa*, *Celtis*, *Quassia*, *Hymenaea*, *Julbernardia*, and *Manilkara* in the canopy, and *Leptonychia*, *Diospyros*, *Warneckea* [*Memecylon*], and many Rubiaceae shrubs in the understory.

#### Additional specimens examined.

**KENYA.** Kwale: Shimba Hills, Makadara, 4.2394 S 39.3937 E, 430 m, [typed label gives locality as Taita Hills, which is a transcription error (R. Faden, personal communication)], 9 Nov 1970 (buds), *Faden et al. 70/847* (K); Shimba Hills, Longomwagandi, 4.2407 S 39.42181 E, 390 m, 30 Dec 2009 (st seedling), *Luke & Luke 13950* (EA); Shimba Hills, Longomwagandi, 4.2351 S 39.4181 E, 390 m, 17 March 1991 (fr), *Luke & Robertson 2723* (EA, K, MO); Shimba Hills, 1 Jul 2006, *Project Mbegu 353* (EA); Shimba Hills, 20 Jul 1967 (st), *Mulwa 015* (EA); Kwale District, Shimba Hills, Lango ya Mwagandi or Longo Mwagandi Forest, 4.2333 S, 39.4167 E, 350 m, 18 Sep 1982 (fr), *Polhill & Robertson 4800* (K).

*Xylopiakeniensis* overlaps in distribution with *X.holtzii*, but it differs from that species in the sharply acuminate and nearly glabrous leaf blades, the pedicels short and usually with only one flower per axil, the inner petals strongly geniculate at anthesis, and the sessile monocarps with the seeds arranged in two rows. In seedlings grown side by side, the more pronounced sheen of the *X.keniensis* leaves is evident (Q. Luke, personal communication).

The species is likely to occur in the Usambara Mountains region of Tanzania, where it may be confused with *X.holtzii*. As currently known, *X.keniensis* is very rare, and was given a conservation assessment of Endangered B1ab(iii)+B2ad(iii) by Johnson et al. (2017). We determined it to have the smallest AOO of any African species, 10 km^2^.

### 19. Xylopia nilotica

Taxon classificationPlantaeMagnolialesAnnonaceae

D. M. Johnson & N. A. Murray sp. nov.

CCCDE9DB-1117-5934-BCE5-68EA94BB52F7

urn:lsid:ipni.org:names:60476242-2

Fig. 29

#### Diagnosis.

Species resembling *Xylopiaholtzii* in its branched inflorescences and leaf blades adaxially hairy, but differing in the higher-order veins of the leaf equal in prominence to the secondary veins and forming a conspicuous raised reticulum on the adaxial surface, and the monocarps conspicuously verrucose but not much wrinkled, with stipes 3.5–5 mm thick.

Type: UGANDA. Northern Region, Leya and Aiyu river junction, W. Madi, 25 Mar 1945, *P. J. Greenway & W. J. Eggeling 7251* (holotype: EA!; isotypes: K! PRE!).

#### Description.

***Tree*** up to 25 m tall with a narrow crown, bole straight, fluted with small sharp buttresses at the base; bark light gray to grayish brown, sometimes exfoliating in irregular patches. ***Twigs*** brown to reddish brown, brownish gray, or blackish brown, sparsely pubescent, the hairs 0.2–0.5 mm long, eventually brownish black to dark gray, glabrate; nodes occasionally with two axillary branches. ***Leaf*** with larger blades (4.1–) 6.1–9.4 cm long, 2.2–3.2 cm wide, chartaceous, concolorous, oblong to lanceolate-oblong, apex obtuse to rounded, occasionally retuse or emarginate, base obliquely cuneate to rounded, sparsely pubescent (densest on the midrib) to glabrous adaxially, sparsely appressed-pubescent abaxially; midrib slightly raised to plane adaxially, raised abaxially, secondary veins weakly brochidodromous, 8–13 per side, diverging at 40–70° from the midrib, these and higher-order veins raised on both surfaces; petiole 3.5–8 mm long, semi-terete to shallowly canaliculate, pubescent. ***Inflorescences*** axillary, 1–3-flowered, pubescent; peduncle 1 per axil, 2.0–5.5 mm long; pedicels 2–3 per peduncle, (1.0–) 2.5–7.5 mm long, 0.5–0.9 mm thick; bracts 2, at or just distal to the pedicel midpoint, caducous or the uppermost persistent, ca. 1.6 mm long, broadly ovate, apex obtuse; buds linear-lanceolate, slightly falciform, apex acute, base globose. ***Sepals*** slightly spreading at anthesis, 1/3–1/2-connate, 1.5–2.5 mm long, 2.2–3.3 mm wide, coriaceous, semicircular to broadly triangular, apex acute, pubescent abaxially. ***Petals*** pale green to yellow-green *in vivo*; outer petals erect to somewhat spreading at anthesis, 11.7–27 mm long, 2.3–2.9 mm wide at base, 1.0–1.2 mm wide at midpoint, coriaceous, linear, apex acute, longitudinally ridged on abaxial surface, gray-puberulent except for glabrous base adaxially, golden-pubescent abaxially; inner petals curved outward or weakly geniculate at anthesis, 11.5–16.8 mm long, ca. 2.4 mm wide at base, ca. 0.7 mm wide at midpoint, coriaceous, linear-subulate, apex acute, base with undifferentiated margin, longitudinally ridged on both surfaces, densely puberulent on both surfaces except for the glabrous base. ***Stamens*** ca. 70–80; fertile stamens 1.0–1.3 mm long, narrowly oblong, apex of connective 0.1–0.2 mm long, depressed-globose to shieldlike, overhanging the anther thecae, glabrous, anthers 8–11-locellate, filament 0.3–0.5 mm long; outer staminodes 1–1.3 mm long, broadly clavate, apex rounded to truncate; inner staminodes 0.8–1 mm long, clavate, apex truncate; staminal cone 0.8–1.9 mm in diameter, 0.7–1.1 mm high, concealing only the bases of the ovaries, rim even to laciniate. ***Carpels*** ca. 7; ovaries 0.8–0.9 mm long, narrowly oblong, pubescent, stigmas loosely connivent, 2–2.5 mm long, linear, apices pubescent. ***Torus*** flat, 1.6–2.7 mm in diameter. ***Fruit*** of up to 12 sparsely pubescent monocarps borne on a pedicel 7–8.5 mm long, 2.5 mm thick, glabrate; torus 5–7.5 mm in diameter, 5.5–7 mm high, globose to depressed-globose. ***Monocarps*** 2.3–4.2 cm long, 0.9–1.3 cm wide, ca. 1.2 cm thick, oblong, weakly torulose, apex obtuse to rounded and somewhat flattened, base contracted into a stipe 6–9 mm long, 3.5–5 mm thick, obliquely wrinkled, strongly verrucose; pericarp 1 mm thick. ***Seeds*** up to 3 per monocarp, in a single row, lying oblique to long axis, 11.8–12.5 mm long, ca. 6.7 mm wide, ca. 5.8 mm thick, oblong-ellipsoid, semicircular in cross-section, narrowed and truncate at micropylar end, rounded at chalazal end, brown, smooth, shiny or dull, raphe/antiraphe not evident, micropylar scar ca. 2 mm long, ca. 1.5 mm wide, elliptic; sarcotesta red to orange *in vivo*; aril absent.

**Figure 29.**
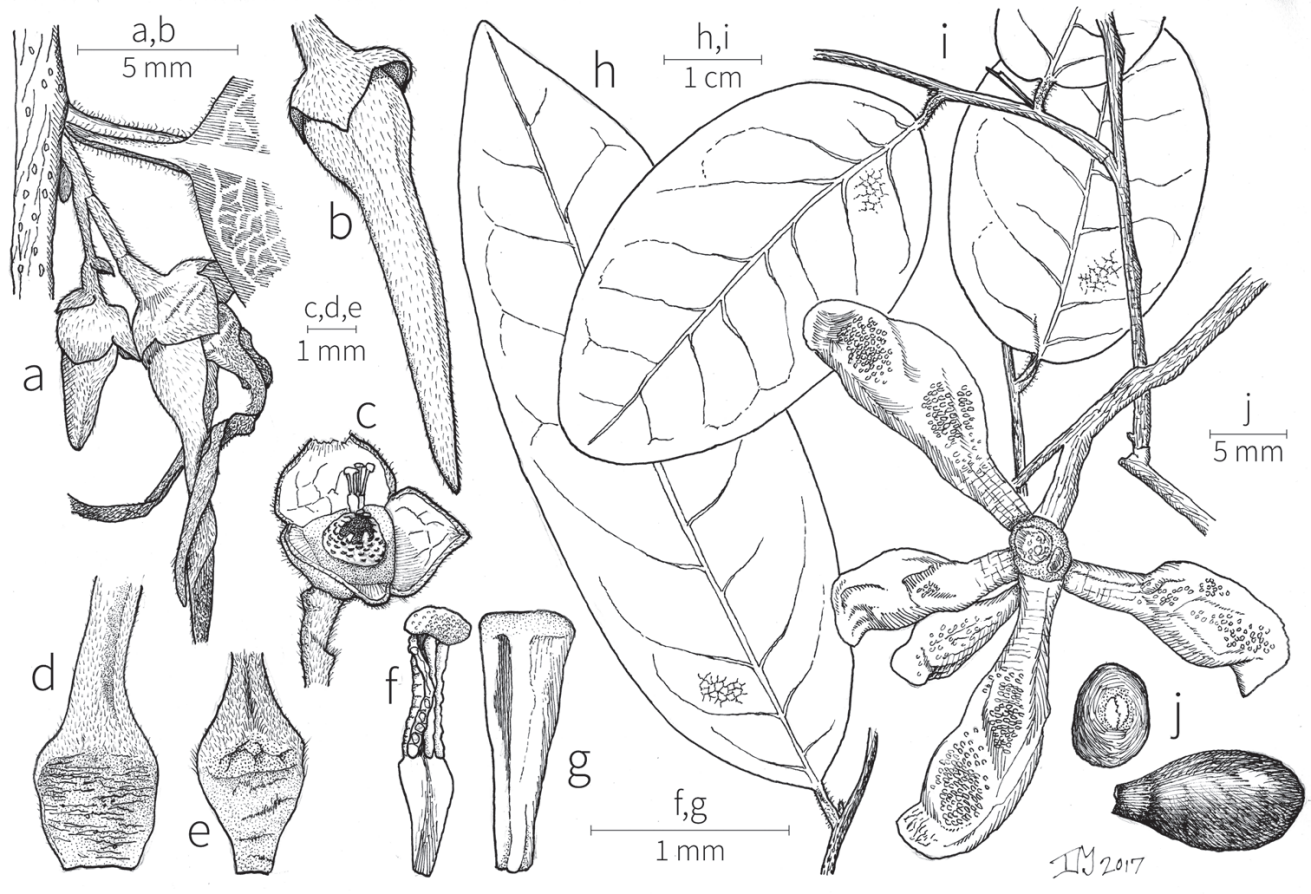
*Xylopianilotica*. **A** Close-up of inflorescence, showing two pedicels branching from the same peduncle **B** Flower bud, lateral view **C** Receptacle with petals, stigmas, and most of the stamens fallen, showing staminal cone **D** Base of outer petal, adaxial view **E** Base of inner petal, adaxial view **F** Fertile stamen, abaxial view **G** Outer staminode, abaxial view **H** Leaf **I** Habit, including leaves and fruit **J** Seed, view of micropylar end and side view. **A, H** from *Greenway & Eggeling 7251* (K) **B** from *Wood 610* (K) **C–G** from *Philip 931* (K) **I** from *Greenway & Eggeling 7251* (PRE) **J** from *Smith 39* (K).

#### Phenology.

Specimens with flowers have been collected from February to May and in July, and with fruits from March to May.

#### Distribution

(Fig. 26). Occurs in upland and lower montane semideciduous forests, one specimen from along a river, in central Sudan, South Sudan, and northern Uganda, all from the upper Nile River watershed, at elevations between 760 and 1220 m.

#### Local names.

Kharûm (Eliri Arab, *Simpson 7778*), munnu (Anuak, *Simpson 7778*).

#### Additional specimens examined.

**SUDAN.** Gebel Amira, Nuba Mts., 16 Apr 1930 (buds), *Simpson 7778* (K—2 sheets); Eastern KMagnolialesfan Province, Jebel Dair, 4000 ft, 17 Jul 1937 (fl), *Turner 241* (K). **SOUTH SUDAN.** West Equatoria: “Large tree of Azza,” [Azza forest is probably SE of Maridi, I. Darbyshire, personal communication, 2016], 28 Apr 1933 (fr), *Smith 39* (K). **UGANDA.** West Nile, Koich River, Rumogi, Mar 1935 (fl), *Eggeling 1650* (K); Madi, 830 m, Feb 1961 (fl), *Philip 931* (EA, K); Murchison Falls National Park, Rabongo Forest, 02°06'N, 31°52'E, 1020 m, 1 May 1993 (fl, fr), *Sheil 1443* (K); Busoga District, Igwe mutalla, 10 mi S of Bugiri, 1260 m, 20 May 1951 (fl), *Wood 610* (EA, K—2 sheets).

*Xylopianilotica* is most similar to *X.holtzii* of coastal Kenya and Tanzania, but is distinguished by the more prominent and raised reticulum of the adaxial leaf surface, with the higher-order veins equal in prominence to the secondary veins, and the strongly verrucose monocarps with thicker stipes. While *Xylopianilotica* is more similar to *X.holtzii* than to *X.longipetala*, it is nearer to the latter geographically, differing in being a large forest tree of upland or even submontane forest rather than a riparian shrub or small tree, and in having much shorter petals and distinctly stipitate monocarps.

*Xylopianilotica* occupies the northernmost distribution of any *Xylopia* species in Africa. Associates occurring at more than one locality include *Holopteleagrandis*, *Miliciaexcelsa*, and species of *Khaya*. Several sterile specimens probably represent this species: SUDAN: *Broun & Broun 1373* (K), *Longe 40*, local name dooru (K), *Myers 10102* (K); ETHIOPIA: *Chaffey & Thomerson 658*, local name orowyee (Anuak) (EA, K), *Friis et al. 2485* (K).

### 20. Xylopia odoratissima

Taxon classificationPlantaeMagnolialesAnnonaceae

Welwitsch ex Oliver, Fl. trop. Afr. 1: 31. 1868.

1708F69F-FF29-58E1-B799-C3D59BAA2CF2

Fig. 3G

Xylopicrum odoratissimum (Welwitsch ex Oliver) Kuntze, Revis. gen. pl. 1: 8. 1891. Type. ANGOLA. Huíla Province, [Morro de Lopollo, 1859-60, ex Hiern (1896, p. 10)], *F. Welwitsch distrib. no. 757* (lectotype: BM! [000511059, flowering specimen on lower half of sheet], photos GH! NY!).
Xylopia antunesii Engler & Diels, Notizbl. Königl. Bot. Gart. Berlin 2: 299. 1899.
Xylopicrum antunesii (Engler & Diels) Kuntze, Deutsch. Bot. Monatsschr. 21: 173–174. 1903. Type. ANGOLA. Benguella, Huíla, *J. M. Antunes 64* (holotype: B! [100153150]).

#### Description.

***Shrub*** or tree up to 9 m tall, d.b.h. up to 15 cm, with wide-spreading crown; bark light gray to brown, smooth or flaking. ***Twigs*** brown to dark brown, densely pubescent, the hairs 0.2–0.6 mm long, eventually light gray, reddish brown or brownish gray, glabrate; nodes occasionally with two axillary branches. ***Leaf*** with larger blades 4.7–6.9 cm long, 2.2–3.3 cm wide, subcoriaceous to chartaceous, concolorous, oblong, lanceolate-oblong, or elliptic, occasionally ovate, oblanceolate, or obovate, apex obtuse or rounded, occasionally broadly acute, base broadly cuneate to rounded, sparsely pubescent (usually denser on the midrib) adaxially, pubescent abaxially; midrib raised to plane adaxially, raised abaxially, secondary veins weakly brochidodromous to camptodromous, 6–11 per side, diverging at 45–70° from the midrib, slightly raised on both surfaces, higher-order veins raised on both surfaces; petiole 3.5–8 mm long, shallowly canaliculate, densely pubescent. ***Inflorescences*** axillary, 1–3-flowered, the pedicels arising separately from axil, only rarely 2 from a short common peduncle, pubescent; pedicels 3.5–7 mm long, 1.3–1.5 mm thick, pubescent; bracts 1–2, attached near the midpoint, caducous, 1.1–2 mm long, semicircular, apex obtuse to rounded, pubescent abaxially; buds linear, often falciform, apex acute, base bulbous. ***Sepals*** somewhat spreading at anthesis, 1/4–2/5-connate, 2.0–3.0 mm long, 2.3–4.5 mm wide, coriaceous, broadly triangular to semicircular, apex acute to obtuse, pubescent abaxially. ***Petals*** creamy yellow, greenish yellow, or dull yellow *in vivo*; outer petals spreading to recurved at anthesis, 17.2–33.5 mm long, 3.4–3.9 mm wide at base, 1.0–1.7 mm wide at midpoint, coriaceous, linear, often somewhat falcate, apex acute to obtuse, keeled on abaxial surface, gray-puberulent adaxially, yellow-brown sericeous abaxially; inner petals spreading to recurved at anthesis, 12.8–24.6 mm long, 2.1–3.0 mm wide at base, 0.6–1.0 mm wide at midpoint, coriaceous, linear, apex acute, base with undifferentiated margin, longitudinally ridged on both surfaces, densely puberulent on both surfaces except for glabrous base. ***Stamens*** 160–200; fertile stamens 1.1–1.6 mm long, narrowly oblong to clavate, apex of connective 0.1–0.3 mm long, shieldlike, overhanging anther thecae, glabrous or minutely papillate, anthers 9–12-locellate, filament 0.3–0.5 mm long; outer staminodes 1.6–1.8 mm long, quadrate or broadly clavate, apex obtuse to truncate; inner staminodes 0.9 mm long, broadly clavate, apex truncate; staminal cone 1.7–2.4 mm in diameter, 1.0–1.4 mm high, completely concealing the ovaries, rim even. ***Carpels*** 7–10; ovaries ca. 0.8 mm long, stigmas more or less connivent, 2.5–2.6 mm long, linear, apices acute, with a tuft of yellow to rusty hairs. ***Torus*** slightly concave beneath the ovaries, otherwise flat, 1.9–2.8 mm in diameter. ***Fruit*** of up to 7 glabrate monocarps borne on a pedicel 7–8 mm long, 1.9–2.5 mm thick, sparsely pubescent; torus of fruit ca. 3 mm in diameter, ca. 2.5 mm high, ovoid to globose. ***Monocarps*** with green to red exterior *in vivo*, endocarp color unknown, 1.8–2.6 cm long, 1.0–1.3 cm wide, ca. 0.8 cm thick, oblong, sometimes weakly torulose, apex obtuse with a short beak 1–2 mm long, base subsessile or contracted into a stipe 2.5–7 mm long, 2.5 mm thick, strongly rugose, occasionally pruinose; pericarp 0.2–0.4 mm thick. ***Seeds*** up to 4 per monocarp, in a single row, lying oblique to perpendicular to long axis, 10.3–12.2 mm long, 6.7–7.7 mm wide, 5.1–6.1 mm thick, ellipsoid, semicircular in cross-section, narrowed and truncate at micropylar end, rounded at chalazal end, brown, smooth, dull, raphe/antiraphe not evident, micropylar scar 1.5 mm long, 1.0–1.5 mm wide; sarcotesta red *in vivo*; aril absent.

#### Phenology.

Specimens with flowers have been collected from January to May and those with fruits from December to February.

#### Distribution

(Fig. 26). Southern Angola and northeastern Namibia, east to western Zambia and south to northern Botswana and west-central Zimbabwe; most frequently reported from woodlands on Kalahari sands dominated by *Baikiaeaplurijuga* (mukusi, Rhodesian teak), occasionally in miombo woodland dominated by *Brachystegiaspiciformis*, or occasionally from the edges of riverbanks; 900–2000 m.

#### Local names.

Mtjibi wenduna (Sindebele, *Goldsmith 51/56*), omuriandele (*Antunes & Dekindt 3142*), setundewanga (Sik, *Pardy 4805*), situndu bawanga (Lozi, *White 2062*), situnduboanga (Barotze, *Jenkins 1*), situndubwanga (“Bantu,” *McFerren 30*), umtshibi omduna (Ndebele, *Mashasha 169*). On the label of *White 2062*, it is explained that the name situndu bawanga is translated as “That which cannot be left,” attesting to the medicinal properties attributed to the plant by local people.

#### Additional specimens examined.

**ANGOLA.** Huíla, clairieres de la montagne de Lopolo, 1800 m, Dec 1900 (fr), *Antunes & Dekindt 3142* (P–3 sheets); Huíla, Polygonal Florestal da Humpata, ca. 5 km NE of Humpata, ca. 8 km SW of Lubongo, 14°58'54.9"S, 13°26'01.6"E, 2018 m, 26 Jan 2009 (fl), *Bester 9279* (MO); Humber A bords du Cunene, Tyipelongo-Mucope-Danguena, 1910–1920 (fl, fr), *Bonnefousc & Villain 92°2* (P); at Kubango near the forte Princeza Amelia, s. d. (fl), *Gossweiler 3940* (BM, K); Bié, Kubango, 1905 (fr), *Gossweiler 4043* (BM); Huíla, Mannyino, 1760 m, Jan 1901 (fl), *Herbar. Huíla 276* (A); Huíla [or Bié], Ganguelas, Vila Artur de Paiva, margens do Cubango, 1450 m, 12 Jan 1960 (fl), *Mendes 2086* (MO); Huíla, Huíla, próx. da M. Catolica do Mennhino, 6 Feb 1955 (fl), *Santos 92* (MO, WAG); Huíla, Quilengues, Lucondo, ca. 1500 m, 28 Oct 1959 (st), *Teixeira & Andrade 358* (PRE); Huíla, prope Lopolo, Jan 1860 (fr), *Welwitsch 92* (BM). **NAMIBIA.** Eastern Caprivi, Katima Mulilo District, Katima Mulilo, 17 Feb 1971 (fl), *Breitenbach 1312* (PRE); Kavango Andara at camp of Dept. Agriculture, Grid Ref. 1821 AB, 10 Feb 1958 (fl), *Hilbert 158* (K); Eastern Caprivi, Katima Mulilo District, Katima Mulilo, 11 Feb 1968 (fl), *McFerren 30* (PRE); Andara, R. C. Miss. Station, on Island near camp on bank of river, 24 Apr 1977 (fl), *Müller & Giess 533* (PRE); Okavango Territory, Andara Camp, Okavango River banks, 18 Mar 1966 (fl), *Tinley 1414* (PRE, WAG); O Caprivi, Katima Mulilo, 30 Jan 1975 (fl), *Vahrmeijer & du Preez 2508* (PRE). **ZAMBIA.** Southern: Livingstone District, Livingstone, 12 Feb 1956 (fl), *Gilges 586* (K, PRE); Livingstone, 12 Feb 1930 (fl), *Jenkins 1* (BM); Livingstone, 22 Feb 1963 (fl), *Lawton 1041* (K).—Western: Mongu District, Makapaela Pan, 25 km N of Mongu, 15°02'S, 23°14'E, 1020 m, 15 Feb 1999 (fl, *Bingham & Luwiika 11893* (K); Mongu District, Kataba local forest, 20 km [“mm”] SE of Mongu, 15°27'S, 23°16'E, 1030 m, 16 Feb 1999 (fr), *Bingham & Luwiika 11905* (K); Shisheke District 103 km SE of Senanga on road to Sesheke, 1050 m, 1 Feb 1975 (fl), *Brummitt et al. 14224* (EA, MO, RSA, WAG); Kalabo District [“Barotse Province”], near Kalabo Boma, 13 Feb 1952 (fl), *White 2062* (BM, K); Kalabo District, 5.6 km W of Kama Health Center, 5.5 km E of Lukona Secondary School in Kalamaba L. F. No. 379, 15°22'36.9"S, 22°53'44.5"E, 930 m, 21 Mar 1996 (fl), *Zimba et al. 829* (MO, PRE).—Province unknown: Baikiaea forest region, Kalahari sand, S. N. Western Rhodesia, 1937 (st), *Martin 786* (BM). **BOTSWANA.** Kazungula, Apr 1936 (fl), *Miller B 132* (BM). **ZIMBABWE.** Wankie District, Fuller Forest Reserve, Mar 1960 (fl), *Armitage 102/60* (MO); Victoria Falls, 10–15 Mar 1932 (fl), *Brain 8875* (K, MO); Shangani/Bubi District, Gwampa Forest Reserve, 3000 ft, Feb 1956 (fl), *Goldsmith 51/56* (K, PRE); Wankie District, Victoria Falls National Park, 14 Feb 1980 (fl), *Kandanda 11* ([= s. n. at B?], MO); Wankie District, Victoria Falls village, 24 Jan 1979 (fl), *Mshasha 169* (K, MO, WAG); Nyamanahlovu Dist., Jan 1929 (fr ped), *Pardy 4502* (MO); Nyamanahlovu Dist., Jan 1931 (fl), *Pardy 4805* (B, BM, K); Bulawayo, May 1898 (st), *Rand 422* (BM).

*Xylopiaodoratissima* is a shrub or spreading treelet with uniformly small leaves and with an inflorescence consisting of 1–3 flowers. The petals are long and often falcate at the apices, even in bud. The outer petals are widened abruptly at the base, such that the flower buds appear bulbous. The monocarps are reported to be red at maturity, and are prominently rugose when dried. Purple coloration at the bases of the inner petals is apparently absent (Fig. 3G). *Xylopiaodoratissima* is a species restricted to the *Baikiaea* and *Brachystegia* woodlands on Kalahari sands from Angola to western Zimbabwe, where it occurs with *Pseudolachnostylis* and *Parinarimobola* as associates. It seems not to occur north or east of the Zambezi River, where it is replaced by *Xylopiashirensis*, which, while similar, can grow to be a tree up to 14 m tall and has larger leaves that are more variable in shape. The petals of *X.shirensis* are shorter and seldom falcate, and the inner petals have purple coloration at the base. The outer petals narrow gradually, and the widening of the base of the buds is therefore gradual as well. The monocarps are reported to be green at maturity, and are smooth or at most weakly wrinkled when dried.

Collectors frequently remark on the fragrance of the flowers. Welwitsch (as translated by Hiern 1896) wrote “flowers very fragrant, exceeded in the pleasantness of the aroma only by the species of *Schrebera* but their fragrance is far more intense.” The label of *Jenkins 1*, from southern Zambia, states “scent much resembling a sweet pea. Only noticeable after sunset. Then the tree → v. fragrant.”

Typification of names based on Welwitsch collections is problematic because Welwitsch held his specimens privately, and they were not distributed to herbaria until after his death. As explained by Albuquerque et al. (2009), the bulk of the collection was returned to LISU during the period 1876 to 1879. The second-best set remained at BM, and duplicates were distributed from LISU to other herbaria later on.

In this particular case, Welwitsch must have shown his collections to Daniel Oliver for use in preparing the first volume of the Flora of Tropical Africa, published in 1868, that is, while they were still in Welwitsch’s private collection and had not been divided up into sets. Oliver’s protologue gives a detailed description of the plant, in particular of the flowers. The fruits are described briefly and the seeds not at all. One of the specimens with flowers therefore seems to be the most appropriate choice as a lectotype.

According to Hiern (1896), *Xylopiaodoratissima* was found by Welwitsch in flower in December of 1859 and in January and February of 1860. Welwitsch numbered his collections as he curated them, grouping together all collections thought to represent the same species under the same collection number (Albuquerque et al. 2009). All of the collections seem to have come from the same locality, so the only piece of information that can separate them is the date. Unfortunately, most of the duplicates seen lack a specific date.

A branch with flowers in the lower half of sheet 000511059 at BM is chosen as the lectotype. It is accompanied by a handwritten label that specifies Lopollo Dec. 1859. Photographs of this specimen have been distributed to a number of herbaria. In the absence of information about the date of collection, however, other specimens with flowers are not considered isolectotypes, although with better information, they may be identifiable as such in the future. For example, some of the specimens with flowers have leaves that have dried a darker color than those of the BM specimen. A specimen at LISU, which we viewed through JSTOR Global Plants, has been annotated as the “holotype,” but it cannot be so, given that the original description was based upon multiple gatherings. In addition, the date given on the specimen is April 1860, which does not accord with the flowering times for the species given in Hiern (1896), and we cannot see the specimen well enough to tell if it has flowers or fruits on it. For the record, we have seen *Welwitsch 757* specimens from B (single sheet, flowering specimen, printed ticket without date, only information written on the label is 757 *Xylopiaodoratissima* Welw.), BM (non-lectotype material on sheet 000511059), C (two sheets, seen via JSTOR), G (single sheet, specimen with flowers but leaves are darker than on BM sheet), K, LD, LISU (2 sheets), M, P (2 sheets, one ex herb. Pierre), and PRE.

The syntype *Smith s. n.*, from the Congo River, is *Xylopialongipetala* and does not seem to have contributed significantly, if at all, to the protologue of *Xylopiaodoratissima* and is therefore excluded as a possible lectotype.

### 21. Xylopia shirensis

Taxon classificationPlantaeMagnolialesAnnonaceae

(Engler & Diels) D. M. Johnson & N. A. Murray comb. nov.

E122CC65-2997-58EE-A813-F703A31043E5

urn:lsid:ipni.org:names:60476247-2

Fig. 27M–P

Xylopia antunesii var. shirensis Engler & Diels, Monogr. afrik. Pflanzen-Fam. 6: 66. 1901. Type. MALAWI. Southern Region, Shiri Highlands [“Shire Hochland”], Chnambola, comm. 1881, *J. Buchanan 237* (holotype: K!).

#### Description.

***Shrub*** or a tree up to 14 m tall, d.b.h. up to 20 cm; bark cracking. ***Twigs*** brown to dark brown, initially tomentose, the hairs 0.3–1.2 mm long, becoming light brown to gray or dark gray-brown, glabrate or with pubescence somewhat persistent; nodes occasionally with two axillary branches. ***Leaf*** with larger blades 4.5–10.2 cm long, 2.1–5.1 cm wide, chartaceous to subcoriaceous, concolorous or slightly discolorous, elliptic, oblong, or ovate, rarely oblanceolate, apex broadly acute, obtuse, or rounded, rarely nearly truncate or emarginate, base broadly cuneate to rounded, occasionally truncate or subcordate, sparsely pubescent on both surfaces; midrib slightly raised to plane adaxially, raised abaxially, secondary veins irregularly brochidodromous, 7–15 per side, diverging at 45–70° from the midrib, these and higher-order veins slightly raised on both surfaces; petiole 3.5–11 mm long, shallowly canaliculate or somewhat flattened, pubescent. ***Inflorescences*** axillary or from axils of fallen leaves, 1–6-flowered, pedicels emerging side by side from axil or rarely from a common peduncle, pubescent; peduncle ca. 3.5 mm long, with 2 pedicels per peduncle; pedicels 2.8–7 mm long, 1.2–1.5 mm thick; bracts 2, at or proximal to midpoint, caducous or somewhat persistent, lower 1.9–2.6 mm long, upper 1–1.7 mm long, semicircular to ovate, apex obtuse or rounded; buds lanceolate, apex more or less acute. ***Sepals*** slightly spreading at anthesis, 1/4–1/2-connate, 1.5–3.0 mm long, 2.3–3.4 mm wide, chartaceous to coriaceous, triangular, broadly ovate, or semicircular, apex acute, pubescent abaxially. ***Petals*** yellow-green to pale yellow, the inner petals crimson or purple-tinged on the inner base *in vivo*; outer petals spreading at anthesis, 12.2–20.9 mm long, 2.3–3.3 mm wide at base, 1.0–1.6 mm wide at midpoint, coriaceous, linear-lanceolate, apex obtuse, densely pubescent on both surfaces, the pubescence more yellowish abaxially; inner petals spreading to weakly geniculate at anthesis, 9.3–14.6 (–21.5) mm long, (1.6–) 2.0–2.9 mm wide at base, 0.5–1.0 mm wide at midpoint, coriaceous, linear-subulate, apex obtuse, base with undifferentiated margin, densely gray-puberulent on both surfaces, except for glabrous base. ***Stamens*** ca. 120; fertile stamens 1.0–1.2 mm long, narrowly oblong, apex of connective 0.1–0.2 mm long, shieldlike, overhanging anther thecae, glabrous, anthers 8–11-locellate, filament 0.3–0.5 mm long; outer staminodes 1.2–1.4 mm long, clavate to oblong, apex obtuse, rounded, or truncate; inner staminodes ca. 0.8 mm long, rectangular, apex truncate; staminal cone 1.2–2.3 mm in diameter, 0.8–1.1 mm high, concealing only the bases of the ovaries, rim laciniate. ***Carpels*** ca. 7; ovaries ca. 1 mm long, narrowly oblong, pubescent, stigmas loosely connivent, 2–2.6 mm long, linear, glabrous except for a tuft of hairs at the apex. ***Torus*** concave beneath ovaries but otherwise flat, 2.0–3.7 mm in diameter. ***Fruit*** of up to 8 glabrate monocarps borne on a pedicel 5.5–10 mm long, 1.9–2.5 mm thick, pubescent to glabrate; torus 3.3–5 mm in diameter, 2–3.0 mm high, staminal cone often persistent on it, depressed-globose. ***Monocarps*** with pale green to purple-red exterior and green endocarp *in vivo*, 1.2–3.8 cm long, 1.0–1.2 cm wide, 0.8–1.1 cm thick, oblong, irregularly torulose, apex rounded or obliquely truncate, occasionally with a short beak ca. 1 mm long, base contracted into a stipe 3.5–9 mm long, 2–3 mm thick, slightly obliquely wrinkled, finely verrucose, occasionally pruinose; pericarp 0.2–0.3 mm thick. ***Seeds*** 1–6 per monocarp, in a single row or two irregular rows, oblique to long axis, 8.5–11 mm long, 6.0–8.3 mm wide, 5.1–6.5 mm thick, ellipsoid, broadly elliptic in cross-section, brown, slightly wrinkled, dull, micropylar end truncate, chalazal end rounded, raphe/antiraphe not evident, micropylar scar ca. 2.5 mm long, elliptic; sarcotesta deep orange *in vivo*; aril absent.

#### Phenology.

Specimens with flowers have been collected from October to February, and with fruits in October and from December to February.

#### Distribution

(Fig. 26). Occurs from southern Democratic Republic of the Congo east to western Tanzania and south to eastern Zambia and Malawi in open woodland, often dominated by *Brachystegia* spp. and often on sand, at elevations of 470–1770 m.

#### Local names.

Akiziga (Kilungu, *Bredo 6276*), chikiza or kikiza (Chimambwe (Musa) and Chilungu), *Michelmore 1045*), kizika (Kikungu, *Bredo 6232*), mshenene (Kinyamezi, *Wigg F.H. 1121*), msyenene (Kinyamwezi, *Shabani for Bullock 10*), mtuzya (Kinyamwezi, *Mwiga et al. 174*).

#### Additional specimens examined.

**DEMOCRATIC REPUBLIC OF THE CONGO.** Haut-Katanga: Kisanga [near Lubumbashi], 1200 m, 20 Jan 1984 (fl), *Malaisse 13134* (MO, WAG); prés de Mulwe forêt Katanguaise, 7 Jan 1949 (fl), *de Witte 5144* (K, P). **TANZANIA.** Iringa: Iringa District, 44 km on Mafinga-Madabira road, 08°14'S, 34°58'E, 1300 m, 26 Mar 2006 (fl), *Bidgood et al. 5122* (K, MO).—Kigoma: Bangwe, Kigoma District, Western Province, T. T., 2450 ft, Nov 1956 (buds), *Procter 558* (NHT).—Mbeya: T7, Mbeya District, above Chimala Mission, 8°50'S, 34°01'E, 1200 m, 23 Mar 1988 (fl), *Bidgood et al. 642* (K, NHT, WAG); Madibira, 1200 m, 28 Jan 2001 (fr), *Congdon 588* (K).—Shinyanga: Kahama, 18 mi along Ushirombo R., 1 Jan 1936 (fl), *Burtt 5459* (BM, K).—Tabora: Tabora, 28 May 1970, *Collinson U.9* (EA); Tabora, s. d., *Hammerstein 5838* (EA, K); S of Kuliwa—Tabora Region, 5°30'S, 32°40'E, 15 Nov 1979 (fl), *Lawton 2178* (K, P); S Tabora, *Lindeman 440* (K); Sikonge South Tabora, 29 Sep 1940 (yg fr), *Lindeman 788* (K); Ulyakulu Forest Reserve, Tabora, *Manolo 183* (K); Tabora, Tabora Rural District, Mtakuja Village, Urumwa Forest Reserve, 4°53'29"S, 32°45'25"E, 1040 m, 6 Jul 1999 (fl, yr fr), *Mwiga et al. 174* (MO); collected from the Tabora Beekeeping Institute Plantation, 28 Nov 1976 (buds), *Shabani 1131* (K, WAG); without definite locality [probably Tabora], *Wigg 523* (MO, US); Simbo Reserve, Tabora, June 1938 (st), *Wigg F.H.1121* (K). **ZAMBIA.** Eastern: Ndundu Hill, 1770 m, 5 Oct 1966 (fl), *Richards 31484* (K); Chadiza, 850 m, 29 Nov 1958 (fr), *Robson 770* (BM, K).—Luapula: Western Province, Fort Rosebery District, near Lake Bangweulu, Samfya Mission, 24 Aug 1952 (fr), *Angus 304* (BM, K, MO); Western Province, Fort Rosebery District, near Lake Bangweulu, Samfya Mission, 28 Aug 1952 (old fls), *White 3156* (K).—Northern: Abercorn, 10 Nov 1948 (st), *Bredo 6232* (BR); Abercorn District, Ulungu County, Mchinda village, 13 Nov 1948 (fr), *Bredo 6276* (BR); Kawimbe, 11 Nov 1949 (fl), *Bullock 1414A* (K, P); Mutinondo Wilderness, Mpika, 1500 m, 24 Dec 2005 (fr), *Congdon 709* (K); Abercorn District, 4500’, Feb 1932 (fl), *Gamwell 106* (BM); NE of Abercorn, 11 Dec 1934 (fl), *Michelmore 1045* (EA, K); Abercorn, 1933, *Miller 133D* (A, BM, K); Abercorn District, Chilongowelo, in open ground close to Plain of Death, 4800 ft, 29 Sep 1954 (fl), *Richards 1881* (K); Abercorn District, close to Lucheche stream by road to Abercorn Club turning, 5000 ft., 23 Feb 1954 (fl), *Richards 2326* (K); Abercorn District, Kawimbe, 1680 m, 2 Jan 1956 (fl, fr), *Richards 6833* (K); Abercorn District, Kawimbe, 1740 m, 20 Sep 1960 (buds, fr), *Richards 13266* (K); Uninji Pond, Mbala, 5000 ft, 3 Nov 1968 (fl), *Sanane 346* (B, K); Northern Province, Abercorn District, near top of Sunzu Hill, 18 Nov 1952 (fl), *White 3711* (K, MO). **MALAWI.** Machinga District, Chikala Mt. Liwonde Forest Reserve, Grid Ref. 36LYU6927, 1460 m, 30 Feb 1982 (fl), *Patel 977* (K); Machinga District, Chikala Mt. Liwonde Forest Reserve, Grid Ref. 36LYU6927, 1460 m, 5 Dec 1982 (fl), *Patel & Tawakali* 1028 (K); N Region, Karonga District, Vinthukutu Forest Reserve, 10 Jan 1990 (fl), *Patel & Usi 4605* (PRE); N. Prov., Nkhata Bay District, Nkhata Bay, Chikale Beach, 1550 ft., 5 Dec 1976 (fl), *Pawek 11992* (MO, WAG; N. Prov., Chitipa District, Kaseye Mission, 10 mi E of Chitipa, 1270 m, 26 Dec 1977 (fl, fr), *Pawek 13379* (K, MO, WAG); N. Prov., Mzimba District, 5 mi. NE of Mzambazi, 22 Jan 1978 (fr), *Pawek 13659* (K, MO, WAG); N. Prov., Mzimba District, 5 mi. NE of Mzambazi, 22 Jan 1978 (fl), *Pawek 13660* (MO); Nkata District, Chinteche nr. Sand Dunes, L. Nyasa, 570 m, 21 Feb 1961 (fl), *Richards 14438* (K); Central Province, Senga Bay Hotel, nr. Salima, 470 m, 16 Feb 1959 (fl), *Robson & Steele 1628* (BM, K, PRE).

*Xylopiashirensis* includes specimens formerly identified as *Xylopiaodoratissima* in Boutique (1951b), Robson (1960), and Verdcourt (1971b), but it differs consistently from that species in the somewhat longer and wider leaves, up to 10.2 cm long and 5.1 cm wide versus 6.9 cm long and 3.3 cm wide, more variable size and shape of the leaves, the smaller flowers that are grouped into inflorescences of 1–6 flowers, flower buds less bulbous at the base, and petals marked with red or purple on the inner base. The two species are not known to overlap in distribution, although both occur frequently in *Brachystegia* woodland.

The label of *Congdon 588* from western Tanzania notes that the plant is a food plant for the larvae of the *Abantis* butterfly; Cock and Congdon (2011) report that this is one of a number of food plants from a variety of families that are used by *Abantisparadisea* (Butler). The Congdon specimen also includes a photograph of the dehisced monocarp, showing the green endocarp and orange sarcotesta that characterize the members of the *X.odoratissima* subgroup. Several specimens show an oblong gall-like structure developing within the androecium of the flower.

### 22. Xylopia tomentosa

Taxon classificationPlantaeMagnolialesAnnonaceae

Exell, J. Bot. 64 Suppl.: 7. 1926.

313FA965-D347-5151-B1C6-0B6D4D295EBB

Fig. 30

Xylopia odoratissima var. minor Engler, Monogr. afrik. Pflanzen-Fam. 6: 66. 1901. Type. ANGOLA. Cuando Cubango Province, “Kunene-Gebiet, Kuelleis (Maramba) auf weissem Sandboden am Waldrand unter hohen Houtboschbäumen [Grv. Kassinga n. d. Kubango], 1400 m, 5 Oct 1899, *H. Baum 224* (holotype: B!; isotypes: BM! [000511042, left hand side of sheet], COI! [00004890], K! [001089662], M! [0107924]).
Xylopia mendoncae Exell, J. Bot. 72: 280. 1934. Type. ANGOLA. Lunda Province, Vila Henrique de Carvalho, 3 Sep 1927, *L. W. Carrisso & F. de A. Mendonça 506* (holotype: BM! [000511043, photos at GH, NY]; isotypes: COI! [00004889, ? as “*506A*”, FI-T! M! [0107920, ? as “*506A*”], MO! [1599594]).

#### Type.

ANGOLA. Cuando Cubango Province, in the mosacollas up River Bunja, Cuito, 5 Feb 1906, *J. Gossweiler 3564* (holotype: BM! [000511045, specimen in lower right of sheet]; COI! [00004893, photos at GH, NY], probable isotype [see below]: K!).

#### Description.

***Shrub or sub-shrub*** 0.4–4 m tall. ***Twigs*** dark brown to gray, longitudinally striate, tomentellous, the hairs 0.2–0.6 mm long, eventually brown to light gray, glabrate, occasionally with bark exfoliating; nodes with 2–3 axillary branches. ***Leaf*** with larger blades 2.5–6.6 cm long, 1.3–3.8 (–4.2) cm wide, subcoriaceous or chartaceous, discolorous, often purplish adaxially and yellow-tan abaxially, elliptic, oblong, or oblong-lanceolate, less frequently lanceolate or ovate, apex rounded or obtuse, base broadly cuneate, truncate, or subcordate, sparsely pubescent on both surfaces, at length glabrate adaxially but persistently pubescent abaxially; midrib plane to slightly raised adaxially, raised abaxially, secondary veins indistinctly brochidodromous, 6–12 per side, diverging at 45–80° from the midrib, these and higher order veins usually forming a raised reticulum on both surfaces but sometimes indistinct; petiole 1.5–5.5 mm long, shallowly canaliculate, tomentellous. ***Inflorescences*** axillary, 1–8-flowered, densely pubescent; peduncles 1 per axil, 1 mm long, or absent; pedicels up to 3 per peduncle, 0.4–2.3 (–5.5) mm long, 0.7–0.9 mm thick; bracts 2–3, congested on pedicel, persistent or caducous, 2.2–2.9 mm long, ovate to broadly ovate, often bilobed, apex obtuse; buds lanceolate, apex obtuse. ***Sepals*** spreading at anthesis, 1/5–1/3-connate, 1.8–3.0 mm long, 2.0–3.7 mm wide, coriaceous, ovate to broadly ovate, apex acute to obtuse, pubescent abaxially. ***Petals*** cream-colored to light yellow with brown pubescence *in vivo*; outer petals erect at anthesis, 7.4–12.9 (–18.5) mm long, 2.2–2.9 (–3.4) mm wide at base, 1.3–2.0 mm wide at midpoint, fleshy, lanceolate, apex acute, densely puberulent on both surfaces except for a glabrous patch at base adaxially; inner petals geniculate at anthesis, with the apices bent sharply outward between the outer petals, 5.1–8.9 (–12.5) mm long, 1.4–2.6 mm wide at base, 0.6–1.0 mm wide at midpoint, fleshy, lanceolate, apex long-acuminate, base with undifferentiated margin, puberulent except for glabrous base adaxially, puberulent on narrowed apex and medial portion of base abaxially, otherwise glabrous. ***Stamens*** ca. 100; fertile stamens 1–1.3 mm long, narrowly oblong or clavate, apex of connective 0.2–0.3 mm long, shieldlike or slightly hemispheric, overhanging the anther thecae, papillate, anthers 10–14-locellate, filament 0.3–0.4 mm long; outer staminodes 1.0–1.4 mm long, broadly clavate, apex rounded to truncate; inner staminodes ca. 0.9 mm long, clavate, apex truncate; staminal cone 1.0–1.4 mm in diameter, ca. 0.7 mm high, concealing only the bases of the ovaries, rim laciniate. ***Carpels*** 5–6; ovaries 0.9–1.4 mm long, oblong, pubescent, stigmas connivent, 1.4–2.4 mm long, linear, setose toward apex. ***Torus*** flat, 1.3–1.8 mm in diameter. ***Fruit*** of up to 11 brown-tomentose monocarps borne on a pedicel 4–8 mm long, 1.2–3.2 mm thick, pubescent to glabrate, with bracts and sepals sometimes persistent; torus 2.5–6.8 mm in diameter, 2.9–6.3 mm high, globose. ***Monocarps*** with green exterior and red to pink endocarp *in vivo*, 1.6–3.3 cm long, 0.9–1.3 cm wide, 0.8–1.4 cm thick, oblong, obliquely torulose, apex obtuse, occasionally with a broad oblique beak ca. 0.4 mm long, base contracted into a stipe 2–8 mm long, 1.8–2.8 mm thick, obliquely wrinkled; pericarp 0.4–0.6 mm thick. ***Seeds*** 1–3 per monocarp, in a single row, oblique to long axis, 6.8–9.4 mm long, 4.9–7 mm wide, 4–6 mm thick, ellipsoid to ellipsoid-pyriform and then narrowed into a cylindrical neck 1.5–2 mm long and 2.2–4.6 mm wide toward micropyle, elliptic in cross section, truncate at micropylar end, rounded at chalazal end, brown, smooth, glossy, raphe/antiraphe not evident, micropylar scar 1.3–1.8 mm long, 1.0–1.2 mm wide, elliptic to ovate; sarcotesta orange to red, fleshy *in vivo*; aril absent.

**Figure 30.**
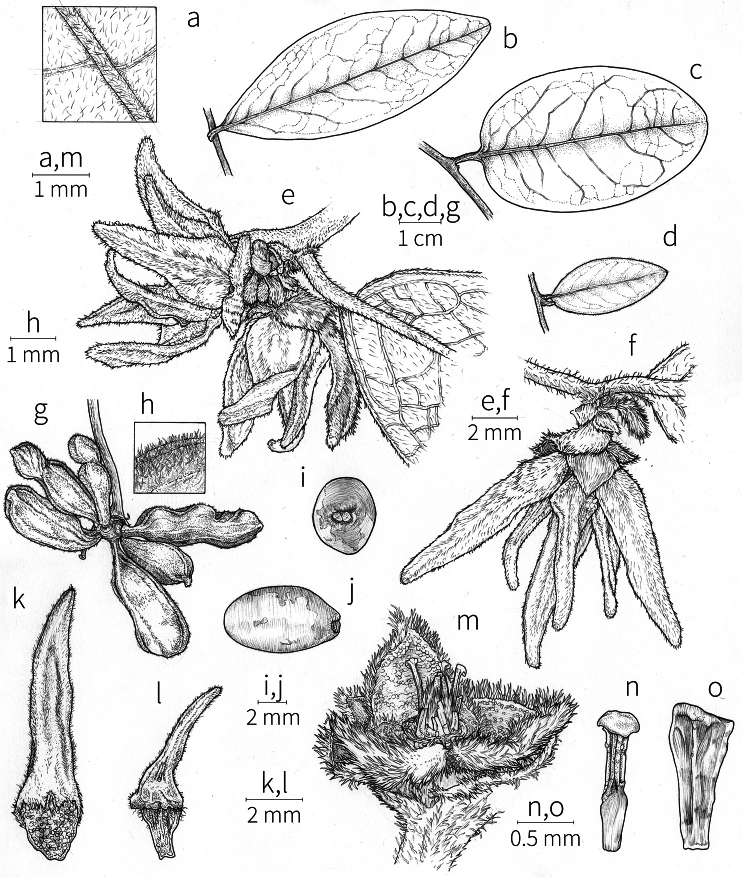
*Xylopiatomentosa*. **A** Close-up of abaxial surface of leaf **B–D** Leaves to show range of variation **E, F** Inflorescences **G** Fruit **H** Close-up of monocarp surface, showing pubescence **I** Seed, micropylar view **J** Seed, side view **K** Outer petal, adaxial view **L** Inner petal, adaxial view **M** Receptacle, with sepals, some stamens, and ovaries still attached **N** Stamen, abaxial view **O** Outer staminode, abaxial view. **A, D** based on *Brummitt et al. 14111* (K) **B, G, H** from *Exell & Mendonça 374* (BM) **C** from *Brummitt et al. 14068* (MO) **E** from *Milne-Redhead 2528* (K) **F, K, L, N, O** from *Holmes 1056* (K) **I, J** from *Brummitt et al. 14068* (K) **M** from *Pocock 445* (A).

#### Phenology.

Specimens with flowers have been collected from December to June and in September and October, and with fruits in every month of the year except September.

#### Distribution

(Fig. 22). Occurs from southern and southwestern Democratic Republic of Congo east to northeastern Zambia and south to northeastern Namibia and southwestern Zambia. *Xylopiatomentosa* grows in thickets or sub-xerophilic forest on sandy or otherwise siliceous soils with species of *Cryptosepalum* (mavunda), *Copaifera*, *Psorospermum*, *Baphia*, *Brachystegia* (mucuwe), *Burkea*, *Uapaca*, and *Swartzia*, at elevations of 1000–2000 m.

#### Local names.

Changue (Wemba, *Brenan & Greenway 8041*), itundubywangu (*Rea 119*), mujimbajimba (Chiokwe, *Exell & Mendonça 1310*), mulamo (Chikaonde, *Milne-Redhead 951*), mundzeemba dzeemba (*Pocock 445*), situndu bawanga (Lozi, *White 2071, 2079*).

#### Additional specimens examined.

**DEMOCRATIC REPUBLIC OF THE CONGO.** Haut-Katanga: 120 km S de Mukulakulu (gare près de Luéna) [9°33'S, 25°47'E], sur la route vers Kanzenze et Kolwezi, 5 Nov 1958 (fr), *Schmitz 6188* (BR).—Kwango: environs de Kitenda [6.8°S, 17.35°E], 15 Feb 1950 (fr), *Callens 2440* (BM); Prov. Leopoldville, Kasongo Lunda, Kibunda [7°29'S, 18°12'E], 4 Feb 1952 (bud, fr), *Callens 3056* (BM, K); Kimbuba [5°45'S, 15°49'E], 28 Dec 1952 (fr), *Callens 3850* (EA); Kimbuba, 29 Dec 1952 (fl), *Callens 3859* (B); Pansi, Aug 1950 (fl), *Callens 2730* (BM); Prov. Léopoldville, Territ. Kasongo-Lunda, Kibunda, 26 Apr 1953 (fr), *Callens 3983* (BM, WAG); Mikondo, Kahemba, 11 Mar 1954 (buds), *Devred 1420* (K); Prov. Leopoldville, Territ Kahemba, Kahemba-Kwango, 28 Apr 1955 (fr), *Devred 1824* (K); de Mto Mbombo á Kinlongo, Terr. Popokabaka, 9 Jan 1959 (st), *Pauwels 1242* (BR); Kimbuba, Terr. Popokabaka, 12 May 1959 (fr), *Pauwels 2961* (BR); Kimbuba, Terr. Popokabaka, 12 May 1959 (st), *Pauwels 2972* (BR). **ANGOLA.** Benguela: Cubal, entre Caimbambo e o Cubal, a 8 kms de Caimbambo, 20 Jul 1969 (buds), *Barbosa 11754* (MO).—Bié: Munhango, 27 Jan 1940 (fl, fr), *Andrada 51* (MO, WAG); Munhango, Mar 1943 (fr), *Andrada 51A* (MO).—Cuando Cubango: Environ à 10 km à l’Est de Longa, 14°49'S, 18°38'E, *Dechamps et al. 1324* (YF), 2 Mar 1974 (fl), *Dechamps et al. 1325* (K, WAG, YF), 2 Mar 1974 (fl), *Dechamps et al. 1330* (MO, YF); Kubango, 2 Oct 1905 (bud, fr), *Gossweiler 2054* (BM, K); Kubango region, between the rivers Cdui and Tunga, 15 Oct 1905 (fr), *Gossweiler 2102* (BM); between Cuiriri and Cuito, 1906 (fl), *Gossweiler 2562* (BM, K); at T’chirandangambe river Luassingua-Ganguellas, s. d. (fr), *Gossweiler 3463* (BM [on same sheet with *Gossweiler 3564*], K); between rivers Kuito and Kuiriri, Jan 1906 (fr), *Gossweiler 3464* (BM); nr. Cassuango, Cuiriri, Jan 1906 (fl), *Gossweiler 3465* (BM—2 sheets [one on same sheet as *Baum 224*, other on same sheet as *Gossweiler 3567*], K); Bunja nascente Cuito, 5 Feb 1906 (fl, fr), *Gossweiler 3567* (BM); at Kassuango near Kuiriri, 24 Mar 1906 (fl), *Gossweiler 3672* (BM, K); Kassuango Kuiriri, Mar 1906 (fl), *Gossweiler 3733* (BM, K); in the valley of River Kuiriri, 1906 (fr), *Gossweiler 4044* (BM); R. Kutri, also at R. Kunzambia, 27 Jun 1925 (fl, fr), *Pocock 445* (A).—Huambo: between Bimbe and Sanga, near Bimbe [15°12'S, 17°41'E], 1300–1700 m, 24 Jun 1937 (fl), *Exell & Mendonça 3063* (BM, MO); Benguella, country of the Ganguellas and the Ambuellas, rec’d 1910, *Gossweiler s. n.* (K).—Huíla: Between Sá da Bandeira and Humpata, 16 May 1937 (fl), *Exell & Mendonça 2030* (BM, M, MO).—Lunda: Xá-Sengue [10°27'S, 20°13'E], 7 Apr 1937 (fl), *Exell & Mendonça 370* (BM, M, MO), Xa-Sengue, ca. 1200 m, 7 Apr 1937 (fr), *Exell & Mendonça 374* (BM, MO); R. Luachimo, between Vila Henrique de Carvalho and Dala, ca. 1300 m (fl), 24 Apr 1937 (fl), *Exell & Mendonça 1024* (BM); entre Vila Henrique de Carvalho e Dala, 24 Apr 1937 (fl, fr), *Exell & Mendonça 1055* (BM, M, MO); between Vila Henrique de Carvalho and Dala, 1100–1300 m, 24 Apr 1937 (fr), *Exell & Mendonça 1064* (BM); Dala, 1200–1230 m, 23 Apr 1937 (fl, fr), *Exell & Mendonça 1101* (BM, M, MO), Dala, 1200–1230 m, 25 Apr 1937 (fr), *Exell & Mendonça 1102* (BM, M); Biula [11°11'S, 20°13'E], R. Chicoso, c. 1200 m, 27 Apr 1937 (fr), *Exell & Mendonça 1310* (BM); Dala, ca. 1230 m, 29 Apr 1937 (fr), *Exell & Mendonça 1430* (BM, MO); Dala, ca. 1230 m, 29 Apr 1937 (fr), *Exell & Mendonça 1431* (BM); Dala, próx. do rio Chimbe, Apr 1937 (fl, fr), *Gossweiler 11202* (BM, M, MO); Mona Quimbundo, proximum flumen Luvo, 1000 m, Apr 1937 (fl), *Gossweiler 11833* (BM); Missão de Luz, 29 Dec 1933 (fl), *Lynes 351a* (BM); Ma-chinga and Ma-Lunda, Jun 1885 (fr), *Marques 165* (BM); Saurimo-Dala, 1 Nov 1932 (fl), *Young 1306* (A, BM).—Moxico: Between Vila Luso and R. Cassai, 4 May 1937 (fl), *Exell & Mendonça 1632* (BM); entre Cachipaque e Munhango, 7 May 1937 (fl), *Exell & Mendonça 1790* (BM, M, MO); Teixeira de Souza, 9 Jul 1940 (fl), *Gossweiler 12340* (BM). **NAMIBIA.** Bei Bikundu, 19 km südlich Andara, 12 Jun 1971 (fl), *Giess 11354* (M—2 sheets, WAG—2 sheets); Okavango Native Territory, border clearance 1 m W of Katwitwi [17°24'S, 18°25'E], *de Winter 3845* (K, M); Okavango Native Territory, 3 mi. S of Omuramba Mpungu [17.8°S, 18.9°E] on rd. to Tsinaabia, *de Winter 3879* (K, M). **ZAMBIA.** Luapula: Fort Rosebery, on low fixed dunes by shore of L. Bangweolo, a short way N of Samfya Mission, 6 Oct 1947 (fl), *Brenan & Greenway 8041* (BM, EA, K—2 sheets), 6 Oct 1947 (fr), *Brenan & Greenway 8044* (EA, K).—Northwestern: Mwinilunga District, near Zambezi R. 4 mi N of Kalene Hill Mission, 23 Sep 1952 (fl), *Angus 529* (K, MO); Solwezi District, proposed transmission line clearing near Kabompo R. crossing point, Chipawa, 12°13'S, 25°11'12"E, 1154 m, 30 Apr 2012 (fl), *Bingham 14167* (K); Mwinilunga District, 28 km on Matonchi road, 11°44'S, 24°14'E, 24 Jan 1975 (fr), *Brummitt et al. 14068* (K, MO); Mwinilunga District, 60 km S of Mwinilunga on road to Kabompo, *Brummitt et al. 14111* (K, WAG); Samfya, 3 Oct 1953 (fr), *Fanshawe F.390* (K); Balovale [Zambezi], Lukolwe [13°33'S, 23°07'E], 9 Aug 1952 (fr), *Gilges 156* (K); Balovale District, about 10 miles S of Chavuma [13°04'S, 22°41'E], 1–10 Feb 1953 (fl), *Holmes 1056* (K); Mwinilunga District, between Mukimina and Kahuku, 24 Aug 1930 (fr), *Milne-Redhead 951* (K); Mwinilunga District, just N of Mwinilunga, 2 Oct 1937 (fl), *Milne-Redhead 2528* (B, K–2 sheets); Mwinilunga District, just S of Matonchi farm, 30 Dec 1937 (fr), *Milne-Redhead 3887* (K); Zambia, K. Gorge, Solwezi, 15 May 1969 (fl), *Mutimushi JMM 3325* (K); Kabompo District, Manyinga, ca. 30 km from Loloma Hospital junction along the Kabompo-Mwinilunga Road, 13°17'47"S, 24°15'04"E, 1140 m, 9 Mar 1995 (fl), *Nawa et al. 213* (MO); Mwinilunga District, 10 mi W of Kakoma, 30 Sep 1952 (fl), *White 3436* (K).—Western: Mongu District, near Mongu, 21 Jul 1961 (fr), *Angus 3010* (K, NY); Namushakende-Nalikwanda road, 24 km, 15°26'45"S, 23°25'48"E, 1000 m, 5 Jul 1997 (fl), *Bingham et al. 11492* (K); Kaoma District, ca. 5 km along road to Luampa Hospital from intersection with Lusaka-Mongu Roads, 14°59'05"S, 24°32'14"E, 1080 m, 2 Mar 1996 (fl), *Harder et al. 3644* (MO); Barotse Province, Senanga District, 18 Jun 1950 (fl), *Rea 119* (K); Barotseland, Mongu District, 44 mi from Mongu along new main road east [15°25'S, 23°17'E], 21 Oct 1972 (fr), *Strid 2370* (MO); Barotse Province, Kalabo District, Sikongo Forest Reserve, Kalabo to Sikongo, mile 7, 14 Feb 1952 (fl), *White 2071* (K); Barotse Province, Kalabo District, Sikongo Forest Reserve, 15 Feb 1952 (st), *White 2079* (K); Kaoma District, 51.3 km NW along Kaoma-Lukulu Road, from junction of Kaoma-Mungu Road, 14°35'40.9"S, 24°20'03.6"E, 1080 m, 1 Mar 1996 (fr), *Zimba et al. 739* (MO).—Province unknown: S. N. W. Rhodesia, Baikiaea forest Region, 1937 (fl), *Martin JDM 762* (BM).

By its shrubby habit, densely tomentose leaves, and congested inflorescences of up to eight small flowers, *Xylopiatomentosa* is readily distinguished from *X.odoratissima*, which may occur in some of the same habitats over its distribution. Several specimens (*Baum 224*, *Exell & Mendonça 1790*, *Gossweiler 11202*) have petals longer than is typical for *X.tomentosa*, the outer petals up to 18.5 mm long in the case of *Gossweiler 11202*, approximating the petal length of *Xylopiaodoratissima*, but the pedicels are shorter and the outer petals taper gradually so that the width at the midpoint is greater (ca. 3.4 mm) than is found in *X.odoratissima* (1.0–1.7 mm wide). However, the sharply geniculate inner petals and pink endocarp of the fruits of *X.tomentosa* are more similar to *X.arenaria*, *X.collina*, and *X.keniensis*.

*Xylopiatomentosa* is variable in the number of flowers per inflorescence and size and shape of the leaves. Specimens with only one flower per leaf axil, for example *Callens 3859* from the Democratic Republic of the Congo, can look very different from those with inflorescences with large numbers of flowers, for example the type specimen of *Xylopiamendoncae*, but all possible intermediates between these two extremes occur. Variation in leaf shape includes specimens with narrowly oblong leaves, such as the type of Xylopiaodoratissimavar.minor (*Baum 224*) and *Dechamps et al. 1325*, and nearly orbicular leaves (*Gossweiler 3563*). The label of one specimen described the plant as re-sprouting after fire, and it is possible that the variability in leaf morphology represents growth plasticity of regenerating vegetative shoots.

Numerous collectors remarked on the sweet-scented flowers, but nothing is known of flower visitors or floral biology.

A specimen of *X.tomentosa* at K is numbered *Gossweiler 3563*, but was annotated by A. W. Exell in 1926 with the comment “*Gossweiler 3563* in Herb. Brit. Mus. is *Vernoniapotamophila* Klatt, while our specimens of *X.tomentosa* are *3463* and *3564*.” The K sheet contains a specimen with flowers and leaves similar in appearance to those on the *Xylopiatomentosa* holotype specimen *Gossweiler 3564* at BM, and it seems probable that the K sheet is in fact an isotype of this name.

### 23. Xylopia torrei

Taxon classificationPlantaeMagnolialesAnnonaceae

N. Robson, Bol. Soc. Brot., Sér. 2, 32: 157–158. 1958.

3A0AF83A-09B3-5901-ABB7-1DEB8423DF30

Fig. 25M–T

#### Type.

MOZAMBIQUE. Gaza [“Sul do Save”] Province, Chibuto, 11 Dec 1940, *A. R. Torre 2350* (holotype: LISC! [000361]; isotypes: K! [000199051], LMA—photo!).

#### Description.

***Shrub*** 2–3 m tall with spreading branches. ***Twigs*** brown to reddish brown, sparsely pubescent, the hairs 0.4–0.6 mm long, becoming light brown to light gray, glabrate, with the bark exfoliating; nodes occasionally with two axillary branches. ***Leaf*** with larger blades 3.8–5.5 cm long, 1.8–2.5 cm wide, chartaceous, concolorous, elliptic, apex obtuse or rounded, rarely emarginate, base broadly cuneate to rounded, glabrous or with a few scattered hairs adaxially, sparsely pubescent abaxially; midrib plane adaxially, raised abaxially, secondary veins weakly brochidodromous, 7–9 per side, diverging at 50–60° from the midrib, these and higher-order veins slightly raised on both surfaces; petiole 3–3.5 mm long, shallowly canaliculate, pubescent. ***Inflorescences*** axillary, 1–3-flowered, pubescent; pedicels arising separately from axil, 4.0–5.4 mm long, 0.5–0.9 mm thick; bracts 2, both attached near pedicel midpoint, caducous, 0.9–1.3 mm long, semicircular to ovate, apex obtuse to rounded; buds linear, apex acute. Sepals spreading at anthesis, 1/4–1/2-connate, 1.8–2.2 mm long, 2.1–2.3 mm wide, chartaceous to coriaceous, broadly ovate to semicircular, apex acute to apiculate, pubescent abaxially. ***Petals*** yellow-green *in vivo*; outer petals loosely spreading at anthesis, 20–30.4 mm long, 2.6–3.1 mm wide at base, 1.2–1.7 mm wide at midpoint, chartaceous, linear, apex acute to obtuse, densely puberulent except for glabrous base adaxially, appressed-pubescent abaxially, faintly ridged or flat abaxially; inner petals curved outward to weakly geniculate at anthesis, 23–28 mm long, 2.0–2.2 mm wide at base, 0.6–0.7 mm wide at midpoint, chartaceous, linear-filiform, apex acute, base with undifferentiated margins, longitudinally ridged on both surfaces, densely puberulent except for glabrous base adaxially, densely puberulent abaxially. ***Stamens*** ca. 100; fertile stamens 1.1–1.2 mm long, narrowly oblong, apex of anther connective red *in vivo*, shieldlike, overhanging anther thecae, anthers 9–10-locellate, filament 0.3–0.4 mm long; outer staminodes 1.1–1.3 mm long, clavate to broadly clavate, apex rounded to truncate; inner staminodes ca. 1.0 mm long, oblong-clavate, apex truncate; staminal cone ca. 1.4 mm in diameter, ca. 0.9 mm high, concealing only the bases of the ovaries, rim laciniate. ***Carpels*** ca. 7; ovaries 0.8–0.9 mm long, ovoid to oblong, pubescent; stigmas connivent, ca. 2.7 mm long, linear, glabrous except for an apical tuft of hairs. ***Torus*** flat, 1.6–2.2 mm in diameter. ***Fruit*** of up to 8 glabrate monocarps borne on a pedicel ca. 6.5 mm long, ca. 3.9 mm thick, glabrate; torus of fruit ca. 6.7 mm in diameter, ca. 6.3 mm high, depressed-globose. ***Monocarps*** green with a light green endocarp *in vivo*, 2.8–3.1 cm long, 1.0–1.1 cm wide, ca. 0.9 cm thick, oblong, irregularly torulose, apex truncate with an oblique beak 0.5–1 mm long, base contracted into a stipe 4.5–6 mm long, 1.8–2.7 mm thick, obliquely wrinkled, minutely verrucose; pericarp 0.2–0.5 mm thick. ***Seeds*** up to 4 per monocarp, in a single row, lying oblique to long axis, 10.5–11.5 mm long, ca. 7.5 mm wide, 6.1–6.7 mm thick, ellipsoid, flattened-elliptic in cross-section, obliquely truncate at micropylar end, rounded at chalazal end, brown, smooth, shiny, raphe/antiraphe not evident, micropylar scar 1.7–2.4 mm long, 1.1–1.4 mm wide, ovate to elliptic; sarcotesta red *in vivo*; aril absent.

#### Phenology.

Specimens with flowers have been collected in February and December, and with fruits in December.

#### Distribution

(Fig. 26). Endemic to southern Mozambique, on sandy sites near the coast at elevations from sea level up to 60 m.

#### Additional specimens examined.

**MOZAMBIQUE.** Gaza [“Sul do Save”]: Chibuto, 11 Feb 1942 (fl), *Torre 3944* (LMA—2 sheets, photos).—Inhambane [“Sul do Save”]: Panda, 25 Feb 1955 (fl), *Exell et al. 598* (BM).—Maputo: Licuati Forest Reserve ca. 20 km W of Bela Vista, 26°20.667'S, 32°28.387'E, 60 m, 8 Dec 2001 (fl, fr), *Goyder et al. 5037* (K); Licuati, sand forest, 25°26'S, 32°30'E, 60 m, 5 Dec 2002 (fr), *Lötter s. n.* (LYD—photo).

*Xylopiatorrei* superficially resembles *X.odoratissima* in its small leaves, long petals, and shrub habit. It may be distinguished by the straighter and sparser hairs of the twigs, the smaller sepals, and the outer petals narrower at the base. The tendency for exfoliation of the bark on the twigs may also be diagnostic. The species is only known from a small area in the southernmost part of Mozambique, where it appears to be restricted to coastal sand forest at low elevations, and has the southernmost distribution of any African *Xylopia* species, remote from the distribution of any other species. We calculated for it an EOO of 7,053 km^2^ and an AOO of 12 km^2^.

### 24. Xylopia toussaintii

Taxon classificationPlantaeMagnolialesAnnonaceae

Boutique, Bull. Jard. Bot. État 21: 111–112. 1951.

3E6F9375-AB4E-5E6E-B55E-6EB401409752

#### Type.

DEMOCRATIC REPUBLIC OF THE CONGO [“Belgian Congo”]. Kongo Central Province, Mayumbe, Luki, vallé de la N’kula, May 1948, *L. Toussaint 373* (holotype: BR!; isotypes: BM! [000511058], BR! [0000008825483], K! [000199052], P! [00169123, 00169124]).

#### Description.

***Tree*** up to 30 m tall (shrub 6 m tall—*Gossweiler 9151*), d.b.h. up to 30 cm, bole up to 14 m high, secondary branches forming a broad round crown; bark finely fissured. ***Twigs*** brown, eventually gray to brown, smooth, spreading-pubescent, the hairs 0.2–0.5 mm long, eventually gray to brown, glabrate; nodes occasionally with two axillary branches. ***Leaf*** with larger blades 4.1–9.1 cm long, 1.9–2.9 cm wide, chartaceous, concolorous or slightly discolorous, oblong or lanceolate-oblong, sometimes ovate, apex acute to rounded, base rounded, densely short-pubescent on midrib but otherwise glabrate adaxially, uniformly pubescent abaxially; midrib plane adaxially, raised abaxially, secondary veins indistinctly brochidodromous, 7–11 per side, diverging at 45–70° from the midrib, slightly raised on both surfaces, higher-order veins slightly raised to indistinct on both surfaces; petiole 2–3.2 mm long, shallowly canaliculate, pubescent. ***Inflorescences*** axillary or from the axils of fallen leaves, 1–3-flowered, pubescent; peduncle 1 per axil, ca. 0.5 mm long; pedicels up to 3 per peduncle, 1.9–3.3 mm long, 0.5-0.8 mm thick; bracts 2, lower attached near pedicel midpoint and upper subtending sepals, caducous or less often persistent, 2.4–3.6 mm long, ovate to broadly ovate, apex obtuse to rounded; buds linear-lanceolate, apex acuminate. ***Sepals*** spreading or slightly reflexed at anthesis, 1/10-connate, 1.3–2.2 mm long, 1.9–2.3 mm wide, coriaceous, broadly ovate to semicircular, apex obtuse to acute, pubescent abaxially. ***Petals*** yellowish to olive *in vivo*, outer petals more or less erect at anthesis, 8.7–11.8 mm long, 2.4–2.9 mm wide at base, 0.6–1 mm wide at midpoint, fleshy, linear-lanceolate, apex more or less acute, pubescent on both surfaces except for the glabrous adaxial base; inner petals probably spreading at anthesis, 6.4–10.3 mm long, 1.9–2.2 mm wide at base, 0.5–0.8 mm wide at midpoint, fleshy, linear-subulate, apex acute, base with margin differentiated into two circular to oblong glands ca. 0.4 wide, pubescent except for the glabrous concavity adaxially, pubescent abaxially. ***Stamens*** 75–80; fertile stamens 0.9–1.0 mm long, oblong, apex of connective ca. 0.2 mm long, shieldlike, overhanging the anther thecae, glabrous, anthers 7–8-locellate, filament 0.2–0.3 mm long; outer staminodes 0.8–1.1 mm long, oblong to clavate, apex obtuse to truncate; inner staminodes 0.5-0.8 mm long, oblong to clavate, apex rounded to truncate; staminal cone 0.9–1.1 mm in diameter, 0.4–0.8 mm high, concealing only the bases of the ovaries, rim laciniate. ***Carpels*** 3–5; ovaries 0.8–1 mm long, narrowly oblong, pubescent, stigmas loosely connivent, 1.9–2.2 mm long, linear, glabrous except for an apical tuft of hairs. ***Torus*** flat, 1.0–1.3 mm in diameter. ***Fruit*** of up to 4 sparsely pubescent monocarps borne on a pedicel 5–7.3 mm long, 2.4–3.4 mm thick, sparsely pubescent; torus 4.5–5.7 mm in diameter, 2.5–3.7 mm high, depressed-globose. ***Monocarps*** with a green, sometimes red-tinged, exterior *in vivo*, endocarp color unknown, 2.8–3.9 cm long, 1–1.3 cm wide, 0.9–1.1 cm thick, oblong to clavate, irregularly torulose, apex irregularly obtuse to truncate, base contracted into a stipe 5–9 mm long, 2.3–3.6 mm thick, obliquely wrinkled, verrucose; pericarp 0.6–1.6 mm thick. ***Seeds*** up to 6 per monocarp, in a single row, oblique to long axis, 8.5–9.5 mm long, 5.0–6.5 mm wide, 3.7–4.5 mm thick, ellipsoid to flattened-ellipsoid, elliptic to wedge-shaped in cross-section, truncate at micropylar end, rounded at chalazal end, reddish brown to brown, smooth, dull or slightly shiny, raphe/antiraphe not evident, micropylar scar 1.8–2.0 mm long, 1.7–2.1 mm wide, obovate, elliptic, or circular; sarcotesta orange to red-orange *in vivo*; aril absent.

#### Phenology.

Specimens with flowers have been collected from April to June, and with fruits from May, August to October, and December.

#### Distribution

(Fig. 24). Occurs near the mouth of the Congo River in Angola, the Republic of the Congo, and the Democratic Republic of Congo in forest bordering savanna and other xerophytic forest types at low elevations.

#### Local name.

Lucangua (*Toussaint 373, 2451*).

#### Additional specimens examined.

**REPUBLIC OF THE CONGO.** Région de Pointe-Noire, Pointe-Indienne, 1 Jun 1966 (fl), *Sita 1232* (P). **DEMOCRATIC REPUBLIC OF THE CONGO.** Kongo Central: Luki, 18 Oct 1948 (fr), *Donis 2065* (K, P); Prov. Léopoldville, Territ. Boma, Luki, Mar 1959 (buds), *Mahieu 290* (K); Mayumbe, Luki, 2 Dec 1946 (fr), *Toussaint 2068* (K, MO, P, PRE); Luki, 3 Apr 1947 (fl), *Toussaint 2241* (K, MO, P); Luki, vallée de la Mikindu, 4 Sep 1947 (fr), *Toussaint 2451* (BR, K, MO, P); Prov. Leopoldville, Territ. Boma, Station a la Mbola, border of savanna and dry forest, 30 Aug 1957 (fr), *Wagemans 1696* (K). **ANGOLA.** Sumba, Peco, 1 May 1923 (fl), *Gossweiler 8741* (BM—2 sheets, K, US), Sumba, Peco, May 1926 (fl, fr), *Gossweiler 9151* (BM, MO, US).

A poorly known species similar to *Xylopiagilbertii*, *X.toussaintii* differs most notably in its longer flowers with proportionately narrower petals. *Xylopiatoussaintii* occurs in an area of strongly seasonal rainfall and corresponding semi-evergreen vegetation (Couralet 2010). We calculated an EOO of 6,766 km^2^ and an AOO of 16 km^2^ for *Xylopiatoussaintii*.

### 25. Xylopia wilwerthii

Taxon classificationPlantaeMagnolialesAnnonaceae

De Wildeman & T. Durand, Ann. Mus. Congo, Sér. 2, Bot. 1(1): 5. 1899.

B6183780-899A-5433-B2C2-A50F2722AEE8

Fig. 31

Xylopia wilwerthii var. cuneata De Wildeman in De Wildeman & T. Durand, Bull. Soc. Roy. Bot. Belg. 40(2): 63. 1901. Type. DEMOCRATIC REPUBLIC OF THE CONGO [“Congo Belge”]. Kinshasa Province, Kimuenza, May 1901, *J. Gillet s. n.* (holotype: BR!; isotype: BR! [0000008825476]).

#### Type.

DEMOCRATIC REPUBLIC OF THE CONGO [“Congo Belge”]. Mongala Province, Rég. III, Upoto, 1896, *Capt. Wilwerth s. n.* (holotype: BR!, isotype: BM! [000511002]).

#### Description.

***Tree*** up to 15 m tall, d.b.h. up to 10 cm, slender with a short-branched crown. ***Twigs*** brown, eventually brownish gray to gray, fine-pubescent, the hairs 0.1–0.2 mm long, soon glabrate; nodes occasionally with two axillary branches. ***Leaf*** with larger blades 5.1–8.9 cm long, 1.5–2.8 cm wide, chartaceous, concolorous, elliptic to elliptic-oblong, apex acuminate to caudate, the acumen 5.5–16 mm long and rounded at the tip, base cuneate and somewhat decurrent on petiole; glabrous adaxially, sparsely pubescent to glabrate, occasionally with longer seta-like hairs along midrib abaxially; midrib plane or impressed adaxially, raised abaxially, secondary veins indistinctly brochidodromous and more or less parallel, 15–22 per side, diverging at 60–80° from the midrib, these and higher-order veins slightly raised or indistinct on both surfaces; petiole 1.5–3.5 mm long, shallowly canaliculate, sparsely pubescent to glabrate. ***Inflorescences*** axillary, 1-flowered, sparsely pubescent; pedicel not pedunculate, 8.5–21.5 mm long, 0.8–1.0 mm thick, articulated at midpoint, sometimes slightly thickened just proximal to lower bract; bracts 2, the upper attached just a few mm proximal to the sepals, the lower just distal to the pedicel midpoint, more or less persistent, 0.8–1.8 (-2.6) mm long, ovate to semicircular, apex rounded or emarginate, ciliate on margins; buds linear-lanceolate, apex obtuse, slightly falciform. ***Sepals*** slightly spreading at anthesis, 1/2–2/3-connate, 2.1–3 mm long, 2.8–3.1 mm wide, coriaceous, broadly ovate, apex acute to mucronate, sparsely pubescent. ***Petals*** white, cream-colored, or yellow *in vivo*; outer petals spreading at anthesis, 19–31 mm long, 3.0–3.5 mm wide at base, 1.9–3.0 mm wide at midpoint, chartaceous, ligulate, apex obtuse, densely puberulent adaxially, sparsely pubescent except for glabrous patch at base abaxially; inner petals probably spreading at anthesis, 14.1–24.0 mm long, 2.0–3.1 mm wide at base, 1.4–1.6 mm wide at midpoint, chartaceous, linear, apex acute, base with undifferentiated margin, puberulent on both surfaces except for the glabrous base. ***Stamens*** 100–120; fertile stamens 1.1–1.5 mm long, narrowly oblong, apex of connective 0.1–0.3 mm long, shieldlike, overhanging the anther thecae, finely papillate, anthers 12–13-locellate, filament ca. 0.2 mm long; outer staminodes 1.1–1.6 mm long, oblong, apex obtuse, truncate, or emarginate; inner staminodes 1.0–1.1 mm long, oblong to quadrate, apex truncate; staminal cone 2–2.1 mm in diameter, 0.6–0.9 mm high, partially to completely concealing the ovaries, rim laciniate. ***Carpels*** 6–13; ovaries 0.8–0.9 mm long, oblong, pubescent, stigmas connivent, 2.9-3.2 mm long, linear, with a tuft of hairs at the apex. ***Torus*** flat, 2.5–3.1 mm in diameter. ***Fruit*** of up to 12 glabrate monocarps borne on a pedicel 7.5–23.5 mm long, 2–3 mm thick, glabrate; torus 4.5–7 mm in diameter, 3–5 mm high, irregularly depressed-globose. ***Monocarps*** with a green exterior and scarlet endocarp *in vivo*, 1.7–4.0 cm long, 0.7–1.2 cm wide, 0.5–1.0 cm thick, asymmetrically oblong or ellipsoid, distinctly torulose, apex rounded or with a broad curved beak 1.4–3 mm long, base tapering gradually into a stipe 3–7 mm long, 2.4–4 mm thick, obliquely or longitudinally wrinkled; pericarp 0.4–0.6 mm thick. ***Seeds*** up to 6 per monocarp, in a single row, lying parallel or oblique to long axis, 9.6–10.8 mm long, 5.2–7.1 mm wide, 4.9–7.0 mm thick, ellipsoid, elliptic to roughly circular in cross-section, rounded or obliquely truncate at micropylar end, rounded at chalazal end, chestnut brown, smooth, somewhat shining, raphe/antriraphe indistinct, flush with surface of seed, micropylar scar sunken, 1.5–2 mm in diameter, more or less circular; sarcotesta orange *in vivo*; aril absent.

**Figure 31.**
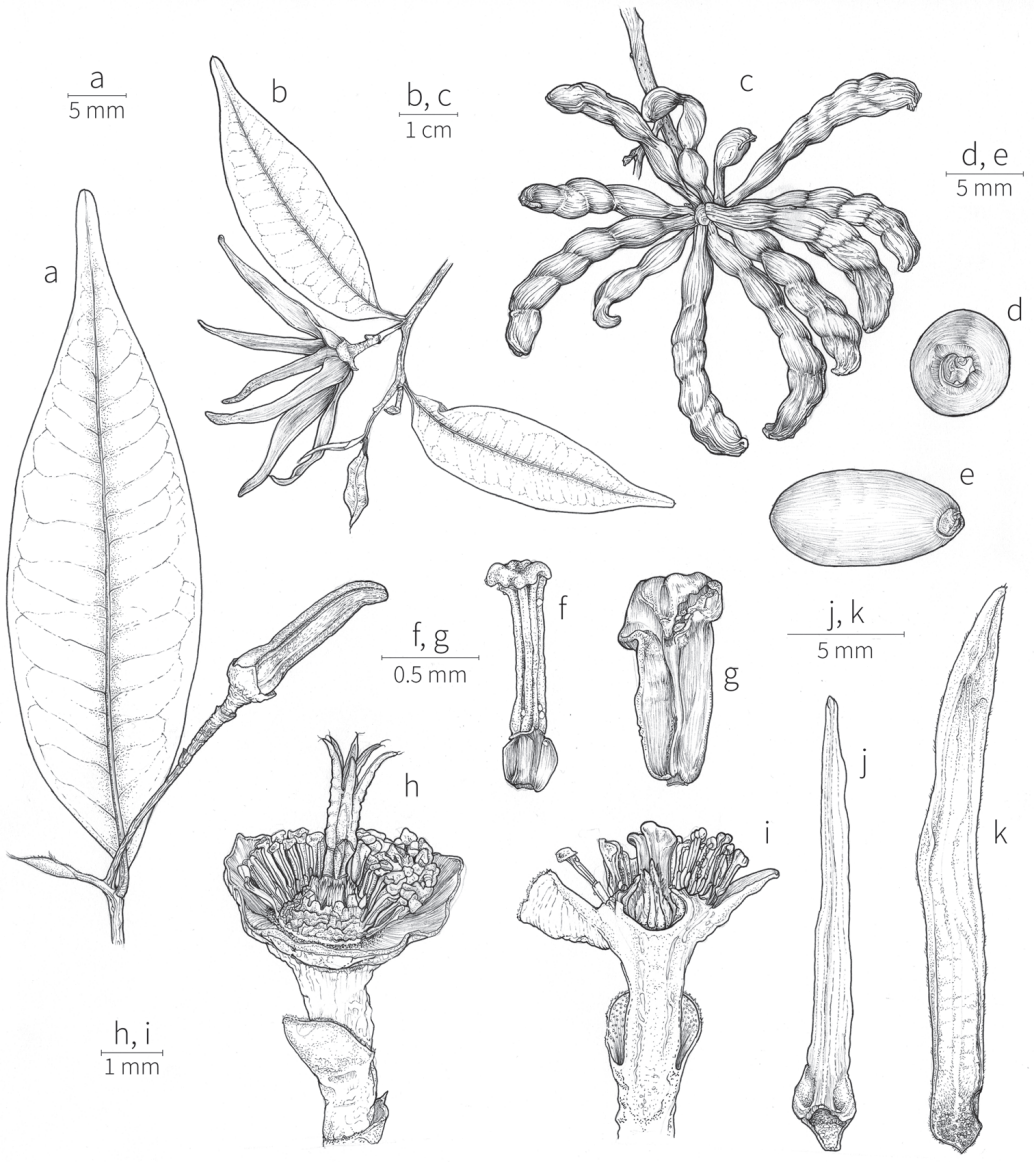
*Xylopiawilwerthii***A** Habit with nearly mature flower bud **B** Habit, with open flower **C** Fruit, side view **D** Seed, view of micropylar end **E** Seed, side view **F** Fertile stamen, abaxial view **G** Staminode, abaxial view **H** Flower with petals and some stamens removed, side view **I** Longitudinal section of flower, with petals removed, showing ovaries surrounded by staminal cone **J** Inner petal, adaxial view **K** Outer petal, adaxial view. **A, C** from *Carrington 7* (K) **B, F–K** from *Donis 2041* (BR) **D, E** from *Trochain 9603* (P).

#### Phenology.

Specimens with flowers have been collected in March, from May to July, and in September and October, and with fruits from January to March, and in June and October. A ten-year phenology dataset from 1948–1957 from the Luki Reserve in southwestern Democratic Republic of Congo was analyzed by Couralet (2010), who found that *X.wilwerthii* in the Reserve consistently flowered with the beginning of the rainy season from January to March, with fruit production following shortly thereafter and seed dispersal extending into the dry season from June to September.

#### Distribution

**Figure 32.**
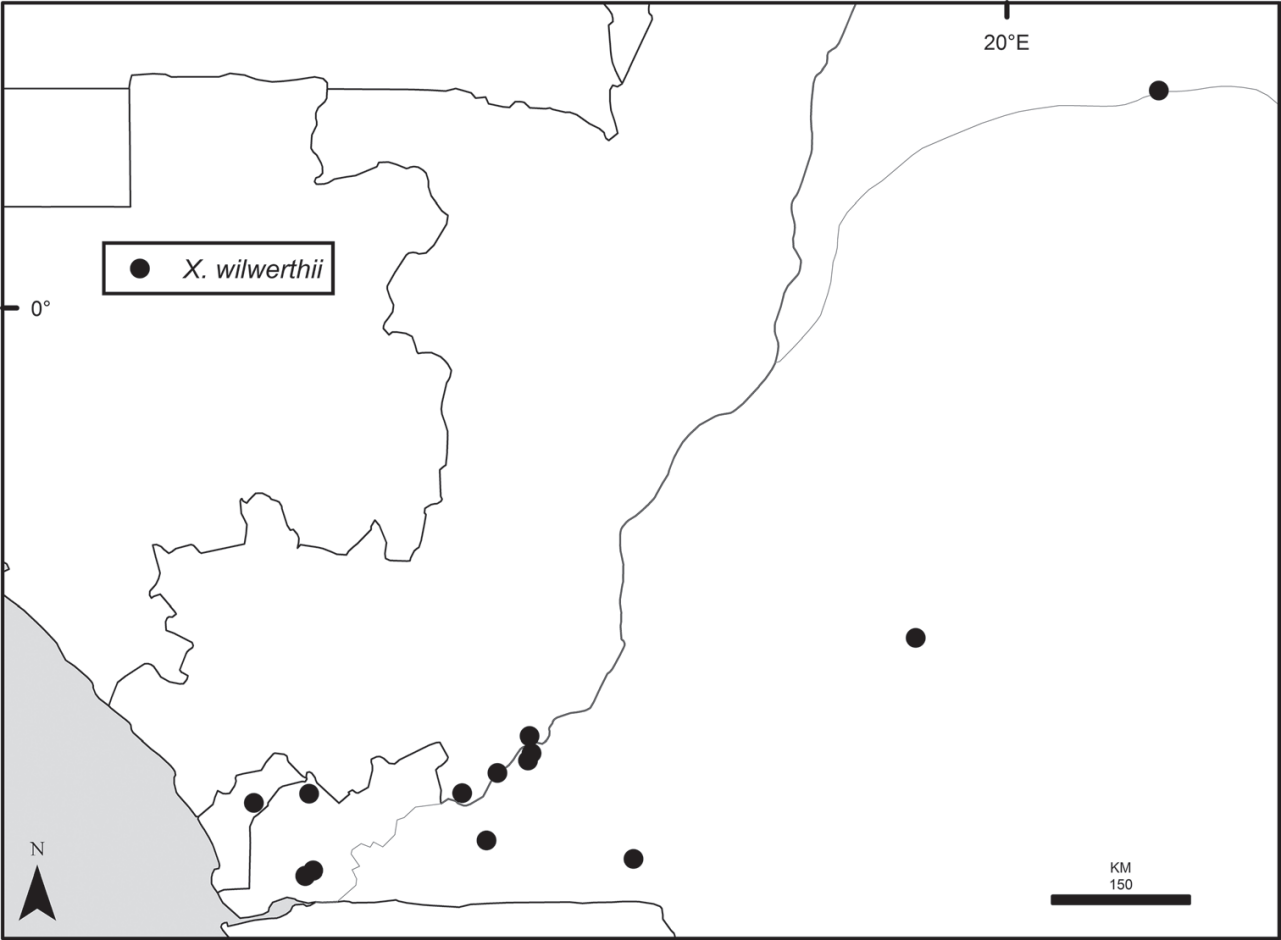
Distribution of *Xylopiawilwerthii*. Bolder lines represent country borders, fainter lines lakes and major rivers.

(Fig. 32). Occurs in and near the lower Congo River drainage in the southern Republic of the Congo, western Democratic Republic of Congo, and the Cabinda Province of Angola, where it grows in lowland forest, gallery forest, and forest/savanna edges at elevations of 400–500 m.

#### Local names.

Bengedele (*Donis 2041*), bengelele (*Devred 3083*), nginsa (*Pauwels 3478*).

#### Additional specimens examined.

**REPUBLIC OF THE CONGO.** Foulakari [see below], 21 Sep 1964 (fl), *Bouquet 497* (P); région ouest de Brazzaville, chutes de la Fulakari [04°34'58"S, 14°58'27"E], 11 Jun 1960 (fl), *Descoings 5852* (P); bord au Congo – Brazzaville, May 1950 (fl), *Koechlin 1112* (P); près de Boko (M. Congo), 8 Jan 1956 (fr), *Trochain 9603* [6451?] (P). **DEMOCRATIC REPUBLIC OF THE CONGO.** Kinshasa: Bas-Congo, Kinshasa, Kimuenza, plateau des restents, Luvanium [4.45917°S, 15.288889°E], 28 Mar 1966 (fl, fr), *Breyne 96* (MO); Lovanium, plateau of the residence, 400 m, 31 Jul 1965 (fl, fr), *Carrington 7* (K); Univ. Lovanium, Léopoldville camp Livulu, 26 June 1961 (fl), *Evrard 6317* (MO); Prov. Leopoldville, Territ. Kasangulu, Keimrwenza-Lobanium, plateau near the guest house, 500 m, 29 Jul 1957 (fl), *Robyns 4394* (K).—Kongo Central: Luki, Lukula, Parc forestier de la Nkula, 31 June 1978 (buds), *Breyne 3348* (MO); Prov. Leopoldville, Zundu, Mbanza-Ngungu [Territ. Thysville], 14 Mar 1960 (fr), *Coupère 1675* (K); I. N. E. A. C. Luki, 3 Feb 1947 (fr), *Devred 3083* (BR, P); Kiobo, 22 Sep 1945 (fr), *Donis 368* (K); Luki, 11 Oct 1948 (fl), *Donis 2041* (BR, K, P); Prov. Léopoldville, Terr. Boma, Luki, parc de la n’Kula, Oct 1948 (fr), *Maudoux 72* (K, WAG); Bas-Congo, Luki, INEAC, 1957 (fr), *Mahieu 53* (BR); Bas-Congo, Luki, 1959, *Mahieu 295* (BR).—Kwango: Terr. Popokabaka, Bombo Makuka, 26 Jun 1959 (st), *Pauwels 3478* (BR); Luki, 13 Jan 1947 (fr), *Toussaint 2114* (K).—Maï-Ndombe: Taketa, terr. Oshwe, 3°15'S, 19°06'E, 21 Jul 1962 (fl), *Jans 1110* (BR). **ANGOLA.** In dense woods E of residence of Bungo Mungo, Mayumbe, 11 Jan 1916 (fr), *Gossweiler 6169* (BM).

The long pedicel of *Xylopiawilwerthii* is unique among African species and unusual within the genus worldwide. This, along with the glabrous caudate leaves, reliably distinguish the species. An unusual aspect of its floral development is that elongation of the axillary pedicel seems to precede elongation of the terminal shoot from which it arises. The orange sarcotesta of its seeds suggest its placement within the *X.odoratissima* group, but it is isolated there.

Xylopiawilwerthiivar.cuneata was distinguished by the leaves being more narrowly cuneate at the bases than in the type specimen of the nominate variety, but we have now seen specimens where this range of variation in the leaf base is encompassed by a single specimen, and the two specimens do not show any other differences, so we do not distinguish them taxonomically. More perplexing is the variation in size and shape of the monocarps; monocarps with larger numbers of seeds are more slender and torulose than those with only 1–3 seeds, which tend to be wider and less strongly constricted between the seeds.

*Xylopiawilwerthii* and *X.flamignii* have similar leaves and overlap in distribution, and are thus likely to be confused, especially in the fruiting condition. *Xylopiaflamignii* differs in having a denser persistent indument of the young twigs, a prominent raised reticulum formed by the higher-order veins on the adaxial leaf surface, a distinctly pubescent lower leaf surface, leaves with 13–17 secondary veins per side, up to 22 monocarps per fruit, and seeds only 7–7.6 mm long. *Xylopiaflamignii* is also a larger tree, of up to 30 m in height, reaching canopy or subcanopy level. In contrast, *X.wilwerthii* is an understory tree with finely pubescent and soon glabrate twigs, indistinct higher-order veins on the adaxial leaf surface, leaves with 15–22 secondary veins per side, never more than 12 monocarps per fruit, and seeds 9.6–10.8 mm long.

Couralet (2010) reported *X.wilwerthii*, along with *Aidiaochroleuca* and *Corynanthepaniculata*, to be a dominant understory species in the Luki Reserve forests, which were characterized as tropical semi-evergreen forest with *Prioriabalsamifera* and *Terminalia superba* as important canopy species. Forest of *Marquesiaacuminata* was mentioned as the habitat at another site. We calculated a relatively large EOO for *X.wilwerthii* of 179,602 km^2^, coupled with an AOO of only 52 km^2^. The size of the EOO is largely due to the great distance of the type locality, Upoto in the Democratic Republic of the Congo, from the remainder of localities where the plant has been collected.

##### *Xylopiaacutiflora* group

Plants of this group vary in habit from shrubs to canopy-sized trees or rarely lianas. The leaves of several species, for example *X.cupularis*, *X.hypolampra*, and *X.villosa*, have a fine shiny appressed indument abaxially, a character not seen elsewhere among African species. The inflorescences may consist of a single flower, as in *X.acutiflora*, or, in the case of *X.paniculata*, up to 32 flowers, and pedunculate inflorescences are common. Monocarps of many species have a thick woody or leathery pericarp and seeds lying in two rows and oriented more or less perpendicular to the long axis of the monocarp. The sarcotesta of most species in this group is pale green, gray, or blue; *X.phloiodora* has an orange sarcotesta, and sarcotesta color is unknown for *X.acutiflora*, *X.calva*, *X.dinklagei*, *X.elliotii*, and *X.talbotii*. Four species within the *X.acutiflora* group were included in the molecular phylogenetic analysis of the genus by Stull et al. (2017), and all fell within a strongly supported clade along with several Madagascar species. In Africa this species group has a Guineo-Congolian distribution, reaching its eastern limits in South Sudan, western Tanzania, and central Angola.

Three subgroups within the *X.acutiflora* group are worth noting, all represented in the Stull et al. (2017) molecular study. The first subgroup comprises *X.acutiflora*, *X.dinklagei*, *X.mildbraedii*, *X.monticola*, *X.piratae*, *X.talbotii*, *X.thomsonii*, and *X.unguiculata*, most of them segregates of the former *X.acutiflora* s. l., These are shrubs, sometimes lianescent, or small trees with single-flowered inflorescences borne on short bract-covered pedicels. A second subgroup includes the common and widespread West and Central African riparian species *X.longipetala*, formerly known as *X.parviflora*, and *X.katangensis*. The third subgroup comprises species with large woody monocarps that often split into three segments upon dehiscence: *X.hypolampra*, *X.letestui*, *X.paniculata*, *X.phloiodora*, *X.tanganyikensis*, *X.villosa*, and probably *X.calva*. Species with a smaller fruit, which may be associated with this final subgroup, include *X.cupularis*, *X.elliotii*, and *X.pynaertii*.

### 26. Xylopia acutiflora

Taxon classificationPlantaeMagnolialesAnnonaceae

(Dunal) A. Richard, Hist. phys. Cuba, Pl. vasc. 1: 55. 1841 [“1845”].

6DFBCF17-E640-5D15-B9C1-C6A120BD6CBA

Fig. 33E–G

Unona acutiflora Dunal, Monogr. Anonac. 98, 116, t. 22. Aug–Nov 1817.
Coelocline acutiflora (Dunal) A. de Candolle, Mém. Soc. Phys. Genève 5: 208–209. 1832.
Xylopicrum acutiflorum (Dunal) Kuntze, Revis. gen. pl. 1: 8. 1891. Type. SIERRA LEONE [“Hab. in Americâ meridionali”]. Without definite locality, *H. Smeathman s. n.* (holotype: G-DC! [00201442]; isotypes: BM! [000510953, 000510954, 000511060, right-hand side of sheet], FI [004821]).
Unona oxypetala Candolle ex Dunal, Monogr. Anonac., Aug–Nov 1817.
Coelocline ? oxypetala (Candolle ex Dunal) A. de Candolle, Mém. Soc. Phys. Genève 5: 209. 1832.
Xylopia oxypetala (Candolle ex Dunal) Engler & Diels, Monogr. afrik. Pflanzen-Fam. 6: 63. 1901. Type. SIERRA LEONE. Without definite locality, *A. Afzelius s. n.* (lectotype: B! [100249555], as to the material with flowers; isotypes: BM! [000511060, left-hand side of sheet], FI! [005602]).

#### Description.

***Tree*** up to 15 m tall, d.b.h. up to 15 cm; bark smooth, pale brown to gray-brown. ***Twigs*** brown, pubescent, with erect hairs 0.7–1.5 mm long mixed with shorter (0.2–0.3 mm) hairs, eventually light gray to gray-brown, glabrate; nodes occasionally with two axillary branches. ***Leaf*** with larger blades 5.3–11.7 cm long, 2.3–4.3 cm wide, chartaceous, concolorous to slightly discolorous, elliptic to elliptic-lanceolate, apex acute to acuminate, the acumen (2.5–) 5–11 mm long, base cuneate to broadly cuneate, sparsely pubescent on the midrib but otherwise glabrous adaxially, sparsely appressed-pubescent with longer hairs on the midrib and margin to glabrate abaxially; midrib plane to slightly impressed adaxially, raised abaxially, secondary veins weakly brochidodromous, 9–14 per side, diverging at 60–70° from the midrib, these and higher order veins slightly raised adaxially, raised and forming a reticulum abaxially; petiole 3.2–5 mm long, canaliculate, pubescent to glabrate. ***Inflorescences*** axillary, 1-flowered, pubescent; pedicels 3.0–5.5 mm long, 1.2–1.5 mm thick; bracts imbricate on proximal half of pedicel, 3–6, persistent, 1.6–2.6 mm long, obovate, ovate, or orbicular, apex rounded and sometimes apiculate; buds linear-lanceolate, slightly falciform, apex acute to obtuse. ***Sepals*** slightly spreading to spreading at anthesis, 1/5–1/4-connate, 2.2–3.7 mm long, ca. 3 mm wide, coriaceous to fleshy, ovate to triangular, apex acute to occasionally acuminate, sericeous abaxially. ***Petals*** white to pale yellow, with a patch of red at the base *in vivo*; outer petals erect to somewhat spreading at anthesis, 19.6–37 mm long, (2.3–) 3.0–3.3 mm wide at base, 1.6–2.0 mm wide at midpoint, coriaceous, linear, apex obtuse, longitudinally ridged abaxially, puberulent but becoming glabrous in the basal third adaxially, sericeous abaxially; inner petals somewhat spreading at anthesis, 16–33 mm long, 2.4–3.2 mm wide at base, 1–1.2 mm wide at midpoint, coriaceous, linear, apex acute, base with undifferentiated margin, longitudinally ridged on both surfaces, pubescent on both surfaces except for the glabrous base. ***Stamens*** numerous; fertile stamens 1.4–1.6 mm long, narrowly oblong to clavate, apex of connective red *in vivo*, 0.2–0.3 mm long, shieldlike, glabrous, anthers 9–12-locellate, filament 0.4–0.5 mm long; outer staminodes 1.5–1.6 mm long, clavate to narrowly oblong, apex rounded to truncate; inner staminodes 0.9–1.1 mm long, rectangular, apex truncate; staminal cone 1.8–2.2 mm in diameter, 1.0–1.6 mm high, concealing lower half of the ovaries, rim laciniate. ***Carpels*** 7–13; ovaries ca. 1.2 mm long, oblong, densely pubescent, stigmas connivent, 2.7–3.0 mm long, filiform, glabrous except for an apical tuft of hairs. ***Torus*** concave beneath ovaries but otherwise flat, 1.8–2.5 mm in diameter. ***Fruit*** of up to 10 glabrate monocarps borne on a pedicel 5–9 mm long, 2.3–5.6 mm thick, sparsely pubescent to glabrate, often with short dead branch attached and bracts and sepals persistent; torus 5.9–11.5 mm in diameter, 4.2–7.5 mm high, depressed-globose, sunken where monocarps attached. ***Monocarps*** with a green, sometimes reddish-tinged, exterior and a scarlet endocarp *in vivo*, 2.5–5.1 cm long, 1.1–1.5 cm wide, 1.2–1.3 cm thick, oblong, sometimes weakly torulose, apex obtuse or with an oblique truncate beak 1.5–2 mm long, base contracted into a stipe 3–7 mm long, 2.9–4.5 mm thick, longitudinally ridged or wrinkled, verrucose; pericarp 0.7–1.2 mm thick. ***Seeds*** up to 20 per monocarp, in two rows, lying perpendicular to long axis, 11–12 mm long, 6.5–7.9 mm wide, 5.8–5.9 mm thick, ovoid to ellipsoid, wedge-shaped in cross-section, truncate at micropylar end, rounded at chalazal end, brown, smooth, dull, raphe/antiraphe not evident, micropylar scar ca. 3.5 mm long, ca. 1.5 mm wide, oblong-ovate; sarcotesta color unknown *in vivo*, forming a waxy crust on dried seeds; aril absent.

**Figure 33.**
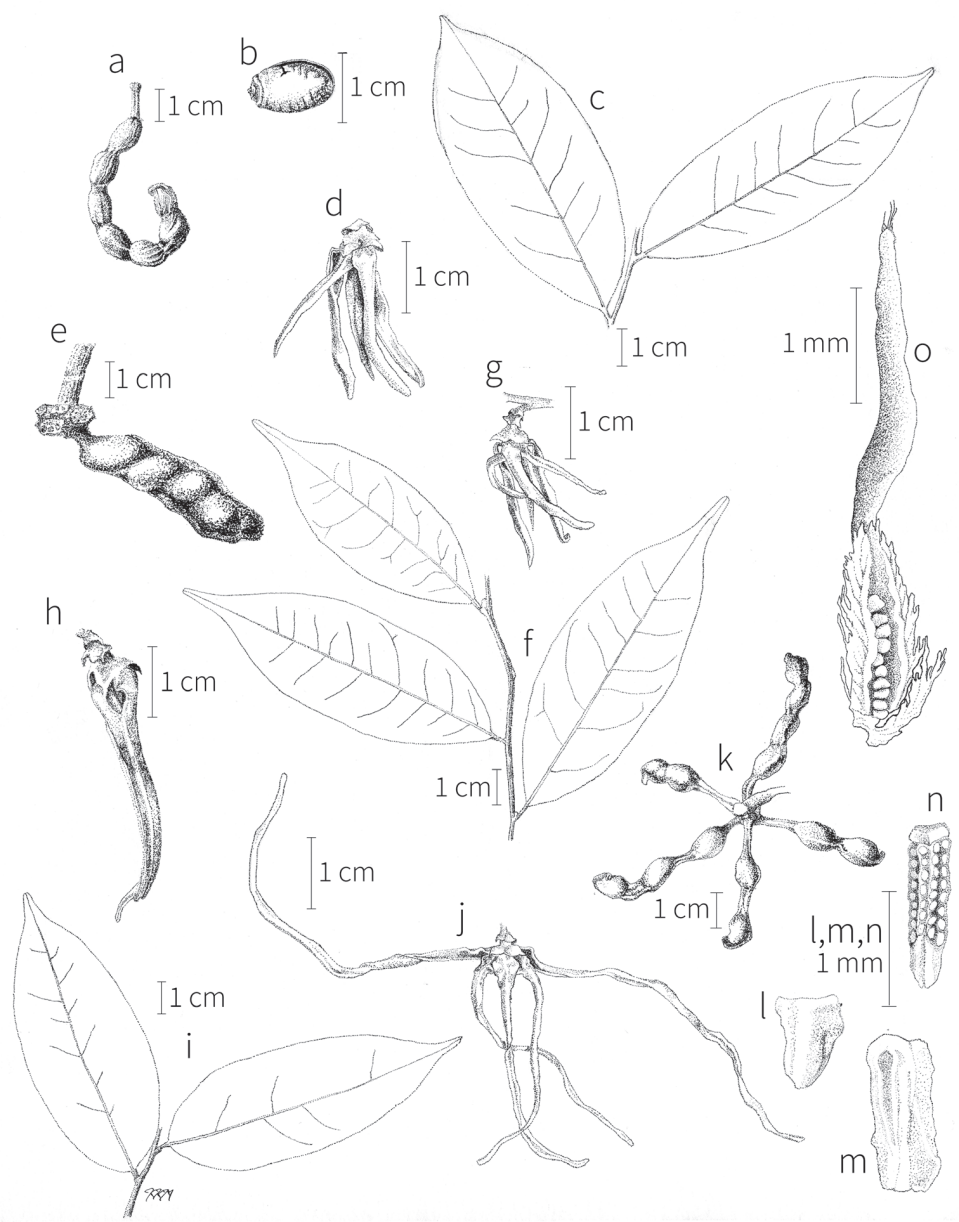
*Xylopiaunguiculata*, *X.acutiflora*, and *X.piratae*. **A–D, L–O***X.unguiculata***A** Monocarp, lateral view **B** Seed **C** Leaves **D** Flower, lateral view **L, M** Staminodes, abaxial view **N** Stamen, abaxial view **O** Carpel, lateral view with ovary wall cut away to show arrangement of ovules **E–G***X.acutiflora***E** Monocarp, lateral view, attached to pedicel **F** Leaves **G** Flower, lateral view **H–K***X.piratae***H** Flower bud, lateral view **I** Leaves **J** Flower, lateral view **K** Fruit. **A–B** from *Le Testu 1179* (BM) **C–D** from *Reitsma & Reitsma 1923* (NY) **E–G** from *Beentje 879* (WAG) **H–J** from *de Koning 4999* (WAG) **K** from de *Wilde 356* (P) **L–O** from *McPherson 16960A* (MO).

#### Phenology.

Specimens with flowers have been collected from February to June and in September, and with fruits in February and from August to October.

#### Distribution

**Figure 34.**
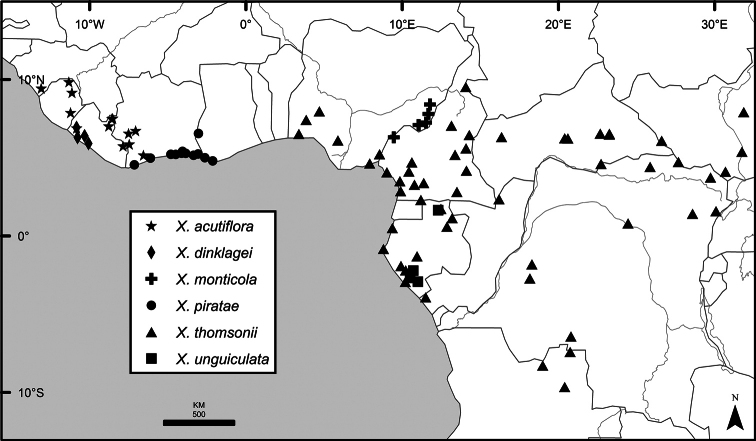
Distributions of *Xylopiaacutiflora*, *X.dinklagei*, *X.monticola*, *X.piratae*, *X.thomsonii*, and *X.unguiculata*. Bolder lines represent country borders, fainter lines lakes and major rivers.

(Fig. 34). Occurs in Sierra Leone, Guinea, Liberia, and Ivory Coast, in both primary and secondary forests at low to middle elevations.

#### Local names.

Elo blanc (*Aubréville 90*), gbaa (*Yallah 51*).

#### Additional specimens examined.

**GUINEA.** Environs de Forécariah, bosquet de Kankan nara, Jun 1937 (fl), *Jacques-Félix 1715* (P). **SIERRA LEONE.** Kabala, Mt. Loma, Seisikoro, 6 Dec 1965 (st), *Adam 22396* (MO); Falaba, 2 Apr 1914 (fl), *Aylmer 29* (MO); Gola Forest, 25 Apr 1952 (buds), *Small 639* (B, MO); Gola Forest, 14 May 1952 (fl), *Small 664* (MO). **LIBERIA.** Yekepa, Mt. Nimba, 13 Oct 1969 (st), *Adam 24221* (MO); Yèkèpa, Yekepa, Mt. Nimba (Mt. Yuelliton), 25 May 1970 (fl), *Adam 25678* (K, MO); Yekepa, Mt. Nimba (Mt. Gangra), 21 Oct 1971 (fr), *Adam 26395* (MO, P); Yekepa, Nimba, New Camp Grassfield, 14 May 1973 (fl), *Adam 27551* (MO, P, PRE, WAG); Eastern Province, Tchien District, Ziah Town [“Zeahtown”], 1 Aug 1947 (fr), *Baldwin 6970* (K, MO); Monserrado Co., Bomi Hills, 3 Feb 1950 (fl), *Baldwin 14086* (K, MO, NY, US); Gola-Yoma National Forest, Bomi Hills, 6 Feb 1966 (fl), *van Meer 390* (MO, WAG); Nimba area, 10 Apr 1962 (fl), *Voorhoeve 1089* (B, M, MO, WAG—2 sheets); Gola National Forest, ca. 15 km NE of Bomi Hills, 17 Apr 1962 (fl), *de Wilde & Voorhoeve 3833* (A, B, BR, K, P, WAG—3 sheets); Mt. Bele, 16 Apr 1965 (fl), *Yallah 51* (K, P). **IVORY COAST.** Taï (Aubréville 1959), without definite locality, 9 Feb 1957 (fr), *Aubréville 90* (P—3 sheets); 10 km ESE of Taï, 05°50'N, 07°22'W, 6 Sep 1975 (fl, fr), *Beentje 879* (WAG—2 sheets); vicinibus oppidi Tienkulà, ad occidentem reipublicae, 1 Mar 1962 (fl), *Bernardi 8315* (A, G—2 sheets, K, M, MO, US—2 sheets); 80 km S of Soubré, 3 Apr 1968 (fl), *Geerling & Bokdam 2478* (K, MO, WAG—2 sheets); Taï, 11 Sep 1975 (fl, fr), *de Koning 5996* (MO, WAG—2 sheets); Guiglo, Taï, 05°52'N, 7°27'W, 1 Mar 1982 (fl), *Stäuble NS 0460* (MO).

*Xylopiaacutiflora* is one of six species belonging to a geographically widespread complex of species that vary in habit, indument, and flower and fruit morphology, but all formerly considered as representing a single species. *Xylopiaacutiflora* s. s. is a small tree, with a mix of short and long hairs on the twigs, petals reaching 37 mm in length, and short broad monocarps with two rows of seeds. It is restricted to a relatively small area of West Africa in lowland and lower montane forests. It overlaps in Liberia with *X.dinklagei*, but that species is a shrub or liana with uniform short hairs on the twigs, petals reaching only 13 mm in length, and longer and narrower monocarps with a single row of seeds. *Xylopiapiratae* from the Ivory Coast and Ghana likewise differs in being a liana with narrow monocarps, but has variable twig indument like *X.acutiflora* and much longer petals, reaching 73 mm in length.

Information about *Xylopiaacutiflora* pertaining to plants from areas east of Ivory Coast probably applies to other species. In most cases, these refer to segregate species of the larger *X.acutiflora* complex, but the report of *X.acutiflora* for the *Flora Zambesiaca* area (Robson 1960) is based on the specimen *Holmes H.1273* (K), which is not a member of this group at all. It is most similar to *X.elliotii* and is discussed under that species.

The ecology of *X.acutiflora* is largely unknown. In Taï National Park in western Ivory Coast, the seeds of *Xylopiaacutiflora* were eaten and spat out by the monkey, *Colobuspolykomo*, which fed on the seeds of other *Xylopia* species as well (Koné et al. 2008). A new rust species, *Sphaerophragmiumxylopiae* Beenken & R. Berndt, was recently described from teliospores found attached to the specimen *Bernardi 8315* (Beenken and Berndt 2010). This rust genus seems to be confined to Fabaceae and Annonaceae.

Historically, there has been confusion over the application of the name *Xylopiaacutiflora*. Bentham (1862) accepted three African species of *Xylopia*, *X.aethiopica*, *X.acutiflora*, and *X.parviflora*, the latter being a new combination based on the *Uvariaparviflora* Rich. Bentham expressed doubt, however, about the distinctness of the latter two species, and even whether the plant described as *Unonaoxypetala* Dun. was distinct from them. The specimens cited by Bentham under both *X.acutiflora* (Barter from the confluence of the Quorra and Chadda Rivers, Chr. Smith from the Congo) and *X.parviflora* (Vogel and Barter from the Niger, G. Mann from the Bagroo River) are all *Xylopialongipetala*. Oliver (1868) maintained Bentham’s concept, and it is not surprising that Vallot (1882) found it necessary to argue that there were two distinct species, and that Bentham had confused them. Vallot proposed *Xylopiadunaliana* Vallot as a replacement name for *X.acutiflora* (under which he placed *Unonaoxypetala* in synonymy), and he retained the name *X.parviflora* for the second species, but *X.dunaliana* Vallot is a superfluous name and illegitimate.

The type material of *Unonaacutiflora* in G-DC comprises 4 sheets of a collection made by Smeathman, each sheet with slightly different labeling but all seeming to contribute to the description and illustration in Dunal (1817) and so here regarded as the holotype. There are three sheets of a Smeathman collection at BM that are considered to be isotypes. As with the combination *Xylopiaaethiopica* made by Richard, we follow the conclusion of Brizicky (1962) in accepting the year of publication of the combination *Xylopiaacutiflora* as 1841, rather than the printed date of 1845. Dunal published Candolle’s manuscript name *Unonaoxypetala* in the same publication as *Unonaacutiflora* (Dunal 1817), which was based on a specimen collected by Afzelius and seen by Candolle in the Lambert Herbarium. The two names therefore have equal priority, but the name *X.acutiflora* has historically been used for the species. According to Miller (1970), the specimens of Afzelius in the Lambert Herbarium were purchased by William Pamplin, working on commission for Wilhelm Friedrich Klotzsch, who acquired the specimens for the Berlin herbarium. An Afzelius specimen, identifiable as this species, survives at B, but the specimen has both flowers and fruits, and only flowers are mentioned in the protologue. The fruits and seeds are not attached to the twigs, and it is possible that they became associated with the specimen later. We initially labeled the B sheet as an isotype, pending a better understanding of the distribution of the Lambert Herbarium collections, but it is now clear that this specimen, and specifically the branches with flowers attached, is more appropriately designated as a lectotype.

### 27. Xylopia calva

Taxon classificationPlantaeMagnolialesAnnonaceae

D. M. Johnson & N. A. Murray sp. nov.

993340B0-18C5-5DD1-A92D-DA13C20A6D9D

urn:lsid:ipni.org:names:60476243-2

Fig. 35Q–T

#### Diagnosis.

Species resembling *Xylopiaphloiodora* in the short branched inflorescences and large ellipsoid fruits, but differing in the much broader oblong-lanceolate petals that are glabrous adaxially except for a fringe of hairs on the apices, the leaves sometimes glaucous abaxially, the staminal cone smaller and the rim laciniate, the anthers only 10–13-locellate, and the lack of pronounced reticulum on the adaxial surface of the leaf.

#### Type.

CAMEROON. South Region, Bipinde, 1903 (fl), *G. A. Zenker 4747* (holotype: BM [000511011]; isotypes: G! K! L! [0191105], M! MO! [751089]).

#### Description.

***Tree*** of unknown height, d.b.h. 30 cm, bole straight, cylindrical, slender, with buttresses reaching a height of 1 m from the base; bark pinkish gray with white flecks, smooth. ***Twigs*** dark brown to gray, pubescent, the hairs 0.3–0.4 mm long, soon glabrate; nodes occasionally with two axillary branches. ***Leaf*** with larger blades 10–17.2 cm long, 3.6–6.5 cm wide, chartaceous to subcoriaceous, concolorous but sometimes slightly glaucous abaxially, elliptic to elliptic-oblong, apex acuminate, the acumen (3–) 9–21 mm long, base broadly cuneate to rounded and short-decurrent on the petiole, with a few hairs along the midrib adaxially, sparsely appressed-pubescent abaxially; midrib plane to slightly impressed adaxially, raised abaxially, secondary veins irregularly brochidodromous, 8–15 per side, diverging at 50–65° from the midrib, these and higher-order veins slightly raised on both surfaces; petiole 3–10 mm long, shallowly canaliculate, sparsely pubescent. ***Inflorescences*** axillary, 1–10-flowered, rusty-pubescent; peduncle 1 per axil, 1–3 mm long; pedicels 1–10 per peduncle, 5–8 mm long, 1.3–1.5 mm thick; bracts 1–2, evenly spaced on pedicel, the lower caducous and the upper persistent, 2–3 mm long, broadly ovate to semicircular, apex obtuse to acute; buds lanceolate, apex obtuse. ***Sepals*** slightly spreading at anthesis, 1/4–1/3 connate, 3.8–4.5 mm long, 4.4–5.1 mm wide, coriaceous, broadly ovate, apex acute to obtuse, densely rusty-pubescent abaxially. ***Petals*** pale yellow, inner petals reddish at the base adaxially *in vivo*; outer petals probably slightly spreading at anthesis, 13–23 mm long, 3.8–6 mm wide at base, 2.8–5.5 mm wide at midpoint, fleshy, oblong-lanceolate, apex obtuse, densely puberulent only on trigonous apex adaxially, appressed-pubescent except for a glabrous patch at the base abaxially; inner petals probably slightly spreading at anthesis, 10–17.5 mm long, 2.5–4 mm wide, 2.2–3.0 mm wide at midpoint, fleshy, oblong-lanceolate, apex acute, base with undifferentiated margin, puberulent at apex but otherwise glabrous adaxially and uniformly puberulent except for the glabrous base abaxially. ***Stamens*** ca. 200; fertile stamens 1.4–2.2 mm long, narrowly oblong, apex of connective red *in vivo*, 0.1–0.3 mm long, dome-shaped to shieldlike, overhanging the anther thecae, papillate, anthers 10–13-locellate, filament 0.7–0.8 mm long; outer staminodes 2.1–2.8 mm long, clavate to oblong, apex obtuse, rounded, or truncate; inner staminodes 1.1–1.2 mm long, oblong, apex truncate; staminal cone 1.6-2.5 mm in diameter, 0.9–1 mm high, partially concealing the ovaries, rim laciniate. ***Carpels*** ca. 9; ovaries ca. 1.8 mm long, narrowly oblong, pubescent, stigmas connivent at the base, ca. 2.2 mm long, linear but thickened in the middle, with a tuft of hairs at the apex. ***Torus*** flat, 2.5–3.5 mm in diameter. ***Fruit*** of up to 8 pubescent monocarps borne on a pedicel 6–6.8 mm long, 2.4–2.5 mm thick, rusty-pubescent; torus 5.8–6 mm in diameter, 3–4.5 mm high, depressed-globose. ***Monocarps*** ca. 3.2 cm long, ca. 1.7 cm wide and thick, ellipsoid, not torulose, apex obtuse, base narrowed, contracted into a stipe ca. 3 mm long, ca. 7 mm thick, longitudinally wrinkled, with strips of rusty pubescence; pericarp ca. 2.5 mm thick. ***Seeds*** 7–8 per monocarp, in two rows, lying perpendicular to long axis, ca. 9.3 mm long, 6.5–6.8 mm wide, 4.5–6.1 mm thick, oblong, elliptic in cross-section, truncate at micropylar end, rounded at chalazal end, brown, smooth or slightly wrinkled, dull, raphe/antiraphe faintly evident, micropylar scar not observed; sarcotesta *in vivo* unknown; aril absent.

**Figure 35.**
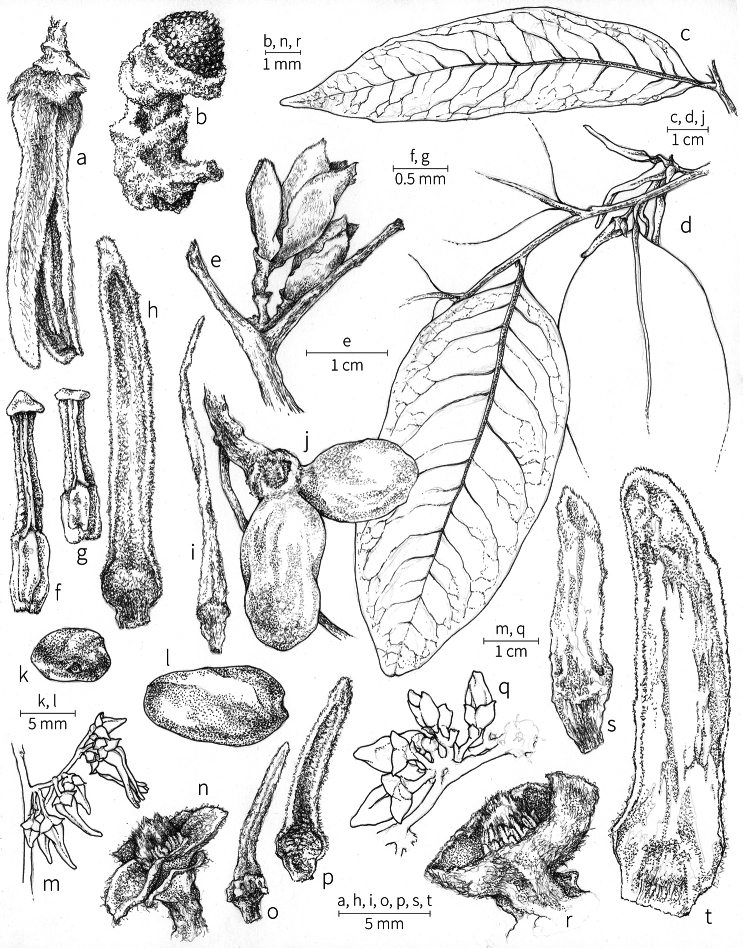
*Xylopiaphloiodora*, *X.paniculata*, and *X.calva*. **A–L***X.phloiodora***A** Opening flower, lateral view **B** Old floral receptacle, showing staminal cone **C** Leaf, narrow extreme **D** Habit **E** Expanding vegetative shoot **F, G** Stamens, abaxial view to show size variation **H** Outer petal, adaxial view **I** Inner petal, adaxial view **J** Fruit **K** Seed, view from micropylar end **L** Seed, lateral view **M–P***X.paniculata***M** Inflorescence **N** Flower with petals and stamens removed, to show staminal cone **O** Inner petal, adaxial view **P** Outer petal, adaxial view **Q–T***X.calva***Q** Inflorescence **R** Flower with stamens and petals removed, to show staminal cone **S** Inner petal, adaxial view **T** Outer petal, adaxial view. **A, B, D, F–I** from *Louis 13430* (US) **C** from *Louis 7220* (WAG) **E** from *Tisserant 1113* (BM) **J** from *Tisserant 1138* (P) **K, L** from *Fay and Harris 1138* (MO) **M–P** from *Reitsma & Reitsma 1163* (NY) **Q–T** from *Letouzey 10306* (P).

#### Phenology.

Specimens with flowers were collected in April, and with young fruits in May.

#### Distribution

**Figure 36.**
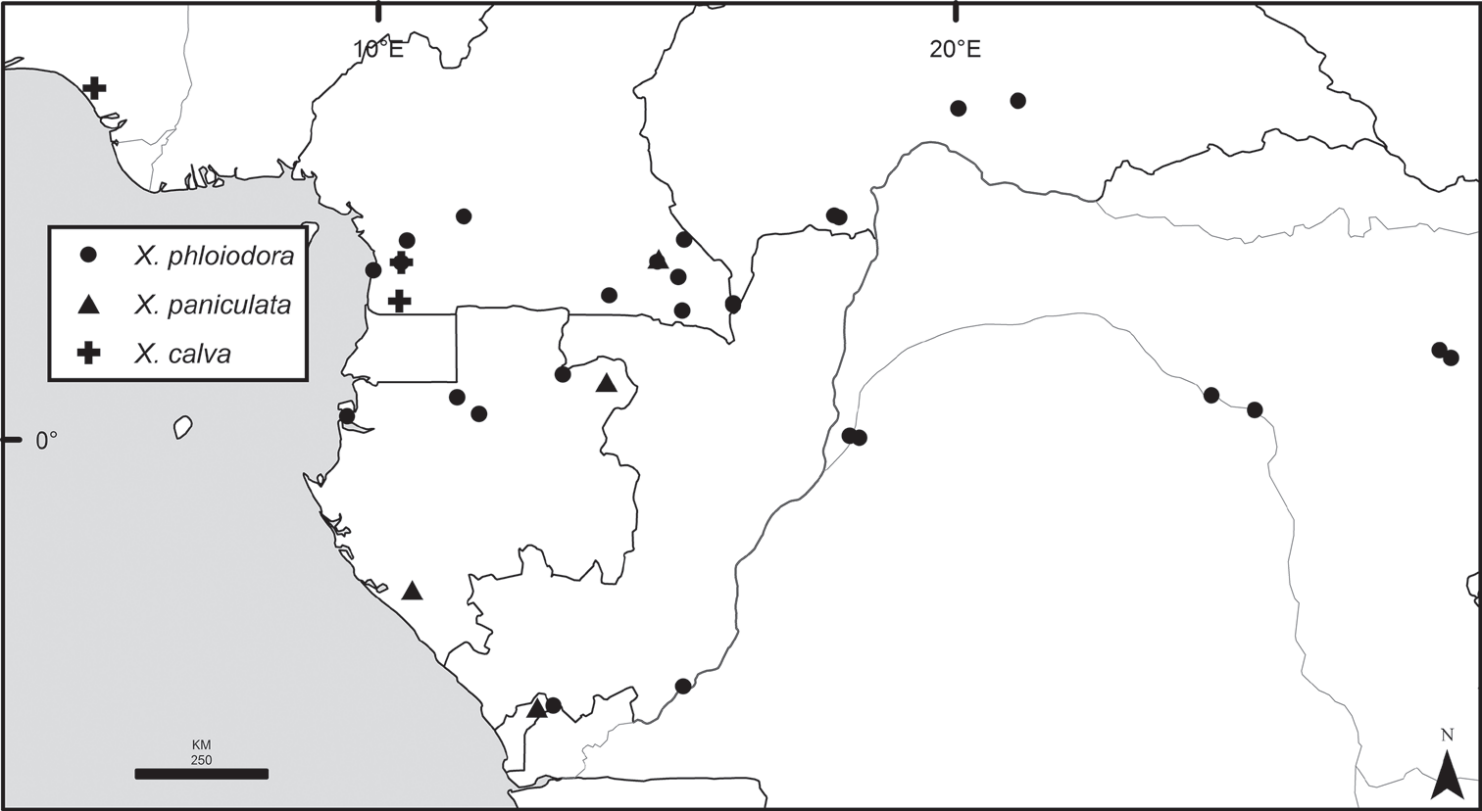
Distributions of *Xylopiaphloiodora*, *X.paniculata*, and *X.calva*. Bolder lines represent country borders, fainter lines lakes and major rivers.

(Fig. 36). Known from three localities, two of them in southwestern Cameroon and the other in south-central Nigeria, from low elevation forest.

#### Local name.

Ohun (*Ross R.202*).

#### Additional specimens examined.

**NIGERIA.** Edo State, Okomu Reserve, near U. A. C. timber camp, Onikaroga, 12 May 1934 (yg fr), *Ross R.202* (K). **CAMEROON.** Près des chutes du Ntem ou de Menve’ele près Nyabessan (60 km E de Campo), 8 Apr 1970 (fl), *Letouzey 10306* (P).

*Xylopiacalva* is distinguished by its long broad petals that are glabrous adaxially (L. *calva*, “bald”) except at the apices. It is most similar to *X.phloiodora*, having the same pedunculate and branched inflorescence found in that species, but may be distinguished by the glaucous tinge to the abaxial surface of the leaves, straight rather than arcuate secondary veins, indistinct higher-order venation, outer petals 2.8–5.5 mm wide at the midpoint, anthers only 10–13-locellate, and laciniate rim of the staminal cone. The only fruit seen was not fully mature.

The type specimen was initially identified as *Xylopiaaethiopica* and filed in herbaria under that name. The other two specimens were only identified as “*Xylopia* sp.” The specimen *Ross R.202* is labeled as having been seen for the second edition of Flora of West Tropical Africa (Keay 1954–1958), but there is no mention of this specimen in the *Xylopia* treatment of that work. We calculated an EOO of 22,406 km^2^ and an AOO of 12 km^2^ for this species; the fact that there are only three collections, the most recent one from 1970, suggests that the species should be of conservation concern.

### 28. Xylopia cupularis

Taxon classificationPlantaeMagnolialesAnnonaceae

Mildbraed, Notizbl. Bot. Gart. Berlin-Dahlem 8: 56–57. 1921.

9ABB649B-BC68-52D9-AE68-0143AB3E140D

Fig. 37

Xylopia gilviflora Exell, J. Bot. 73: Suppl. 4. 1935. Type. ANGOLA [“Portuguese Congo”]. Cabinda Province, in the forests at Buco Zau, Mayumbe, 15 Jan 1917, *J. Gossweiler 6933* (holotype: BM!, photos at GH! MO! NY!; isotypes: B! [100153140], COI! [00004882], LISC! [000308, 000309, 000310, 000311, 000312, 000313, 000314, 000315, 000316]).
Xylopia chrysophylla J. Louis ex Boutique, Bull. Jard. Bot. État 21: 108–109. 1951. Type. DEMOCRATIC REPUBLIC OF THE CONGO [“Belgian Congo”]. Tshopo Province, District Forestier Central, Yangambi, à 7 Km à l’Est du poste, alt ± 470 m, June 1937, *J. Louis 4309* (holotype: BR!; isotypes: AAU! BR! [0000008824752], L! MO! [1639095, 3007016], NY! [00066781], US! [2091336]).

#### Type.

CAMEROON. East Region, weit nach Norden vorgeschobener Ausläufer des äquatorialen Waldgebietes südlich Deng-deng, etwa 250 km nordöstlich Jaunde, Mar 1914, *J. Mildbraed 8649* (holotype: B! [100153141]; isotype: BM! [000511053]).

**Figure 37.**
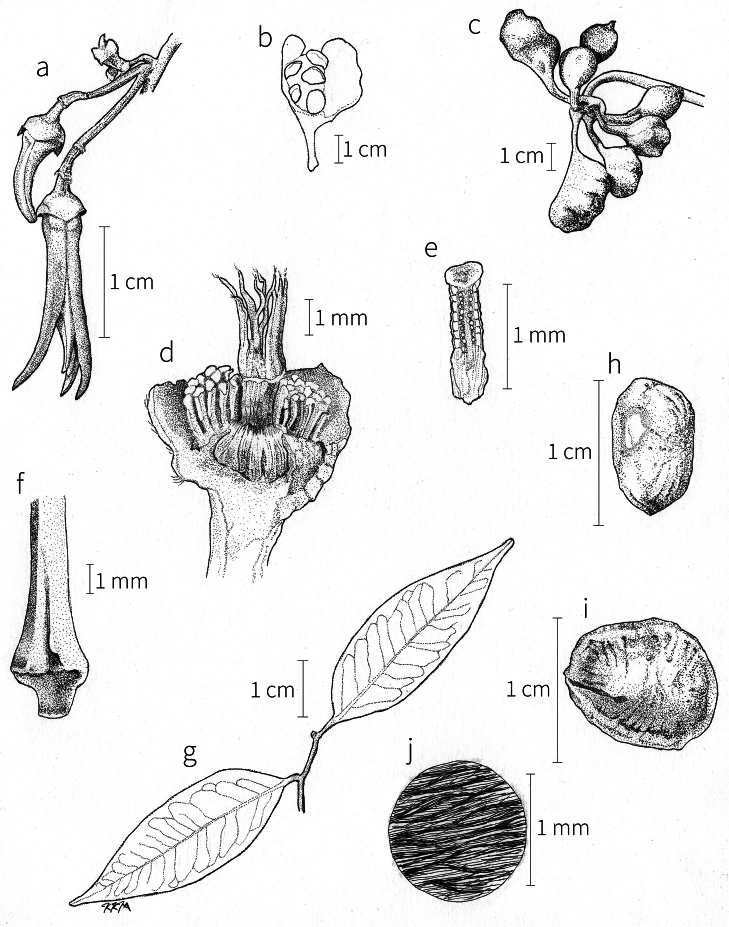
*Xylopiacupularis*. **A** Bud and partially open flower **B** Diagrammatic drawing of dehisced monocarp, showing seed arrangement **C** Fruit **D** Longitudinal section of flower with petals removed, to show the calyx, stamens attached to staminal cone, and carpels inside the cone **E** Stamen, abaxial view **F** Base of inner petal, adaxial view **G** Leaves **H** Seed, viewed from micropylar end **I** Seed, side view **J** Detail of indument on lower leaf surface. **A, D–G** from *Gerard 5583* (MO) **B, C, H–J** from *Sita 4118* (P).

#### Description.

***Tree*** up to 35 m tall, d.b.h. up to 50 cm; bole cylindrical, with small buttresses at the base; bark light-colored, tinted with pink, gray, brown, or green, smooth. ***Twigs*** reddish brown, finely appressed-pubescent, the hairs 0.1–0.3 mm long, eventually gray brown to light gray, glabrate; nodes commonly with 2–3 axillary branches. ***Leaf*** with larger blades 4.6–7.8 cm long, 1.3–2.4 cm wide, chartaceous, discolorous, lanceolate to elliptic or elliptic-oblong, apex acuminate (sometimes minutely truncate), the acumen 6–11 mm long, base cuneate to broadly cuneate, often slightly oblique, with a few hairs on the midrib but otherwise glabrous adaxially, sparsely to densely golden-sericeous abaxially, becoming more sparsely so with age; midrib slightly raised adaxially, raised abaxially, secondary veins indistinctly brochidodromous, 10–15 per side, diverging at 45–60° from the midrib, these and higher-order veins slightly raised on both surfaces, less distinct abaxially; petiole 2–5 mm long, flat to shallowly canaliculate, sparsely pubescent. ***Inflorescences*** axillary, 1–2 (–3)-flowered, sparsely pubescent; peduncles rarely present, up to 4 mm long; pedicels usually not pedunculate, rarely up to 3 per peduncle, 4.5–12 mm long, 0.7–0.9 mm thick; bracts 2, both attached distal to pedicel midpoint, caducous, 1.5–1.9 mm long, semicircular, apex broadly acute to obtuse; buds linear-lanceolate, apex acute. ***Sepals*** erect to slightly spreading at anthesis, 1/2–3/4-connate, 2.1–3.3 mm long, 2.5–3.5 mm wide, coriaceous, broadly ovate, apex broadly acute to obtuse, pubescent abaxially. ***Petals*** cream-colored to yellow *in vivo*; outer petals appear spreading at anthesis, 15.5–36 mm long, 2.2–3.4 mm wide at base, 1.2–2.2 mm wide at midpoint, fleshy, linear-lanceolate, apex acute, densely pubescent adaxially, sericeous abaxially; inner petals appear spreading at anthesis, 13.5–17.7 mm long, 2.4–3.5 mm wide at base, 0.9–1.5 mm wide at midpoint, fleshy, linear, apex acute, base with undifferentiated margin, puberulent on both surfaces except for glabrous base. ***Stamens*** 160–200; fertile stamens 1.0–1.6 mm long, narrowly oblong, apex of connective 0.1–0.3 mm long, depressed-globose, overhanging the anther thecae, glabrous, anthers 9–10-locellate, filament 0.2–0.6 mm long; outer staminodes 1.3–1.4 mm long, oblong, apex obtuse to rounded; inner staminodes 0.9–1.0 mm long, oblong, apex truncate; staminal cone 1.7–2.3 mm in diameter, 0.7–1.0 mm high, partially concealing the ovaries, rim laciniate. ***Carpels*** 12–20; ovaries ca. 1 mm long, ellipsoid to oblong, densely pubescent, stigmas connivent, 2.5–2.8 mm long, filiform, with a tuft of hairs at the apex. ***Torus*** flat, 2.1–2.7 mm in diameter. ***Fruit*** of up to 18 glabrate monocarps borne on a pedicel 6–15 mm long, 3–10 mm thick, glabrate; torus 6.6–16 mm in diameter, 5–6.5 mm high, depressed-globose. ***Monocarps*** with reddish green exterior and pink to dark red endocarp *in vivo*, 2.3–5.4 cm long, 1.4–1.9. cm wide, 1.2.–2.0 cm thick, irregularly oblong or obovoid, weakly torulose, apex rounded, base contracted into a stipe (5–) 7–24 mm long, 1.5–5 mm thick, sometimes wrinkled, verrucose; pericarp 0.8–2 mm thick. ***Seeds*** 6–8 per monocarp, in two rows, lying oblique to perpendicular to long axis, 9.6–13.4 mm long, 6.1–9.6 mm wide, 5.5–7 mm thick, flattened-ellipsoid to broadly ellipsoid, wedge-shaped in cross-section, truncate at micropylar end, rounded at chalazal end, dark brown, mostly smooth but finely marked with pits and wrinkles, dull, raphe forming a keel, antiraphe partially sunken into a groove but not evident by the micropylar scar, micropylar scar 1.5–2.6 mm long, 0.6–2.3 mm wide, ovoid, oblong, or deltoid; sarcotesta glaucous blue or gray *in vivo*; aril absent.

#### Phenology.

Specimens with flowers have been collected in all months of the year, and with fruits in all months except February, March, and December.

#### Distribution

**Figure 38.**
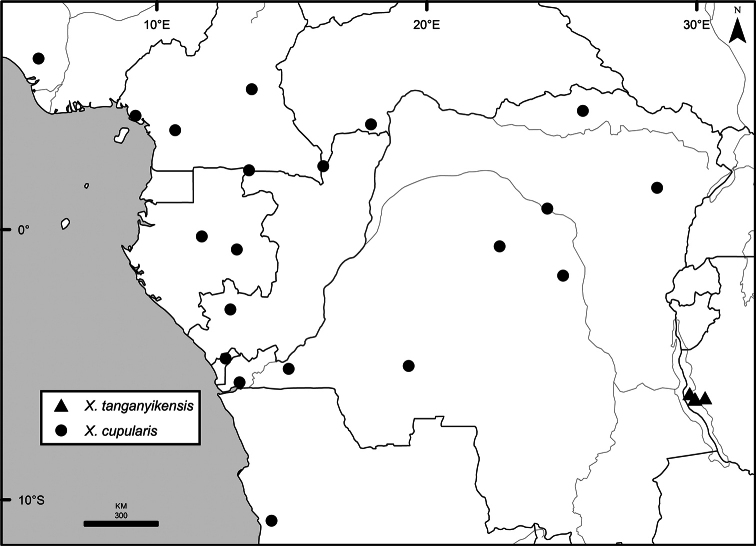
Distributions of *Xylopiacupularis* and *X.tanganyikensis*. Bolder lines represent country borders, fainter lines lakes and major rivers.

(Fig. 38). Distributed from southeastern Nigeria east to the southern Central African Republic and south to Cuanza Sul Province of Angola and the northeastern Democratic Republic of the Congo, occurring in rainforest, semi-deciduous forest, forest-savanna edges, and secondary forest at elevations of 50–800 m.

#### Local names.

Aganda (Basoko, *Louis 4309*), bompaie bo fufow bo lowe (Turumbu, *Louis 4309*), inaolo a bompaie bo fufow (Turumbu, *Louis 16550*), lucangua (*Matton 11*), molo-nyama (Lissongo, *Tisserant 2295*), molo-nzange (Lissongo, *Tisserant 1768*), odjobi (Yaoundé, *Mbarga 1940 SRFK*), sange (*Ekuba 1615*, *Hart 572*), sange-petite (Kibila, *Marabo 1546*).

#### Representative specimens.

**NIGERIA.** Benin Province, Iyekuselu District, in field 8 at W. A. I. F. O. R., 8 Dec 1961 (fl), *Daramola FHI 45672* (K). **CAMEROON.** Subdivision Eseka près de Badjob, 17 Jun 1955 (fl), *Mbarga 58 [SRFK 1940*] (P); Likomba-Pflanzung, 15–35 km NE von Victoria, 50–100 m, Nov 1928 (st), *Mildbraed 10629* (A, K); PK 30 route Mintom I (70 km E de Djoum)-Alati (100 km SE de Djoum), 8 Jan 1973 (fl), *Letouzey 11801* (BR, K, P, WAG); South West Province, Mount Cameroon, Mabeta peninsula, Mabeta, 19 Oct 1997 (st), *Tchouto et al. 1756* (MO). **CENTRAL AFRICAN REPUBLIC.** Ndakan Gorilla Study Area, 02°21'N, 16°10'E, 385 m, 15 Jul 1988 (fr), *Fay & Harris 8535* (MO); Sangha-Mbaere, Ndakan Gorilla Study Area, 385 m, 25 Jul 1988 (fr), *Fay & Harris 8546* (MO); [Oubangui], Boukoko, 19 Aug 1947 (fl, fr), *Tisserant 138* (BM, BR, K, MO, P); 17 Sep 1947 (fl), *Tisserant 246* (BM, G, P); [Oubangui], Boukoko, 6 Oct 1947 (fl), *Tisserant 315* (BM, K, P); 15 Dec 1948 (fl), *Tisserant 1287* (BM, K, P); 7 Jun 1950 (fr), *Tisserant 1768* (BM, P); région de Boukoko, 28 Nov 1951 (fr), *Tisserant 2295* (BM, BR, P). **GABON.** Ogooué-Ivindo: Lopé-reserve, 0°30'S, 11°33'E, 28 Nov 1986 (st), *Reitsma & Reitsma 2622* (MO, NY, WAG); Reserve de la Lopé, 0°15'S, 11°40'E, 3 Oct 1992 (fr), *White 371* (MO); SEGC Lopé Reserve, 0°15'S, 11°40'E, 18 Jun 1991 (fr), *White 489* (MO).—Ogooué-Lolo: Bambidie, about 30 km E of Lastoursville, 300 m, ca. 0°44'S, 12°58'E, 13 Nov 1999 (fr), *Breteler 15468* (WAG); région de Lastoursville, Lastoursville, 25 Feb 1930 (fl), *Le Testu 7937* (BM, NY, P). **REPUBLIC OF THE CONGO.** Chaillu, ouest-Mossendjo, région de Kouyi, plaine ou steppe de Mussanda sur dalle cuirassée [02°57'S, 12°44'E], 23 Apr 1977 (fr), *Sita 4118* (BR, P, WAG). **DEMOCRATIC REPUBLIC OF THE CONGO.** Bas-Uele: Digba, Prov. Orientale, Terr. Ango, forêt des Akare entre riviére Bili et Asa, 29 Nov 1963 (fl), *Gerard 5583* (K, MO, WAG).—Équateur: Yalifake (Région Mondombe, Terr. Ikela) [0°37'S, 22°42'E], 6 May 1959 (fl), *Evrard 6266* (BR, K).—Ituri: Zone de Mambasa (Ituri Forest), Lenda, 1°19'N, 28°38'E, 750 m, 2 Jun 1994 (fr), *Ekuba 1615* (MO, WAG); Zone de Mambasa (Ituri), Epulu, 1°25'N, 28°35'E, 750 m, forêt mixte à 25 km nord-ouest d’Epulu, 26 Jul 1986 (fl), *Hart 572* (BR, MO); Zone de Mambasa (Ituri Forest), Afarama, 1°33'N, 28°32'E, 800 m, 27 Apr 1973 (fl), *Marabo 1546* (BR, K, MO).— Kongo Central: Prov. Léopoldville, Mbanza-Ngungu [“Territ. Thysville”], forêt de Kinganga, Oct 1958 (fl, fr), *Davio 10* (A, B, BR, P, WAG); Luki, 8 May 1947 (fr), *Donis 1441* (P); Route Interieure, Luki, 27 Jul 1957 (fr), *Matton 11* (BR); Luki, *Toussaint 2187* (K).— Kwilu: Kiyaka-Kwongo, Prov. Kinshasa, Terr. Kikwit, 6 Sep 1955 (fl), *Devred 2565* (K, WAG).—Maniema: Kivu Prov., Territ. Kindu, Beondo, 21 Apr 1959 (fr), *Bamps 597* (K).—Tshopo: Prov. Orientale, Terr. Tsangi, Yangambi, ca. 470 m, 14 Jan 1952 (fr), *Donis 3404* (K, MO); Yangambi, 470 m, 8 Aug 1936 (fl), *Louis 2393* (B, BM, K, MO, P, US), 15 Jan 1938 (st), *Louis 7522* (NY, PR, RSA), s. d. (fr), *Louis 16550* (BR, K). **ANGOLA.** Cabinda: Pango Manga, 22 Jan 1916 (fl), *Gossweiler 6185* (BM).—Cuanza Sul: Amboim, Capir, proximum flumen Carloango-Cuvo, 900 m, Sep 1932 (fr) *Gossweiler 9903* (K—2 sheets).

*Xylopiacupularis* is distinctive among African *Xylopia* species for its combination of small acuminate leaves with abaxial golden appressed pubescence, relatively long pedicels, sepals connate into a cup-shaped calyx, and the large number (up to 18) of monocarps, which are usually distinctly stipitate, and the circular seeds on which the raphe is distinctly keeled for part of its length. Some specimens of *X.phloiodora*, e.g. *Tisserant 955*, can resemble it, but may be distinguished by the strongly reticulate adaxial surface of the leaf, the persistent distal bract on the pedicel, the more rounded staminal cone, the smaller number of carpels, and the stigmas broader, warty, and lacking apical hairs.

While the long stipes on the monocarps are distinctive, there is variability in the character. Collections from the Central African Republic exemplify this. In the collections of Fay and Harris, the stipes are short and wide, i.e. 5 by 5 mm, while those in *Tisserant 2295* are long and slender (up to 24 mm long). On the specimen *Tisserant 1768*, the stipes are 5–8 mm long and 3.5–5 mm thick. In the case of specimens with short stipes on the monocarps, the tendency toward large numbers of monocarps per fruit is helpful in identification.

In many herbaria, collections of this species were filed under the *Xylopiachrysophylla*, a name based on a specimen from Yangambi in the Democratic Republic of the Congo. We could not distinguish either the type of *X.chrysophylla* or the type of *X.gilviflora* from *X.cupularis*, and follow Paiva (1966) in placing the newer names as taxonomic synonyms of *X.cupularis*.

In the protologue for *Xylopiacupularis*, Mildbraed mentions a second specimen, *Staudt 525*, but points out that the specimen is sterile and that it cannot be identified as this species with certainty. The specimen is therefore not regarded as having any type status with respect to this name.

### 29. Xylopia dinklagei

Taxon classificationPlantaeMagnolialesAnnonaceae

Engler & Diels, Notizbl. Königl. Bot. Gart. Berlin 2: 298. 1899.

D1542BE5-2A93-5255-A278-95519F467883

Fig. 39G–I

Xylopicrum dinklagei (Engler & Diels) Kuntze, Deutsch. Bot. Monatsschr. 21: 173–174. 1903. Type. LIBERIA [“Oberguinea”]. Grand Bassa County, Grand Bassa, 18 Oct 1896, *M. Dinklage 1760* (lectotype, here designated: B! [100249554]; isolectotype: A!; possible isolectotypes (see below): B! [100153126, 100153127]).

#### Description.

***Shrub*** or low climber. ***Twigs*** brown, pubescent, the hairs 0.1–0.3 mm long, eventually glabrate; ultimate branches often thickened at base. ***Leaf*** with larger blades 6–9.9 cm long, 1.8–3.3 cm wide, chartaceous to subcoriaceous, concolorous to slightly discolorous, oblong to elliptic, apex acuminate, the acumen 5–11 mm long, base broadly cuneate to rounded, pubescent on the midrib but otherwise glabrous adaxially, finely appressed-pubescent abaxially; midrib plane to slightly impressed adaxially, raised abaxially, secondary veins weakly brochidodromous, 10–11 per side, diverging at 60–70° from the midrib, plane or slightly raised and indistinct adaxially, slightly raised abaxially, higher-order veins indistinct or slightly raised on both surfaces; petiole 2–4.5 mm long, canaliculate, sparsely pubescent to glabrate. ***Inflorescences*** axillary, 1-flowered, pubescent; pedicels 2.3–3.3 mm long, 1.2–1.4 mm thick; bracts 3–4, imbricate, persistent, 1.1–1.8 mm long, broadly ovate to semicircular, apex obtuse to rounded; buds lanceolate, apex acute. ***Sepals*** slightly spreading at anthesis, 1/4–1/3-connate, 2.3–2.7 mm long, 2.9–3.3 mm wide, coriaceous, broadly triangular to orbicular, apex acute, sericeous abaxially. ***Petals*** pale yellow *in vivo*; outer petals possibly somewhat spreading at anthesis, 10.5–13 mm long, 2.5–3.4 mm wide at base, 1.6–2.0 mm wide at midpoint, coriaceous, narrowly lanceolate, apex obtuse, pubescent, but becoming glabrous in the center toward the base adaxially, sericeous abaxially; inner petals possibly erect or spreading at anthesis, 8.5–10.7 mm long, 2.4–2.7 mm wide at base, 0.8–1.2 mm wide at midpoint, coriaceous, linear-subulate, apex acute, base with undifferentiated margins, pubescent on both surfaces except for the glabrous base. ***Stamens*** ca. 120; fertile stamens 1.3–1.5 mm long, narrowly oblong, apex of connective 0.2–0.3 mm long, shieldlike, overhanging anther thecae, glabrous, anthers 9–10-locellate, filament ca. 0.4 mm long; outer staminodes 1.4–1.5 mm long, clavate, apex obtuse to truncate; inner staminodes 0.8–1.0 mm long, quadrate, apex obtuse to truncate; staminal cone 1.7–2.0 mm in diameter, 0.7–1.1 mm high, concealing the ovaries, rim laciniate. ***Carpels*** 12–15; ovaries 1.3–1.4 mm long, narrowly oblong, densely pubescent, stigmas connivent, 2.4–2.8 mm long, filiform, apex acute, with tuft of hairs at apex but otherwise glabrous. ***Torus*** flat, ca. 2.1 mm in diameter. ***Fruit*** of up to 13 glabrate monocarps borne on a pedicel 4.5–5 mm long, 3.2–5.6 mm thick, sparsely pubescent, sometimes with short dead branch attached and bracts and sepals persistent; torus 8–11 mm in diameter, 5–6.3 mm high, depressed-globose. ***Monocarps*** with green exterior *in vivo*, endocarp color unknown, 3.6–7.7 cm long, 1.2–1.5 cm wide, 0.9–1.1 cm thick, narrowly oblong and slightly falciform, torulose, apex with a curved beak 2–4 mm long or occasionally obtuse, base contracted into a flat stipe 6–8 mm long, 2–3 mm thick, longitudinally ridged or wrinkled, verrucose; pericarp ca. 0.4 mm thick. ***Seeds*** up to 10 per monocarp, commonly 5–7, in a single row, lying oblique to long axis, 10.5–11.5 mm long, 6.8–7.6 mm wide, 5–6 mm thick, flattened-ellipsoid, oblong to elliptic in cross-section, truncate at micropylar end, rounded at chalazal end, tan, smooth, faintly shiny, raphe/antiraphe not evident, micropylar scar 4.5–5 mm long, 2.5–3.5 mm wide, elliptic; sarcotesta unknown *in vivo*; aril absent.

**Figure 39.**
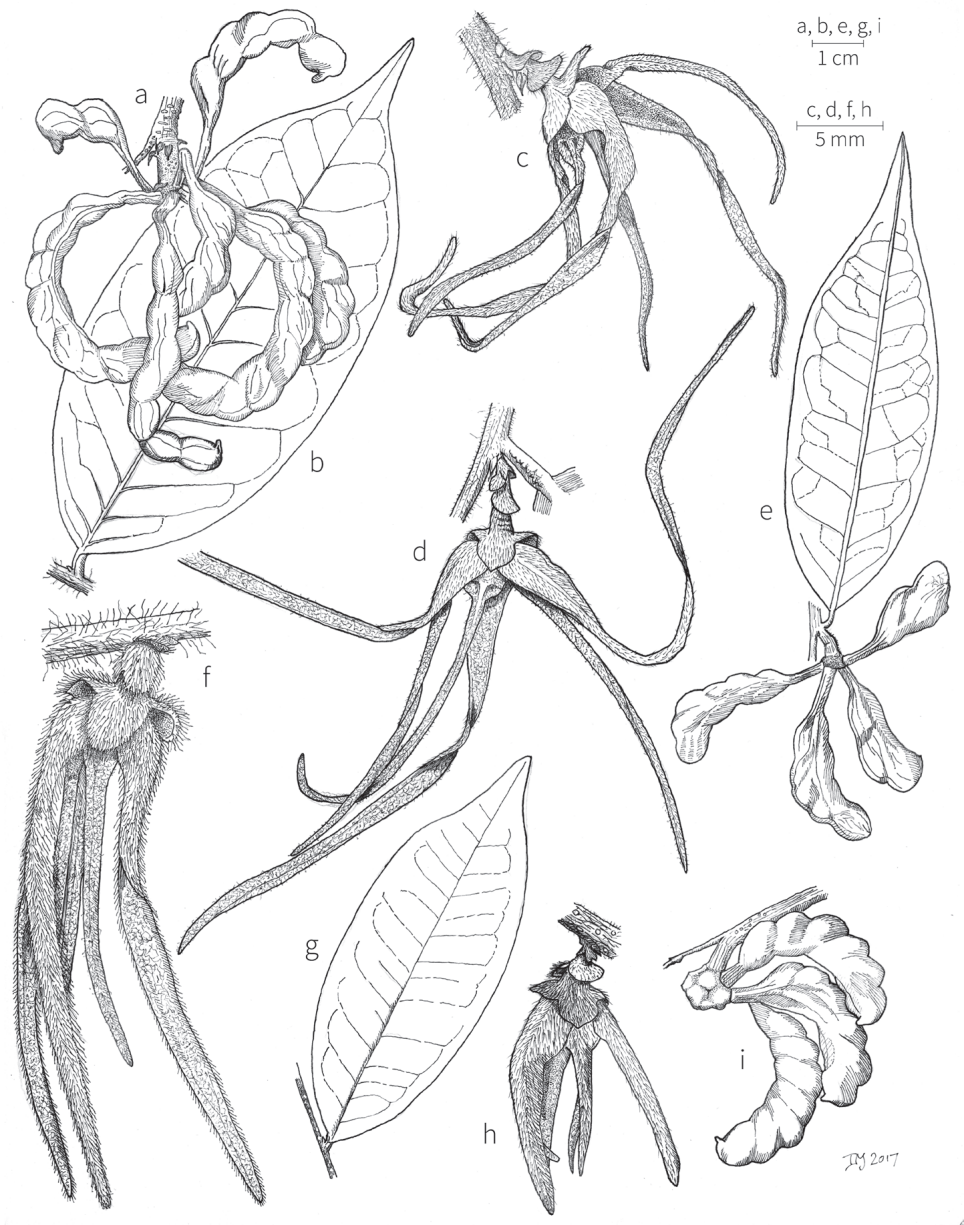
*Xylopiathomsonii*, *X.monticola*, *X.talbotii*, and *X.dinklagei*. **A–C***X.thomsonii***A** Fruit **B** Leaf **C** Flower **D–E***X.monticola***D** Flower **E** Leaf and fruit **F***X.talbotii*, flower, side view **G–I***X.dinklagei***G** Leaf **H** Flower **I** Fruit. A from *de Wilde & de Wilde-Duyfjes 2703* (K) **B, C** from *Talbot & Talbot 3267* (BM) **D** from *Chapman 3755* (K) **E** from *Thomas et al. 7400* (MO) **F** from *Thomas 3204* (MO) **G, H** from *Dinklage 1840* (A) **I** from *Baldwin 10805* (K).

#### Phenology.

Specimens with flowers have been collected in February, April, and May, and with fruits from August to December.

#### Distribution

(Fig. 34). Collected only along the coast of Liberia, in swamps near sea level.

#### Additional specimens examined.

**LIBERIA.** Buchanan, 23 Sep 1971 (fr), *Adam 26092* (MO); Grand Cape Mount Co., Mecca, 24 Dec 1947 (fr), *Baldwin 10805* (K); in fruticetis et ad margines silvarum prope Monrovia et alibi, 20 m, 17 May 1906, *Dinklage 1760* bis (B-100153125); Grand Bassa, *Dinklage 1840* (A, WU), Grand Bassa, s. d. (st), *Dinklage 1858* (A); Grand Bassa, 30 Apr 1898 (fl), *Dinklage 1873* (BM, G, P—3 sheets); Grand Bassa, 1 May 1898 (fl), *Dinklage either s. n. or 1898*—might be 1858 (K); 5 mi N of Bomi Hills, 18 Nov 1970 (fr), *Jansen 2255* (WAG); SW Monrovia, 13 Aug 1909 (fr), *Massey 49/27* (NY); Monserrado, Firestone Plantation, division 18, ca. 6°24'N, 10°19'W, 11 Feb 1970 (fl), *Stoop-v. d. Kasteele, F. S. C. 140* (WAG); without definite locality, s. d. (fr), *Straub 290* (US).

*Xylopiadinklagei* is a shrub or low climber of swampy habitats, with a fine twig pubescence made up exclusively of appressed hairs 0.1–0.3 mm long. The leaf base is often rounded or less often broadly cuneate, and the indument on the abaxial surface of the leaf is not readily visible but is dense, fine, and appressed. The outer petals are only 10.5–13 mm long. The monocarps have a single row of seeds oriented obliquely to the long axis of the monocarp, and the monocarps themselves are usually distinctly torulose, with a thin pericarp, only ca. 0.4 mm thick. The stipe of the monocarp is 6–8 mm long and 2–3 mm thick, and the seeds are oblong to elliptic in cross section. *Xylopiadinklagei* is superficially similar to *X.acutiflora* and the species overlap slightly in distribution. *Xylopiaacutiflora*, however, is an upland forest tree reaching at least 15 m in height, with coarser twig pubescence including erect hairs over 1 mm long. Its leaves are cuneate to broadly cuneate at the base, with sparser but longer hairs abaxially. The outer petals are 19.6–37 mm long. The monocarps have two rows of seeds oriented perpendicular to the long axis of the monocarp, and the monocarps are oblong and at most weakly torulose, with a fibrous pericarp ca. 1 mm thick. The stipe of the monocarp is proportionally shorter and thicker than in *X.dinklagei*, 3–7 mm long and 2.9–4.5 mm thick, and the seeds are wedge-shaped in cross-section.

As in a related climbing species, *Xylopiapiratae*, the ultimate branches on specimens of *X.dinklagei* are often short (4–7 nodes with ca. 1–3 cm long internodes) and emerge at right angles, with the bases thickened just above where they join the supporting branch.

Engler and Diels based the name *Xylopiadinklagei* on two Dinklage collections, n. 1760 and n. 1858. The sheets of *Dinklage 1760* at B, however, bear different information. One of them, B-100153125, was collected in 1906 from a different locality and therefore cannot be type material. Of the remaining sheets, the two with flower buds (B-100153126 and B-100153127) have labels with the type locality printed on them, but no date information. The collection with mature fruits (B-100249554) is dated 18 October 1896 and is chosen as lectotype. Of the second syntype, *Dinklage 1858*, only a sheet at A has been seen; this specimen was identified as *X.oxypetala* by Engler and Diels (1901) but it is more similar to the specimens separated here as *X.dinklagei*.

### 30. Xylopia elliotii

Taxon classificationPlantaeMagnolialesAnnonaceae

Engler & Diels, Monogr. afrik. Pflanzen-Fam. 6: 65. 1901.

4DDB619E-3DEE-564C-BDAC-BE6675E6DB01

Fig. 40

#### Type.

GUINEA [“Sierra Leone”]. Farana Region, riverside woods of Niger, Farana, 26 Mar 1892 (fl), *G. F. Scott-Elliot 5325* (lectotype, here designated: B! [100153142]; isolectotypes: BM! [000510796, lower half of sheet], GH! K! [000199071], P! [00169156]).

#### Description.

***Shrub or small tree*** up to 10 (–18) m tall, much-branched; bark gray or dark brown. ***Twigs*** reddish brown to dark gray, eventually light gray, densely erect-hairy, the hairs 0.2–0.8 mm long, on new flushes of growth (bud scales still visible at base), eventually glabrate, sometimes with bark exfoliating; nodes commonly with two axillary branches. ***Leaf*** with larger blades 4.5–9.5 cm long, 1.8–5.6 cm wide, subcoriaceous, discolorous, lanceolate-ovate to elliptic, oblong, or oblong-lanceolate, apex obtuse, attenuate, emarginate, or acute, occasionally with a short acumen 4 mm long, base cuneate to rounded, short-decurrent on petiole, glabrous except for the pubescent midrib adaxially, finely appressed-pubescent to glabrate abaxially; midrib raised or impressed adaxially, raised abaxially, secondary veins indistinctly brochidodromous, 10–16 per side, diverging at 45–70° from the midrib, these and higher-order veins slightly raised on both surfaces; petiole 3.5–6 mm long, shallowly canaliculate, pubescent. ***Inflorescences*** axillary, 1(–2)-flowered, pubescent; pedicels not pedunculate, 3.0–6.1 mm long, 0.9–1.1 mm thick; bracts 2, one to either side of pedicel midpoint, caducous or persistent, 1.6–2.6 mm long, ovate to semicircular, apex acute to obtuse; buds linear-lanceolate, sometimes falciform, apex acute. ***Sepals*** slightly spreading at anthesis, 1/4–1/2-connate, 2.3–4.5 mm long, 2.5–3.6 mm wide, coriaceous, ovate to broadly ovate, apex acute or apiculate at the apex, densely sericeous abaxially. ***Petals*** white, tinged with purple at the base *in vivo*; outer petals spreading at anthesis, (12.6–) 19–32 mm long, 2.3–4 mm wide at base, 1.2–2.5 mm wide at midpoint, fleshy, linear, apex obtuse, densely puberulent adaxially, densely appressed-pubescent abaxially; inner petals bent outward from the base at anthesis, 16–24 mm long, 2.3–3.9 mm wide at base, 0.9–1.4 mm wide at midpoint, fleshy, linear, apex acute, base with undifferentiated margin, pubescent on both surfaces, becoming glabrous at base. ***Stamens*** ca. 120; fertile stamens 1.1–1.6 mm long, narrowly oblong-clavate, apex of connective 0.2–0.3 mm long, depressed-globose to shieldlike, overhanging the anther thecae, minutely papillate or glabrous, anthers 10–13-locellate, filament 0.2–0.5 mm long; outer staminodes 1.3–1.7 mm long, oblong to clavate, apex acute, obtuse or truncate; inner staminodes 0.8–1.1 mm long, oblong, apex truncate, occasionally innermost stamens reduced in size but still with a few anther locelli so not truly staminodial; staminal cone 1.4–1.8 mm in diameter, 0.6–1.3 mm high, completely concealing the ovaries, rim irregularly laciniate. ***Carpels*** 9–10; ovaries 1.3–1.5 mm long, linear-oblong, pubescent, stigmas connivent, 1.3–2.3 mm long, trowel-shaped, apex obtuse, glabrous or pubescent at the apex. ***Torus*** flat, 1.9–2.5 mm in diameter. ***Fruit*** of up to 8 sparsely pubescent to glabrate monocarps borne on a pedicel 10–12 mm long, 1.3–6 mm thick, glabrate; torus 2.5–16 mm in diameter, 2.5–4 mm high, depressed-globose. ***Monocarps*** with red- to purple-tinged green exterior and red endocarp *in vivo*, 2.0–3.8 cm long, 0.9–1.3 cm wide, 1.1–1.2 cm thick, irregularly oblong or obovoid, slightly torulose, apex rounded, base sessile or contracted into a stipe 3–6 mm long, 2–3 mm thick, slightly wrinkled, finely verrucose and somewhat shiny; pericarp 0.8–0.9 mm thick. ***Seeds*** up to 9 per monocarp, in two rows, lying oblique to perpendicular to long axis, 9.2–11.8 mm long, 6.8–7.8 mm wide, 5.3–6.8 mm thick, oblong to flattened-ellipsoid, elliptic to semicircular in cross-section, truncate at micropylar end, rounded at chalazal end, light brown, smooth, dull, raphe/antiraphe not evident, micropylar scar 3.5–4 mm long, 2–2.8 mm wide, broadly elliptic to obovate; sarcotesta unknown *in vivo*; aril absent.

**Figure 40.**
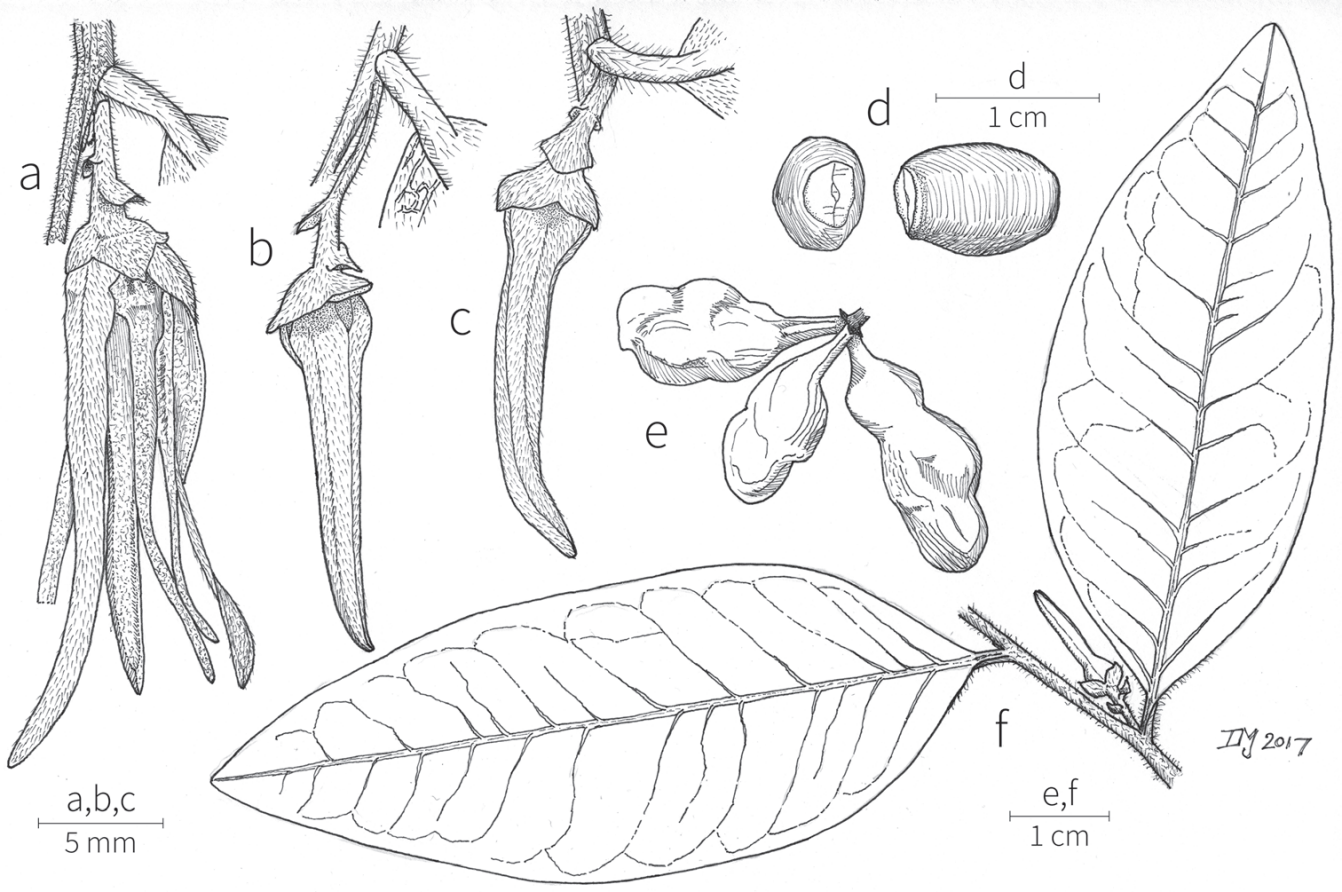
*Xylopiaelliotii*. **A** Flower, side view, of specimen from Guinea **B** Flower bud, side view, of specimen from Central African Republic **C** Flower bud, side view, of isolectotype specimen from Sierra Leone **D** Seed, view of micropylar end and side view **E** Fruit **F** Habit. **A** from *Adam 11846* (MO) **B** from *Le Testu 3787* (P) **C** from *Scott-Elliot 5325* (GH) **D** from *Pobéguin 1529* (P) **E** from *Scott-Elliot 5328* (GH) **F** from *Scott-Elliot 5288* (BM).

#### Phenology.

Specimens with flowers have been collected from January to May, and with fruit from October to March and in May.

#### Distribution

**Figure 41.**
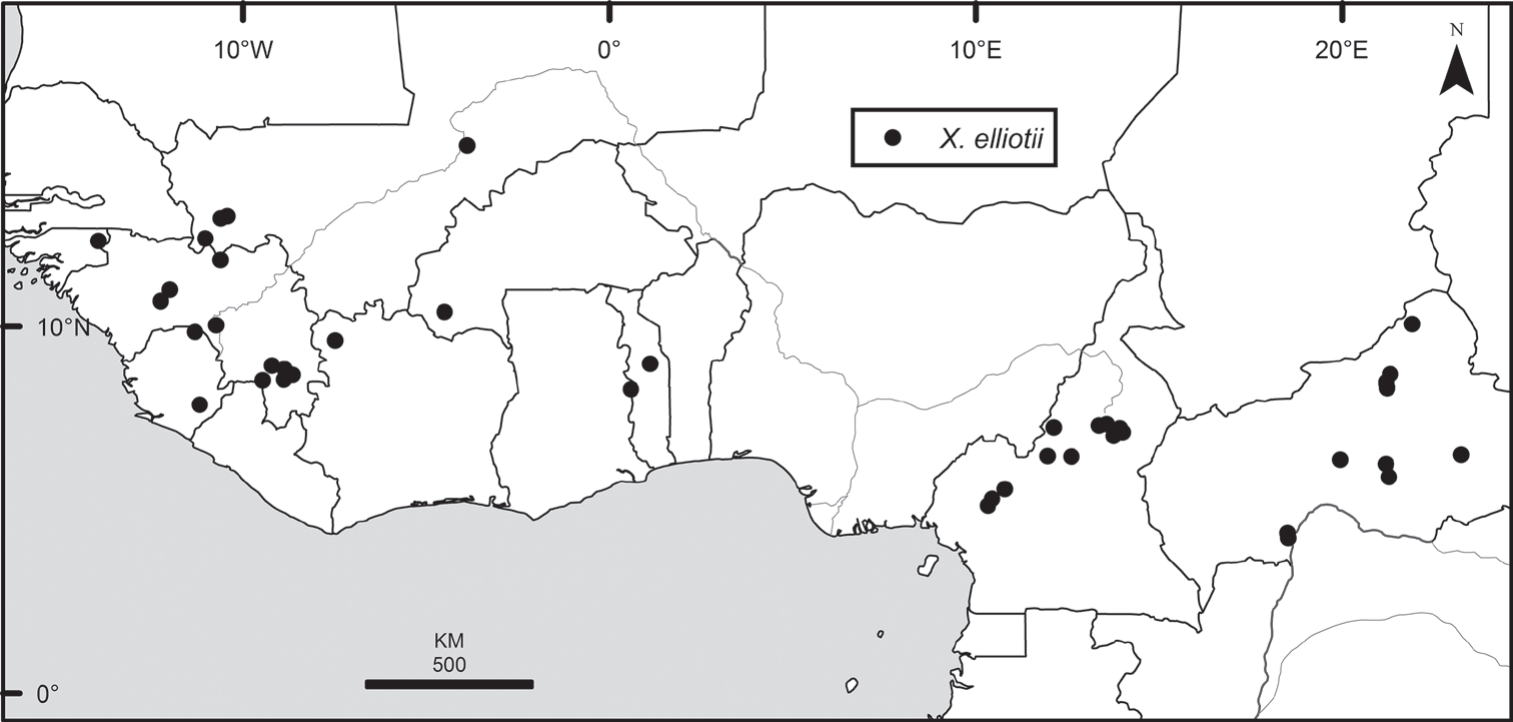
Distribution of *Xylopiaelliotii*. Bolder lines represent country borders, fainter lines lakes and major rivers.

(Fig. 41). Occurs from northeastern Sierra Leone eastward to Togo, then disjunct to central Cameroon and the Central African Republic, in gallery forest along streams and rivers and occasionally extending into drier uplands, at elevations of 280–1400 m.

#### Local names.

nkankalan jé (Malinké, *Duvall 271*), ké (*Westphal & Westphal-Stevels 10047, 10048, 10049, 10172*), kenema (Nongowa, *Jordan 2063*).

#### Representative specimens.

**GUINEA BISSAU.** Gabú, Pitche, Cambore (Fondo de Cambore), 7 Dec 1955 (yg fr), *Explorações Botânicas 3780* (B, FI-T, K, MO, WAG). **GUINEA.** Beyla, Moribadougou, Apr 1945 (fl), *Adam 123* (K, MO, P); Macenta, Tènèmadou, 10 Aug 1949 (st), *Adam 5917* (MO); Dalaba, Dalaba, 9 Apr 1956 (fl), *Adam 11846* (MO); Chaine de Tibè, 1943 (fr), *Adam 26905* (P); Macenta+Beyla Prefectures, Simandou Range, N of Pic de Fon, near the pass on path between villages Moribadou and Lamadou, 8°35'23"N, 8°53'52"W, 1040 m, 27 Mar 2008 (fr), *van der Burgt 1167* (K); Fouta Djallon, 23 Apr 1907 (fl), *Caille 18149* (P); Macenta+Beyla Prefectures, Simandou Range, Monts Simandou, Pic Dabatini, 8°33'16"N, 8°53'12"W, 1008 m, 23 Mar 2008 (fl), *Haba 180* (K); environs de Pita, Jan 1936 (fr), *Jacques-Félix 735* (P); Timbo, Mar 1907 (fl, fr), *Pobéguin 1529* (P); Lorbé à Pita, May 1909 (fl), *Pobéguin 2130* (P); in woods by Niger River, Farana, 26 Mar 1892 (fr), *Scott-Elliot 5328* (GH, K); Beyla Prefecture, Tibé Mont., collines SE du Pic de Tibé, en face du village Sondou, 910 m, 8°50'27"N, 8°52'16"W, 10 Dec 2007 (fr), *Traoré 35* (K). **SIERRA LEONE.** Nongawa, Kambui Forest Reserve, Neaboi Valley, 15 Apr 1955 (bud, fl), *Jordan 2063* (K, P); Sulimania Road, Falaba Marsh, 24 Mar 1892 (fl), *Scott-Elliot 5288* (B, GH, K, P). **MALI.** 32.8 km SW of Manantali, Arrondissement de Bamafélé, Cercle de Bafoulabé, Région de Kayes, 12°57.009'N, 10°36.613'W, 280-320 m, 12 Nov 1999 (fr), *Duvall 371* (MO—4 sheets); near the village of Solo, northern edge of Korofing National Park (formerly Bafing Faunal Reserve), 13°00.362'N, 10°26.137'W, 29 Jan 2004 (st), *Duvall 513* (MO); Pilimili Riv. Konnoa, s. d. (fl), *Vuillet 68* (P). **BURKINA FASO.** Tourny (Cascade) (SW Haute Volta), 1 Mar 1971 (fr), *Buonounou Ouétien 35* (P). **IVORY COAST.** Entre Odienné et Sangouani, 1 Mar 1973 (fl), *Aké Assi 11969* (K). **GHANA.** Shiare, Buem-Krachi Dist., 2500’ alt., 18 Apr 1959 (fl), *Hall 1450* (K). **TOGO.** Sakoda, Apr 1905 (fl, old fr ped), *Kersting I 84.a* (A); without definite locality or date (fl), *Kersting A.567* (GH, PH). **CAMEROON.** W. Cameroons, Ndop Plain, road to French Cameroons, 3800 ft, ca. 6°N, 10°30'E, 30 Mar 1962 (fl), *Brunt 261A* (K); Mbalang, 16 km E Ngdéré, 27 Jan 1978 (fl), *Fotius 2984* (P); Tchal Mbabo, 16 Mar 1978 (fl), *Fotius 3108* (P); Bayangam, Jan 1939 (fr), *Jacques-Félix 2965* (P); Koutchamkap, Feb 1939 (fl), *Jacques-Félix 3039* (P); Adamoua oriental, Hosséré Sillé, 20 Oct 1967 (fr), *Jacques-Félix 8724* (P); Tibati, 10 Nov 1967 (fr), *Jacques-Félix 9110* (P); Sadolkoulay (36 km E Ngaoundéré), 5 Dec 1964 (fr), *Raynal & Raynal 12228* (P—2 sheets); a 20 km E de Foumbot, 5°34'N, 10°48'E, 1100 m, 26 Oct 1974 (fr), *Sabatie 15* (MO, P); Près Katil-Foulbe, 50 km SE Ngaoundéré, 20 Oct 1983 (fr), *Sabatie 687* (P); Bayangam, 17 May 1978 (fr), *Westphal & Westphal-Stevels 10047* (P, WAG—2 sheets); Chutes de Tello, 45 km E de Ngaoundéré, 7°14'N, 13°57'E, 1250 m, 9 Apr 1983 (fl), *van der Zon 2105* (WAG). **CENTRAL AFRICAN REPUBLIC.** Oubangui, Reg Zango, Jan 1920 (fl), *Allouette s. n.* (L); Chari Oriental (Pays dou Snoussi), [Voyage au Mamoun] Golo près Noellé, 7–28 Mar 1903 (fl), *Chevalier 7741* (G, P—2 sheets); Manovo-Gounda-St. Floris National Park, 5 km S of Camp Koumbala along the Koumbala River (8°27'N, 21°13'E), 600 m, 3 Feb 1983 (fr), *Fay 4191B* (MO); Manovo-Gounda-St. Floris National Park, Koumbala River at Camp Koumbala, 08°29'N, 21°13'E, 590 m, 30 Mar 1983 (fr), *Fay 4351* (K), 30 Mar 1983 (fl), *Fay 4359* (K); Manovo-Gounda-St. Floris National Park, 9.3 km S of Koumbala Pende confluence on Pende Creek, 8°21'N, 21°14'E, 610 m, 15 May 1984 (fl), *Fay 6641* (MO); région de Yalinga, Haut Oubangui, entre Wanda-Djalé et Wadda, 26 Feb 1922 (fl), *Le Testu 3787* (BM, P, US); région de la Ouaka, région Ippy, Riv. Monga, 35 km NW Moronbas, 18 Feb 1925 (fl), *Tisserant 1778* (BM, P).

*Xylopiaelliotii* is more widespread than has been previously understood but has a number of characteristics consistent across its distribution: it is a small tree of gallery forest, the twigs have a dense indument of erect reddish brown hairs 0.2–0.8 mm long, the abaxial surface of the leaf blade has fine appressed hairs creating a dull surface to the leaf, the pedicels are somewhat elongate and always bear two bracts, one to either side of the pedicel midpoint (Fig. 40A–C), and the monocarps are sessile to short-stipitate with seeds usually in two rows and with a thin pericarp.

The species is largely restricted to the Sudanian biogeographic region (Linder et al. 2012) where few other species of *Xylopia* occur, but it is not an Upper Guinea endemic as indicated in Holmgren et al. (2004). There is a large disjunction in the distribution between Togo and Cameroon, and specimens from the eastern portion of the range tend to have slightly larger leaves and slightly longer pedicels and outer petals.

The collection *McPherson 21337* (MO) from the Nimba Mountains of Guinea is problematic. The leaves and fruit resemble those of other specimens of *X.elliotii* from Guinea, but the habit, a tree 25 m tall, and the habitat, a forested slope at 860 m, are unusual for the species. The seeds were described as orange. A specimen identified as *X.acutiflora* in Robson (1960), *Holmes H.1273* (K), from the Mwinilunga region of Zambia, also resembles *X.elliotii* in its reddish-brown erect twig pubescence and relatively long pedicels with only two bracts. The leaves on the specimen, however, are smaller than those of *X.elliotii*, the tree is described as occurring in *mushitu* swamp forest, and the specimen is widely disjunct from the range of *X.elliotii*.

There is little ecological information for *Xylopiaelliotii*. The collections of Fay from Manovo-Gounda-St. Floris National Park, Central African Republic list the following associates in gallery or gallery-associated swamp forests: *Adinamicrocephala*, *Gardeniaimperialis*, *Ixorabrachypoda*, *Ourateaflava*, *Rhynchosporacorymbosa*, *Syzygiumguineense*, and *Uapacatogoensis*, as well as species of *Ancistrophyllum*, *Berlinia*, *Cyrtosperma*, *Gaertnera*, *Mitragyna*, and *Raphia*. The fragrance of the flowers was noted by several collectors. Two flowers on the collection *Fay 6641* (MO) are distorted by galls, similar to those seen in flowers of *X.mwasumbii*.

Collections made by Westphal and Westphal-Stevels from the area of Bayangam, Cameroon, in 1978 document that the plant is locally kept around houses and the fruits used as a condiment. A collection by Jacques-Félix from the same locality made in 1939 notes that the plant is “cultivé” suggesting a long-standing local use for the fruits of this species.

The protologue for the name *Xylopiaelliotii* Engl. & Diels gives the type locality as Sierra Leone, but Gledhill (1969) has documented that the type locality, Farana on the Niger River, is actually in present-day Guinea.

### 31. Xylopia hypolampra

Taxon classificationPlantaeMagnolialesAnnonaceae

Mildbraed, Notizbl. Königl. Bot. Gart. Berlin, Append. 27: 18. [11 Oct] 1913.

B4C86B1F-9B7F-5C08-A905-39A299C14E2B

Figs 4G , 42E–F

Xylopia brieyi De Wildeman, Bull. Jard. Bot. État 4: 385. 1914. Type. DEMOCRATIC REPUBLIC OF THE CONGO [“Belgian Congo”]. Kongo Central Province, Ganda-Sundi, 8 Oct 1911, *J. de Briey 108* (lectotype, here designated: BR! [0000008824844]; isotypes: BR! [0000008824790, 0000008824806, 0000008824813, 0000008824837], US! [1270066]).

#### Type.

CAMEROON. East Region, zwischen Station Lomie, Bidjum und dem Dscha-Posten, 600–700 m, 13 May 1911, *J. Mildbraed 5183* (holotype: B!; isotype: HBG! [502479]).

#### Description.

***Tree*** up to 43 m tall, d.b.h. up to 80 cm, bole round, rising to small open crown of horizontal branches; bark brown-gray, smooth, very finely cracked in all directions. ***Twigs*** brown, eventually grayish black, tomentellous, the hairs 0.2–0.4 mm long, eventually glabrate; no nodes seen with two axillary branches. ***Leaf*** with larger blades 5.7–7.5 cm long, 1.4–1.9 cm wide, subcoriaceous, concolorous, lanceolate, apex attenuate or rarely acuminate, the acumen ca. 12 mm long, and minutely retuse or obtuse, base obliquely cuneate, rarely rounded or somewhat angular, glabrous except for the pubescent midrib adaxially, densely golden- or silvery-sericeous abaxially; midrib plane adaxially, raised abaxially, secondary veins indistinctly brochidodromous, 10–18 per side, diverging at 50–70° from the midrib, these and higher-order veins indistinct adaxially, obscured by indument abaxially; petiole 4–5.5 mm long, semi-terete, pubescent. ***Inflorescences*** axillary, 1–3-flowered, pubescent; peduncle absent; pedicels superposed in leaf axil, 2.2–3.8 mm long, 1.0–1.3 mm thick; bracts 3–4, evenly spaced and somewhat imbricate, persistent, 1.8–2.3 mm long, ovate to broadly ovate, apex obtuse to rounded; buds linear, often falciform, apex acute, sometimes uncinate. ***Sepals*** slightly spreading at anthesis, 1/3-connate, 2.1–3.2 mm long, 2–2.5 mm wide, coriaceous, broadly triangular, apex acute, pubescent abaxially. ***Petals*** pale green to yellow *in vivo*; outer petals slightly spreading at anthesis, 25–28.7 mm long, 2.5–2.6 mm wide at base, 1.0–1.1 mm wide at midpoint, coriaceous, filiform, apex obtuse and slightly incurved, densely puberulent except for glabrous basal concavity adaxially, sericeous except for a glabrous patch at base abaxially; inner petals more or less erect at anthesis, 16–31 mm long, 1.9–2.5 mm wide at base, 0.6–1.0 mm wide at midpoint, coriaceous, filiform, apex acute, base with undifferentiated margin, longitudinally ridged and puberulent on both surfaces. ***Stamens*** ca. 100; fertile stamens 1.0–1.5 mm long, narrowly oblong, apex of connective purplish red *in vivo*, ca. 0.2 mm long, shieldlike, overhanging anther thecae, minutely papillate, anthers 7–8-locellate, filament 0.4–0.6 mm long; outer staminodes 1.3–1.7 mm long, oblong, apex obtuse to truncate; inner staminodes ca. 1.1 mm long, oblong, apex rounded to truncate; staminal cone 1.3–1.7 mm in diameter, 0.6–0.7 mm high, completely concealing the ovaries, rim laciniate. ***Carpels*** 7–12; ovaries 0.7–0.8 mm long, oblong, pubescent, stigmas connivent or sometimes free at the very tips, 1.6–2.5 mm long, filiform, with a tuft of hairs at the apices. ***Torus*** flat, slightly concave under the ovaries, 1.5–2.2 mm in diameter. ***Fruit*** of up to 8 glabrate monocarps borne on a pedicel 2.5–6 mm long, 3–4.8 mm thick, with the bracts persistent, glabrate; torus 4.5–12 mm in diameter, 3.5–7.5 mm high, depressed-globose. ***Monocarps*** with a greenish brown exterior flecked with pale brown lenticels, sometimes tinged with cinnamon or red, and pink-red endocarp *in vivo*, 2.6–4.1 cm long, 1.1–2.2 cm wide, 1.5–2.1 cm thick, obovoid to oblong or ellipsoid, rarely globose, not torulose, apex rounded, sessile and rounded to truncate at base, often marked by longitudinal ridges but somewhat sunken between them so that the cross-section is bluntly angled, densely lenticellate; pericarp 0.5–1.4 mm thick, fibrous, woody. ***Seeds*** up to 10 per monocarp, in two rows, lying perpendicular to long axis, 7.1–10.6 mm long, 6.3–7.9 mm wide, 4.2–5.7 mm thick, elliptic, ovate, oblong or nearly circular, wedge-shaped to semicircular in cross section, truncate at micropylar end but micropylar scar bent to the side, rounded at chalazal end, light reddish brown, smooth, dull, raphe/antiraphe not evident, micropylar scar 1.3–4 mm long, 1.4–2 mm wide, ovate to circular; sarcotesta greenish white *in vivo*; aril absent.

**Figure 42.**
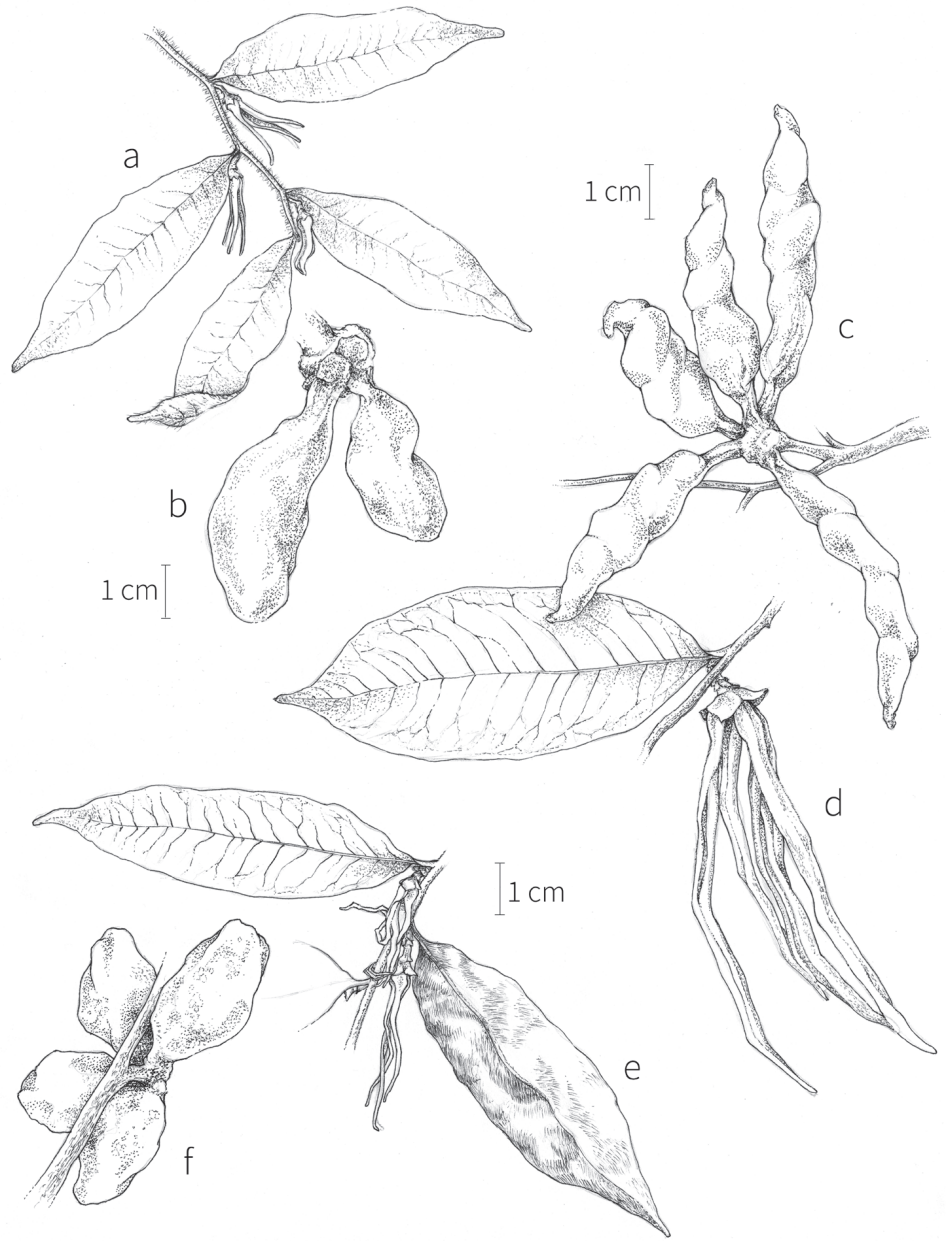
*Xylopiapynaertii*, *X.mildbraedii*, and *X.hypolampra*. **A, B***X.pynaertii***A** Habit **B** Fruit **C, D***X.mildbraedii***C** Fruit **D** Habit **E, F***X.hypolampra***E** Habit **F** Fruit. **A** from *McPherson 13825* (MO) **B** from *Letouzey 12317* (P) **C** from *Breteler et al. 12632* (WAG) **D** from *Reitsma & Reitsma 2816* (NY) **E** from *Le Testu 2023* (F) **F** from *Le Testu 8094* (BM).

#### Phenology.

Collections with flowers have been obtained from November to June, and those with fruits in all months of the year except September; in Lopé National Park, Gabon, a peak of flowering was observed in October and November, followed by production of fruits from June to August (E. Bush and K. Abernethy, personal communication).

#### Distribution

**Figure 43.**
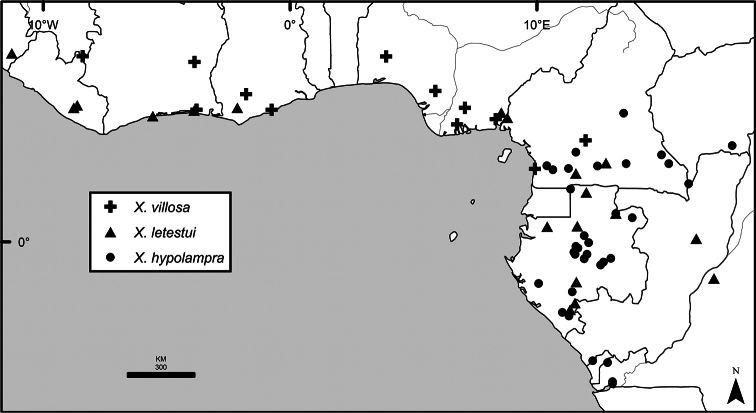
Distributions of *Xylopiavillosa*, *X.letestui*, and *X.hypolampra*. Bolder lines represent country borders, fainter lines lakes and major rivers.

(Fig. 43). Occurs from central Cameroon east to southwestern Central African Republic and south to the Cabinda Province of Angola and southwestern Democratic Republic of the Congo, where it grows in evergreen or semi-deciduous forest, sometimes along forest edges, and in gallery forest, at elevations of 400–900 m. In the northern Republic of the Congo, seedlings are reported as growing on roadsides (congotrees.rbge.org.uk: Royal Botanic Garden Edinburgh); the report is probably reliable, although we have seen no *X.hypolampra* specimens from the Republic of the Congo.

#### Local names.

Abiès (Bulu, *de Wilde 7963*), canzi (Bayaka, *LeTestu 1749*), ekui (reported as timber trade name, ITTO), lucanga (*Murta 43*), lukanga ivembuka (lukanga jaune) (*Sargos 141*), molo-nzange (Lissongo, *Tisserant 1246*, *1385*), ndong-eli np (+ Fang, *Wilks WIL 1040*), nyanghogha (Mitsogo, *Wilks WIL 1040*), odjobi (*Foury 101*), sangi (Babindjere, *Harris & Fay 222*).

#### Additional specimens examined.

**CAMEROON.** Bitya near River Ja, s. d. (fl), *Bates 1757* (K); South Province, Department Ocean, Mvie, about 11 km (along a straight line) ENE of village Mvie, 12 km N of Akom II by road, 2°55'N, 10°39'E, 400 m, 28 Jan 1998 (fl), *van der Burgt & Laan 364* (K, MO, WAG); Central Division, Gazette: Ndanan 1, near Mefou town, 3°37'29"N, 11°34'56"E, alt. 710 m, 8 Mar 2004 (fr), *Cheek 11487* (K); without definite locality, 1935 (fl), *Foury 101* (OWU, P); a 30 km au NE de Bange (km 75 route Yokadouma-Moloundou), 25 May 1963 (fr), *Letouzey 5139* (P); Bipindi–Ebolowa, Aug or Dec 1913, *Mildbraed 7618* (B, K); Dengdeng, Sommer 1914 (buds), *Mildbraed 8827* (BM, K—4 sheets); 16 km on the road from Ebolowa to Minkok, 2°58'N, 11°17'E, 670 m, 6 Feb 1975 (fl), *de Wilde 7963* (B, BR, K, M, NY, P, U, WAG). **CENTRAL AFRICAN REPUBLIC**. Sangha Economique Prefecture, Ndakan, gorilla study area, 02°21'N, 16°09'E, 350 m, 13 Feb 1988 (fr only), *Harris & Fay 222* (MO); Sangha Economique Prefecture, Ndakan, gorilla study area, 02°21'N, 16°09'E, 350 m, 27 Feb 1988 (fr), *Harris & Fay 266* (MO); Sangha Economique Prefecture, Ndakan, gorilla study area, 02°21'N, 16°09'E, 350 m, 21 Mar 1988 (fr), *Harris & Fay 319* (MO); Boukoko, 20 Nov 1948 (fl), *Tisserant 1246* (K, P); 23 Feb 1949 (fr), *Tisserant 1385* [fruit of *Tisserant 1246*] (P). **GABON.** Ngounié: St. Martin – Munungu, July 1939 (fl, fr), *Walker s. n.* or *3454* (BM, K, MO, P); 6 km SSE du village Bilengui, 2°01'S, 11°26'E, 5 Feb 1985 (buds), *Wilks WIL 1040* (WAG).—Nyanga: Mayombe Bayaka, région du Nyanga, Inganga, 20 May 1914 (fl), *Le Testu 1749* (BM, K, MO, P); Tchibanga, 21 Feb 1919 (fl), *Le Testu 2023* (BM, F, MO–-2 sheets, P).—Ogooué-Ivindo: forêt des Abeilles, 40 km SSW of confluence of Ogooué-Ivindo, 00°30'S, 12°02'E, 5 Aug 1993 (fl, fr), *Dibata 1171* (K—2 sheets); Bélinga, Mines de Fer, 700–900 m, 4 Jun 1966 (fr), *Hallé 3736* (P); Réserve de la Lopé, au sud d’Ayem, chantier SOFORGA, 0°25'S, 11°30'E, 13 Mar 1989 (st), *McPherson 13763* (F, MO, NY); ca. 25 km ENE of Booué, 0°02'S, 12°06'E, 18 May 1987 (fr), *Reitsma et al.* (MO, NY, RSA); Reserve de Lopé-Okanda, west-central region of tourist zone, top of “Point of View” hill, 00°10'47"S, 11°34'48"E, 363 m, 3 Nov 2000 (fr), *Stone et al. 3171* (MO); MPAM/Lopé River, Lopé Reserve, 0°15'S, 11°40'E, 16 May 1991 (fl), *White LJTW 0483* (MO); road Mékambo to Makokou, 4 km W of Mbela-Baya, 0°58.5'N, 13°52'E, 500 m, 31 Dec 2000 (fl, fr), *Wieringa et al. 3748* (WAG).—Ogooué-Lolo: About 30 km E of Lastoursville, 0°40'S, 13°00'E, 19 Nov 1991 (fl, fr), *Breteler & Jongkind 10590* (MO, WAG); about 20–40 km NNE of Koumémayong, 0°15'N, 11°55'E, 13 Apr 1988 (fl, fr), *Breteler et al. 8669* (WAG); Makande surroundings, about 65 km SSW of Booué, ca. 0°41'S, 11°55'E, 21 Jan 1999 (fr), *Breteler et al. 14716* (WAG); région de Lastoursville, 1929–1931 (fl), *Le Testu 8094* (BM, BR, K—2 sheets, NY, P, US); km 16 E-most road on chantier 19 branching off 21 km from Lastoursville direction Koula-Moutu, 0°56'S, 12°35'E, 23 Nov 1988 (fr), *van der Maesen 5790* (MO, WAG).—Woleu-Ntem: without definite locality, May (fr), *Louis et al. 3419* (MO).—Province unknown: Kouilou infr., *Sargos 141* (P). **DEMOCRATIC REPUBLIC OF THE CONGO.** Kongo Central: Luki, 10 Dec 1947 (fl), *Donis 1617* (K); INEAC, Luki, Mayumbe Prov. Kinshasa, Terr. Lukula, 27 Mar 1959 (st), *Mahieu 292* (WAG), 27 Marh 1959 (fl, fr) *Mahieu 293* (WAG); Luki, vallée de la Minkudu à flanc de esteau, 27 Jun 1927 (fl, fr), *Toussaint 2395* (K, MO, P, US, WAG); Leopoldville Prov., Boma Territory, Luki, 7 Mar 1955 (fr), *Wagemans 956* (K). **ANGOLA.** Mayombe, Oct–Nov 1921 (st), *Dawe 267* (K); Buco Zau, 28 Jul 1916 (fr), *Gossweiler 6532* (BM); Mayumbe, Buco Zau, Dec 1916 (fr), *Gossweiler 7225* (BM); Cabinda-Chiaca, na Estrada para Buco Zau, 15 Mar 1959 (fl), *Murta 43* (BM).

*Xylopiahypolampra*, with its distinctive narrow coriaceous abaxially sericeous leaves, is readily separable from other African *Xylopia* species. The pedicels are always short, such that the flowers and fruits appear sessile in the leaf axils. The outer surface of the monocarps at maturity is brown and strongly marked with lenticels, and typically splits into three segments instead of the usual two. The monocarps are often longitudinally ridged and bluntly angled when dry.

*Xylopiahypolampra* becomes a slender small-crowned canopy tree in forests across its distribution and appears to be locally common. Associates mentioned by collectors include “Sterculiaceae” (two separate collections from Cameroon and Central African Republic), *Megaphryniummacrostachyum*, *Piptadeniastrumafricanum*, and species of *Albizia*, *Celtis*, and *Entadophragma*. A photograph of flowers (*Couvreur 568* from Bélinga, Gabon, Nov 2013), shows a small beetle visiting the flower, but the species could not be determined, nor is it known whether the insect was behaving as a pollinator. Hornbills and, perhaps less frequently, monkeys (ex *Harris & Fay 266*) feed on the fruits and seeds; at the Dja Preseerve site in Cameroon, hornbills were particularly effective in extending the seed shadow for individual trees (Holbrook and Smith 2000).

Hutchinson (1923) observed the similarity of the leaves of *Xylopiahypolampra* to those of a number of Neotropical species of the genus such as *X.sericea* and *X.discreta*, implying a phylogenetic connection between South American and African species, but recent phylogenetic work has shown that *Xylopiahypolampra* is not closely related to any tropical American species (Stull et al. 2017).

The original German description of *Xylopiahypolampra* given by Mildbraed in 1913 makes no mention of a type specimen. A Latin description and citation of a type specimen were provided by Mildbraed and Diels (Bot. Jahrb. Syst. 53: 445, 1915), but in the meantime (1914) the name *Xylopiabrieyi*, a taxonomic synonym for *X.hypolampra*, was published by De Wildeman. All of these dates precede the requirement for a Latin diagnosis (ICN 2012, Article 39.1) and for citation of a type (ICN 2012, Article 40.1) so the 1913 description constitutes valid publication and the name *X.hypolampra* with Mildbraed as the sole author is accepted. The holotype for *Xylopiahypolampra* is extant at B and thus the lectotypification provided by Le Thomas (1969) of a duplicate at HBG was unnecessary.

### 32. Xylopia katangensis

Taxon classificationPlantaeMagnolialesAnnonaceae

De Wildeman, Ann. Mus. Congo, Sér. 4, Bot. 1: 32–33. 1902.

41556D4E-EBBC-57DF-A291-1583B1E75100

Fig. 27L

? Xylopiakatangensisvar.gillardinii Boutique, Bull. Jard. Bot. État 21: 109–110. 1951. Type. DEMOCRATIC REPUBLIC OF THE CONGO [“Belgian Congo”]. Kasaï Province, vallée du Kasai, Versant, Makumbi, Terr. de Tshikapa, Jan 1938, *J. Gillardin 352* (holotype: BR!; isotypes: BR ! [0000008824820], K! [000199055]).

#### Type.

DEMOCRATIC REPUBLIC OF THE CONGO [“Belgian Congo”]. Haut-Katanga Province, Lukafu, May 1900, *Ct. E. A. A. Verdick 503* (holotype: BR!).

#### Description.

***Tree*** up to 13 m tall, perhaps taller, d.b.h. up to 60 cm, bole with buttresses at base; bark gray, smooth. ***Twigs*** brown to blackish brown, eventually grayish brown to dark gray, sparsely pubescent, the hairs ca. 0.2 mm long, soon glabrate; nodes with two axillary branches occasional. ***Leaf*** with larger blades 7.1–9.4 cm long, 2.4–3.3 cm wide, subcoriaceous, slightly discolorous, often shiny blue-green adaxially, whitish green abaxially, lanceolate to lanceolate-elliptic, apex acute to short-acuminate, the acumen 4–11 mm long, base cuneate to broadly cuneate, glabrous on both surfaces, rarely with a few hairs abaxially; midrib slightly raised or plane adaxially, raised abaxially, secondary veins somewhat arcuate, irregularly brochidodromous, 10–15 per side, diverging at 50–60° from the midrib, these and higher-order veins raised to strongly raised on both surfaces; petiole 3.5–9 mm long, shallowly canaliculate, glabrous. ***Inflorescences*** axillary or from the axils of fallen leaves, 1–12-flowered, sparsely pubescent to pubescent; peduncles 1–2 per axil, 1–3.5 mm long; pedicels 2–9 per peduncle, 5.7–9.1 mm long, 0.4–0.8 mm thick; bracts 2–4, the upper subtending the sepals and persistent, the lower at or just proximal to pedicel midpoint and caducous, 0.9–1.8 mm long, ovate to semicircular, apex obtuse to rounded; buds linear, occasionally somewhat falcate, apex acute. ***Sepals*** slightly spreading at anthesis, 1/5–1/4-connate, 1.6–2.3 mm long, 2.1–3.4 mm wide, subcoriaceous, broadly ovate to triangular, apex acute to obtuse, pubescent abaxially. ***Petals*** greenish yellow with red on the adaxial bases *in vivo*; outer petals spreading at anthesis, 19–37 mm long, 2.7–3.2 mm wide at base, 0.7–1.4 mm wide at midpoint, chartaceous, filiform, apex obtuse, puberulent adaxially, sparsely pubescent abaxially; inner petals slightly bent outward at the base at anthesis, (11.4–) 17.4–29 mm long, 2.3–2.9 mm wide at base, 0.4–0.6 mm wide at midpoint, chartaceous, needle-like, apex acute, base with undifferentiated margin, puberulent on both surfaces except for the glabrous claw. ***Stamens*** ca. 90; fertile stamens 1.0–1.1 mm long, oblong, apex of connective 0.1–0.2 mm long, depressed-globose to shieldlike, overhanging the anther thecae, minutely papillate, anthers 7–10-locellate, filament 0.4–0.5 mm long; outer staminodes 1.1–1.2 mm long, clavate, apex truncate to emarginate; inner staminodes ca. 0.9 mm long, clavate, apex truncate; staminal cone 0.7–1.5 mm in diameter, 0.4–1.0 mm high, concealing the lower half of the ovaries, rim irregularly laciniate. ***Carpels*** 3–4; ovaries 1.3–1.4 mm long, narrowly oblong, sericeous, stigmas loosely connivent at base with tips free, 2.5–4.4 mm long, linear, glabrous or with a tuft of hairs at the apex. ***Torus*** flat, 1.3–1.8 mm in diameter. ***Fruit*** of up to 2–4 glabrate monocarps borne on a pedicel 8.4–14 mm long, 1.7–2.5 mm thick, glabrate; torus 2.5–4.4 mm in diameter, ca. 2.3–4.1 mm high, depressed-globose. ***Monocarps*** with green exterior and red endocarp *in vivo*, 1.9–3.4 cm long, 1.2–1.5 cm wide, 0.9–1.0 cm thick, oblong, weakly torulose, apex obtuse, base contracted into a stipe 3.5–4 mm long, ca. 3.0–3.5 mm thick, longitudinally wrinkled, verrucose; pericarp 0.4–1 mm thick. ***Seeds*** 1–5 per monocarp, in a single row or in two irregular rows, lying oblique to perpendicular to long axis, 9.3–10.7 mm long, 7.7–7.9 mm wide, 6.7–6.8 mm thick, broadly ellipsoid, broadly elliptic in cross-section, truncate at micropylar end, rounded at chalazal end, brown, smooth or slightly pitted, dull, raphe/antiraphe visible but not raised or sunken, micropylar scar 2.4–2.5 mm long, 2.1–2.4 mm wide, elliptic to circular; sarcotesta gray or light green *in vivo*; aril absent.

#### Phenology.

Specimens with flowers have been collected in February, March, May, August, October, and November, and with fruits in February, April, May, August, and October.

#### Distribution

(Fig. 45). Occurs from north-central Nigeria east to central Cameroon and south to Gabon, southern Democratic Republic of the Congo and northeastern Zambia, where it grows in riparian inundated habitats and mushitu swamp forest at elevations of 470–1220 m.

#### Local names.

Likungu (Turumbu, *Louis 7886*), ogana (Gabonais, *Dybowski 129*).

#### Additional specimens examined.

**NIGERIA.** Jos Plateau, Gindiri, 1040 m, 24 Oct 1957 (fl), *Hepper 1143* (BR, K, P); Northern Nigeria, Zaria Province, Anara F. R., Kan Gini, 20 Oct 1947 (fl), *Keay FHI 20129* (K—2 sheets, OWU spirit collection). **CAMEROON**. Près de Mbeuga (entre Vos et Akowolinga), 8 Mar 1962 (fl), *Letouzey 4498* (K, MO—2 sheets, P). **GABON.** Bords de l’Ogo[o]ué en fore Achouka, 26 Aug 1895 (fl, fr), *Dybowski 129* (P). **DEMOCRATIC REPUBLIC OF THE CONGO**. Haut-Katanga: Prov. Katanga, station de Keyberg, 8 km SW de Lubumbashi, 23 Mar 1954 (fl), *Schmitz 4748* (K, WAG).—Haut-Lomami: Mupulu, récolté à Kaniama galerie Luba, Feb 1938 (fl), *Herman 2212* (BR); Mupulu, Kaniama galerie Luba, Feb 1938 (fl), *Prignon 4* (BR).—Lualaba: Kisanga, 1220 m, 2 Feb 1979 (buds), *Malaisse 9667* (BR).—Tshopo: Yangambi, 1949 (buds), *Gilbert 7739* (K, P); Yangambi, 1949 (fl), *Gilbert 9060* (K, P); Yangambi, 1949 (st), *Gilbert 9344* (K); Yangambi, ca. 470 m, Ile Tutuku, en face du plateau de l’Isalowe, 15 Feb 1938 (fr), *Louis 7886* (B, BM, BR, FI-T, K, MO, P, US); en face d’Tsangi, 470 m, 14 May 1938 (fl), *Louis 9374* (B, BM, K, MO); en face d’Tsangi, ca. 470 m, 7 Sep 1938 (bud), *Louis 11151* (F, NY, RSA). **ZAMBIA**. River bank Chambezi, 26 Aug 1927 (fr), *Bourne 96* (FHO); Kasama, Kasama Luwingu, 3 Sep 1927 (st), *Bourne 124* (FHO); Zambezi Rapids, Ikelenge, 2 Nov 2004 (fl), *Congdon 673* (K); Kapalala, Kapalala, Luapula R., 16 Apr 1933 (fr), *Duff 134/33* (FHO); Luapula R., 29 Oct 1949 (fl), *Fanshawe 1423* (K); Lumangwe District, 14 Nov 1957 (fl), *Fanshawe F.3989* (BR, K); Central Province, Kasanka National Park, along Musande River near confluence of Musande and Luwombwa Rivers, 12°31'38"S, 30°07'51"E, 1170 m, 17 Nov 1993 (fl), *Harder et al. 1918* (MO); Northern Province, Mporokoso District, Mukubwe River, S of Mweru Wantipa, 28 Oct 1949 (fl), *Hoyle 1329* (FHO); Nchelenje, L. Mweru, 7 Oct 1961 (fr), *Lawton RML768* (FHO, K); Nchelenje, 5 Feb 1962 (fl), *Lawton RML830* (FHO); Western Province, Fort Rosebery District, Lake Bangweulu, Lake Bangweulu (island), N end, 20 May 1931 (fr), *Stevenson 265/31* (FHO); Western Province, Kawambwa District, at edge of Lake Mweru, near Kafulwe Mission, 4 Nov 1952 (buds), *White 3610* (FHO, K).

*Xylopiakatangensis* is similar to *X.longipetala*, resembling that species in the long and exceedingly narrow petals and the long stigmas, but differing in the proportionately longer petioles, more complex inflorescences with larger numbers of flowers, sepals not reflexed at anthesis, petals sparsely hairy and rigid (without the strip-of-crepe-paper effect), and outer petals narrower at the base, thus not producing a globose flower base. The shiny blue-green upper surface of the leaf is evident on many specimens.

Specimens from Tshopo Province of the Democratic Republic of the Congo, and from Gabon, Cameroon, and Nigeria, do not seem distinguishable from the other material, despite the great geographic separation. In addition to their morphological similarity, all come from similar inundated habitats, including *mushitu* forest in Zambia and periodically inundated riverbanks elsewhere. The label of *Letouzey 4498* describes the habitat as “Abondant dans le rideau forestier fragmentaire, uniquement inondé, sur la berge du Nyong [river], en bordare de la prairie à *Echinochloastagnina*,” the latter a grass tolerant of flooding.

The specimen *Gillardin 352*, the type of Xylopiakatangensisvar.gillardinii, exhibits a number of small differences from nominate *X.katangensis*: the leaves are chartaceous and the blades are distinctly decurrent on the petioles, the inflorescences have only one or two flowers and the pedicels are only 3–5 mm long with 3–5 imbricate bracts. The petals of both whorls are shorter than is typical for *X.katangensis*, the outer ca. 16.5 mm long and the inner ca. 12.5 mm long. We have seen no additional specimens combining this set of characters, but the specimen *Letouzey 4498* has some pedicels with 4 bracts, the specimen *Fanshawe F.3989* had some pedicels as short as 5.7 mm, and the specimen *Keay FHI 20129* has some inflorescences with only one flower and inner petals as short as 11.4 mm. We conclude that X.katangensisvar.gillardinii does not merit taxonomic recognition based on current information, but wish to document these points of difference.

### 33. Xylopia letestui

Taxon classificationPlantaeMagnolialesAnnonaceae

Pellegrin, Bull. Mus. Hist. Nat. Paris 26: 658. 1920.

58787FD8-CFD0-5D9E-B80A-D81DA96CDBBC

Fig. 44

Xylopia letestui var. longepilosa Le Thomas, Fl. Gabon 16: 178 + t. 33, 10–11. 1969. Type. GABON. Ngounié Province, Moumba, Haute Ngounyé, 3 Sept 1926, *G. Le Testu 6046* (holotype: P! [00169154]; isotypes: BM! [000511049], BR! [0000008825315], P! [00169153, 00169155]).

#### Type.

GABON. Nyanga Province, Mayombe Bayaka, Tono-Sangama, 9 Aug 1914, *G. Le Testu 1760* (holotype: P! [00169125]; isotypes: BM! [000511048], BR! [0000008825322], EA!, K! [000199054], LISC! [000403], P! [00169126, 00169127]).

#### Description.

***Tree*** up to 40 m tall, d.b.h. up to 28.5 cm, bole straight, buttresses narrow and thin, up to 80 cm high and extending up to 50 cm from the trunk, branches horizontal from trunk; bark pinkish beige, with shallow vertical grooves or striations. ***Twigs*** initially often lax and sinuous or somewhat zigzag, bearing a sheaf of overlapping new leaves at the apex, reddish-brown, brown or gray, densely covered with hairs 0.4–1.0 mm long, often marked with decurrent ridges from either side of the petiole base, eventually glabrate; nodes occasionally with two axillary branches. ***Leaf*** with larger blades 4.7–10.9 cm long, 1.2–2.7 cm wide, subcoriaceous, discolorous, lanceolate to lanceolate-oblong, apex acute, base truncate and often slightly asymmetrical, glabrous or pubescent along the midrib adaxially, sparsely to densely appressed-pubescent abaxially; midrib plane to slightly raised adaxially, raised abaxially, secondary veins brochidodromous, 8–14 per side, diverging at 45–50° from the midrib, these and higher order veins plane or slightly raised adaxially, raised abaxially; petiole 1–2.2 mm long, semi-terete, pubescent. ***Inflorescences*** axillary, 1–4-flowered, pubescent; peduncle 1 per axil, ca. 2.5 mm long; pedicels 2 per peduncle or arising directly from the axil, 1.4–6.9 mm long, 1.1–1.2 mm thick; bracts 2, evenly spaced along pedicel, often with lower bract caducous and upper bract persistent, 2.3–6.0 mm long, ovate, deltate, semicircular, or circular, apex acute or sometimes rounded; buds lanceolate to lanceolate-oblong, apex acute to rounded. ***Sepals*** spreading at anthesis, 1/4–2/3 connate, 4.2–5.6 mm long, 3.1–3.2 mm wide, ovate to elliptic, apex obtuse to acute, appressed-pubescent abaxially. ***Petals*** cream-colored adaxially, becoming red toward the base *in vivo*; outer petals with position at anthesis not determinable, 13–22 mm long, 3.0–3.7 mm wide at base, 2.3–3.2 mm wide at midpoint, somewhat fleshy, lanceolate-ligulate, linear-lanceolate, or narrowly triangular, apex acute, puberulent along margins and on apex but otherwise glabrous adaxially, densely appressed-pubescent except for two small glabrous patches at the base abaxially; inner petals with position at anthesis not determinable, 10.3–18.1 mm long, 2.4–3.2 mm wide at base, 1.4–1.9 mm wide at midpoint, somewhat fleshy, linear-lanceolate, apex acute, densely appressed-pubescent for distal 1/3, sparsely pilose in center proximal to that and base glabrous adaxially, with tufts of hairs on margin at the widest point of the base, densely appressed-pubescent abaxially. ***Stamens*** numerous; fertile stamens 1.3–1.6 mm long, narrowly oblong, apex of connective 0.2–0.3 mm long, depressed-globose to shieldlike, overhanging anther thecae, glabrous, anthers 12–13-locellate, filament 0.3–0.5 mm long; outer staminodes ca. 1.5 mm long, broadly clavate, apex obtuse to truncate; inner staminodes 1.2–1.3 mm long, oblong, apex truncate; staminal cone 1.2–1.6 mm in diameter, 0.9–1.0 mm high, concealing all but the apices of the ovaries, rim laciniate. ***Carpels*** 7–10; ovaries 1–1.1 mm long, oblong, pilose, stigmas loosely appressed in lower half with the tips free, 3–3.5 mm long, linear, sinuate, pilose. ***Torus*** flat, 1.8–2.0 mm in diameter. ***Fruit*** of up to 6 brown-pubescent monocarps borne on a pedicel 5–16 mm long, 5–10 mm thick, this in turn borne upon a leafless branch ca. 15 cm long, glabrate; torus 8–19 mm in diameter, 7–11 mm high, irregularly depressed-globose. ***Monocarps*** with green exterior and bright red endocarp *in vivo*, 2.7–4.5 cm long, 2.1–3.4 cm wide, 2.0–3.5 cm thick, nearly spherical to ellipsoid, not torulose, apex rounded or obtuse, tapered to a sessile base 7–10 mm wide, weakly wrinkled and verrucose; pericarp 5–7 mm thick. ***Seeds*** up to 6 per monocarp, in two rows, lying perpendicular to long axis, 15–19 mm long, 8–13 mm wide, 8–10 mm thick, ellipsoid, flattened-elliptic in cross-section, truncate at micropylar end, rounded at chalazal end, brownish white, smooth, dull, raphe/antiraphe not evident, micropylar scar 4.5–5.5 mm long, 4.2–4.8 mm wide, broadly elliptic to nearly circular; sarcotesta grayish white (“glaucous”) *in vivo*; aril absent.

**Figure 44.**
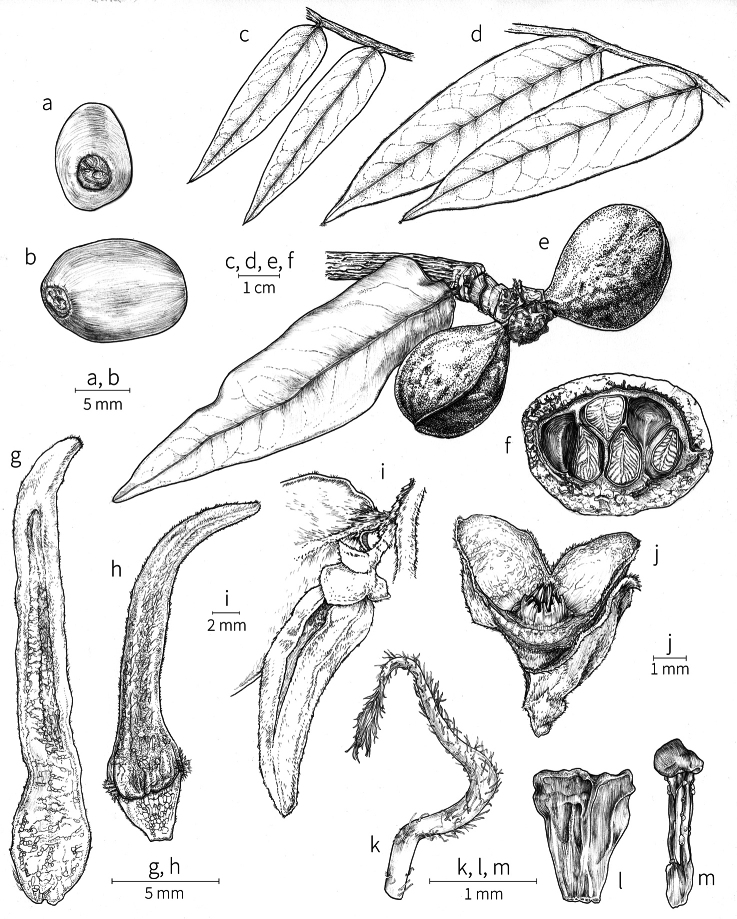
*Xylopialetestui*. **A** Seed, view of micropylar end **B** Seed, side view **C, D** Habit **E** Habit including fruit **F** Fruit, transmedian section showing seed arrangement **G** Outer petal, adaxial view **H** Inner petal, adaxial view **I** Flower bud **J** Bract, sepals, and staminal cone **K** Stigma, side view **L** Staminode, abaxial view **M** Fertile stamen, abaxial view. **A, B, E, F** from *Breteler et al. 8867* (WAG) **C** from *Hallé 4274* (WAG) **D** from *Leeuwenberg 3073* (WAG) **G–I** from *van der Burgt 1129* (MO) **J–M** from *Le Testu 5975* (BM).

#### Phenology.

Collections with flowers have been made in February and April, and from July to September, and with fruits in January, March, April, November, and December.

#### Distribution

(Fig. 43). Occurs from Sierra Leone to southwestern Ghana, and from southeastern Nigeria south to southern Gabon and western Democratic Republic of the Congo, growing in lowland wet forests, sometimes secondary forests, on both clay and well-drained sandy soil, at elevations of 50–450 m.

#### Local name.

Canzi (Bayaka, *Le Testu 1760*).

#### Additional specimens examined.

**SIERRA LEONE.** Nyagoi Protected Forest, s. d. (fr), *King 158* (K). **LIBERIA.** Sino, Sapo NP, buffer zone, along new logging road, 5°25'N, 08°46.2'W, 29 Nov 2002 (fl buds), *Jongkind 5544* (WAG); first part of Babooni road walking in the direction of Sapo NP, 5°31.20'N, 8°37.800'W, 110–140 m, 5 Mar 2009 (fr), *Jongkind et al. 8821* (K). **IVORY COAST.** 55 km ENE of Sassandra, about 15 km N of Fresco, 16 Mar 1959 (fr), *Leeuwenberg 3073* (K, P, WAG–3 sheets); Banco, *Martineau 227* (P). **GHANA.** Fure Forest Reserve near Prestea, 15 Dec 1971 (yg fr), *Deaw Sp 465* (MO, NY, RSA). **NIGERIA.** Oban, 1912 (fl), *Talbot s. n.* (BM). **CAMEROON.** Southwest Province, Ndian Division, Korup National Park, 5°01'N, 8°48'E, 100 m, 24 Feb 2008 (fl), *van der Burgt 1129* (K, MO); East, Department Haut-Nyong, Dja Reserve, Bouamir Research Area, 90 km SE of Akonolinga, 3°11'05"N, 12°47'39"E, 640–700 m, 23 May 1997 (st), *Fogiel 2098* (MO); TDC, Sud Cameroun, 26 Nov 1991 (fr), *Hallé 4274* (WAG). **GABON.** 5–10 km E of Saint Germain, E of Okamo River, 11°38’ E, 0°38'N, 20 Apr 1988 (fr), *Breteler et al. 8867* (MO, WAG); Bélinga Mines de Fer, 21 Jul 1966 (fl), *Hallé & Le Thomas 122* (K, P); Mimongo, *Le Testu 5975* (BM); Woleu-Ntem, Mbe National Park, Monts de Cristal, Tchimbele Dam area, 00°37'02"N, 10°24'49"E, 400 m, Apr 2004 (st), *SIMAB 012002* (MO); Woleu-Ntem, Monts du Cristal, 10 Sep 2001 (st), *Nguema 341* (MO). **REPUBLIC OF THE CONGO.** Chaillu, region de Komono, route de Mbila, forêt des environs du village Mitoko, 24 Jan 1977 (fr), *Sita 4081* (P). **DEMOCRATIC REPUBLIC OF THE CONGO.** Équateur: Terr. Bikoro, route Weti-Iboko, 16 Apr 1959 (fl), *Evrard 6189* (K).

The nearly sessile leaves with truncate bases, the short inner petals bearing conspicuous basal hair tufts, and the thick-walled globose monocarps readily distinguish *Xylopialetestui* from its congeners. It is similar to *X.villosa*, both being large trees with narrow buttresses, leathery leaves that are appressed-pubescent beneath, and relatively large thick-walled monocarps. In addition to the truncate leaf bases and the hair tufts on the outer petals, *X.letestui* differs from *X.villosa* in having abaxial leaf indument that is dull-colored, not golden-sericeous, and four or fewer flowers in the inflorescences. The sinuous young shoots with conduplicate new leaves sometimes seen in *X.letestui* have not been noted in specimens of *X.villosa*. The presence of *X.letestui* in West Africa was previously unreported (Keay 1954–1958, 1989, Holmgren et al. 2004), probably in part due to its similarity to *X.villosa*, which occurs over much of the same range. Associates listed on herbarium labels include *Alstoniaboonei*, *Dichostemmaglaucescens*, *Podococcusbarteri* (understory), *Santiriatrimera*, *Tabernaemontanacrassa*, *Terminalia superba*, and *Uapacaguineensis*.

Le Thomas (1969) distinguished two specimens from Gabon with longer and denser indument as a separate variety, X.letestuivar.longepilosa. We found density and length of hairs to vary over the range of the species, and that the densest hairs seem to accompany specimens bearing new flushes of growth, suggesting that the characteristic is variable and does not merit taxonomic recognition.

The label of *Leeuwenberg 3073* describes the specimen as being from a large liana about 25 m high, but this growth form has not been corroborated by any other specimen data, all of which describe the plant as an erect forest tree.

### 34. Xylopia longipetala

Taxon classificationPlantaeMagnolialesAnnonaceae

De Wildeman & T. Durand, Ann. Mus. Congo, Sér. 2, Bot. 1(1): 4. 1899.

0155D4C0-7E7D-50EF-B559-CC3E4AF10074

Figs 3E , 27A–F , 28B

Uvaria parviflora A. Richard in Guillemin, Perrottet, & A. Richard, Fl. Senegamb. tent., part 1, 9 + t. 3, fig. 1. 1831.
Coelocline ? parviflora (A. Richard) A. de Candolle, Mém. Soc. Phys. Genève 5: 209. 1832.
Unona parviflora (A. Richard) Steudel, Nomencl. Bot., ed. 2, 2: 730. 1841.
Xylopia parviflora (A. Richard) Bentham, Trans. Linn. Soc. 23: 479. 1862, non X.parviflora Spruce, 1861.
Xylopicrum parviflorum (A. Richard) Kuntze, Revis. gen. pl. 1: 8. 1891.
Xylopia vallotii Chipp ex Hutchinson & Dalziel, Fl. W. Trop. Afr. 1(1): 53. Mar 1927, nom. nov. Type. SENEGAL. Ziguinchor Region [?], “crescit ad oram sylvarum et in locis siccis riparum Casamanciae prope Maloum,” 3 or 4 Apr 1829, *G. G.-S. Perrottet s. n.* (lectotype, here designated: P! [00169145]; isotypes: B! [10 0273361, probable], BM! [000511054], G! [00190717], P! [00169144, plus 4 additional sheets lacking bar codes]).

#### Type.

DEMOCRATIC REPUBLIC OF THE CONGO [“Belgian Congo”]. Équateur Province, Rég. III, Makanza [“Bangala”], May 1896, *A. Dewèvre 876* (holotype: BR!; isotypes: BR! [0000008825360, 0000008825377]).

#### Description.

***Shrub or small tree*** up to 15 m tall, d.b.h. up to 40 cm, bole cylindrical, sometimes fluted toward the base, crown branched profusely; bark dark gray to dark brown, occasionally whitish gray or yellowish, smooth. ***Twigs*** initially dark gray to brown, soon light gray, sparsely erect-pubescent, the hairs 0.1–0.5 mm long, soon glabrate; nodes frequently with two axillary branches. ***Leaf*** with larger blades 4.2–8.8 cm long, 1.7–3.7 cm wide, subcoriaceous to chartaceous, slightly discolorous, elliptic to oblong, lanceolate, lanceolate-ovate, or ovate-oblong, apex obtuse to acuminate, the acumen 4–8 mm long, base rounded and short-decurrent on petiole, glabrous or with a few hairs along lower midrib adaxially, sparsely appressed-pubescent, rarely with only hairs along midrib, abaxially; midrib slightly raised or plane adaxially, raised abaxially, secondary veins indistinctly and weakly brochidodromous, 9–14 per side, diverging at 45–70° from the midrib, these and higher-order veins slightly raised on both surfaces; petiole 2.5–6 mm long, shallowly canaliculate, pubescent to glabrate. ***Inflorescences*** axillary, 1–4-flowered, sparsely pubescent to glabrate; peduncle 1 per axil, 1–1.5 mm long, or absent; pedicels 2 per peduncle, 6.2–12 mm long, 0.6–0.9 mm thick; bracts 2–3, all attached at or proximal to the pedicel midpoint, persistent, 1.1–1.8 mm long, ovate to semicircular, apex rounded or emarginate; buds linear, apex acute, occasionally somewhat falcate, base bulbous. ***Sepals*** usually reflexed at anthesis, 1/6–1/3-connate, 2.1–2.8 mm long, 1.8–2.4 mm wide, chartaceous, triangular, apex acute, pubescent abaxially. ***Petals*** pale green to greenish yellow and bright pink, red, or purple at base *in vivo*; outer petals bent outward at the base but with the tips curved inward at anthesis, 16–62 mm long, 2.7–5 mm wide at base, 0.9–1.8 mm wide at midpoint, chartaceous to membranous, linear, lax and ribbonlike, apex obtuse, sparsely tomentose, becoming glabrous in basal concavity adaxially, densely tomentose abaxially; inner petals sharply bent outward at the base but with the tips curved inward at anthesis, 19–48 mm long, 3.1–5.2 mm wide at base, 0.4–0.8 mm wide at midpoint, chartaceous to membranous, linear-subulate, lax and ribbonlike, apex acute to obtuse, base with undifferentiated margin but slightly auriculate, somewhat thickened at the widest point adaxially, glabrous or with a few scattered hairs, except for the densely pubescent, almost setose, concavity, adaxially, pubescent, densely so at the base, abaxially. ***Stamens*** 80–100; fertile stamens 1.1–1.5 mm long, narrowly oblong, apex of connective red *in vivo*, 0.1–0.3 mm long, depressed globose to shieldlike, overhanging anther thecae, minutely papillate, anthers 9–11-locellate, filament 0.3–0.4 mm long; outer staminodes 1.3–1.6 mm long, clavate, apex truncate to emarginate; inner staminodes 0.8–1.1 mm long, oblong to broadly clavate, apex truncate; staminal cone 0.9–1.4 mm in diameter, 1.0–1.3 mm high, partially concealing the ovaries, rim laciniate. ***Carpels*** 5–7; ovaries 1.2–1.4 mm long, narrowly oblong, tomentose, stigmas white *in vivo*, loosely connivent but with apices separated, 3.8–7 mm long, linear, sometimes somewhat falcate, glabrous or with a tuft of hairs at the apex. ***Torus*** flat, 1.2–2.3 mm in diameter. ***Fruit*** of up to 6 glabrate monocarps borne on a pedicel 10–17 mm long, 2–3.2 mm thick, glabrate or with a few hairs; torus 6.5–11 mm in diameter, 4–8 mm high, depressed-globose. ***Monocarps*** with green exterior, sometimes purple to red tinged, and bright red endocarp *in vivo*, 3.0–4.4 cm long, 1.1–1.9 cm wide, 1.1–1.3 cm thick, irregularly oblong, often weakly torulose, apex obtuse to rounded, sometimes with a short beak up to 2 mm long, base sessile or contracted into a stipe 1–5 mm long, 2.5–8 mm thick, longitudinally marked with 3–4 strong ridges, wrinkled, verrucose; pericarp 0.1–0.3 mm thick. ***Seeds*** 7–12 per monocarp, in two rows, lying perpendicular to long axis, 10.3–12.4 mm long, 6.0–8.6 mm wide, 5.0–6.9 mm thick, ellipsoid to ellipsoid-pyriform, narrowed toward micropyle into a cylindrical neck 1–2 mm long and 3.1–3.8 mm wide, ovate in cross-section, truncate at micropylar end, rounded at chalazal end, brown, smooth, dull, raphe/antiraphe not or only faintly evident, micropylar scar 1.8–2.5 mm long, 1.5–2 mm wide, obovate to roughly circular; sarcotesta white to green *in vivo*; aril absent.

#### Phenology.

Specimens with flowers have been collected from all months of the year, but in slightly higher numbers from November to January. Specimens with fruits have been collected from all months of the year except February and March, with the greatest number from June.

#### Distribution

**Figure 45.**
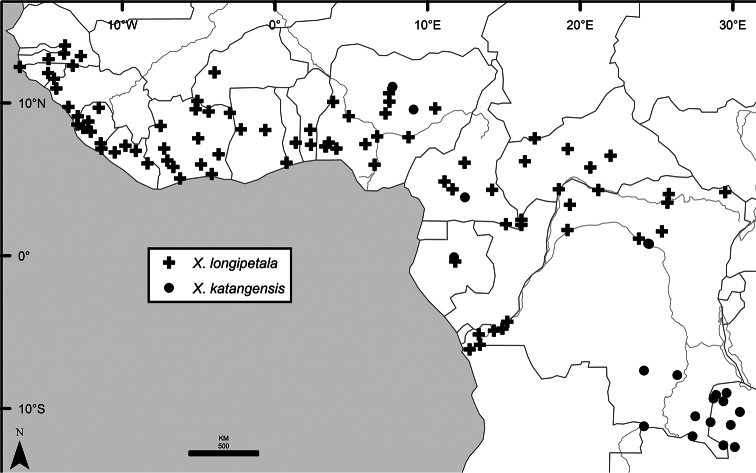
Distributions of *Xylopialongipetala* and *X.katangensis*. Bolder lines represent country borders, fainter lines lakes and major rivers.

(Fig. 45). *Xylopialongipetala* is a species of inundated riparian forest, sometimes on sandy soils, distributed from Senegal to southern Chad and south to northwestern Angola and northeastern Democratic Republic of the Congo, at elevations of 30–700 m. It does not extend beyond the Congo River basin in the northeastern part of its distribution.

#### Local names.

Le Thomas (1969) lists only “ogana” as a common name for the species, a name applied to other species of *Xylopia* in Gabon as well; no other local names have been reported.

#### Representative specimens.

**SENEGAL.** Tambacounda, Miokolo-koba, 12 Jun 1958 (fr), *Adam 14340* (MO, P); Tambacounda, Mieriko, 3 Dec 1959 (fl), *Adam 17332* (MO); Oussouye, Kabrousse, 8 Oct 1961 (fr), *Adam 18369* (MO); Ziguirihor, Elinkine, 8 Oct 1961 (fl), *Adam 26904* MO); Niokolo-Koba, Apr 1951 (fr), *Berhaut 1476* (P); Ouassadou, 1 Jan 1954 (fl), *Berhaut 4295* (P), 4304 (P); d’Oussouye, 6 Aug 1983 (fr), *Berhaut 6314* (BR, M, P); Cabrousse, 22 Feb 1964 (bud), *Berhaut 7086*(M, P); forêt classe de Mahon, close to Kolda town, along the Casamance River, 50 m, 12°52'N, 14°50'W, 5 Jul 1990 (fr), *Lawesson & Goudiaby 7079* (AAU, MO); prope Maloume Cap Rouge, May 1826 (fr), *Leprieur s. n.* (P); Tambacounda Region, Parc National du Niokolo Koba, Passage Koba, 13°03'N, 13°10'W, 30 m, 17 Dec 1993 (fl), *Madsen et al. 2970* (AAU). **GUINEA BISSAU.** Bafatá, Chitole, Cusselinta, 10 Dec 1952 (fl), *Explorações Botânicas 3171* (BR, K); Boé, entre Contabane e Guilege, 9 Jun 1953 (fr), *Explorações Botânicas* 3206 (WAG). **GUINEA.** Bassin de la Gambie, Youkounkoun, Jan 1952 (fl), *Berhaut 2145* (P); Carcle de Bokè entre Songolon et Falaba, Region gresense, 18 Apr 1926 (fr), *Chillou s. n.* (P); env. de Conakry, Jun 1913 (fl), *Morran s. n.* [herb. Ch. d’Alleizette] (L). **SIERRA LEONE.** Kabala, M’Loma, Kruto, 6 Feb 1966 (fl), *Adam 23587* (MO); Njala, 13 Feb 1928 (fl), *Deighton 1081* (BM—2 sheets; K); Njala, 29 May 1935 (yg fr), *Deighton 2997* (K); near Taiama, 26 Apr 1940 (fr), *Deighton 3958* (K); Moa River at Koteimahun, (fl), *Lane-Poole 347* (K); near Kambia, Scarcies, 5 Jan 1892 (fr), *Scott-Elliot 4733* (BM); Moa bridge towards Zimmi, Nongowa Chiefdom, 10 Dec 1965 (fl), *Samai 278* (K); near Port Lokko, Karena District, Apr 1892 (fr), *Scott-Elliot 5730* (K); Laminaiya, 350’, 28 Apr 1914 (yg fr), *Thomas 144* (K). **LIBERIA.** Central Province, Gbarnga District, Yila, St. John River, 19 Aug 1947 (fr), *Baldwin 9131* (K, MO, US); Western Province, Boporo District, Ba, on Mano River, near Boundary with Grand Cape Mount County, 18 Dec 1947 (fl), *Baldwin 10714* (K, MO, US); Tapeta-Chien road, bank of Cestos river, Nimba side, 13 Jan 1967 (fl), *Bos 2754* (K, WAG); Piahtah [“Peáhtah,” 7°12'N, 09°48'W], 5 Oct 1926 (fr), *Linder 910* (A, K, P), 17 Oct 1926 (fl, fr), *Linder 1115* (A, K—2 sheets); road Bomi Hills to Lofa River, 11 Dec 1965 (fl), *van Meer 250* (MO, WAG). **BURKINA FASO.** Dédougou: Bondoukuy, 11°59'42"N, 3°57'16"W, 268 m, 23 Nov 2009 (fl, fr), *Sanou BUR-766* (K). **IVORY COAST.** Comonbêlo no. 2, Comivé Adikokoi, 10 Jan 1952 (fl), *Aubréville 667* (A, B, K, P); Comonbelo, Touba, 23 Apr 1932 (fr), *Aubréville 1245* (P); Kouofi, Lalérabah, May 1932 (st), Aubréville 1422 (P); La Lerabah, (fl), *Aubréville 2201* (P); Ferké, (fl), *Aubréville 2622* (P); R. Sassandra au N de Guessabo, 31 Jan 1969 (fl), *Bamps 2006* (BR, P); Parc Komoé, Galerie Iringou, 8 Jun 1977 (st), *César 553* (P); bassin de la moyen Sassandra Soubré (bord de la Sassandra), 18 Jun 1907 (fr), *Chevalier 19113* (P); 30 km NE of Bouna, 9°21'N, 2°57'W, 3 Dec 1967 (fl), *Geerling & Bokdam 1625* (K, MO, WAG); Bord du N’Zi, Pont Gt Reste, 22 Dec 1956 (fl), *Institute D’Enseignement et de Recherches Tropicales (Adiopodoumé) 4069* (P); Sassandra District, Louga, near Sassandra River, 25 Jan 1975 (fl), *de Koning 5214* (WAG); Ferké village, Kafolo, à 120 km à l’est de Ferké ville, au safari lodge, Taillis a proximite du pont, 23 Nov 1977 (fl), *Munzinger 30* (K, MO, P); 25 km NNE of Bouaké, direction Sibrikro, 20 Jun 1969 (fr), *Versteegh & den Outer 359* (WAG); forest exploitation of Mr. Nesvadba on Sassandra River, W of Soubré near the village Niamagbi, 11 Jun 1963 (fr), *de Wilde 164* (K, P); border of the N’zi (tributary of the Bandama river), near the bridge crossing N’zi between Ndouci and Singrobo, 6 Nov 1961 (fl), *de Wilde 3236* (A, B, K, P); Sassandra River between Péhiri and Kopréagui, ca. 7 km WNW of Soubré, 25 Nov 1961 (fl), *de Wilde 3306* (K). **GHANA.** Yeli Yeji (on R. Volta), 7 Jun 1937 (yg fr), *Akpabla 656* (K); Brong-Ahafo Region, banks and islands of Black Volta River, E of Agbadzikrom, 300 m, 8°16'N, 2°14'W, 29 Sep 1996 (fr), *Jongkind & Nieuwenhuis 3130* (WAG); without definite locality, *Vigne 3883* (BM, US). **TOGO.** Entre Mission Tové et Doviè, Forêt d’Assomè, 29 Jun 1994 (fr), *Aké Assi 19025* (MO); Nangbeto, en oval du borrage, ca. 110 m, 22 Nov 1986 (fl), *Schäfer 8910* (B, K, MO). **BENIN.** Bords de la riviére Ouémé, entre Save et Ogougou, 5 May 1910 (fr), *Chevalier 23586* (K, P); Adja Ouèrè [7°0'N, 2°37'E], 21 Dec 1901 (fl), *Le Testu 257* (BM, P); forêt de Zarnon près Zaguanado, 16 Nov 1900 (fl), *Poisson 36* or *3_122* (P). **NIGERIA.** Conf. of Quarre & Chadda et ad Nupe [Lokoja *fide*Keay 1954–1958], 1858 (fl), *Barter 426* (K, P); Nupe [*fide*Keay 1954–1958, but is name of ethnic group], s. d. (fl, fr), *Barter 1035* (GH—2 sheets, K, P); confluence of the Kwoma and Tchadda, 1858 (fl), *Barter s. n.* (K); Kaduna State, Damari River Basin, 15 km up river from jct. at Tubo River, vicinity of Mai-Gishiri, 1900–1950 ft, ca. 10°05'N, 7°30'E, 14 Nov 1981 (fl), *Croat 53409* (K, MO); Abinsi & vicinity, 3 Dec 1912 (fr), *Dalziel 713* (BM, K—2 sheets, MO, P); Kwara State, Borgu District, Babana, bank of river Ofa, 18 Dec 1972 (fl), *Eimunjeze & Latilo FHI 65625* (K); banks of the Gurara River near Gornapara, 26 Jul 1906 (fr), *Elliott 171* (K—2 sheets); Bendel State, Kwale District, Aboh Forest Reserve, 15 Aug 1978 (fr), *Ekwuno et al. PFO.150* (*FHI 87627*) (MO); Lagos, Olokemeji, s. d. (fl), *Foster 99* (*s. n.* at P) (K—2 sheets, P); Oyo, Iseyin, Upper Ogun Cattle Ranch, bank of River Ogun, 14 Jun 1977 (fr), *Gbile 5018* (MO); NE State, Bauchi Province, Yankari Game Reserve, R. Gaji near Guruntun bridge, 28 Oct 1970 (fl), *Geerling 3098* (WAG); Jebba, on the Niger, 35 m, 11 Dec 1927 (fl), *Hagerup 725* (BM, K, P); Olokmeji Forest Reserve, 8 Nov 1969 (fl), *Jackson-Etukendo UIH 281169* (K); bank of Ofum or Ofur, Olohem eji Reserve, 25 Apr 1945 (fl), *Onochie & Jones FHI 14516* (K, P); Ilorin Province, Central Borgu Game Reserve, River Oli at hippopotamus pool, 12 Jan 1970 (st), *Medler 201* (MO); Kaduna Div., roadside at Rigachikun, 7 Dec 1949 (fl), *Meikle 757* (K, P); Abeokuta Province, Egba District, 28 May 1958 (fr), *Onochie FHI 38347* (K); Niger State, Abuja District, Gurara water fall, 23 Nov 1987 (fl), *Oyayomi et al. OFOOA:127* (*FHI 79827*) (MO—2 sheets); Ondo Province, Owo District, Idogun-25 mi N of Owo, bank of Osse River, 24 Oct 1961 (fl), *Stanfield 45712* (K); Abeokuta Province, Egba District, ½ mile S of Olokemegi relay station, 30 Nov 1945 (fl), *Tenejong FHI 14346* (K, P); Niger Expedition, Quorra, *Vogel 103* (K), *Vogel s. n.* (K). **CAMEROON.** Rive Sangha, 5 Nov 1945 (fr), *Aubréville 241* (P—2 sheets, WAG); on island in the Sangha River adjacent to the Ndakan gorilla study site, 02°01'N, 16°09'E, 13 Mar 1988 (fl), *Fay 8307* (MO); Eastern Province, W bank of Sangha River, 350 m, 2°23'N, 16°10'E, 22 May 1988 (fr), *Harris & Fay 757* (K); East, Dzanga-Sangha Reserve, 45 km S of Lidjombo, E bank of Sangha River from Ndakan, 02°21'N, 16°09'E, 350 m, 4 Nov 1988 (fl), *Harris & Fay 1535* (K, MO, P, PRE); left bank Sanaga River, near ferry Nachtigal, about 20 km N of Obala, 400 m, 19 Nov 1965 (fl), *Leeuwenberg 7033* (B, BR, K, MO, P, WAG); Djerem entre Niadam et Tagbou, 27 Jun 1954 (fr), *Letouzey 2261* (K, P); rives du Djerem près Mbakaou, 8 Dec 1959 (fl), *Letouzey 2459* (BR, K, P); Yangafok II-25 km ENE de Bafia, 26 Nov 1969 (fl), *Letouzey 9614* (K, P); rive du Dja près Ndongo, a 40 km WNW de Moloundou, 18 Mar 1963 (fl), *Letouzey 12138* (K, P); Bertoua Batouri, 1962 (fr), *Tchinaye 103* (P); 6 km NW du confluent Boumba Dja Ngoba, 17 Apr 1971 (fl), *Villiers 666* (P); left bank Sanaga River, near ferry Nachtigal, ca. 20 km N of Obala, 400 m, 11 Jun 1964 (fl, fr), *de Wilde & de Wilde-Duyfjes 2676* (B, BR, K, MO, P, WAG). **CHAD.** Komba (c. Paoua), River Tchad Nana Barga, 2 Nov 1968 (fl), *Gaston 2488* (P). **CENTRAL AFRICAN REPUBLIC.** Gubungui (Ft. Crunfort), 23 Nov 1902 (fl), *Chevalier 6363* (P); Haute-Kotto, Brio, 10 Feb 1921 (fl), *Le Testu 2446* (BM, K, P, US); Waka, *Tisserant 477* (BM, P), *934* (BM), Bambari, *Tisserant 934 bis* (BM, P); 15 km S Bozoum, 10 Jan 1938 (fl), *Tisserant 3640* (BM); Ouaka, *Tisserant s. n.* (A, K). **GABON.** Ogooué-Ivindo: Petit Okano, viaduc du Transgabonais, ca. 0°04'S, 11°52'E, rive de l’Ogo[o]ué, 10 Oct 1983 (fl, fr), *Floret et al. 1804* (P); Bord d’Ogooué, Booué, 26 Jul 1966 (fl), *Hallé & Le Thomas 190* (MO, P); northern edge of Lopé Reserve, along riverbank near hotel, 00°15'S, 11°40'E, 200 m, 14 Sep 2000 (fl), *McPherson 17911* (MO); Lope Hotel ground, 15 Mar 2004 (fl), *Randrianasolo et al. 820* (MO, NY); border of Ogooué, ca. 20 km W of Lopé-reserve, 0°15'S, 11°28'E, 13 Jun 1966 (fl), *Reitsma & Reitsma 2318* (MO, NY, RSA, WAG); Reserve de Lopé-Okanda, ca. 200 m N of Lope Hotel on Ogooué River, 00°05'38"S, 11°34'58"E, 120 m, 28 Oct 2000 (fr), *Stone et al. 3122* (MO); Lope Reserve, Ogooué-Portes d’Okanda, 0°15'S, 11°40'E, ca. 200 m, 30 Jul 1993 (fl), *White 0944* (MO).—Province unknown: Ogooué, Feb 1895 (fl), *Thollon 144* (P); Ogooué, Feb 1887 (fl), *Thollon 743* (P). **REPUBLIC OF THE CONGO.** Rive de l’Oubangui ou Bangui a la Kemo, 10–20 Aug 1902 (fl), *Chevalier 5268* (P); Région de Brazzaville, Cataractes de Pool, 5 Aug 1912 (fr), *Chevalier 27746* (P); Confluent du Djoué près de Brazzaville, 29 May 1960 (fl, fr), *Descoings 5719* (P); R. Kemó, 13 Feb 1892 (fl), *Dybowski 669* (P); confluent de la Loua avec le Congo, à 15 kms de Brazzaville, 12 Aug 1963 (fl), *de Nere 1710* (P); Moyen Congo, rives de la Sangha, Feb 1920 (fl), *Pobéguin 48* (P). **DEMOCRATIC REPUBLIC OF THE CONGO.** Bas-Uele: Prov. Orientale, Terr. Bambesa, 11 Jan 1960 (fl), *Gerard 4346* (WAG); Ongo, rives de l’Uele, ca. 700 m, Nov 1945 (fl), *Germain 4362* (K, P).—Haut-Uele: Prov. Orientale, Terr. Dungu, Parc national de La Garamba, Dec 1949 (fl), *de Saeger 29 G* (K); Kibali [ca. 3°50'N, 28°30’—29°00'E], 19 Apr 1870 (fr), *Schweinfurth 2527* (K).—Kinshasa: Kinshasa, île des Mimosas, Kinshasa Territ., 3 Apr 1966 (fl), *Breyne 86* (BR); Léopoldville, Stanley-Pool, Ile des Mimosas, Léopoldville Territ., 4 Apr 1964 (fl), *Evrard 6620* (MO); Ngombe, bord du Fleuve, 16 Jul 1964 (fl, fr), *Pauwels 4583* (WAG); île des Mimosas, Kinsuka, Terr. Ngaliema, 8 Dec 1976 (fr), *Pauwels 5774* (MO, WAG).—Kongo Central: Zongo (chute), Kasangulu, 30 May 1966 (fl), *Breyne 153* (WAG); Matadi, *Chevalier 4086* (P); Matadi à Loumba, *Chevalier 4094* (P); Matadi, 11–12 Sep 1912 (fr), *Chevalier 28389* (P); route de Kimvusa à Inga (Terr. Seke-Banza), 24 Sep 1959 (fr), *Compere 496* (BR, K).—Nord-Ubangi: Banzyville (Ubangi), Jan 1931 (fl), *Lebrun 2110* (K, MO, P).—Sud-Ubangi: Prov. Équateur, Libenge, Zongo, petite île rocheuse quartzitique, au milieu des rapides de l’Ubangi, s. d. (fr), *Evrard 2574* (P); entre Libenge et Gemena, Dec 1930 (fl), *Lebrun 1792* (BR, K, MO, P, US).—Tshopo: Penghe, forêt aux bords de P’Aruwimi, 4 Feb 1914 (fl), *Bequaert 2281* (K); Lileko et Basako, 470 m, 28 Sep 1938 (fl), *Louis 11426* (K, P); Haut-Zaïre, S-Rég. Tshopo, Zone Banalia, village Panga, sur les rapides de l’Aruwimi, 28 Jan 1987 (fl), *Szafranski 1218* (WAG).—Province unknown: Without definite locality, *Smith s. n.* (BM—3 sheets). **ANGOLA.** Peco, Sumba, proximum flumen Congo, 20 m, Apr 1922 (fl), *Gossweiler 8658* (BM, K, US); Sumba, Peco, proximum flumen Zaire (Congo), 15 May 1925 (fl), *Gossweiler 8937* (B, BM, US).

*Xylopialongipetala* is a distinctive and readily identified species. The leaves are rounded at the base and usually short-acuminate, the inflorescences have long pedicels bearing persistent but thin bracts, the sepals are usually reflexed at anthesis, the petals of both whorls are long and ribbonlike, with the inner petals mostly glabrous but densely hairy inside the concave base, the staminal cone is usually higher than wide, the ovaries exceed the apex of the staminal cone, the stigmas are long, falcate, and extend beyond the aperture created by the bases of the petals (Fig. 3E), the monocarps are longitudinally ridged, and the seeds are covered by a pale gray, green, or blue sarcotesta. It occurs in riparian habitats, often on sandy soils. Associates include *Guibortiademeusei*, *Irvingiasmithii*, *Parinaricongensis*, *Uapacaguineensis*, and *U.heudelotii*.

*Xylopiakatangensis* is the species most similar to *X.longipetala*, but it has shorter and more rigid petals that are not as wide at the base, thus not producing the bulbous base of the bud and flower seen in *X.longipetala*. The sepals in *X.katangensis* are only slightly spreading at anthesis, and the inflorescences have larger numbers of flowers. The two species overlap in distribution in Nigeria, Cameroon, Gabon, and the Democratic Republic of the Congo, but *X.longipetala* is much more common within this range. Together they form a distinctive subgroup within the *X.acutiflora* group.

*Xylopialongipetala* differs markedly from plants of Uganda, Kenya, and Tanzania that had previously been identified as this species under the name *X.parviflora* (A. Rich.) Benth. (Verdcourt 1971b). The East African plants are now distinguished as *X.gracilipes*, *X.holtzii*, and *X.nilotica*. With their green fruit endocarp, orange sarcotesta of the seeds, more pubescent foliage, and small differences in floral structure (reviewed in Johnson et al. 2017), these three species belong to the *X.odoratissima* group within sect. Stenoxylopia.

Bentham (1862) published the combination *Xylopiaparviflora*, based on *Uvariaparviflora* A. Rich., citing three specimens, *Vogel s. n.* and *Barter s. n.* from the Niger and *G. Mann s. n.* from the Bagroo River, under this name. In the same publication, he accepted *X.acutiflora* (Dunal) A. Rich., and cited two specimens, *Barter s. n.* at the confluence of the Quorra and Chadda and *Chr. Smith s. n.* from the Congo. He concluded, however, “I do not feel at all confident in the real distinction between the above two species and the *Unonaoxypetala*, Dun. Anon. 114, t. 23, or *Coeloclineoxypetala*, A. DC., *l. c.*, which must also be a *Xylopia*, nor my having correctly identified our specimens; for the foliage and fruit seem to be the same in all, the differences consisting in the comparative length of the pedicels, and especially the length of the petals; but that is known to change so much in Anonaceae as the flowering advances, that, until we have good specimens in all the different stages of growth from the young bud to the fading flower, the question can scarcely be decided.”

Bentham’s confusion is understandable, because the Vogel, Barter, and Smith specimens are now all identified as *X.longipetala*, but Baillon (1864) and Oliver (1868) continued to conflate the two species, with *X.oxypetala* still taxonomically associated with them in various ways. Vallot in 1882, however, pointed out that Bentham’s description and specimen citations were not in keeping with the concept of *X.acutiflora* in the sense of its type, and Engler and Diels (1901) accepted Vallot’s conclusion.

In 1926, Exell used the name *Xylopiavallotii* Chipp as an identification for plants collected by Gossweiler from northern Angola. Hutchinson and Dalziel (1927a) supplied *Xylopiavallotii* as a *nomen novum* for *X.parviflora*, explaining (Hutchinson and Dalziel 1927b) that *Xylopiaparviflora* (A. Rich.) Benth. was a later homonym for *X.parviflora* Spruce and explicitly proposed *X.vallotii* as a *nomen novum*. While they cited the authorship as Chipp ex Exell in the latter publication, in truth Exell did not explain how the name was to be applied and authorship should be attributed to Hutchinson and Dalziel. The use of the name *Xylopiavallotii* was followed in several floras, e.g. Andrews (1950), Boutique (1951b), and Paiva (1966), but in this same time period, floras began to return to the name *X.parviflora* for the species (Tisserant and Sillans 1953, Le Thomas 1969, Verdcourt 1971b). Maas et al. (1986) argued for the valid publication of Spruce’s *Xylopiaparviflora* and pointed out that the earliest available name for African *X.parviflora* was *X.longipetala*.

### 35. Xylopia mildbraedii

Taxon classificationPlantaeMagnolialesAnnonaceae

Diels, Bot. Jahrb. Syst. 53: 444. 1915.

68ACE74B-0B6D-5DDF-AF13-59AC83689097

Fig. 42C–D

Xylopia lastoursvillii Pellegrin, Mém. Soc. Bot. France 1949: 71. 1950. Type. GABON. Ogooué-Lolo Province, région de Lastoursville, Koula motou, 13 Apr 1931, *G. Le Testu 8742* (lectotype, here designated: P! [00169128]; isolectotypes: BM! [000511051], BR! [0000008825308], LISC! [000404], OWU! P! [00169129, 00169130], WAG! [0247282, 0247283]).

#### Type.

CAMEROON. South Region, Bezirk Kribi, im Vorland mit eizeln Hügeln, bei Beson, 45 km östlich Groß-Batanga, bei 100–400 m, 22 Jul 1911, *J. Mildbraed 6055* (holotype: B! [100153148]; isotypes: HBG! [502477, 502478].

#### Description.

***Tree*** up to 9 m tall, rarely a shrub 3–4 m, d.b.h. up to 9 cm, trunk crooked, with diverging branches; bark smooth. ***Twigs*** brown, appressed-pubescent, the hairs 0.1–0.5 mm long, eventually gray-brown to orange-brown, glabrate; no nodes seen with two axillary branches. ***Leaf*** with larger blades 9.5–17.5 cm long, 3.2–5.6 cm wide, chartaceous, often discolorous but sometimes concolorous, elliptic to oblong, occasionally oblanceolate or lanceolate, apex acuminate, the acumen 8–19 mm long, base broadly cuneate, less commonly cuneate or rounded, glabrous except for the pubescent midrib adaxially, thinly and finely sericeous to glabrate abaxially, midrib impressed adaxially, raised abaxially, secondary veins indistinctly brochidodromous and slightly arcuate, 12–20 per side, diverging at 45–70° from the midrib, these and higher-order veins indistinct to slightly raised adaxially and slightly raised abaxially, rarely forming a raised reticulum on both surfaces; petiole 2–5 mm long, canaliculate, sparsely pubescent. ***Inflorescences*** axillary, 1-flowered, rarely 2-flowered from a second axillary pedicel superposed above the first, pubescent; peduncles absent; pedicels 3.7–6.4 (–8.5) mm long, 1.0–1.6 mm thick; bracts 3–4, evenly spaced along the pedicel, caducous or occasionally with uppermost persistent, 1.9-3.5 mm long, broadly triangular to semicircular, apex obtuse, acute, or apiculate; buds linear, sometime falcate, apex obtuse. ***Sepals*** slightly spreading at anthesis, 1/3-connate, 4.0–6.5 mm long, 3.6–5 mm wide, coriaceous, broadly triangular, apex acute, pubescent abaxially. ***Petals*** cream-colored *in vivo*; outer petals somewhat spreading at anthesis, 45–79 mm long, 3.6–5.5 mm wide at base, 2.2–4.1 mm wide at midpoint, fleshy, linear-ligulate, apex obtuse, with a longitudinal groove down the midline and puberulent adaxially, with a weak longitudinal ridge and sericeous, the hairs densest at the base, abaxially; inner petals more or less erect at anthesis, 35–61 mm long, 4.9–5.4 mm wide at base, 1.9–3.3 mm wide at midpoint, fleshy, linear, apex acute, base with undifferentiated margin, puberulent on both surfaces with a sericeous tuft just above the basal concavity adaxially, the hairs somewhat connivent. ***Stamens*** ca. 100; fertile stamens 1.4–1.9 mm long, narrowly oblong to clavate, apex of connective 0.3–0.5 mm long, capitate, overhanging anther thecae, papillate, anthers 11–12-locellate, filament 0.4–0.6 mm long; outer staminodes ca. 1.6 mm long, clavate, apex rounded to truncate; inner staminodes apparently absent; staminal cone ca. 2 mm in diameter, ca. 0.6 mm high, rudimentary, concealing only the very bases of the ovaries. ***Carpels*** 10–11; ovaries 1.2–1.5 mm long, oblong, densely pubescent, stigmas connivent, 4.5–6 mm long, linear, bearing a tuft of hairs at apex and pilose on the sides. ***Torus*** flat, 2.7–3.3 mm in diameter. ***Fruit*** of up to 14 sparsely pubescent to glabrate monocarps borne on a pedicel 5–7 mm long, 5–7 mm thick, glabrate; torus 11–13 mm in diameter, 7–8 mm high, irregularly depressed-globose. ***Monocarps*** with pale green or yellowish green exterior and red to pink-red endocarp *in vivo*, 4.2–6.5 cm long, 1.3–1.5 cm wide, ca. 1.1 cm thick, oblong and sometimes falciform, weakly torulose, apex with a curved beak 1 mm long, base contracted into a stipe 9–15 mm long, 3–5 mm thick, flattened and grooved longitudinally, obliquely wrinkled, verrucose; pericarp ca. 0.5 mm thick. ***Seeds*** 5–9 per monocarp, in a single row, lying oblique to long axis, 12.5–14 mm long, 6.5–8.5 mm wide, 5–7 mm thick, irregularly ellipsoid to oblong, wedge-shaped to oblong in cross-section, obliquely truncate at micropylar end, rounded at chalazal end, dark brown, smooth, shiny, raphe/antiraphe not evident, micropylar scar 3.5–4 mm long, 1.5–3 mm wide, ovate to elliptic, sometimes with a slight “beak” protruding through the center; sarcotesta frosty greenish blue, greenish white, or gray *in vivo*, sometimes visible as a white crust on dried seeds; aril absent.

#### Phenology.

Specimens with flowers have been collected in January, March, April, June, July, and December, and with fruits from December to February and in September.

#### Distribution

**Figure 46.**
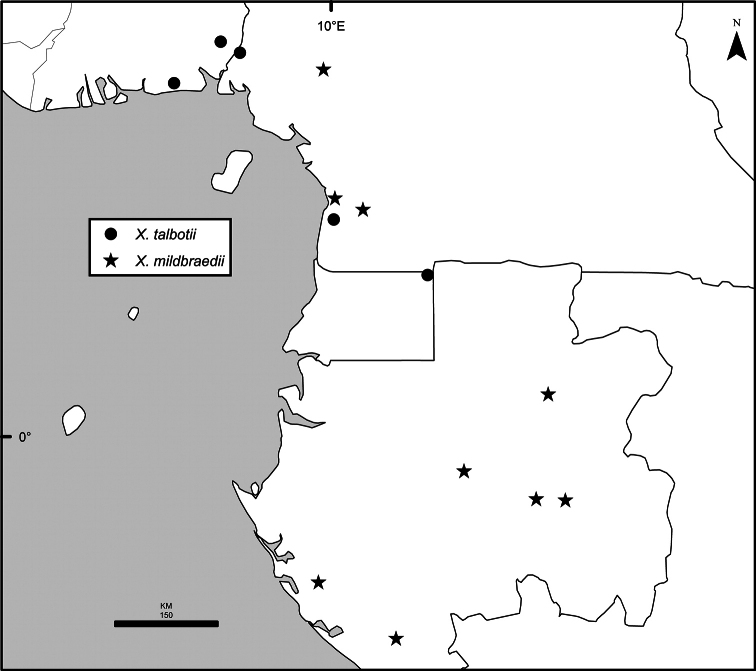
Distributions of *Xylopiatalbotii* and *X.mildbraedii*. Bolder lines represent country borders, fainter lines lakes and major rivers.

(Fig. 46). Occurs in southern Cameroon and Gabon, in primary and older secondary forest at elevations of 100–520 m.

#### Additional specimens examined.

**CAMEROON.** 20 km from Kribi, Lolodorf road, 3°00'N, 10°03'E, newly opened forest exploitation (SFIA logging road), 9 Jun 1969 (fl), *Bos 4772* (B, M, MO, WAG—3 sheets); without definite locality, *M. Elad 1253* (WAG); forêt de Bakaka, 3 km E of Eboné, a village on km 11 Nkongsamba-Loum road, 4°50'N, 9°54'E, 520 m, 13 Sep 1971 (fr), *Leeuwenberg 8362* (BR, K, MO, WAG—2 sheets). **GABON.** Nyanga: ca. 50 km SW of Doussala, 2°36'S, 10°35'E, 10 Jan 1987 (fl), *Reitsma & Reitsma 2816* (MO, NY, RSA, WAG).—Ogooué-Ivindo: Mboundou, SE Makokou, 27 Feb 1961 (fr), *Hallé 1350* (P); eastern border of Lopé-Okanda Reserve, along roads south of SEG lumber camp, west of Offoué River, 0°27'S, 11°45'E, 200 m, 15 Jan 1993 (fr), *McPherson 16070* (MO).—Ogooué-Lolo: about 40 km E of Lastoursville, ca. 0°50'S, 13°05'E, 14 Dec 1993 (fl, fr), *Breteler & Breteler-Klein 12632* (WAG—2 sheets); Lastoursville, 27 Dec 1929 (fl), *Le Testu 7824* (BM, OWU, P).—Ogooué-Maritime: Rabi, ca. 1°55'S, 9°50'E, 26 Mar 1990 (fl), *Breteler et al. 9526* (WAG).

*Xylopiamildbraedii* is a seldom-collected species, restricted in distribution. It is readily distinguished from its congeners by the petals reaching 79 mm in length, surpassing all other African *Xylopia* species. It most resembles *X.thomsonii* and *X.unguiculata* in its overall appearance, but differs in having the hairs on the young twigs short and tightly appressed, larger leaves with more numerous and slightly arcuate secondary veins, a pronounced tuft of hairs on the inside of the inner petal base, a rudimentary to absent staminal cone, and larger and thicker-walled monocarps containing larger seeds. With these differences established, it was possible to determine that “*Xylopia* species A” of Le Thomas (1969) is a fruiting collection of *X.mildbraedii*. The sterile collections *Reitsma & Reitsma 2732* and *Sosef et al. 1466*, both from Gabon, may also represent this species.

We calculated an EOO of 129,258 km^2^ and an AOO of 36 km^2^ for *Xylopiamildbraedii*. Onana and Cheek (2011) gave it a conservation assessment of Endangered, B2ab(iii).

Examination of the type material suggests that Pellegrin based the description of *Xylopialastoursvillii* primarily upon one Paris collection of *Le Testu 8742*, which bears detailed drawings of flower parts; this collection is chosen as lectotype of this name.

### 36. Xylopia monticola

Taxon classificationPlantaeMagnolialesAnnonaceae

D. M. Johnson & N. A. Murray sp. nov.

522D69CC-11D7-5102-AEA6-4A0049B2B316

urn:lsid:ipni.org:names:60476244-2

Fig. 39D–E

#### Diagnosis.

Species resembling *Xylopiaacutiflora* s. s. and *X.thomsonii* in its indument of mixed long and short hairs, and 1-flowered inflorescences with the short pedicels bearing 3 or more persistent bracts, but differing from *X.acutiflora* in the prominent abaxial leaf reticulum, the longer pedicels (5.2–8.1 mm), the outer petals (15.6–) 36–52 mm long, and the monocarps with wrinkled stipes 8–13 mm long and 3–5 seeds in a single row; from *X.thomsonii* it differs in the erect tree habit and the monocarps not exceeding 4 cm in length with proportionately longer stipes.

#### Type.

NIGERIA. Taraba [“N. E. State”] State, Sardauna Province, Kurmin Kugapa, below Kurmin Dodo below the western edge of Cabbal Wade [“Chappal Waddi”], ca. 5500 ft, 28 Feb 1975, *J. D. Chapman 3755* (holotype: K!).

#### Description.

***Tree*** up to 10 m (–20 m) tall. ***Twigs*** brown, pubescent, the hairs 0.1–1.2 mm long, eventually light grayish brown, glabrate; no nodes with two axillary branches seen. ***Leaf*** with larger blades 7.8–10.9 cm long, 2.7–5.1 cm wide, chartaceous to subcoriaceous, concolorous, lanceolate, lanceolate-oblong, elliptic to oblong or oblong-oblanceolate, apex blunt-acuminate, the acumen 4.5–16 mm long, base broadly cuneate to nearly rounded, pubescent on the midrib but otherwise glabrous adaxially, sparsely pubescent to glabrate abaxially; midrib plane adaxially, raised abaxially, secondary veins somewhat arcuate, weakly brochidodromous, 7–13 per side, diverging at 65–70° from the midrib, plane to slightly raised adaxially, raised abaxially, higher-order veins plane or slightly raised and distinct in patches adaxially, raised and reticulate abaxially; petiole 3.5–9 mm long, shallowly canaliculate, sometimes slightly flattened, sparsely pubescent to pubescent. ***Inflorescences*** axillary, 1-flowered, pubescent; pedicels 5.2–8.1 mm long, 1–1.3 mm thick; bracts 3–4, evenly spaced along pedicel, persistent, 2–3.1 mm long, ovate but sometimes bifid when torn down the middle, apex acute; buds linear, slightly falciform, apex acute. ***Sepals*** slightly spreading at anthesis, connate at base, 2.7–3.5 mm long, 2.9–3.1 mm wide, coriaceous, triangular to ovate, apex acute, pubescent abaxially. ***Petals*** creamy white *in vivo*; outer petals spreading, (15.6–) 36–52 mm long, 2.7–3.6 mm wide at base, 1.6–1.7 mm wide at midpoint, coriaceous, linear, weakly longitudinally ridged abaxially, apex acute, pubescent adaxially, sericeous abaxially; inner petals erect to spreading, (15.5–) 29–37 mm long, 2.0–2.6 mm wide at base, 0.7–0.8 mm wide at midpoint, coriaceous, filiform, apex obtuse, base with undifferentiated margin, with weak longitudinal ridges on both surfaces, pubescent on both surfaces except for glabrous base. ***Stamens*** 160–200; fertile stamens 1.8–2.2 mm long, narrowly oblong, apex of connective 0.2–0.3 mm long, shieldlike, overhanging the anther thecae, glabrous, anthers 10–12-locellate, filament ca. 0.6 mm long; outer staminodes 1.5–1.9 mm long, clavate, apex truncate or sometimes emarginate; inner staminodes 0.7–0.9 mm long, broadly clavate or oblong, apex truncate; staminal cone 1.7–1.9 mm in diameter, 1.3–1.5 mm high, completely concealing the ovaries, rim laciniate. ***Carpels*** 7–8; ovaries 1.2–1.3 mm long, lanceolate or narrowly oblong, densely pubescent, stigmas loosely connivent, 2–3.4 mm long, linear, glabrous except for a tuft of hairs at the apex. ***Torus*** flat, 1.7–2.4 mm in diameter. ***Fruit*** of up to 5 glabrate monocarps borne on a pedicel 6–8.7 mm long, 1.5–2.8 mm thick, sparsely pubescent, sometimes with bracts or sepals persistent; torus 3.1–5 mm in diameter, 1.5–2.9 mm high, depressed-globose. ***Monocarps*** with a green exterior and red endocarp *in vivo*, 3.4–4.0 cm long, 0.9–1.0 cm wide, 0.6–0.7 cm thick, narrowly oblong and slightly falciform, torulose, apex obtuse, occasionally with an offset beak 0.5–2 mm long, base contracted into a stipe 8–13 mm long, 2.1–2.6 mm thick, longitudinally wrinkled with furrows extending down stipes, verrucose; pericarp 0.2–0.4 mm thick. ***Seeds*** up to 5 per monocarp, in a single row, lying oblique to long axis, 11–13.1 mm long, 6.4–6.5 mm wide, 5.8–6.2 mm thick, oblong, broadly elliptic to circular in cross-section, truncate at micropylar end but with endostome protruding, rounded at chalazal end, tan, smooth, faintly shiny, raphe/antiraphe visible only as a faintly raised ridge, micropylar scar 2.1–3.2 mm long, 1.8–3.3 mm wide, irregularly circular; sarcotesta glaucous *in vivo*; aril absent.

#### Phenology.

Specimens with flowers have been collected in February and March, and with fruits in February, March, and May.

#### Distribution

(Fig. 34). Easternmost Nigeria and adjoining Cameroon; gallery forest along streams and in understory of foothill forests: the label of *Chapman 2739* lists as associates species of *Dracaena*, *Osmunda*, *Salix*, and *Vitex*, as well as *Phoenixreclinata*; at elevations of 650–1670 m.

#### Local name.

Kimba (Chapman and Chapman 2001).

#### Additional specimens examined.

**NIGERIA.** Adamawa: Gongola State, Ganye, Local Govt. area, well up the western slopes of Vogel Peak above Jangla, 24 Feb 1977 (fl), *Chapman 4730* (K).—Taraba: N. E. State, Mambilla Plateau, bank of the Jigawal stream about 1½ hours walk downstream from the Maisamari plantation, 28 Mar 1972 (fl), *Chapman 2739 [FHI 45180*] (K); N. E. State, Sardauna Province, Mambilla Plateau, SW foothills, ca. 3500 ft, 31 Mar 1975 (fl), *Chapman 3786* (K); Gongola State, Ngel Nyaki F./R., Mambilla Plateau, Sardauna Division, 3 Feb 1977 (fr), *Chapman 4618* (K); Gongola State, Sardauna Div., Leinde Fadale [“Linedi Faadahree”] high up on the Mambilla escarpment at the NE corner overlooking Mayo Sabere, 5000 ft, 11 Feb 1977 (fr), *Chapman 4671* (K); Gongola State, Sardauna, L. G. area, western foothills of Mambilla Plateau, Akwaizantar [“Akwaijantar”] forest, ca. 3500 ft, 2 Feb 1978 (fl), *Chapman 5186* (K). **CAMEROON.** Southwest: savanna with forest galleries near Aguosho, 10 km SSW of Akwaya, 6°18'N, 9°28'E, 1200 m, 19–20 Mar 1985 (fl, young fr), *Thomas 4558* (MO); Takamanda Forest Reserve, along footpath from Malishi to Kaluma, 06°15'N, 09°26'E, 650 m, 1 May 1987 (fr), *Thomas et al. 7400* (B, K, MO).

*Xylopiamonticola* is another segregate of *X.acutiflora* s. l., which may be distinguished by the leaves with a prominent vein reticulum abaxially, relatively long outer petals (up to 52 mm long, exceeded only by *X.mildbraedii* and *X.piratae* in the *X.acutiflora* subgroup), and relatively small monocarps with proportionally long stipes. Unlike the seeds of *X.acutiflora*, those of *X.monticola* are up to 5 per monocarp and arranged in a single row. *Xylopiamonticola* is most similar to *X.thomsonii*, which, in addition to the distinctions above, is a scandent shrub rather than a tree.

Chapman and Chapman (2001) provided habitat details for *X.monticola* in eastern Nigeria, where it was consistently an understory species of lower montane forest. At the Cabbal Wade site, the canopy dominants were *Newtoniabuchananii*, *Aubrevilleakerstingii*, *Parkiafilicoidea*, *Albizia* sp., and *Polysciasfulva*. In the Leinde Bumay forest on the Tiba Plateau, *A.kerstingii* and *P.filicoidea* were again present as canopy species, and, in addition, *Symphoniaglobulifera*. *Xylopiamonticola* was stated to be locally common at several of the Nigerian localities, but the species is not widely distributed: it has an EOO of 14,393 km^2^ and an AOO of 36 km^2^ (Table 1). Some collections were from designated forest reserves, but the current protection status of these reserves is not known. This is, however, a poorly explored area botanically.

The label of *Chapman 5186* states that the fruit is an ingredient in “yaje,” (Hausa: pepper) and local people gather the wild fruits of this species (Chapman and Chapman 2001).

### 37. Xylopia paniculata

Taxon classificationPlantaeMagnolialesAnnonaceae

Exell, J. Bot. 64 (Suppl.): 8. 1926.

269CB479-B73D-5BD1-A7DE-2582AF521780

Fig. 35M–P

#### Type.

ANGOLA [“Portuguese Congo”]. Cabinda Province, Belize, Mayumbe, 22 Feb 1917, *J. Gossweiler 6988* (holotype: BM! [photos GH, NY]; isotypes: COI! [00004886], LISC! [000321, 000322, 000323, 000324]).

#### Description.

***Tree*** up to 35 m tall, d.b.h. up to 36 cm, bole straight with stilt roots or small buttresses at the base, crown small; bark gray, fissured. ***Twigs*** brown, eventually light gray, initially loosely appressed-pubescent, the hairs 0.4–0.9 mm long, eventually glabrate; nodes often with two axillary branches. ***Leaf*** with larger blades 7.2–11.5 cm long, 1.8–3.7 cm wide, subcoriaceous or chartaceous, somewhat shining above, and slightly discolorous, narrowly elliptic, oblong-elliptic, lanceolate-oblong, or narrowly oblong, apex acuminate, the acumen 2–7 mm long, base broadly cuneate and short-decurrent on the petiole, glabrous or with a few hairs on the midrib adaxially, sparsely appressed-pubescent abaxially; midrib slightly impressed adaxially, raised abaxially, secondary veins strongly arcuate, indistinctly brochidodromous, 8–16 per side, diverging at 45–50° from the midrib, slightly raised on both surfaces, higher-order veins forming a fine raised reticulum adaxially, indistinct abaxially; petiole 2–3 mm long, canaliculate, pubescent. ***Inflorescences*** axillary, up to 32-flowered, spreading-pubescent; peduncles 2–3 per axil, highly branched, 1.0–4.5 mm long, sometimes with a longer floriferous axis emerging from among the cluster of flowers in an axil; pedicels 2 per ultimate peduncle branch, subtended by a basal bract 1.5-2 mm long, individual pedicels 2.2–5.7 mm long, 0.6–0.9 mm thick; bracts 1 or 2, attached at or distal to the pedicel midpoint, persistent, 1.5–3.0 mm long, ovate to broadly ovate, apex acute to rounded; flower buds lanceolate, apex acute. ***Sepals*** slightly spreading at anthesis, 1/8–1/3-connate, 2.1–3.0 mm long, 2.4–2.5 mm wide, coriaceous, ovate to semicircular, apex acute to obtuse, pubescent abaxially. ***Petals*** yellow-green to yellow, red at the base *in vivo*; outer petals slightly spreading at anthesis, 10–19.7 mm long, 2.3–3.6 mm wide at base, 1.0–1.7 mm wide at midpoint, slightly fleshy, linear, apex obtuse, glabrous medially in the proximal half but otherwise densely pubescent adaxially, densely pubescent abaxially; inner petals connivent at widest point of base with tips spreading at anthesis, 9.7–13.1 mm long, 2.2–2.5 mm wide at base, 0.7–1.0 mm wide at midpoint, slightly fleshy, linear, apex acute, base with undifferentiated margin, sparsely pubescent above widest point, densely pubescent in a corrugated band across the widest point, and glabrous at the very base adaxially, densely pubescent except for the glabrous base abaxially. ***Stamens*** 120–130; fertile stamens 1–1.7 mm long, capitate, apex of connective red *in vivo*, 0.3-0.4 mm long, depressed-globose, overhanging anther thecae, papillate, anthers 6–9-locellate, filament 0.3–0.5 mm long; outer staminodes 1.0–1.3 mm long, clavate, apex obtuse to rounded; inner staminodes ca. 1.1 mm long, clavate, apex rounded; staminal cone 1.1–1.5 mm in diameter, 0.3–0.6 mm high, concealing the bases of the ovaries, rim laciniate. ***Carpels*** 3–6; ovaries 0.9–1.1 mm long, narrowly oblong, densely pubescent, stigmas connivent, 2.5–2.7 mm long, filiform, sparsely pubescent along entire length. ***Torus*** flat, 1.5–2.0 mm in diameter. ***Fruit*** pedicels and torus unknown. ***Monocarps*** with green exterior [silvery brown ex Letouzey] and carmine, red, or pink-red endocarp *in vivo*, 5.6–8.5 cm long, 3.1–4 cm wide, ca. 2.7 cm thick, oblong or obovoid, not torulose, apex rounded, base sessile, longitudinally ridged and wrinkled, transversely pleated and grainy, glabrate; pericarp ca. 3 mm thick. ***Seeds*** 3–4 per monocarp, in a single row, perpendicular to long axis, 21–21.6 mm long, 15.7–17.3 mm wide, 9.5–11.0 mm thick, flattened-ellipsoid, elliptic in cross-section, truncate at micropylar end, rounded at chalazal end, brown to black, smooth, dull, raphe/antriraphe visible as a flat band encircling the seed, micropylar scar 6–8 mm long, 4.5–6 mm wide, elliptic to circular; sarcotesta grayish blue or greenish blue, waxy *in vivo*; aril absent.

#### Phenology.

Specimens with flowers have been collected in February and from June to August, and with fruit in June.

#### Distribution

(Fig. 36). Occurs from southern Cameroon south to the Cabinda Province of Angola, in low elevation primary rainforest.

#### Local name.

Boëso (Bakota, *Hallé & Le Thomas 379*).

#### Additional specimens examined.

**CAMEROON.** East: A 23 km a l’Ouest de Masea (village situé à 50 km au SSW de Yokadouma), 4 Jul 1963 (fl), *Letouzey 5402* (P—4 sheets). **GABON.** Ogooué-Ivindo: 25 km SE de Mékambo 25 km SE, 7 Aug 1966 (fl), *Hallé & Le Thomas 379* (P).—Nyanga: Chantier CEB, Inventory, ca. 50 km SW of Doussala, 2°36'S, 10°35'E, 14 Jun 1985 (fl, fr), *Reitsma & Reitsma 1163* (BISH, MO, NY, RSA, WAG).

*Xylopiapaniculata*, as its specific epithet suggests, has highly branched inflorescences of up to 32 flowers, a characteristic unique among African species. It also has one of the largest monocarps of any African *Xylopia* species, and the largest seeds. The other species tending to have large numbers of flowers per inflorescence are *X.calva*, *X.katangensis*, *X.phloiodora*, and *X.villosa*, but all of these species have 12 flowers or fewer. In addition, *X.calva* has much larger leaves and broader petals, *X.phloiodora* usually has larger leaves with pinkish-red coloration on the secondary veins, longer petioles, longer petals, a larger and more conspicuous staminal cone, and smooth but lenticellate monocarps, and *X.villosa* has leaves that are densely sericeous abaxially.

*Xylopiapaniculata* was included in Le Thomas (1969) as “*Xylopia* sp. B,” on the basis of *Hallé & Le Thomas 379*, which is fragmentary although with abundant disarticulated flower material. The single leaf is at the small end of the range of variation for this species, and the petals are slightly larger than those of the other collections, but nonetheless match in qualitative features, such as the shape of the inner petals.

We calculated *Xylopiapaniculata* to have an EOO of 105,473 km^2^ and an AOO of 16 km^2^, a wide disparity between the two measurements seen for several Congo Subregion lowland forest species. The most recent collection is from 1985. The label of the collection from Cameroon describes that plant as rare in ombrophilous forest of *Baillonellatoxisperma* and *Pentaclethramacrophylla*.

### 38. Xylopia phloiodora

Taxon classificationPlantaeMagnolialesAnnonaceae

Mildbraed, Notizbl. Bot. Gart. Berlin-Dahlem 8: 55–56. 1921.

877E8441-DB4C-5CD4-B6F1-4BC604E8EF2D

Fig. 35A–L

#### Type.

CAMEROON. South Region, zwischen Bipindi und Ebolowa bei Malakat, Dec 1913, *J. Mildbraed 7592* (lectotype, here designated: B! [100153152]; isolectotype: K! [000199053]).

#### Description.

***Tree*** up to 38 m tall, d.b.h. up to 80 cm, bole straight, cylindrical, sometimes with small buttresses up to 1.5 high extending up to 50 cm from the base, rarely with stilt roots, secondary branches radiating horizontally from trunk to form a small crown; bark yellowish gray to light gray, finely fissured and brown-punctate. ***Twigs*** brown, eventually light gray, initially densely appressed-pubescent, the hairs 0.1–0.4 mm long, soon glabrate; nodes occasionally with two axillary branches, young shoots often with flattened conduplicate leaves. ***Leaf*** with larger blades 5.7–17.2 cm long, 1.9–5.9 cm wide, chartaceous or subcoriaceous, concolorous or slightly discolorous, elliptic or oblong-elliptic to lanceolate, apex obtuse to gradually acuminate, base broadly cuneate, rounded, or rarely subcordate, sometimes slightly oblique, short-decurrent on petiole, glabrous or with a few hairs along the midrib adaxially, initially golden-sericeous but soon sparsely appressed-pubescent abaxially; midrib slight impressed to slightly raised adaxially, raised abaxially, secondary veins arcuate, indistinctly brochidodromous, often drying pinkish red and contrasting with the gray of the lamina, 10–16 per side, diverging at 40–60° from the midrib, these and higher-order veins slightly raised on both surfaces; petiole 3.5–8 mm long, flattened to canaliculate, pubescent. ***Inflorescences*** axillary or from the axils of fallen leaves, 1–10-flowered, commonly 2–3-flowered, pubescent; peduncle 1 per axil, 2–4 mm long; pedicels 3 per peduncle or not pedunculate, 2.5–5.5 mm long, 0.9–1.0 mm thick; bracts 2, both attached near the pedicel midpoint, the lower caducous and the upper persistent, 1.5–2.5 mm long, ovate, semicircular, or rounded, apex obtuse to bifid; buds linear-lanceolate to linear-oblong, often falcate, apex obtuse. ***Sepals*** slightly spreading at anthesis, 1/5–2/3 connate, 2–3.6 mm long, 2.5–2.8 mm wide, coriaceous, ovate to broadly ovate or triangular, apex obtuse to acute, appressed-pubescent abaxially. ***Petals*** cream-colored to pale yellow with a blotch of purple at the base *in vivo*; outer petals spreading at anthesis, 19–23 mm long, 3–4.3 mm wide at base, 1.7–2 mm wide at midpoint, fleshy, linear-lanceolate, apex obtuse, densely puberulent except for the glabrous base adaxially, sericeous to the base abaxially; inner petals spreading, possibly bent outward at anthesis, 15.5–21 mm long, 2–3 mm wide, 0.6–0.8 mm wide at midpoint, fleshy, linear, apex acute, base with undifferentiated margin, puberulent on both surfaces except for glabrous base. ***Stamens*** ca. 120; fertile stamens 1.3–2 mm long, narrowly oblong, apex of connective 0.1–0.3 mm long, shieldlike or slightly hemispheric, overhanging anther thecae, glabrous, anthers 20–24-locellate, filament 0.4–0.5 mm long; outer staminodes 1.3–1.6 mm long, broadly clavate to narrowly oblong, apex obtuse, rounded, bifid, or truncate; inner staminodes 1.1–1.3 mm long, broadly clavate, apex truncate; staminal cone 1.2–1.6 mm in diameter, 1.1–1.5 mm high, completely concealing the ovaries, rim even. ***Carpels*** 5–8; ovaries 1.1–2 mm long, narrowly ellipsoid, pubescent, stigmas connivent, 2.1–3.0 mm long, linear, sometimes widened at the midpoint, glabrous, sometimes warty, rarely with a few hairs at the apex. ***Torus*** flat, 2–2.5 mm in diameter. ***Fruit*** of up to 9 glabrate monocarps borne on a pedicel 5–16 mm long, 5–8 mm thick, glabrate; torus 11–19 mm in diameter, 6–12 mm high, depressed-globose. ***Monocarps*** with greenish purple, dark brown, brownish gray, reddish brown, or cinnamon-colored exterior and light pink endocarp *in vivo*, 2.7–4.3 cm long, 1.7–3.1 cm wide, 1.7–2.4 cm thick, ovoid, oblong, or broadly ellipsoid, not torulose, apex rounded, base sessile but slightly narrowed, slightly rugose, usually conspicuously lenticellate; pericarp 1.5–4 mm thick. ***Seeds*** 7–12 per monocarp, in two rows, lying nearly perpendicular to long axis, 16–21 mm long, 7–10 mm wide, 4.5–7 mm thick, oblong, elliptic to wedge-shaped in cross-section, truncate at micropylar end, rounded at chalazal end, brown to blackish brown, smooth, glossy or dull, raphe/antiraphe not evident, micropylar scar 1.7–2.5 mm long, 1.6–2.7 mm wide, oblong, obovate, or triangular; sarcotesta orange, fleshy *in vivo*, sometimes visible as a white crust on dried seeds; aril absent.

#### Phenology.

Specimens with flowers have been collected in all months of the year except October, and with fruits in January, and from April to September.

#### Distribution

(Fig. 36). Occurs from south-central Nigeria east to northeastern Democratic Republic of the Congo and south to southern Republic of the Congo and southeastern Democratic Republic of the Congo. It has also been reported from Angola (Paiva 2008) but it is not possible from the specimen images (LISC 16349, 16350) to confirm the identification. *Xylopiaphloiodora* has been collected from a variety of lowland moist forest habitats, at elevations of 200–900 m.

#### Local names.

Aghako (*Kennedy 2612*), bendjo (*Corbisier-Baland 1627*), bompaie bo fufow (Turumbu, *Louis 2477*), ddong-éli (Fang, *Duboislouveau 903*), mbeb (Balavele, *Grison FG 22/RC 827*), molo-nzange (Lissongo, *Tisserant 955*), molo-mosome (Lissongo, *Tisserant 1113*), molo-nyama (Lissongo, *Tisserant 1138*), molonzangue (Issongo, *Guigonis 2320*), odjobbo (Bulu, Mildbraed (1921)), odzobi (*Service Forestier du Cameroun 67*), ohunegbo (*Kennedy 2612*), sange (Bibaya, *Letouzey & Villiers 10418*; Kibila, *Hart TH 1177*; *Hart 1654*), sangue (Baya & Babinga, *Guigonis 3078*), uyen (*Kennedy 2612*).

#### Additional specimens examined.

**NIGERIA.** “S. Nigeria” [without definite locality, probably Sapoba], 2 Apr 1945 (fl), *Kennedy 2612* (A, BR, F, MO, PR, US, YF). **CAMEROON.** Est, Ndakan, Sango River, 02°22'N, 16°09'E, 10 May 1988 (st), *Gentry et al. 62649* (MO); près Ngola (30 km à l’Est de Yokadouma, 11 May 1963 (fl, fr), *Letouzey 5027* (P—2 sheets; mistakenly cited as *Letouzey 5057* in Le Thomas (1969)); a 24 km à l’Ouest de Masea (village situé à 50 km au SSW de Yokadouma, 4 Jul 1963 (fl), *Letouzey 5401* (P); a 25 km environ à l’ENE de Mikel village siuté à 85 km au N de Moloundou au route de Yokadouma, 24 Feb 1971 (fl), *Letouzey & Villiers 10418* (K, P); près Nkongong II sur axe Lomie-Ngoila-Souanke, à 15 km SSW de Ngoila, 22 Feb 1973 (st), *Letouzey 12026* (BR, K); 20 km ENE of Moloundou-Nguilili chantier, 10 Mar 1973 (fl), *Mbenkum 310* (P); Bezirk Kribi, Vorland mit einzeln Hügeln bei Adjab, 35 km östlich Groß-Batanga, ca. 100 m, Jul 1911 (fr), *Mildbraed 6090* (HBG); Reserve d’Ototomo prés Yaoundé, s. d. (fr), *Service Forestier du Cameroun 67* (P); bank Nyong River, near the new bridge, about 65 km SW of Eséka, alt. ca. 200 m, 16 Jul 1964 (buds), *de Wilde & de Wilde-Duyfjes 2838A* (BR, K, MO, WAG—2 sheets), *2838B* (WAG—2 sheets); Bipinde or Yaunde Nknambe, 1907 (fl), *Zenker & Staudt 3314* (A, B, BM—2 sheets, G-81404, G-81405 as to leaves only, K, L, MO). **CENTRAL AFRICAN REPUBLIC.** Sangha Economic Prefecture, Ndakan Gorilla Study Area, 2°20'N, 16°09'E, 21 Jun 1988 (fr), *Fay & Harris 8457* (MO); Grima, 25 Dec 1961 (fl), *Guigonis 2320* (P); Sekamba Route 6° parallèle, 16 May 1964 (fl), *Guigonis 3078* (P); Région de Mbaiki et Boukoko, 5 Jun 1948 (fl), *Tisserant 955* (BM, P); Région de Mbaïki, Station Central de Boukoko, 27 Aug 1948 (fl), *Tisserant 1113* (BM); 9 Sep 1948 (fr), *Tisserant 1138* (BM, P). **GABON.** Estuaire: Liby, 25 Feb 1952 (fl, yg fr), *Duboislouveau 903 S. R. F.* (P).—Ogooué-Ivindo: Bélinga Mines de Fer, 20 Jul 1966 (fl), *Hallé & Le Thomas 104* (P); Bélinga, Mines de Fer, 4 km on the road to Mvadi, 900 m, 1°05'N, 13°12'E, 5 Nov 2005 (fr), *Sosef et al. 2218* (OWU); E border of Lopé-Okanda Reserve, along roads S of SEG lumber camp, W of Offoué River, 0°27'S, 11°45'E, 14 Jan 1993 (fl), *McPherson 16058* (OWU).—Woleu-Ntem: ca. 25 km WSW of Mitzic [“Mintsic”], 7 Feb 1987 (st), 0°44'N, 11°22'E, *Reitsma & Reitsma 2900* (MO, NY, WAG). **REPUBLIC OF THE CONGO.** Ouesso, 25 Apr 1971 (fl), *Grison FG 22/RC 827* (P); Ile M’Bamou, forêt et prairie à 10 km environ de Moutou ya N’Gombé—Brazzaville, 16 May 1967 (fl), *Sita 1634* (P). **DEMOCRATIC REPUBLIC OF THE CONGO.** Équateur: Eala, 3 Aug 1932 (fl), *Corbisier-Baland 1627* (BR); Bikoro, 31 Mar 1958 (fl, yr fr), *Evrard 3849* (K); Wendji [near Coquilhatville], Aug 1930 (fl), *Lebrun 1014* (BM, K, MO, NY, P, RSA, US).—Ituri: Epulu, Zone: Mambasa (Ituri), 1°25'N, 28°35'E, 750 m, 25 Jun 1986 (fl), *Hart 628* (BR, MO); Haut-Zaire: Zone de Mambasa (Ituri Forest), Epulu, 1°25'N, 28°35'E, 750 m, 6 Jul 1991 (fl), *Hart TH 1177* (BR, K); Zone de Mambasa (Ituri Forest), Afarama, 1°33'N, 28°23'E, *Hart 1654* (K, MO).—Kasai Oriental: camp de Kifuku, 27 Jan 1949 (fr), *Michelson 881* (K, P).—Maniema: Secteur Bangengele, Parc National Proposé de la Lomami, ca. 6.2 km au NNE de Katopa, 02°42'00"S, 025°08'10"E, 470 m, 19 Apr 2015 (fr), *Gereau et al. 7565* (MO) .—Tshopo: Yangambi, 12 Jan 1936 (bud), *Louis 1007* (BM, NY); Yangambi, km 8, 400 de la route de Ngazi, à l’E (abatages pour extensions Elaeis), 20 Aug 1936 (fr), *Louis 2477* (BR); Yangambi, ca. 470 m, 19 May 1937 (old fl), *Louis 3909* (NY, RSA); Yangambi, ca. 470 m, 25 Dec 1937 (fl), *Louis 7220* (K, MO, US, WAG); *7918* (FI-T, MO), 27 Jan 1939 (fl), *Louis 13430* (K, MO, NY, US); Kisangani [“Stanleyville”], 12 Mar 1939 (fl), *van der Meiren 70* (K, P).

*Xylopiaphloiodora* is most easily identified in fruit. Its monocarps are among the largest of the African species, 2.7–4.3 by 1.7–3.1 cm, brown, oblong, sessile, glabrate, and strongly lenticellate, and lacking external ridges at maturity. The leaves, with a fine raised reticulum on the adaxial surface, are variable in size and shape, but have strongly arcuate secondary veins often drying a pinkish red color that contrasts with the grayish background color of the lamina. The bark, as noted in the specific epithet, is aromatic when cut, a trait frequently noted by collectors.

Specimens in flower, especially those with smaller and narrower leaves, are more difficult to identify. The pedicels are short, with a persistent bract closely subtending the rigid calyx. The petals are narrow and densely pubescent on both surfaces. The anthers have more locelli, 20–24, than those of any other African species, and the staminal cone is dome-shaped with an even rim, completely enclosing the ovaries (Fig. 35B).

*Xylopiaphloiodora* most closely resembles *X.paniculata*, but that species has shorter petioles, more pronounced branching of the inflorescence, shorter petals, and a shorter more open staminal cone with a laciniate rim. *Xylopiacupularis* is also similar to *X.phloiodora*, but it has longer pedicels with caducous bracts and larger numbers of carpels. In the sum of its characters, *X.phloiodora* belongs to the group of African *Xylopia* species with large thick-walled monocarps and large seeds with light green sarcotestas, but the labels of two specimens, *Louis 2477* and *Harris 2622*, report the seeds to be orange. The fallen monocarps of *Harris 2622* were all split into three segments, as happens in other species of this group, such as *X.hypolampra*.

The habitat of *Xylopiaphloiodora* is not well defined. Reported associates in the southwestern Central African Republic are *Eribromaoblonga* and species of *Celtis*, *Entadophragma*, *Guibortia*, *Myrianthus*, *Tetrapleura*, and *Megaphrynium*. Seeds are reported to be taken by hornbills (*Harris 2622*) and the fruits fed upon by monkeys (*Fay & Harris 8457*). The conservation status of the species was assessed by Onana and Cheek (2011), who proposed Not Threatened (NT) status. The range of the species covers a large area but it was only represented in this study by 42 collections.

Mildbraed (1921) based the name *Xylopiaphloiodora* on *Mildbraed 7592* and *Mildbraed 6090*, neither of which was designated as a type. The former agrees well with the protologue and was identified as the type of this name by Le Thomas; we formally designated it here as the lectotype.

### 39. Xylopia piratae

Taxon classificationPlantaeMagnolialesAnnonaceae

D. M. Johnson & N. A. Murray sp. nov.

2C3C1678-A39D-519E-800E-14295D8C6512

urn:lsid:ipni.org:names:60476245-2

Figs 3F , 33H–J

#### Diagnosis.

Species resembling *Xylopiathomsonii* in the twig indument consisting of both short and long erect hairs, the one-flowered inflorescence with multiple overlapping bracts on the short pedicel, and the linear petals, but differing in the more strongly lianescent habit with ultimate branches departing at right angles to the twig that bears them, the leaves only 4.1–11.3 cm long, the outer petals reaching 73 mm in length, carpels only 4–9 per flower, and monocarps only 0.5–0.7 cm in width with a more pronounced beak.

#### Type.

IVORY COAST. Grand Bassam Department, forêt de l’Abouabou, between Abidjan and Grand Bassam, 2 m, 6 Jan 1959, *A. J. M. Leeuwenberg 2365* (holotype: WAG! [0005952]; isotypes: K! P!).

#### Description.

***Liana*** extending to 20 m, or occasionally a shrub, the ultimate branches often departing at right angles to the twig that bears them and slightly thickened at the base. ***Twigs*** brown, pubescent, the hairs 0.5–1.2 mm long, soon dark brown to gray-brown, glabrate, sometimes with bark exfoliating; no nodes with two axillary branches seen. ***Leaf*** with larger blades 4.1–11.3 cm long, 1.9–3.9 cm wide, chartaceous, concolorous to discolorous, elliptic, oblong, oblong-oblanceolate, or lanceolate, apex acuminate, the acumen 2–11 mm long, base broadly cuneate to rounded, pubescent on the midrib but otherwise glabrous adaxially, finely appressed-pubescent to glabrate abaxially; midrib plane adaxially, raised abaxially, secondary veins weakly brochidodromous, 8–15 per side, diverging at 65–70° from the midrib, these and higher-order veins indistinct to raised adaxially, slightly raised to raised abaxially; petiole 1.2–3.5 mm long, shallowly canaliculate, sparsely pubescent to glabrate. ***Inflorescences*** axillary, 1-flowered, pubescent; pedicels 3.2–4.5 mm long, 0.8–1.2 mm thick; bracts 4–5, imbricate over length of pedicel, persistent, 1.7–3 mm long, ovate to circular, apex acute to rounded; buds linear-lanceolate, somewhat falciform, apex acute. ***Sepals*** slightly spreading at anthesis, 1/4–1/3-connate, 2.4–3.1 mm long, 2.2–3.0 mm wide, chartaceous, ovate to broadly triangular, apex acute to acuminate, sericeous abaxially. ***Petals*** cream-colored to white *in vivo*; outer petals spreading but curved inward toward the apices at anthesis, 24.3–73 mm long, 2.6–4 mm wide at base, 1.0–1.5 mm wide at midpoint, subcoriaceous, linear, apex acute, puberulent but becoming glabrous and warty in the proximal 1/3–1/2 adaxially, puberulent abaxially; inner petals spreading but curved inward toward the apices at anthesis, 25.8–48 mm long, 2.1–3.4 mm wide at base, 0.6–1.0 mm wide at midpoint, subcoriaceous, linear, apex acute, base with undifferentiated margin, puberulent, glabrous in the proximal 1/3 adaxially, sparsely puberulent abaxially. ***Stamens*** ca. 100; fertile stamens 0.9–1.5 mm long, narrowly oblong, apex of connective ca. 0.2 mm long, shieldlike to dome-shaped, overhanging the anther thecae, glabrous, anthers ca. 12–13-locellate, filament 0.2–0.4 mm long; outer staminodes 1.3–1.6 mm long, clavate, apex obtuse to obliquely truncate; inner staminodes 0.8–1 mm long, oblong to clavate, apex truncate; staminal cone 1.7–2.0 mm in diameter, 0.6–1.1 mm high, concealing lower half of the ovaries, rim laciniate. ***Carpels*** 4–9; ovaries 1.0–1.4 mm long, narrowly oblong, densely pubescent, stigmas connivent, 3.4–4 mm long, filiform, apex acute, with short tuft of hairs at apex but otherwise glabrous. ***Torus*** flat, ca. 2.4 mm in diameter. ***Fruit*** of up to 6 sparsely pubescent to glabrate monocarps borne on a pedicel 6.5–6.8 mm long, 2.2–2.7 mm thick, sparsely pubescent to glabrate; torus ca. 5.5 mm in diameter, 3.5 mm high, depressed-globose. ***Monocarps*** with a green, sometimes purple-tinged, exterior and red endocarp *in vivo*, 3.1–6.6 cm long, 0.5–0.7 cm wide, ca. 0.7 cm thick, narrowly oblong and slightly falciform, torulose, apex with a curved beak 1.5–3 mm long or occasionally rounded, base contracted into a stipe 5–12 mm long, 1.2–3.2 mm thick, verrucose; pericarp ca. 0.4 mm thick. ***Seeds*** up to 9 per monocarp, in a single row, lying oblique to long axis, 9–10.9 mm long, 5.5–7.5 mm wide, 5.6–6.1 mm thick, ellipsoid, broadly elliptic in cross-section, obliquely truncate at micropylar end, rounded at chalazal end, light brown, smooth, dull, raphe/antiraphe not evident, micropylar scar 1.7–5 mm long, 1.4–3.5 mm wide, elliptic, oblong, or circular with the endostome protruding; sarcotesta pale gray to green *in vivo*; aril absent.

#### Phenology.

Specimens with flowers have been collected from October to February and in April, May, July, and August; specimens with fruits have been collected from November to January and in July and August.

#### Distribution

(Fig. 34). Occurs from southern Ivory Coast east to southwestern Ghana, growing in coastal thickets and savanna, sometimes in secondary forest, at elevations from sea level up to 50 m.

#### Additional specimens examined.

**IVORY COAST.** Bords de la lagune Ebrié, 1932 (fr), *Aubréville 1537* (K); Km 25 Sassandra-Gagnoa, ca. 5.5N, 6.5W, 30 Oct 1968 (fl), *Breteler 5856* (K, MO, WAG); near Grand Bassam, NW along Rd. to Aboisso, ca. 5°13'N, 3°43'W, 12 Nov 1968 (fl), *Breteler 5983* (B, K, M, MO, NY, U, WAG); near Maféré, 5°23'N, 3°05'W, 23 Apr 1974 (fl), *Breteler 7445* (MO); Banco Forest Reserve, near Abidjan, 50 m, 24 Jan 1970 (fl), *de Koning 84* (WAG—2 sheets); Banco Forest Reserve, 22 Dec 1972 (fl), *de Koning 983* (WAG); 25 Apr 1973 (fl), *de Koning 1555* (WAG); 10 Dec 1974 (fl), *de Koning 4999* (WAG—2 sheets), 10 Dec 1974 (bud, fr), *de Koning 5000* (WAG); Abidjan, Banco Forest Reserve, 5 May 1976 (fl), *de Koning 6856* (WAG—2 sheets); forêt de l’Abouabou, between Abidjan and Grand Bassam, 5°14'N, 3°33'W, 2 m, 1 Aug 1970 (fl), *Leeuwenberg 8022* (B, K, MO, P, U); about 10 km W of Jacqueville, island Aladian, 5°11'N, 4°32'W, 0 m, 3 Aug 1970 (yg fr), *Leeuwenberg 8089* (K); E of Tabou, Yokobo, 4°26'N, 7°22'W, 9 Nov 1981 (fl), *Leeuwenberg 12304* (WAG); Ile Bonbay, près Adiopodoumé, 29 Aug 1955 (fr), *Nozeran s. n.* (BR, P); savane d’Abouabou, between Abidjan and Grand Bassam, 28 Nov 1963 (fl), *Oldeman 682* (K, P); Arboretum, forêt du Banco (cultiv.), 6 Dec 1956 (fl), *de Wilde 982* (WAG); I. R. H. O. (Cocos cultures) ca. 20 km W of Grand Bassam, near the beach on sandy soil between Port Bouet and G. Bassam, 1 Jul 1963 (fl), *de Wilde 354* (K, P), 1 Jul 1963 (fr), *de Wilde 356* (K, P); ca. 5 km SE of O. R. S. T. O. M., Ile Boulay, beyond Lagune Ebrié, 22 Jul 1963 (fr), *de Wilde 497* (BR, K); forêt d’Andouin, 24 Aug 1955 (fr), *de Wit 7999* (WAG). **GHANA.** Princes [Town], 9 Jan 1939 (fl, fr), *Akpabla 767* (K—2 sheets); Atwabo, W. Province, Feb 1934 (fl), *Irvine 2299* (K); Western, Bia Reserves, along borderline between Bia National Parc and Bia Production Reserve, near Camp 15, 6°32'42"N, 3°02'00"W, 13 Nov 1993 (fr), *Jongkind et al. 1301* (MO).

*Xylopiapiratae* bears pure white flowers with slender petals that, at 70 mm, are among the longest known in the genus. The climbing habit of the plants is more strongly developed than in any other species of *Xylopia*: label descriptions invariably refer to the climbing or scrambling growth form, and on the label of *de Koning 983* the plant is described as reaching a length of 20 m. Plants identified as *X.acutiflora* by Hall and Swaine (1981) from forest plots in southwestern Ghana and described as “a woody climber with leafy shoots of limited growth inserted approximately at right angles to the main stem, as in *Uvariaovata*,” probably represent this species rather than *X.acutiflora*. In addition, there is a tendency in *X.piratae* for the shoots inserted at right angles to have a slight thickening where they join the supporting branch; *Xylopiadinklagei* shows a similar tendency. The sweet scent of the flowers has been noted by many collectors; *Leeuwenberg 12304* added that the flowers were fragrant at 1700 h. This species is named in memory of Celine Pirat, who photographed the plants (Fig. 3F) on Ile Assoko, Ivory Coast, on 10 May 2014.

*Xylopiapiratae* most closely resembles *X.thomsonii* from central Africa. Both species have a tendency for the branches to become lianescent, although this is more extreme in *X.piratae*. Both have a mixture of both long and short erect hairs persistent on the twigs. The petals of *X.thomsonii*, while they do not reach the length of those of *X.piratae*, are long for this species subgroup but broader. The liana habit and extremely long petals combine to distinguish *X.piratae* from both *X.acutiflora* and *X.dinklagei*, the other two species of the *X.acutiflora* subgroup found in West Africa.

Three specimens from southern Togo may represent *Xylopiapiratae*: *Schäfer 7577* (GH, MO, WAG—2 sheets), *de Wit & Morton A2895* (WAG), and *Ern 2710* (B—2 sheets). The specimens are incomplete, and no habitat information is given for these specimens.

*Xylopiapiratae* is one of three *Xylopia* species endemic to the Guinea Coast region of West Africa, all of them with relatively narrow distributions and all belonging to the *Xylopiaacutiflora* subgroup. For *Xylopiapiratae*, we calculated an EOO of 31,476 km^2^ and an AOO of 64 km^2^. Its narrow coastal savanna distribution may make it vulnerable to habitat loss. It may be somewhat tolerant of disturbance, however, as several collections are from secondary forest habitat and the collection *Leeuwenberg 8022*, bearing flowers, was taken from a shoot regenerating from a stump in *Alchorneacordifolia* thicket.

### 40. Xylopia pynaertii

Taxon classificationPlantaeMagnolialesAnnonaceae

De Wildeman, Ann. Mus. Congo, Sér. 5, Bot. 3: 79. 1909.

F1F1F46E-8207-542F-9817-EC750A62DFB5

Fig. 42A–B

#### Type.

DEMOCRATIC REPUBLIC OF THE CONGO [“Belgian Congo”]. Équateur Province, Eala, 15 Oct 1906, *L. A. E. J. Pynaert 567* (lectotype, here designated: BR!; isolectotypes: BR! [0000008825339, 0000008825346]).

#### Description.

***Tree*** up to 35 m tall, d.b.h. up to 40 cm, bole forming buttresses ca. 0.5 m high and extending up to 50 cm from the trunk at the base; upper bark red, rough, scaly. ***Twigs*** brown to dark brown, pubescent, hairs 0.4–1.5 mm long, at length light brownish gray, glabrate, bark somewhat exfoliating on older twigs; nodes occasionally with two axillary branches. ***Leaf*** with larger blades 3.6–8.7 cm long, 1.2–2.3 cm wide, chartaceous to subcoriaceous, discolorous or occasionally concolorous, lanceolate, ovate, or elliptic, apex obtuse to acute, base cuneate to broadly cuneate, glabrous except for the pubescent midrib adaxially, sparsely but uniformly appressed-pubescent abaxially; midrib slightly impressed adaxially, raised abaxially, secondary veins indistinctly brochidodromous, 8–13 per side, diverging at 60–70° from the midrib, these and higher-order veins indistinct adaxially, slightly raised abaxially; petiole 1–2.5 mm long, shallowly canaliculate to semi-terete, pubescent. ***Inflorescences*** axillary, 1–2-flowered, pubescent; peduncle 1 per axil, 0.5–0.8 mm long, or absent; pedicels 2 per peduncle, 4–5.4 mm long, ca. 0.9 mm thick; bracts 2, evenly spaced along pedicel, persistent or caducous, 1–1.4 mm long, semicircular, apex rounded; buds linear-lanceolate, sometimes slightly falcate, apex acute. ***Sepals*** slightly spreading to spreading at anthesis, 1/4–1/2 connate, 1.5–2.7 mm long, 2.1–2.4 mm wide, coriaceous, ovate to triangular, apex acute, pubescent abaxially. ***Petals*** yellowish white *in vivo*; outer petals slightly spreading at anthesis, 15.2–20.5 mm long, 2.4–3.0 mm wide at base, 1.0–1.5 mm wide at midpoint, coriaceous, linear, apex acute to obtuse, densely puberulent but becoming glabrous in the medial region toward the base adaxially, yellow-brown sericeous except for glabrous base abaxially; inner petals with position at anthesis uncertain, 12.3–16.8 mm long, 2.2–3.2 mm wide at base, 0.7–1.1 mm wide at midpoint, coriaceous, linear, apex acute, densely puberulent on both surfaces except for glabrous base. ***Stamens*** ca. 140; fertile stamens 0.8–1.3 mm long, oblong, apex of connective 0.1–0.3 mm long, shieldlike, overhanging anther thecae, minutely papillate, anther locules 4–9-locellate, filament 0.2–0.5 mm long; outer staminodes 1.1–1.3 mm long, oblong, apex obtuse to obliquely truncate; inner staminodes ca. 0.9 mm long, oblong, apex truncate; staminal cone 1.7–1.9 mm in diameter, 0.7–0.9 mm high, concealing all but the apices of the ovaries, rim laciniate. ***Carpels*** 9–11; ovaries ca. 1.1 mm long, narrowly oblong, pubescent, stigmas connivent, 2.5–3.8 mm long, linear, glabrous except for tuft of hairs at apex. ***Torus*** flat, 1.8–2.1 mm in diameter. ***Fruit*** of up to 8 glabrate monocarps borne on a pedicel 3.2–24 mm long, 2.3–3.4 mm thick, the pedicel occasionally adnate to a short dead branch, sparsely pubescent; torus 5.5–14 mm in diameter, 3.7–7 mm high, irregularly globose. ***Monocarps*** with green exterior and red endocarp *in vivo*, 2.6–4.8 cm long, 1.3–1.8 cm wide and thick, oblong to obovoid, apex rounded, base contracted into a stipe 1.5–8 mm long, 3.5–6 mm thick, or monocarp sessile, longitudinally ridged, slightly and obliquely wrinkled, verrucose; pericarp 0.2–1 mm thick, fibrous. ***Seeds*** 5–6 per monocarp, in two rows, lying perpendicular to long axis, 9.3–13.1 mm long, 6.2–8.8 mm wide, 4.2–6.7 mm thick, ellipsoid, wedge-shaped or flattened-ellipsoid in cross-section, obliquely truncate at micropylar end, rounded at chalazal end, brown, smooth, dull or slightly shiny, raphe/antiraphe not evident, micropylar scar 3–4.9 mm long, 1.5–4.6 mm wide, roughly circular or transversely elliptic; sarcotesta white to grayish blue *in vivo*, sometimes visible as a white crust on dried seeds; aril absent.

#### Phenology.

The collections with flowers are from March, October, and November, and with fruits from April, May, October, and December.

#### Distribution

**Figure 47.**
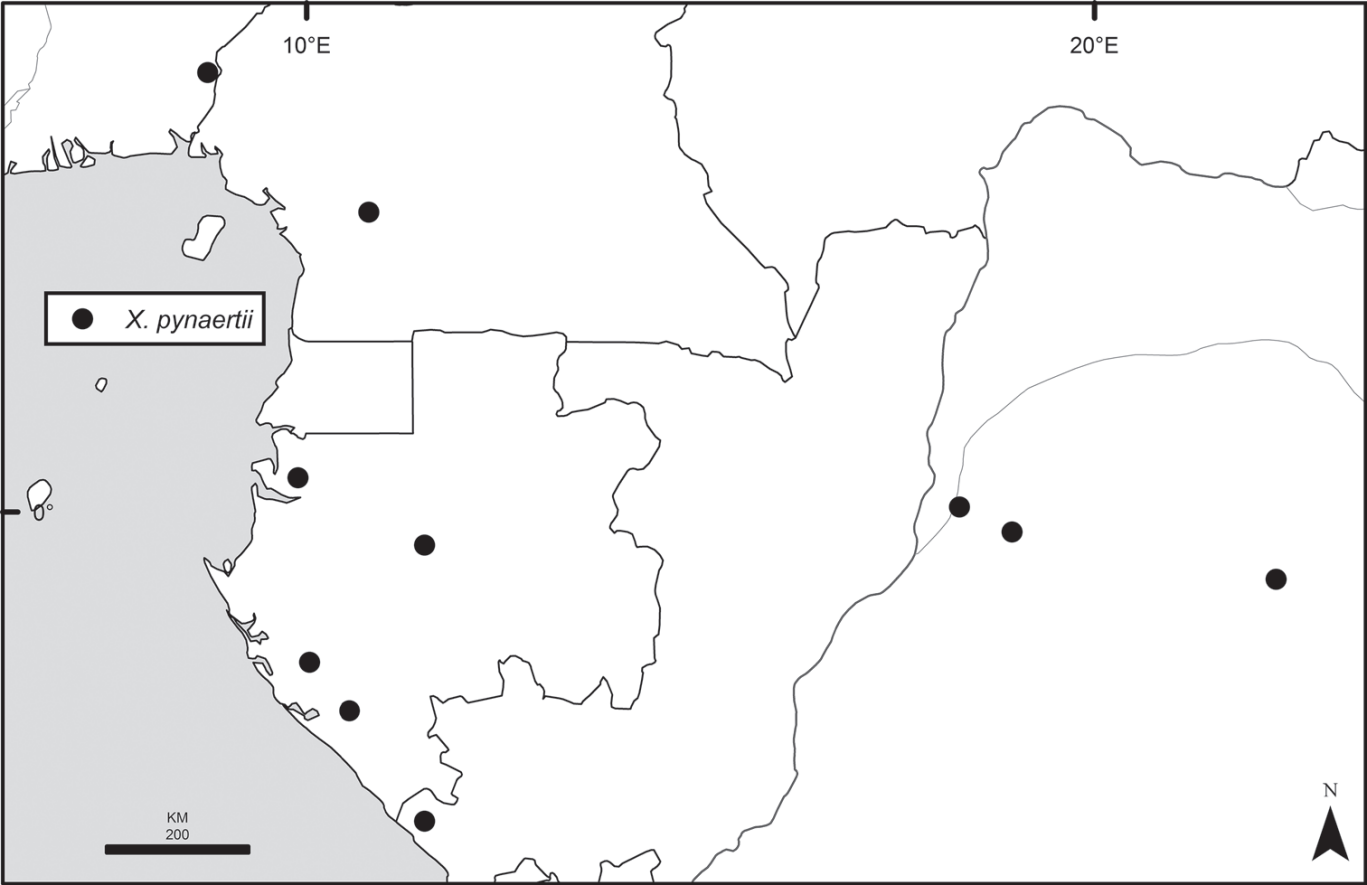
Distribution of *Xylopiapynaertii*. Bolder lines represent country borders, fainter lines lakes and major rivers.

(Fig. 47). Occurs from southeastern Nigeria to central Democratic Republic of the Congo and south to southern Republic of the Congo, where it grows in primary rainforest and semi-deciduous forest at elevations of 20–200 m.

#### Local names.

Bolonge (Boki, *Catterall 51*), n’tana (Fang, *Le Thomas 23*).

#### Additional specimens examined.

**NIGERIA.** Ikom, Cross River, Okwangwo, 16 Nov 1934 (fl), *Catterall 51* (K); Ikom, Cross River, 500’, 21 Dec 1934 (fr), *Catterall 61* (K). **CAMEROON**. Pout-Kelle 20 km N of Eseka, 9 Dec 1973 (fr), *Letouzey 12317* (K, P). **GABON**. Estuaire: N’Loulounga, 50 km E of Libreville, 5 Jul 1966 (fr), *Le Thomas 23* (P).—Nyanga: Chantier CEB, ca. 50 km SW of Doussala, 2°36'S, 10°35'E, 14 Jun 1985 (st), *Reitsma & Reitsma 1156* (NY, WAG); Chantier CEB, ca. 50 km SW of Doussala, 2°36'S, 10°35'E, 16 Oct 1985 (fl), *Reitsma & Reitsma 1621* (MO, NY, RSA, WAG).—Ogooué-Ivindo: réserve de la Lopé, au sud d’Ayem, chantier SOFORGA, 0°25'S, 11°30'E, 29 Mar 1989 (fl), *McPherson 13825* (BR, MO, PRE).—Ogooué-Maritime: Rabi area, NE Divangui, 40 m, 1°54.1'S, 10°02.3'E, 2 Oct 1994 (fr), *Wieringa & Nzabi 2831* (WAG). **REPUBLIC OF THE CONGO.** Environs du carrefour de N’Dindi, sur la route N’Tiétié, 12 May 1974 (fr), *Sita 3766* (P). **DEMOCRATIC REPUBLIC OF THE CONGO.** Équateur: Route Ingende-Bokatola, km 13, 9 Apr 1959 (fr), *Evrard 6059* (BR).—Tshuapa: Prov Équateur, Territ Bokungu, Bokota, 2 Apr 1959 (fr), *Evrard 5666* (K).

*Xylopiapynaertii* is a poorly known species, but readily distinguished from other central African *Xylopia* species by the scaly bright reddish brown upper bark, dense indument of the young twigs and abaxial leaf surfaces, small leaves, and short wide monocarps with seeds in two rows. Where noted, the red bark is a particularly useful character: Keay (1954–1958) listed the two Catterall collections as an “Imperfectly known species,” but quoted label descriptions “with red upper bark” that led to their correct identification. There is variation in the nature of the indument: collections with flowers have a mixture of long (>1.0 mm) and short hairs (ca. 0.5 mm long), while those with fruits had hairs that often appeared abraded and were never over 0.8 mm in length. The flowers of *McPherson 13825* were described as having a ginger fragrance.

While *Xylopiapynaertii* has a relatively wide distribution, the species is either rare or undercollected. We calculated an EOO of 582,432 km^2^, but an AOO of only 40 km^2^. In the Rabi 25-ha plot in southern Gabon, however, a total of 544 individuals were tabulated (Memiaghe et al. 2016), indicating that the plant may be locally common.

The protologue for *Xylopiapynaertii* mentions two collections, *Pynaert 567*, a collection with flowers made on 15 Oct 1906, and *Pynaert 1353*, a collection with fruits made in May 1907. The former was cited as the type by Le Thomas (1969) without elaboration. We have not seen the latter specimen, but, because the description pertains mostly to the flowering collection, *Pynaert 567* is formally designated here as the lectotype.

### 41. Xylopia talbotii

Taxon classificationPlantaeMagnolialesAnnonaceae

Exell, J. Bot. 69: 98–99. 1931.

6D2F9BF3-2A59-5645-A9DF-C11300E43832

Fig. 39F

#### Type.

NIGERIA. Cross River State, Oban, S. Nigeria, 1912, *P. A. Talbot 1601* (holotype: BM! (photos GH, NY); isotype: K! [000199068]).

#### Description.

***Tree*** up to 5 m tall; branches slender and somewhat flexuous. ***Twigs*** light brown, prominently ferruginous-pilose, with sparser hairs 1.5–2 mm long mixed with denser hairs 0.2–0.4 mm long, at length gray-brown, glabrate; no nodes seen with two axillary branches. ***Leaf*** with larger blades 9.2–12.5 cm long, 3.0–4.2 cm wide, chartaceous, concolorous to discolorous, lanceolate-oblong to elliptic, occasionally elliptic-oblanceolate or oblong-oblanceolate, apex acuminate, the acumen 9–17 mm long, base cuneate to rounded, glabrous except for the ferruginous-pubescent midrib adaxially, thinly sericeous to coarsely villous, especially along midrib, abaxially; midrib plane adaxially, raised abaxially, secondary veins weakly brochidodromous, 11–15 per side, diverging at 50–75° from the midrib, these and higher-order veins indistinct to slightly raised adaxially, slightly raised abaxially; petiole 2.5–5 mm long, shallowly canaliculate, ferruginous-pilose. ***Inflorescences*** axillary, 1-flowered, ferruginous-pilose; peduncles absent; pedicels 3.0–5.5 mm long, 1.3–1.5 mm thick; bracts 2–3, evenly spaced along pedicel, persistent, 2.8–4 mm long, ovate to broadly circular, apex acute to obtuse; buds lanceolate, apex acute and sometimes falciform. ***Sepals*** erect to slightly spreading at anthesis, 1/4-connate, 4–6 mm long, 3.1–3.8 mm wide, coriaceous, triangular to ovate, apex acute to acuminate, densely ferruginous-pubescent abaxially. ***Petal*** color *in vivo* unknown; outer petals slightly spreading at anthesis, 32–38 mm long, 3.1–3.3 mm wide at base, 2.0–3.0 mm wide at midpoint, coriaceous, linear, apex acute to obtuse, sometimes with a faint line down the center of the petal adaxially, with a weak longitudinal ridge abaxially, glabrous from base to one-third of length and then densely puberulent adaxially, sericeous abaxially; inner petals more or less erect at anthesis, 23–25 mm long, 2.4–3.3 mm wide at base, 1–1.5 mm wide at midpoint, coriaceous, linear, apex acute, base with undifferentiated margin, longitudinally ridged on both surfaces, densely gray-puberulent on both surfaces except for the glabrous base. ***Stamens*** ca. 120; fertile stamens 0.9–1.6 mm long, narrowly oblong, apex of connective 0.1–0.2 mm long, shieldlike, overhanging anther thecae, minutely papillate, anthers ca. 10–12-locellate, filament 0.3–0.4 mm long; outer staminodes 1.2–1.8 mm long, clavate to oblong, apex obtuse, rounded, or truncate; inner staminodes 1.1–1.2 mm long, clavate to oblong, apex truncate; staminal cone 1.3 mm in diameter, 1–1.1 mm high, concealing lower half of ovaries, rim laciniate. ***Carpels*** at least 8; ovaries ca. 1.3 mm long, oblong, pubescent, stigmas connivent, ca. 2.5–3 mm long, linear, a little bent at midpoint, glabrous except for apical tuft of hairs. ***Torus*** flat, 1.5–2.0 mm in diameter. Only immature fruit (< 6 mm long) seen, so pedicel and torus not expanded; ***Monocarps*** 8, clavate and somewhat falcate, apex with small curved beak, stipitate, sparsely pubescent. ***Seeds*** unknown.

#### Phenology.

Specimens with flowers have been collected in late November or December, and between late February and early April; immature fruits were collected in May.

#### Distribution

(Fig. 46). Occurs from southeastern Nigeria to Equatorial Guinea in gallery or lowland forest at ca. 50 m.

#### Additional specimens examined.

**NIGERIA.** Eket District, S. Nigeria, 1912–1913, *Talbot & Talbot 3209* (BM, MO). **CAMEROON.** About 10 km from Kribi, S of Lolodorf road, 2°58'N, 9°58'E, gallery forest on N bank of Kienke River, 27 May 1969 (yg fr), *Bos 4650* (BR, MO, P, WAG); Southwest Province, Korup National Park, 5°03'N, 8°48'E, 50 m, 28 Feb–3 April 1984 (fl), *Thomas 3204* (K, MO). **EQUATORIAL GUINEA.** Bebai, Camposgebiet, Weg u. [along Cameroon border, Tessmann 1913; locality is in NE corner of modern Equatorial Guinea], 23 Nov or Dec 1908 (fl), *Tessmann 747* (K).

*Xylopiatalbotii* is a species of the *X.acutiflora* subgroup, readily distinguished by the striking and persistent ferruginous pubescence of the twigs, petioles, pedicels, and calyx, and the long-acuminate leaves. Exell compared the species to *X.villosa*, which has a similar pubescence and overlaps somewhat in distribution, but *X.talbotii* differs in the sparser, coarser, and more erect indument of the abaxial leaf surface, versus the dense fine golden-sericeous indument of *X.villosa*, as well as inflorescences consisting of a single flower, rather than up to 8 flowers. The specimen *Tessmann 747* from Equatorial Guinea differs from the other specimens in the less pronounced leaf acumen and twig indument but is otherwise similar.

We calculated an EOO of 27,199 km^2^ and an AOO of 20 km^2^ for *Xylopiatalbotii*. It is one of several central African species that are represented in herbarium collections by a small number of specimens collected over a relatively wide area.

### 42. Xylopia tanganyikensis

Taxon classificationPlantaeMagnolialesAnnonaceae

D. M. Johnson, Kew Bull. 72:11: 9–11. 2017.

C0369849-FE88-5CAA-B475-2900B93FB600

Figs 4H , 28F–O

#### Type.

TANZANIA. Kigoma Region, T4, Kigoma District, Mahali Mts., 800–1500 m, s. d., *T. Nishida 57* (holotype: K!; isotype: EA!).

#### Description.

***Tree*** up to 25 m tall, d.b.h. up to 20 cm; bark light gray, finely fissured or scaly. ***Twigs*** reddish brown to blackish brown, sparsely and finely pubescent, the hairs 0.2–0.6 mm long, eventually gray to gray-brown, sparsely pubescent to glabrate; nodes occasionally with two axillary branches. ***Leaf*** with larger blades 6.4–9.0 cm long, 1.8–2.8 cm wide, chartaceous, usually discolorous, gray adaxially, yellow-olive to tan and paler abaxially, lanceolate to elliptic, apex acute, acuminate, obtuse, or retuse and more or less mucronate, the acumen, if present, 6–11 mm long, base cuneate to broadly cuneate, glabrous except for the pubescent midrib adaxially, finely appressed-pubescent abaxially; midrib plane to slightly impressed adaxially, raised abaxially, secondary veins brochidodromous, 10–17 per side, diverging at 60–70° from the midrib, these and higher-order veins indistinct to slightly raised on both surfaces; petiole 2–4 mm long, shallowly canaliculate, pubescent. ***Inflorescences*** axillary, 1(–2)-flowered, sparsely pubescent; peduncle 1 per axil, 1.3–2.2 mm long; pedicels 1 per peduncle, articulated with peduncle, 1–1.5 mm long; 1.5–1.6 mm thick; bracts 2–3, evenly spaced along pedicel, caducous, 1.7–3.5 mm long, broadly ovate to semicircular, apex acute to rounded; buds linear, falciform, apex acute. ***Sepals*** slightly spreading to spreading at anthesis, 1/4-connate, 2–3 mm long, 2.8–3.2 mm wide, coriaceous or sometimes slightly fleshy at base, ovate to broadly ovate, apex acute or apiculate, densely sericeous abaxially. ***Petals*** of unknown color *in vivo*; outer petals slightly spreading at anthesis, 28–38 mm long, 3.8–3.9 mm wide at base, 1.4–1.8 mm wide at midpoint, coriaceous, linear, apex acute, puberulent on distal half and downward along the margins but otherwise glabrous adaxially, appressed-pubescent abaxially; inner petals curved outward from the base but with the tips incurved at anthesis, 27–31 mm long, 2.7–3.0 mm wide at base, 0.9–1.0 mm wide at midpoint, coriaceous, linear, apex acute, base with undifferentiated margin, grayish silver-pubescent on both surfaces. ***Stamens*** 140–170; fertile stamens 1.3–2.7 mm long, narrowly oblong, apex of connective 0.1–0.2 mm long, shieldlike, overhanging the anther thecae, finely papillate, anthers 9–11-locellate, filament 0.4–0.6 mm long; outer staminodes ca. 1.7 mm long, oblong to broadly clavate, apex rounded; inner staminodes 1.0–1.2 mm long, oblong, apex obtuse to truncate; staminal cone ca. 2.1 mm in diameter, 0.8–1 mm high, concealing all but apices of the ovaries, rim laciniate. ***Carpels*** ca. 11; ovaries ca. 1.2 mm long, oblong, pubescent, stigmas more or less discrete, 2.5–3.3 mm long, linear, glabrous except for an apical tuft of hairs. ***Torus*** flat, 2.0–2.7 mm in diameter. ***Fruit*** of up to 9 glabrate monocarps borne on a pedicel 5–16 mm long, 1.3–6 mm thick, sparsely pubescent to glabrate; torus 9–14 mm in diameter, 8–9 mm high, globose to depressed-globose. ***Monocarps*** with a green exterior and red endocarp *in vivo*, 3.0–5.4 cm long, 1.6–2.0 cm wide, 0.8–1.6 cm thick, obovoid to oblong-obovoid, not or only weakly torulose, apex rounded, base sessile or contracted into a stipe 2–10 mm long, 3.5–6 mm thick, finely verrucose; pericarp 0.5–0.7 mm thick. ***Seeds*** 3–7 per monocarp, in a single or irregular double row, lying oblique to perpendicular to long axis, 9.4–14.0 mm long, 6.9–9.2 mm wide, 6.2–8.4 mm thick, ellipsoid to oblong, irregularly elliptic to wedge-shaped in cross section, truncate at micropylar end, rounded at chalazal end, brown, smooth, slightly shiny, raphe/antiraphe not evident, micropylar scar 5–5.5 mm long, 3–4 mm wide, ovoid; sarcotesta pale gray, fleshy *in vivo*; aril absent.

#### Phenology.

The single flowering collection lacks a date. Flowering phenology information in Johnson et al. (2017) indicating flowering in March and April is an error. Specimens with fruits have been collected in February, April, and July.

#### Distribution

(Fig. 38). Known only from western Tanzania along or near Lake Tanganyika, growing in evergreen forest at 800–1600 m.

#### Local names.

Kafwibili (Kitongwe, *Nishida 57*), kahwibili (*Itoh & Sakamaki NI97-62*, *Uehara 580*), tunda-yai (Kibembe, *Abeid et al. 1028*).

#### Additional specimens examined.

**TANZANIA.** Katavi: Mpanda District, SW of Mwese village, Lubalisi Village, Mtakala Forest, Kuleba Hill Peak, 06°13 00 S, 030°18 00 E, 1600 m, 20 Jul 2001 (fr), *Abeid et al. 1028* (L, MO).—Kigoma: Mahale Mts., 860–880 m, 23 Feb 1998 (fr), *Itoh & Sakamaki NI97-62* (K); Mahale National Park, *Ken’ichi Masni [Masui?] 2-20* (EA); T4, Kigoma District, Mahali Mts., 800–1500 m, s. d. (fr), *Nishida 51* (EA, K). T4, Lake Tanganyika, Mahale Mts., Kasiha, 850 m, 25 Apr 1978 (fr), *Uehara 580* (EA, K).

*Xylopiatanganyikensis* has narrow discolorous leaves with appressed abaxial pubescence, short-pedicellate flowers with petals up to 38 mm long, and sessile monocarps widest at or beyond the middle and rounded at the apex. It resembles *Xylopiaelliotii* of the Sahel and Sudanian regions in its discolorous leaves and oblong to obovoid monocarps with rounded apices, but the latter species is a smaller tree of gallery forest with longer flower pedicels and smaller monocarps. *Xylopiatanganyikensis* also resembles *X.cupularis*, but lacks the golden-sericeous abaxial leaf surface, longer pedicels, and long-stipitate monocarps of that species. *Xylopiashirensis*, with which it possibly overlaps in distribution, is a smaller tree of miombo (*Brachystegia* spp.) woodland with broader and more pubescent leaves.

This is the easternmost species of the *Xylopiaacutiflora* group. It is probably allied to the other members of the group with large ovoid or oblong monocarps, such as *X.hypolampra* and *X.phloiodora*, which share a tendency for the monocarp to split into three segments upon dehiscence.

*Xylopiatanganyikensis* has a proposed IUCN Conservation Assessment of Endangered B1ab(iii) + B2ab(iii), recognizing threats from habitat alteration despite the fact that it occurs within the protected area of Mahale Mountains National Park (Johnson et al. 2017). The specimen *Léonard 4713* (BR), from the eastern Democratic Republic of the Congo, may also represent *X.tanganyikensis*.

### 43. Xylopia thomsonii

Taxon classificationPlantaeMagnolialesAnnonaceae

Oliver, Fl. trop. Afr. 1: 31. 1868.

3F76E728-EFF4-54BD-8F19-34FDD7697CEC

Fig. 39A–C

Xylopicrum thomsonii (Oliver) Kuntze, Revis. gen. pl. 1: 8. 1891. Type. NIGERIA [“Upper Guinea”]. Rivers State, Old Calabar, s. d., *W. C. Thomson 63* (holotype: K! [000199064]).
Xylopia pyrifolia Engler, Pflanzenw. Ost-Afrikas C, 179. 1895. Type. DEMOCRATIC REPUBLIC OF THE CONGO. Ituri Province, Bataibo am Duki, 850 m, 7 Nov [1891], *F. L. Stuhlmann 2781* (holotype: B! [100153155]).
Xylopia tenuifolia Engler & Diels, Notizbl. Königl. Bot. Gart. Berlin 2: 298. 1899.
Xylopicrum tenuifolium (Engler & Diels) Kuntze, Deutsch. Bot. Monatsschr. 21: 173–174. 1903. Type. CAMEROON. Southwest Region, Urwald zwischen Mowange und Isongo [? = Port Isongo, 4°04'N, 9°01'E], Mar 1897, *P. R. Preuss s. n.* (type B, apparently destroyed).
Xylopia seretii [“*sereti*”] De Wildeman, Ann. Mus. Congo, Sér. 5, Bot. 3: 79–80. 1909. Type. DEMOCRATIC REPUBLIC OF THE CONGO [“Belgian Congo”]. Haut-Uele Province, bords d’une riviere sur la route de Faradje à Vankerkhovenville, 12 Apr 1906, *F. Seret 555* (holotype: BR!; isotype: BR! [0000008824370]).

#### Description.

***Shrub or small tree*** up to 10 m tall, often with lianescent branches that may extend up to 10 m, d.b.h. up to 17.3 cm. ***Twigs*** light to dark brown, erect-pubescent, the hairs 0.1–1.3 mm long, but often with a mix of denser short (0.1–0.5 mm) hairs and sparser long (0.8–1.3 mm) hairs, eventually brownish gray, glabrate, with the bark exfoliating; nodes rarely (seen in one specimen) with two axillary branches. ***Leaf*** with larger blades 8.4–13.7 cm long, 2.5–4.7 cm wide, chartaceous, concolorous or more often discolorous, lanceolate, elliptic to oblong, or oblong-oblanceolate, apex acuminate, the acumen 3–16 mm long, base cuneate to rounded, rarely almost truncate, glabrous except for the pubescent midrib adaxially, sparsely appressed-pubescent to glabrate abaxially; midrib slightly impressed to plane adaxially, raised abaxially, secondary veins irregularly brochidodromous, 11–17 per side, diverging at 45–90° from the midrib, these and higher-order veins slightly raised but indistinct adaxially, raised abaxially; petiole 1.5–5.5 mm long, shallowly canaliculate, sparsely pubescent. ***Inflorescences*** axillary or from axils of fallen leaves, 1(–2)-flowered, rusty-sericeous; pedicels not pedunculate, 2.5–7.9 mm long, 0.8–1.3 mm thick; bracts 3–6, usually closer together and more overlapping toward pedicel base, persistent, 1.6–3.5 mm long, semicircular, elliptic, or ovate, apex obtuse, acute, slightly acuminate, or sometimes bilobed; buds linear, apex acute, sometimes slightly falciform. ***Sepals*** erect or slightly spreading at anthesis, 1/6–2/5-connate, 1.8–3.8 mm long, 2.6-3.0 mm wide, chartaceous to coriaceous, ovate, elliptic, or triangular, apex acute, acuminate, or sometimes obtuse, rusty-sericeous abaxially. ***Petals*** white, cream, or pale mauve *in vivo*; outer petals somewhat spreading at anthesis, (14.6–) 22–49 mm long, 2.4–3.8 mm wide at base, 1.0–2.0 mm wide at midpoint, linear, apex acute to obtuse, puberulent, becoming glabrous toward the base adaxially, rusty-sericeous, becoming glabrous at the base abaxially; inner petals curved outward to weakly geniculate but curved inward toward the apices at anthesis, 16–33 mm long, 2.1–3.2 mm wide at base, 0.6–1.2 mm wide at midpoint, coriaceous, linear, apex acute, base with undifferentiated margin, transversely thickened at the widest point, longitudinally ridged on both surfaces, puberulent on both surfaces but becoming glabrous toward the base. ***Stamens*** ca. 200; fertile stamens 1.2–1.8 mm long, narrowly oblong; apex of connective 0.2–0.4 mm long, dome-shaped, minutely papillate, anthers 9–13-locellate, filament 0.3–0.5 mm long; outer staminodes 1.3–1.8 mm long, clavate to oblong, apex obtuse to obliquely truncate; inner staminodes 0.7–1.2 mm long, clavate to oblong, apex rounded to truncate; staminal cone 1.3–2.0 mm in diameter, 0.5–1.1 mm high, concealing the lower portion of the ovaries, rim laciniate. ***Carpels*** 7–13; ovaries 0.9–1.3 mm long, oblong, densely pubescent, stigmas connivent, 1.8–3.4 mm long, linear-falcate, apices pubescent. ***Torus*** 1.7–2.6 mm in diameter. ***Fruit*** of up to 12 glabrate monocarps borne on a pedicel 5–12 mm long, 2.2–7.5 mm thick, basal bract or sepals sometimes persistent, sparsely appressed-pubescent; torus of fruit 2–9 mm in diameter, 3.5–5 mm high, globose. ***Monocarps*** with a green exterior and red to red-purple endocarp *in vivo*, 2.1–6.5 cm long, 0.6–1.2 cm wide, 0.5–0.6 cm thick, narrowly oblong and often somewhat falciform, weakly torulose, apex obtuse or occasionally with a broad beak 1–4 mm long, base contracted into a stipe 3–11 mm long, 1.7-2.7 mm thick, smooth to longitudinally or obliquely wrinkled, verrucose; pericarp 0.2–0.3 mm thick. ***Seeds*** up to 9, commonly 4–8 per monocarp, in a single row, lying oblique or occasionally nearly perpendicular to long axis, 9.2–12.3 mm long, 5.5–6.7 mm wide, 4.8–6.3 mm thick, ellipsoid to oblong, elliptic to nearly circular in cross section, truncate at micropylar end, rounded at chalazal end, brown, smooth or with shallow pits or wrinkles, dull to slightly shiny, raphe/antiraphe sometimes visible but flush with seed surface, micropylar scar 2.5–4.5 long, 1.5–3 mm wide, broadly elliptic to nearly circular; sarcotesta thin, waxy, green *in vivo*, sometimes visible as a white crust on dried seeds; aril absent.

#### Phenology.

Specimens with flowers have been collected in all months of the year except August, most frequently from November to May. Specimens with fruits have been collected in all months except March and August.

#### Distribution

(Fig. 34). Occurs from southeastern Nigeria east to South Sudan and south to northern Angola and south-central Democratic Republic of the Congo, in understory of mature forest or gallery forest, occasionally in marshy forest or forest edges near water, at elevations from sea level to ca. 1000 m.

#### Local names.

Akwi (Ewondo, *Breteler 931*), anzungunzungu (Zande, *Gerard 1611*), apanjinji (Kibila, *Hart 284*), azungu-zungu (*de Graer 241*), likungu (Turumbu, *Louis 1842*), sange (*Hart 68*), sangi tingoo (Central African Republic, *Harris & Fay 607*).

#### Representative specimens.

**NIGERIA.** Loe, Ibadan District, *Ainslie 169* (PR); Calabar Province, Agoi Forest Reserve, 26 Jan 1962 (fr), *Binuyo FHI 45456* (K—2 sheets); Lagos, (fl), *Dalziel 1144* (M); without definite locality [probably Sapoba], *Kennedy 169* (A); “Southern Nigeria” [probably Sapoba], *Kennedy 2270* (A—2 sheets, BM, F, MO, US); Oban, 1912 (fl), *Talbot 1353* (BM); Oban, 1912 (fl), *Talbot 1486* (BM); Eket District, 1912–13 (fl), *Talbot & Talbot 3267* (BM); Iba, Jan 1933 (fl bud), *Thomewill 200* (K). **CAMEROON.** Bitya, near the River Dja [“Ja”], s. d. (fl), *Bates 1852* (K); S bank of Lobé R. SE of Gr. Batanga ferry, 2°52'N, 9°54'E, 11 Oct 1969 (fl), *Bos 5475* (B, K, MO, P, WAG—3 sheets); forest along river Sanaga near Goyoum 20 km W of Deng Deng, ca. 635 m, 27 Jan 1961 (fl, fr), *Breteler 931 [Letouzey 3261*] (BR, K, P, WAG—2 sheets); Bétaré Oya, 5 km along road to Bertoua, ca. 900 m, 17 Feb 1961 (fl), *Breteler 1063* (A, FI-T, K, M, P, WAG—2 sheets); Bétaré Oya, 5 km along road to Bertoua, ca. 900 m, 26 Feb 1961 (fr), *Breteler 1137* (WAG—2 sheets); bank of the Doumé River, 40 km SW of Batouri, near village Bimba, 15 Apr 1962 (fl, fr), *Breteler 2797* (A, BR, FI-T, K, M, P), Southwest, Korup National Park, P transect, P plot, subplot 20F, 5°01'N, 8°48'E, 100 m, 10 Mar 2004 (fl), *van der Burgt 662* (K); Prov. Southwest, along Lobe River near Ekondo Titi, 4°36'N, 9°00'E, 28 Feb 1987 (fl), *Doumenge 313* (MO); 8 km W of Masok, 400 m, 27 Mar 1965 (fl), *Leeuwenberg 5217* (B, DSM, K, L, MO, P, W, WAG—2 sheets); 10 km à l’Ouest de Bagodo, 28 Jul 1966 (fr), *Letouzey 7563* (K, P); Mayo Banyo, 20 km NNE of Banyo, 8 Jun 1967 (fl), *Letouzey 8552 (leg Mpom Benoit* (K); Colline de Mill (5 km NE Lolodorf), 26 Jan 1974 (fl, fr), *Letouzey 12797* (P); Bamenda, Fonfuka [6°31'N, 10°26'E], 3000-3500’, May 1931 (fl), *Maitland 1731* (K); Southwest, Three-corners Foe, 04°33'N, 09°04'E, 100 m, 28 Jul 1986 (fr), *Mambo & Thomas 97* (G, MO); Ebolowa-Jaünde, Jan 1914 (fl), *Mildbraed 7716* (K); etwa 180 km NW Jaunde, May 1914 (fl), *Mildbraed 8422* (K); Mbüssa, 8–900 m, 6°N, 14°20'E, 29 Apr 1914 (fl), *Mildbraed 9069* (K—2 sheets); South West Province, Mount Cameroon, Njonji, 4°07'N, 9°01'E, 420 m, 18 Apr 1997 (fl), *Nning 364* (MO); Ngoasik (10 km SSE Ambam), rive du Ntem, 1 Mar 1963 (fl), *Raynal & Raynal 10130* (P, W); Bertoua—Batouri, 1962 (fl), *Tchinaye 113* (P); Korup Reserve, [ca. 4°59'N, 8°51'E], 12 Jan 1979 (fl), *Thomas 595* (K); Southwest Province, Korup National Park, 5°03'N, 8°48'E, 50 m, 28 Feb-3 Apr 1984 (buds), *Thomas 3242* (MO); Lombe Camp, Tissongo Study Area, Douala-Edea Reserve, Jun 1976 (fr), *Waterman & McKey 809* (K); ca. 10 km SW of Ambam, S of Ebolowa, on N bank of Ntem River, 2 Mar 1964 (fl), *de Wilde & de Wilde-Duyfjes 2043* (B, K, MO); ca. 50 km S of Badjob, ca. 60 km NW of Eséka, along the Njong River, 20 Mar 1964 (fl), *de Wilde & de Wilde-Duyfjes 2172* (B, K, MO, PRE, WAG); bank of Nyong River, near the new bridge, ± 65 km SSW of Eséka, ± 200 m, 16 Jun 1964 (fr), *de Wilde & de Wilde-Duyfjes 2703* (B, K, MO, WAG—2 sheets); about 10 km S of Meiganga, ca. 900 m, 23 Nov 1964 (fr), *de Wilde et al. 3994* (WAG—2 sheets); region M’Bamileke [“M'Bamileleke”], 19 Dec 1957 (fl), *de Wit 352* [de Wit Herbarium 7952] (WAG—2 sheets). **CENTRAL AFRICAN REPUBLIC.** Oubangui, Rég. E Yango, Feb 1920 (fl), *Adjt. Allouette s. n.* [herb. De Ch. d’Alleizette] (L); Sangha Economique Prefecture, Ndakan, gorilla study area Njéké from M 5400 to C 5800, 16°12'N, 02°21'E, 350 m, 7 May 1988 (fl), *Harris & Fay 607* (K, MO); Yalinga, 30 Mar 1921 (fl), *Le Testu 2605* (BM, P, US); entre Yalinga et Bria (km 60), Guyao, 29 Apr 1921 (fl), *Le Testu 2681* (BM, BR, MO—2 sheets); Yalinga, Haut Ubangi, 16 Mar 1923 (fl), *Le Testu 4617* (BM, MO, P); Yalinga, 30 Apr 1923 (fl), *Le Testu 4702* (BM); Yalinga, 25 Feb 1921 (fr), *Le Testu s. n.* (BM); bank of the Sangha River (international frontier) collected 30 km from Libongo southwards, 02°30'N, 16°04’ E, 350 m, 27 Jul 1987 (fl), *Thomas & Fay 7281* (MO, WAG); dans les roches Riv. Yakumburu 35 km E Moroubas, 1 May 1922 [1921 on P sheet, mai 1923 in Tisserant and Sillans 1953] (fl), *Tisserant 476* (BM, P); Riv. Dakpete 30 km E Moroubas, 1 Apr 1924 (fl), *Tisserant 1470* (BM); Bozoum, 1 May 1932 (buds), *Tisserant 3068* (BM). **SOUTH SUDAN.** SW Equatorial Province, Aloma Plateau, ca. 1 mile SSW of Iwatoka, 23 Mar 1939 (fl), *Hoyle 823* (BM, K); Korobe Forest, Yei District, Equatoria, 2 Jul 1958 (st), *Jackson 3894* (K); am Nabambim, 19 Feb 1870 (bud, fr), *Schweinfurth 3032* (K); Lado, Yei River, Asugi, 23 Oct 1919, *Sillitoe 353* (K); Lado, Yei River, 10 Nov 1919 (fl), *Sillitoe 479* (K). **GABON.** Estuaire: 8 km N of Libreville, 30 Jan 1961 (fl), *Hallé 961* (WAG); forêt de la Mondah, 0°33'N, 9°23'E, 19 Dec 1993 (fl), *Jongkind & Breteler 1329* (MO—2 sheets); ca. 1 km sur petite piste à droite, 2 km avant Cap Estérias, 12 Nov 1982 (fl), *Louis 97* (WAG—2 sheets); N of Libreville, ca. 10 km on road to Cap Esterias, 0°30'N, 9°22'E, 10 m, 20 Nov 1991 (fr), *McPherson 15526* (MO); ca. 20 km N of Libreville, 0°35'N, 9°22'E, 29 Jan 1987 (fl), *Reitsma & Reitsma 2862* (MO, NY, RSA); Cap Esterias, Forêt de la Mondah, marsh forest, 10 m, 0°36.7'N, 9°23.3'E, 23 Oct 1994 (fr), *Wieringa et al. 2884* (WAG); about 14 km along the road Libreville to Cape Esterias, 26 Nov 1983 (bud), *de Wilde et al. 785* (MO).—Ngounié: Waka, forest exploitation road near what is called the Falaise, ca. 400 m, 1°18'S, 10°57'E, 22 Nov 1964 (fl), *Arends et al. 389* (MO, P).—Nyanga: Doudou Mountains, 8 Dec 1984 (fl), *Arends et al. 708* (MO, P, WAG).—Ogooué-Ivindo: Ile de l’éléphant, Makokou, 21 Sep 1971 (fl, fr), *Hladik 1540 part A* and *part B* (P); Ile de l’éléphant, pointe nord Makokou, 20 Jan 1972 (fl), *Hladik 1924 part A* (P); Ipassa, 10 km S of Makokou, small island in Ivindo River, 500 m, 20 Jan 1972 (fl), *Hladik 1924 part C* (US).—Ogooué-Maritime: SE of Port Gentil, ca. 0°40'S, 8°50'E, 16 Sep 1968 (fr), *Breteler & van Raalte 5564* (WAG); Rabi-Kounga, ca. 1°55'S, 9°55'E, 25 Dec 1991 (fl), *Breteler & Jongkind 10111* (WAG); 1 km on the road from Rabi to Divangui, along track, 1°54'S, 9°53'E, 25 Nov 1989 (fl), *de Wilde et al. 9740* (WAG); Gamba, 19.5 km Gamba airport road to Mayonami, then 5 km along track to Nyanga River, ca. 2°56'S, 10°12'E, 23 Nov 1994 (fl, fr), *de Wilde & de Wilde-Bakhuizen 11194* (WAG).—Woleu-Ntem: région entre Ogooué et Cameroun, bords de la Kyé, à Méyo’, 12 Mar 1933 (fl), *Le Testu 9025* (BM, P); Bélinga, 19 Dec 1964 (fr), *Hallé 3545* (P). **REPUBLIC OF THE CONGO.** Environs de N’tiétié, à 13 km du village vers N’Gongo, 9 Dec 1974 (fr), *Sita 3813* (P). **DEMOCRATIC REPUBLIC OF THE CONGO.** Bas-Uele: Tukpwo, galerie de la Diagbo, 22 Nov 1954 (fr), *Gerard 1611* (BR).—Haute-Uele: without definite locality [probably vicinity of Doruma], (fr), *de Graer 241* (BR); Province Orientale, District Haut Uele, Territoire Dungu, Region Doruma, 4 Feb 1958 (fr), *Leclercq 346* (BR).—Ituri: Epulu, zone de Mambasa (Ituri), 1°25'N, 28°35'E, 750 m, 12 May 1981 (fl), *Hart 68* (K); Epulu, zone de Mambasa (Ituri), 1°25'N, 28°35'E, 750 m, 12 May 1982 (fl), *Hart 284* (K).—Mai-Ndombe: entre Kole et Dekese (Lac Leo II), Oct 1932 (fl), *Lebrun 6447* (K, P); Kutu (Lac Leo II), Nov 1932 (st—old pedicel), *Lebrun 6599* (MO).—Tshopo: Yangambi, 7 Mar 1958 (fl), *Leonard 247* (BR); Yangambi, plateau de la Lusambila, 8 May 1936 (fr), *Louis 1842* (BR); Yangambi, embouchure Isalowe, 25 Mar 1938 (fl), *Louis 8620* (RSA); Yangambi, au borde de la rivière Isolowe, Jul 1938 (fl), *Louis 10255* (B, MO, NY, US); Yangambi, au bord de l’Etehwa, Oct 1938 (fl), *Louis 13217* (BM, K—2 sheets, P); Yangambi, près de la rivière Lusambila, 12 Jan 1961 (fl), *Yafunga F.60* (BR).—Province unknown: am Mbowole [I. Darbyshire, personal communication, indicates that this locality is in present-day Democratic Republic of the Congo], Mar 1870 (fl), *Schweinfurth 3234* (K); **ANGOLA.** Lunda: Vila Henrique de Carvalho (Saurimo), pr. rio Chicapa, 1015 m [1050 m on MO sheet], Apr 1937 (yg fr), *Gossweiler 11650* (BM, K, MO); nordeste de Lunda, Dundo, proximum flumen Luachima, 3 Sep 1946 (fl), *Gossweiler 13648* (BM—2 sheets, K, US); nordeste de Lunda, circunscrição de Chitato, Marhura, river Chicapa, 700 m, 17 Jun 1948 (fl), *Gossweiler 14110* (BM, K).

*Xylopiathomsonii* is a lianescent shrub or occasionally a small tree occurring in lowland forests, usually near wetlands. Within the *Xylopiaacutiflora* subgroup, the species can be distinguished by the broadly cuneate to rounded leaf base, narrow petals variable in length but with the outer petals significantly longer than the inner petals, and slightly torulose blunt-tipped monocarps with up to 9 seeds in a single row. Indument of the twigs varies from a mixture of erect long and short hairs to short hairs only, the latter occurring primarily in specimens from coastal Gabon and Cameroon. The type specimen of *X.thomsonii* has some axils with two flowers, and the original description of the species indicates up to 20 carpels per flower. In our material, the inflorescences usually consisted of a single flower and the flowers never had more than 13 carpels. Even segregated from *X.acutiflora*, there is variability in this widespread species.

The range of *X.thomsonii* overlaps with that of *X.unguiculata* in the southern part of its distribution, and approaches that of *X.monticola* along the border between Cameroon and Nigeria. *Xylopiaunguiculata* can be distinguished by the cuneate rather than rounded leaf bases, the broader outer petals, and the claw-like beak of the monocarps. In addition, the pedicels of *X.unguiculata* are shorter and the monocarps more strongly torulose. *Xylopiamonticola* does not exhibit the liana habit and has monocarps with fewer seeds and proportionately longer stipes.

For a frequently collected species, little is known about the biology of *X.thomsonii*. The label of *Léonard 247* from Democratic Republic of the Congo described it as occurring in forest of *Scorodophloeus* along water but no additional details were given.

The type of *X.pyrifolia* Engler at B, figured in Engler and Diels (1901, Plate XXII, Fig. B), consists of branches with leaves and one fruit of immature monocarps borne on a short pedicel. The leaves, while proportionately broader than is typical for *X.thomsonii*, have the same texture and rounded base. The monocarps, while immature, have a short stipe, blunt apex, and a single row of 2–4 seeds, all typical for the species. More problematic is the name *Xylopiatenuifolia*. The type specimen appears to be lost, but Engler and Diels (1901) placed it as a synonym of *Xylopiaoxypetala*. The description agrees with that of *X.thomsonii* in the relatively thin oblong to elliptic-oblong leaves 10–13 cm long with few hairs and the usually solitary flowers, and the type locality in coastal Cameroon is plausible for *X.thomsonii*. Although the type specimen of *Xylopiaseretii* comes from the northeastern extreme of the distribution, it agrees well with other specimens of *X.thomsonii* from that area.

### 44. Xylopia unguiculata

Taxon classificationPlantaeMagnolialesAnnonaceae

D. M. Johnson & N. A. Murray sp. nov.

9AB87A39-1367-5A2A-8196-8C1B5146D454

urn:lsid:ipni.org:names:60476246-2

Fig. 33A–D, L–O

#### Diagnosis.

Species resembling *X.acutiflora* in the tree habit, mixture of long and short erect hairs on the twigs, short pedicels 2.8–4.8 mm long, and outer petals up to 2.5 mm wide at midpoint, but differing in the cuneate to broadly cuneate leaf base and the strongly torulose monocarps with seeds in a single row and a prominent beak at the apex.

#### Type.

GABON. Nyanga Province, ca. 50 km SW of Forestry Camp Doussala, 2°36'S, 10°35'E, 20 Feb 1986, *J. M. Reitsma & B. Reitsma 1923* (holotype: WAG! [0050003]; isotypes: MO! [3879083], NY! RSA!).

#### Description.

***Tree*** up to 17 m tall, rarely a shrub, d.b.h. up to 16 cm; bark smooth. ***Twigs*** brown, pilose-pubescent, with a mixture of dense hairs 0.1–0.2 mm long and sparser longer hairs 0.7–1.3 mm long, at length gray-brown to dark brown, glabrate, with the bark somewhat exfoliating; nodes rarely with two axillary branches. ***Leaf*** with larger blades 8.9–12 cm long, 3.0–4.5 cm wide, chartaceous, slightly discolorous, elliptic to somewhat oblanceolate, obovate, or oblong, apex acuminate, the acumen 4–13 mm long, base cuneate to broadly cuneate, glabrous except for the pubescent midrib adaxially, sparsely appressed-pubescent to glabrate abaxially; midrib slightly impressed adaxially, raised abaxially, secondary veins indistinctly brochidodromous, 8–14 per side, diverging at 45–75° from the midrib, these and higher-order veins plane to slightly raised adaxially, raised abaxially; petiole 2.5–6 mm long, canaliculate, sparsely pubescent. ***Inflorescences*** axillary or from the axils of fallen leaves, 1-flowered, densely pubescent; pedicels not pedunculate, 2.8–4.8 mm long, 1.4–1.5 mm thick; bracts 3–4, imbricate, persistent, 2.3–3.8 mm long, orbicular, apex rounded to emarginate; buds linear, apex obtuse. ***Sepals*** slightly spreading at anthesis, 1/4–1/3-connate, 2.8–3.9 mm long, 3.4–3.6 mm wide, coriaceous, broadly ovate, apex acute to obtuse, densely brown-tomentose abaxially. ***Petals*** white to pale yellow with a patch of purple on the adaxial base *in vivo*; outer petals slightly spreading at anthesis, 22–30 (–46) mm long, 3.4–4.5 mm wide at base, (1.4–) 2.2–2.5 mm wide at midpoint, coriaceous, linear, apex acute to obtuse, glabrous and somewhat verrucose on lower half but otherwise puberulent adaxially, sericeous abaxially; inner petals appearing bent outward at the base at anthesis, 15.5–25 (–29) mm long, 3.0–3.6 mm wide at base, 1.2–1.6 mm wide at midpoint, coriaceous, linear, apex acute, glabrous on proximal 1/3–1/2 but otherwise puberulent adaxially, puberulent except for the glabrous base abaxially. ***Stamens*** 90–160; fertile stamens 1.2–1.9 mm long, clavate to narrowly oblong, apex of connective purple *in vivo*, 0.2–0.4 mm long, dome-shaped to shieldlike, overhanging the anther thecae, minutely papillate, anthers 9–13-locellate, filament 0.2–0.7 mm long; outer staminodes 1.2–1.9 mm long, clavate or oblong, apex obtuse to truncate; inner staminodes 0.8–0.9 mm long, clavate, apex truncate; staminal cone 1.9–2.1 diameter, 0.6–1.1 mm high, concealing the lower half of the ovaries, rim laciniate. ***Carpels*** 10–15; ovaries ca. 1 mm long, oblong, densely pubescent, stigmas connivent except for the free apices, ca. 3 mm long, linear, glabrous except for tuft of hairs at apex. ***Torus*** flat, 1.8–2.5 mm in diameter. ***Fruit*** of up to 20 sparsely pubescent to glabrate monocarps borne on a pedicel 6.7–9 mm long, 3.3–6.5 mm thick, with sepals and bracts persistent, glabrate; torus of fruit ca. 8 mm in diameter, 5 mm high, depressed-globose. ***Monocarps*** with green exterior and red endocarp *in vivo*, 4.9–8.5 cm long, 0.8–0.9 cm wide, 0.7–0.8 cm thick, narrowly oblong, strongly torulose, almost moniliform, apex rostrate, the beak 2.5–6 mm long, strongly curved, base contracted into a flattened and grooved stipe 8–18 mm long, 1.9–2.1 mm thick, slightly wrinkled and verrucose; pericarp 0.1–0.3 mm thick. ***Seeds*** up to 8 per monocarp, in a single row, parallel or oblique to long axis, 11.9–12.4 mm long, 6.6–7.5 mm wide, 6.0–7.0 mm thick, oblong, more or less circular in cross-section, truncate at micropylar end, rounded at chalazal end, smooth or slightly bumpy, dull, raphe/antiraphe not evident, micropylar scar 3.3–4.6 mm long, 2.9-4.5 mm wide, broadly elliptic or roughly circular; sarcotesta unknown *in vivo*, sometimes forming a white crust on dried seeds; aril absent.

#### Phenology.

Specimens with flowers have been collected in February, March, May, July, and December, and with fruits from October to December.

#### Distribution

(Fig. 34). Gabon, with one collection from the northern part of the country near the border with Cameroon and the remainder from southern Gabon, in primary rainforest or exploited high forest, at elevations of 250–650 m.

#### Additional specimens examined.

**GABON.** Nyanga: Tchibanga, 5 Oct 1907 (fr), *Le Testu 1179* (BM, P); forêts du Mayombe, Tchibanga, Dabilila, 15 Nov 1908 (fr), *Le Testu 1446* (BM, P); Mayombe bayaka, Dabilila, 11 Dec 1908 (fl), *Le Testu 1514* (BM—2 sheets, MO, P); région Nyanga, Tchibanga, 8 Dec 1914 (fr), *Le Testu 1903* (BM, P); chantier CEB, ca. 50 km SW of Doussala, 2°36'S, 10°35'E, 26 Aug 1985 (buds), *Reitsma & Reitsma 1401* (MO, NY, RSA); about 30 km NW of Doussala, in the direction of Bongo, 400 m, 16 Mar 1988 (fl), *de Wilde & Jongkind 9393* (MO, WAG).—Ogooue-Maritime: 32 road-km N of Igotchi-Mouenda, Bakker timber concession, 250 m, 02°41'S, 10°30'E, 13 May 1977 (fl), *McPherson 16960* (MO), *McPherson 16960A* (MO).—Woleu-Ntem: région entre Ogooué et Cameroun, Mbabou, 31 Jul 1933 (fl), *Le Testu 9208* (BM, K, P).

*Xylopiaunguiculata* bears monocarps with distinctive claw-like apices; the specific epithet alludes to this apex shape. The illustration of the fruit identified as *X.acutiflora* in Le Thomas (1969) shows the appearance of the apex well. The new species shares with *X.acutiflora* from West Africa a tree habit and a short pedicel covered by imbricate bracts. *Xylopiaunguiculata* differs, however, in its cuneate to broadly cuneate, rather than broadly cuneate to rounded leaf base, broader outer petals, and a larger number of falciform, strongly torulose monocarps with the prominent claw-like beak and seeds in a single row. [Note: The illustrations in Le Thomas (1969) provided for *Xylopiaacutiflora* show a mixture of two species, the flowers and floral parts being based on *Bates 1852*, identified here as *X.thomsonii*, and the fruits on *Le Testu 1179*, identified as *X.unguiculata*.]

*Xylopiaunguiculata* may prove to be more widely distributed, but as known at present it is endemic to the Congo Subregion within the Congolian Biogeographic Region of Linder et al. (2012). It is of potential conservation concern, with an EOO of 15,417 km^2^ and AOO of 20 km^2^.

### 45. Xylopia villosa

Taxon classificationPlantaeMagnolialesAnnonaceae

Chipp, Kew Bull. Misc. Inform. 1923: 183. 1923.

C2B250C4-0FDF-5CD4-9774-CA31A6346768

Fig. 48A–G

Xylopiastrum villosum (Chipp) Aubréville, Flor. For. Côte d’Ivoire, ed. 2, 1: 140. 1959. Type. NIGERIA. Lagos State, Ibadan Forest Reserve, Lagos, 17 Nov 1900, *C. Punch 119* (lectotype, here designated: K! [000199069]).

#### Description.

***Tree*** up to 30 m tall, d.b.h. up to ca. 90 cm, bole cylindrical, fluted at the base, forming narrow concave buttresses ca. 1 m high and extending up to 50 cm from the trunk, branches horizontal from trunk, forming a pyramidal crown [ex *Bernardi 8679*]; bark pale gray to brownish orange, rough or longitudinally wrinkled. ***Twigs*** initially brown, densely villous, with erect orangish or reddish brown hairs 0.5–1.3 mm long, eventually gray-brown to black, sparsely pubescent to glabrate; nodes occasionally with two axillary branches. ***Leaf*** with larger blades 8.6–12.6 cm long, 2.6–4.1 cm wide, subcoriaceous, discolorous, lanceolate to lanceolate-elliptic or oblong-lanceolate, apex acute to acuminate, the acumen 4–13 mm long, base broadly cuneate to rounded, glabrous or with a few hairs along the midrib adaxially, golden-sericeous, sometimes only sparsely so, abaxially; midrib impressed adaxially, raised abaxially, secondary veins weakly brochidodromous, 10–15 per side, diverging at 45–55° from the midrib, these and higher order veins indistinct or slightly raised on both surfaces; petiole 2–4 mm long, canaliculate, villous. ***Inflorescences*** axillary, 1–8-flowered, but branch apices sometimes with terminal bud aborting and the distalmost nodes lacking leaves so that a congested pseudo-terminal inflorescence is formed, densely pubescent; peduncles 1 or 2 per axil, sometimes developing into an extended rachis 8.5–17 mm long with flower pedicels branching from it, and with bracts ca. 7.4 mm long; pedicels 2–7 per peduncle or rachis, 2.4–4.5 mm long, 1.5–2 mm thick; bracts 2–4, spaced evenly along the pedicel, imbricate, more or less persistent, 3.0–3.7 mm long, ovate to nearly circular but sometimes split in the middle, apex rounded or emarginate; buds lanceolate, slightly angled in cross-section, apex acute to obtuse. ***Sepals*** somewhat spreading at anthesis, 1/4–1/2-connate, 4.3–5.5 mm long, 3.3–4.1 mm wide, coriaceous or a little fleshy, ovate, apex acute, warty, warts visible where hairs abraded, densely appressed-pubescent abaxially. ***Petals*** light yellow *in vivo*; outer petals slightly spreading at anthesis, (22–) 27–34 mm long, 3.5–3.9 mm wide at base, 1.7–2.3 mm wide at midpoint, coriaceous, linear, apex acute to obtuse, densely pubescent on both surfaces except for glabrous base adaxially; inner petals probably more or less erect at anthesis, connivent at broadest point of the base but slightly spreading toward apices, 17.4–23 mm long, 2.5–3.6 mm wide at base, 1.2–1.7 mm wide at midpoint, coriaceous, linear, apex acute, base with undifferentiated margin, with slight transverse thickening adaxially at widest point, densely pubescent except medial portion below the midpoint, but with a transverse strip of hairs across widest point of base adaxially, densely pubescent except at base abaxially. ***Stamens*** 60–80; fertile stamens 1.1–1.8 mm long, narrowly oblong, apex of connective 0.2–0.3 mm long, dome-shaped to shieldlike, overhanging the anther thecae, papillate, anthers 9–12-locellate, filament 0.4–0.5 mm long; outer staminodes 1.7–2.0 mm long, clavate, apex rounded; inner staminodes 0.8–1.1 mm long, oblong, apex truncate; staminal cone 1.6–2.0 mm in diameter, ca. 0.8 mm high, partially concealing the ovaries, rim laciniate. ***Carpels*** 10–12; ovaries 1–1.3 mm long, oblong, densely white-pubescent, stigmas connivent with tips free, 2.5–4 mm long, linear, densely pubescent at apices and sparsely pubescent along sides. ***Torus*** flat, 2.5–2.7 mm in diameter. ***Fruit*** of up to 10 sparsely pubescent monocarps borne on a pedicel 6–42 mm long, 7–11 mm thick, glabrate; torus 14–33 mm in diameter, 8–27 mm high, depressed-globose. ***Monocarps*** with a green exterior *in vivo*, endocarp color unknown, ca. 4.6 cm long, 2.3 cm wide, 2.5 cm thick, oblong, apex rounded, base sessile or contracted into a stipe ca. 3 mm long, 9 mm thick, slightly wrinkled and verrucose; pericarp 3–4 mm thick. ***Seeds*** unknown, apart from illustration of Aubréville (1959, Plate 37, Fig. 10), where the seed illustrated is semicircular in lateral view, truncate at micropylar end, rounded at chalazal end, and lacking an aril.

**Figure 48.**
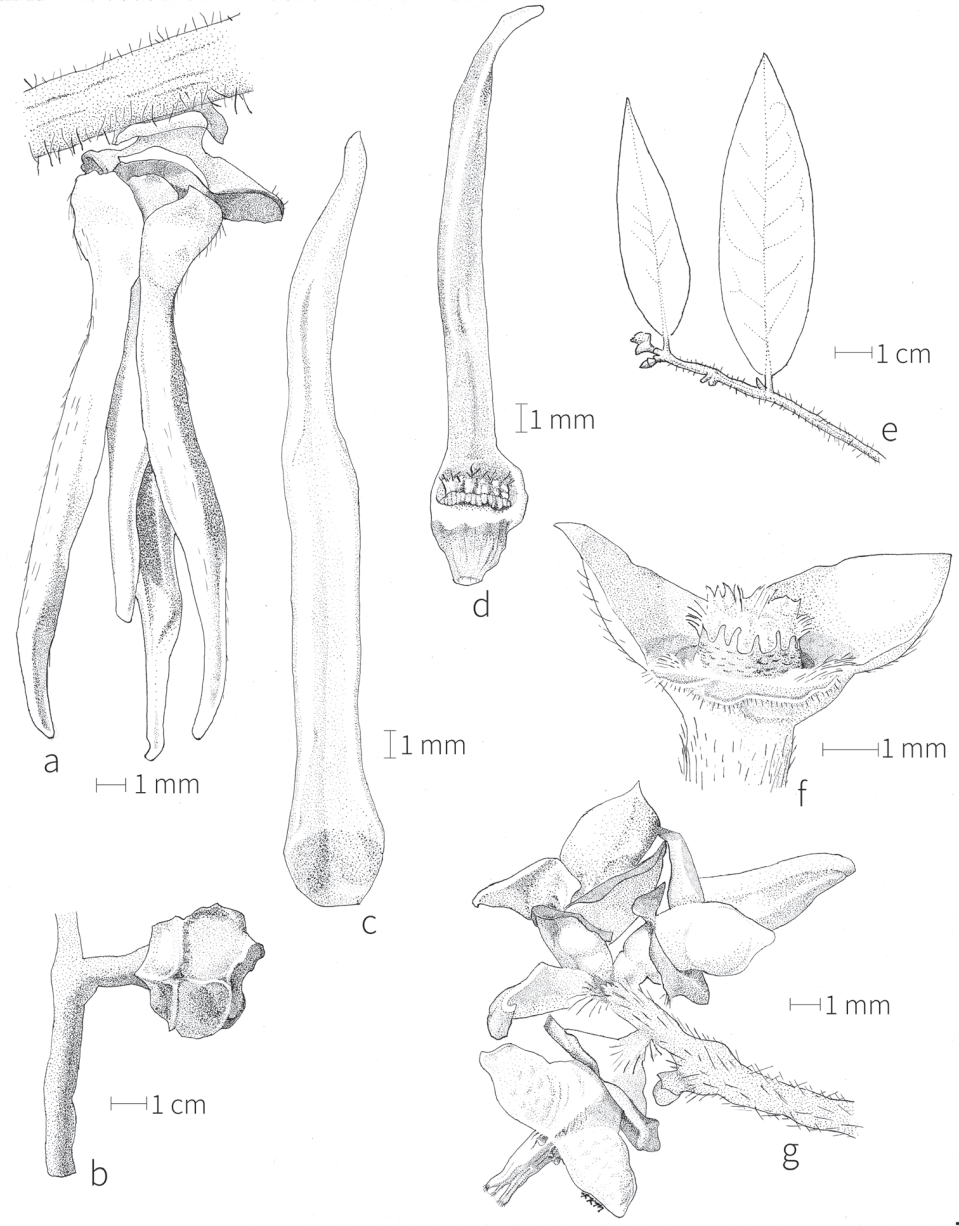
*Xylopiavillosa*. **A** Flower, lateral view **B** Pedicel and torus of fruit **C** Outer petal, adaxial view **D** Inner petal, adaxial view **E** Shoot **F** Torus with sepals and staminal cone attached **G** Close-up of portion of inflorescence. **A, C–G** from *Bernardi 8679* (A) **B** from *Chevalier 22516* (P).

#### Phenology.

Collections with flowers have been gathered from October to December, and in February and March (Aubréville (1959) gives the flowering period in Ivory Coast as November to April). The collections with fruits are from March, April, and December, but Aubréville (1959) gives June as the time of fruiting.

#### Distribution

(Fig. 43). Occurs near the coast from Ivory Coast to southern Cameroon, growing in evergreen forest, semi-deciduous forest of Sterculiaceae and Ulmaceae, and old secondary forest with *Lophiraalata*, *Coulaedulis*, and *Sacoglottisgabonensis*, at ca. 100 m.

#### Local names.

Aghako (Benin, *Kennedy 417*), elo (*Aubréville 198, 1159*), oda’ (*Thomas 1951*), ohun (Benin, *Kennedy 2574*), orogbo-erin (*Foster 354*), oyakwi (Yaoundé, *Letouzey 9524*), unien (Benin, *Kennedy 415*), and palufon (Dudu, *Punch 119*). Burkill (1985) lists additional names, but given the confusion regarding the identification of this species, those names and uses are not recounted here.

#### Additional specimens examined.

**IVORY COAST.** Abidjan, *Aubréville 198* (A, P); Danipleu, 31 Mar 1932 (fl), *Aubréville 1159* (P); in vicinioribus Yapo-Nord, ad orientem oppidi, 15–16 Mar 1962 (fl), *Bernardi 8679* (A, F, K, M, MO, P, US, WAG—4 sheets); Ano, entre Eterokrou [7°15'N, 3°46'W] et Tchoumkrou, 8 Dec 1909 (fl), *Chevalier 22516* (B, K, OWU, P—5 sheets); Abidjan, Banco Forest Reserve, near Esso station, c. 5°23'N, 4°03'W, 28 Apr 1976 (fr), *de Koning 6823* (MO). **GHANA.** Dunkwa [5°58'N, 1°47'W], s. d. (st), *Vigne 81* (K); “Gold Coast” [without specific locality], s. d., *Vigne 2561* (BM). **NIGERIA.** S. Nigeria, Sapoba, 1929 (fl), *Kennedy 415* (K); S. Nigeria, Sapoba, 1929–30 (fl), *Kennedy 417* (K); S. Nigeria, *Kennedy 1983* (K); Sapoba, Feb 1935 (fl), *Kennedy 2574* (A, BM, F, MO, US); Calabar Province, Calabar District, Dukwe felling area, Calabar River Division, 9 Mar 1959 (fr), *Latilo FHI 40346* (K); Sapoba, 29 Nov 1934 (fl), *Ross 236* (K); Degema District, southern Nigeria, 1916 (fl), *Talbot & Talbot 3775* (BM, K); S. Nigeria, Ala, 350’, 2 Nov 1912 (buds), *Thomas 1951* (K). **CAMEROON.** 2 km E of km 58 of road Edéa-Kribi, ca. 100 m, 5 Oct 1965 (fl), *Leeuwenberg 6815* (BR, K, MO, P, WAG—2 sheets); près Nkomeyo, 10 km E d’Esse, 7 Nov 1969 (fl), *Letouzey 9524* (P).

*Xylopiavillosa* has subcoriaceous leaves with shining abaxial hairs, a branching inflorescence, and large monocarps. The limits of its distribution have been unclear because of confusion with *X.letestui*. The two species share densely hairy young twigs and short-stipitate or sessile monocarps, but *X.villosa* differs in the broadly cuneate to rounded rather, than truncate, leaf bases, slightly longer petioles, and longer outer petals. *Xylopiavillosa* also lacks tufts of hairs on the bases of the inner petals found in *X.letestui*. The monocarps are oblong rather than globose. Sterile specimens resembling *X.villosa*, for example *Small 633* (K, MO) from the Gola Forest of Sierra Leone, suggest that the species may be more widespread in West Africa than indicated here.

Given past confusion, it is necessary to re-evaluate literature on the two species. For example, Holmgren et al. (2004) report the formation of aerial roots on the base of the trunk in *X.villosa*, Yapi et al. (2012) list chemical constituents distilled from the bark, and Koné et al. (2008) report four species of monkeys in Ivory Coast as swallowing the seeds, but no voucher specimens have been cited to document these observations.

In *Forest flora of southern Nigeria*, the voucher specimen cited by Kennedy (1936) as *Hexalobusmonopetalus* (A. Rich.) Engl. is a *Xylopia* species, probably *X.villosa*, described as having white axillary flowers and monocarps “splitting irregularly into three valves which reflex to expose about ten bean shaped seeds packed transversely” against a red endocarp. Dehiscent fruits do not occur in *Hexalobus*, and the specimen was not cited in a 2011 revision of *Hexalobus* (Botermans et al. 2011). A photograph of the spirit collection of the specimen at FHO is plausible for *X.villosa*.

The name *Xylopiamacrocarpa* A. Chevalier (Chevalier 1920) is associated with the specimen *Chevalier 22516* cited above, but the name was published without a validating description. Roberty (1953), in proposing the generic name *Xylopiastrum* and making the combination *Xylopiastrummacrocarpum* (Vahl) Roberty, mentioned the name *Xylopiamacrocarpa* Oliv., which we have been unable to trace; it is possible that this was a copying error for *Xylopiamacrocarpa* Chev. Roberty’s combination was explicitly based, however, on *Unonamacrocarpa* Vahl ex Dunal, which is today identified as *Uvariachamae* (Junghans 1961); the name *Xylopiastrum* Roberty should therefore properly be considered a generic synonym of *Uvaria*, not of *Xylopia*. As Chevalier (1920) cited no basionym, the name *Xylopiamacrocarpa* is treated as a *nomen nudum* and not a new combination.

The specimen *Punch 119* agrees well with the protologue and it is designated as the lectotype of the name *Xylopiavillosa*; the paratype *Foster 354* (K) could not be located at K.

##### Excluded names based on types from continental Africa and published in *Coelocline*, *Habzelia*, *Unona*, *Xylopia*, and *Xylopiastrum*

*Coelocline? polycarpa* (Dunal) A. de Candolle, Mém. Soc. Phys. Genève 5: 209. 1832.

Type: Based on *Unonapolycarpa* Dunal.

= ***Annickiapolycarpa*** (Dunal) Maas & Setten

*Habzelia* A. de Candolle, Mém. Soc. Phys. Genève 5: 208. 1832.

Type: *Habzeliaaethiopica* (Dunal) A. de Candolle.

This name is illegitimate and to be rejected under ICN (2012) Article 52.1 because it included at the time of its publication *Unonadiscreta*, the type of the genus *Unona*, among its four species. The binomials published by Candolle in the genus are likewise not legitimate names. However, the name was taken up by Hooker and Thomson (1855) who placed two species into the genus when it was adopted; the name may therefore be accepted as valid from the date of publication for *Flora Indica*, and one of the two named species chosen as lectotype.

*Habzeliaaethiopica* (Dunal) A. de Candolle, Mém. Soc. Phys. Genève 5: 208. 1832.

Type: Based on *Unonaaethiopica* Dunal, but an illegitimate name under ICN Article 52.1.

= ***Xylopiaaethiopica*** (Dun.) A. Rich.

*Habzeliaundulata* (Palisot de Beauvois) A. de Candolle, Mém. Soc. Phys. Genève 5: 208. 1832.

Type: Based on *Xylopiaundulata* Palisot de Beauvois.

= ***Monodoraundulata*** (Pal. Beauv.) Couvreur

*Unonaalbida* Engler ex Engler & Diels, Notizbl. Königl. Bot. Gart. Berlin 2: 297. 1899.

Type: CAMEROON. South Region, Bipindi, 6 Mar 1898 (fl), *G. A. Zenker 1715* (holotype: B! [100153057]; isotypes: B! [100153058] MO! PRC! WU!).

= ***Sphaerocorynegracilipes*** (Benth.) X. Guo & R. M. K. Saunders (Guo et al. 2017)

*Unonabuchananii* Engler, Pflanzenw. Ost-Afrikas C: 179. 1895.

Type: MALAWI: Without definite locality, 1891 (fr), *J. Buchanan 1152* (lectotype, here designated: B! [100153048]). The two Stuhlmann syntypes, 6238 and 6703, from Usaramo, Kisserewe, apparently do not survive.

= ***Monanthotaxisbuchananii*** (Engler) Verdc. (Verdcourt 1971a).

*Unonacaffra* E. Meyer in Pl. Drege. A nomen nudum, referred to by Sonder (1860) in publication of the name *Guatteriacaffra* E. Meyer ex Sonder, Fl. Cap. 1: 9. 1860.

= ***Monanthotaxiscaffra*** (Sond.) Verdc.

*Unonaconfinis* Pierre, nomen nudum. Cited by Maas et al. (2003) as only a herbarium name.

= ***Duguetiaconfinis*** (Engl. & Diels) Chatrou

*Unonacongensis* Engler & Diels, Notizbl. Königl. Bot. Gart. Berlin 2: 296. 1899.

Type: DEMOCRATIC REPUBLIC OF THE CONGO [“Kongo-Gebiet”]. Équateur Province, Bangala, *M. D. J. Laurent s. n.* (holotype: BR).

= ***Monanthotaxislaurentii*** (De Wild.) Verdc. (Verdcourt 1971a)

*Unonadielsiana* Engler, Bot. Jahrb. Syst. 39: 476–477. 1907.

Type: CAMEROON. South Region, bei Bipinde im Urwald, Dec 1901 (fl), *G. A. Zenker 2473* (holotype: B!; isotypes: B! WAG!).

= ***Monanthotaxisdielsiana*** (Engl.) Hoekstra (Guo et al. 2017)

*Unonaelegans* Engler ex Engler & Diels, Notizbl. Königl. Bot. Gart. Berlin 2: 296. 1899, non Thwaites, 1864.

Type: CAMEROON. South Region, Bipinde, *G. A. Zenker 1321* (holotype: B!).

= ***Monanthotaxiselegans*** Verdc. (Verdcourt 1971a) [Note: as a later homonym, *Unonaelegans* Engl. & Diels is not valid as a basionym.]

*Unonaeminii* Engler, Pflanzenw. Ost-Afrikas C: 179. 1895.

Types: “Bu.” [= Bukoba?], *F. L. Stuhlmann 1556*, *F. L. Stuhlmann 4022* (B, not found).

= ***Monanthotaxisferruginea*** (Oliv.) Verdc. (Verdcourt 1971a)

*Unonaferruginea* Oliver, Fl. trop. Afr. 1: 35–36. 1868.

Type: ANGOLA [“Lower Guinea”]. Cuanza Norte Province, Golungo Alto and Cazengo [Dist. Golungo, 1000–2400’], *F. Welwitsch 761* (isotype: B [100153029])

= ***Monanthotaxisferruginea*** (Oliv.) Verdc., (Verdcourt 1971a)

Unonaferrugineavar.brevifolia Engler, Pflanzenw. Ost-Afrikas C: 179. 1895.

Type: TANZANIA. Usaramo, *s. coll*.

= ***Monanthotaxistrichocarpa*** (Engl. & Diels) Verdc. (Verdcourt 1971b, p. 103)

*Unonaglauca* Engler & Diels, Notizbl. Königl. Bot. Gart. Berlin 2: 296–297. 1899.

Type: GABON [“Gabun”]. Munda, Sibange, 6 Feb 1881 or 1882 (fr), *H. Soyaux 203* (lectotype, designated by Hoekstra in Guo et al. 2017: B; isolectotype: K).

= ***Monanthotaxismontana*** (Engl. & Diels) Hoekstra (Guo et al. 2017)

*Unonahirsuta* Bentham, Trans. Linn. Soc. 23: 469. 1862.

Type: EQUATORIAL GUINEA. Bioco, Fernando Poo, 1860, *G. Mann 559* (holotype: P [00363313]; isotypes: K [000198950], P [00363314]).

= ***Monanthotaxishirsuta*** (Benth.) Hoekstra (Guo et al. 2017)

Unona?lepidota Oliver, Fl. trop. Afr. 1: 36. Oct 1868.

Type: As for *Unonaoliveriana* Baillon below.

*Unonalucidula* Oliver, Fl. trop. Afr. 1: 35. 1868.

Type: ANGOLA [“Lower Guinea”]. Malanje Province, Pungo Andungo, *F. Welwitsch 762* (lectotype, designated by Paiva 1966, p. 44: LISU).

= ***Monanthotaxislucidula*** (Oliv.) Verdc. (Verdcourt 1971a)

*Unonamacrocarpa* Vahl ex Candolle ex Dunal, Monogr. Anonac. 103–104. Aug–Nov 1817.

Types: GHANA. “Hab. in Guineâ,” *P. Thonning s. n., P. E. Isert s. n.* (DC. v. s. herb. Juss.)

= ***Uvariachamae*** Pal. Beauv.

*Unonamillenii* Engler & Diels, Monogr. afrik. Pflanzen-Fam. 6: 40. 1901.

Type: NIGERIA [“Oberguinea”]. Lagos State, Lagos, Mar 1896 (fl), *H. Millen 149* (holotype: K).

= ***Monanthotaxisgracilis*** (Hook. f.) Hoekstra (Guo et al. 2017)

*Unonamontana* Engler & Diels, Notizbl. Königl. Bot. Gart. Berlin 2: 296. 1899.

Type: CAMEROON. Centre Region, Yaoundé [“Yaunde”], 1894, *G. A. Zenker & A. Staudt 431* (holotype: B).

= ***Monanthotaxismontana*** (Engl. & Diels) Hoekstra (Guo et al. 2017)

*Unonaobanensis* E.G. Baker, Cat. Talbot’s Nigerian Pl. 4. 1913.

Type: NIGERIA. Cross River State, Oban, 1911, *P. A. Talbot 1246* (holotype: BM).

= ***Monanthotaxisenghiana*** (E.G. Baker) Hoekstra (Guo et al. 2017)

*Unonaobovata* Bentham, Trans. Linn. Soc. London 23: 469. 1862.

Type: MOZAMBIQUE [“Mozamb. Distr.”]. Zambezia Province, foot of Moramballa, Zambesia, 31 Dec 1858 (fl), *J. Kirk s. n.* (holotype: K; isotype: B).

= ***Monanthotaxisobovata*** (Benth.) Hoekstra (Guo et al. 2017)

*Unonaoliveriana* Baillon, Adansonia 8: 307. May–June 1868.

Type: EQUATORIAL GUINEA. Muni River, Aug 1862, *G. Mann 1774* (lectotype, here designated: K! [000795931]; isotypes: K! [000795932], P! [00362615]).

This name appears to be based on the same type as *Unonalepidota* above, for the plant currently known as *Meiocarpidiumlepidotum* (Oliv.) Engl. & Diels, but the name of Baillon (1868) was published several months earlier and has priority. The correct name should be ***Meiocarpidiumoliverianum* (Baillon) D. M. Johnson & N. A. Murray, comb. nov.**

*Unonaovata* Vahl ex Candolle ex Dunal, Monogr. Anonac. 104. Aug–Nov 1817.

Type: GHANA. “Hab. in Guineâ,”] *P. Thonning s. n.* (DC. v. s. h. Juss.).

= ***Uvariaovata*** (Dun.) A. DC. subsp. ***ovata*** (Keay 1954–1958)

*Unonaovata* var. ß *afzeliana* Candolle, Prodr. 1: 89. 1824.

Type: “Priori fere similes, sed folia adulta omnino (excepto nervo longitudinali) glabra fusco-ferruginea. (v. s. in h. Lamb.)”

= ***Uvariaovata*** (Dun.) A. DC. subsp. ***afzeliana*** (DC.) Keay (Keay 1954–1958)

*Unonaparvifolia* Oliver, Fl. trop. Afr. 1: 36, 1868.

Type: ANGOLA [“Lower Guinea”]. Cuanza Norte Province, Golungo Alto, *F. Welwitsch s. n.* (holotype: LISU [P. Hoekstra, personal communication]).

= ***Monanthotaxisparvifolia*** (Oliv.) Verdc. (Verdcourt 1971a)

Unonaparvifoliavar.petersii Engler, Pflanzenw. Ost-Afrikas C: 179. 1895.

Type: MOZAMBIQUE. Sena, *W. C. H. Peters s. n.* (holotype: B).

= ***Cleistochlamyskirkii*** (Benth.) Oliv. (Sprague & Hutchinson 1916, Verdcourt 1971b)

*Unona ? polycarpa* Candolle ex Dunal, Monogr. Anonac. 117–118. Aug–Nov 1817.

Type: “Hab. in Sierra Leonâ,” *A. Afzelius s. n.* (DC. v. s. sinè fl. in herb. Lamb.)

= ***Annickiapolycarpa*** (Dun.) P. Maas & van Setten (Versteegh and Sosef 2007)

*Unonastuhlmannii* Engler, Pflanzenw. Ost.-Afr. C: 179. 1895.

Type: TANZANIA. Pwani Region, Bagamoyo, Feb 1890 (fl, fr), *F. L. Stuhlmann 229* (holotype: B! [100154088]).

= ***Huberanthastuhlmannii*** (Engl.) Chaowasku (Chaowasku et al. 2015).

*Unonaundulata* (Palisot de Beauvois) Dunal, Monogr. Anonac. 111. Aug–Nov 1817.

Type: Based on *Xylopiaundulata* Palisot de Beauvois.

= ***Monodoraundulata*** (Pal. Beauv.) Couvreur (Couvreur 2008).

*Xylopiabokoli* De Wildeman & T. Durand, Ann. Mus. Congo, Sér. 2, Bot. 1(2): 2. 1900.

Type: DEMOCRATIC REPUBLIC OF THE CONGO [“Belgian Congo”]. Équateur Province, Bokakata, 10 Mar 1896 (fl, fr), *A. Dewèvre 785* (holotype: BR!; isotypes: BR! [0000008804020, 0000008804358]).

= ***Monanthotaxisbokoli*** (De Wild. & T. Durand) Verdc. (Verdcourt 1971a)

*Xylopiadunaliana* Vallot, Bull. Soc. Bot. France 29: 219. 1882, nomen illegit., non Planchon & Linden, 1863. A nomen novum proposed by Vallot for the plant that had been known as *Xylopiaacutiflora*.

Xylopiaelliotiivar.hedinii Robyns & Ghesquière, nomen nudum on *Zenker 4739* at BR

= ***Greenwayodendronsuaveolens*** (Engl. & Diels) Verdc.

*Xylopiafernandopoana*, nomen nudum on *Guinea 1658*

= ***Xylopiaafricana*** (Benth.) Oliv.

*Xylopiahouttei*, nomen nudum on type specimen of *Xylopiadekeyzeriana*

= ***Xylopiaaethiopica*** (Dun.) A. Rich.

*Xylopialehmbachii* Diels, nomen nudum on *Lehmbach 137a*

= ***Xylopiaafricana*** (Benth.) Oliv.

*Xylopiamacrocarpa* A. Chevalier, Expl. Bot. Afr. Occ. Franç. 1: 15. 1920, nomen nudum

= ***Xylopiavillosa*** Chipp

*Xylopiaotunga* Exell, J. Bot. 69: 99. 1931.

Type: CAMEROON. Centre Region, Bitye, Yaunde, *G. L. Bates 1226* (holotype: BM! [000513697]).

= ***Greenwayodendronsuaveolens*** (Engl. & Diels) Verdc. (Verdcourt 1969)

*Xylopiapoggeana* Engler & Diels, Monogr. afrik. Pflanzen-Fam. 6: 65–66. 1901.

Type: DEMOCRATIC REPUBLIC OF THE CONGO [“Belgian Congo”]. Lualaba Province, Mukenge, s. d. (fr), *P. Pogge 634* (holotype: B, not found). Diels (1915) commented that with study of more fruiting material the placement of *X.poggeana* in *Xylopia* was doubtful, and suggested that it might be more similar to a *Popowia* such as *P.laurentii* De Wild., but that he had not examined material of that species. Verdcourt (1971a) did not discuss the name *Xylopiapoggeana* in his revision of *Monanthotaxis*, where African *Popowia* species are now placed.

*Xylopiapolycarpa* (Candolle ex Dunal) Oliver, Fl. trop. Afr. 1: 32. 1868.

Type: Based on *Unonapolycarpa* Candolle ex Dunal.

= ***Annickiapolycarpa*** (DC. ex Dun.) P. Maas & van Setten

*Xylopiasmithii*, nomen nudum in sched.

= ***Xylopialongipetala*** De Wild. & T. Durand

*Xylopia* [“Xilopia”] *undulata* Palisot de Beauvois, Fl. Owar. 1: 27–28 + t. 16. 20 May 1805 [“1804”].

Type: NIGERIA. Without definite locality, s. d., *A. M. F. J. Palisot de Beauvois s. n.* (holotype: G-DC (Couvreur 2008)).

= ***Monodoraundulata*** (Pal. Beauv.) Couvreur

*Xylopiastrum* Roberty, Bull. Inst. Franç. Afr. Noire 15: 1397. 1953.

Type: *Unonamacrocarpa* Vahl.

= ***Uvariachamae*** Pal. Beauv. (Junghans 1961)

*Xylopiastrummacrocarpum* (Vahl ex Dunal) Roberty, Bull. Inst. Franç. Afr. Noire 15: 1398. 1953.

Type: Based on *Unonamacrocarpa* Vahl ex Candolle ex Dunal

= ***Uvariachamae*** Pal. Beauv.

*Xylopiastrumtaiense* Aubréville, Flor. For. Côte d’Ivoire, ed. 2, 1, 140 + pl. 41, 3-4. 1959. This name was based on the specimen *Aubréville 4090* from Taï, Ivory Coast, but the protologue lacks a Latin diagnosis or description and the name is thus not validly published under Article 39.1 of the ICN (2012).

= ***Xylopiaacutiflora*** (Dun.) A. Rich.

*Xylopicrumpolycarpum* (Candolle ex Dunal) Kuntze, Revis. gen. pl. 1: 8. 1891.

Type: Based on *Unonapolycarpa* Candolle ex Dunal.

= ***Annickiapolycarpa*** (DC. ex Dun.) P. Maas & van Setten

## Supplementary Material

XML Treatment for Xylopia

XML Treatment for Xylopia Section Neoxylopia

XML Treatment for Xylopia africana

XML Treatment for Xylopia globosa

XML Treatment for Xylopia rubescens

XML Treatment for Xylopia staudtii

XML Treatment for Xylopia Section Ancistropetala

XML Treatment for Xylopia aurantiiodora

XML Treatment for Xylopia congolensis

XML Treatment for Xylopia quintasii

XML Treatment for Xylopia Section Xylopia

XML Treatment for Xylopia aethiopica

XML Treatment for Xylopia Section Verdcourtia

XML Treatment for Xylopia lukei

XML Treatment for Xylopia mwasumbii

XML Treatment for Xylopia tenuipetala

XML Treatment for Xylopia Section Stenoxylopia

XML Treatment for Xylopia arenaria

XML Treatment for Xylopia collina

XML Treatment for Xylopia flamignii

XML Treatment for Xylopia gilbertii

XML Treatment for Xylopia gracilipes

XML Treatment for Xylopia holtzii

XML Treatment for Xylopia keniensis

XML Treatment for Xylopia nilotica

XML Treatment for Xylopia odoratissima

XML Treatment for Xylopia shirensis

XML Treatment for Xylopia tomentosa

XML Treatment for Xylopia torrei

XML Treatment for Xylopia toussaintii

XML Treatment for Xylopia wilwerthii

XML Treatment for Xylopia acutiflora

XML Treatment for Xylopia calva

XML Treatment for Xylopia cupularis

XML Treatment for Xylopia dinklagei

XML Treatment for Xylopia elliotii

XML Treatment for Xylopia hypolampra

XML Treatment for Xylopia katangensis

XML Treatment for Xylopia letestui

XML Treatment for Xylopia longipetala

XML Treatment for Xylopia mildbraedii

XML Treatment for Xylopia monticola

XML Treatment for Xylopia paniculata

XML Treatment for Xylopia phloiodora

XML Treatment for Xylopia piratae

XML Treatment for Xylopia pynaertii

XML Treatment for Xylopia talbotii

XML Treatment for Xylopia tanganyikensis

XML Treatment for Xylopia thomsonii

XML Treatment for Xylopia unguiculata

XML Treatment for Xylopia villosa

## Appendix 1

**Voucher specimens used in anatomical and molecular studies**

The descriptions of leaf and floral anatomy by Kramer (1969) were based on the following specimens (all from WAG): *X.acutiflora* (*Voorhoeve 1089*, leaf and flower, originally identified as *X.elliotii*); *X.aethiopica* (*de Wilde 3633*, *Leeuwenberg & Voorhoeve 4924*, both for leaves and flowers); *X.letestui* (*Leeuwenberg 3073*, leaf and flower, originally identified as *X.villosa*); *X.longipetala* (*de Wilde 3236*, leaf and flower, originally identified as *X.parviflora*); *X.piratae* (*Leeuwenberg 2365*, *de Wilde 769*, leaf and flower, both originally identified as *X.acutiflora*); *X.quintasii* (*Leeuwenberg 2406*, *Harley 1294*, *Breteler et al. 2398*, *Voorhoeve 144a*, *Zenker 359* (originally identified as *X.striata*, all leaf and flower), *X.staudtii* (*Voorhoeve 19*, leaf and flower), and *X.thomsonii* (*Breteler 1137*, *Breteler 1063*, leaf and flower, originally identified as *X.acutiflora*).

Voucher specimens for the molecular material of African species, analyzed by Stull et al. (2017), are the following: *Xylopiaaethiopica*, Tanzania, *Johnson 1943A* (OWU); *X.aurantiiodora*, Central African Republic, *Harris 2750* (OWU); *X.flamignii*, Gabon, *Bradley et al. 1136* (OWU); *X.globosa* (as X.sp. aff.africana), Gabon, *Sosef et al. 2180* (OWU, WAG); *X.hypolampra*, Gabon, *Sosef 1866* (WAG); *X.letestui*, Gabon, *SIMAB 012002* (MO); *X.longipetala*, Gabon, *de Wilde 11959*; *X.lukei*, Mozambique, *Luke 10166* (MO); *X.mildbraedii*, Cameroon, *Elad 1253* (WAG); *X.mwasumbii*, Tanzania, *Johnson 1964B* (OWU); *X.quintasii*, Ghana, *Schmidt 2267* (MO); *X.staudtii*, Gabon, *Niangadouma & Walters 144* (MO).

## Appendix 2

**Index to numbered collections**

Abeid 550 (holtzii), [et al.] 1028 (tanganyikensis), [et al.] 1942 (holtzii).

Adam, J.-G., 123 (elliotii), 417 (aethiopica), 5910 (rubescens), 5911 (rubescens), 9261 (longipetala), 14340 (longipetala), 16733 (staudtii), 16813 (staudtii), 17332 (longipetala), 18369 (longipetala), 20994 (aethiopica), 22870 (acutiflora), 23553 (quintasii), 23587 (longipetala), 25287 (staudtii), 25627 (aethiopica), 26378 (staudtii), 26395 (acutiflora), 26904 (longipetala), 27753 (aethiopica), 29894 (staudtii).

Aguiar Macedo [& Balsinhas] 1510 (gracilipes), 3716 (gracilipes), 3274 (rubescens), [& Macuácua] 3291 (aethiopica).

Aké Assi, L., 10378 (rubescens), 10493 (longipetala), 13159 (aethiopica), 16073 (aethiopica), 16784 (aethiopica), 17812 (acutiflora), 19025 (longipetala).

Akpabla, 656 (longipetala), 767 (piratae), 797 (aethiopica).

Allen 44 (aethiopica).

Ambe 186 (aethiopica).

Andel, T. van, et al., 3318 (*Greenwayodendronsuaveolens*), 3345 (quintasii), 4023 (acutiflora).

Andrada, 51 (tomentosa), 51A (tomentosa).

Andrada, E. C., 1529 (gracilipes), 1898 (gracilipes).

Angus, A., 304 (shirensis), 529 (tomentosa), 870 (rubescens), 1942 (rubescens), 3010 (tomentosa).

Annet, S., 319 (quintasii).

Antunes, J. M., 64 (odoratissima), [& De Kindt] 3142 (odoratissima).

Arends, J. C., [et al.] 389 (thomsonii), [et al.] 708 (thomsonii).

Armitage 102/60 (odoratissima).

Aubréville, A., 37 (villosa), 38 (quintasii), 42 (quintasii), 70 (hypolampra), 90 (acutiflora), 108 (quintasii), 125 (rubescens), 133 (aethiopica), 198 (villosa), 241 (longipetala), 458 (quintasii), 493 (staudtii), 507 (rubescens), 526 (aethiopica), 667 (longipetala), 828 (aethiopica), 920 (aethiopica), 1009 (acutiflora), 1109 (acutiflora), 1159 (villosa), 1245 (longipetala), 1329 (aethiopica), 1345 (quintasii), 1422 (longipetala), 1511 (rubescens), 1537 (piratae), 1941 (staudtii), 1943 (quintasii), 1944 (aethiopica), 1945 (quintasii), 2176 (hypolampra), 2201 (longipetala), 2622 (longipetala).

Audru 5205 (longipetala).

Aylmer, G., 29 (acutiflora), 202 (quintasii), 239 (staudtii).

Baldwin 6970 (acutiflora), 9131 (longipetala), 10714 (longipetala), 10805 (dinklagei), 11141 (aethiopica), 14086 (acutiflora).

Bamps, P., 597 (cupularis), 1376 (quintasii), 2006 (longipetala), 2044 (staudtii), 2125 (acutiflora), 2260 (quintasii).

Barbosa, G., 11754 (tomentosa), [& Carvalho] 2862 (aethiopica).

Barter, C., 426 (longipetala), 1035 (longipetala).

Bates, G. L., 561 (rubescens), 1226 (*Greenwayodendronsuaveolens*), 1317 (rubescens), 1813 (aethiopica), 1852 (thomsonii).

Baum, H., 224 (tomentosa).

Beentje, H., 879 (acutiflora).

Benoit, M., 61 (SRFK 1719) (aethiopica), 111 (SRFK 1916) (aethiopica), 121 (phloiodora), 282 (aethiopica), 353 (quintasii), 362 (SRFK 4187) (hypolampra).

Bequaert, J., 2281 (longipetala), 6994 (aurantiiodora).

Berg, C. C., & J. T. Wiebes 1475 (aethiopica).

Bergen, M. A. van, 153 (quintasii), 207 (aethiopica).

Berhaut, R. P., 1035 (aethiopica), 1476 (longipetala), 2145 (longipetala), 4295 (longipetala), 4304 (longipetala), 6207 (longipetala), 6314 (longipetala), 7086 (longipetala).

Bernardi, L., 8315 (acutiflora), 8679 (villosa).

Bester, S. P., 9279 (odoratissima).

Bidgood, S., et al., 642 (shirensis), [et al.] 1535 (collina), 1544 (collina), 1600 (collina), [et al.] 5122 (shirensis).

Bingham, M., 3250 (aethiopica), [et al.] 11492 (tomentosa), [& Luwiika] 11893 (odoratissima), [& Luwiika] 11905 (odoratissima), 14167 (tomentosa).

Binuyo, A., FHI 41375 (aethiopica).

Birch, W. M., 62/188 (holtzii).

Black, J., M.37 (longipetala).

Blyden, F., 94 (aethiopica).

Bolema, D., 372 (phloiodora), 374 (aethiopica), 1192 (aurantiiodora).

Bonnefousc A Villain 92°2 (odoratissima).

Bos, J. J., 2198 (aethiopica), 2754 (longipetala), 3341 (aethiopica), 4650 (talbotii), 4772 (mildbraedii), 4773 (quintasii), 5475 (thomsonii), 6296 (aethiopica), 6382 (aethiopica), 6296 (aethiopica), 6561 (aethiopica).

Boupoya, A., 807 (quintasii).

Bouquet, A., 253 (wilwerthii), 315 (rubescens), 476 (aethiopica), 497 (wilwerthii), 617 (aethiopica), 685 (aethiopica), 808 (aethiopica), 1286 (pynaertii), 1315 (pynaertii), 1447 (flamignii), 1653 (hypolampra), 1996 (pynaertii).

Bourne, R., 96 (katangensis), 124 (katangensis).

Bourobou Bourobou, H. P., 654 (aethiopica), 655 (quintasii), 707 (staudtii), 930 (quintasii), B581 (aethiopica).

Bradley, A. F., et al. 1136 (flamignii).

Brain 8875 (odoratissima).

Bredo 6232 (shirensis), 6276 (shirensis).

Breitenbach, F. von, 1312 (gracilipes), 16459 (gracilipes).

Bremekamp & Schweickerdt 348 (gracilipes).

Brenan, J. P. M., & P. J. Greenway 8022 (rubescens), 8041 (tomentosa), 8044 (tomentosa).

Brent, R. W., 389 (quintasii).

Breteler, F. J., [& J. J. F. E. de Wilde] 246 (aethiopica), 931 (thomsonii), 1063 (thomsonii), 1137 (thomsonii), [et al.] 2398 (quintasii), 2797 (thomsonii), 5251 (aethiopica), [& R. A. van Raalte] 5563 (aethiopica), [& R. A. van Raalte] 5564 (thomsonii), [& R. A. van Raalte] 5612 (aethiopica), 5856 (piratae), 5983 (piratae), 7445 (piratae), [et al.] 8669 (hypolampra), [et al.] 8867 (letestui), [et al.] 9526 (mildbraedii), [& C. Jongkind] 10111 (thomsonii), [& C. Jongkind] 10446 (rubescens), [& C. Jongkind] 10458 (aethiopica), [& C. Jongkind] 10470 (quintasii), [& C. Jongkind] 10590 (hypolampra), 11626 (aethiopica), [& B. J. M. Breteler-Klein] 12632 (mildbraedii), [et al.] 12944 (aethiopica), [et al.] 12978 (globosa), [et al.] 13000 (globosa), 15468 (cupularis), 15795 (quintasii).

Breyne, H., 86 (longipetala), 153 (longipetala), 864 (rubescens), 934 (aethiopica).

Briey, Comte J. de, 108 (hypolampra), 219 (staudtii).

Brummitt, R. K., et al., 14068 (tomentosa), 14111 (tomentosa), 14224 (odoratissima).

Brunt, M., 261A (elliotii).

Buchanan, J., 237 (shirensis).

Bullock, A. A., 1414A (odoratissima).

Buonounou Ouétien 35 (elliotii).

Burgt, X. M. van der, 328 (aethiopica), [& E. A. Laan] 364 (hypolampra), 662 (thomsonii), 1129 (letestui), 1167 (elliotii), 1305 (quintasii), 1722 (africana).

Burrows, J., [& S. Burrows] 9848 (gracilipes), [& S. Burrows] 10746 (tenuipetala), [& S. Burrows] 10942 (collina), [et al.] 11390 (lukei).

Burtt 5459 (shirensis).

Busse, W., 2858 (collina).

Butaye, R. P., coll. J. Gillet 2239 (rubescens).

Butt 45 (staudtii).

Bytebier et al. B 3367 (staudtii).

Cable 2870 (africana), 3814 (africana).

Caille 14771 (aethiopica), 14812 (aethiopica), 18149 (elliotii).

Callens, H., 2440 (tomentosa), 2730 (tomentosa), 3056 (tomentosa), 3850 (tomentosa), 3983 (tomentosa).

Carrington 7 (wilwerthii).

Carrisso, L. W. & F. de A. Mendonça 506 (tomentosa).

Carroll 1077 (aethiopica).

Carvalho 2646 (rubescens), 4746 (aethiopica), 4751 (aethiopica).

Centre Technique Forestier Tropical (CTFT) 170 (aethiopica).

César 553 (longipetala).

Chalot 45 (aethiopica).

Chapman, J. D., 494 (aethiopica), 2739 [FHI 45180] (monticola), 3755 (monticola), 3786 (monticola), 4618 (monticola), 4671 (monticola), 4730 (monticola), 5186 (monticola), [& E. G. Chapman 6829] (aethiopica), [& E. G. Chapman] 6921 (aethiopica).

Chase 7238 (gracilipes).

Cheek, M., 7605 (africana), 9192 (africana), 9923 (thomsonii), 10527 (africana), 11057 (aethiopica), 11487 (hypolampra).

Chevalier, A., 2545 (aethiopica), 4086 (longipetala), 4094 (longipetala), 4305 (aethiopica), 5268 (longipetala), 6363 (longipetala), 7331 (aethiopica), 7741 (elliotii), 16118 (quintasii), 16321 (rubescens), 16321 bis (rubescens), 19113 (longipetala), 19912 (aethiopica), 22516 (villosa), 22767 (aethiopica), 23299 (longipetala), 23586 (longipetala), 24177 (aethiopica), 27105 (rubescens), 27746 (longipetala), 28289 (aurantiiodora), 28331 (aethiopica), 28389 (longipetala), 34547 (longipetala).

Chiponja 2032 (hypolampra).

Choo, J., 764 (aethiopica).

Codd, L. E., 6806 (gracilipes).

Collinson U.9 (shirensis).

Compere 496 (longipetala).

Congdon, T. C. E., 558 (shirensis), 588 (shirensis), 673 (katangensis), 709 (shirensis).

Cooper, G. P., 60 (staudtii), 139 (staudtii), 174 (aethiopica), 222 (quintasii), 234 (staudtii), 337 (quintasii), 372 (quintasii), 464 (quintasii).

Corbisier-Baland, A., 806 (aethiopica), 1627 (phloiodora), 1687 (aurantiiodora).

Couteaux 236 (aethiopica), 425 (aurantiiodora).

Couvreur, T. L. P., [et al.] 18 (aethiopica), [et al.] 688 (letestui), [et al.] 912 (aethiopica), [et al.] 925 (aethiopica), 949 (africana).

Cusset, G. 1200 (globosa).

Dalziel, J. M., 713 (longipetala).

Dam, G. van, 22934 (gracilipes).

Daramola, B. O., 434 (quintasii), 441 (aethiopica), FHI 45672 (cupularis), FHI 45673 (quintasii).

Davio, C., 10 (cupularis).

Dawe, M., 43 (aethiopica), 118 (aethiopica), 229 (aethiopica).

De Koning, J., 84 (piratae), 983 (piratae), 985 (quintasii), 1555 (piratae), 4009 (quintasii), 4070 (rubescens), 4077 (staudtii), 4999 (piratae), 5000 (piratae), 6856 (piratae), 5214 (longipetala), 5296 (rubescens), 5996 (acutiflora), 6615 (rubescens), 6823 (villosa), 6827 (quintasii), 6833 (staudtii), 6854 (rubescens), 6855 (staudtii).

De Winter, B., 3845 (tomentosa), 3879 (tomentosa).

De Witte 4121 (rubescens), 5144 (shirensis)

Deaw, J., Sp 463 (quintasii), Sp 465 (letestui).

Deighton, F. C., 1081 (longipetala), 2997 (longipetala), 3958 (longipetala), 4153 (quintasii).

Deistel 154 (africana), 454 (africana).

Descoings, B., 5719 (longipetala), 5852 (wilwerthii), 5904 (rubescens), 8272 (aethiopica).

Devred, R., 885 (aethiopica), 1420 (tomentosa), 1824 (tomentosa), 2510 (aethiopica), 2565 (cupularis), 2585 (aethiopica), 3083 (wilwerthii).

Dewèvre, A., 660 (aurantiiodora), 785 (*Monanthotaxisbokoli*), 876 (longipetala).

Dhetchuvi 756 (aethiopica), 874 (aethiopica), 1067 (aurantiiodora).

Dinklage, M., 1760 (dinklagei), 1760 bis (dinklagei), 1840 (dinklagei), 1858 (dinklagei), 1873 (dinklagei), 2005 (aethiopica), 2006 (rubescens).

Donis, C., 368 (wilwerthii), 1354 (aethiopica), 1441 (cupularis), 1617 (hypolampra), 2041 (wilwerthii), 2065 (toussaintii), 3261 (aethiopica), 3404 (cupularis), 3634 (gilbertii).

Doumenge, C., 313 (thomsonii).

Drummond & Hemsley 4015 (holtzii), 4714 (aethiopica).

Dubois 485 (aurantiiodora), 780 (aethiopica), 972 (aurantiiodora).

Duboislouveau 903 S. R. F. (phloiodora).

Duff, C. E., 134/33 (katangensis).

Duparquet, R. P., cat. 1864 N°1 n°1 (aethiopica).

Duvall 371 (elliotii), 513 (elliotii).

Dybowski, J., 129 (katangensis), 669 (longipetala).

Eaux Forêts et Chasses 1946 (aethiopica), 1989 (aethiopica).

Eggeling, W. J., EG 1583 [=1524 at K?] (aethiopica), 1650 (nilotica), E.5775 (rubescens), E.6386 (collina), 6241 (aethiopica).

Ekuba 1615 (cupularis).

Ekwuno et al. PFO.150 (FHI 87627) (longipetala).

Elad, M., 1253 (mildbraedii).

Engler, A., 2173 (arenaria).

Enti, A. A., 890 (rubescens), FE-1315 (staudtii), FE-1878 (aethiopica), FH 6881 (quintasii), FE-2095 (staudtii), R.1002 (aethiopica), [& P. R. Awriah] R1136 (rubescens).

Etuge, M., [& D. Thomas] 56 (quintasii), [& D. Thomas] 148 (aethiopica).

Evrard, C., 813 (quintasii), 1594 (aurantiiodora), 1658 (aurantiiodora), 2262 (aethiopica), 2574 (longipetala), 2964 (aurantiiodora), 3436 (aethiopica), 3601 (aurantiiodora), 3849 (phloiodora), 4431 (aurantiiodora), 5212 (staudtii), 5666 (pynaertii), 6059 (pynaertii), 6189 (letestui), 6266 (cupularis), 6317 (wilwerthii), 6620 (longipetala).

Exell, A. W., [& F. A. Mendonça] 370 (tomentosa), [& F. A. Mendonça] 374 (tomentosa), 490 (aethiopica), [et al.] 598 (torrei), 620 (aethiopica), [& F. A. Mendonça] 1024 (tomentosa), [& F. A. Mendonça] 1055 (tomentosa), [& F. A. Mendonça] 1064 (tomentosa), [& F. A. Mendonça] 1101 (tomentosa), [& F. A. Mendonça] 1102 (tomentosa), [& F. A. Mendonça] 1310 (tomentosa), [& F. A. Mendonça] 1430 (tomentosa), [& F. A. Mendonça] 1431 (tomentosa), [& F. A. Mendonça] 1632 (tomentosa), [& F. A. Mendonça] 1790 (tomentosa), 2030 (tomentosa).

Explorações Botânicas 1717 [=1709] (aethiopica), 1809 (aethiopica), 3171 (longipetala), 3206 (longipetala), 3780 (elliotii).

Faden, R., 70/201 (holtzii), 70/847 (keniensis), 71/696 (arenaria), [& Faden] 74/306 (aethiopica), [& Faden] 74/1225 (arenaria).

Fanshawe, D. B., F.390 (tomentosa), 1423 (katangensis), 1447 (aethiopica), 3557 (rubescens), F.3989 (katangensis), F10170 (rubescens).

Farrell, J. A., 202 (gracilipes).

Farron, C., 4892 (staudtii).

Fay, J. M., 4121 (aethiopica), 4191B (elliotii), 4208 (elliotii), 4351 (elliotii), 4359 (elliotii), 6551 (elliotii), 6639 (rubescens), 6641 (elliotii), 8307 (longipetala), [& D. Harris] 8457 (phloiodora), [& D. Harris] 8535 (cupularis), [& D. Harris] 8546 (cupularis).

Fidalgo de Carvalho 685 (gracilipes).

Flamigni 9026 ter (flamignii).

Fleury, F., 26582 (aethiopica), 26594 (quintasii), 33135 (rubescens), 33338 (aethiopica), 33517 (quintasii), 33530 (aethiopica), 33531 (aethiopica), 33532 (aethiopica).

Floret, J. J., et al. 1804 (longipetala).

Fogiel, M., 864 (staudtii), 2098 (letestui).

Foster, E. W., s. n. or 99 (longipetala), 208 (aethiopica).

Fotius, G., 2984 (elliotii), 3108 (elliotii).

Foury 36 (quintasii), 101 (hypolampra).

Fyffe, R., 18 (aethiopica).

Gamwell 106 (shirensis).

Gaston, A. 2488 (longipetala).

Gbile 5018 (longipetala).

Geerling, C., [& J. Bokdam] 1625 (longipetala), [& J. Bokdam] 2314 (quintasii), [& J. Bokdam] 2478 (acutiflora), 3098 (longipetala).

Gentry, A., [& G. Pilz] 32875 (quintasii), 33219 (staudtii), 33323 (aethiopica), [et al.] 62649 (phloiodora).

Gerard, P., 1532 (aethiopica), 1611 (thomsonii), 2162 (aurantiiodora), 4346 (longipetala), 5574 (aethiopica), 5583 (cupularis), 5616 (aethiopica).

Gereau, R. E., et al. 7565 (phloiodora), 7632 (aurantiiodora).

Germain, R., 737 (aethiopica), 2793 (rubescens), 4362 (longipetala), 7368 (aurantiiodora), 7631 (staudtii), 8202 (aethiopica).

Giess, W., 11354 (tomentosa).

Gilbert, 18 (phloiodora), 291 (aurantiiodora), 1054 (phloiodora), 1099 (gilbertii), 1164 (aethiopica), 1423 (gilbertii), 7664 (aethiopica), 7739 (katangensis), 7893 (aethiopica), 8967 (phloiodora), 9018 (aethiopica), 9060 (katangensis), 9344 (katangensis), 10145 (aethiopica), 14335 (flamignii).

Gilges, W., 156 (tomentosa).

Gillardin, J., 352 (katangensis).

Gillett, J. B., & Kibuwa 19846 (holtzii), 19847 (holtzii).

Gillet, J., 207 (aethiopica), 812 (congolensis), [for R. P. Butaye] 2239 (rubescens).

Gillman, H., 398 (aethiopica), 1055 (collina), 1261 (aethiopica), 1293 (collina), 1465 (lukei).

Gisau 10 (arenaria).

Goldsmith 51/56 (odoratissima), 159/68 (gracilipes).

Goossens, V., 4572 (aethiopica).

Gosline, G., 423 (africana).

Gossweiler, J., 2054 (tomentosa), 2102 (tomentosa), 2562 (tomentosa), 3463 (tomentosa), 3464 (tomentosa), 3465 (tomentosa), 3563 bis (tomentosa), 3564 (tomentosa), 3567 (tomentosa), 3672 (tomentosa), 3733 (tomentosa), 3940 (odoratissima), 4043 (odoratissima), 4044 (tomentosa), 6169 (wilwerthii), 6185 (cupularis), 6222 (rubescens), 6275 (*Greenwayodendronsuaveolens*), 6532 (hypolampra), 6612 (aethiopica), 6845 (quintasii), 6903 (aethiopica), 6933 (cupularis), 6988 (paniculata), 7032 (aethiopica), 7168 (aurantiiodora), 7169 (aurantiiodora), 7225 (hypolampra), 7845 (aethiopica), 7992 (staudtii), 8658 (longipetala), 8741 (toussaintii), 8747 (rubescens), 8937 (longipetala), 8957 (rubescens), 8972 (aethiopica), 9006 (aurantiiodora), 9060 (aethiopica), 9073 (aethiopica), 9126 or 9126a (rubescens), 9134 (aethiopica), 9138 (aethiopica), 9151 (toussaintii), 9903 (cupularis), 11202 (tomentosa), 11650 (thomsonii), 11833 (tomentosa), 12340 (tomentosa), 13648 (thomsonii), 14110 (thomsonii), 14222 (aethiopica).

Goyder, D. J., et al. 5037 (torrei), 5084 (collina), 5089 (tenuipetala), 6090 (tenuipetala).

Graer, P. A. M. de, 115 bis (thomsonii + non-*Xylopia*), 241 (thomsonii).

Greenway, P. J., 1006 (aethiopica), 4909 (aethiopica), 4959 (holtzii), 5452 (aethiopica), [& Trapnell] 5573 (rubescens), 9471 (holtzii), [& W. J. Eggeling] 7251 (nilotica).

Griffon du Bellay, Cat. 3, 37 (aethiopica).

Grison FG 22/RC 827 (phloiodora), FG 87/RC 892 (aethiopica).

Groenendijk, L., et al. 856 (gracilipes).

Guigonis 2320 (phloiodora), 3078 (phloiodora).

Guinea, E., 1658 (africana).

Haegens & van der Burgt 233 (aethiopica).

Haerdi, F., 214/0 (holtzii).

Hafashimana 0504 (staudtii).

Hagerup 725 (longipetala).

Hall, A. V., 469 (gracilipes).

Hall, J. B., 1450 (elliotii), GC43251 (staudtii), [& Abbiw] GC45145 (quintasii).

Hallé, F., 4240 (quintasii), 4274 (letestui).

Hallé, N., [& A. Le Thomas] 104 (phloiodora), [& A. Le Thomas] 122 (letestui), [& A. Le Thomas] 190 (longipetala), [& A. Le Thomas] 379 (paniculata), [& A. Le Thomas] 487 (hypolampra), [& A. Le Thomas] 581 (aethiopica), 961 (thomsonii), 1350 (mildbraedii), 3545 (thomsonii), 3735 (gilbertii), 3736 (hypolampra).

Hammerstein 5838 (shirensis).

Harder, D. K., et al. 1918 (katangensis), 2126 (aethiopica), 3145 (rubescens), 3164 (rubescens), 3406 (staudtii), 3644 (tomentosa).

Hardy, D. S., 6914 (gracilipes).

Harris, B. J., [et al.] DSM 2606 (arenaria), [& Harris] 4989 (arenaria), [& Schlieben] BJH 5346 (arenaria), [et al.] 5859 (arenaria), [& Siddiqi] BJH 6749 (arenaria), 6771 (arenaria).

Harris, D. J., [& J. M. Fay] 222 (hypolampra), [& J. M. Fay] 266 (hypolampra), [& J. M. Fay] 319 (hypolampra), [& J. M. Fay] 447 (aethiopica), [& J. M. Fay] 479 (aethiopica), [& J. M. Fay] 536 (aethiopica), 607 (thomsonii), [& J. M. Fay] 752 (aurantiiodora), [& J. M. Fay] 757 (longipetala), [& J. M. Fay] 1132 (aethiopica), [& J. M. Fay] 1535 (longipetala), [& J. M. Fay] 1846 (aurantiiodora), 2622 (phloiodora), 2750 (aurantiiodora).

Hart, T. B., 68 (thomsonii), 284 (thomsonii), 572 (cupularis), 628 (phloiodora), TH 1177 (phloiodora), (Abasi) 1654 (phloiodora).

Hauzer 18 (congolensis), 29 (staudtii).

Hawthorne, W., 1360 (mwasumbii), 1469 (mwasumbii), 1714 (mwasumbii), 1750 (arenaria), 1790 (mwasumbii).

Hedin 1668 (rubescens).

Heitz 3 (quintasii).

Hemm, G., 881 (gracilipes).

Hepper, F. N., 1143 (longipetala).

Herbar. Huíla 276 (odoratissima).

Herman 2212 (katangensis).

Heudelot 566 (aethiopica).

Hilbert, E., 158 (odoratissima).

Hladik, A., 1470 part A (aethiopica), 1470 part B (aethiopica), 1470 part C (aethiopica), 1540 part A (thomsonii), 1540 part B (thomsonii), 1689 part A (congolensis), 1689 part C (congolensis), 1924 part A (thomsonii), 1924 part C (thomsonii), 1943a (aethiopica), 2334 (staudtii).

Holmes, W. D., 1056 (tomentosa), H.1179 (aethiopica), 1192 (rubescens), H.1273 (*Xylopia* sp.).

Holtz, 321 (arenaria), 393 (arenaria), 897 (holtzii), 3202 (holtzii).

Hombert 475 (quintasii).

Hopkins, B., FHI 54307 (africana).

Hopper, R. L., 1 (rubescens).

Hoyle, A. C., 823 (thomsonii), 1329 (katangensis).

Hulstaert, G., 203 (aurantiiodora).

Hutchinson, J., 2240 (gracilipes).

Institute D’Enseignement et de Recherches Tropicales (Adiopodoume) 4069 (longipetala).

Irvine 2299 (piratae).

Itoh & Sakamaki NI97-62 (tanganyikensis).

Jackson, J. K., 3894 (thomsonii).

Jackson-Etukendo UIH 281169 (longipetala).

Jacques-Félix, H., 735 (elliotii), 1715 (acutiflora), 2965 (elliotii), 3039 (elliotii), 4673 (aethiopica), 8724 (elliotii), 9110 (elliotii).

Jaeger, P., 2018 (aethiopica), 7616 (aethiopica), 9283 (quintasii).

Jansen, J. W. A., 1874 (aethiopica), 2184 (aethiopica), 2219 (aethiopica), 2255 (dinklagei), 2281 (aethiopica), 2433 (aethiopica).

Jenkins 1 (odoratissima).

Johnson, D. M., [& H. J. Ndangalasi] 1884 (mwasumbii), [& L. B. Mwasumbi] 1899 (mwasumbii), 1890 (arenaria), [& J. Swedi] 1906 (collina), [& J. Swedi] 1910 (collina), [& J. Swedi] 1913 (collina), [& J. Swedi] 1914 (collina), 1920 (mwasumbii), 1924A (arenaria), 1928 (mwasumbii), [& L. B. Mwasumbi] 1936 (mwasumbii), [& N. A. Murray] 1937 (arenaria), [& N. A. Murray] 1938 (holtzii), [& H. J. Ndangalasi] 1948 (mwasumbii), [et al.] 1943A (aethiopica), [et al.] 1943B (aethiopica), [et al.] 1944 (aethiopica), [& F. M. Mbago] 1963 (mwasumbii), 1964B (mwasumbii).

Jolly 287 (aethiopica).

Jones, A. P. D. & C. F. Onochie FHI 17012 (quintasii).

Jongkind, C. C. H., [et al.] 1301 (piratae), [& F. J. Breteler] 1329 (thomsonii), [et al.] 1783 (quintasii), [& Nieuwenhuis] 3130 (longipetala), [et al.] 4218 (quintasii), 5544 (letestui), [et al.] 7407 (aethiopica), [et al.] 7818 (aethiopica), [et al.] 8821 (letestui).

Jordan, H. D., 2024 (staudtii), 2063 (elliotii).

Junod, H. A., 1427 (gracilipes).

Kandanda 11 (odoratissima).

Kayombo 1589 (holtzii).

Kenfack, D. 1187 (quintasii).

Ken’ichi Masni [Masui?] 2-20 (tanganyikensis).

Kennedy, J. D., 169 (thomsonii), 415 (villosa), 417 (villosa), 702 (quintasii), 1319 (quintasii), 1543 (quintasii), 1662 (quintasii), 1960 (rubescens), 1983 (villosa), 2050 (rubescens), 2059 (rubescens), 2172 (aethiopica), 2270 (thomsonii), 2574 (villosa), 2612 (phloiodora), 2752 (rubescens).

Kersting I 84.a (elliotii), A.181 (aethiopica), A.567 (elliotii).

Kibure 320 (holtzii), [et al.] 1019 (arenaria).

King, H. C., 158 (letestui).

Klaine, T.-J., 176 (aethiopica), 186 (rubescens), 287 (aethiopica), 310 (aethiopica), 334 (thomsonii), 719 (rubescens), 951 (aethiopica), 1327 (rubescens), 2047 (thomsonii), 2680 (thomsonii).

Koechlin, J., 1112 [= IEC 2048] (wilwerthii), 5299 [10218?] (rubescens).

Kokwaro, J. O., 3951 (arenaria).

Kruif, A. P. M. de, E 20 (rubescens), 57 (rubescens).

Laffitte, La Colonel, 92 (aethiopica).

Lamont 27984 (gracilipes).

Lane-Poole, C. E., 123 (quintasii), 210 (quintasii), 319 (aethiopica), 347 (longipetala).

Latilo, M. G., FHI 40346 (villosa).

Lawesson, J. E., & A. Goudiaby 7079 (longipetala).

Lawton, R. M., RML768 (katangensis), RML830 (katangensis), 2178 (shirensis).

Le Testu, G., 257 (longipetala), 1179 (unguiculata), 1446 (unguiculata), 1514 (unguiculata), 1748 (staudtii), 1749 (hypolampra), 1760 (letestui), 1877 (aethiopica), 1903 (unguiculata), 1913 (staudtii), 2006 (congolensis), 2023 (hypolampra), 2178 (aethiopica), 2446 (longipetala), 2605 (thomsonii), 2613 (aethiopica), 2681 (thomsonii), 3787 (elliotii), 3998 (rubescens), 4617 (thomsonii), 4635 (aethiopica), 4702 (thomsonii), 5975 (letestui), 6046 (letestui), 7824 (mildbraedii), 7937 (cupularis), 7960 (aethiopica), 8094 (hypolampra), 8630 (staudtii), 8742 (mildbraedii), 9019 (rubescens), 9025 (thomsonii), 9095 (rubescens), 9208 (unguiculata), 9287 (staudtii), 9438 (quintasii).

Le Thomas, A., 23 (pynaertii).

Leal et al. 634 (aethiopica).

Lebrun, J., 806 (aethiopica), 922 (aurantiiodora), 1014 (phloiodora), 1246 (rubescens), 1425 (staudtii), 1792 (longipetala), 1964 (aethiopica), 2110 (longipetala), 2167 (aurantiiodora), 6117 (aethiopica), 6403 (phloiodora), 6447 (acutiflora), 6576 (thomsonii), 6599 (thomsonii).

Leclercq, F., 346 (thomsonii).

Lecomte, H., 68 (aethiopica).

Leemans 488 (aethiopica).

Leeuwenberg, A. J. M., 2365 (piratae), 2406 (quintasii), 3073 (letestui), [& A. G. Voorhoeve] 4924 (aethiopica), 5217 (thomsonii), 5504 (aethiopica), 6815 (villosa), 7033 (longipetala), 8022 (piratae), 8089 (piratae), 8362 (mildbraedii), 11276 (quintasii), [& Louis] 12304 (piratae), 12424 (aethiopica), [& J. G. M. Persoon] 13614 (aethiopica), [& J. G. M. Persoon] 13701 (aethiopica).

Lejoly, J., 2915 (aurantiiodora).

Léonard, A., 247 (acutiflora), 516 (aurantiiodora), 4713 (*Xylopia* sp.), 4877 (staudtii).

Léonard, J., 311 (aethiopica), 980 (aethiopica), 3759 (aethiopica), 3783 (*Xylopia* sp.), 3837 (aethiopica).

Letouzey, R., SRFK 1374 (aurantiiodora), 1611 (rubescens), 2261 (longipetala), 2459 (longipetala), 3261 (thomsonii), 3433 (aethiopica), 4464 (rubescens), 4498 (katangensis), 5027 (phloiodora), 5139 (hypolampra), 5401 (phloiodora), 5402 (paniculata), 5404 (staudtii), 5456 (aethiopica), 5510 (quintasii), 7563 (thomsonii), 8178 (staudtii), 8186 (aethiopica), [leg. Mpom Benoit] 8552 (thomsonii), 9524 (villosa), 9614 (longipetala), 10306 (calva), [& J. F. Villiers] 10418 (phloiodora), 10701 (gilbertii), 11773 (aethiopica), 11801 (cupularis), 11854 (staudtii), 11865 (staudtii), 11876 (gilbertii), 12026 (phloiodora), 12138 (longipetala), 12317 (pynaertii), 12797 (thomsonii), 13300 (africana), 13327 (quintasii), 14551 (africana).

Lindeman 440 (shirensis), 788 (shirensis).

Linder, D. H., 66 (staudtii), 881 (aethiopica), 910 (longipetala), 1017 (longipetala), 1115 (longipetala).

Lisowski B-7179 (aethiopica), 47959 (aurantiiodora), 52352 (aurantiiodora).

Lissouba, G., 22 (rubescens).

Lötter, M., 261 (gracilipes), 269 (gracilipes), 284 (gracilipes), [& Turpin] 1882 (collina), [& Turpin] 1898 (aethiopica).

Louis, A. M., 97 (thomsonii), [et al.] 350 (quintasii), [et al.] 798 (aethiopica), [& Reitsma] 2006 (aethiopica), 2239 (thomsonii), [et al.] 2618 (thomsonii), [& A. Moungazi] 3293 (aethiopica), 3304 (quintasii), [et al.] 3419 (hypolampra).

Louis, J., 733 (gilbertii), 1007 (phloiodora), 1203 (aethiopica), 1842 (thomsonii), 2147 (aethiopica), 2301 (phloiodora), 2393 (cupularis), 2477 (phloiodora), 2741 (gilbertii), 2772 (phloiodora), 2804 (phloiodora), 2848 (aethiopica), 3909 (phloiodora), 4309 (cupularis), 5804 (aurantiiodora), 6324 (gilbertii), 6777 (gilbertii), 7220 (phloiodora), 7522 (cupularis), 7839 (aurantiiodora), 7886 (katangensis), 8542 (aurantiiodora), 8962 (*Xylopia* sp.), 9099 (aethiopica), 9374 (katangensis), 9984 (*Xylopia* sp.), 10543 (aurantiiodora), 10671 (aurantiiodora), 10790 (aurantiiodora), 11151 (katangensis), 11426 (longipetala), 13430 (phloiodora), 13464 (aurantiiodora), 15674 (aurantiiodora), 16550 (cupularis), 16985 (rubescens).

Ludanga 1166 (holtzii), 1193 (holtzii).

Luke, Q., [& Robertson] 318 (arenaria), [& Robertson] 326 (holtzii), [& Robertson] 508 (holtzii), [& Robertson] 1008 (arenaria), [& Robertson] 1686 (arenaria), [& Robertson] 2134 (arenaria), [& Robertson] 2709 (aethiopica), [& Robertson] 2723 (keniensis), 3378 (aethiopica), [et al.] 10039 (collina), [et al.] 10166 (lukei), [et al.] 11858 (africana), 13606 (africana), [& P. Luke] 13760 (lukei), [& P. Luke] 13794 (lukei), [& P. Luke] 13884 (tenuipetala), [& P. Luke] 13949 (keniensis), [& P. Luke] 13950 (keniensis).

Lynes, H., 351a (tomentosa).

Maclaud, Dr., 189 (aethiopica).

Madsen, J. E., et al., 2970 (longipetala).

Maesen, L. J. G. van der, et al., 5790 (hypolampra).

Magogo 618 (mwasumbii), [& Innes] RRI 471 (gracilipes).

Mahieu 53 (wilwerthii), 144 (cupularis), 206 (aurantiiodora), 290 (toussaintii), 292 (hypolampra), 293 (hypolampra), 295 (wilwerthii).

Maitland 233 (africana), 1731 (thomsonii), 1922 (aethiopica).

Malaisse, F., [& Kisimba Kibuye] 117 (rubescens), 6127 (rubescens), 9667 (katangensis), 13134 (shirensis), 13239 (*Xylopia* sp.), 14705 (aethiopica), [& Claes] 14911 (aethiopica).

Mambo, P., & D. W. Thomas 97 (thomsonii).

Mann, G., 485 (aethiopica), 914 (acutiflora), 1193 (africana).

Manolo 183 (shirensis).

Marabo 1546 (cupularis).

Marques, S., 165 (tomentosa).

Martin, J. D., 762 (tomentosa).

Martineau 227 (letestui), 302 (staudtii).

Matton 11 (cupularis).

Mayer, K. R., 97 (staudtii), 150 (rubescens).

Mbarga Apollonaire 58 (SRFK 1940) (cupularis).

Mbenkum, T. F., 310 (cupularis).

McDonald & Ismail 3526 (elliotii).

McFerren, D. C., 30 (odoratissima).

McKey, D., 28 (aethiopica).

McPherson, G., 13712 (aethiopica), 13763 (hypolampra), 13766 (quintasii), 13825 (pynaertii), 14003 (staudtii), 15113 (staudtii), 15168 (aethiopica), 15432 (quintasii), 15526 (thomsonii), 15698 (quintasii), 16058 (phloiodora), 16070 (mildbraedii), 16960 (unguiculata), 16960A (unguiculata), 17911 (longipetala).

Medler 201 (longipetala).

Meer, P. P. C. van, 74 (aethiopica), 184 (aethiopica), 250 (longipetala), 390 (acutiflora), 882 (aethiopica), 985 (rubescens), 1768 (africana).

Meikle, R. D., 757 (longipetala).

Meiren, T. van der, 70 (phloiodora).

Mendes 2086 (odoratissima).

Mendonça, F. de A., 4442 (aethiopica).

Mhoro 2917 (holtzii).

Michelmore, A. P. G. 1045 (shirensis).

Michelson 881 (phloiodora).

Mildbraed, J., 4003 (quintasii), 5183 (hypolampra), 6055 (mildbraedii), 6090 (phloiodora), 7592 (phloiodora), 7613 (quintasii), 7618 (hypolampra), 7716 (thomsonii), 8171 (staudtii), 8294 (quintasii), 8422 (thomsonii), 8649 (cupularis), 8826 (aethiopica), 8827 (hypolampra), 9069 (thomsonii), 9289 (thomsonii), 10629 (cupularis).

Miller B 132 (odoratissima), 133D (shirensis).

Milne-Redhead, E., 927 (tomentosa), 951 (tomentosa), 2528 (tomentosa), 2883 (aethiopica), 3887 (tomentosa), [& P. Taylor] 7617 (collina).

Monteiro, R., & F. C. Murta 181 (flamignii), 227 (flamignii).

Morel, J., 63 SRF (aethiopica), 106 (pynaertii).

Mshasha 169 (odoratissima).

Murta, F. C., 43 (hypolampra).

Mwangoka, M. A., [& A. Saidi] 2099 (mwasumbii), [et al.] 5801 (collina), [& E. Mulungu] 7959 (arenaria).

Mwiga et al. 174 (shirensis).

Myers, J. G., 13586 (rubescens), 13598 (rubescens).

Nauman 92 (aethiopica).

Nek, van, 300 (aethiopica), 345 (aethiopica).

Nemba, J., [& D. W. Thomas] 293 (quintasii), et al. 812 (aethiopica).

Nere, de, 404 (rubescens), 1710 (longipetala).

Ngok Banak, L., et al. 1057 (gilbertii).

Nguema 341 (letestui).

Nishida, T., 51 (tanganyikensis), 57 (tanganyikensis).

Nkongmeneck, B. A., 399 (aethiopica), 580 (africana).

Nolde, B., 216 (aethiopica), 305 (*Xylopia* sp.), 845 (rubescens).

Obama, C., 954 (staudtii).

Obermeyer, A. A., 839 (gracilipes).

Oldeman, 514 (aethiopica), 682 (piratae).

Okafor, J. C., & M. G. Latilo FHI 57285 (quintasii).

Onana, J.-M., 1825 (africana), 1835 (africana).

Onochie, C. F. A., FHI 33383 (quintasii).

d’Orey, J. 264 (aethiopica).

Oyayomi et al. OFOOA:127 (FHI 79827) (longipetala).

Pardy 4502 (odoratissima), 4805 (odoratissima).

Paroisse, M., 7 (aethiopica), 54 (aethiopica).

Patel, 977 (shirensis), [& Tawakali] 1028 (shirensis), [& Usi] 4605 (shirensis).

Paulo 118 (arenaria).

Pauwels, L., 1242 (tomentosa), 2961 (tomentosa), 2972 (tomentosa), 3391 (flamignii), 3411 (aethiopica), 3478 (wilwerthii), 3617 (aethiopica), 3760 (aethiopica), 3936 (aethiopica), 4583 (longipetala), 4877 (aethiopica), 5774 (longipetala).

Pawek, J., 11992 (shirensis), 13379 (shirensis), 13659 (shirensis), 13660 (shirensis).

Pedro 4394 (gracilipes), [& Pedrogão] 5279 (collina), [& Pedrogão] 5285 (aethiopica).

Perdue & Kibuwa 10046 (arenaria).

Perrottet 7 (longipetala), 9 (aethiopica).

Philip, M. S., 931 (nilotica).

Pierlot 1707 (aethiopica).

Pobéguin, M., 48 (longipetala), 89 (aethiopica), 255 (aethiopica), 849 (aethiopica), 1529 (elliotii), 2130 (elliotii).

Pocock, M. A., 445 (tomentosa).

Poisson, M. E., 36 (longipetala).

Prignon 4 (katangensis).

Procter 558 (shirensis).

Punch, C., 119 (villosa).

Pynaert, L. A. E. J., 567 (pynaertii).

Quarré 6201 (aethiopica).

Quintas, F., 3 (quintasii), 1083 (quintasii).

Raynal, A., 13652 (aethiopica).

Raynal, J., & A. Raynal 10130 (thomsonii), 12228 (elliotii).

Reitsma, J. M., [& B. Reitsma] 855 (aethiopica), [et al.] 1071 (staudtii), [& B. Reitsma] 1156 (pynaertii), [& B. Reitsma] 1163 (paniculata), [& B. Reitsma] 1168 (staudtii), [& B. Reitsma] 1401 (unguiculata), [& B. Reitsma] 1423 (quintasii), [& B. Reitsma] 1442 (aethiopica), [& B. Reitsma] 1621 (pynaertii), [et al.] 1821 (gilbertii), [& B. Reitsma] 1923 (unguiculata), [& B. Reitsma] 2318 (longipetala), [& B. Reitsma] 2342 (quintasii), [& B. Reitsma] 2622 (cupularis), [& B. Reitsma] 2816 (mildbraedii), [& B. Reitsma] 2862 (thomsonii), [& B. Reitsma] 2900 (phloiodora).

Renny, A. T., 253 (gracilipes).

Richards, Mrs. H. M., 1881 (shirensis), 2326 (shirensis), 3696 (rubescens), 4036 (rubescens), 6833 (shirensis), 13266 (shirensis), 14438 (shirensis), 17104 (shirensis), 19193 (rubescens), 31484 (shirensis).

Richards, P. W., 3159 (quintasii), 3334 (quintasii), 3434 [= Ross 3434] (quintasii).

Risopoulos, S., 531 (rubescens).

Robertson, R. G., 184 (rubescens).

Robertson, S. A., [et al.] MDE 50 (holtzii), 3727 (arenaria), [& Q. Luke] 6000 (arenaria), [& Q. Luke] 6063 (arenaria), [& R. Brummitt] 6752 (arenaria), 6957 (holtzii).

Robson, N. K. B., 770 (shirensis), [& Steele] 1628 (shirensis).

Robyns, W., 287 (longipetala), 465 (aurantiiodora), 580 (aurantiiodora), 4394 (wilwerthii).

Rodgers, W. A., 388 (holtzii), 1148 (holtzii).

Rose 386 (aethiopica).

Ross, A. F., R.202 (calva), 236 (villosa).

Ross, R., 68 (quintasii).

Ruffo 301 (mwasumbii), [& Mmari] 1855 (holtzii), [& Kisena] 3235 (rubescens).

Sabatie, B., 15 (elliotii), 687 (elliotii).

Saint Aubin, de, SRF 1972 (hypolampra), 2082 (hypolampra).

Sanane 346 (shirensis), 1293 (rubescens).

Santos 92 (odoratissima).

Sargos 60 (aethiopica), 65 (rubescens), 126 (aethiopica), 141 (Bois N 941) (hypolampra), 206 (aethiopica), 223 (aethiopica), 242 (quintasii).

Schijff, H. P. v. d., 969 (gracilipes), 3779 (gracilipes), 5686 (gracilipes).

Schlieben, H. J., 1480 (aethiopica), 5470 (collina), 5571 (gracilipes).

Schmitt, K., et al. 429 (aethiopica), 2267 (quintasii).

Schmitz, A., 3176 (rubescens), 4748 (katangensis), 5764 (aethiopica), 5771 (rubescens), 6188 (tomentosa).

Schnell, R., 2681 (aethiopica).

Schnetler, F., 8177 (gracilipes).

Schoenmaker, J., 306 (aethiopica).

Schweinfurth, G., 2527 (longipetala), 3032 (thomsonii), 3234 (thomsonii).

Scott-Elliot, G. F. 4733 (longipetala), 5288 (elliotii), 5325 elliotii), 5328 (elliotii), 5730 (longipetala).

Semsei, S. R., 1718 (holtzii), 3704 (mwasumbii), 51285 (arenaria).

Seret, F., 555 (thomsonii).

Service forestier (Côte d’Ivoire) 458 (quintasii).

Service forestier du Cameroun 60 (quintasii), 67 (phloiodora), 1112 (hypolampra).

Shabani [for Bullock] 10 (shirensis), 43 (collina), 1131 (shirensis).

Sheil, D., 1443 (nilotica).

Sillitoe, F., 353 (thomsonii), 479 (thomsonii).

SIMAB 012002 (letestui), 020102 (aethiopica).

Simon Laizer, G., [et al.] 1352 (collina).

Simpson, N. D., 7778 (nilotica).

Sita, P., 1232 (toussaintii), 1634 (phloiodora), 2737 (flamignii), 3766 (pynaertii), 3813 (thomsonii), 4081 (letestui), 4118 (cupularis).

Small, D., 605 (aethiopica), 639 (acutiflora), 664 (acutiflora), 669 (quintasii), 724 (staudtii).

Smith, G., 39 (nilotica).

Sosef, M. S. M., [et al.] 1167 (acutiflora), 1759 (aethiopica), 1830 (aethiopica), 1866 (hypolampra), 2180 (globosa), 2218 (phloiodora).

Soyaux, H., 131 (aethiopica).

SRFK 1913 (quintasii).

Stalmans, M., 285 (gracilipes).

Stäuble NS 0460 (acutiflora).

Staudt, A., 504 (acutiflora), 530 (staudtii).

Stévart, T., et al. 4642 (quintasii).

Stevenson 265/31 (katangensis).

Stirbo 126 (aethiopica).

Stoffelen, P., 453 (aurantiiodora).

Stone, J. [et al.] 3122 (longipetala), [et al.] 3171 (hypolampra), [& Niangadouma] 3664 (aethiopica).

Stopp, K., M79 (gracilipes).

Strid, A., 2370 (tomentosa).

Stuhlmann, F. L., 1233 (aethiopica), 2781 (thomsonii).

Szafranski, F., 1218 (longipetala).

Talbot, P. A., 226 (aethiopica), [& Mrs. Talbot] 1302 (quintasii), [& Mrs. Talbot] 1353 (thomsonii), 1486 (thomsonii), 1601 (talbotii), [& Mrs. Talbot] 3209 (talbotii), [& Mrs. Talbot] 3267 (thomsonii), 3281 (aethiopica), [& Mrs. Talbot] 3775 (villosa).

Tchinaye, V., 103 (longipetala), 113 (thomsonii).

Tchouto et al. 1756 (cupularis).

Tenejong FHI 14346 (longipetala).

Tessmann, G., 160 (rubescens), 644 (staudtii), 747 (talbotii), 760 (quintasii), 913 (aethiopica).

Thoiré, M., 85 (aethiopica).

Thollon, M., 144 (longipetala), 743 (longipetala), 4026 (rubescens).

Thomas, D. W., 595 (thomsonii), 2981 (africana), 3177 (aethiopica), 3204 (talbotii), 3242 (thomsonii), 3305 (africana), 3463 (staudtii), 4554 (africana), [et al.] 4558 (monticola), [& C. M. Wilks] 6515 (staudtii), [et al.] 7400 (monticola), [et al.] 7401 (staudtii), [& J. M. Fay] 7281 (thomsonii), [et al.] 8746 (aethiopica).

Thomas 144 (longipetala).

Thomson, A. P., 2 (staudtii).

Thomson, W. C., 53 (rubescens), 63 (thomsonii).

Timberlake, J., et al. 5570 (tenuipetala), 5640 (collina).

Tinley 1414 (odoratissima), 2382 (gracilipes).

Tisserant, C., 138 (cupularis), 246 (cupularis), 315 (cupularis), 337 (aethiopica), 476 (thomsonii), 477 (longipetala), 527 (quintasii), 625 (gilbertii), 791 (aethiopica), 934 (longipetala), 934 bis (longipetala), 936 (aurantiiodora), 955 (phloiodora), 1113 (phloiodora), 1138 (phloiodora), 1246 (hypolampra), 1287 (cupularis), 1385 (hypolampra), 1436 (aethiopica), 1470 (thomsonii), 1768 (cupularis), 1778 (elliotii), 1786 (quintasii), 1885 (rubescens), 2295 (cupularis), 2329 (gilbertii), 3068 (thomsonii), 3640 (longipetala).

Torre, A. R., 2350 (torrei), 3944 (torrei), 6192 (gracilipes), [& Paiva] 9413 (gracilipes), [& M. F. Correia] 14168 (gracilipes), 16453 (aethiopica), [& Correia] 16460 (aethiopica), [& Correia] 16681 (gracilipes).

Toussaint, L., 373 (toussaintii), 2068 (gilbertii), 2114 [tag 2074] (wilwerthii), 2187 (cupularis), 2241 (toussaintii), 2395 (hypolampra), 2430 (aethiopica), 2451 (gilbertii).

Trochain, J., 9603 [= IEC 6451] (wilwerthii).

Troupin, G., 2104 (rubescens).

Trump, E. C., 96 (arenaria).

Turner, L., 241 (nilotica).

Tutin, C., 80 (congolensis).

Uehara, S., 580 (tanganyikensis).

Vahrmeijer & du Preez 2508 (odoratissima).

Van Andel, T., et al., 3318 (*Greenwayodendronsuaveolens*), 3345 (quintasii), 4023 (acutiflora).

Verdick, E. A. A., 503 (katangensis).

Verger, P., 913 (aethiopica).

Vermoesen 2315 (aethiopica).

Versteegh & den Outer 97 (aethiopica), 359 (longipetala).

Vigne 81 (villosa), 856 (quintasii), 982 (staudtii), 2561 (villosa), 3883 (longipetala).

Villiers, J. F., 666 (longipetala).

Vollesen, K., 2992 (holtzii), 4619 (holtzii).

Voorhoeve, 19 (staudtii), 144a (quintasii), 910 (quintasii), 1089 (acutiflora).

Vroumsia Tchinaye 103 (longipetala).

Vuillet 68 (elliotii).

Walker, A., 3454 (hypolampra).

Walters et al. 465 (aethiopica), 1079 (aethiopica).

Warmelo, N. J. van, 5159/14 (gracilipes), 31838 (gracilipes), 51219/7 (gracilipes).

Waterman & D. McKey 872 (thomsonii).

Welwitsch 92 (odoratissima), 757 (odoratissima), 764 (africana), 765 (aethiopica).

Westphal, E., [& J. M. C. Westphal-Stevels] 10047, 10048 10048, 10172 (all elliotii), 10202 (aethiopica).

Wheatley, J. I., 605 (africana).

White, F., 2062 (odoratissima—BM and K sheets, MO sheet not *Xylopia*), 2071 (tomentosa), 2079 (tomentosa), 3156 (shirensis), 3165A (rubescens), 3182 (rubescens), 3309 (rubescens), 3610 (katangensis), 3711 (shirensis).

White, L. J. T., [series 2] 29 (aethiopica), [series 2] 154 (aethiopica), 239 (quintasii), 345 (quintasii), 371 (cupularis), [SEGC] 444 (quintasii), 471 (aethiopica), 483 (hypolampra), 489 (cupularis), 0944 (longipetala), 960 (congolensis), 1530 (quintasii).

Wieringa, J. J., 485 (aethiopica), [& H. M. van de Poll] 1506 (aethiopica), [et al.] 2884 (thomsonii), [et al.] 3031 (aethiopica), 4514 (aethiopica), 5019 (staudtii), 5138 (staudtii), 5233 (aethiopica).

Wigg, F. H., 523 (shirensis), 1121 (shirensis).

Wild 5267 (aethiopica), [et al.] 6618 (aethiopica), [et al.] 6651 (gracilipes).

Wilde, J. J. de, [et al.] 9040 (aethiopica), [et al.] 9156 (quintasii), [& C. Jongkind] 9393 (thomsonii), [et al.] 9740 (thomsonii).

Wilde, J. J. F. E. de, [et al.] 220 (africana), [et al.] 430 (aethiopica), [et al.] 785 (thomsonii), [et al.] 804 (aethiopica), 982 (piratae), 3236 (longipetala), [& A. G. Voorhoeve] 3633 (aethiopica), 3833 (acutiflora), 7871 (quintasii), 7889 (quintasii), 7931 (aethiopica), 7941 (staudtii), 7963 (hypolampra), 7970 (aethiopica), 8029 (staudtii), 8085 (aethiopica), 8166 (rubescens), 8707 (aethiopica), [& R. W. de Wilde-Bakhuizen] 11172 (aethiopica), [& R. W. de Wilde-Bakhuizen] 11194 (thomsonii), [& R. W. de Wilde-Bakhuizen] 11959 (longipetala), [et al.] 12161 (staudtii).

Wilde, W. J. J. O. de, [& B. E. E. de Wilde-Duyfjes] 164 (longipetala), 354 (piratae), 356 (piratae), [& B. E. E. de Wilde-Duyfjes] 497 (piratae), [& B. E. E. de Wilde-Duyfjes] 1320 (quintasii), 1322 (aethiopica), [& B. E. E. de Wilde-Duyfjes] 2043 (thomsonii), [& B. E. E. de Wilde-Duyfjes] 2172 (thomsonii), [& B. E. E. de Wilde-Duyfjes] 2676 (longipetala), [& B. E. E. de Wilde-Duyfjes] 2703 (thomsonii), [& B. E. E. de Wilde-Duyfjes] 2838A (phloiodora), [& B. E. E. de Wilde-Duyfjes] 2838B (phloiodora), [& B. E. E. de Wilde-Duyfjes] 3994 (thomsonii).

Wilks, C., WIL 922 (aethiopica), WIL 991 (staudtii), WIL 1040 (hypolampra), 1536 (quintasii), 1890 (aethiopica), [et al.] 2107 (quintasii), 2610 (aethiopica).

Wit, H. C. D. de, [& Morton] 7947 (africana), 7952 (thomsonii), 7999 (piratae).

Wood, G. H. S., 610 (nilotica).

Wyk, P. van, BSA 2023 (odoratissima).

Yafunga, F. 3 (aethiopica), 7 (gilbertii), F. 60 (thomsonii).

Young, R. G. N., 1306 (tomentosa).

Zenker, G. A., 246 [= 2093?] (staudtii), 359 (quintasii), 408 (quintasii), 499 (aethiopica), 580 (quintasii), 2080 (quintasii), 2094 [= 408?] (quintasii), 2095 [= 580?] (quintasii), 2112 (aethiopica), 2655 (quintasii), 2663 (quintasii), 2827 (rubescens), 3289 (*Uvariastrumzenkeri* Engl. & Diels (Couvreur 2014) [distributed as *Xylopia* sp.]), 3314 (phloiodora, mixed with staudtii), 3653 (staudtii), 3953 (staudtii), 4096 (quintasii), 4098 (*Greenwayodendronsuaveolens*), 4738 (quintasii), 4739 (*Greenwayodendronsuaveolens*), 4747 (calva), 4862 (staudtii).

Zimba, N. B., et al., 739 (tomentosa), 829 (odoratissima).

Zimmerman 2630 (aethiopica).

Zon, A. P. M. van der, 2105 (elliotii).

## Supplementary materials

Supplementary material 1 KML files Data type: occurence Explanation note: KML files for African *Xylopia* species distributions. https://binary.pensoft.net/file/199134This dataset is made available under the Open Database License (http://opendatacommons.org/licenses/odbl/1.0/). The Open Database License (ODbL) is a license agreement intended to allow users to freely share, modify, and use this Dataset while maintaining this same freedom for others, provided that the original source and author(s) are credited.David M. Johnson, Nancy A. Murray
